# Transcriptomic and phylogenetic analysis of a bacterial cell cycle reveals strong associations between gene co-expression and evolution

**DOI:** 10.1186/1471-2164-14-450

**Published:** 2013-07-05

**Authors:** Gang Fang, Karla D Passalacqua, Jason Hocking, Paula Montero Llopis, Mark Gerstein, Nicholas H Bergman, Christine Jacobs-Wagner

**Affiliations:** 1 Department of Molecular, Cellular and Developmental Biology, Yale University, New Haven, CT 06511, USA; 2 Department of Molecular Biophysics and Biochemistry, Yale University, New Haven, CT 06511, USA; 3 School of Biology, Georgia Institute of Technology, Atlanta, GA 30332, USA; 4 Howard Hughes Medical Institute, Yale University, New Haven, CT 06511, USA; 5 Program in Computational Biology and Bioinformatics, Yale University, New Haven, CT 06511, USA; 6 Department of Microbial Pathogenesis, Yale School of Medicine, New Haven, CT 06511, USA

**Keywords:** Cell cycle phylogenomics, *Caulobacter crescentus*, Co-expression network, Functional modules, Selective pressure

## Abstract

**Background:**

The genetic network involved in the bacterial cell cycle is poorly understood even though it underpins the remarkable ability of bacteria to proliferate. How such network evolves is even less clear. The major aims of this work were to identify and examine the genes and pathways that are differentially expressed during the *Caulobacter crescentus* cell cycle, and to analyze the evolutionary features of the cell cycle network.

**Results:**

We used deep RNA sequencing to obtain high coverage RNA-Seq data of five *C. crescentus* cell cycle stages, each with three biological replicates. We found that 1,586 genes (over a third of the genome) display significant differential expression between stages. This gene list, which contains many genes previously unknown for their cell cycle regulation, includes almost half of the genes involved in primary metabolism, suggesting that these “house-keeping” genes are not constitutively transcribed during the cell cycle, as often assumed. Gene and module co-expression clustering reveal co-regulated pathways and suggest functionally coupled genes. In addition, an evolutionary analysis of the cell cycle network shows a high correlation between co-expression and co-evolution. Most co-expression modules have strong phylogenetic signals, with broadly conserved genes and clade-specific genes predominating different substructures of the cell cycle co-expression network. We also found that conserved genes tend to determine the expression profile of their module.

**Conclusion:**

We describe the first phylogenetic and single-nucleotide-resolution transcriptomic analysis of a bacterial cell cycle network. In addition, the study suggests how evolution has shaped this network and provides direct biological network support that selective pressure is not on individual genes but rather on the relationship between genes, which highlights the importance of integrating phylogenetic analysis into biological network studies.

## Background

Advances in next-generation sequencing methodologies have significantly reduced the time and cost constraints of determining genome-wide expression levels of various organisms, including bacteria. These technologies present major advantages over hybridization-based microarrays [1,2]. Along with high throughput, they allow single-nucleotide resolution as well as quantification of absolute RNA abundance. These benefits combined with strand-specificity and greater dynamic range in gene expression measurement have provided great insight into the transcriptional landscape of various bacteria under different growth conditions [2]. However, no deep RNA sequencing (RNA-Seq) studies have so far reported a transcriptome analysis of a bacterial cell cycle, which would provide an important step toward understanding the genetic pathways involved in bacterial multiplication.

**Figure 1:**
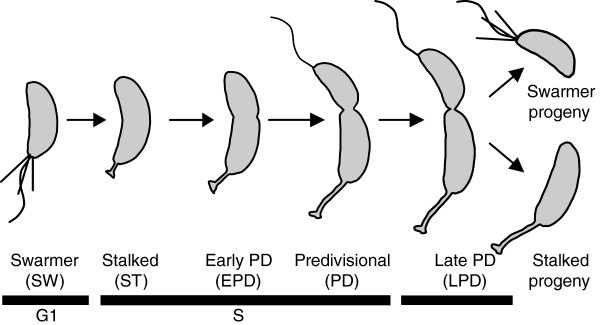
**The *C. crescentus *cell cycle.** Cartoon showing the different stages of the *C. crescentus* cell cycle: swarmer (SW), stalk (ST), early predivisional (EPD), predivisional (PD), and late predivisional (LPD). Each stage corresponds to the time point (0, 30, 60, 90 or 120 min following the cell synchrony) taken for RNA-Seq analysis.

The ease of obtaining synchronized cell populations of the Gram-negative bacterium *Caulobacter crescentus* through a physical method [3] has made this organism a prominent bacterial model for analyzing the cell cycle [4]. The cell cycle of *C. crescentus* has also generated interest because of its inherent association with a developmental process [5,6]. Each division produces two distinct daughter cells: a flagellated and piliated “swarmer” (SW) progeny and a slightly longer, stalk-containing “stalked” (ST) progeny (Figure 1). SW cells, which can be isolated from an asynchronous culture using a simple gradient centrifugation method [3], are in G1 phase as they cannot replicate their single chromosome until they grow to a similar size to their ST siblings [7]. Following flagellum ejection and pili retraction, DNA replication initiates and a polar stalk develops to produce a ST cell (Figure 1). After some growth, cell constriction is initiated and a new flagellum is built at the pole opposite to the stalk. Completion of cytokinesis followed by cell separation results in the production of the SW and ST progeny. The SW cell then reiterates the aforementioned cell cycle whereas the ST cell skips the G1 phase and initiates the S phase immediately.

Decades of single-gene studies in *C. crescentus* have uncovered regulatory components and molecular mechanisms that govern the cell cycle and the spatial and temporal biogenesis of different organelles and molecular machineries. Following the resolution of the *C. crescentus* genome [8,9], a variety of “omics” and modeling studies have been undertaken to understand the *C. crescentus* cell cycle at a system level [10-17]. Important studies have led the way to understanding the transcriptional cascades generated by the oscillatory expression of cell cycle master regulators [10,12,18-20].

In this work, we took advantage of the benefits of RNA-Seq to provide absolute measures of gene expression during the *C. crescentus* cell cycle, using biological replicates for each cell cycle stage. We uncovered novel properties of gene expression and regulation, identified over 1,500 cell cycle-regulated genes, and organized them into a co-expression network. Furthermore, we expanded phylogenomics [21] to co-expression network study by comparing network and gene evolutionary properties, and discovered strong correlations between co-expression and evolution.

## Results and discussion

### Single-nucleotide resolution whole-genome mapping of RNA-Seq

**Figure 2:**
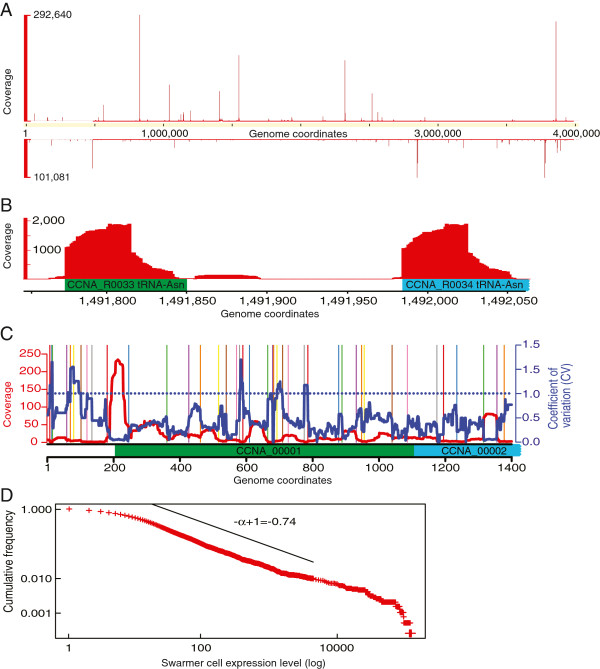
**RNA-Seq mapping results. (A)** A bird’s eye view of global RNA-Seq mapping on each strand using the SW time point as an example. Spikes are mostly non-coding RNAs (ncRNAs). **(B)** Example of a normalized RNA-Seq mapping. Two annotated tRNA-Asn are represented by the green and cyan boxes. The two DNA sequences of tRNA-Asn are identical, which leads to RNA-Seq reads being “ambiguously” mapped to two locations. Therefore, the expression value was calculated by dividing the amount of reads by two. **(C)** Scheme illustrating the gene expression quantification algorithm. RNA-Seq coverage (the red curve) and CV (blue curve) per nucleotide are plotted. The colored vertical bars border the dynamic programming segmentations based on the CV curve. In this particular case, CV is divided into 42 segments. We adopted a threshold of CV =1.0 (horizontal dotted blue line) to filter out segments with CV<1.0, allowing us to keep quality segments for gene expression quantification. **(D)** Distribution of gene expression values (using the SW cell as an example) follows a power-law distribution. The probability density *p*(*e*) ∽ *e*^- *a*^, where e is gene expression, is best fitted with α=1.74 (according to Clauset et al’s algorithm [22]). This panel shows the cumulative distribution Pr(E>e) of gene expression along with the power law fit exponent -α+1=-0.74. Genes with expression ranging from 15x to about 1000x (indicated by the slant) fall into the power-law distribution, in good agreement with a previous report [23].

To examine the cell cycle transcriptome of *C. crescentus*, cells grown in the M2G minimal medium were subjected to Ludox (percoll) density centrifugation to isolate swarmer (G1 phase) cells, which were then re-suspended in M2G medium to resume cell cycle progression synchronously. Samples were collected for RNA extraction at 5 different time points (0, 30, 60, 90, and 120 min) following synchronization, with each time point corresponding to a different cell cycle stage referred to as swarmer (SW), stalked (ST), early predivisional (EPD), predivisional (PD), and late predivisional (LPD) (Figure 1). By performing synchronies on different days, we obtained a total of three biological replicates of each cell cycle stage. The extracted RNAs were labeled in a strand-specific manner and sequenced using the SOLiD platform. In total, we obtained over 600 million (M) SOLiD RNA-Seq reads. Fifty bp-long reads were trimmed of 10 bp from the 3′ end, and 300 M of these reads were mapped on the genome of *C. crescentus* NA1000 (also known as CB15N). This resulted in a single nucleotide resolution transcriptome composed of 15 sets of mappings with a sum of 962x coverage per nucleotide. Figure 2A shows a bird’s eye view of the whole transcriptome, and Figure 2B shows the RNA-Seq mapping details of the two asparagine tRNAs, both using the SW cell stage as an example. When comparing biological replicates, we found that, in some regions, the mapping was less consistent than in others, and that the regions of low consistency were correlated with enriched GC content (data not shown), as previously reported [1]. Since the *C. crescentus* genome is GC-rich (67%), traditional quantitative methods such as calculating mean coverage on genes or RPKM (Reads Per Kilobase per Million mapped reads) may reduce the accuracy of gene expression quantification [1]. Therefore, we employed a dynamic segmentation algorithm based on coefficient of variation (CV; standard deviation/mean) analysis of the replicates to locate and discard low consistency mapping regions, and we only used the highly reliable mapping regions to calculate gene expression values (Figure 2C; see methods). The level of gene expression was then calculated as the average coverage of retained nucleotides within the gene. With this quantification method, we obtained an average coverage of 278x (mean of replicates) for gene expression. We found that the distribution of gene expression for each cell cycle stage follows a power-law distribution (Figure 2D and Additional file 1: Figure S1), in agreement with the evolutionary conserved power-law organization of genome-wide expression levels [23]. The whole transcriptome data, with raw mapping results and normalized gene expression values, are provided in Additional files 2, 3, 4: Table S1a-c.

### Genes cluster into three groups according to their expression level

**Figure 3:**
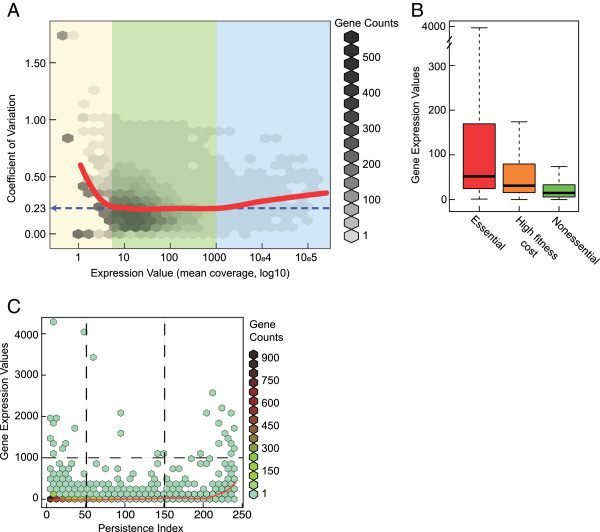
**Relationship between gene expression, essentiality and persistence. (A)** Genes are classified into three groups based on their expression levels and CV. Here data from 5 cell cycle time points were pooled for the analysis of gene expression and CV from 3 replicates. The background hexbin density plot describes the relationship between CV and expression values for all genes. The red curve is a local regression of CV values as a function of expression (using the LOESS R package). Based on this fitting, genes are classified into three groups indicated by different colors. Most genes fall into the group in the middle, where their average CV remains constant at 0.23 (the blue dotted arrow). The group on the left has, on average, high CV, and shows negative correlation between CV and expression. This group is suggested to include background transcription noise. The group on the right consisting of highly expressed genes also shows significantly higher CV. **(B)** Gene expression versus essentiality. Box and whisker plot of expressions grouped by essential, high-fitness-cost and nonessential genes. **(C)** Hexbin plot showing genes expression versus persistence. The red trend curve is a local regression of median expression versus PI (LOESS R package). The horizontal bar distinguishes the highly expressed genes (>1,000x) from the others, whereas the vertical bars delineates less conserved (PI < 50) genes from persistent (PI >150) genes.

Past RNA-Seq studies have shown that the distribution of expression levels in bacteria is continuous, without an obvious breaking point between background transcription and biologically relevant expression [24,25]. While this continuum in gene expression levels was confirmed in our study (Additional file 1: Figure S1), we found that CV analysis of replicates as a function of gene expression can identify global patterns of gene expression and regulation (Figure 3A). The CV, which is defined as the ratio of the standard deviation to the mean, was used here as a convenient way to quantify both “signal”, from regulated expression, and “noise”, from background transcriptional activity. Background transcription due to random binding of the RNA polymerase is expected to generate low amounts of RNA and to be poorly consistent across replicates, thereby generating a high CV value. Conversely, transcription that is biologically relevant should have higher expression and lower CV value, the latter because of a higher reproducibility between biological replicates. Plotting the CV values for all genes as a function of their average expression (Figure 3A) reveals that the genes fall into three groups. The first group consists of 738 genes, or almost a fifth of the genome, that have a low expression level (with the maximal expression being below 5x) and an average CV value of 0.38. In this group (expression <5x), the CV negatively correlates with expression (Figure 3A, red line). This negative correlation no longer exists for other genes with expression values above 5x. The enrichment of high CV values for genes with expression values below 5x suggests that it includes transcriptional noise. While small integers tend to generate higher CV values, gene phylogeny and essentiality analyses (see below) further support the notion that this first group primarily includes background transcription. In Figure 3A, the CV curve reaches a plateau at about 0.23 for expression values between 5x and 1000x. This plateau defines the second group of genes, which consists of 3,136 genes or 79% of the *C. crescentus* genome. The constant low CV value suggests that expressions of most genes follow the same shape of distribution and are under precise biological regulation. Interestingly, the CV curve slightly increases when the expression levels rise above 1000x. This third group of genes, which consists of 90 highly expressed genes (2.3% of the genome), had an average CV of 0.29. A statistical test (t-test, p<1e-10) confirmed that this group of genes indeed had higher CV values than the second group, which includes the majority of genes. Fifty-four of the 90 highly expressed genes are non-coding RNAs (including 48 tRNAs). When we considered these 54 RNAs alone, their average CV increased to 0.31, suggesting that these highly expressed RNAs may be under less stringent regulation than most genes; while they have strong promoters, the cells may not (or may not need to) have an efficient mechanism to maintain the precise amount of such RNA species within the cell.

A recent genome-wide transposon insertion study in *C. crescentus* has identified 480 essential or high-fitness-cost genes [14]. As shown in Figure 3B, we found that gene essentiality is correlated with gene expression as the essential genes generally had higher expression values (median = 52x) than non-essential genes (median = 15x), with the high-fitness-cost genes having intermediate values (median = 31x, ANOVA test, p<1e-10). Only 4 essential and 6 high-fitness-cost genes fell to the group of poorly expressed genes (Additional file 5: Table S2). Their essentiality was determined based on colony formation on solid rich growth medium [14]. The low expression levels of these genes under our experimental conditions (exponential-phase liquid cultures in minimal medium) suggests that their essentiality may be specific to growth conditions.

Gene essentiality as determined by laboratory mutagenesis are dependent on experimental contexts, and only identifies genes whose inactivation results in rapid lethality or high-fitness cost under the tested conditions. On the other hand, gene persistence, which measures how widely conserved a gene is among extant species [26], informs about the importance of a gene in natural environments, with competitions, under harsh conditions, and over 3 billion years of natural evolution [27]. Therefore, we also compared the gene expression levels with evolutionary gene persistence. To obtain a persistence index (PI) [26] of each *C. crescentus* gene, we first determined the distribution of orthologs among 236 bacterial species selected to represent an unbiased phylogenetic tree (see methods). The expression level of each gene was then plotted as a function of its PI (Figure 3C), with PI>150 and PI<50 used as borders to distinguish “persistent” genes that have been retained in most species during evolution (with over 150 orthologs among the 236 selected genomes) from the “less conserved” genes (with less than 50 orthologs). We found that poorly expressed genes, as a group, have been poorly conserved during evolution as among the 738 genes with low expression (<5x), 675 of them (92%) had PI<50, and only 6 poorly expressed genes had a PI>150 (Additional file 5: Table S2). When considering all genes, chi-square test clearly showed that as expected [27], the persistent genes overall display a higher expression than less conserved genes (p<1e-10). The positive correlation between expression and persistence in very broadly conserved genes (PI>200, Figure 3C) is in good agreement with the toolbox model of bacterial evolution [28]. Interestingly, however, we observed a few highly expressed (>1000x) genes that were present almost equally among both persistent and poorly conserved genes (Figure 3C). In fact, when we only examined highly expressed (>1000x) genes, there was no longer a correlation between PI values and expression levels (i.e., t-test of gene expressions from the two groups PI<50 and PI>150 shows no difference). This indicates once again that highly expressed genes tend to behave distinctly from the rest of the genome; they are under different regulatory and evolutionary constraints than most genes.

### Identification of 1,586 differentially expressed genes

To identify cell cycle-regulated (CCR) genes, we used the baySeq package. This program took the gene expression values from the biological replicates across the 5 cell cycle time points, and estimated posterior likelihoods of differential expression via an empirical Bayesian method [29]. Through this analysis (see methods), we identified 1,586 genes (Additional file 6: Table S3) that we will hereafter refer to as CCR genes. We note that a small fraction of our CCR genes are likely to be false positives due to the potential stresses (e.g., cold shock) associated with the cell cycle synchronization technique (see Additional file 7: SI and Additional file 8: Table S9). Most genes whose transcription is induced with the method are expected to display a peak expression in the first time point (i.e., the SW/G1 cell stage) with a lower expression profile in subsequent time point samples. The presence of these method-induced genes does not, however, affect our conclusions because we obtain similar results when the whole set of SW/G1-specific genes is excluded from all the analyses (including those described below, see Additional file 7: SI).

**Figure 4:**
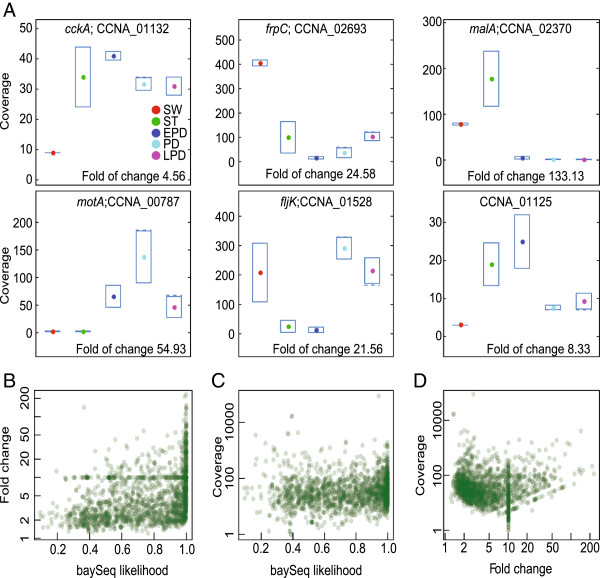
**Cell cycle regulation. (A)** Examples of expression profiles across the 5 cell cycle time points (red, SW; green, ST; dark blue, EPD; light blue, PD; and purple, LPD). **(B)** Plot showing fold changes in expression during the cell cycle as a function of the baySeq likelihood. Each spot represents a gene. **(C)** Plot showing peak expression levels as a function of the baySeq likelihood. The dotted line is due to the log scale. **(D)** Plot showing peak expression levels as a function of fold changes in expression during the cell cycle. The dotted line is due to the log scale.

A variety of cell cycle expression patterns were observed among the 1,586 CCR genes (see Figure 4A for 6 examples, and Additional file 9: Figure S2 for all the other CCR genes). For verification, we used 47 experimentally identified CCR genes as a gold reference (Additional file 10: Table S4). All of these genes were correctly assigned as CCR genes in our analysis. We also compared our list of CCR genes with two previously reported CCR gene sets obtained from DNA microarray studies that used the same synchronization technique [10,11]. These two sets include 551 and 433 genes, with an overlap of 138 genes. The reason of the relatively small gene overlap between these two sets is unclear and may be attributed to differences in methods used, or to a lack of experimental replicates in these studies. Combining these two CCR gene lists results in a set of 846 genes, and 543 (64%) of them are reported in our new CCR list. Importantly, because our study includes biological replicates, the baySeq likelihood value from 0 to 1 provides a measure of confidence in cell cycle expression for each CCR gene (Additional file 6: Table S3). This information is useful because, while there is a positive correlation between the fold of change in expression and the likelihood, small differences in expression level during the cell cycle can be associated with high likelihood values (Figure 4B), indicating that they are highly reliable.

In general, the level of peak gene expression does not appear to influence the baySeq likelihood values (Figure 4C). Among the 1,586 CCR genes that we identified, 84% (1,331) of them had expression changes > 2 fold (Figure 4D). The maximal fold of change in cell-cycle expression was over 229, and the mean was 8.2. In terms of peak expression, 96% (1,521) CCR genes had coverage > 5x (Figure 4D) and therefore, are likely above background transcription.

**Figure 5:**
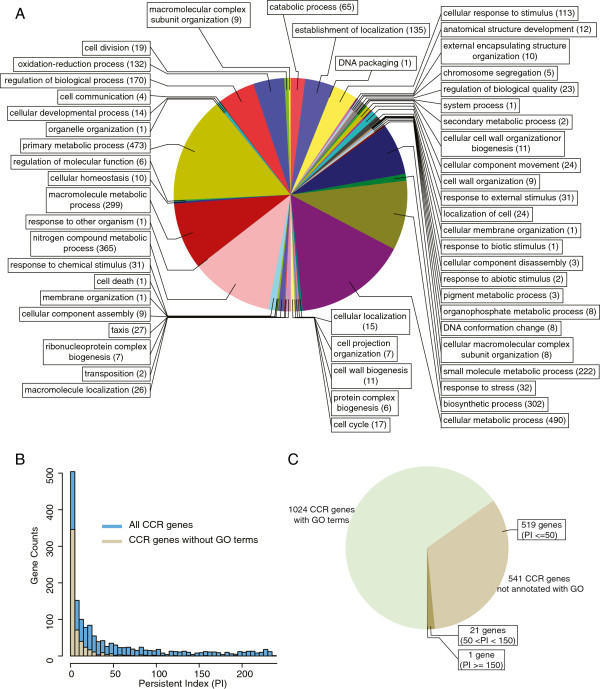
**Gene ontology analysis. (A)** Pie chart showing the GO term distribution at the biological process level 3 among the 1,024 CCR genes with GO terms. **(B)** Distributions of PI values for all protein-encoding CCR genes and for protein-encoding CCR genes without GO terms. **(C)** Pie chart showing CCR genes with and without GO terms. Genes without GO terms were discriminated based on their PI.

Among the CCR genes, 21 were annotated non-coding RNAs (ncRNA) (Additional file 6: Table S3). For example, the expression of CCNA_R0092 varies by 23-fold during the cell cycle, with a peak expression of ~1550x (Additional file 9: Figure S2, Additional file 6: Table S3). The remaining CCR genes (1,565) were predicted to encode proteins whose ontology we surveyed. Using the UniProt-GOA data set, which includes 2,564 *C. crescentus* NA1000 genes [30], we obtained the gene ontology (GO) annotation for 1,024 protein-encoding CCR genes (Figure 5A, Additional file 6: Table S3). In a previous microarray study, 101 metabolism-related genes had been reported to change their expression during the *C. crescentus* cell cycle [10]. In our CCR gene dataset, 473 genes were assigned under primary metabolic process category, and 490 genes were annotated as cellular metabolic process. These two GO terms included a total of 541 CCR genes, indicating that over one third of all CCR genes are related to metabolic functions. A total of 1,337 genes of the *C. crescentus* genome are classified under primary and cellular metabolic processes based on UniProt-GOA [30]. Thus, over 40% of them display differential cell cycle expression under our conditions. This is surprising as metabolic genes are often thought of as housekeeping genes and as such, are expected to be constitutively expressed during the cell cycle. Their cell cycle regulation suggests potential fluxes of primary metabolites during the cell cycle.

GO term enrichment analysis that compares CCR with non-CCR genes revealed over-representations and under-representations of genes with particular GO terms (Additional file 11: Figure S3, Additional file 12: Table S5). For example, genes associated with flagellar motility, chemotaxis, division and DNA synthesis were enriched among CCR genes, consistent with their known cell cycle regulation. Genes encoding two-component signal transduction proteins (response regulators and histidine kinases) were also significantly enriched among CCR protein-encoding genes, while genes encoding sequence-specific DNA-binding proteins (e.g., transcriptional regulators) were overall under-represented. In addition, this analysis showed that some metabolic pathways (e.g., nitrogen and sulfur compound metabolic processes) were over-represented in terms of cell cycle regulation while others (e.g., respiration) were under-represented.

Five-hundred forty-one CCR genes did not have a GO term and these genes were in general less conserved across the phylogenetic tree than the 1,024 CCR genes with GO terms based on PI distributions (Figure 5B). However, a subset of them (22) were subject to strong selective pressure with PI > 50 (Figure 5C, KS test, p<1e-3). These conserved genes are interesting candidates for future cell cycle studies.

### Cell cycle co-expression network and modules

Since genes with correlated expression profiles can suggest correlations in biological function or regulatory mechanism, we used Weighted Gene Correlation Network Analysis (WGCNA) [31-33] to determine co-expression profiles among the 1,586 identified CCR genes. From this analysis, we were able to cluster the CCR genes into 76 modules. Each module contains genes with similar cell cycle expression profiles, and the overall expression profile of each module can be represented by the first eigenvector of the module. On average, the first eigenvector was able to explain over 85% of the total variance, with even the worst case (the maroon module) still explaining 78% of the total variance (Additional file 13: Figure S4, see Eigen_varExplained.txt file).

**Figure 6:**
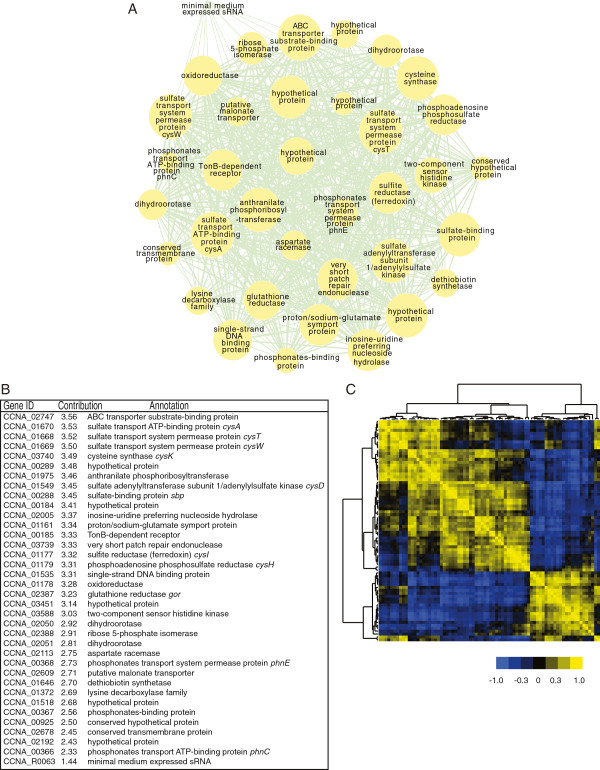
**WGCNA co-expression modules. (A)** WGCNA modules were constructed based on the Topological Overlap Matrix. This is an example displaying the topology of the magenta module. Nodes are genes, and the size of node is proportional to its contribution in forming this module. Width of the edges is proportional to strength of correlation between two connected genes. **(B)** Table showing the contribution of all member genes to the formation of the magenta module. **(C)** Clustering of the 76 modules shows as a 76 by 76 heat map matrix resulted from bi-clustering based on the module eigenvectors. The eigenvector of the expression matrix for each module was used to represent its expression profile. Each row or column is one module, and the color in each small cell describes the relationship between two modules. Yellow shades indicate positive correlations (i.e., similar expression profiles) between two modules, whereas blue shades mean anti-correlations. Black indicates no correlation.

Figure 6A shows one of the modules as an example, with each node representing a specific gene of the module and with the size of the node being proportional to its contribution to the module (see methods; Figure 6B lists the contribution of each gene in forming the module, and Additional file 14: Table S6a provides gene contributions in all modules). The edges between genes (nodes) indicate connectivity; wider lines indicate stronger connectivity and are indicative of greater similarity in cell cycle expression profile between the two connected genes. All 76 modules are displayed in Additional file 13: Figure S4. We used the eigenvectors to cluster the 76 modules according to their cell cycle expression profiles to examine the relationship between the 76 modules (Additional file 15: Figure S5). This clustering analysis, by and large, resulted in three large groups with peak expression primarily at the SW, ST or PD cell cycle stage (Additional file 16: Table S6b).

**Figure 7:**
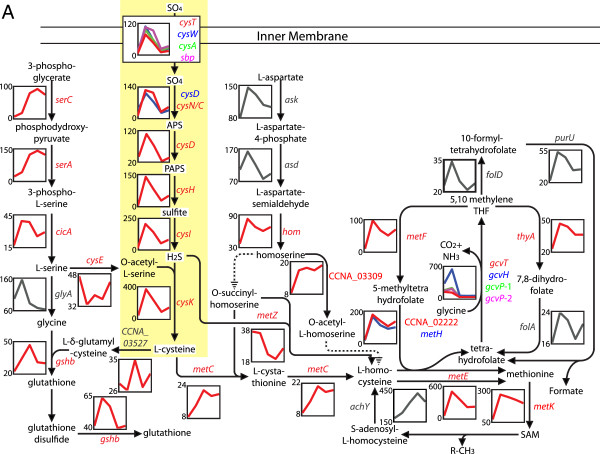
**Correlated cell cycle expression of the sulfur metabolic network. (A)** Every reaction performed by a predicted enzyme has the gene name and mRNA expression profile flanking the reaction arrow. Dotted arrows indicate that no *C. crescentus* gene has been predicted to perform this specified reaction. The color of protein names corresponds to the color of the expression profile. Protein and expression profiles in grey indicate that they were not identified as cell cycle-regulated because of the variance between biological replicates. Genes in yellow box are major contributors of the magenta module (see Figure 6B) and are involved in sulfur import and assimilation to cysteine. The expression data used to build the cell cycle profiles are provided in Additional file 3: Table S1b.

Individual modules can be searched for functional relationships among genes, and this search can be broadened to modules with similar expression profiles (Figure 6C) to identify functionally related genes. For example, genes involved in the assimilation of sulfur into cysteine are among the strongest contributors of the magenta module (Figure 6B), with changes in expression up to 130-fold and with peaks of expression in the ST and EPD cell cycle stages (Figure 7). Aside from its use in protein synthesis, cysteine is the primary donor of sulfur in the metabolism of a variety of sulfur-containing compounds, including methionine, S-adenosylmethionine (SAM), coenzyme A, glutathione, thiamine, lipoic acid, cobalamin, biotin, molybdenum cofactor, and iron-sulfur clusters [34,35].

When we examined co-expression modules that cluster with the magenta module because of their similarity in cell cycle expression pattern, we identified genes from pathways tangential to cysteine synthesis. Using this strategy, we were able to identify the metabolic network involved in cysteine, methionine, serine, glycine, glutathione, and SAM synthesis (Figure 7). The entire network is created from 38 genes, expressed from at least 25 transcriptional units (the genes had to be separated by at least 2 kb or had to be transcribed in opposite directions to be considered as distinct transcription units). Of these 38 genes, 31 display differential cell cycle expression, and most are up-regulated at the ST and/or EPD cell time point (Figure 7). Thus, gene and module clustering can be used to infer functional coupling between genes and pathways.

### Cell cycle transcriptome analysis from an evolutionary perspective

In terms of gene persistence, CCR genes and non-CCR genes showed no differences (Additional file 17: Figure S7). However, the contribution of each CCR gene in forming a co-expression module was not equal, with the persistent genes (PI≥150) being more prone to be major contributors compared to the rest of CCR genes (KS test, p<1e-5). In other words, CCR genes that are widely conserved across bacterial phyla tend to determine the expression profile of their module, suggesting that evolution plays a role in shaping gene co-expression networks.

**Figure 8:**
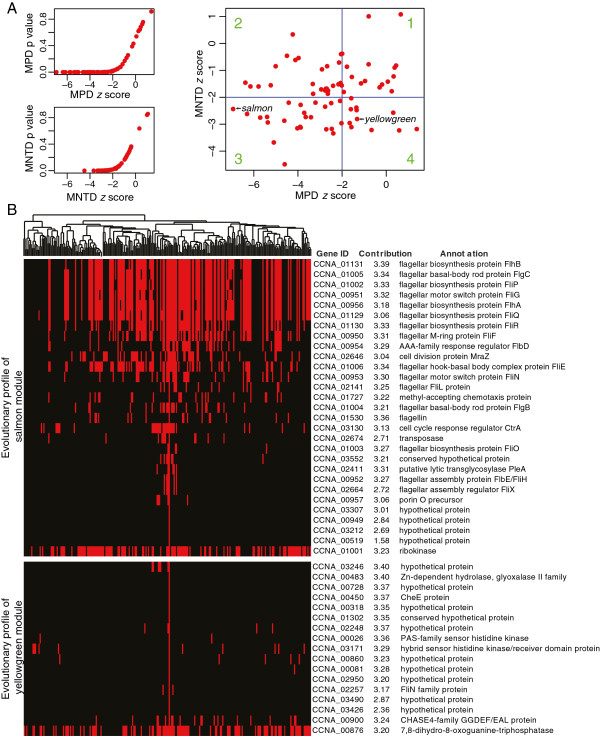
**Phylogenetic analysis of co-expression modules. (A)** Mean pairwise distance (MPD) and mean nearest taxon distance (MNTD) were used to measure the tree-wide and tip-level clustering of each module, respectively. The two small panels on the left show that -2 is a significant threshold (with p=0.01) for both MPD and MNTD *z* scores. In the right panel, modules (red dots) are displayed in 4 quadrants. Modules in the 2nd and 3rd quadrants have significant low MPD values (< -2), indicating that their genes display tree-wide clustering. Modules in the 4th quadrant have significantly low MNTD (<-2) but not significant MPD values, indicating that their genes exhibit tip-level clustering. **(B)** Phylogenetic profiles showing the distribution of the member genes of the salmon and yellowgreen modules shown here as examples. The dendrogram on the top of the module profile is the phylogenetic tree based on 16 s rRNA of 236 species selected in an unbiased fashion (see methods). The solid vertical red line corresponds to *C. crescentus*.

Previous studies have shown a correlation between co-expression and co-evolution by examining conserved synteny and/or co-expression of conserved gene pairs across different organisms, [36-40]. We were therefore interested in understanding the link between co-expression and evolutionary relatedness from the perspective of a model organism’s biological network by leveraging our co-expression modules. For each module, we computed the phylogeny clustering of its member genes (see methods) using the *K-*statistics [41] in the picante package [42]. Sixty-nine (91%) modules had strong phylogenetic signals (p < 0.05, Additional file 18: Table S7); that is, the genes in these 69 modules are phylogenetically clustered (see methods). To more precisely study this phylogenetic clustering, we calculated the mean pairwise distance (MPD) and mean nearest taxon distance (MNTD) values using picante. The MPD value can provide a measure of the phylogenetic tree-wide patterns of clustering. MNTD is, on the other hand, more sensitive to clustering closer to the tips of the phylogenetic tree [43]. For example, some genes may be randomly distributed across the tree, but phylogenetically clustered near the tips. MNTD would show a significant value for such clustering. Genes that are specific to species or to narrow clades will also show significant MNTD values. The distribution of MPD and MNTD *z* scores are shown in Figure 8A. We found that values ≤ -2 for both MPD and MNTD *z* scores are significant (with p=0.01; Figure 8A). Hence, we divided the MPD and MNTD coordinates into 4 quadrants using the cut-off value -2 (Figure 8A). Forty-nine (64%) modules in quadrants 2 and 3 display tree-wide clustering; the salmon module is such an example (Figure 8B). Eleven modules in quadrant 4 are more likely to be clade- or species-specific modules; the yellowgreen module provides an example (Figure 8B). The evolutionary profile of each module is provided in Additional file 19: Figure S6, whereas the MPD and MNTD *z* scores are listed in Additional file 18: Table S7.

Collectively, these phylogenetic analyses suggest that gene conservation and co-expression are highly correlated: broadly conserved genes presumably organized into functional modules in ancestral species and have co-evolved as groups into many extant organisms, whereas narrowly conserved genes tend to be co-expressed together as clade- or species-specific modules. Understanding the mechanism that drives co-expressed genes to co-evolve, or co-evolved genes to be co-expressed, will be of great interest, as it is beyond the influence of operon organization [26,44].

## Conclusion

In this work, we leveraged the CV analysis of biological replicates to refine our expression measurements and to correct systematic biases associated with GC-rich genomes. Using this strategy, we identified three global patterns of gene expression that appear to be under distinct regulatory constraints. By integrating two popular tools, WGCNA and baySeq, we generated a list of CCR genes and identified previously unknown relationships between these CCR genes. Through phylogenetic analysis of expression network modules, we found a correlation between stronger co-expression and broader conservation of genes. By investigating the evolutionary profiles of the modules and their MPD/MNTD coordinates, we found that most (64%) modules with strong tree-level clustering were dominated by widely conserved genes, and that 11 modules with strong tip-level clustering were dominated by clade-specific genes. In total, this accounted for 79% of the 76 modules, which argues that evolutionary profiles are highly related to gene co-expressions and that evolution has shaped the cell cycle expression network. This further implies that selective pressure is not on single genes but rather on the relationships between genes (i.e., the biological network), emphasizing the value of including phylogenetic analysis to the study of gene co-expression networks.

## Methods

### Bacterial growth and RNA collection

*C. crescentus* NA1000 (also known as CB15N) was grown at 30°C in M2G until the exponentially growing culture reached an OD_660_ of about 0.3. Cell synchronization, which includes a centrifugation in a density gradient of silica (Ludox) at 4°C, was performed as previously described [13], using 1 L of culture. After synchronization, the purified swarmer cell population was resuspended in pre-warmed M2G medium. A total of 5 synchronies were done to obtain 3 time points such that, in total, we obtained three replicates (50 ml aliquots of cells) at 0, 30, 60, 90 and 120 min following synchronization. Total bacterial RNA was isolated using phenol-chloroform extraction, as described previously [45]. The quality of the extracted RNA was assessed by agarose electrophoresis; rRNA bands appeared intact and no RNA smear was apparent. RNA samples were immediately frozen and stored at -80°C. RNA samples were later enriched for mRNA using the Invitrogen Ribominus Transcriptome Isolation Kit (Yeast and Bacteria) to remove ribosomal RNA per the manufacturer’s protocols except for the use of custom-made nucleic acid probes (Invitrogen) designed against *C. crescentus* ribosomal sequences. All RNA samples were tested for integrity on a BioRad Experion capillary electrophoresis system. Possible residual DNA was removed by addition of Ambion Turbo DNase.

### Library preparation, sequencing and mapping

Fifteen sequencing libraries for Applied Biosystems SOLiD system sequencing were created using the Applied Biosystems Whole Transcriptome Library Preparation for SOLiD Sequencing, and individual samples were barcoded using Applied Biosystems Small RNA Expression Kit (SREK) barcodes (per the manufacturer’s protocols). Transcriptome library preparation was performed for labeling in a strand-specific manner. Samples were run on the Applied Biosystems SOLiD 3 Platform using Shotgun Sequencing (50 base pair reads) using standard sequencing protocols. Each experimental time point was run on an individual flow cell containing the 3 biological replicates with different barcodes. Raw color space data from SOLiD sequencing was mapped to the *C. crescentus* NA1000 chromosome (NC_011916) (plus and minus strands, separately) using SOCS software with a mismatch cutoff of 5 nucleotides [46], which discards about half of the reads. We assigned weights of 1, 0.95, 0.9, 0.85, 0.8 and 0.75 to reads with 0 to 5 mismatches, respectively, when summing them together.

### RNA-Seq normalization

From the bird’s eye view of raw RNA-Seq mapping (Figure 2A), we observed some spikes, indicating large concentrations of RNA-Seq reads at those locations. From Figure 2D, we also detected tails of highly expressed genes, which did not follow the major power-law distribution of the genome. Furthermore, a Chi-square test confirmed that the amount of reads mapped to highly expressed genes (>1000x) did not follow the same distribution than those mapped to the bulk of genome (p<1e-8). Hence, samples with ≤1000x and >1000x were normalized by the sum of each replicate separately.

### Quantification of gene expression

We employed a dynamic programming segmentation algorithm from the tillingArray package [47] to divide the CV curve into segments, as shown in Figure 2C. We removed segments with CV >1 before quantifying gene expression. We then calculated the weighted mean coverage in the remaining segments that fell within annotated CDS or RNA coordinates as gene expression value.

### Gene ontology analysis

GO (gene ontology) annotation was downloaded from EBI UniProt-GOA [30], which included 2,564 *C. crescentus* NA1000 genes. We mapped our CCR genes to this dataset and obtained the GO for 1,024 protein-encoding CCR genes, and their biological process (level 3) GO terms distribution (Figure 5A) was summarized and drawn by Blast2GO [48]. GO terms enrichment analysis was also carried out using Blast2GO, and significant GO terms were reported in Additional file 18: Table 5S with their Fisher’s exact test p-value < 0.01. We also provided FDR corrected p-values for reader’s reference.

### Identification of cell cycle-regulated genes and construction of the WGCNA co-expression network construction

The baySeq package [29] was used to identify CCR genes. Based on baySeq minimum requirement, we assumed two conditions for each gene, up or down regulated. We enumerated all possible combinations of the up and down regulation across 5 time points (each with three identical replicates), and included no expression as well as constant expression without changes, as the models to be evaluated by baySeq for each gene. baySeq considered the variance in the three biological replicates when estimating the likelihood, and assigned genes into the model that best described their cell cycle expression profile. Genes that were assigned to models with differential expressions were considered as CCR genes. Similar to our normalization procedure, we ran the baySeq workflow for the highly expressed genes and for the bulk genome separately. To construct the gene co-expression modules, we first followed WGCNA’s data filter suggestion and removed one replicate from each of the SW, ST and EPD time points. We then constructed signed network with β=36 and minimum module size of 5 using the WGCNA default Topological Overlap Matrix (TOM) [33]. The eigenvector of each module’s expression matrix was used to represent the expression profile of the module, and scaled gene expression profiles were projected onto this eigenvector to calculate contributions from the member genes. Cytoscape was used to draw the network topology of the module [49].

### Phylogenetic signal and evolutionary profiles of co-expression modules

We used 1 or 0 to represent whether or not a CCR gene is conserved in a species. For each module, we summed the conservation values of all member genes in each of the 236 species to obtain a distribution profile across the selected species. This distribution profile was then treated as the trait data, and the *K*-statistic and the associated p-value were calculated according to Blomberg et al’s algorithm [41]. MPD and MNTD values were calculated based on the same species-distribution profiles for each module, and null model used in the calculation was generated by randomizing the species-distribution of each module 9,999 times, while maintaining the phylogenetic relationships [42].

### Orthology and gene persistence

The large 16 s rRNA phylogenetic tree from Greengenes [50], which covers over 800,000 bacterial species, was first cut into about 300 evenly speciated clades. We selected all fully sequenced bacterial genomes with > 1.5 M from EMBL, and mapped them into 236 Greengenes clades. From each clade, we randomly selected one species as representative (Additional file 20: Table S8). The persistence index (PI) of a *C. crescentus* gene was defined by the number of orthologs found in the 236 selected species. Orthology was acquired by bi-directional best hits with protein sequences similarity over 40% and protein length difference under 20% [26,51]. In addition to obtaining the PI value for each *C. crescentus* gene, we used a set of less stringent criteria to identify all proteins (referred to as homologs) with over 40% of similarity and less than 50% of length difference. The results are documented in Additional file 15: S1 and Additional file 21: Table S10.

## Availability of supporting data

The data set supporting the results of this article is available in the NCBI Gene Expression Omnibus (GEO) repository, with access number GSE46915 (http://www.ncbi.nlm.nih.gov/geo/query/acc.cgi?acc=GSE46915).

## Abbreviations

CCR: Cell cycle-regulated; PI: Persistence index; CV: Coefficient of variation; SW: Swarmer; ST: Stalked; EPD: Early predivisional; PD: Predivisional; LPD: Late predivisional; SAM: S-adenosylmethionine; MPD: Mean pairwise distance; MNTD: Mean nearest taxon distance.

## Competing interests

The authors declare that they have no competing interests.

## Authors’ contribution

GF performed the data analysis and introduced the quantitative phylogenetic analysis of gene co-expression modules. CJ-W, PML, NHB and KP designed the experiments. PML prepared the RNA samples. KP, under NHB's supervision, carried out the RNA-Seq sequencing and mapping. JH analyzed the sulfur pathway. MG participated in the bioinformatic analysis. CJ-W initiated and supervised the study. GF, JH and CJ-W wrote the manuscript. All authors read and approved the final manuscript.

## Supplementary Material

Additional file 1: Figure S1 Frequency distribution of gene expression values. Click here for file

Additional file 2: Table S1a Is a zipped csv file of the raw single-nucleotide resolution RNA-Seq mappings of three replicates from five cell cycle time points. Click here for file

Additional file 3: Table S1b Is a table listing gene expression values after CV correction, for each replicate. Click here for file

Additional file 4: Table S1c Is a table of gene expression values for each cell cycle time point. Click here for file

Additional file 5: Table S2 Is the list of lowly expressed genes that are either essential or persistent genes. Click here for file

Additional file 6: Table S3 Is a table of detailed annotations of the 1,586 identified CCR genes. Click here for file

Additional file 7 **Additional discussions are in the file of Supplemental Information (SI).** Additional files also include supplemental figures S1-S7 with figure legends in file SI. Click here for file

Additional file 8: Table S9 Lists the potential method-introduced CCR genes. Click here for file

Additional file 9: Figure S2 Expression profiles of all identified CCR genes. Click here for file

Additional file 10: Table S4 Is a list of well-studied CCR genes collected from the literature used here as ‘gold standard’. Click here for file

Additional file 11: Figure S3 Directed acyclic graph (DAG) of over- and under-represented gene ontology (GO) terms in CCR genes. Click here for file

Additional file 12: Table S5 Is the result of GO term enrichment analysis of CCR genes. Click here for file

Additional file 13: Figure S4 Co-expression network topologies of all 76 modules. Click here for file

Additional file 14: Table S6a Details how each co-expression module is conserved. Click here for file

Additional file 15: Figure S5 Module expression profile represented by its 1st eigenvector. Click here for file

Additional file 16: Table S6b Is the hierarchical clustering of co-expression modules based on their expression profiles. Click here for file

Additional file 17: Figure S7 Persistent index distributions. Click here for file

Additional file 18: Table S7 Shows phylogeny values (K, MPD and MNTD) for each module. Click here for file

Additional file 19: Figure S6 Phylogenetic profiles and positions in MPD and MNTD coordinates for all modules. Click here for file

Additional file 20: Table S8 Lists the selected bacterial species used to evaluate the conservation of co-expression modules across bacterial phyla. Click here for file

Additional file 21: Table S10 Shows the less stringent PI for each gene. Click here for file
