# Supplementary figures and images for: Transcriptomic and phylogenetic analysis of a bacterial cell cycle reveals strong associations between gene co-expression and evolution (part 1 of 4)
Source: BMC Genomics. 2013 Jul 5;14:450. doi: 10.1186/1471-2164-14-450 (PMC3829707; doi:10.1186/1471-2164-14-450)

# Distribution of gene expression levels

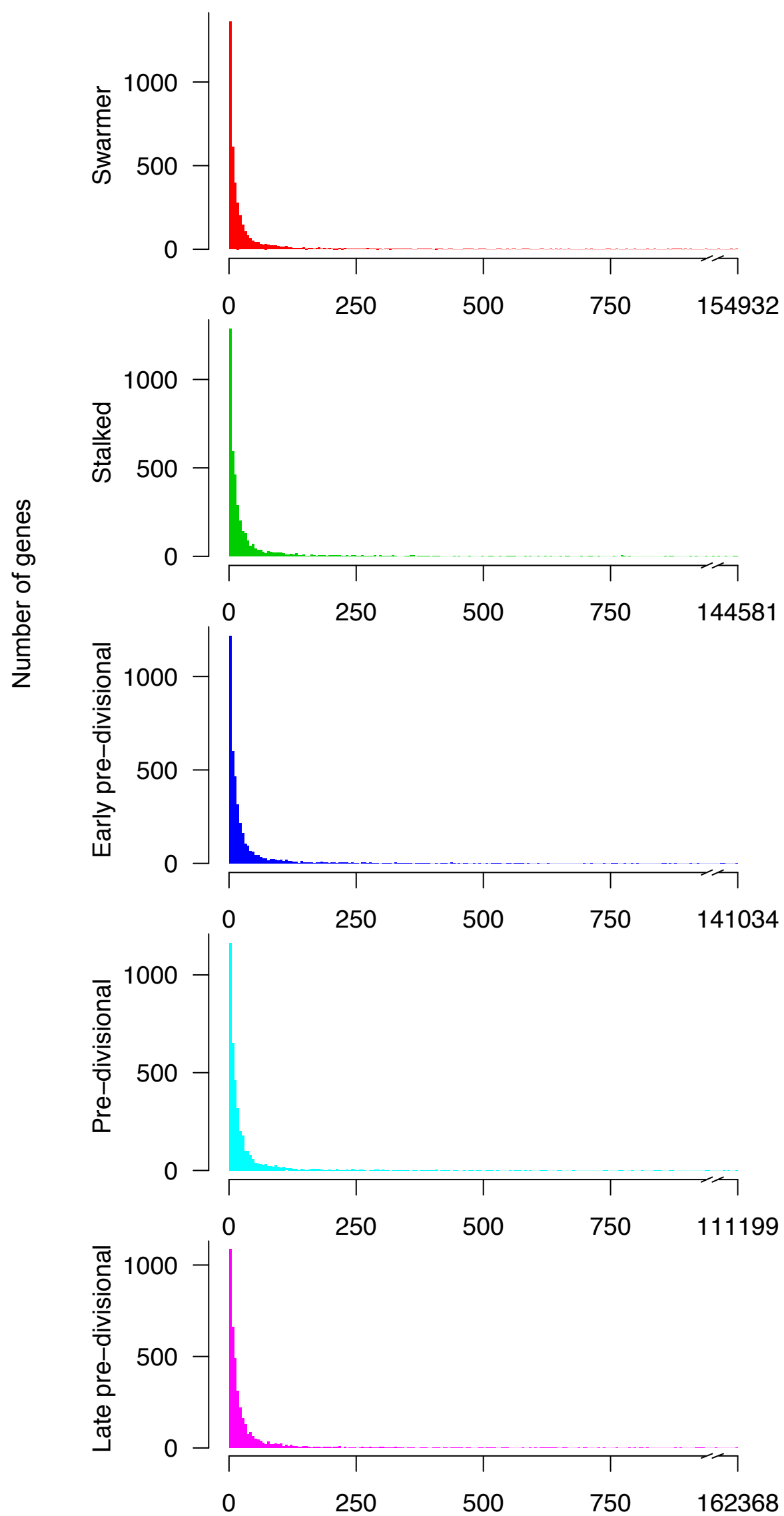

# Power-law fitting to gene distribution

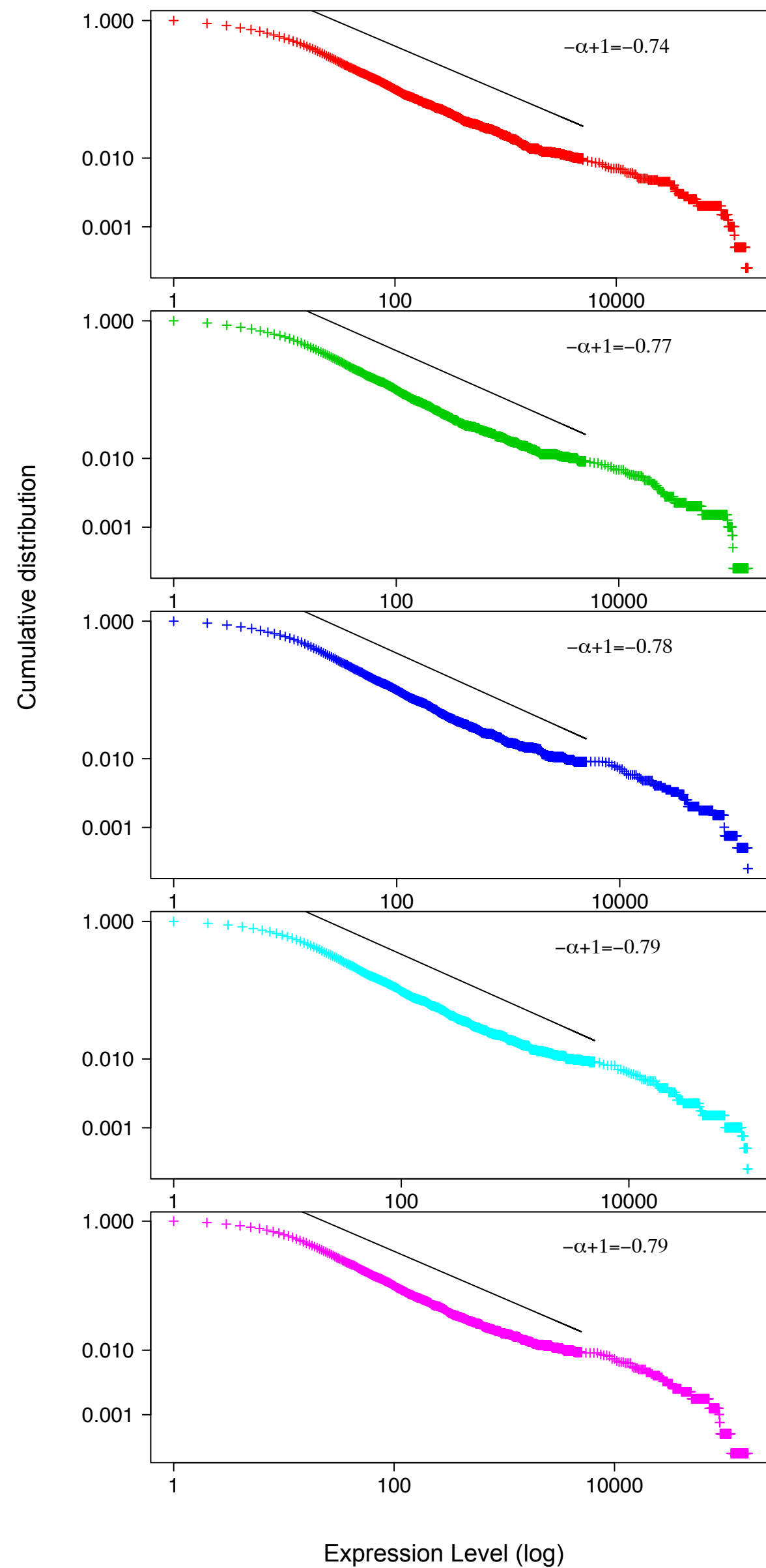

Supplement: Additional file 1: Figure S1 — Frequency distribution of gene expression values. [file 1471-2164-14-450-S1.pdf]

dnaQ;CCNA\_00005

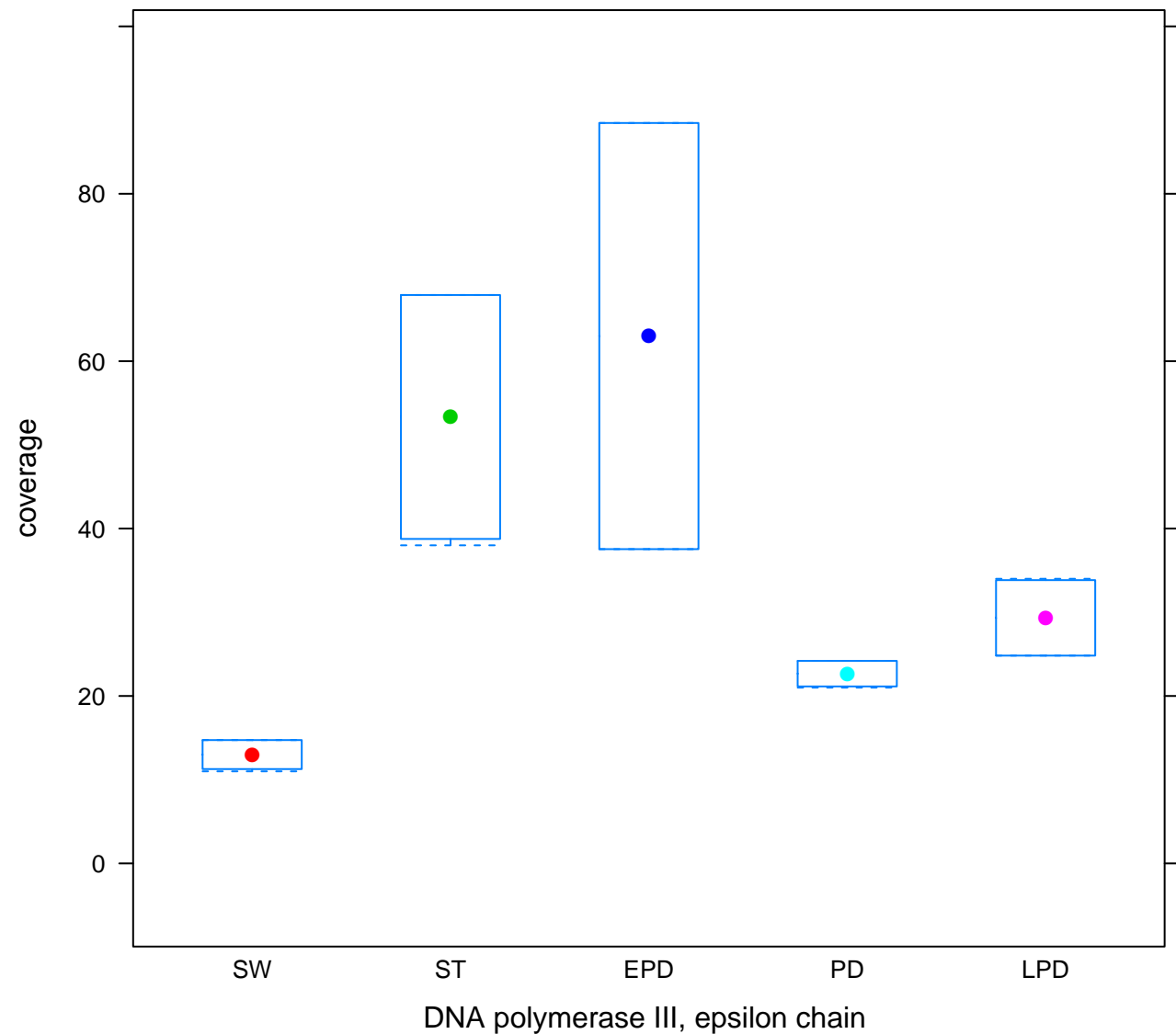

**Fold of change: 5.04**  
**baySeq likelihood: 0.626**

Supplement: Additional file 9: Figure S2 — Expression profiles of all identified CCR genes. [file 1471-2164-14-450-S9.zip › FigureS2/CCNA_00005.pdf]

# CCNA\_00007

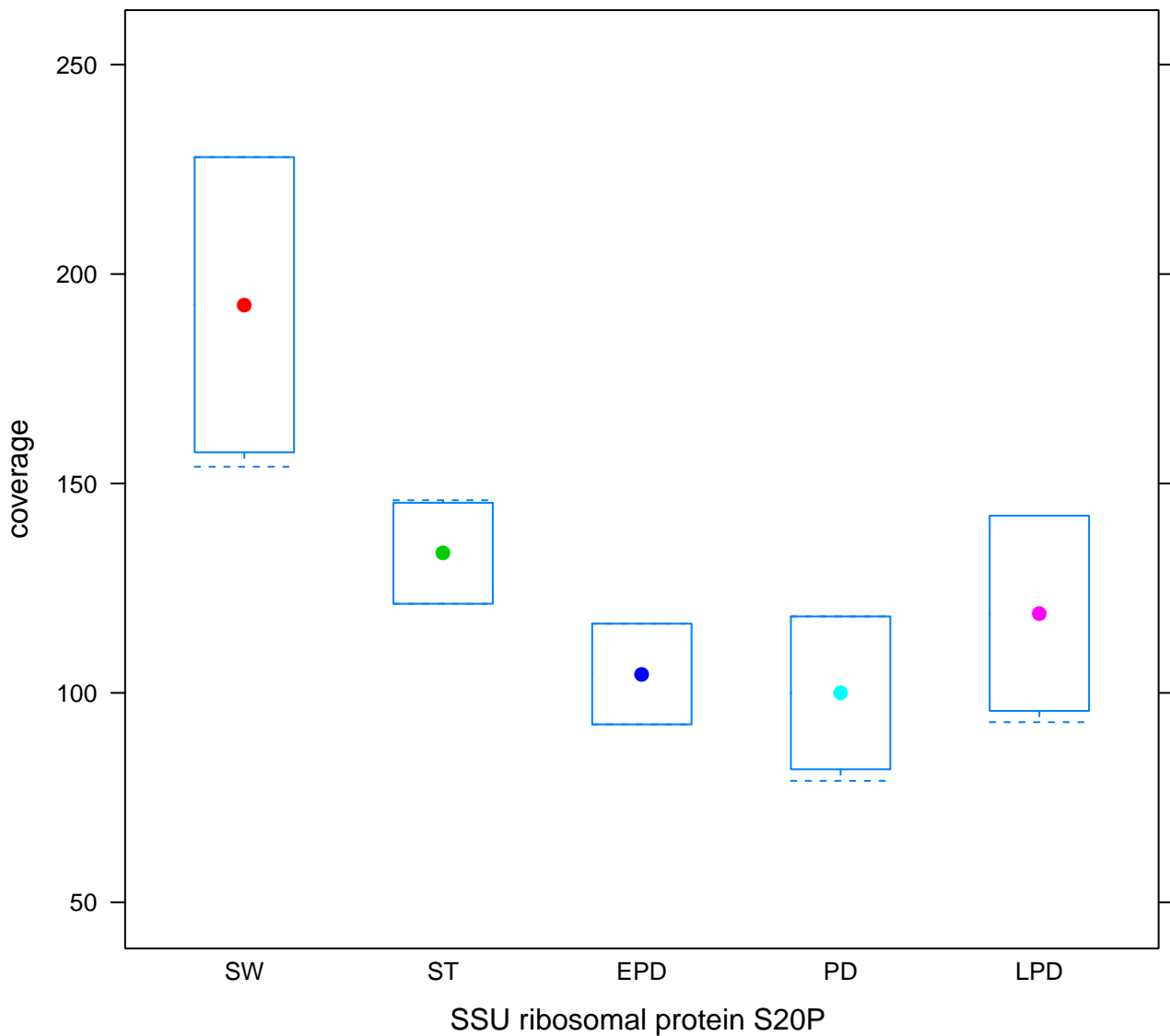

**Fold of change: 1.88**  
**baySeq likelihood: 0.876**

Supplement: Additional file 9: Figure S2 — Expression profiles of all identified CCR genes. [file 1471-2164-14-450-S9.zip › FigureS2/CCNA_00007.pdf]

# dnaA;CCNA\_00008

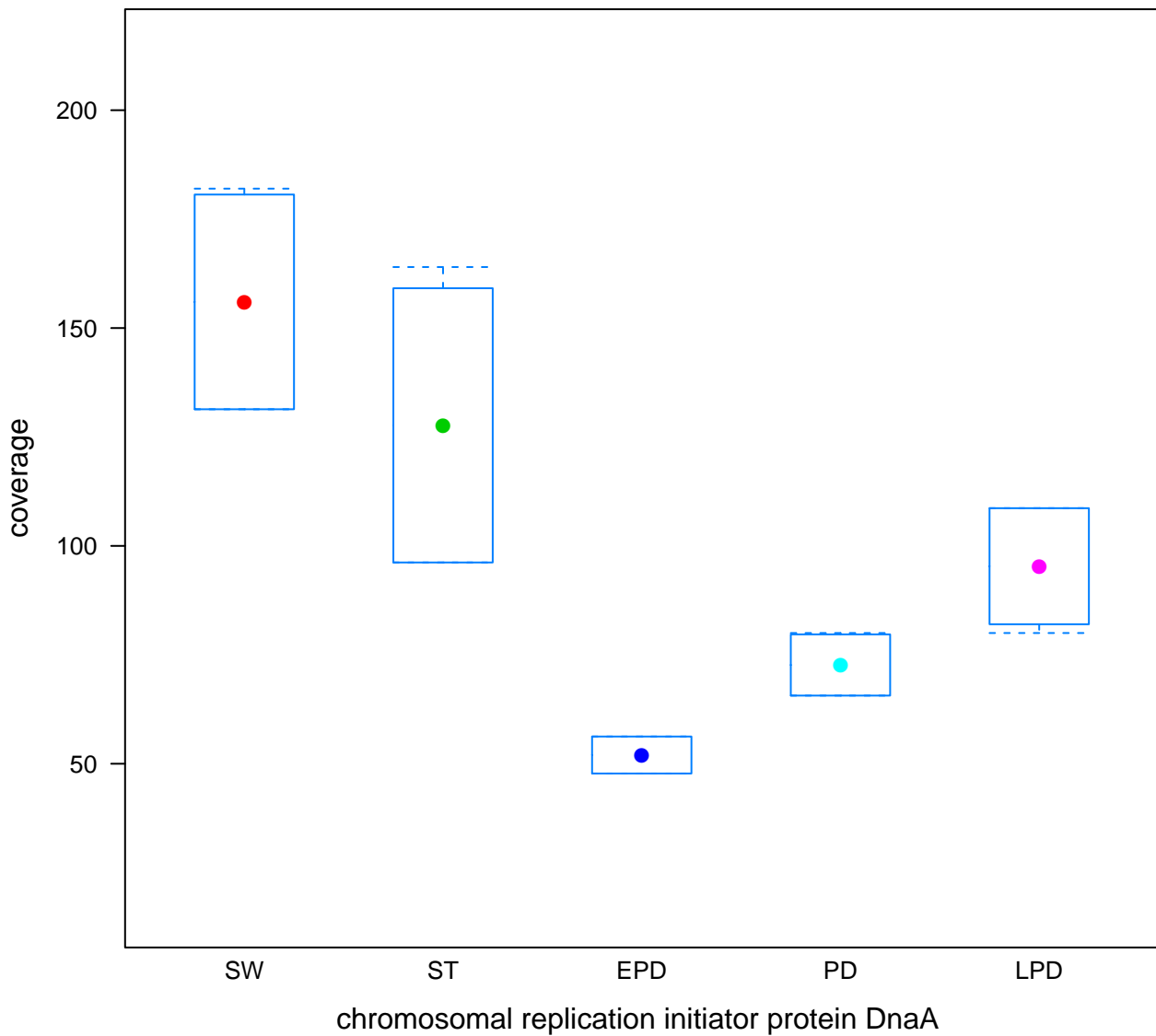

**Fold of change: 3.22**  
**baySeq likelihood: 0.664**

Supplement: Additional file 9: Figure S2 — Expression profiles of all identified CCR genes. [file 1471-2164-14-450-S9.zip › FigureS2/CCNA_00008.pdf]

# dnaJ;CCNA\_00011

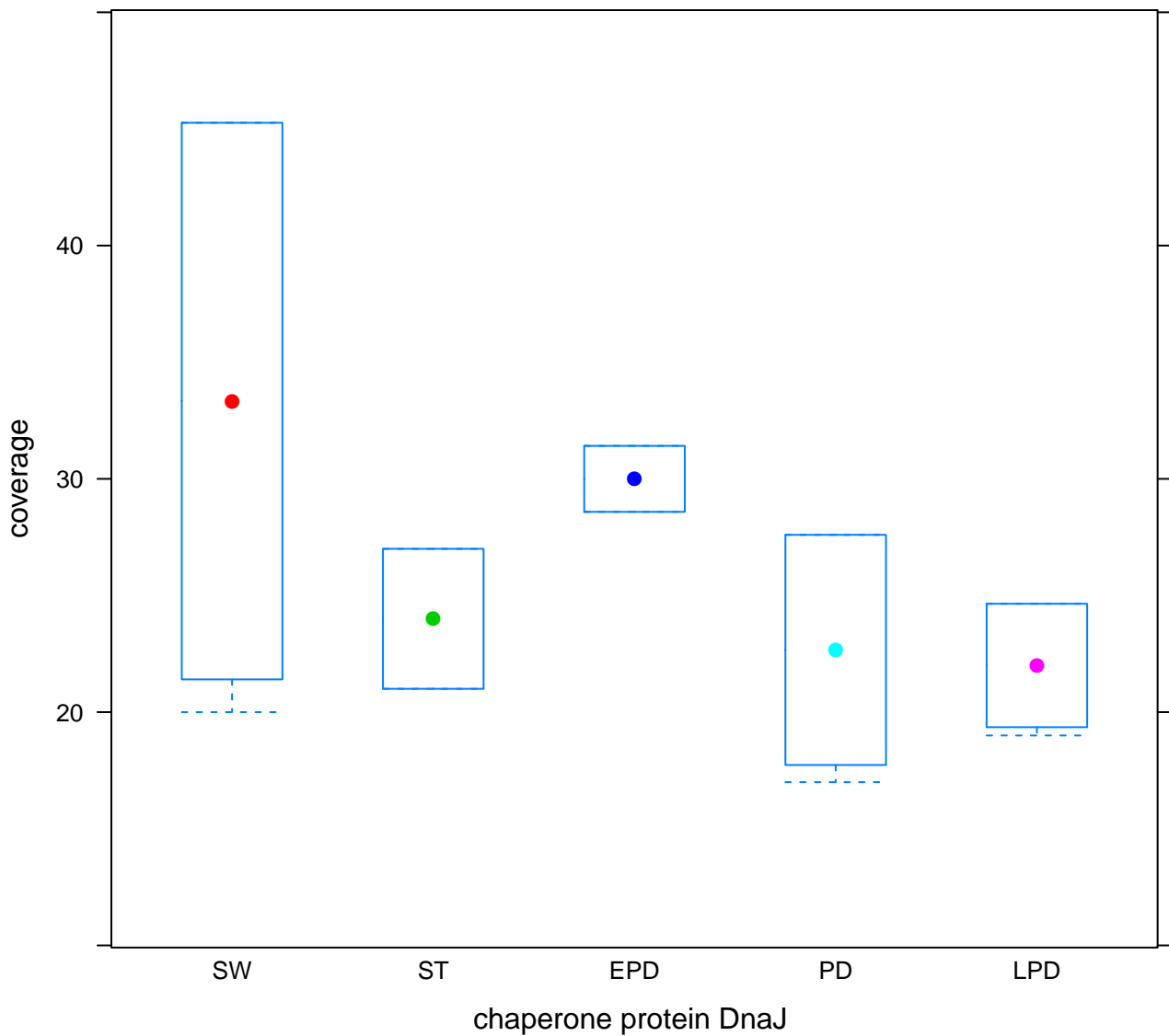

**Fold of change: 1.43**  
**baySeq likelihood: 0.529**

Supplement: Additional file 9: Figure S2 — Expression profiles of all identified CCR genes. [file 1471-2164-14-450-S9.zip › FigureS2/CCNA_00011.pdf]

# mutS;CCNA\_00012

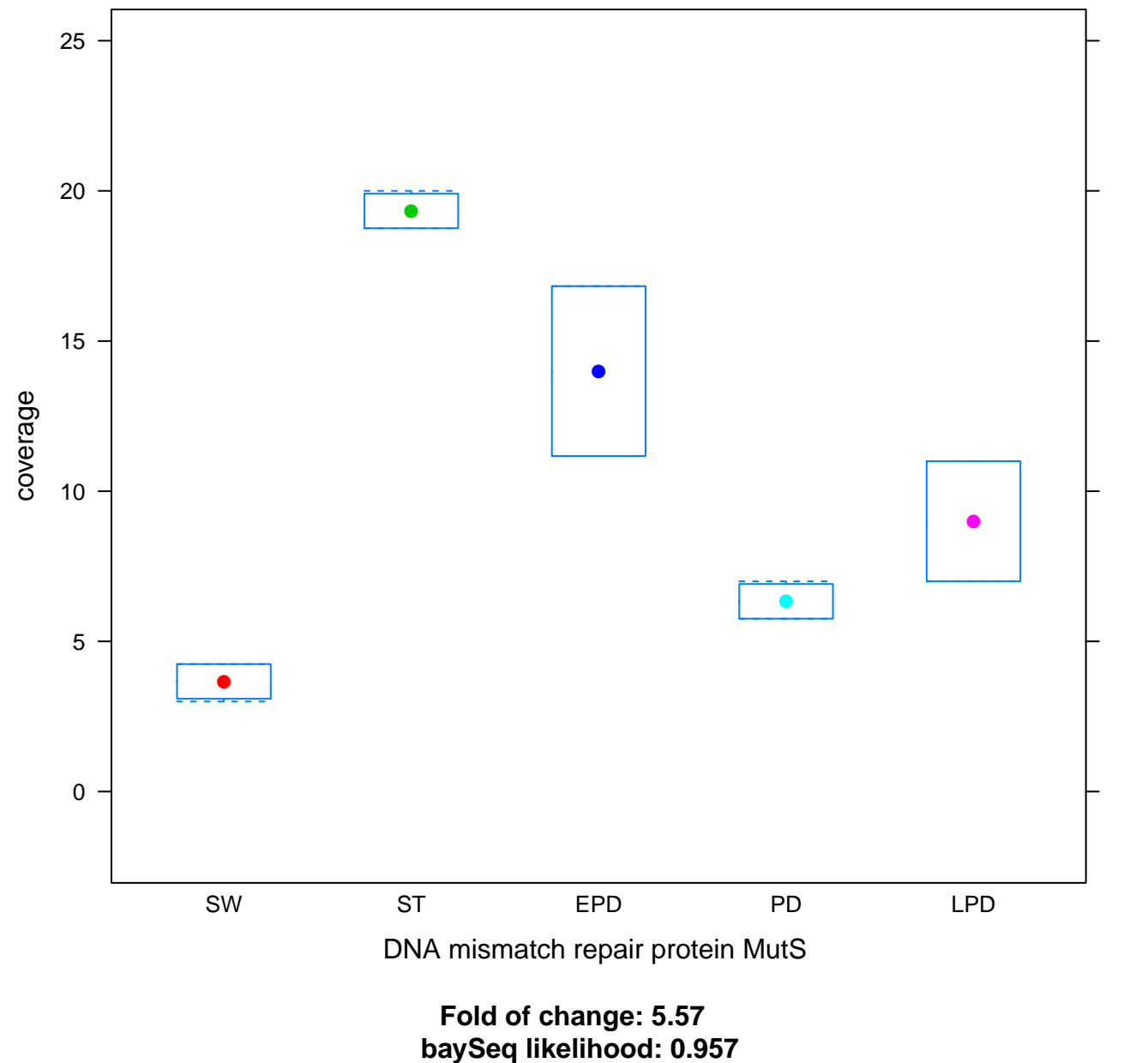

Supplement: Additional file 9: Figure S2 — Expression profiles of all identified CCR genes. [file 1471-2164-14-450-S9.zip › FigureS2/CCNA_00012.pdf]

# CCNA\_00013

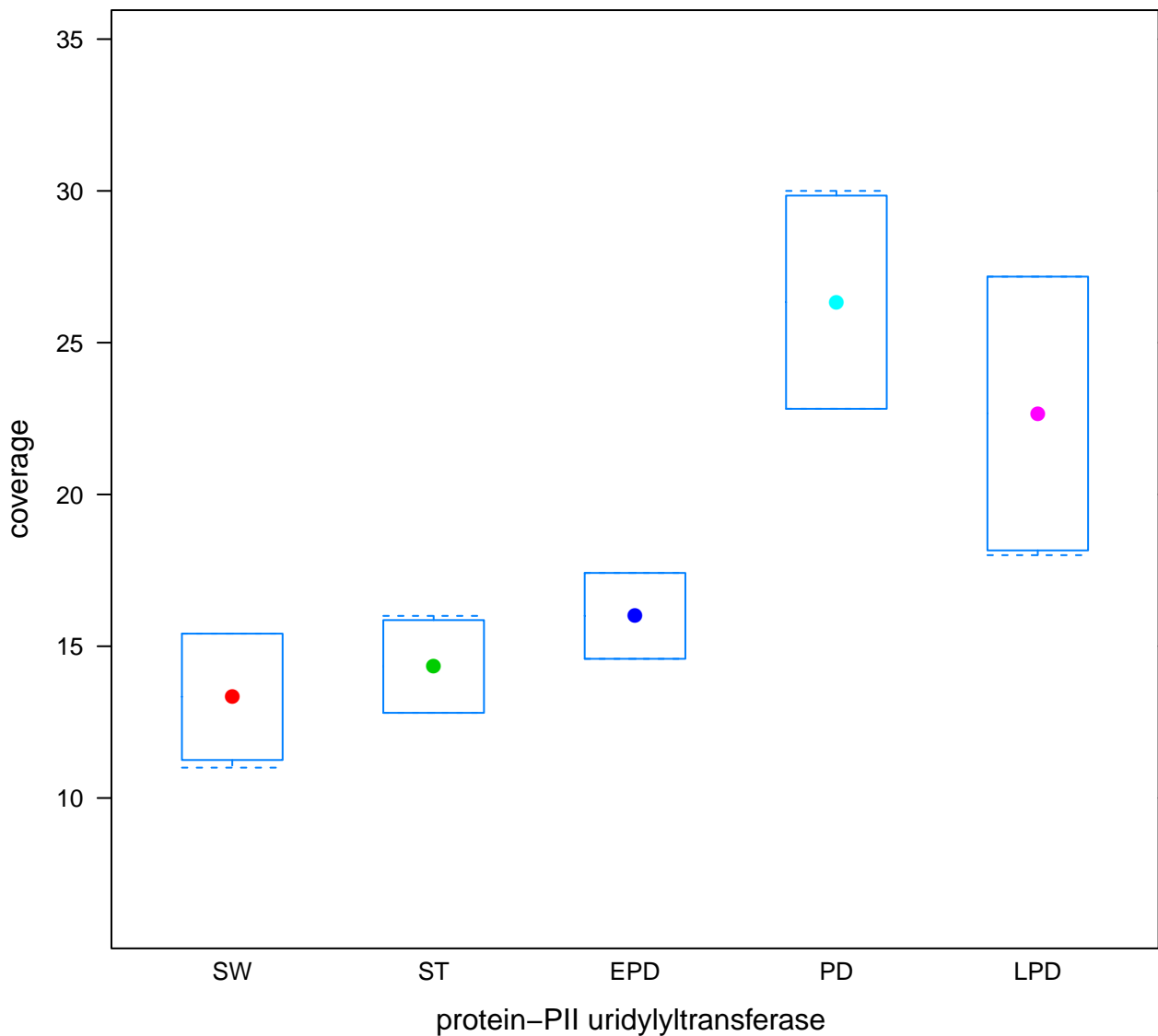

**Fold of change: 2.03**  
**baySeq likelihood: 0.723**

Supplement: Additional file 9: Figure S2 — Expression profiles of all identified CCR genes. [file 1471-2164-14-450-S9.zip › FigureS2/CCNA_00013.pdf]

# CCNA\_00021

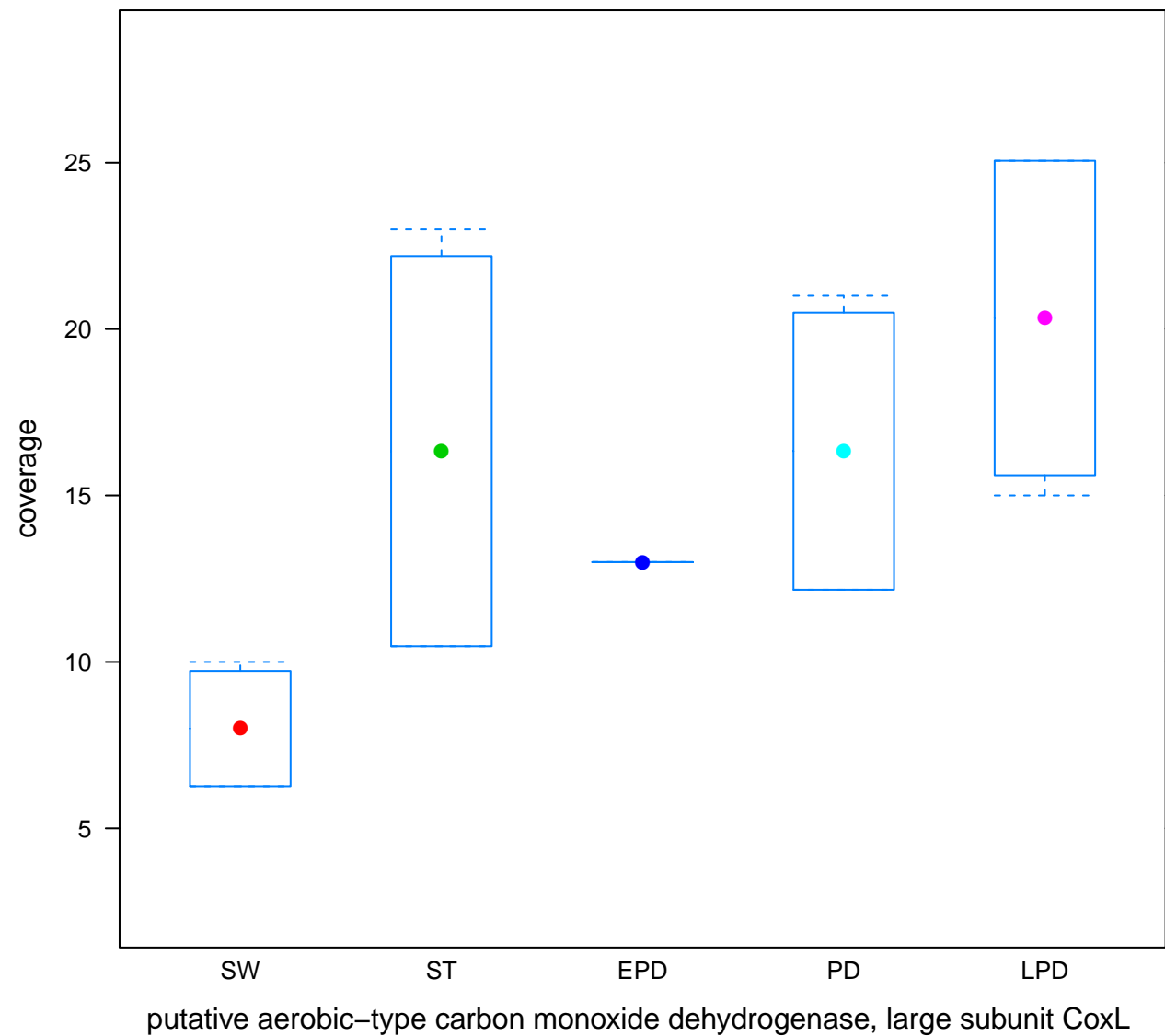

**Fold of change: 2.9**  
**baySeq likelihood: 0.84**

Supplement: Additional file 9: Figure S2 — Expression profiles of all identified CCR genes. [file 1471-2164-14-450-S9.zip › FigureS2/CCNA_00021.pdf]

# CCNA\_00025

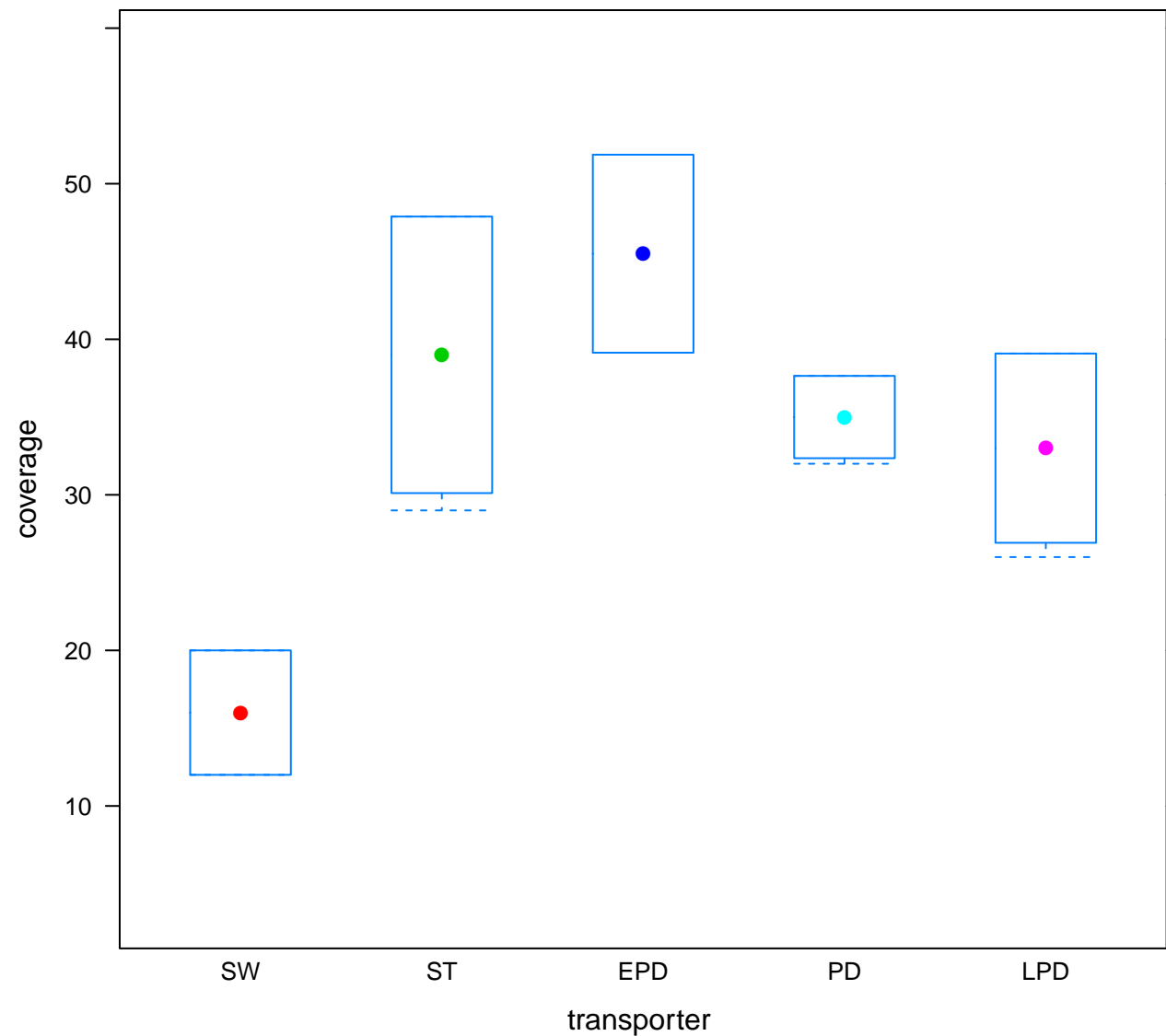

**Fold of change: 3.25**  
**baySeq likelihood: 0.997**

Supplement: Additional file 9: Figure S2 — Expression profiles of all identified CCR genes. [file 1471-2164-14-450-S9.zip › FigureS2/CCNA_00025.pdf]

# CCNA\_00026

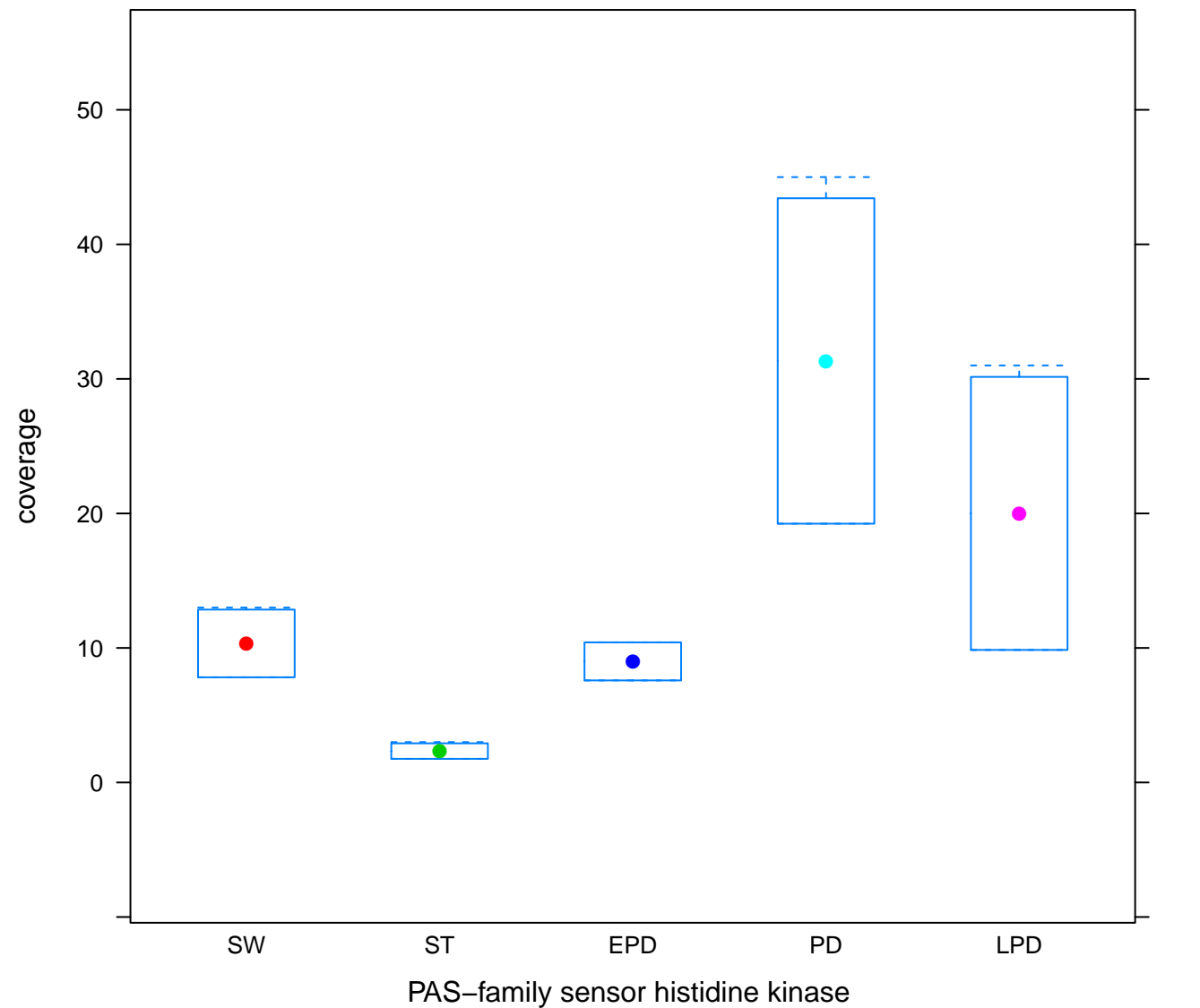

**Fold of change: 12.53**  
**baySeq likelihood: 0.725**

Supplement: Additional file 9: Figure S2 — Expression profiles of all identified CCR genes. [file 1471-2164-14-450-S9.zip › FigureS2/CCNA_00026.pdf]

# CCNA\_00027

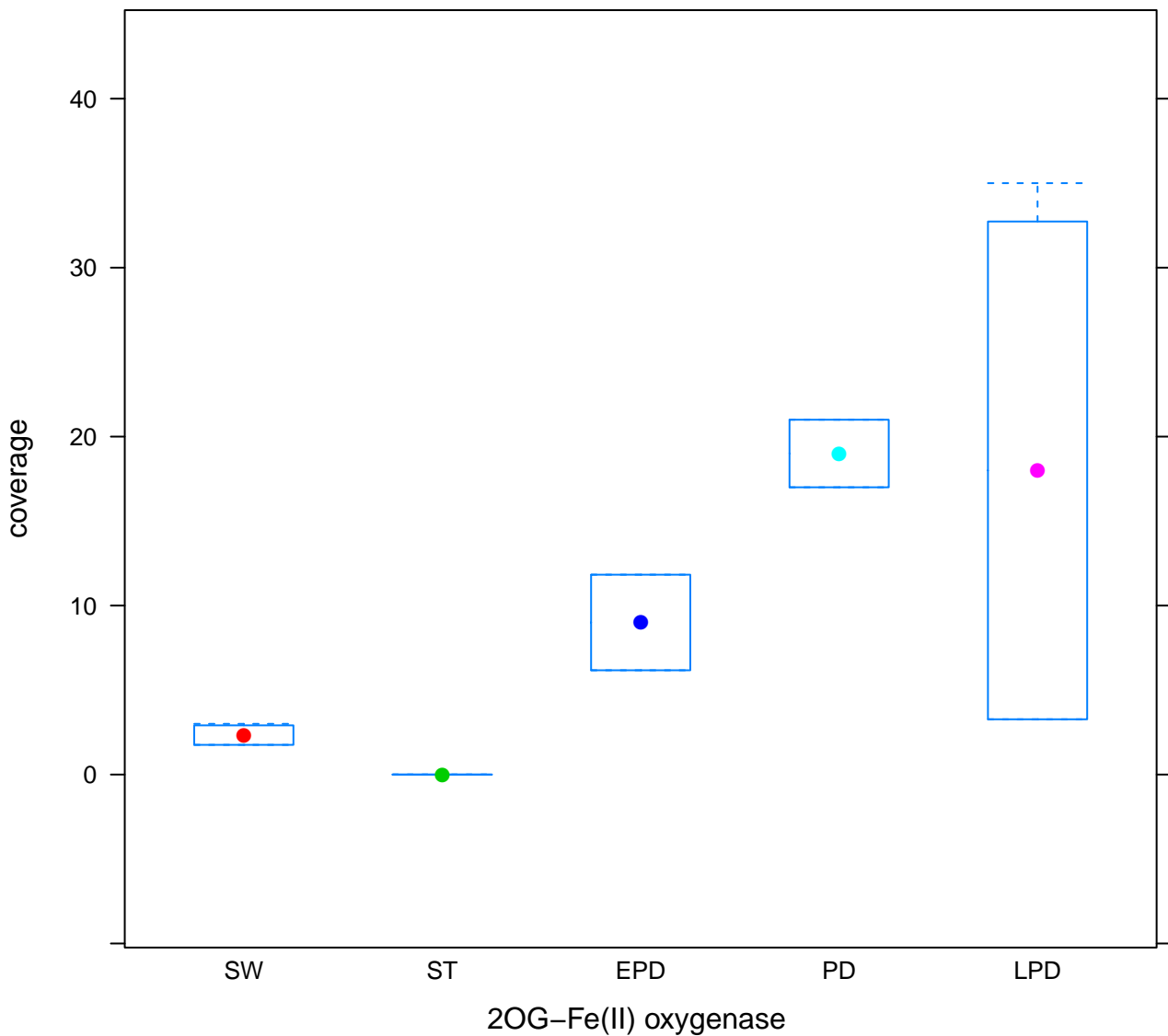

Fold of change: Inf  
baySeq likelihood: 0.996

Supplement: Additional file 9: Figure S2 — Expression profiles of all identified CCR genes. [file 1471-2164-14-450-S9.zip › FigureS2/CCNA_00027.pdf]

# CCNA\_00028

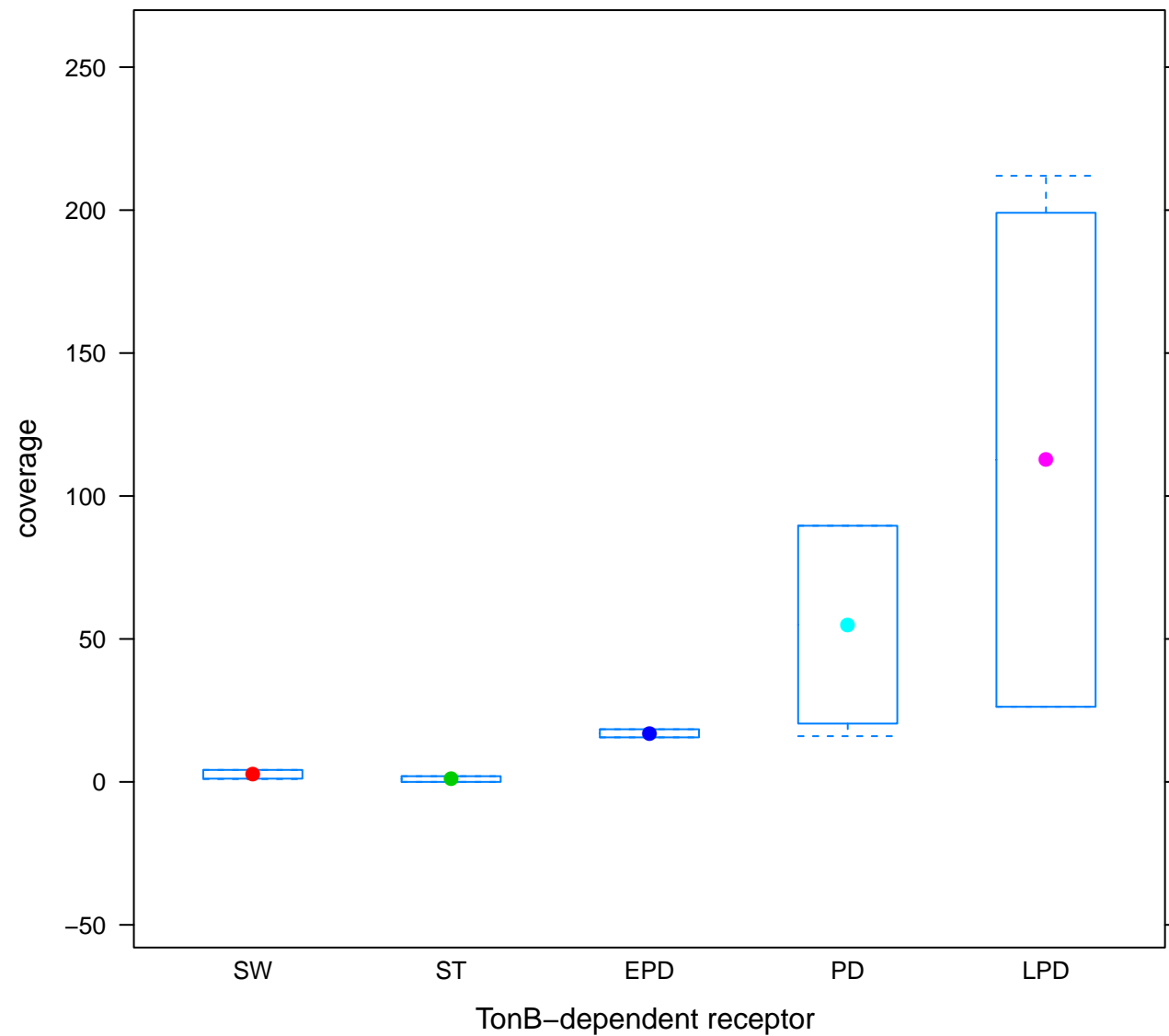

**Fold of change: 112.67**

**baySeq likelihood: 1**

Supplement: Additional file 9: Figure S2 — Expression profiles of all identified CCR genes. [file 1471-2164-14-450-S9.zip › FigureS2/CCNA_00028.pdf]

# CCNA\_00036

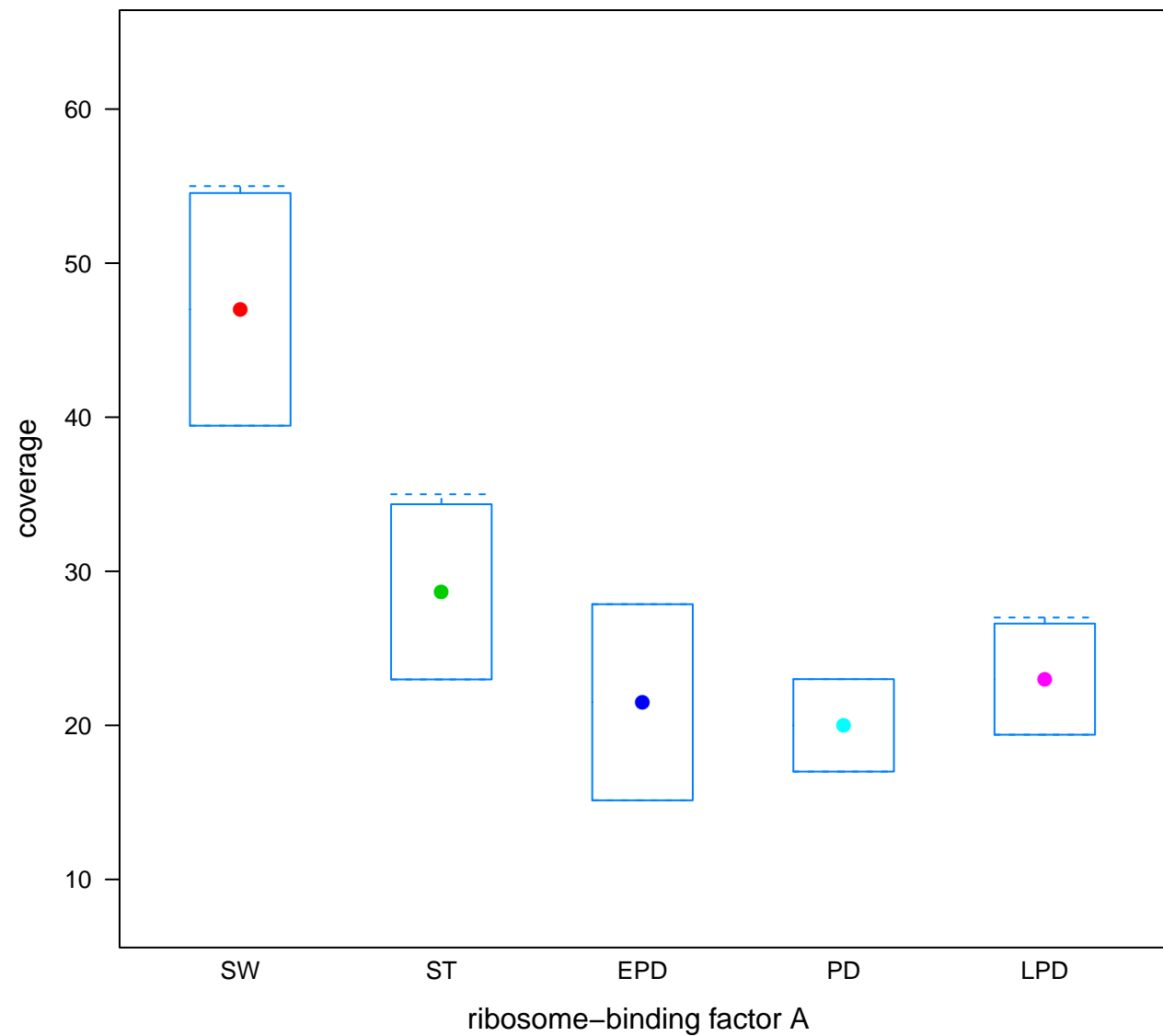

**Fold of change: 2.38**  
**baySeq likelihood: 0.964**

Supplement: Additional file 9: Figure S2 — Expression profiles of all identified CCR genes. [file 1471-2164-14-450-S9.zip › FigureS2/CCNA_00036.pdf]

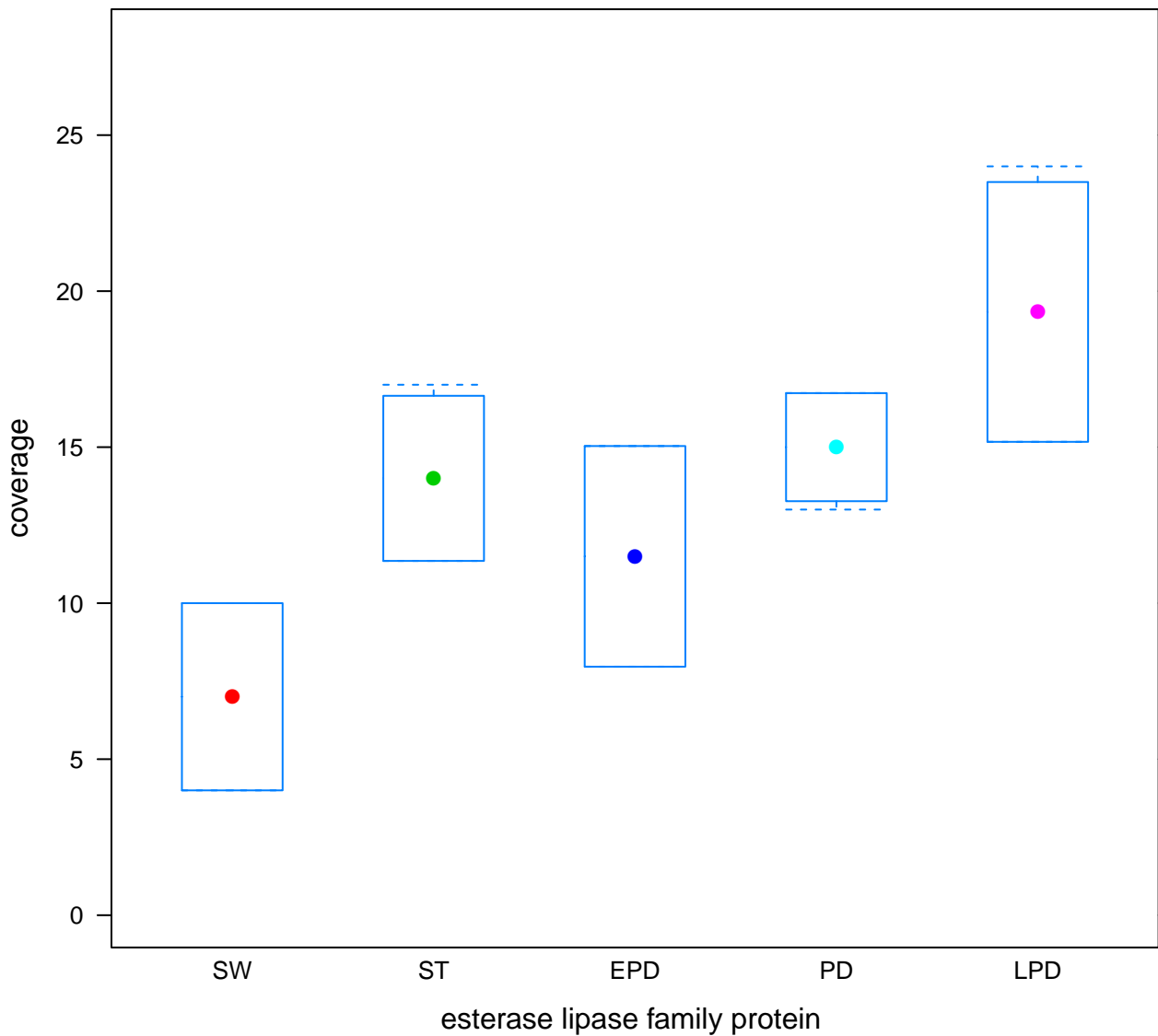

**Fold of change: 3.52**  
**baySeq likelihood: 0.819**

Supplement: Additional file 9: Figure S2 — Expression profiles of all identified CCR genes. [file 1471-2164-14-450-S9.zip › FigureS2/CCNA_00037.pdf]

# CCNA\_00040

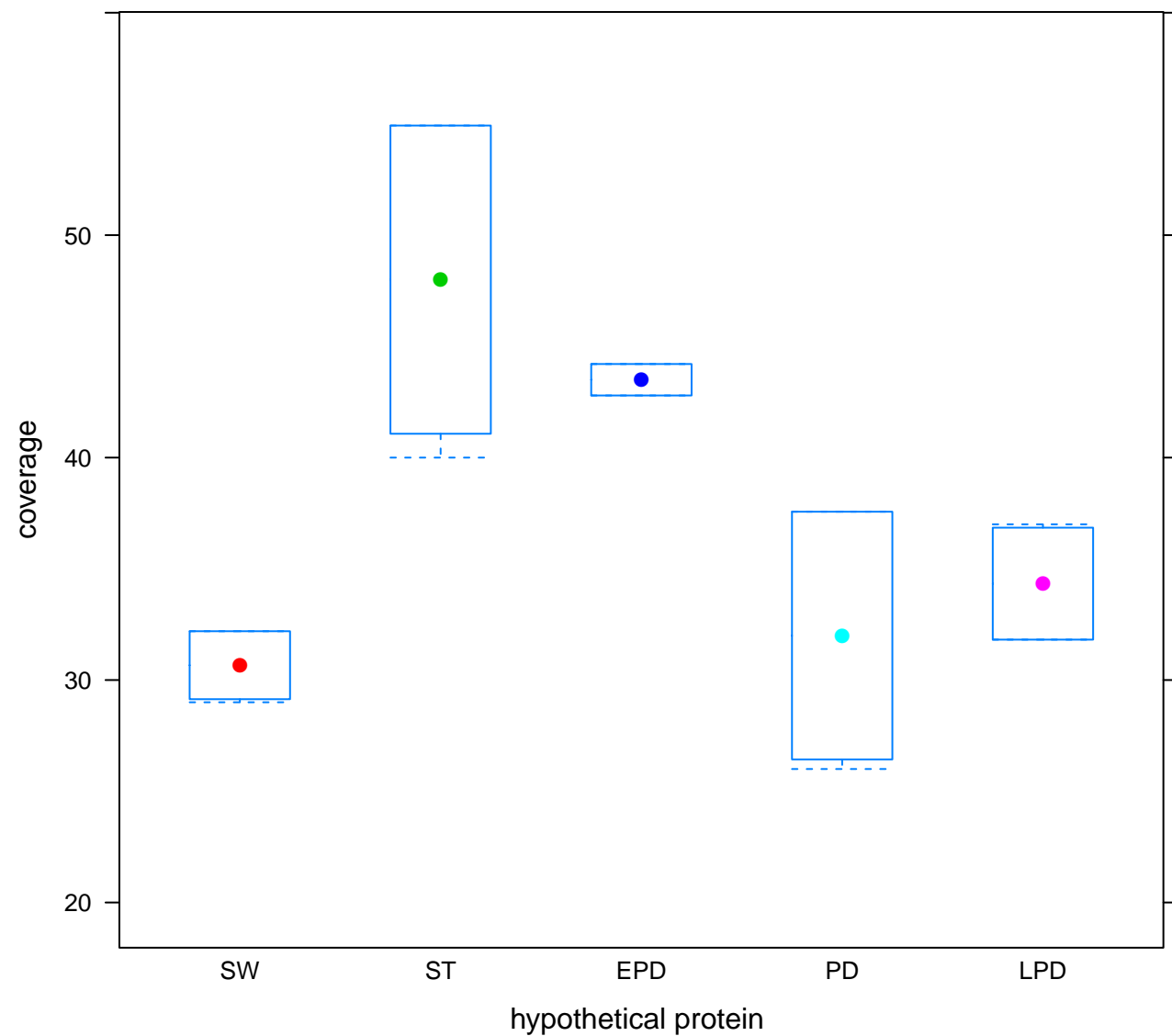

**Fold of change: 1.53**  
**baySeq likelihood: 0.592**

Supplement: Additional file 9: Figure S2 — Expression profiles of all identified CCR genes. [file 1471-2164-14-450-S9.zip › FigureS2/CCNA_00040.pdf]

# CCNA\_00042

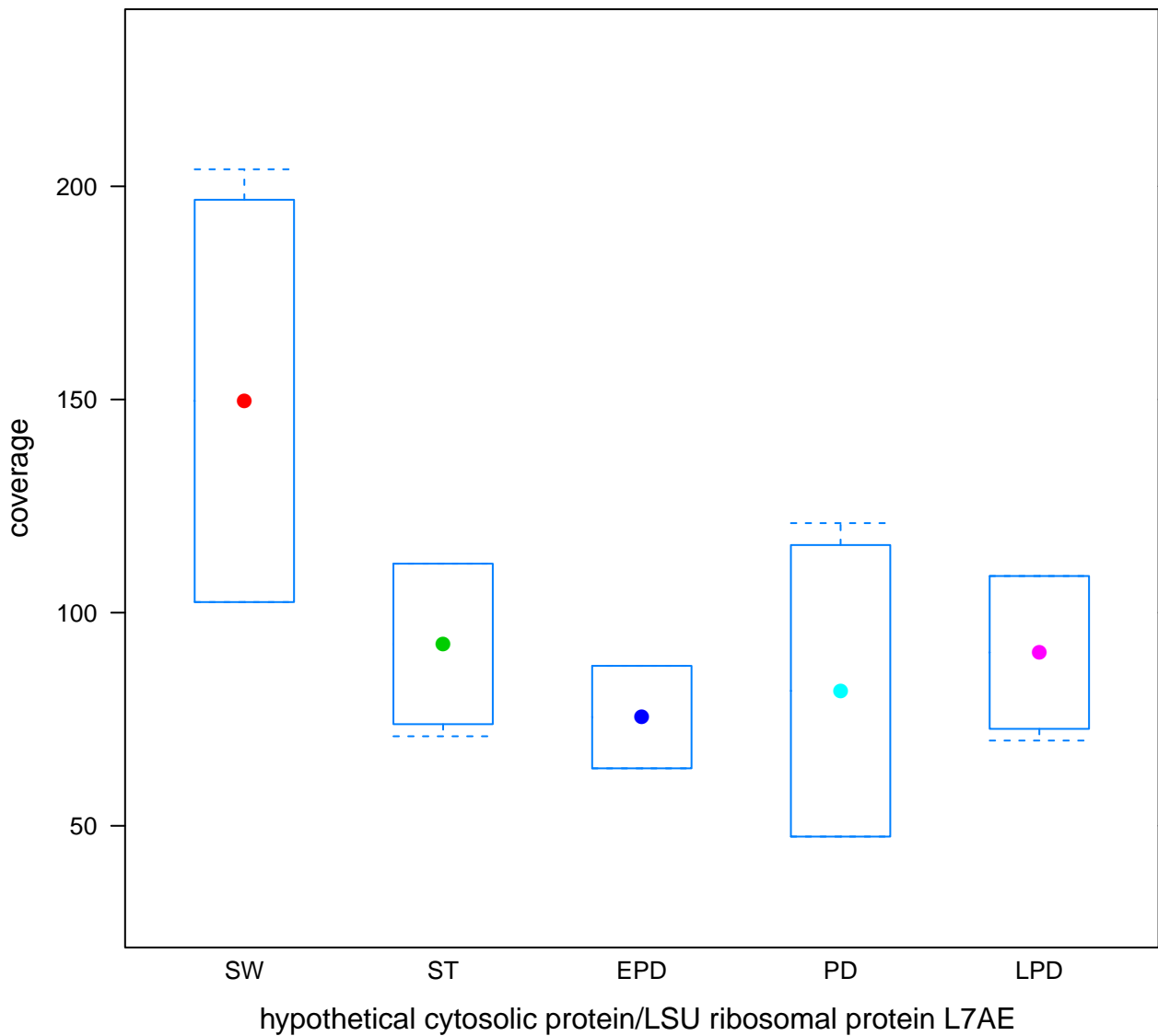

**Fold of change: 2.19**  
**baySeq likelihood: 0.44**

Supplement: Additional file 9: Figure S2 — Expression profiles of all identified CCR genes. [file 1471-2164-14-450-S9.zip › FigureS2/CCNA_00042.pdf]

# CCNA\_00045

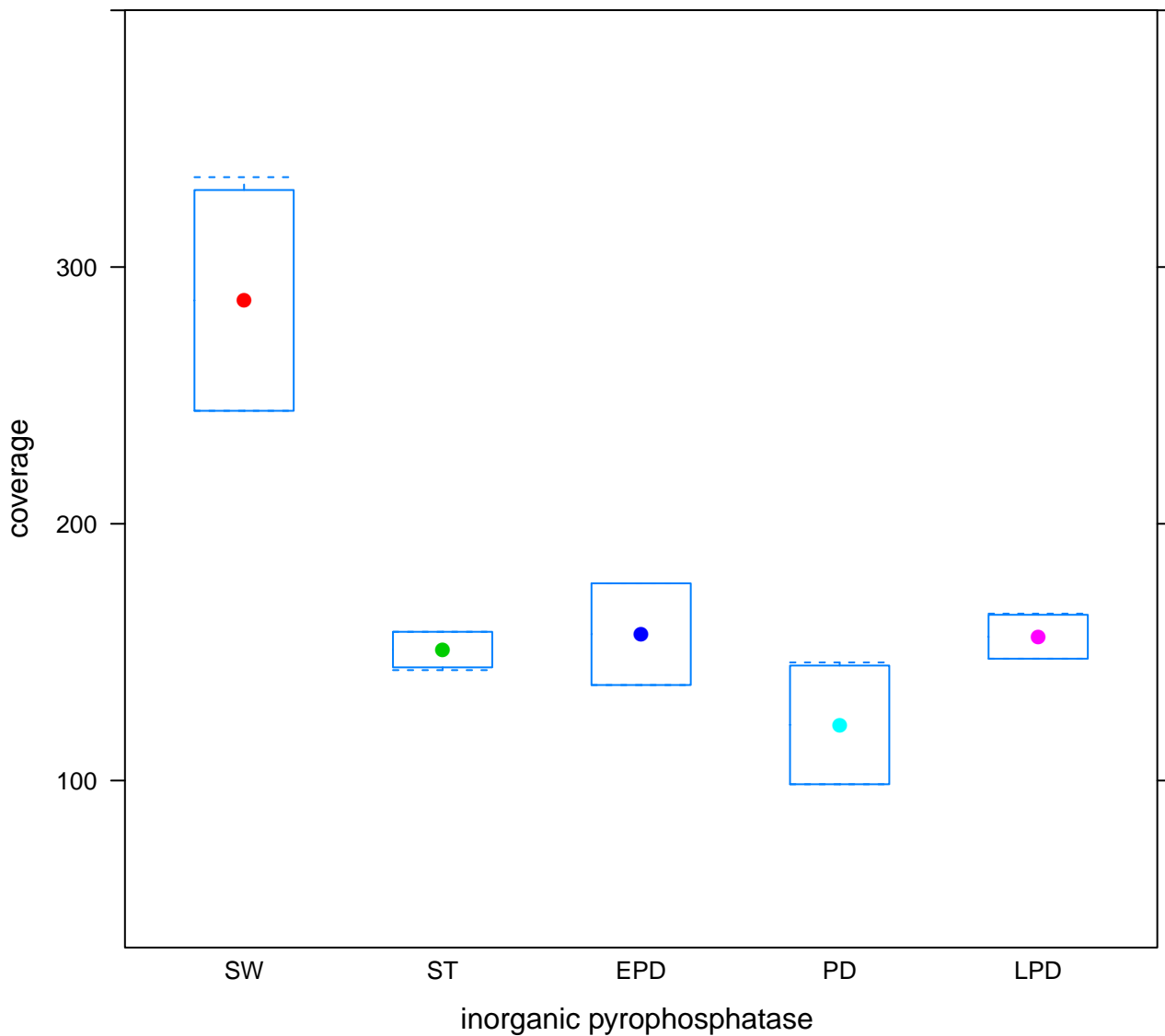

**Fold of change: 2.5**  
**baySeq likelihood: 0.998**

Supplement: Additional file 9: Figure S2 — Expression profiles of all identified CCR genes. [file 1471-2164-14-450-S9.zip › FigureS2/CCNA_00045.pdf]

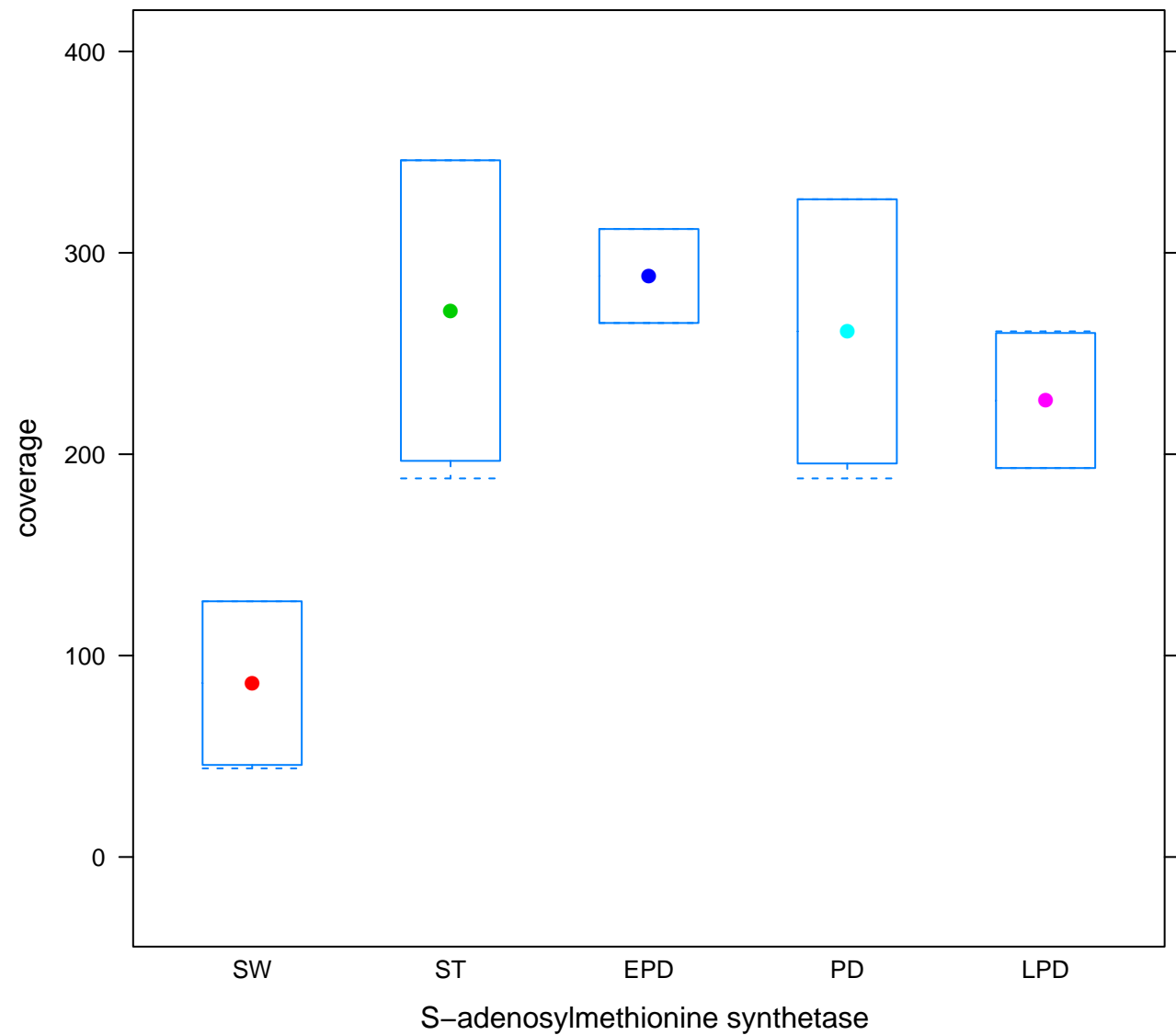

**Fold of change: 4.67**  
**baySeq likelihood: 0.993**

Supplement: Additional file 9: Figure S2 — Expression profiles of all identified CCR genes. [file 1471-2164-14-450-S9.zip › FigureS2/CCNA_00048.pdf]

# CCNA\_00052

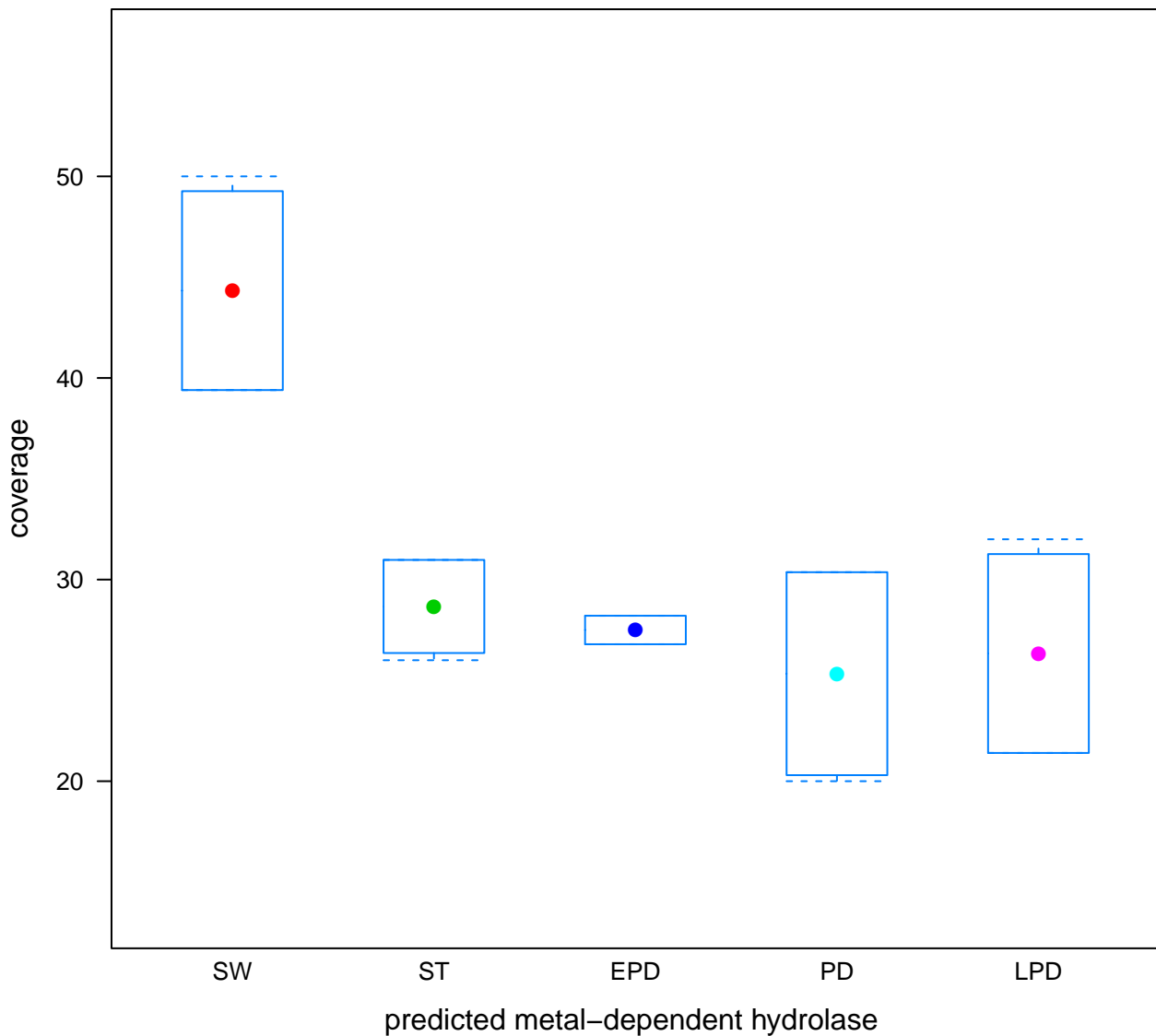

**Fold of change: 1.82**  
**baySeq likelihood: 0.989**

Supplement: Additional file 9: Figure S2 — Expression profiles of all identified CCR genes. [file 1471-2164-14-450-S9.zip › FigureS2/CCNA_00052.pdf]

# phoH;CCNA\_00053

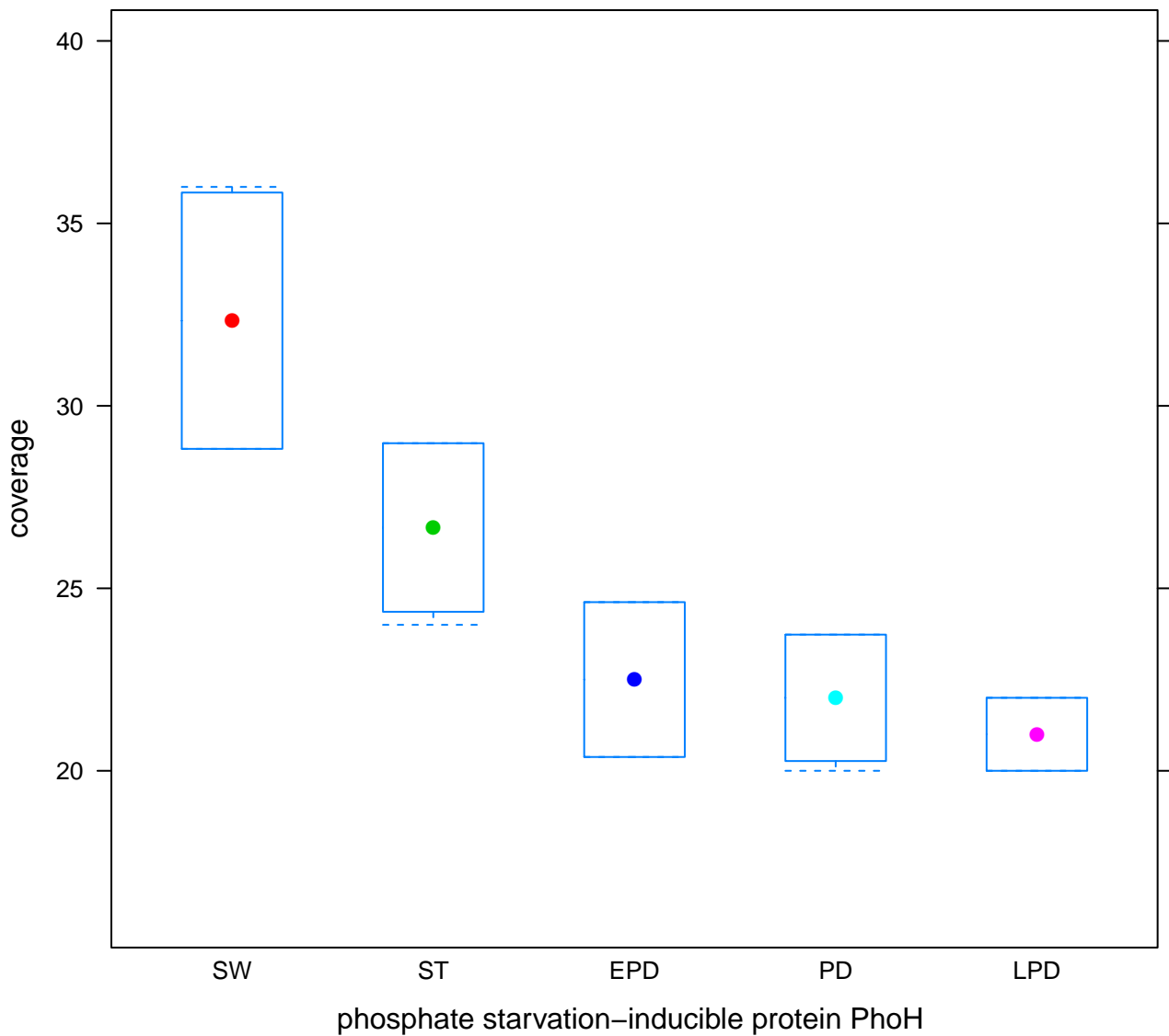

**Fold of change: 1.62**  
**baySeq likelihood: 0.505**

Supplement: Additional file 9: Figure S2 — Expression profiles of all identified CCR genes. [file 1471-2164-14-450-S9.zip › FigureS2/CCNA_00053.pdf]

# CCNA\_00054

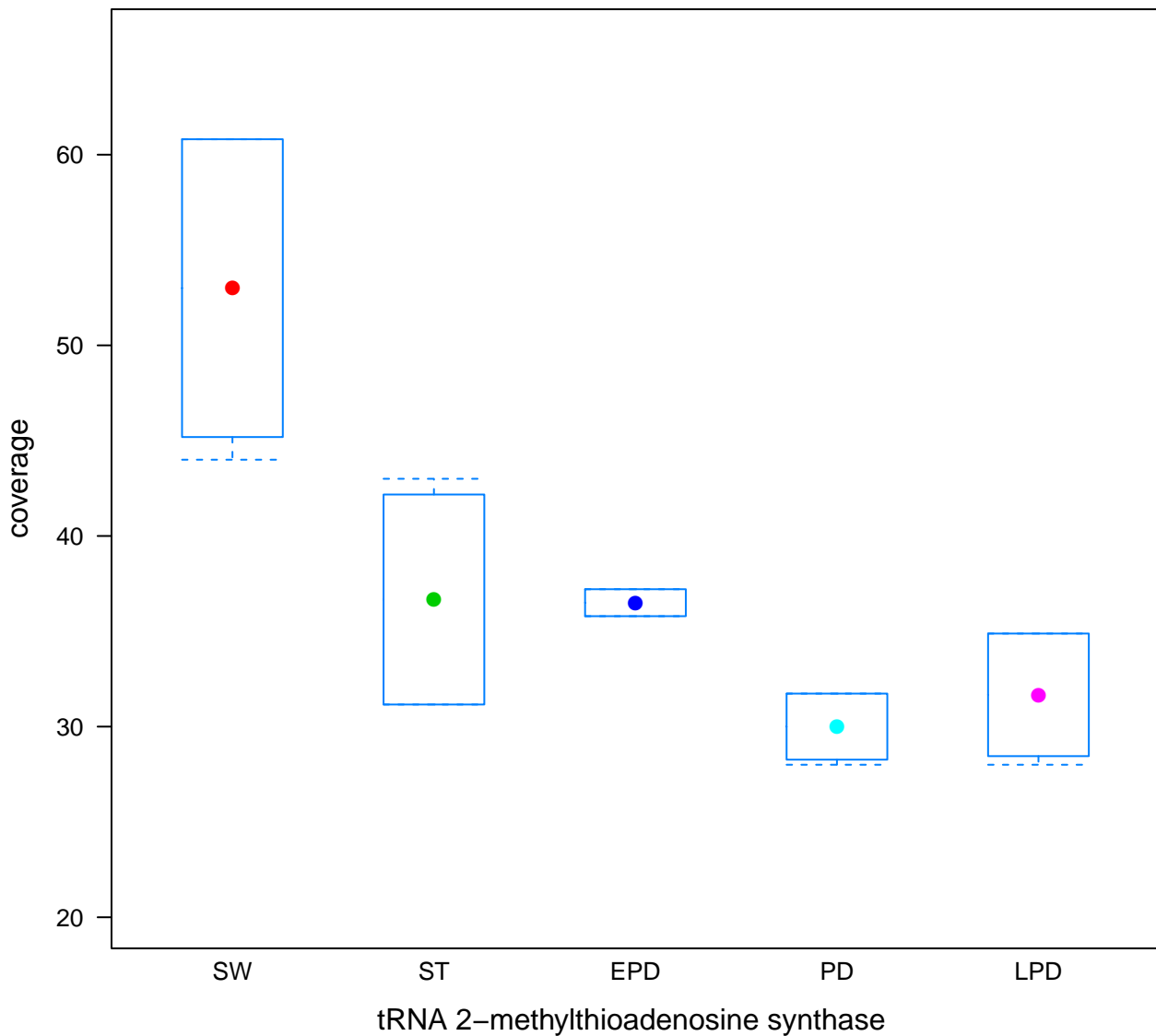

**Fold of change: 1.92**  
**baySeq likelihood: 0.966**

Supplement: Additional file 9: Figure S2 — Expression profiles of all identified CCR genes. [file 1471-2164-14-450-S9.zip › FigureS2/CCNA_00054.pdf]

# CCNA\_00055

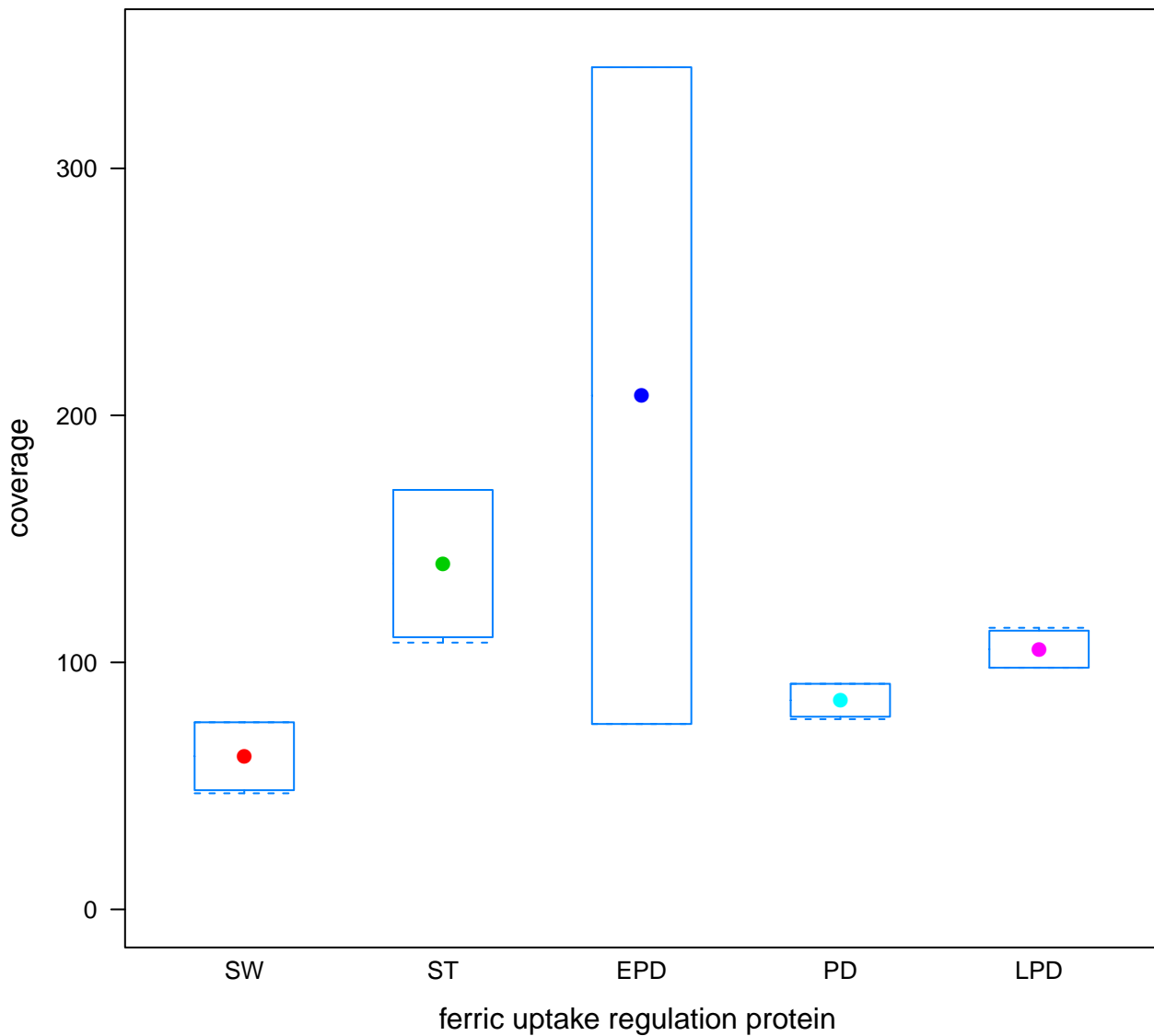

**Fold of change: 3.71**  
**baySeq likelihood: 0.457**

Supplement: Additional file 9: Figure S2 — Expression profiles of all identified CCR genes. [file 1471-2164-14-450-S9.zip › FigureS2/CCNA_00055.pdf]

# trpS;CCNA\_00062

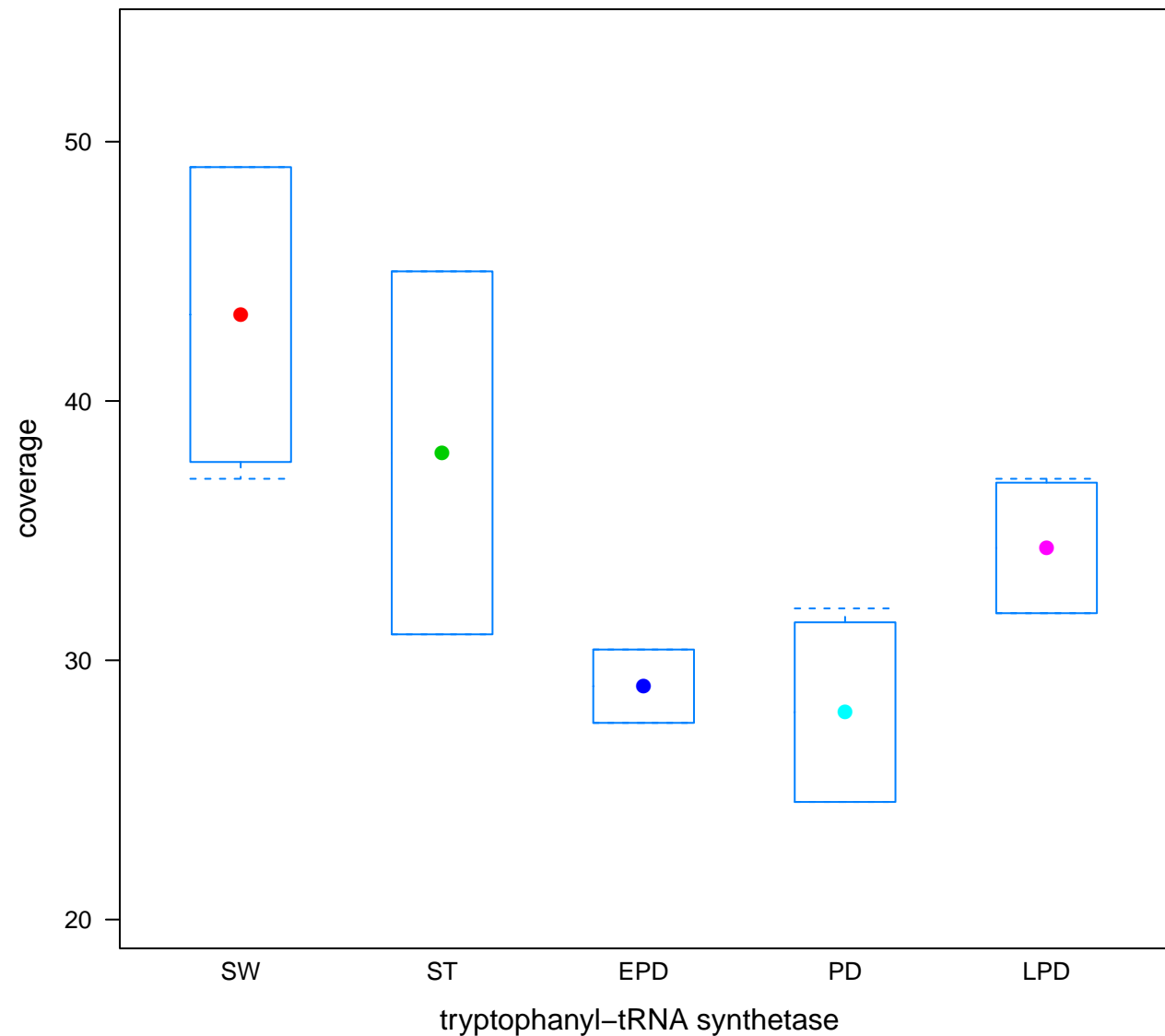

**Fold of change: 1.52**  
**baySeq likelihood: 0.287**

Supplement: Additional file 9: Figure S2 — Expression profiles of all identified CCR genes. [file 1471-2164-14-450-S9.zip › FigureS2/CCNA_00062.pdf]

# CCNA\_00065

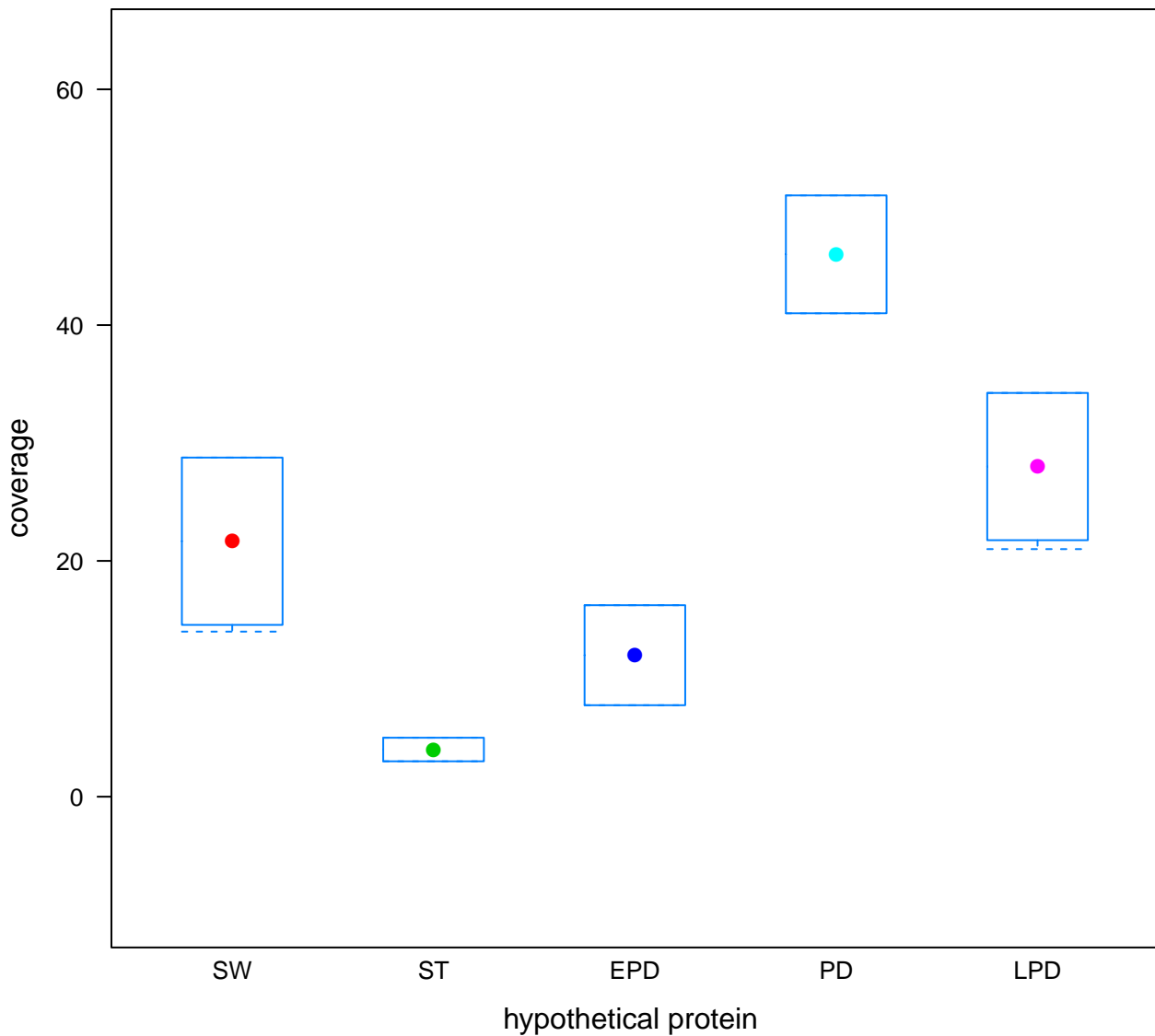

**Fold of change: 11.5**  
**baySeq likelihood: 0.584**

Supplement: Additional file 9: Figure S2 — Expression profiles of all identified CCR genes. [file 1471-2164-14-450-S9.zip › FigureS2/CCNA_00065.pdf]

# CCNA\_00070

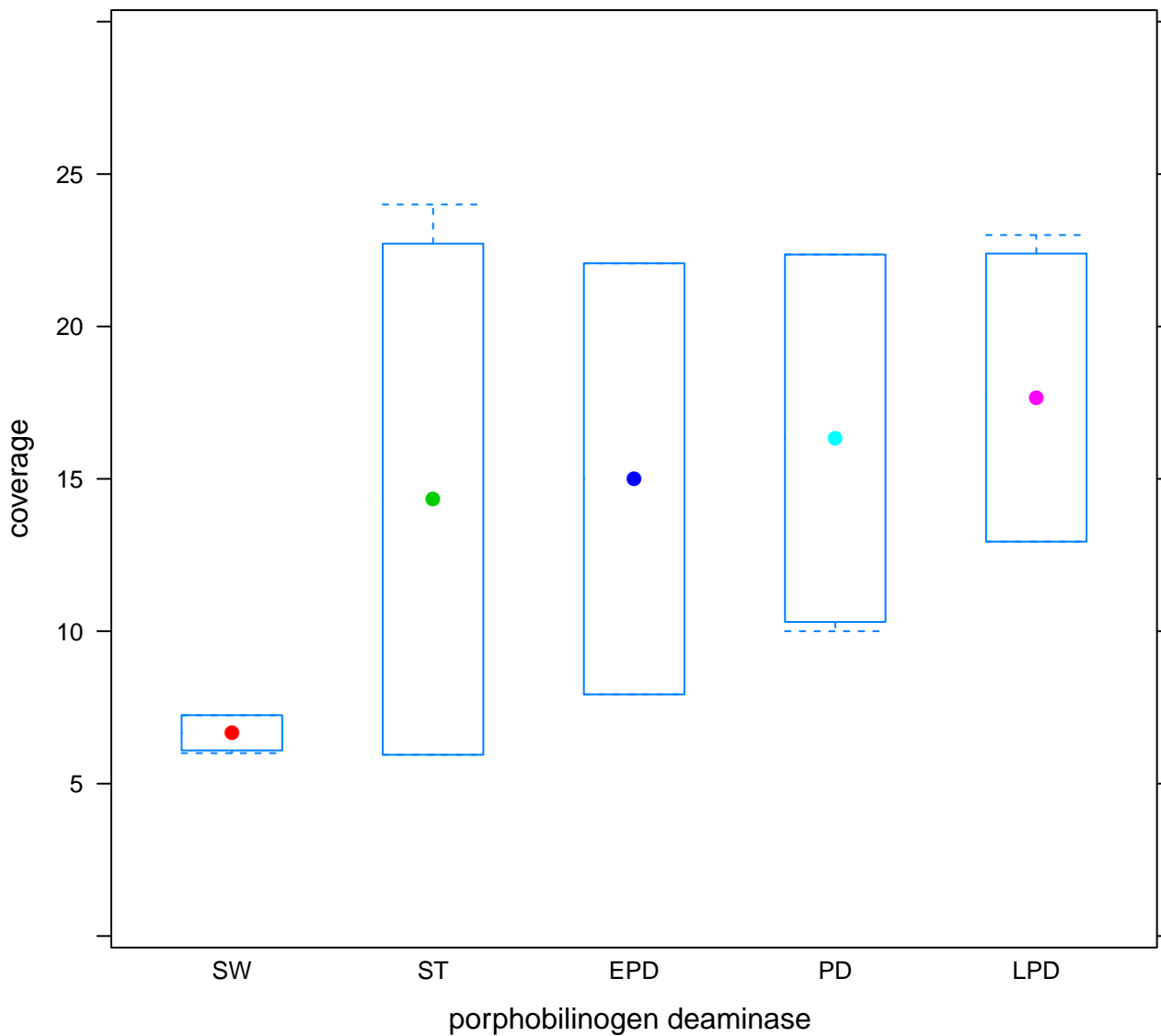

**Fold of change: 2.72**  
**baySeq likelihood: 0.883**

Supplement: Additional file 9: Figure S2 — Expression profiles of all identified CCR genes. [file 1471-2164-14-450-S9.zip › FigureS2/CCNA_00070.pdf]

# CCNA\_00071

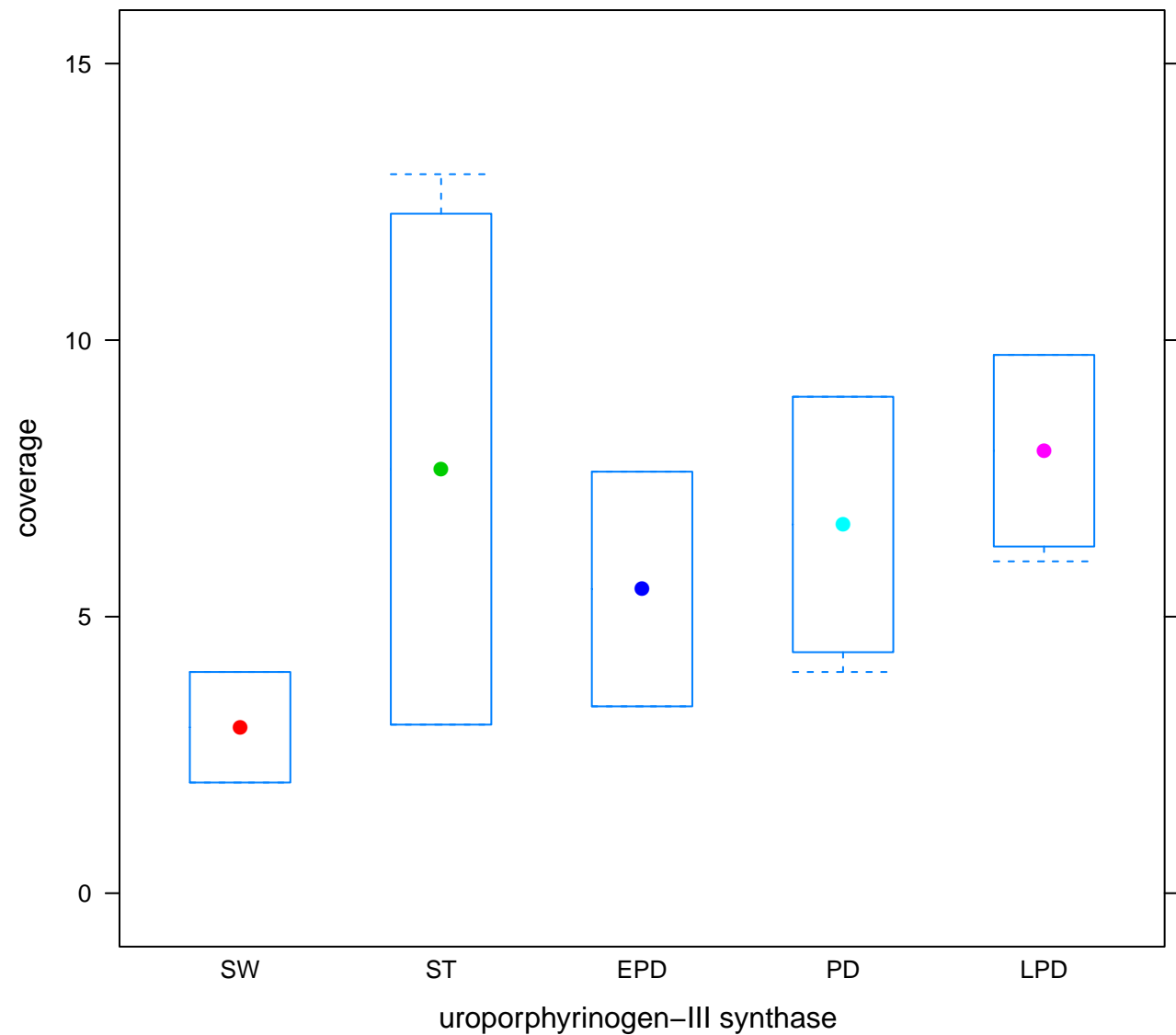

**Fold of change: 2.29**  
**baySeq likelihood: 0.453**

Supplement: Additional file 9: Figure S2 — Expression profiles of all identified CCR genes. [file 1471-2164-14-450-S9.zip › FigureS2/CCNA_00071.pdf]

# CCNA\_00072

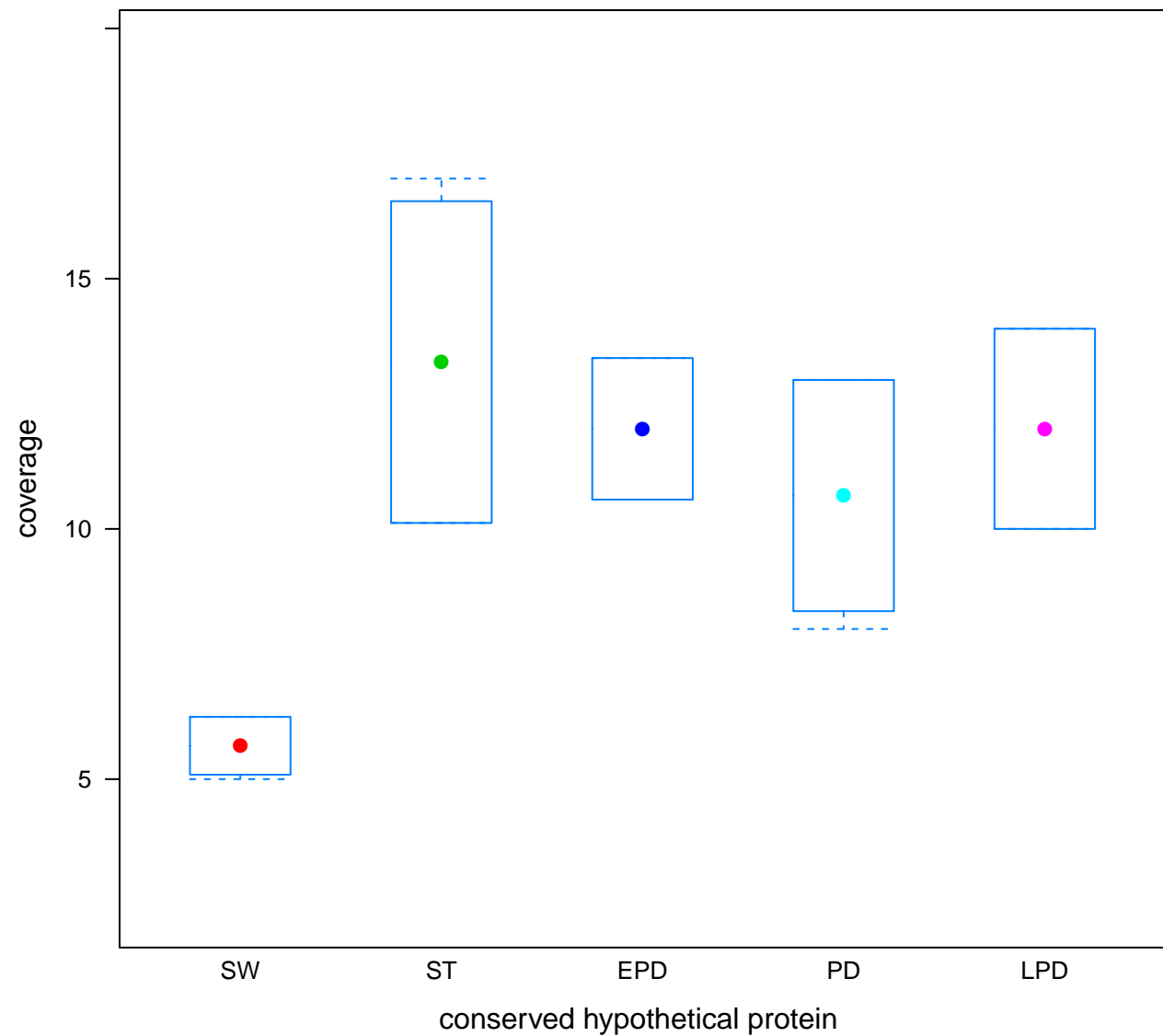

**Fold of change: 2.18**  
**baySeq likelihood: 0.742**

Supplement: Additional file 9: Figure S2 — Expression profiles of all identified CCR genes. [file 1471-2164-14-450-S9.zip › FigureS2/CCNA_00072.pdf]

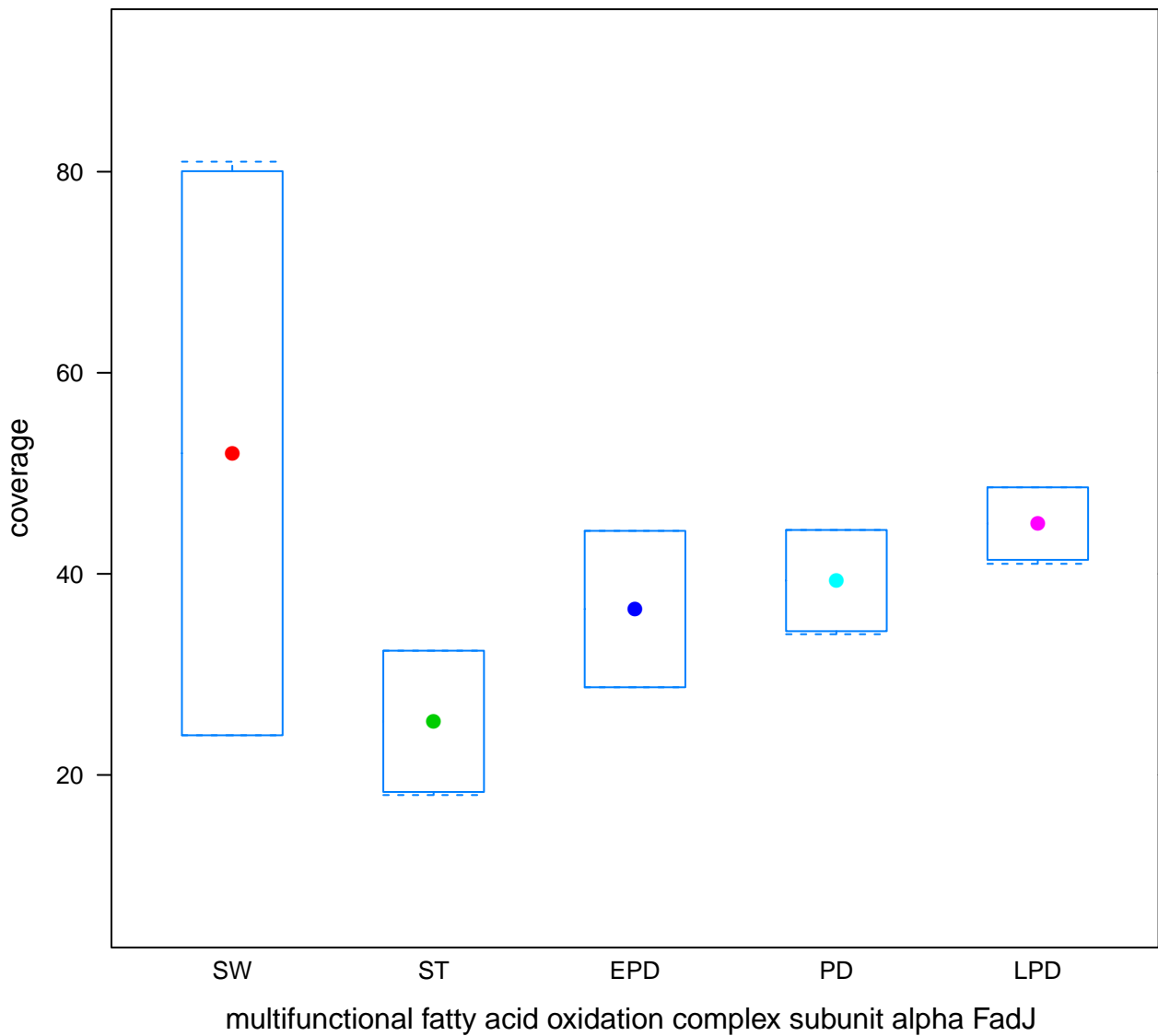

**Fold of change: 2.05**  
**baySeq likelihood: 0.266**

Supplement: Additional file 9: Figure S2 — Expression profiles of all identified CCR genes. [file 1471-2164-14-450-S9.zip › FigureS2/CCNA_00074.pdf]

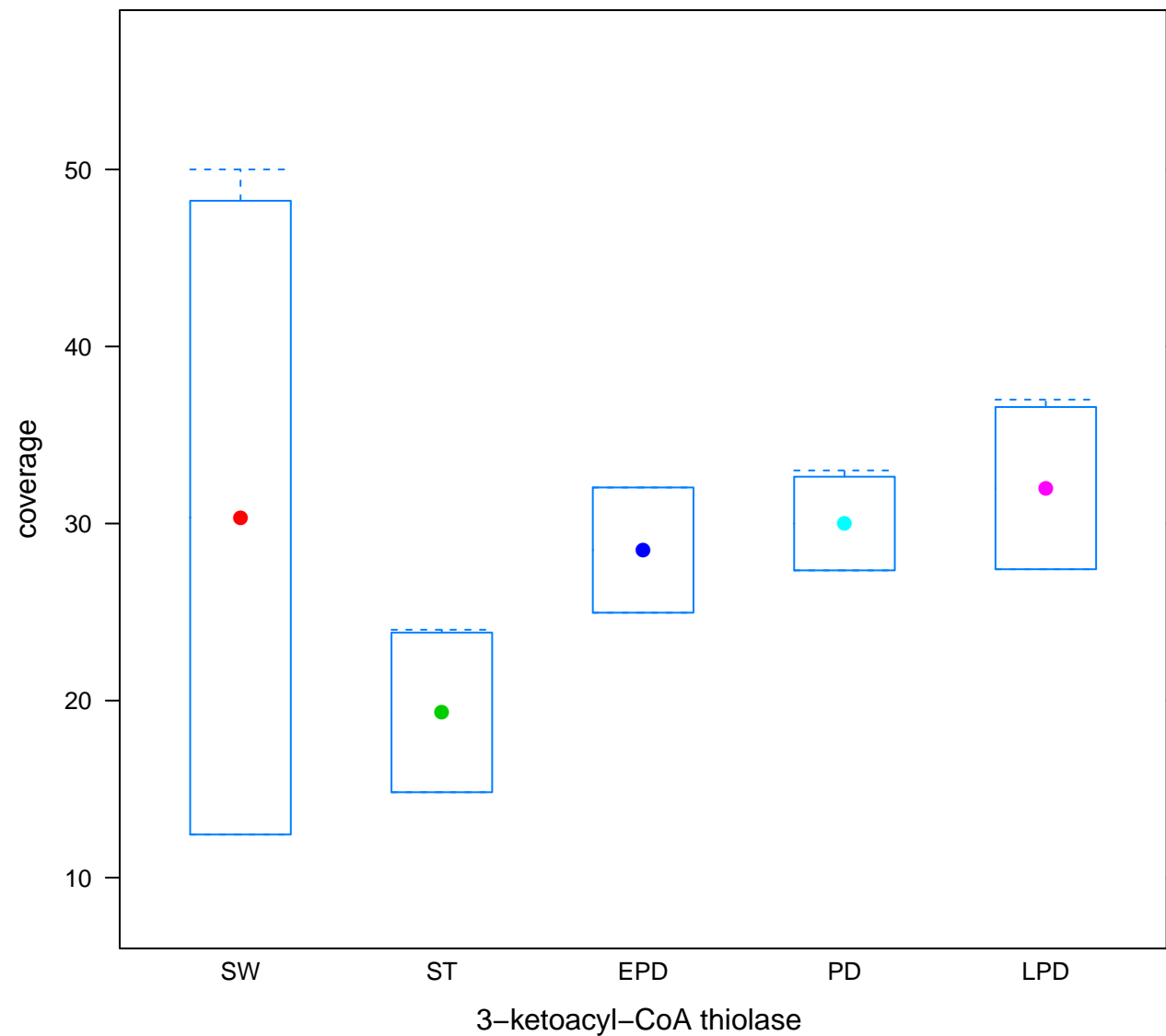

**Fold of change: 1.88**  
**baySeq likelihood: 0.251**

Supplement: Additional file 9: Figure S2 — Expression profiles of all identified CCR genes. [file 1471-2164-14-450-S9.zip › FigureS2/CCNA_00075.pdf]

# CCNA\_00077

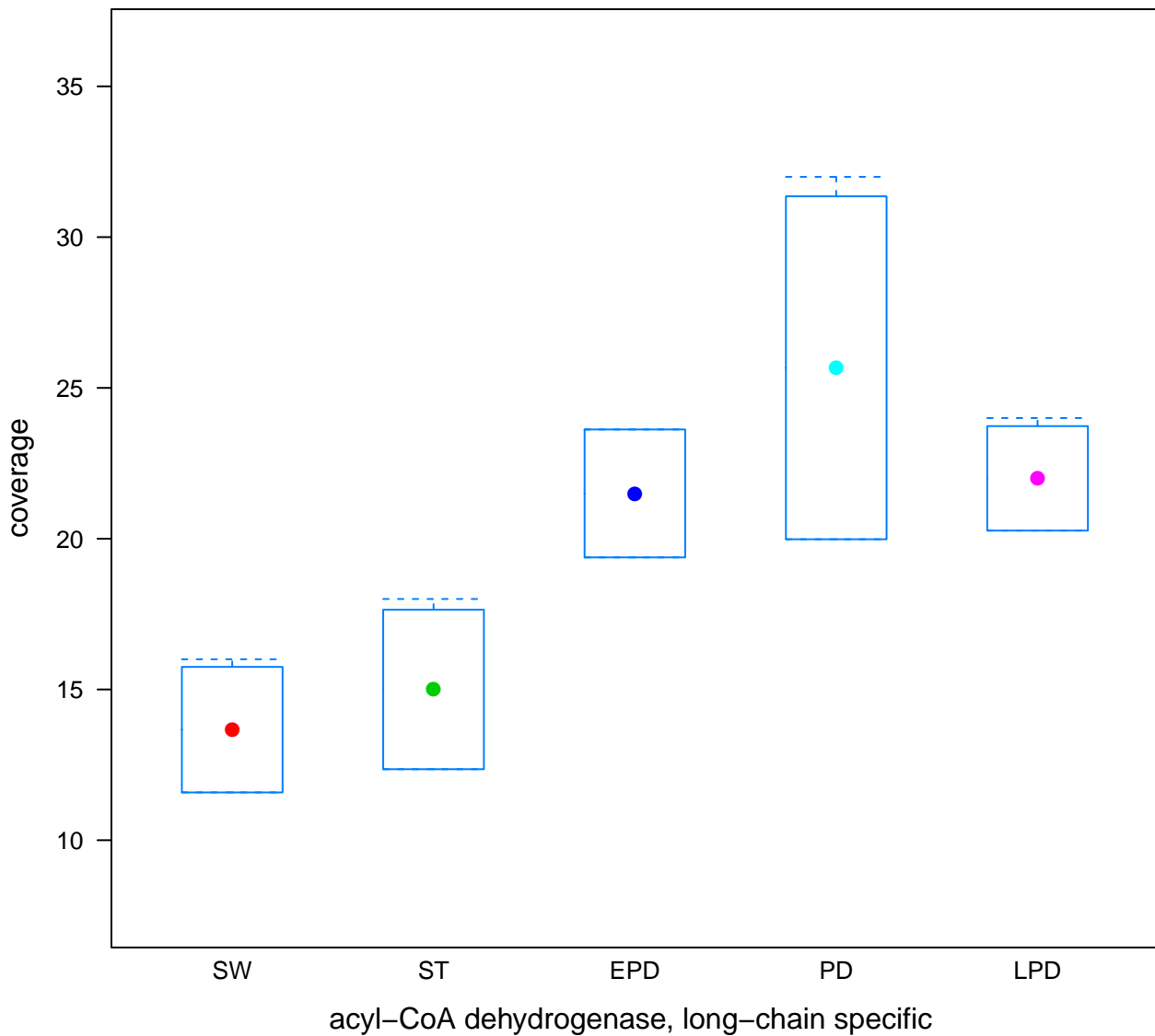

**Fold of change: 2.05**  
**baySeq likelihood: 0.705**

Supplement: Additional file 9: Figure S2 — Expression profiles of all identified CCR genes. [file 1471-2164-14-450-S9.zip › FigureS2/CCNA_00077.pdf]

# CCNA\_00078

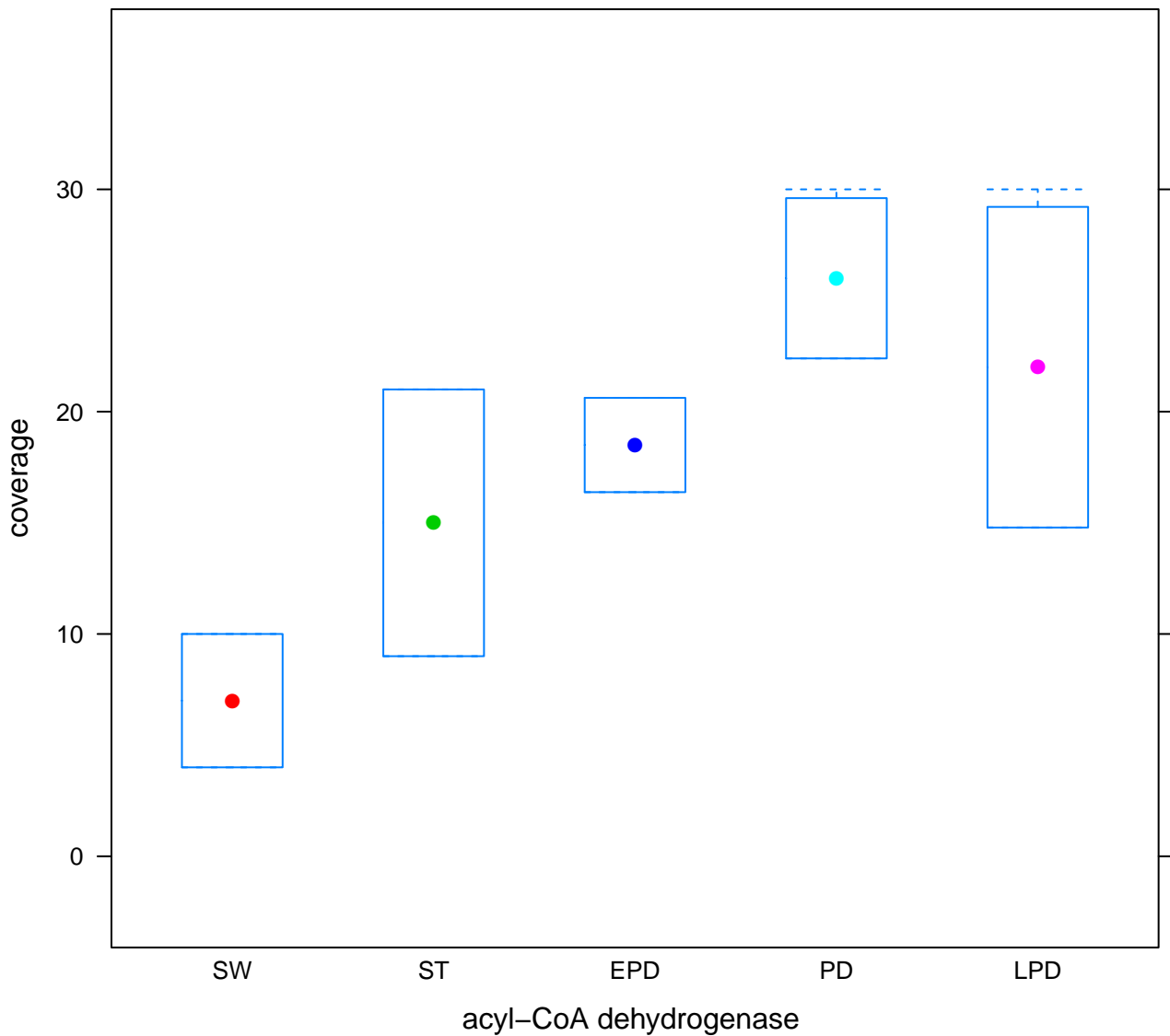

**Fold of change: 4.73**  
**baySeq likelihood: 0.831**

Supplement: Additional file 9: Figure S2 — Expression profiles of all identified CCR genes. [file 1471-2164-14-450-S9.zip › FigureS2/CCNA_00078.pdf]

# CCNA\_00079

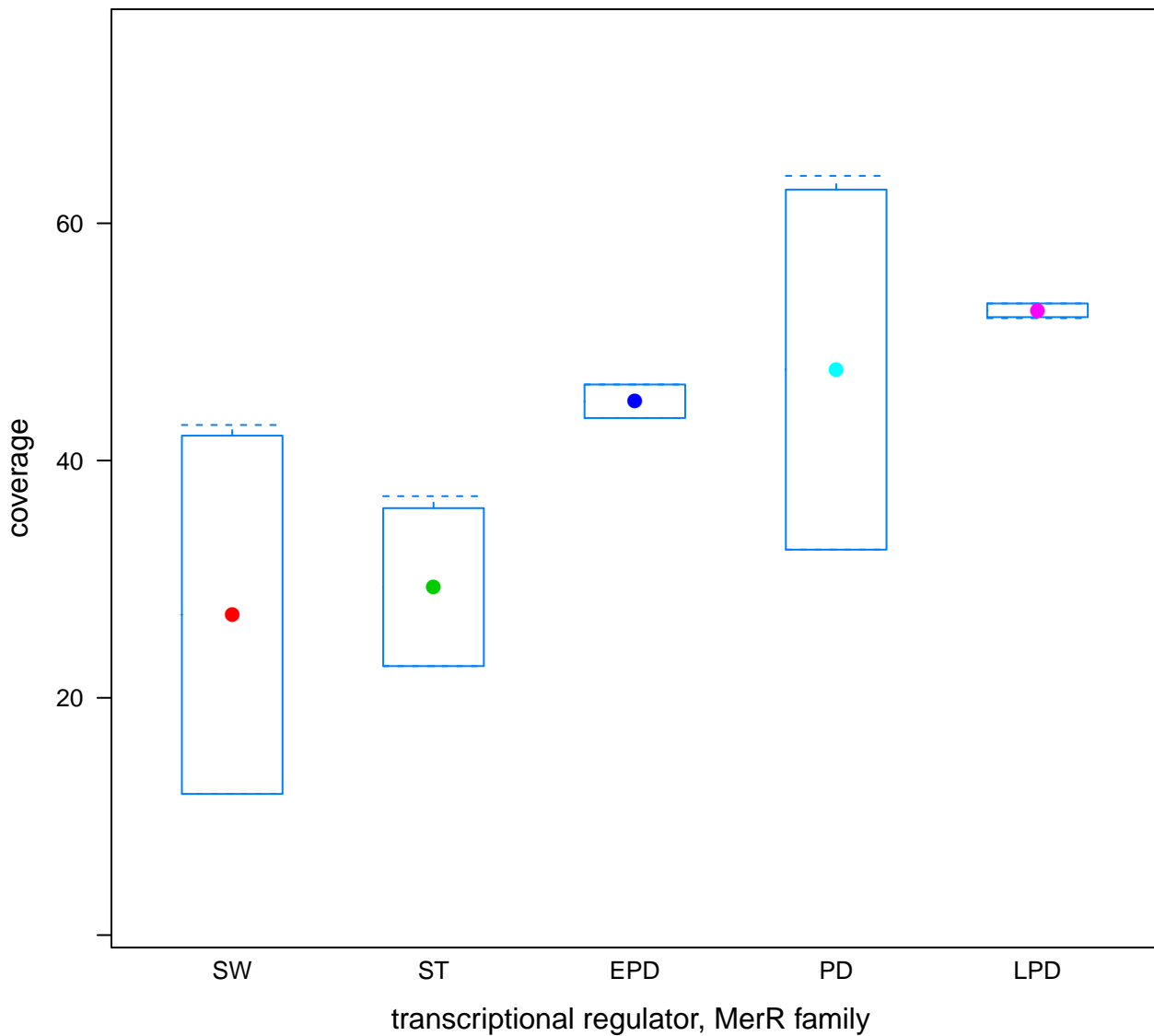

**Fold of change: 2.77**  
**baySeq likelihood: 0.751**

Supplement: Additional file 9: Figure S2 — Expression profiles of all identified CCR genes. [file 1471-2164-14-450-S9.zip › FigureS2/CCNA_00079.pdf]

# CCNA\_00081

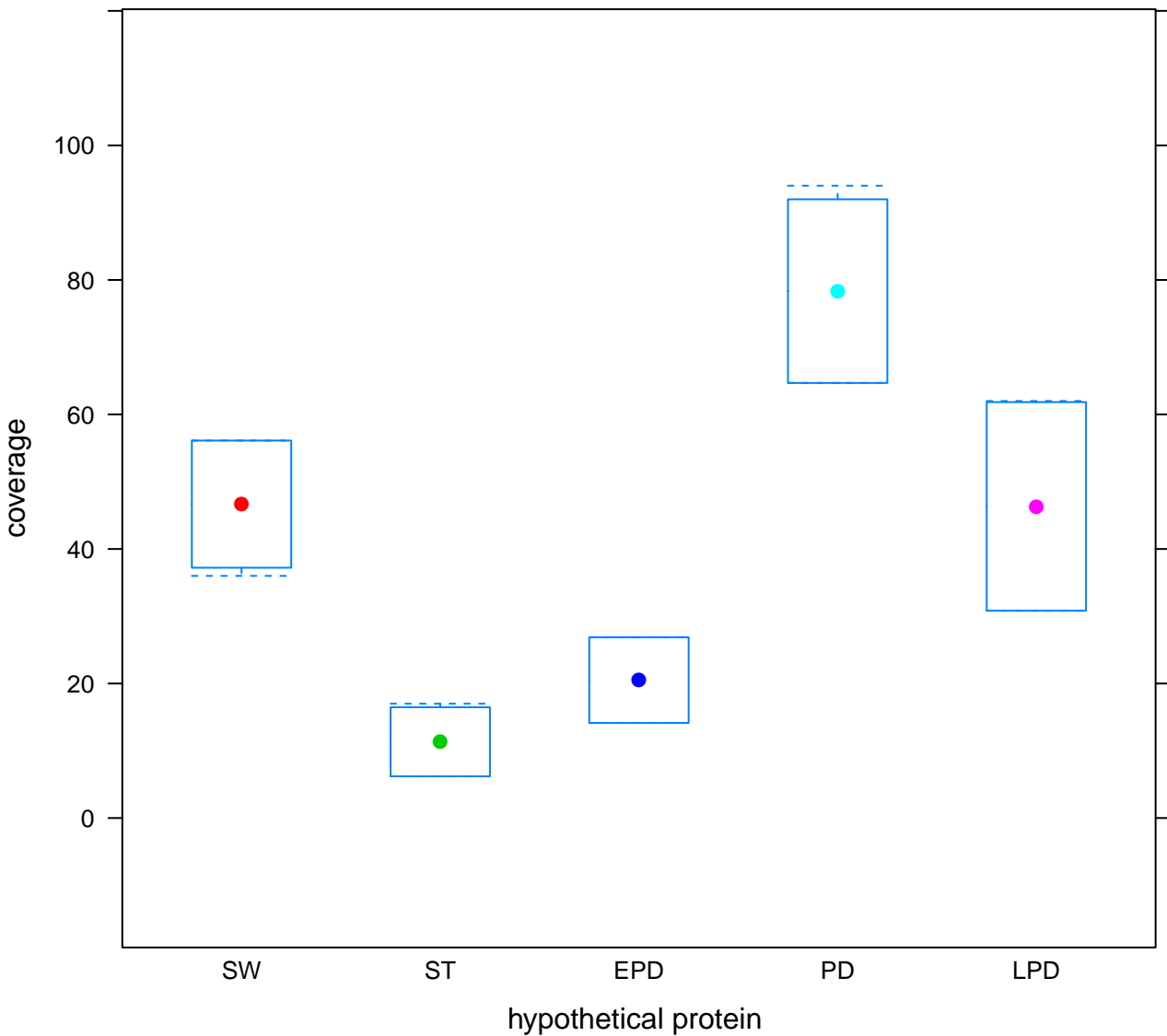

**Fold of change: 9.22**  
**baySeq likelihood: 0.936**

Supplement: Additional file 9: Figure S2 — Expression profiles of all identified CCR genes. [file 1471-2164-14-450-S9.zip › FigureS2/CCNA_00081.pdf]

# lysS;CCNA\_00082

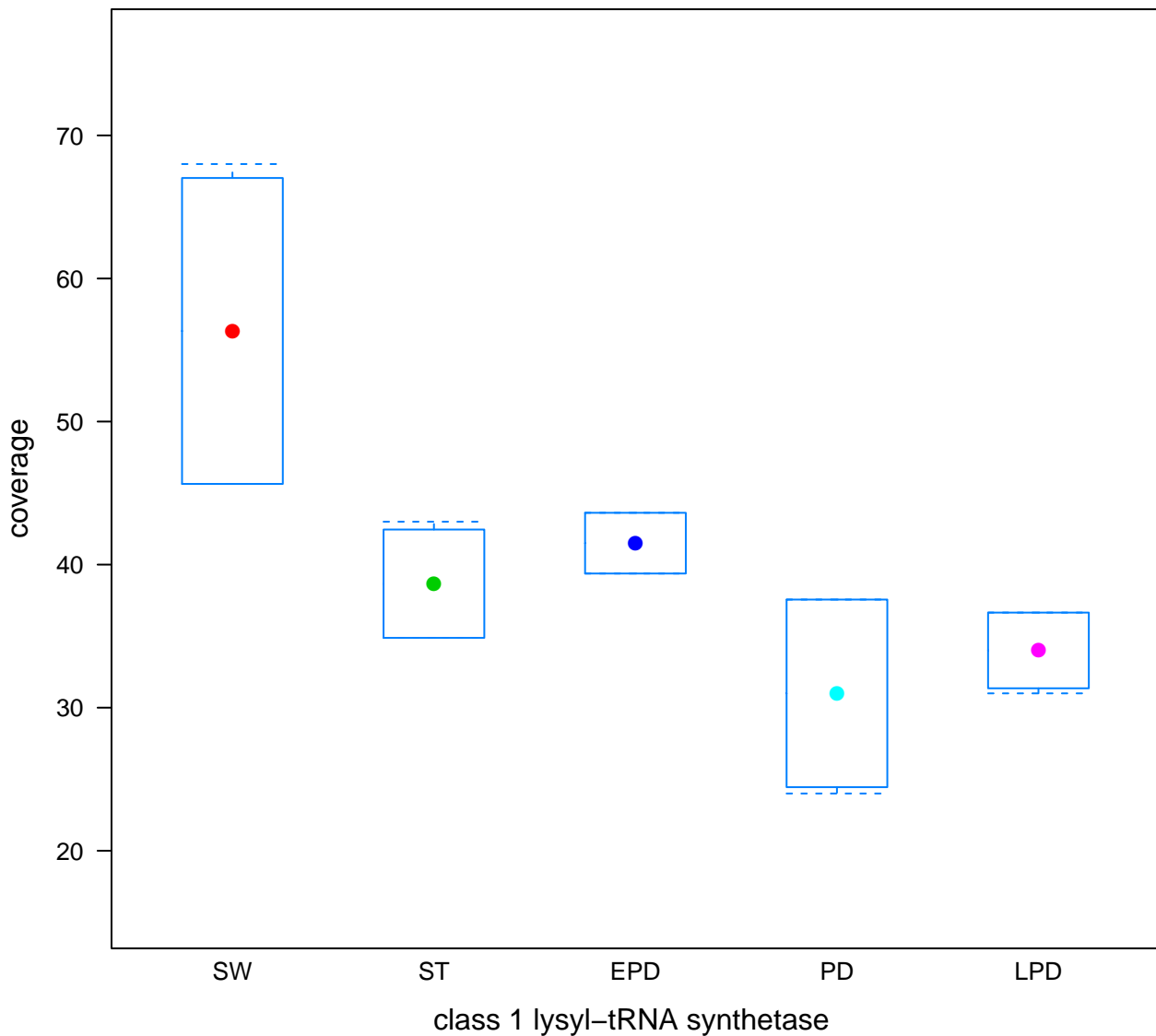

**Fold of change: 1.97**  
**baySeq likelihood: 0.941**

Supplement: Additional file 9: Figure S2 — Expression profiles of all identified CCR genes. [file 1471-2164-14-450-S9.zip › FigureS2/CCNA_00082.pdf]

# CCNA\_00085

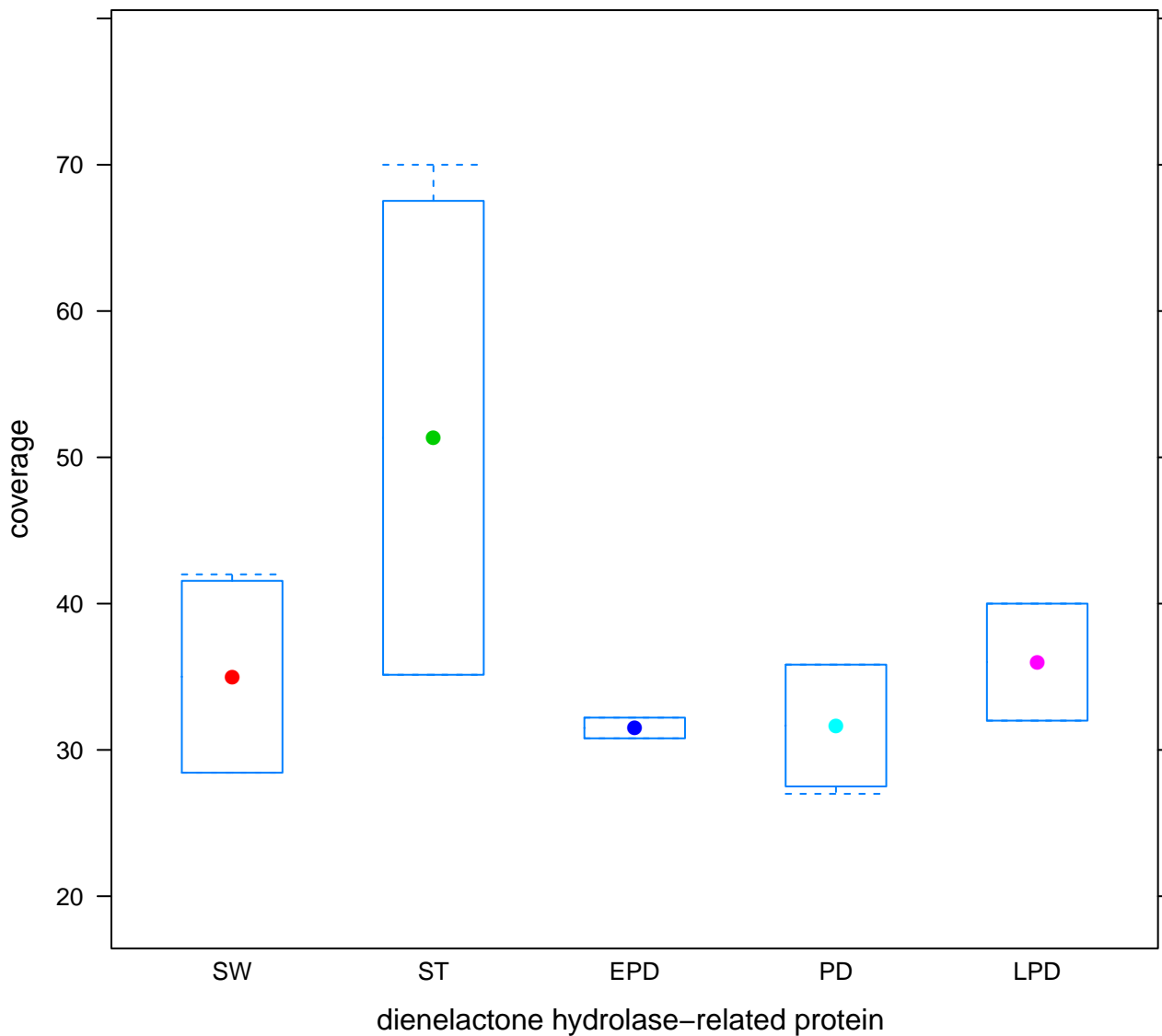

**Fold of change: 1.79**  
**baySeq likelihood: 0.398**

Supplement: Additional file 9: Figure S2 — Expression profiles of all identified CCR genes. [file 1471-2164-14-450-S9.zip › FigureS2/CCNA_00085.pdf]

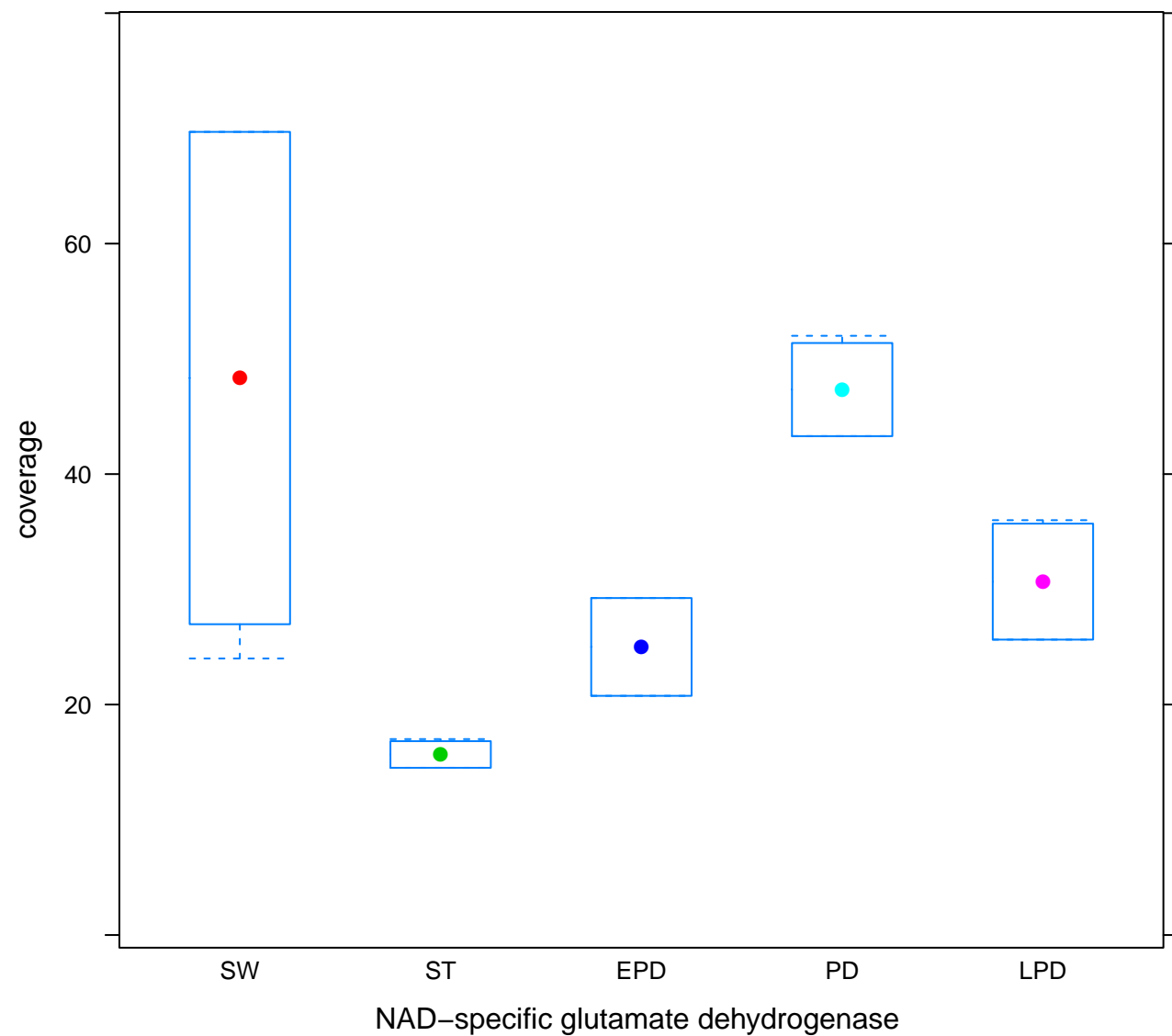

**Fold of change: 4.03**  
**baySeq likelihood: 0.493**

Supplement: Additional file 9: Figure S2 — Expression profiles of all identified CCR genes. [file 1471-2164-14-450-S9.zip › FigureS2/CCNA_00086.pdf]

# CCNA\_00089

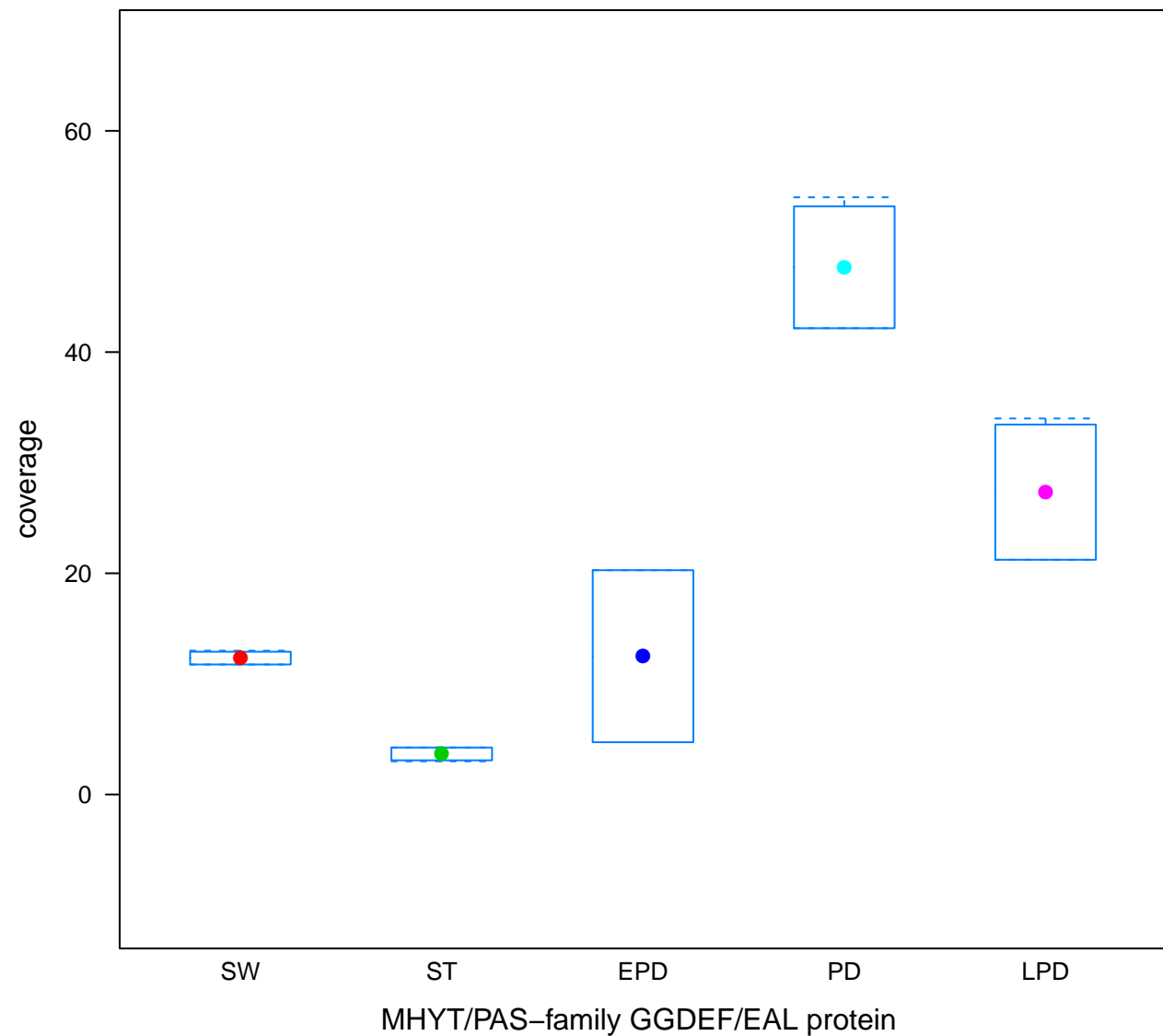

**Fold of change: 13.62**  
**baySeq likelihood: 0.652**

Supplement: Additional file 9: Figure S2 — Expression profiles of all identified CCR genes. [file 1471-2164-14-450-S9.zip › FigureS2/CCNA_00089.pdf]

# CCNA\_00093

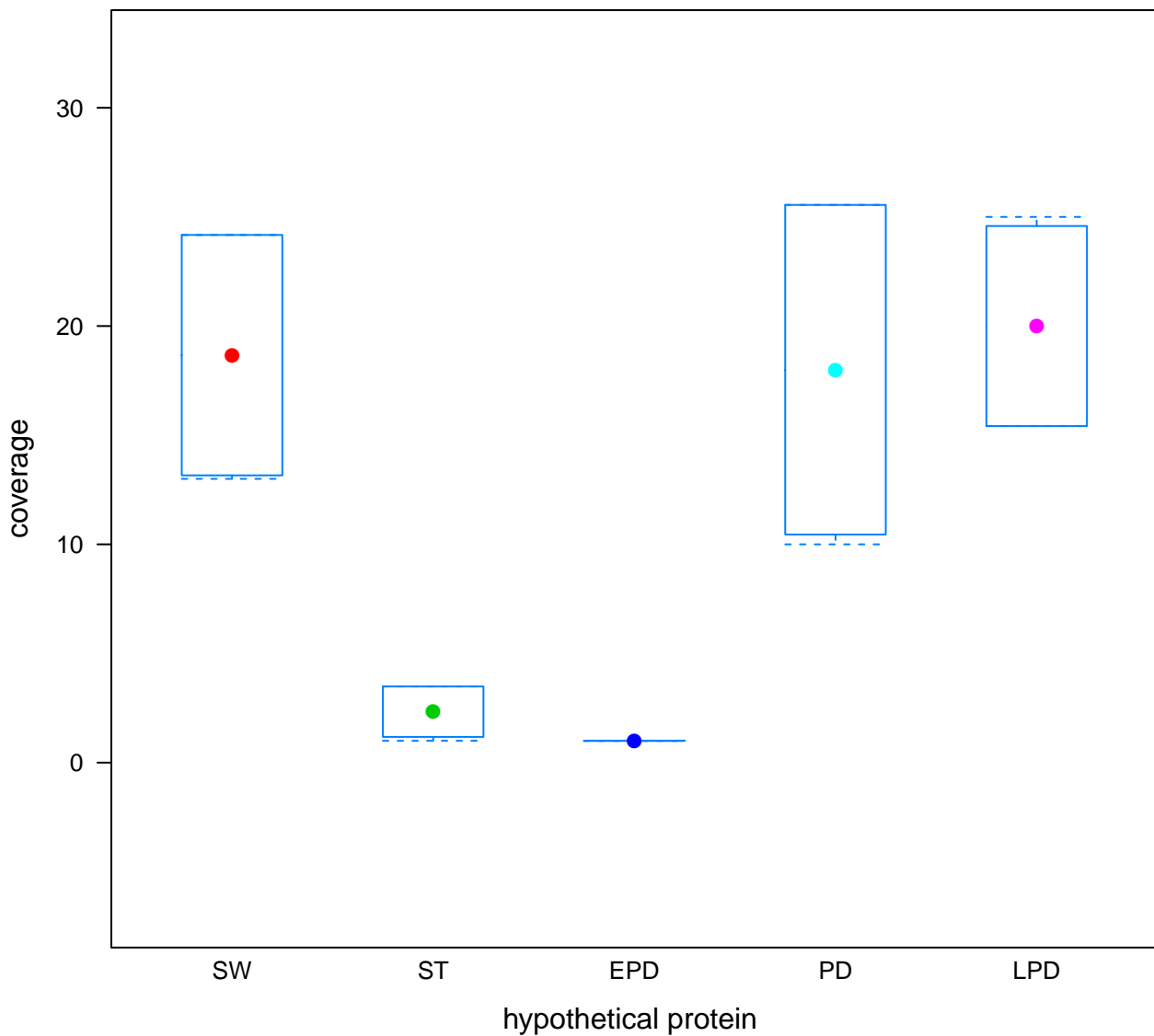

**Fold of change: 20**  
**baySeq likelihood: 1**

Supplement: Additional file 9: Figure S2 — Expression profiles of all identified CCR genes. [file 1471-2164-14-450-S9.zip › FigureS2/CCNA_00093.pdf]

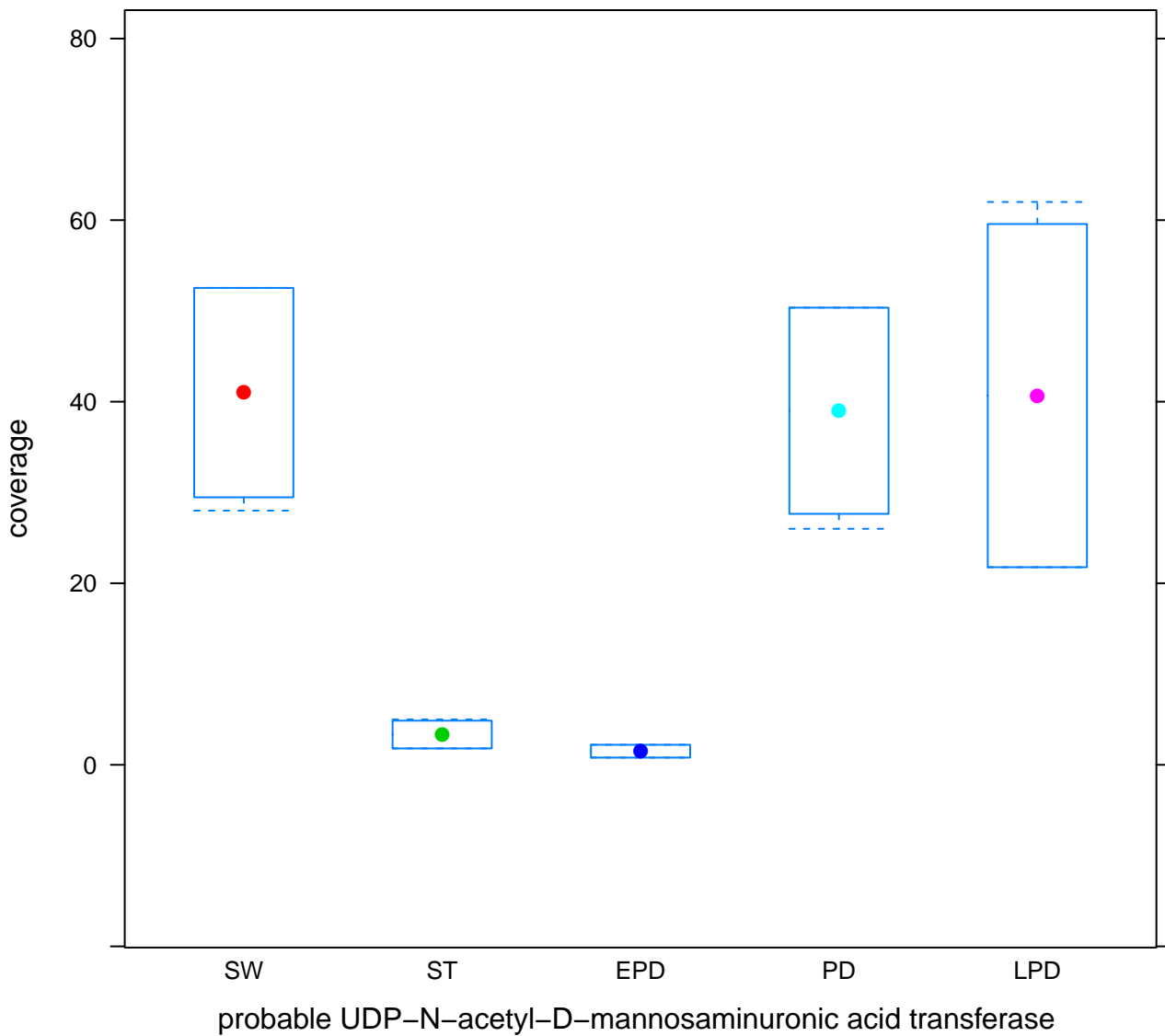

**Fold of change: 27.11**

**baySeq likelihood: 1**

Supplement: Additional file 9: Figure S2 — Expression profiles of all identified CCR genes. [file 1471-2164-14-450-S9.zip › FigureS2/CCNA_00094.pdf]

# CCNA\_00106

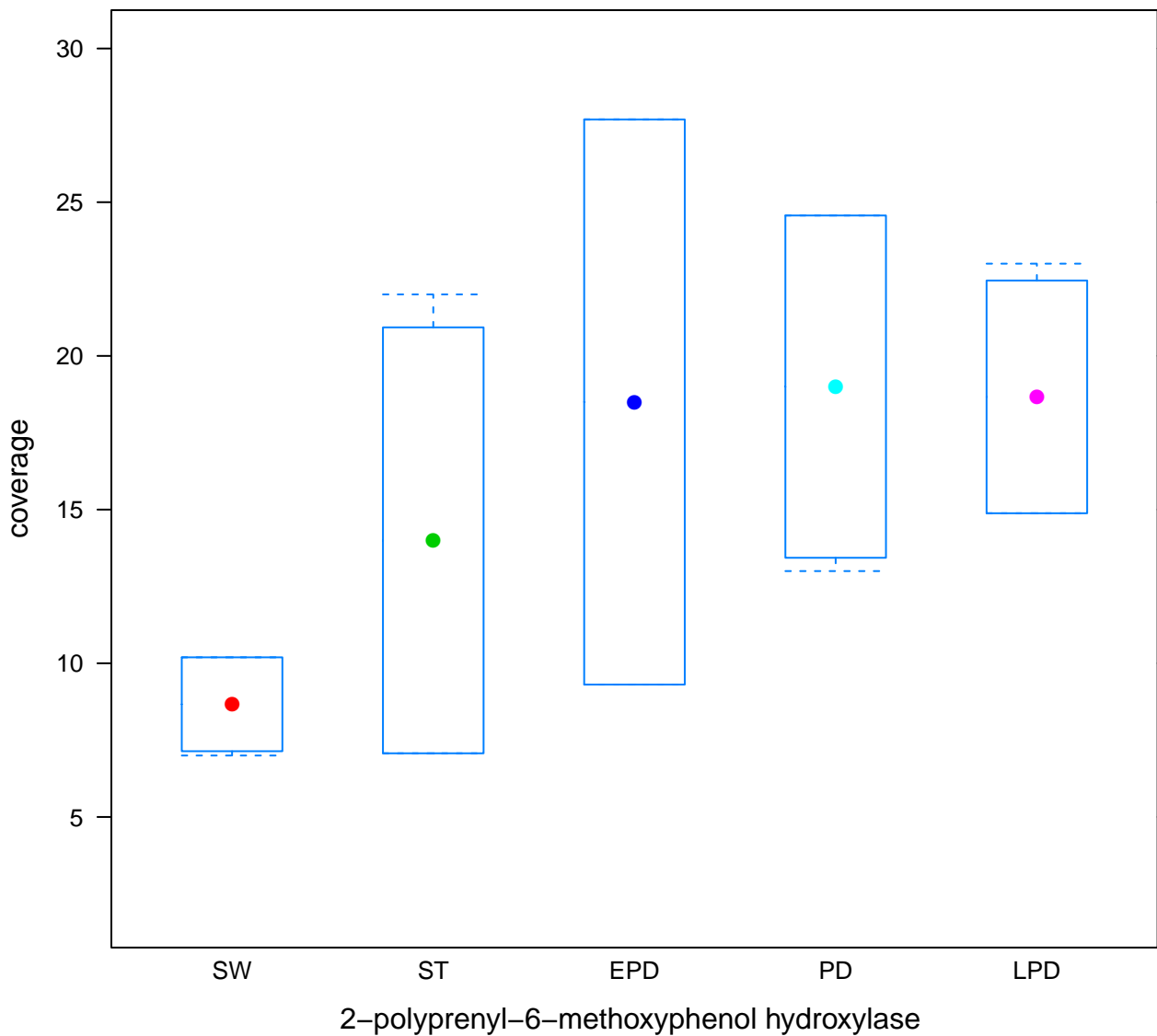

**Fold of change: 2.38**  
**baySeq likelihood: 0.623**

Supplement: Additional file 9: Figure S2 — Expression profiles of all identified CCR genes. [file 1471-2164-14-450-S9.zip › FigureS2/CCNA_00106.pdf]

# CCNA\_00111

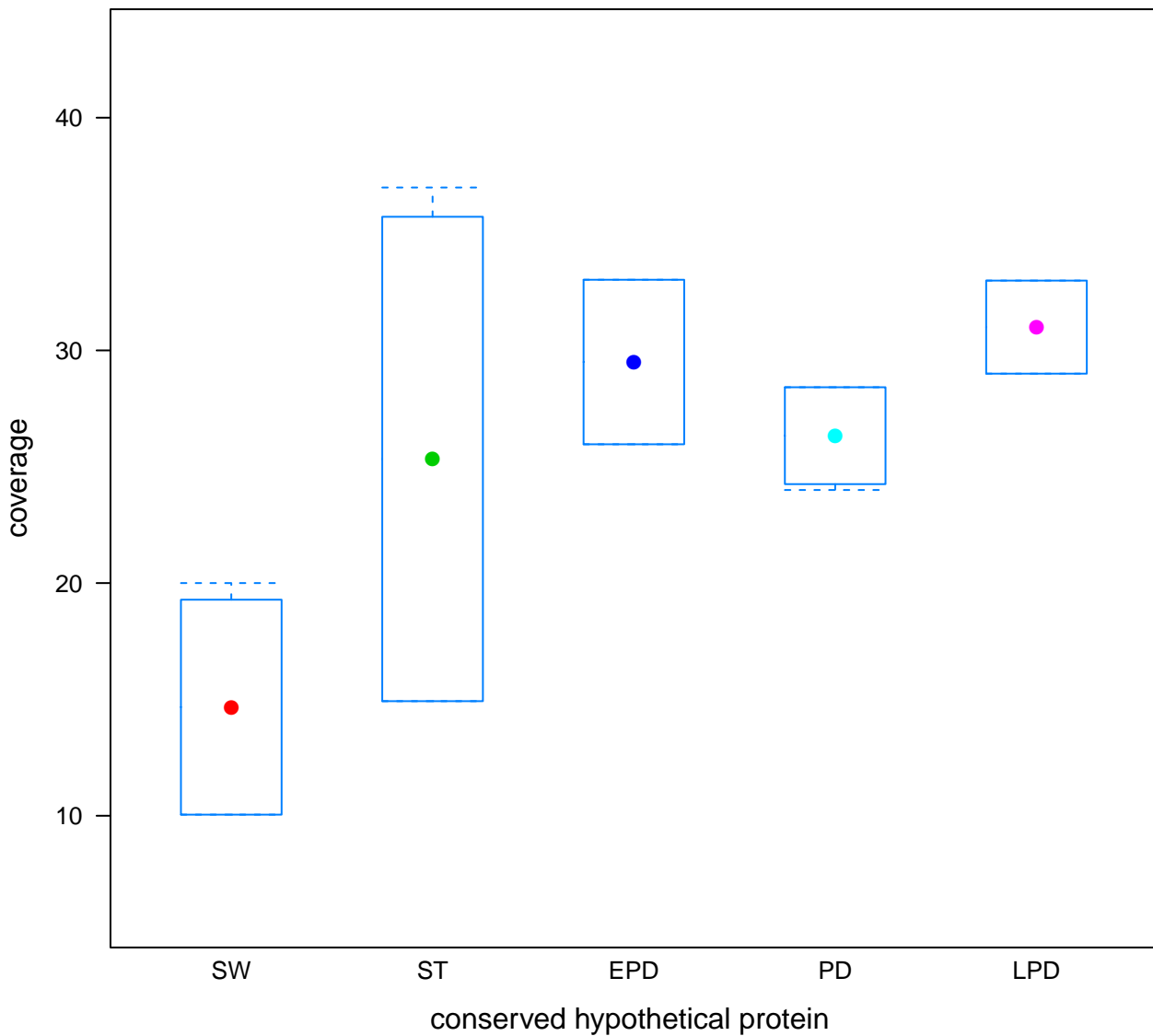

**Fold of change: 2.58**  
**baySeq likelihood: 0.887**

Supplement: Additional file 9: Figure S2 — Expression profiles of all identified CCR genes. [file 1471-2164-14-450-S9.zip › FigureS2/CCNA_00111.pdf]

# CCNA\_00113

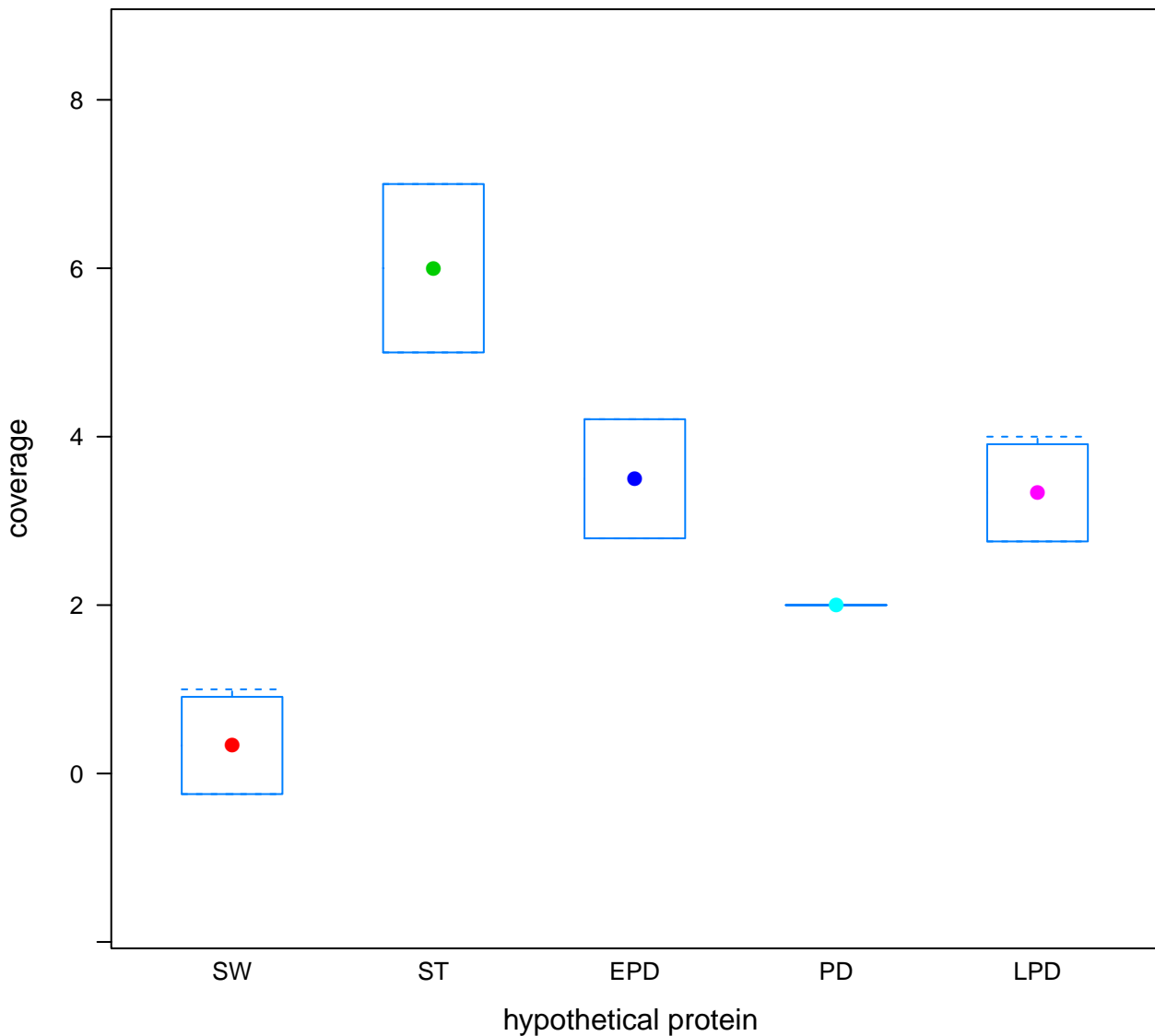

**Fold of change: 13**  
**baySeq likelihood: 0.826**

Supplement: Additional file 9: Figure S2 — Expression profiles of all identified CCR genes. [file 1471-2164-14-450-S9.zip › FigureS2/CCNA_00113.pdf]

# CCNA\_00114

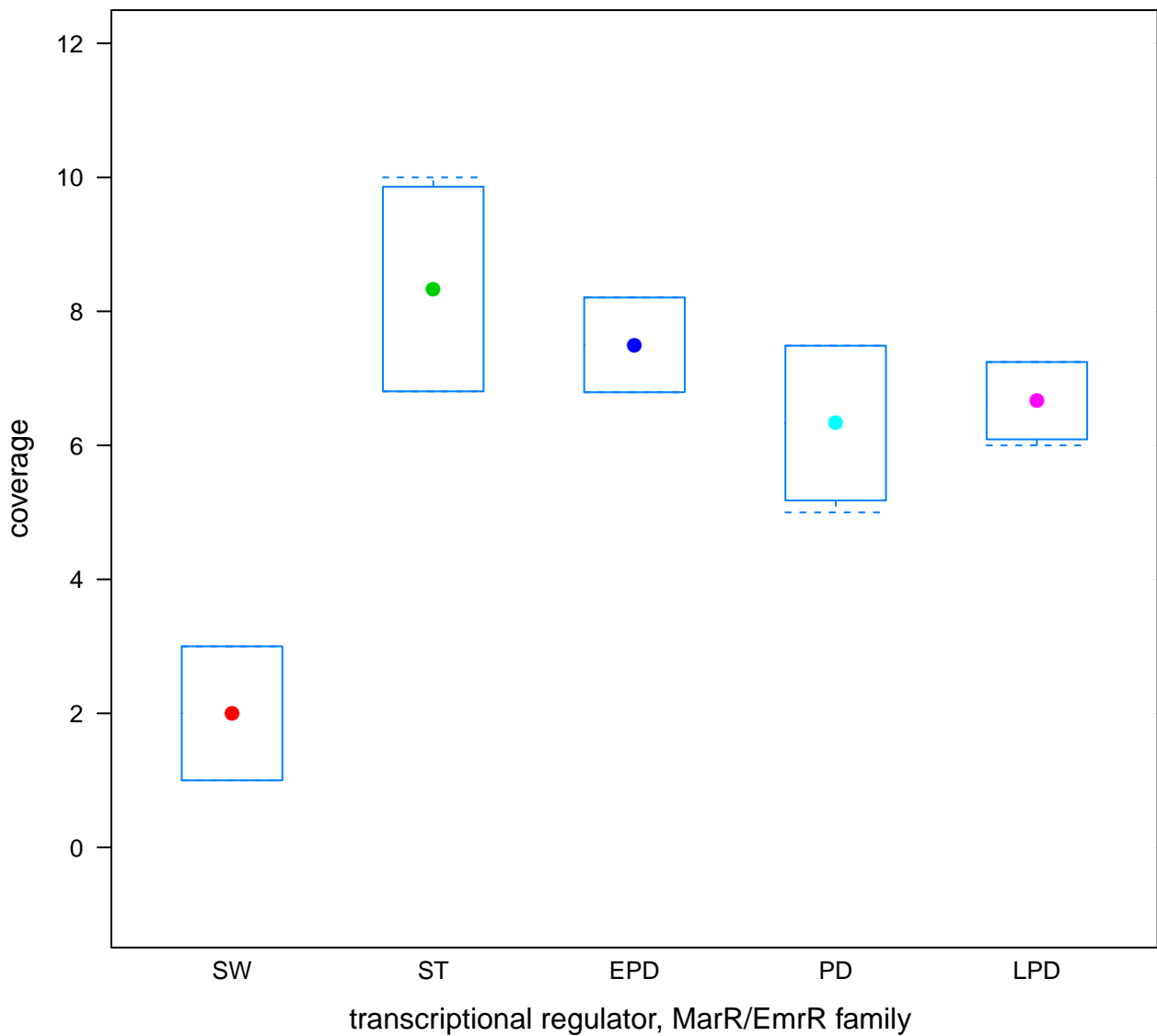

**Fold of change: 3.4**  
**baySeq likelihood: 0.919**

Supplement: Additional file 9: Figure S2 — Expression profiles of all identified CCR genes. [file 1471-2164-14-450-S9.zip › FigureS2/CCNA_00114.pdf]

# CCNA\_00115

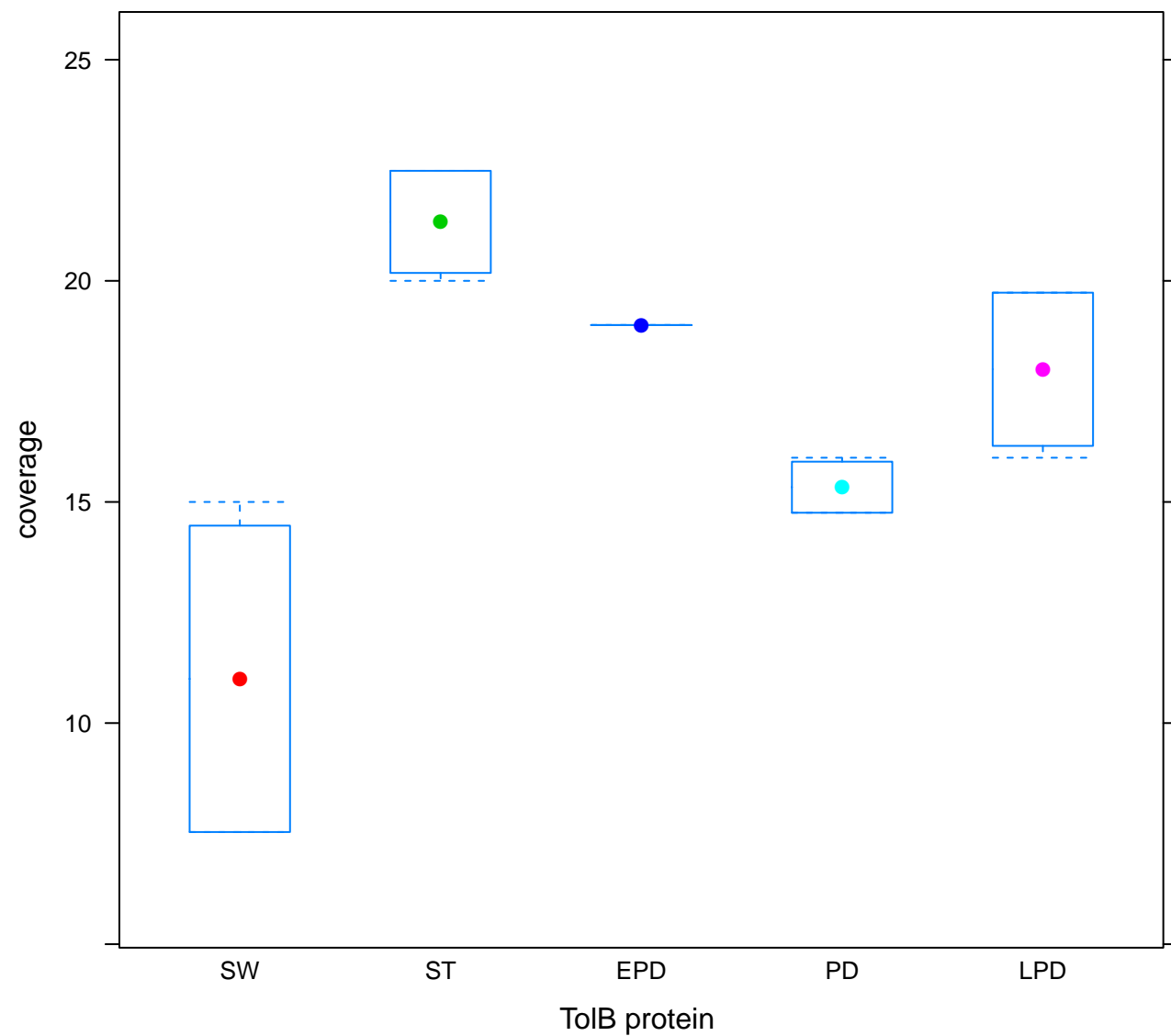

**Fold of change: 2.33**  
**baySeq likelihood: 0.419**

Supplement: Additional file 9: Figure S2 — Expression profiles of all identified CCR genes. [file 1471-2164-14-450-S9.zip › FigureS2/CCNA_00115.pdf]

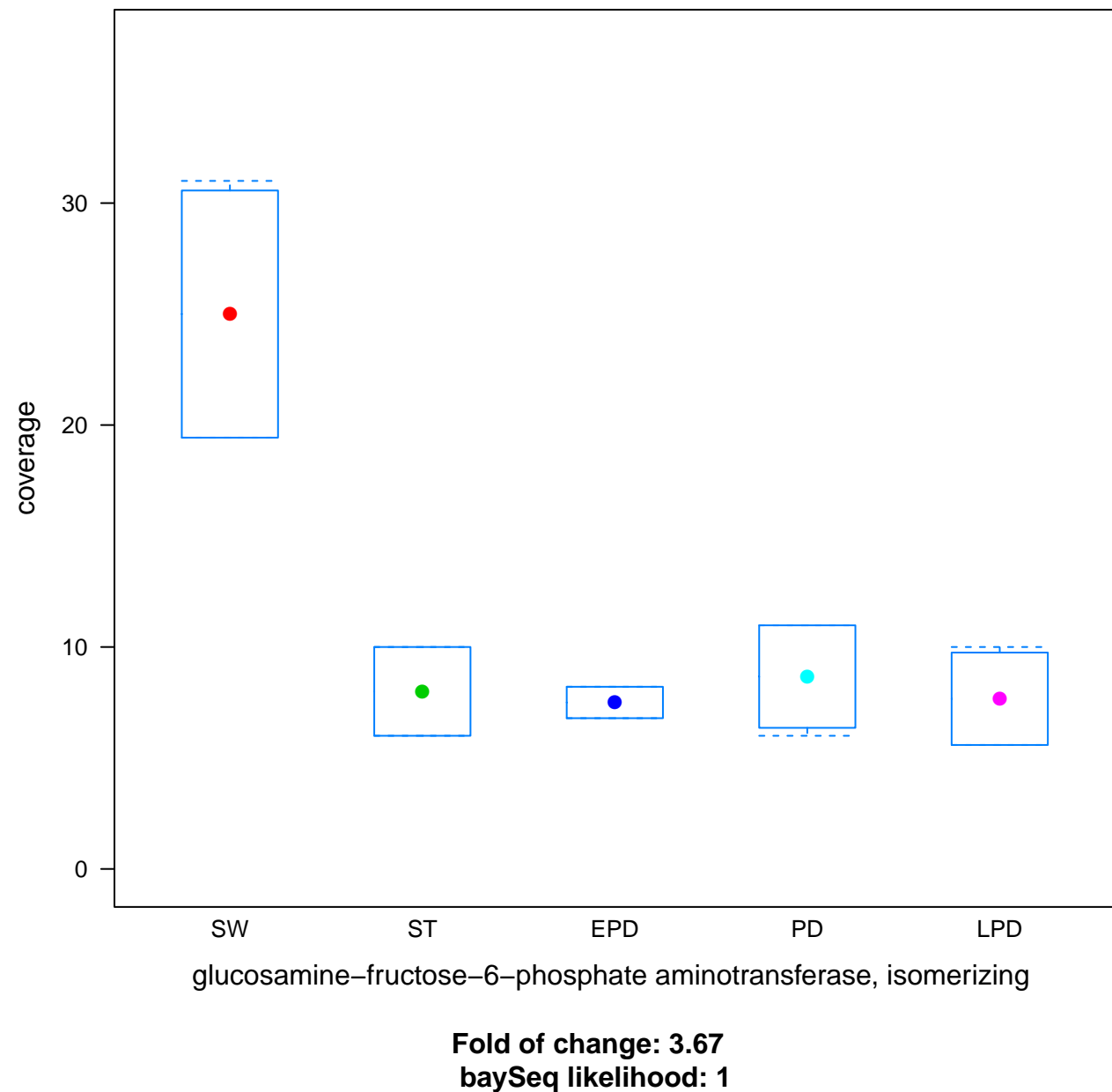

Supplement: Additional file 9: Figure S2 — Expression profiles of all identified CCR genes. [file 1471-2164-14-450-S9.zip › FigureS2/CCNA_00117.pdf]

# CCNA\_00118

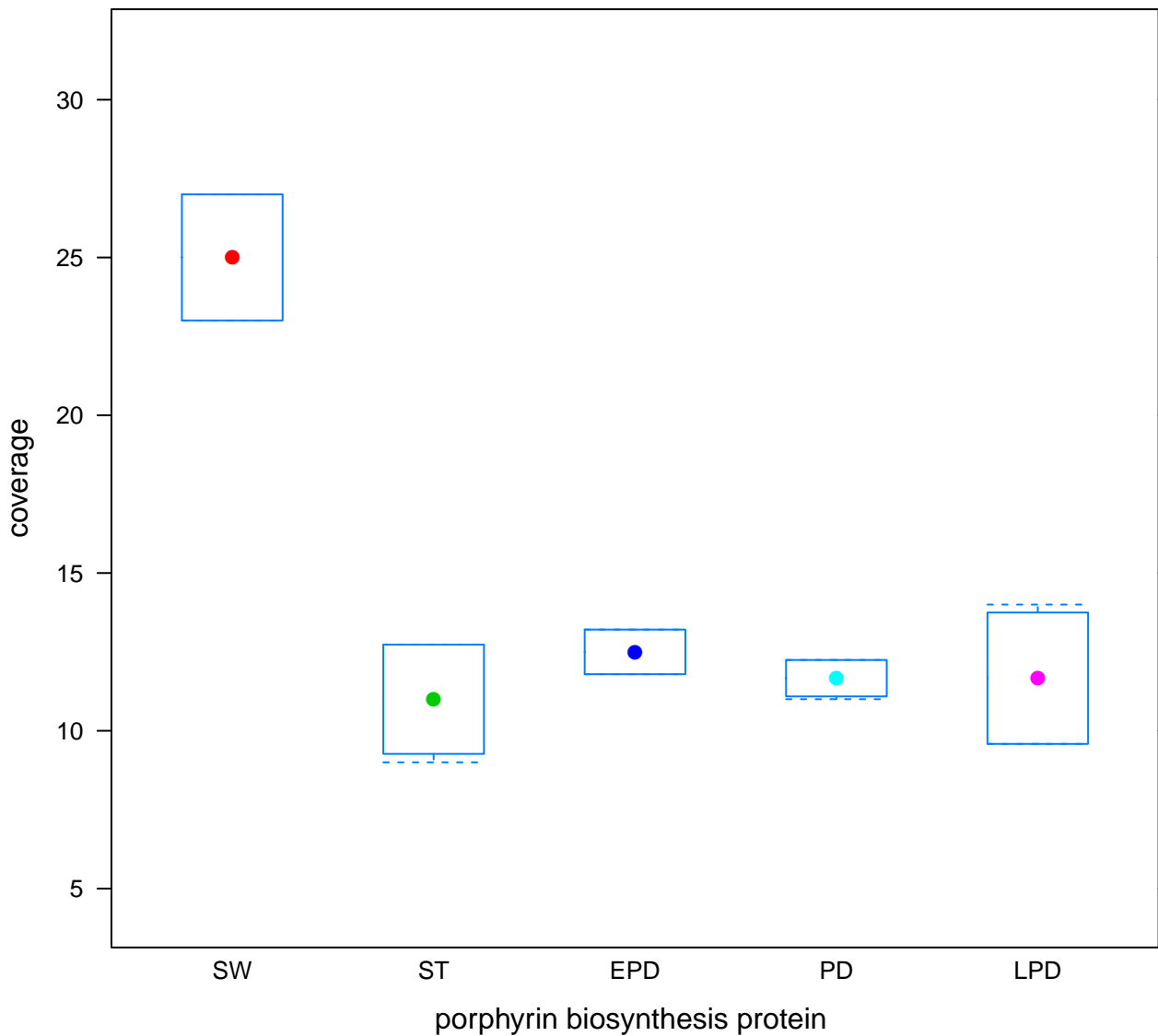

**Fold of change: 2.14**  
**baySeq likelihood: 0.999**

Supplement: Additional file 9: Figure S2 — Expression profiles of all identified CCR genes. [file 1471-2164-14-450-S9.zip › FigureS2/CCNA_00118.pdf]

# CCNA\_00119

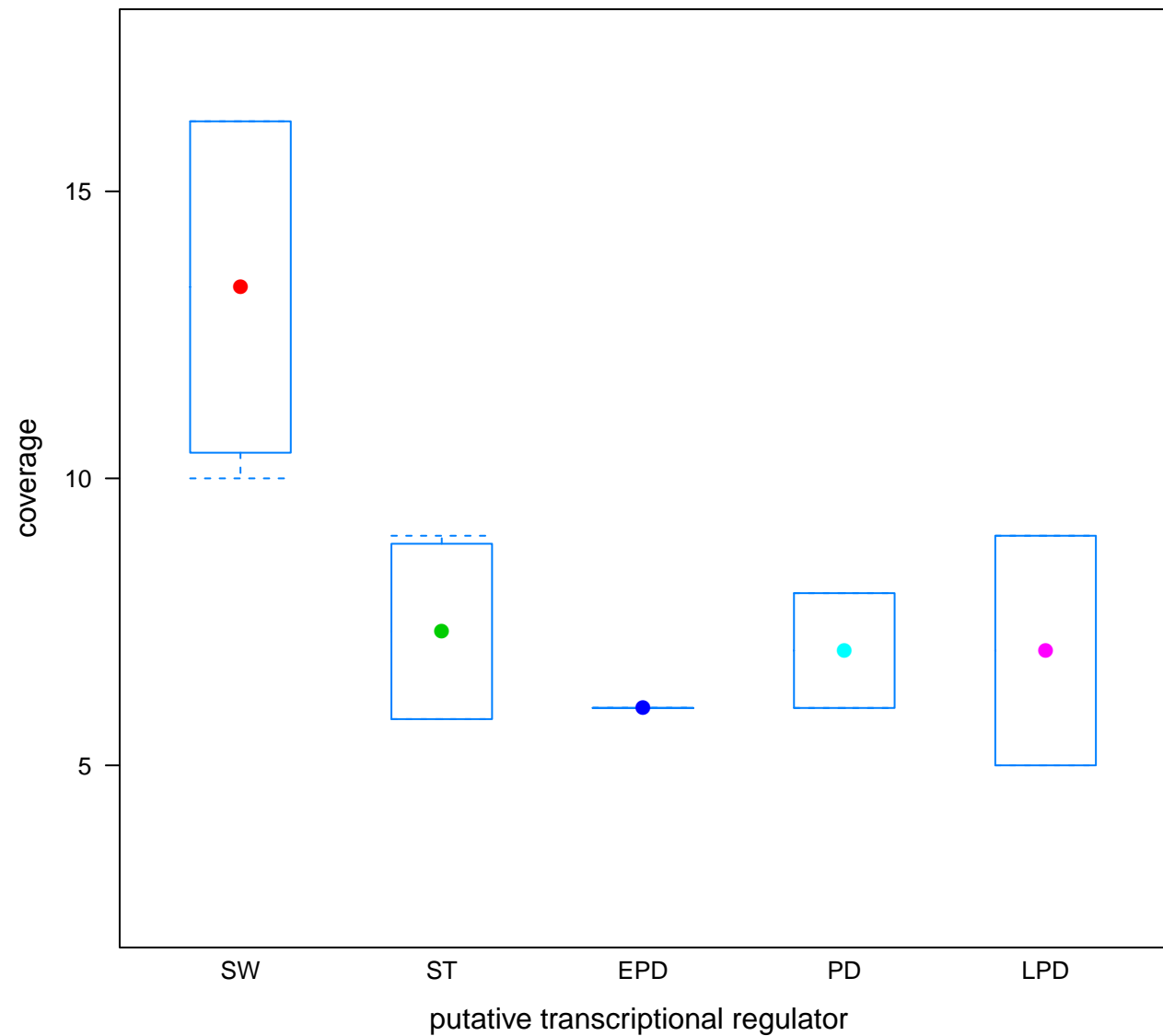

**Fold of change: 2.08**  
**baySeq likelihood: 0.882**

Supplement: Additional file 9: Figure S2 — Expression profiles of all identified CCR genes. [file 1471-2164-14-450-S9.zip › FigureS2/CCNA_00119.pdf]

# CCNA\_00122

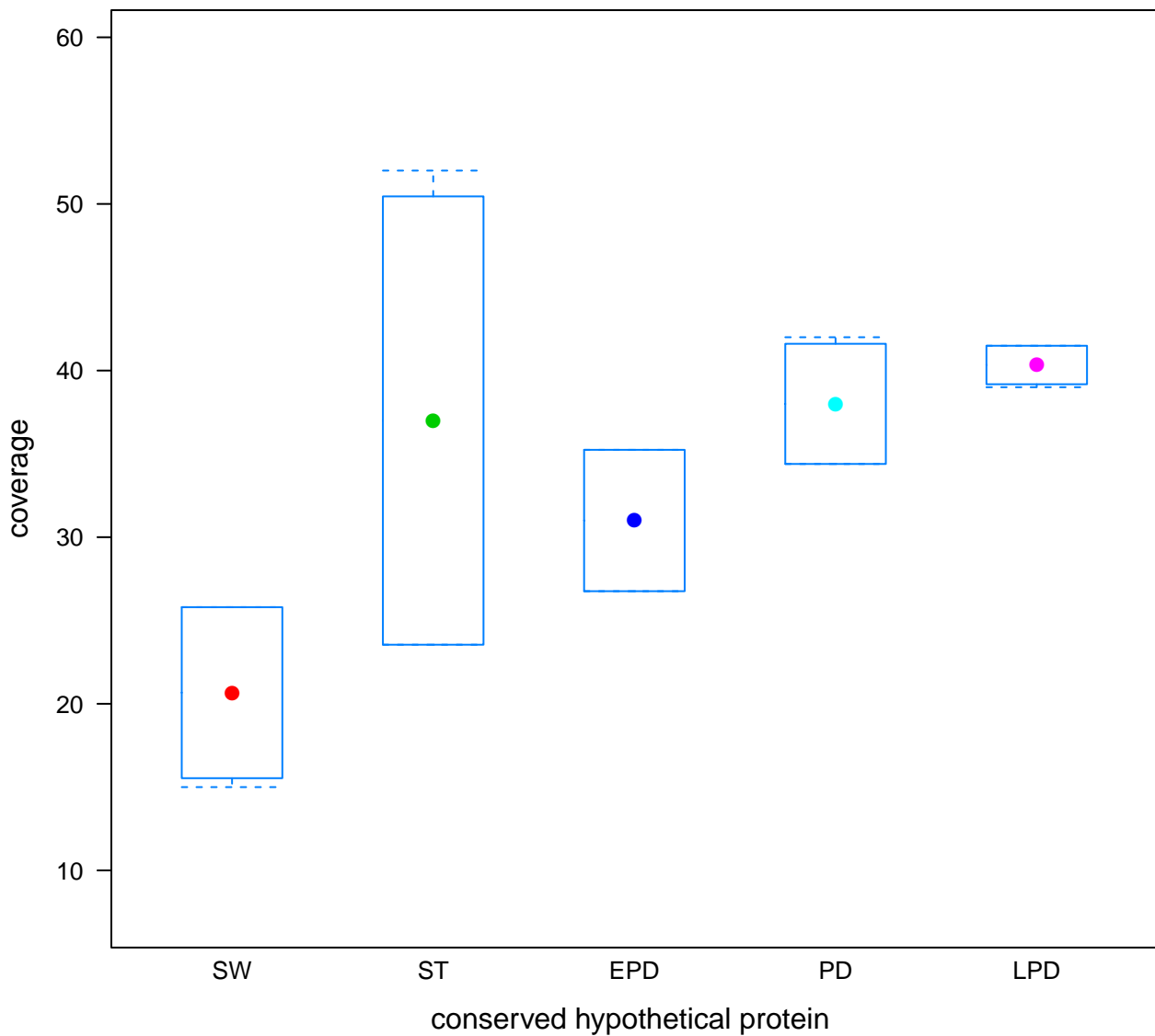

**Fold of change: 2.18**  
**baySeq likelihood: 0.876**

Supplement: Additional file 9: Figure S2 — Expression profiles of all identified CCR genes. [file 1471-2164-14-450-S9.zip › FigureS2/CCNA_00122.pdf]

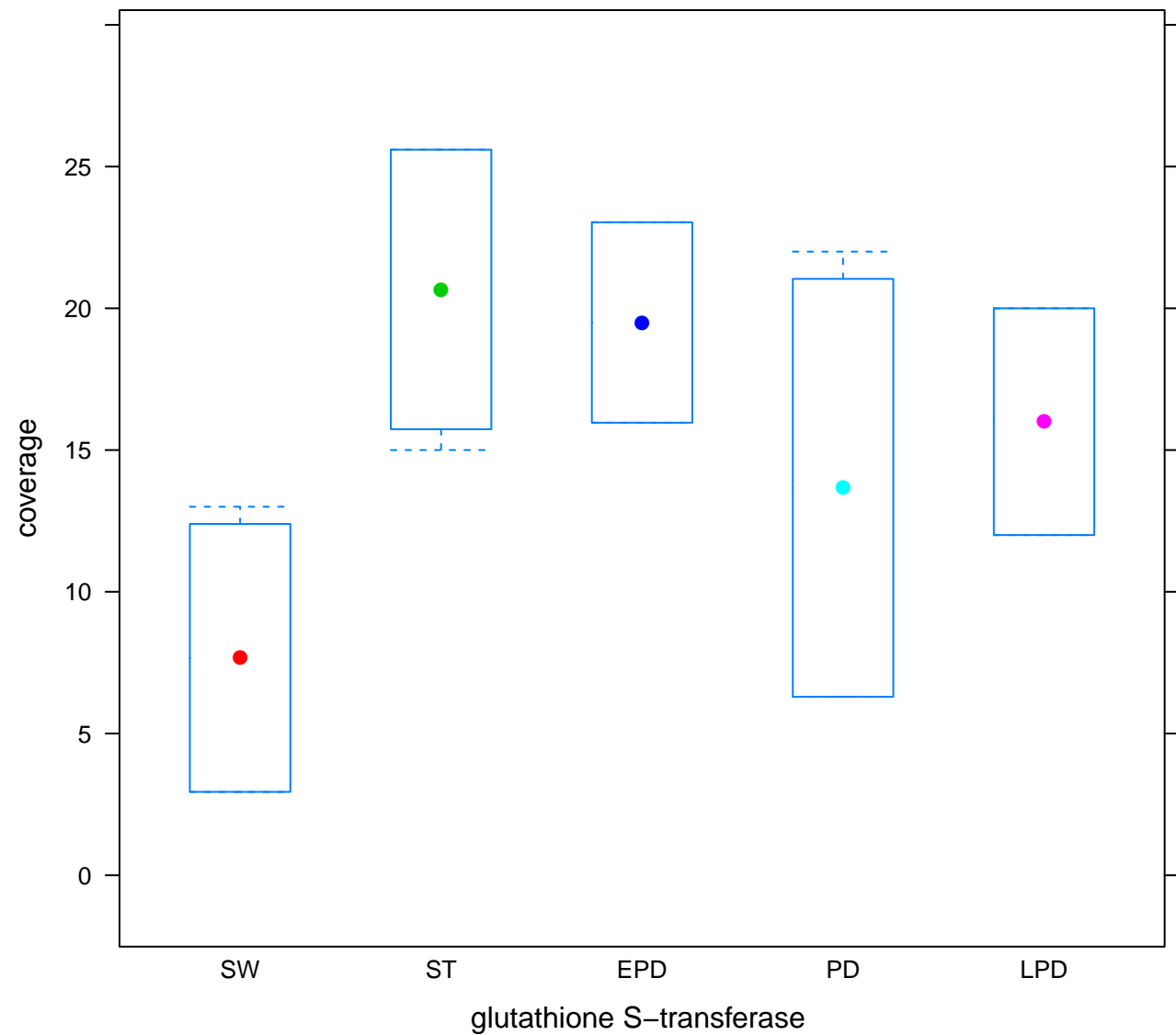

**Fold of change: 3.9**  
**baySeq likelihood: 0.698**

Supplement: Additional file 9: Figure S2 — Expression profiles of all identified CCR genes. [file 1471-2164-14-450-S9.zip › FigureS2/CCNA_00126.pdf]

# CCNA\_00128

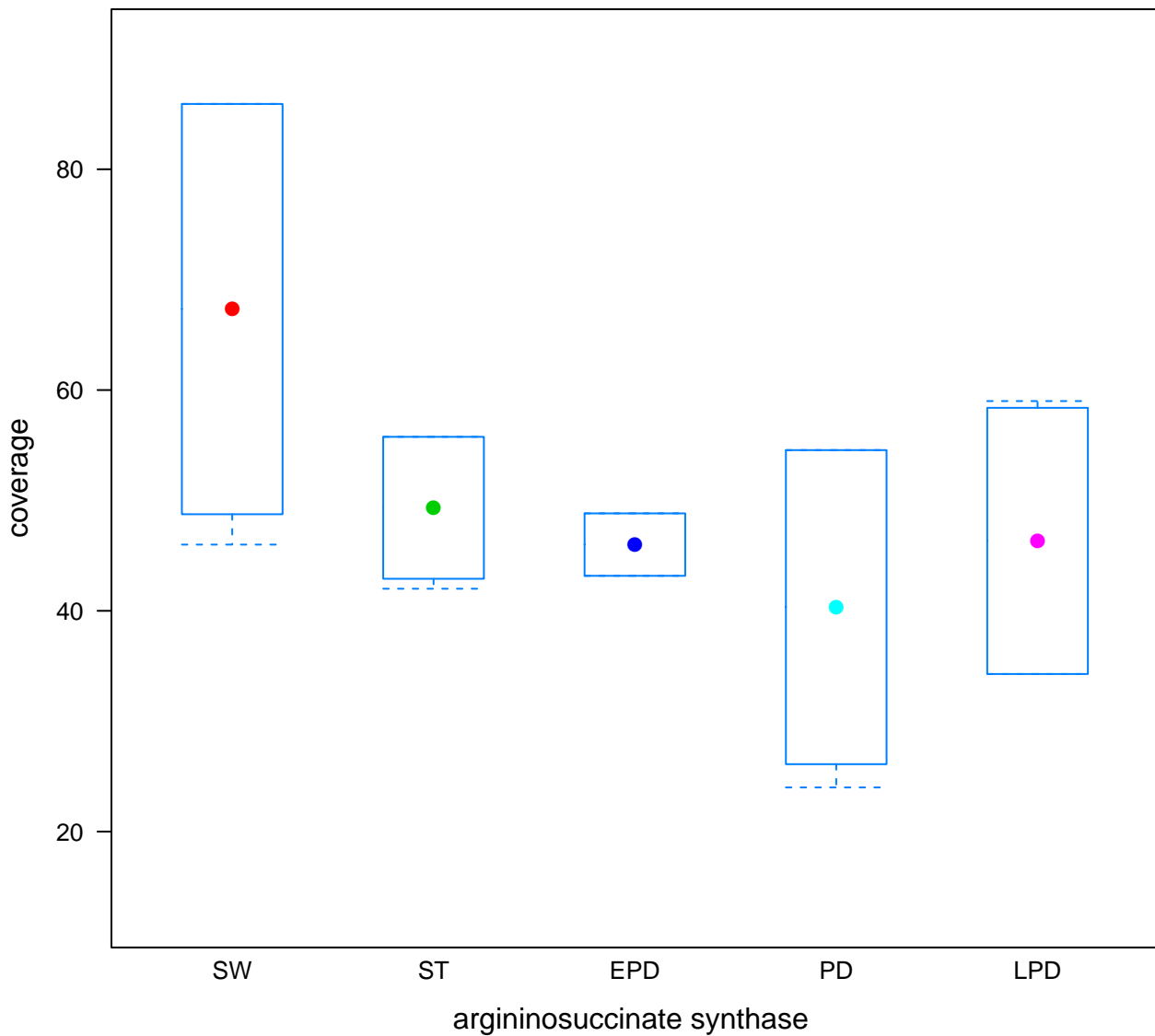

**Fold of change: 1.93**  
**baySeq likelihood: 0.461**

Supplement: Additional file 9: Figure S2 — Expression profiles of all identified CCR genes. [file 1471-2164-14-450-S9.zip › FigureS2/CCNA_00128.pdf]

# CCNA\_00129

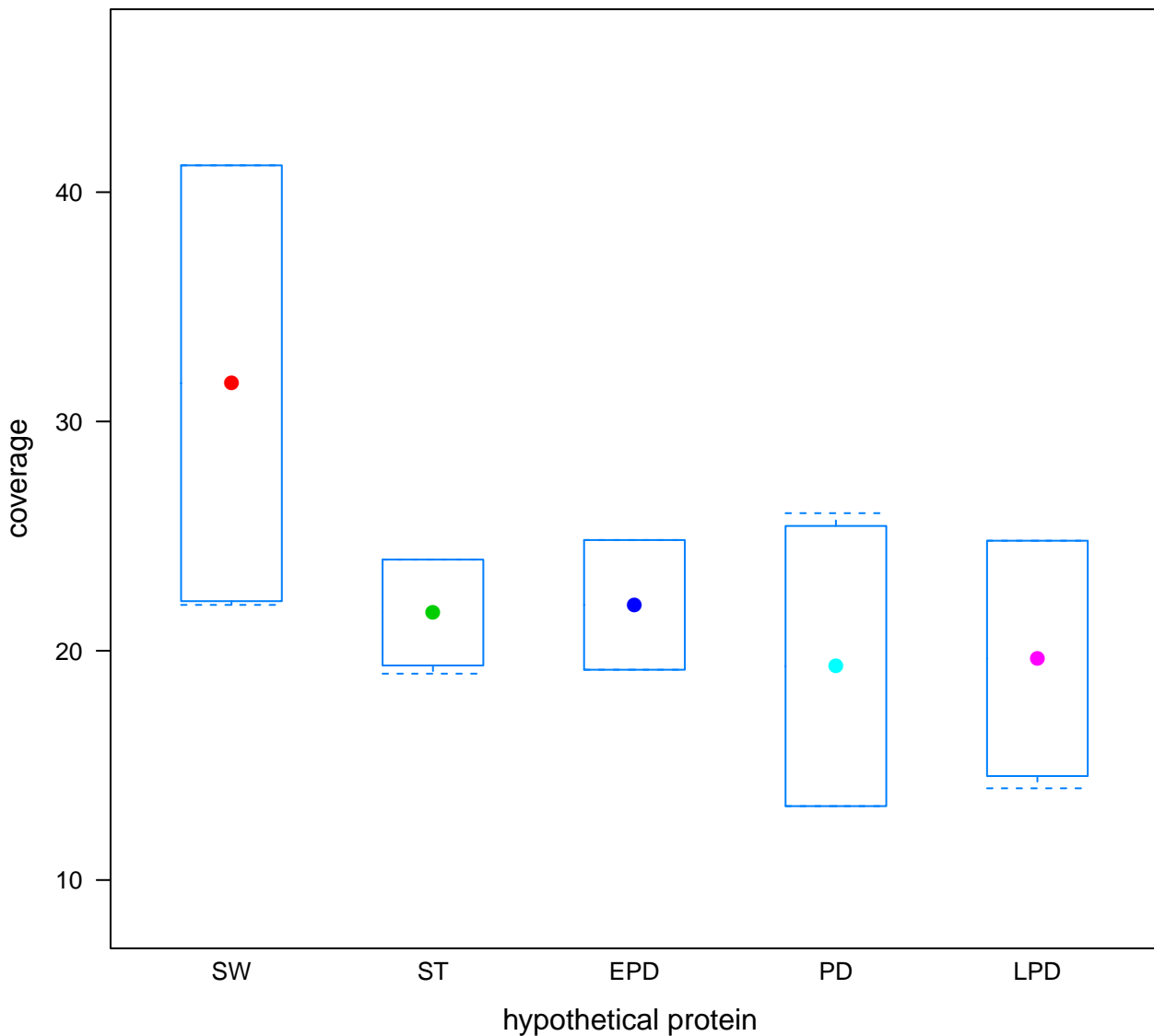

**Fold of change: 1.89**  
**baySeq likelihood: 0.872**

Supplement: Additional file 9: Figure S2 — Expression profiles of all identified CCR genes. [file 1471-2164-14-450-S9.zip › FigureS2/CCNA_00129.pdf]

# CCNA\_00130

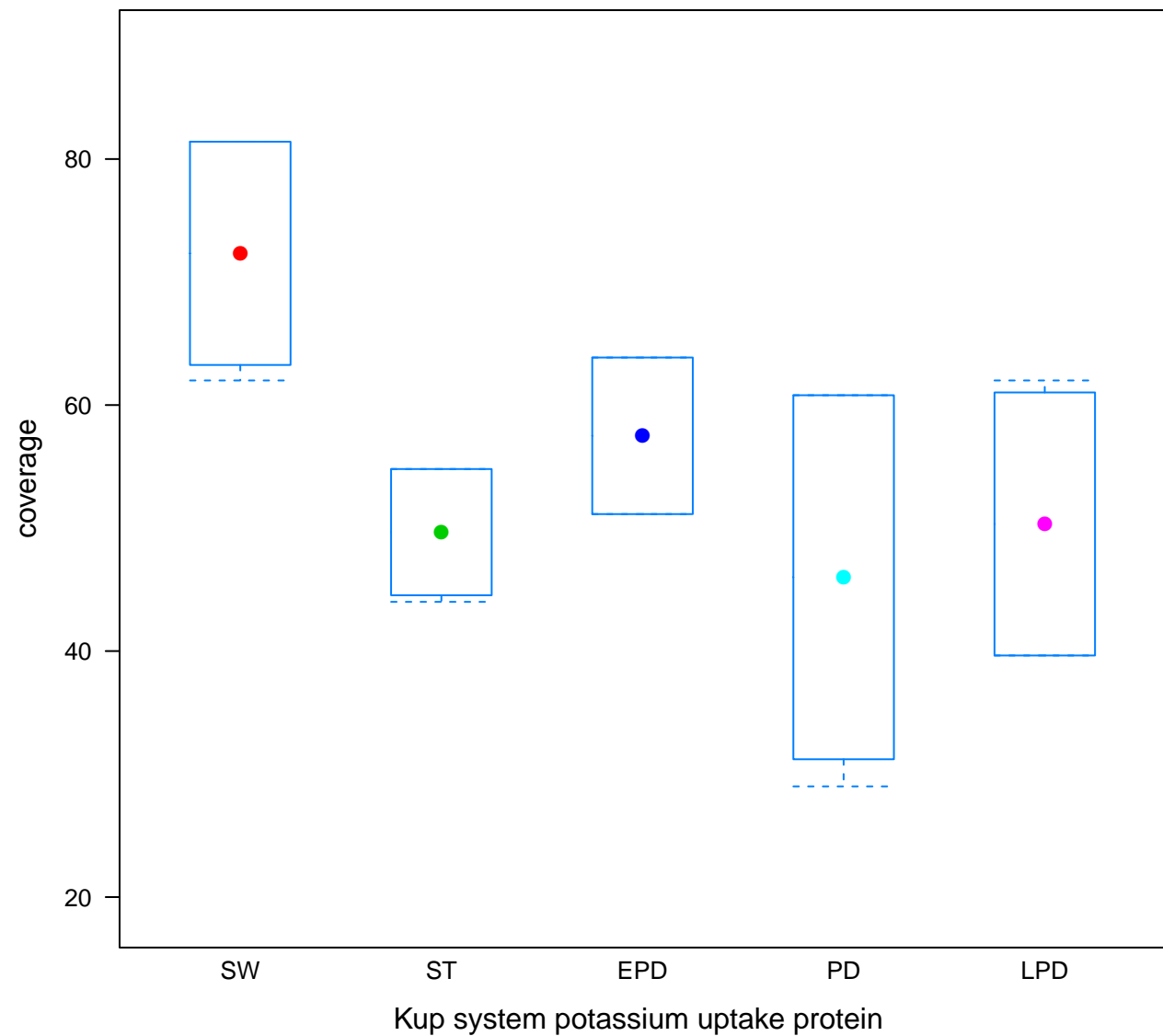

**Fold of change: 1.68**  
**baySeq likelihood: 0.702**

Supplement: Additional file 9: Figure S2 — Expression profiles of all identified CCR genes. [file 1471-2164-14-450-S9.zip › FigureS2/CCNA_00130.pdf]

# CCNA\_00136

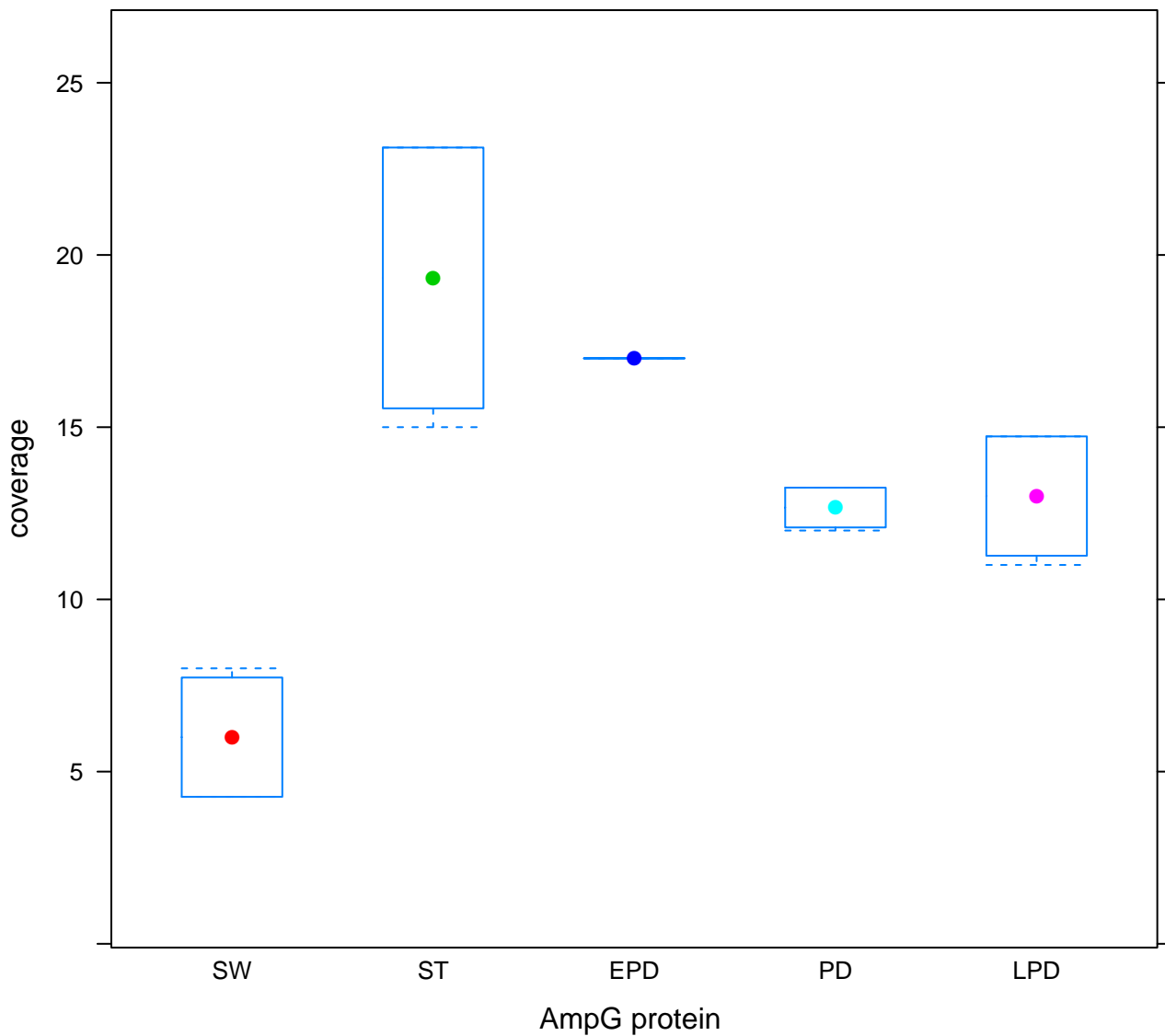

**Fold of change: 3.6**  
**baySeq likelihood: 0.932**

Supplement: Additional file 9: Figure S2 — Expression profiles of all identified CCR genes. [file 1471-2164-14-450-S9.zip › FigureS2/CCNA_00136.pdf]

# shkA;CCNA\_00137

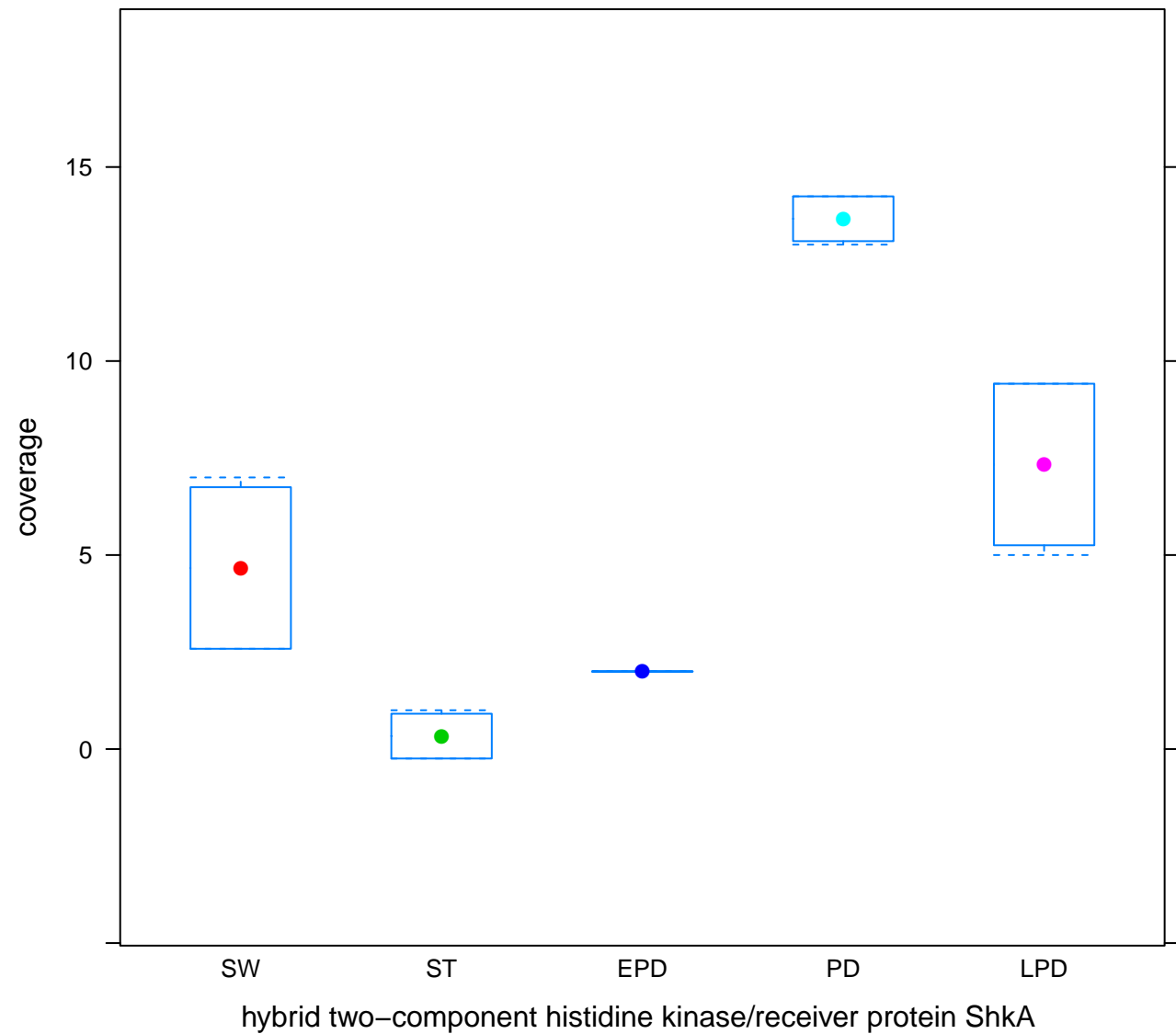

**Fold of change: 27.33**  
**baySeq likelihood: 0.854**

Supplement: Additional file 9: Figure S2 — Expression profiles of all identified CCR genes. [file 1471-2164-14-450-S9.zip › FigureS2/CCNA_00137.pdf]

# CCNA\_00138

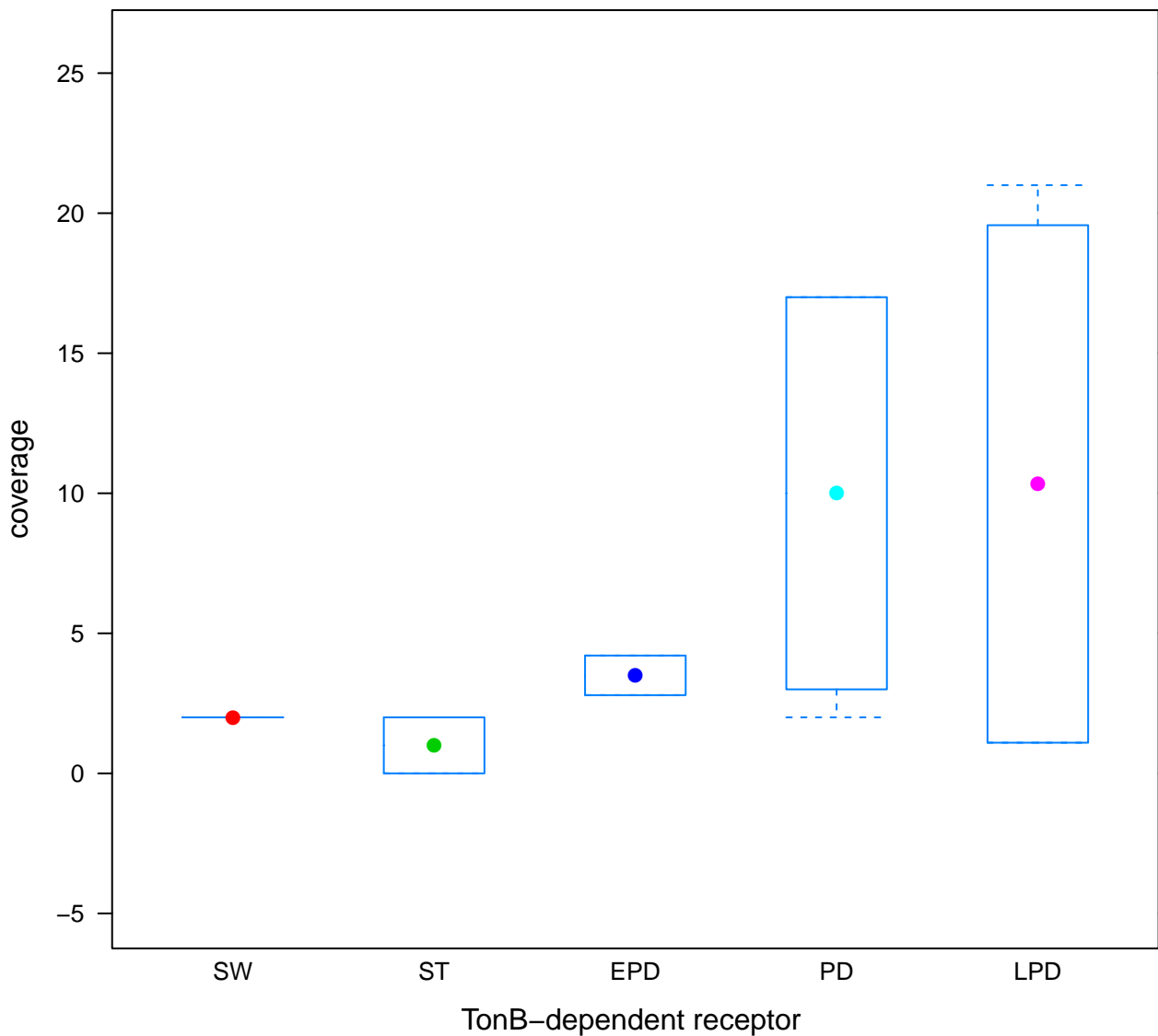

**Fold of change: 20.67**  
**baySeq likelihood: 0.704**

Supplement: Additional file 9: Figure S2 — Expression profiles of all identified CCR genes. [file 1471-2164-14-450-S9.zip › FigureS2/CCNA_00138.pdf]

# CCNA\_00140

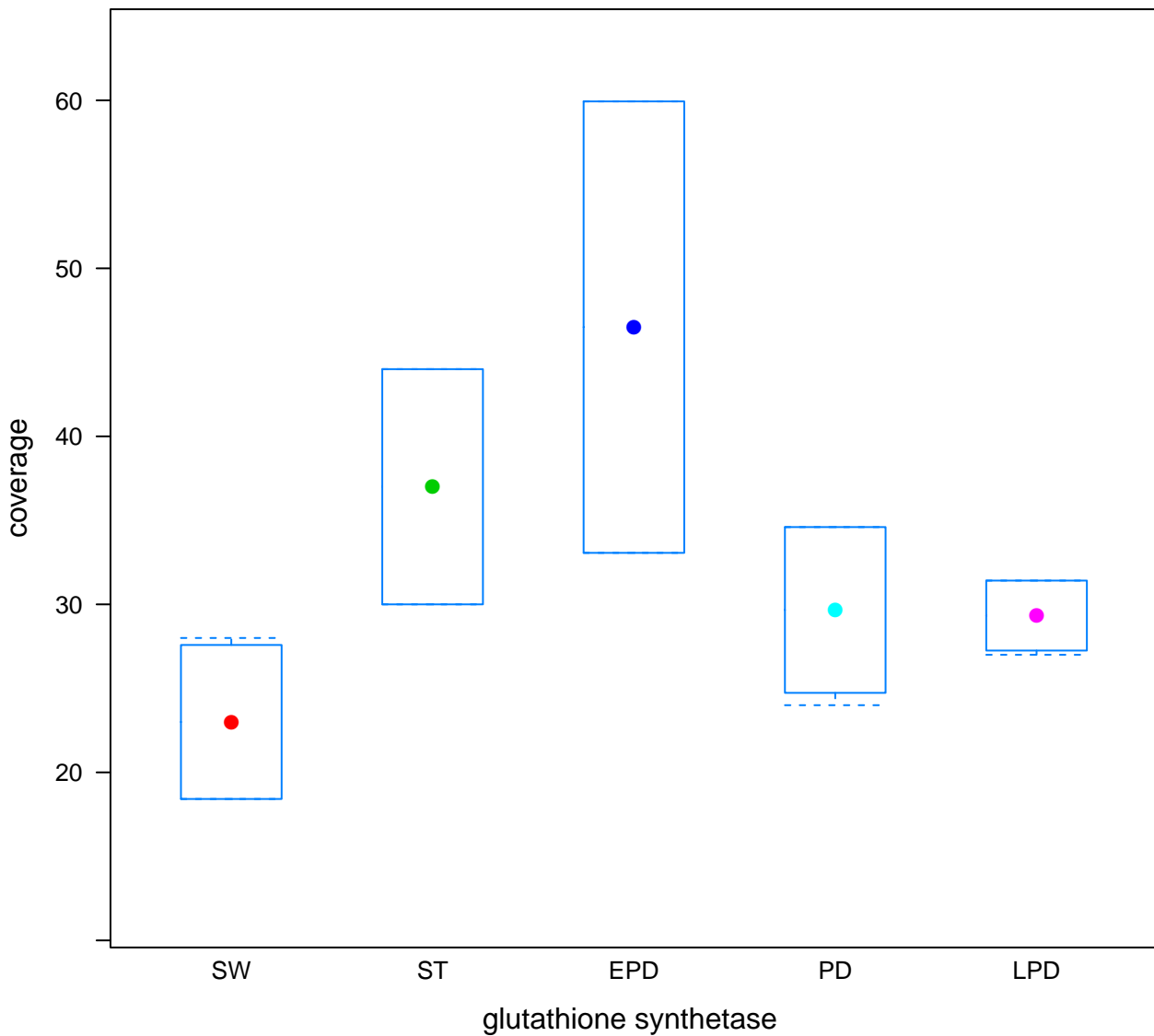

**Fold of change: 2.27**  
**baySeq likelihood: 0.511**

Supplement: Additional file 9: Figure S2 — Expression profiles of all identified CCR genes. [file 1471-2164-14-450-S9.zip › FigureS2/CCNA_00140.pdf]

# CCNA\_00150

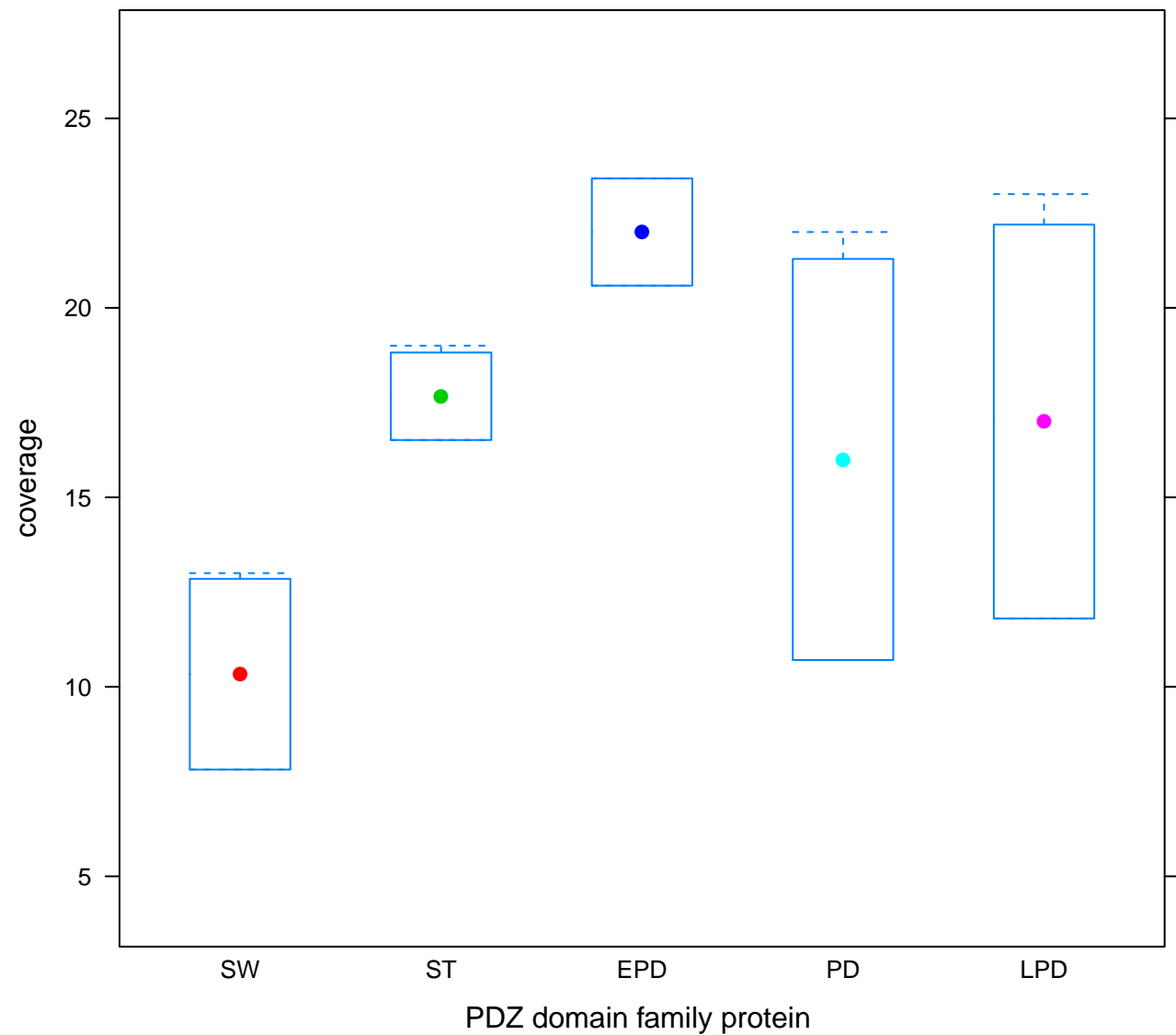

**Fold of change: 1.91**  
**baySeq likelihood: 0.498**

Supplement: Additional file 9: Figure S2 — Expression profiles of all identified CCR genes. [file 1471-2164-14-450-S9.zip › FigureS2/CCNA_00150.pdf]

# CCNA\_00151

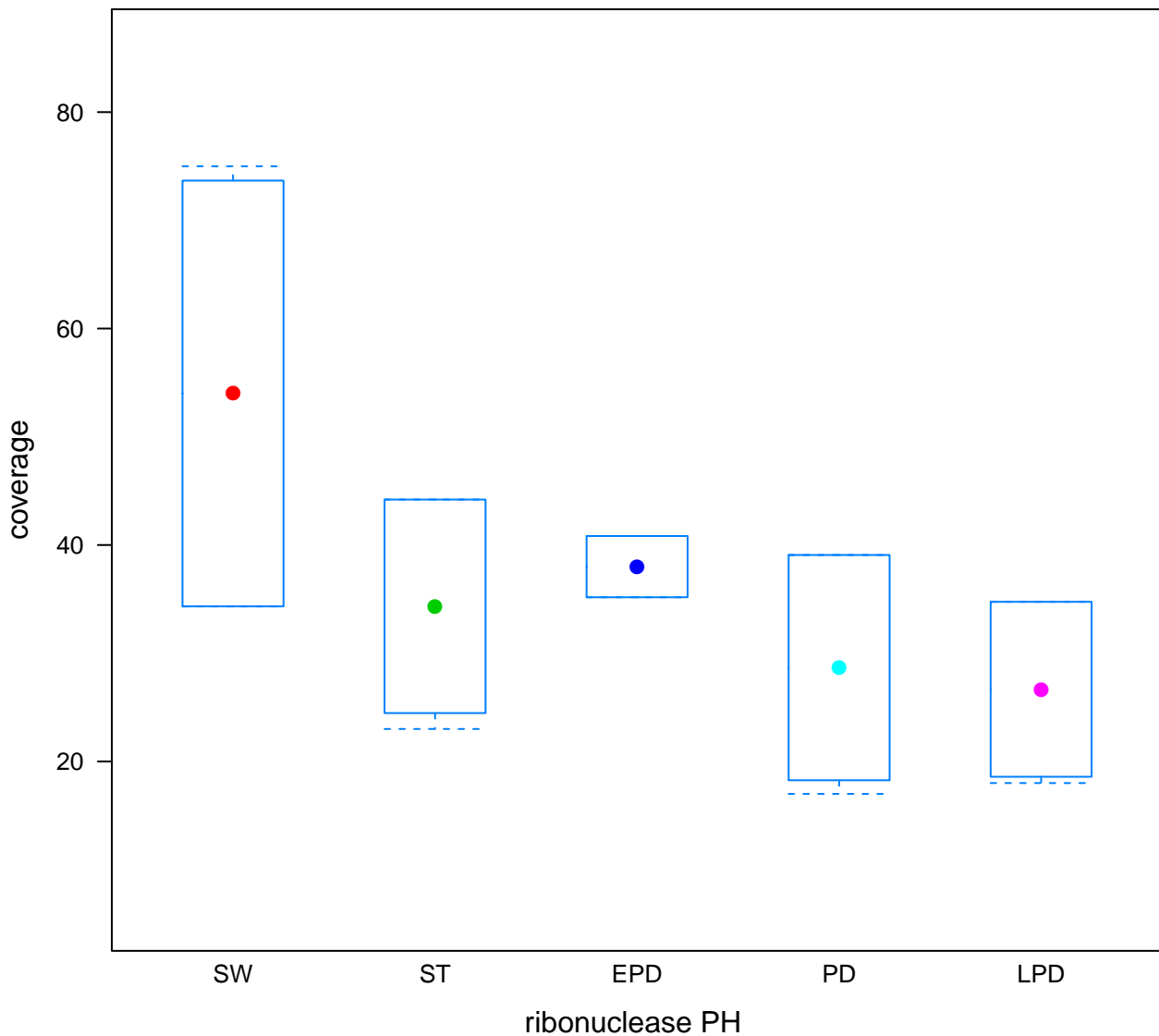

**Fold of change: 2.36**  
**baySeq likelihood: 0.527**

Supplement: Additional file 9: Figure S2 — Expression profiles of all identified CCR genes. [file 1471-2164-14-450-S9.zip › FigureS2/CCNA_00151.pdf]

# grpE;CCNA\_00153

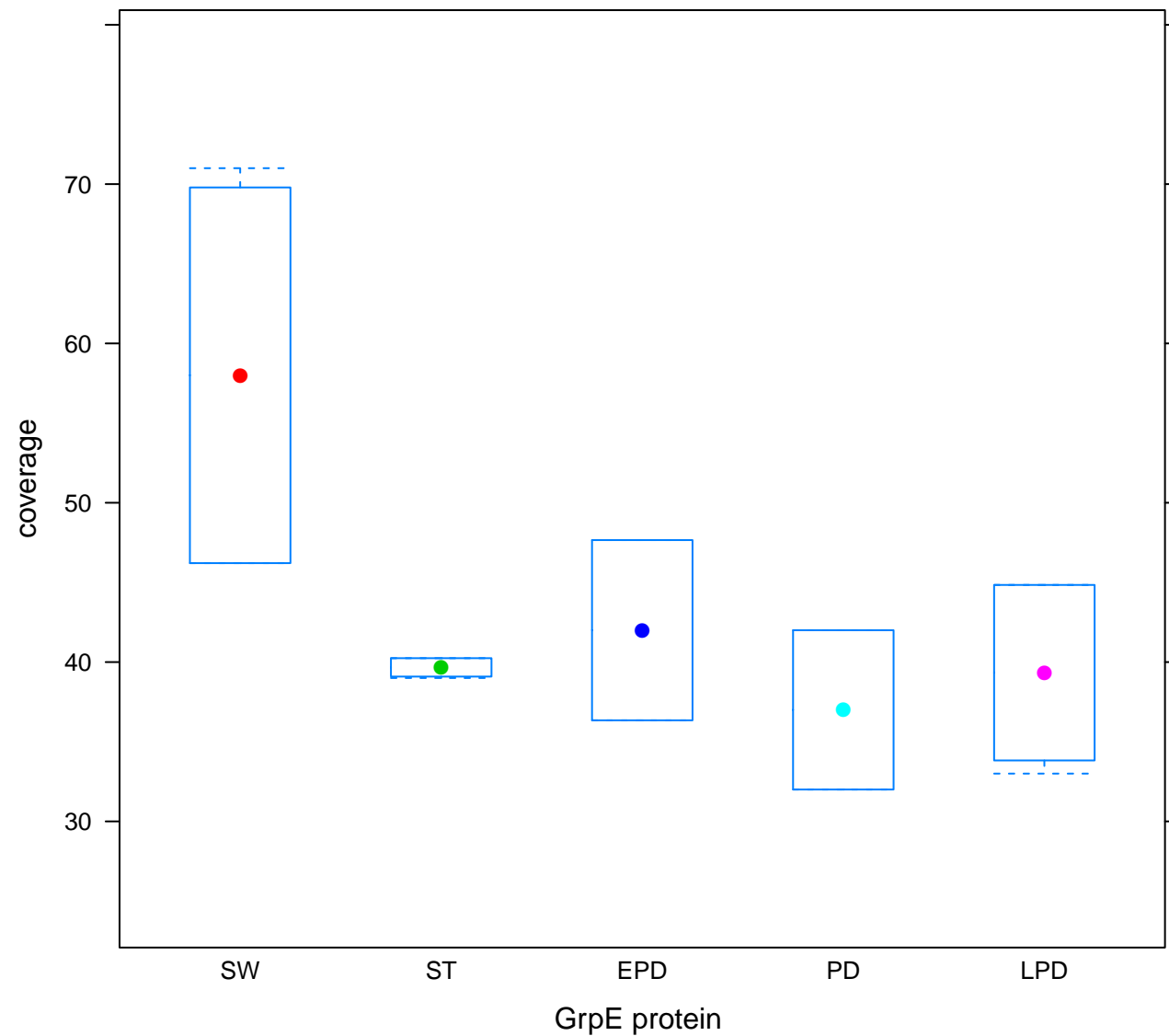

**Fold of change: 1.61**  
**baySeq likelihood: 0.94**

Supplement: Additional file 9: Figure S2 — Expression profiles of all identified CCR genes. [file 1471-2164-14-450-S9.zip › FigureS2/CCNA_00153.pdf]

# CCNA\_00155

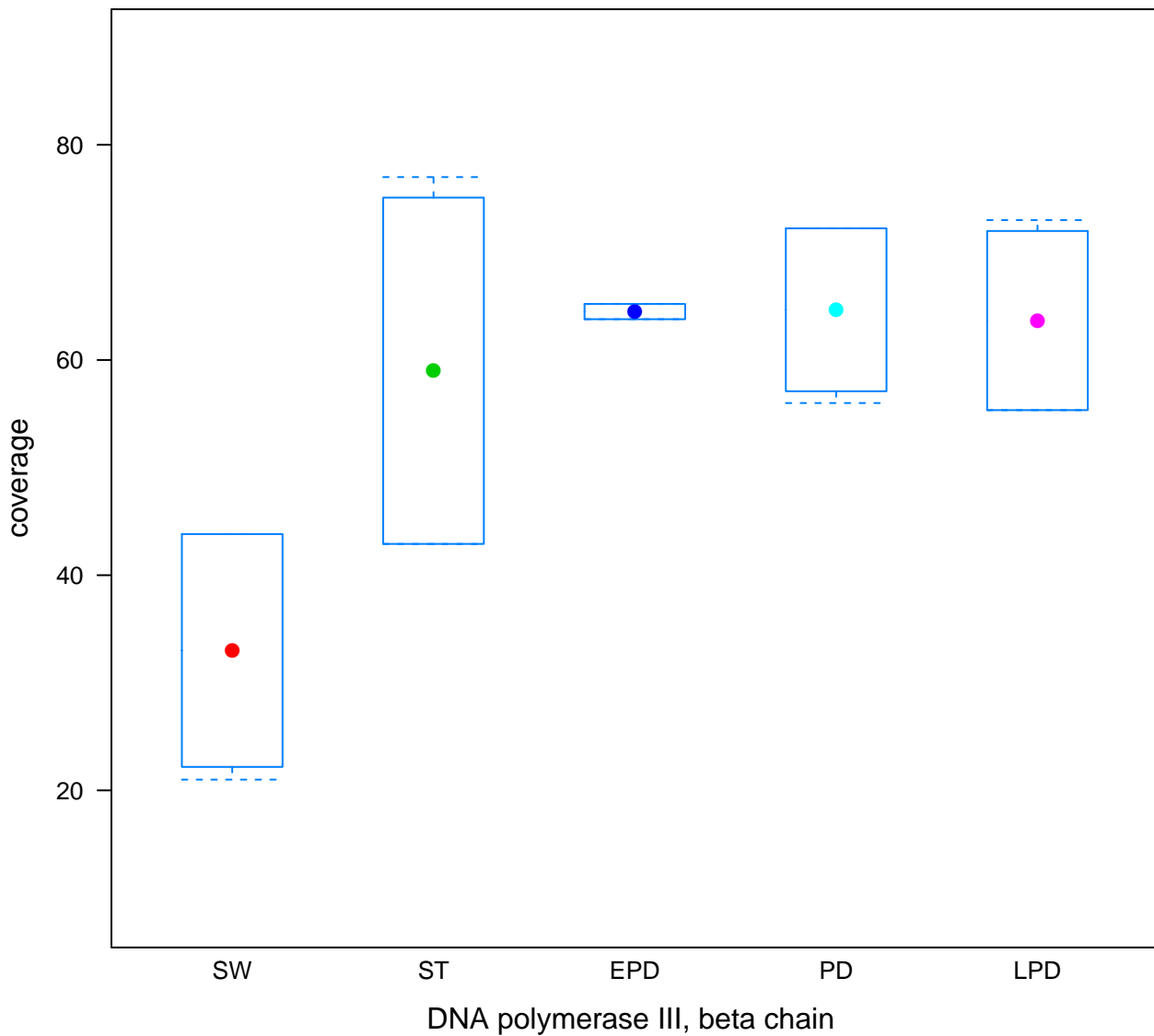

**Fold of change: 2.27**  
**baySeq likelihood: 0.927**

Supplement: Additional file 9: Figure S2 — Expression profiles of all identified CCR genes. [file 1471-2164-14-450-S9.zip › FigureS2/CCNA_00155.pdf]

# CCNA\_00157

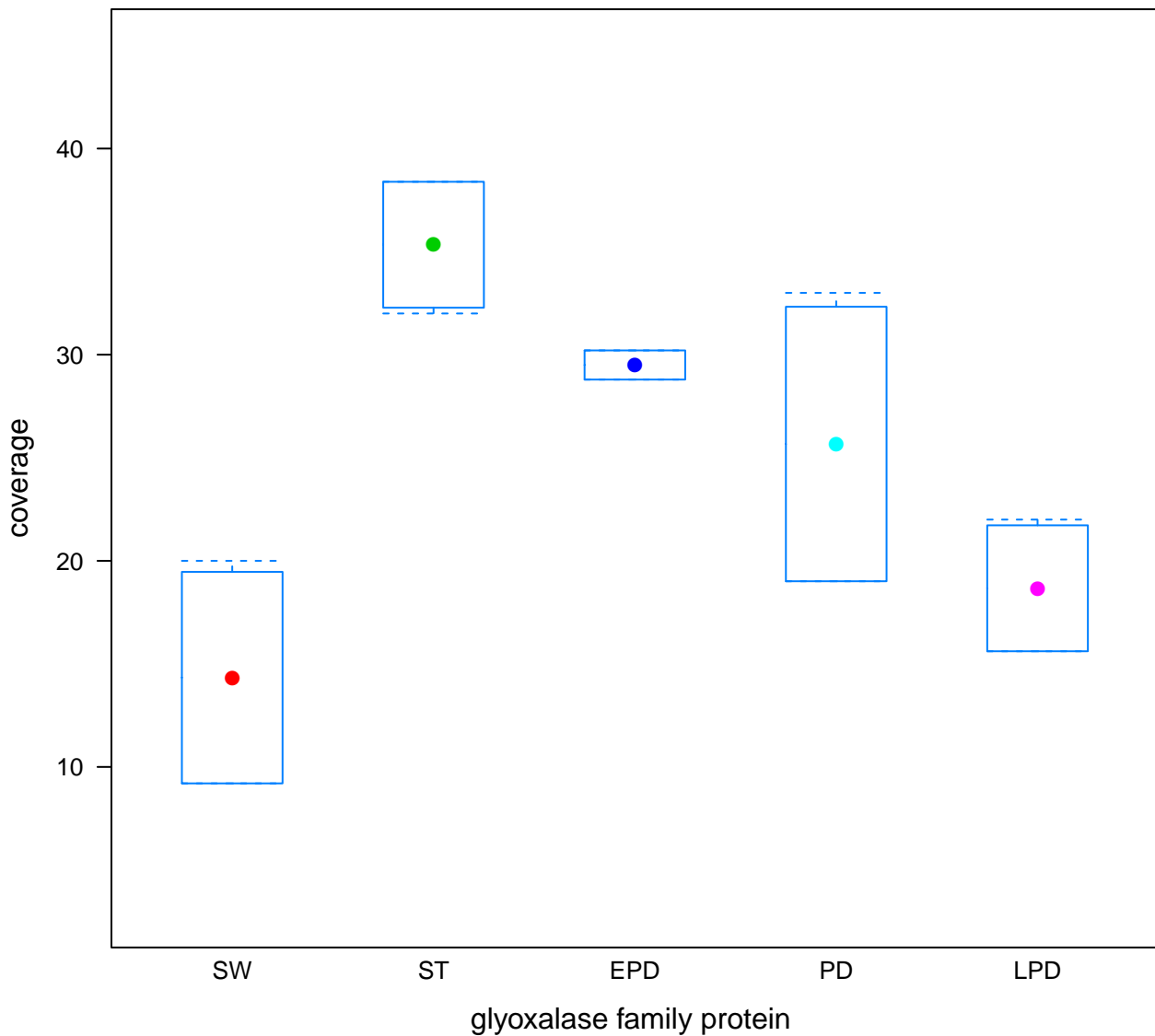

**Fold of change: 2.96**  
**baySeq likelihood: 0.434**

Supplement: Additional file 9: Figure S2 — Expression profiles of all identified CCR genes. [file 1471-2164-14-450-S9.zip › FigureS2/CCNA_00157.pdf]

# gyrB;CCNA\_00159

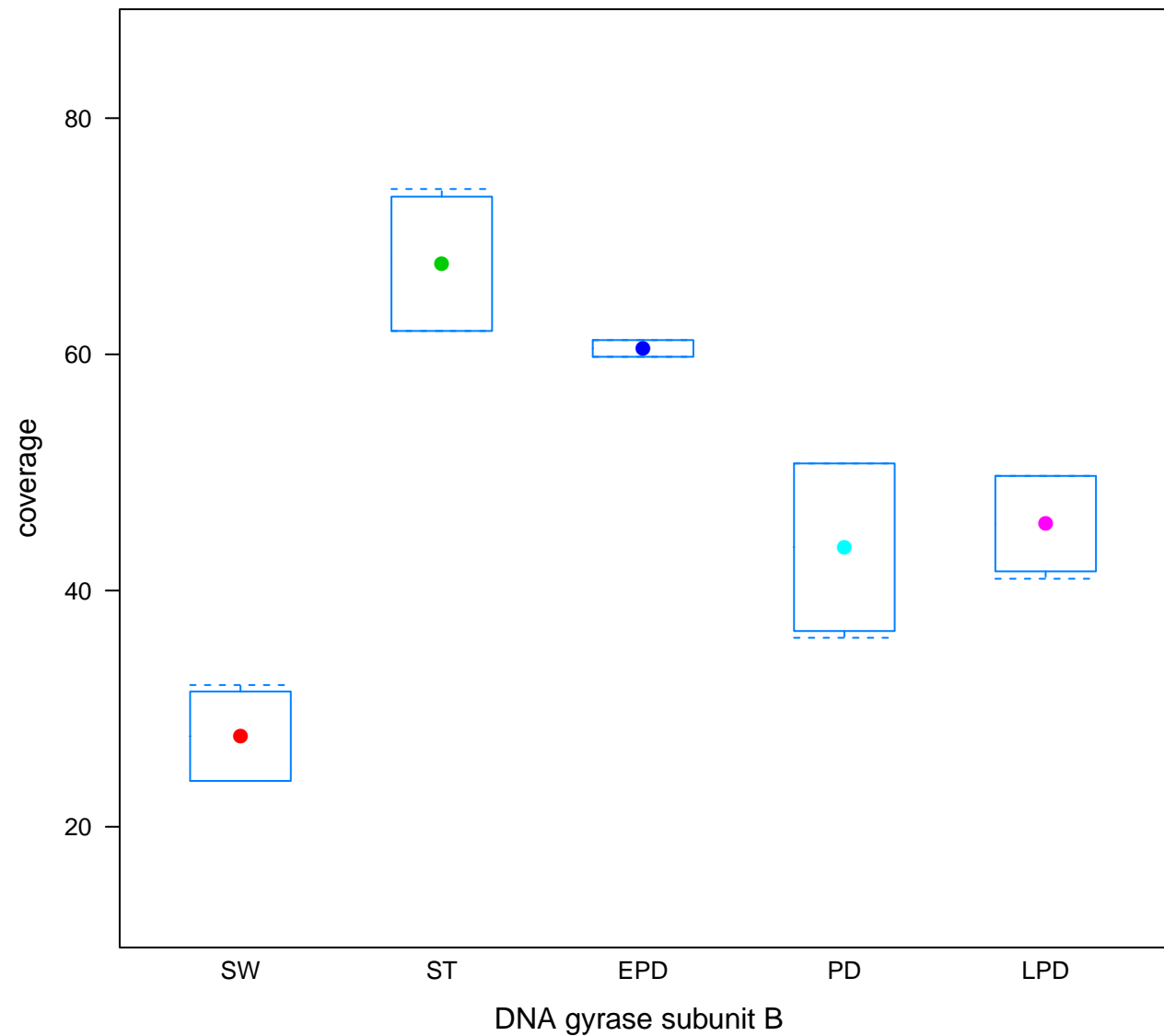

**Fold of change: 2.4**  
**baySeq likelihood: 0.567**

Supplement: Additional file 9: Figure S2 — Expression profiles of all identified CCR genes. [file 1471-2164-14-450-S9.zip › FigureS2/CCNA_00159.pdf]

# CCNA\_00161

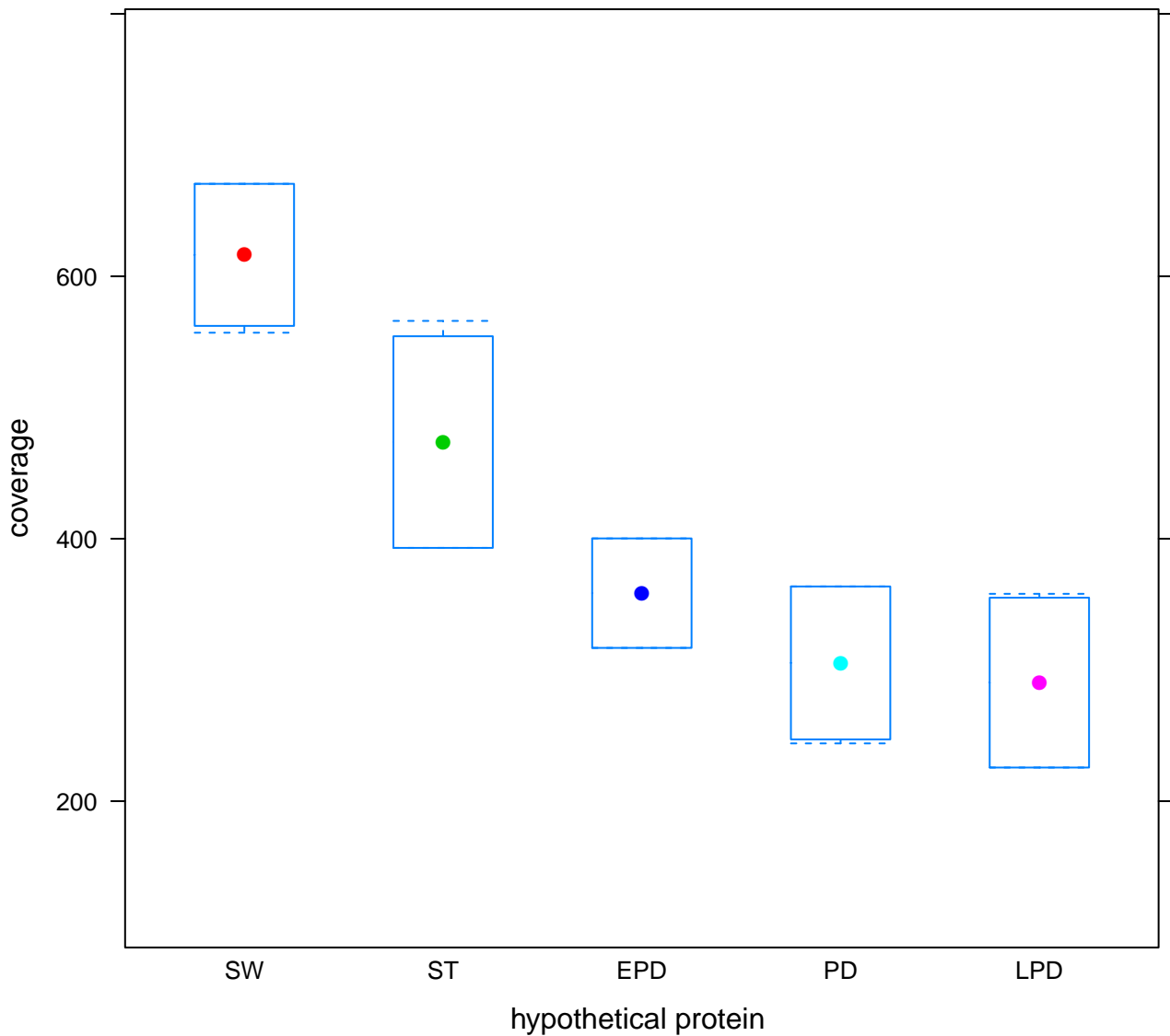

**Fold of change: 2.23**  
**baySeq likelihood: 0.83**

Supplement: Additional file 9: Figure S2 — Expression profiles of all identified CCR genes. [file 1471-2164-14-450-S9.zip › FigureS2/CCNA_00161.pdf]

# CCNA\_00162

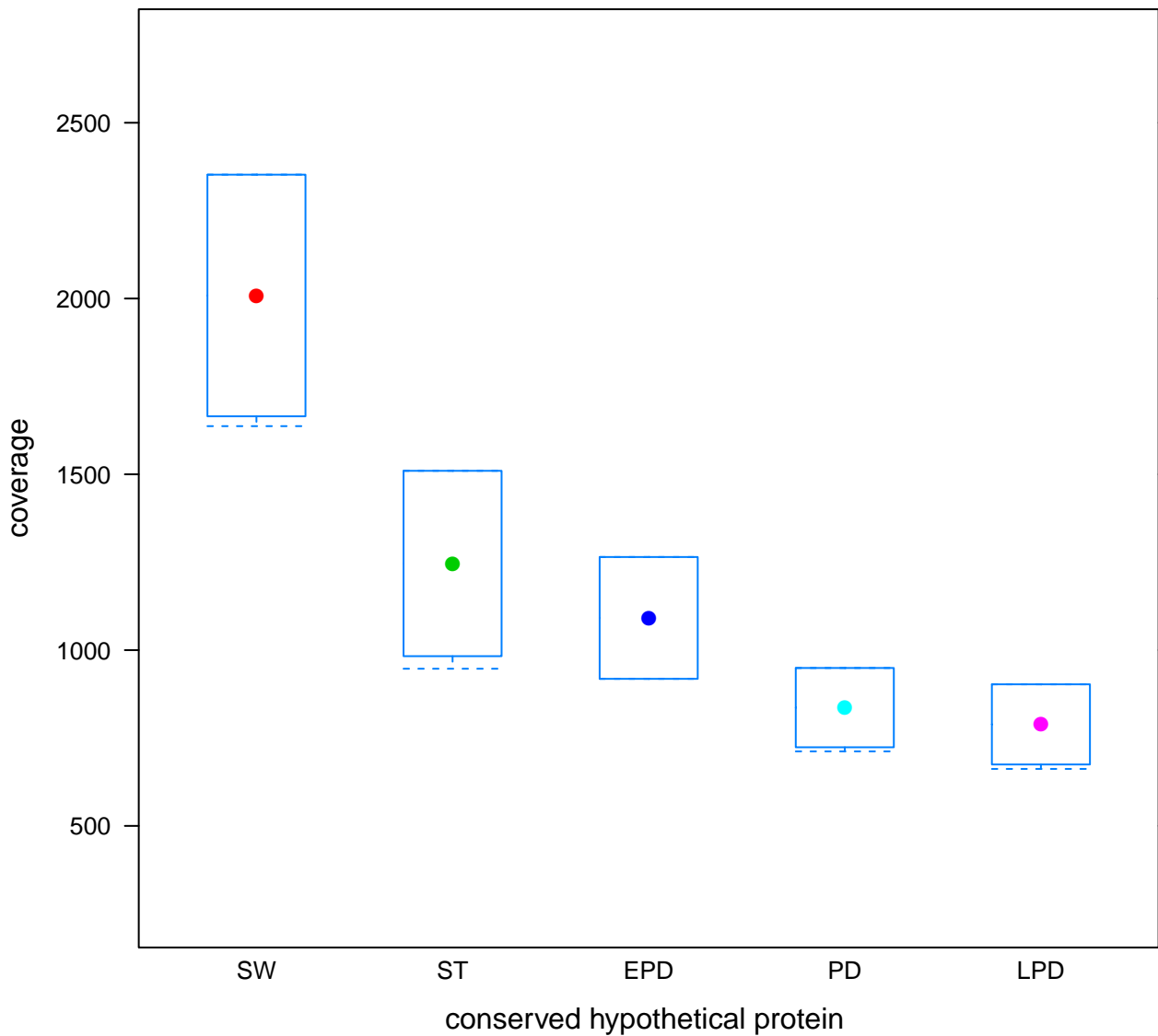

**Fold of change: 2.78**  
**baySeq likelihood: 0.737**

Supplement: Additional file 9: Figure S2 — Expression profiles of all identified CCR genes. [file 1471-2164-14-450-S9.zip › FigureS2/CCNA_00162.pdf]

# CCNA\_00163

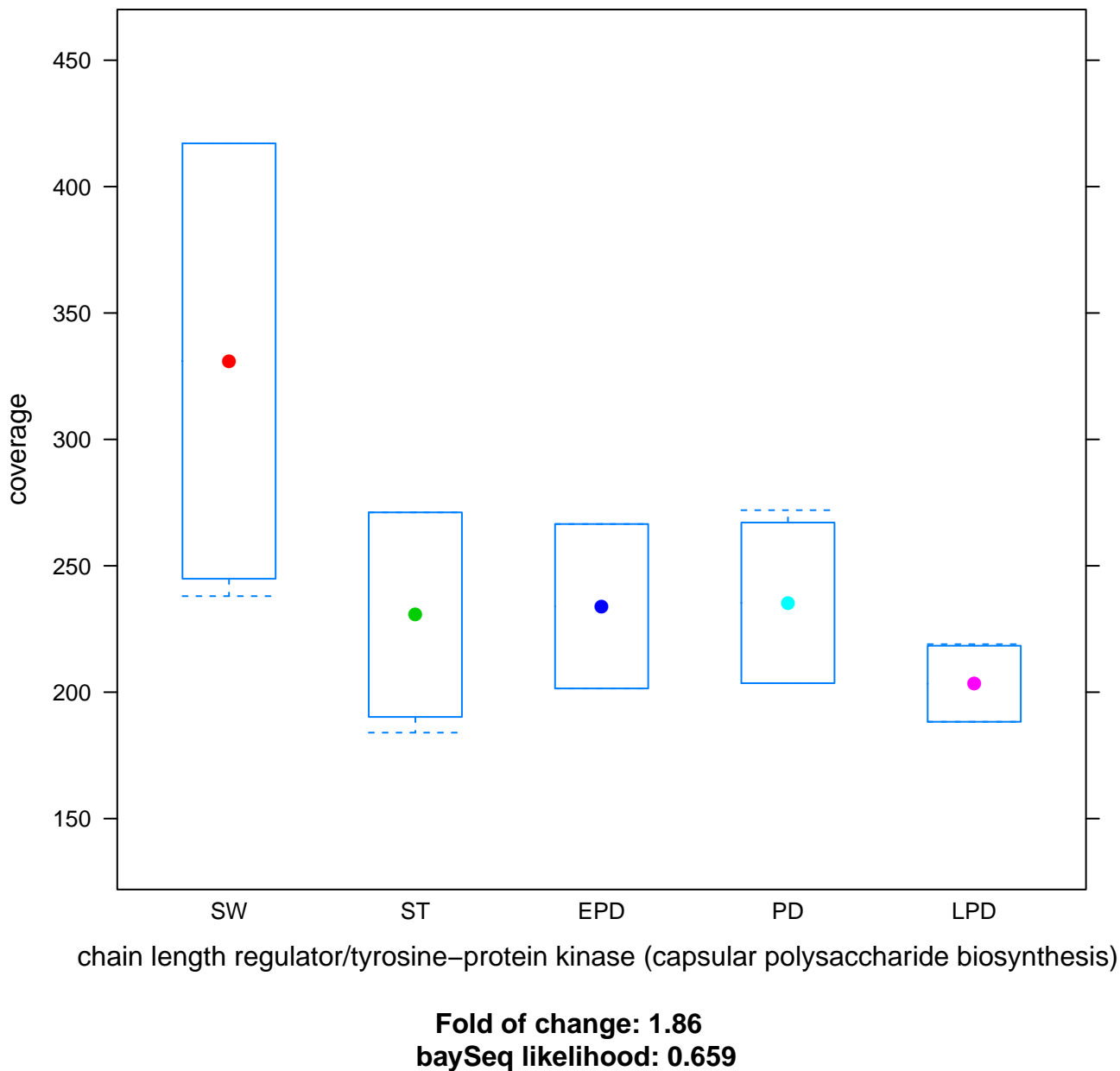

Supplement: Additional file 9: Figure S2 — Expression profiles of all identified CCR genes. [file 1471-2164-14-450-S9.zip › FigureS2/CCNA_00163.pdf]

# CCNA\_00166

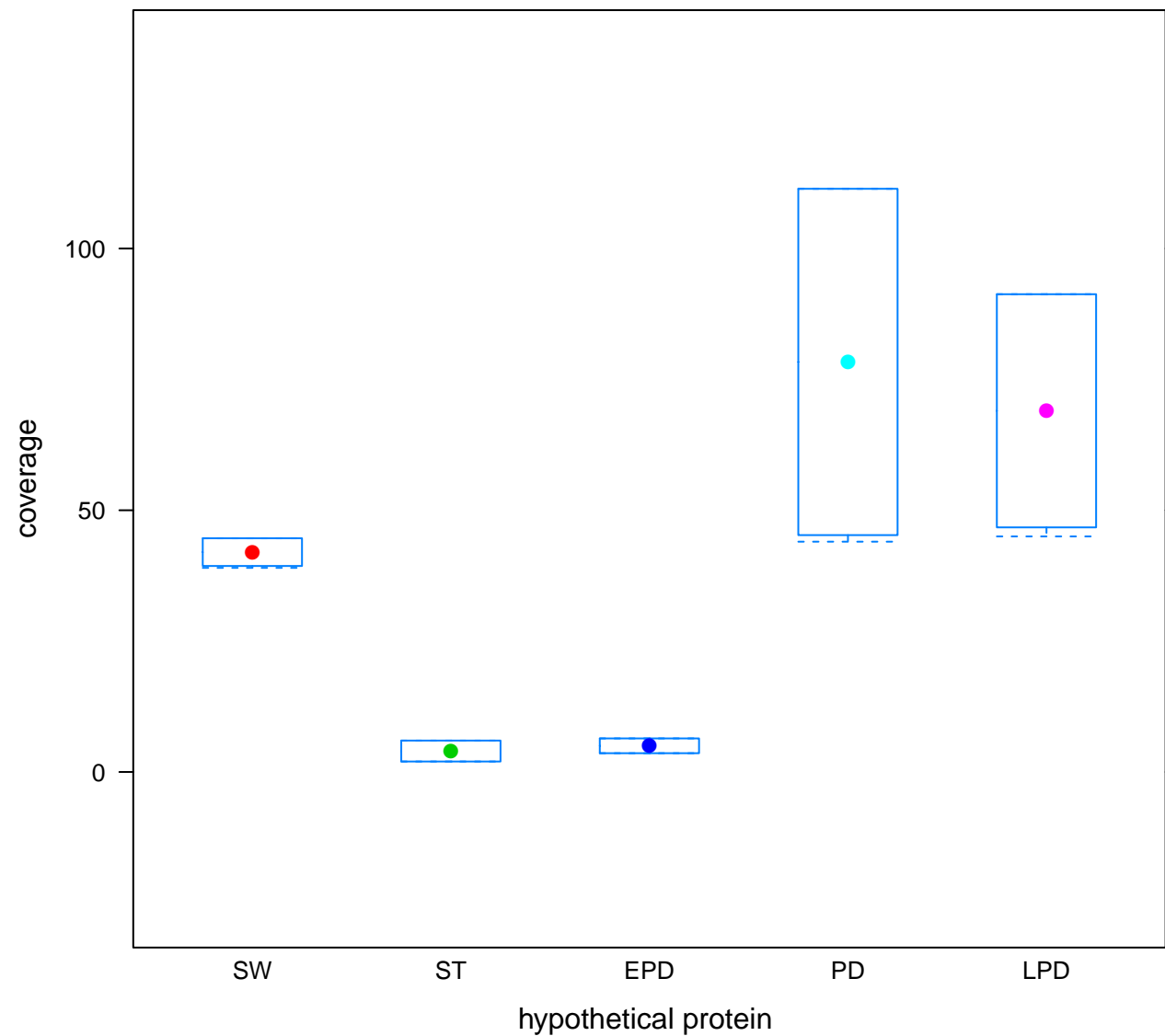

**Fold of change: 15.67**

**baySeq likelihood: 1**

Supplement: Additional file 9: Figure S2 — Expression profiles of all identified CCR genes. [file 1471-2164-14-450-S9.zip › FigureS2/CCNA_00166.pdf]

# CCNA\_00167

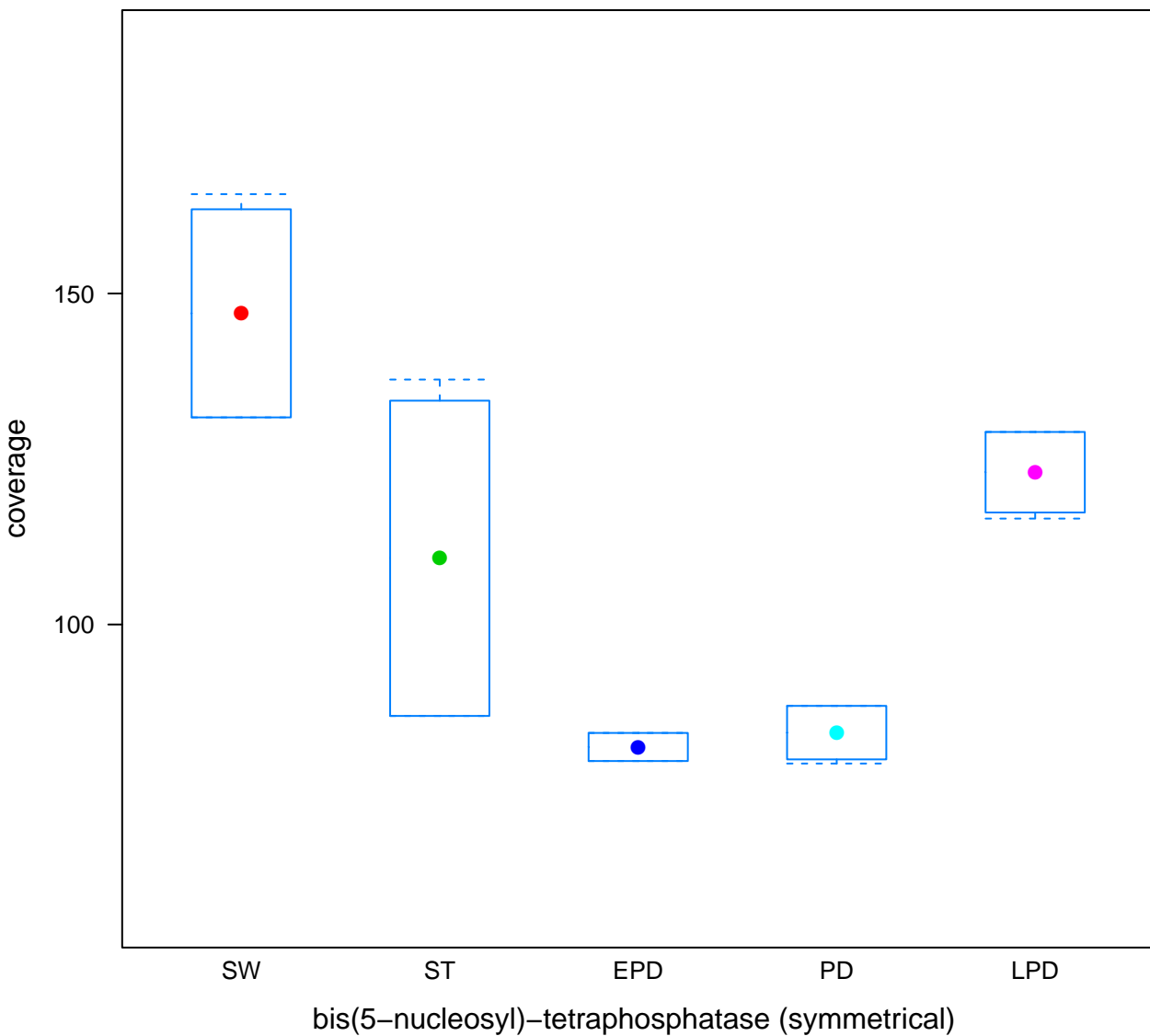

**Fold of change: 1.69**  
**baySeq likelihood: 0.323**

Supplement: Additional file 9: Figure S2 — Expression profiles of all identified CCR genes. [file 1471-2164-14-450-S9.zip › FigureS2/CCNA_00167.pdf]

# CCNA\_00168

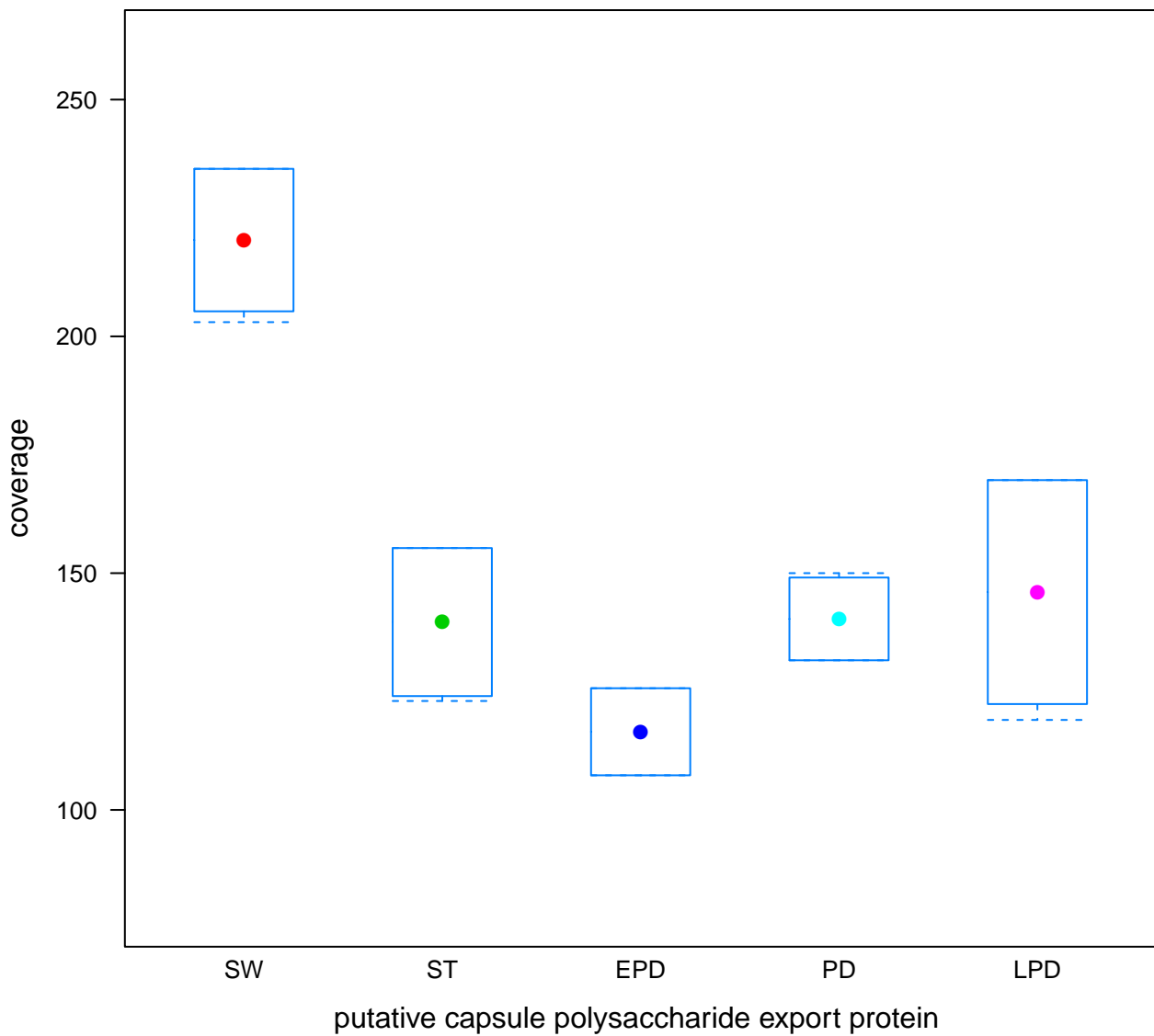

**Fold of change: 1.97**  
**baySeq likelihood: 0.99**

Supplement: Additional file 9: Figure S2 — Expression profiles of all identified CCR genes. [file 1471-2164-14-450-S9.zip › FigureS2/CCNA_00168.pdf]

# CCNA\_00169

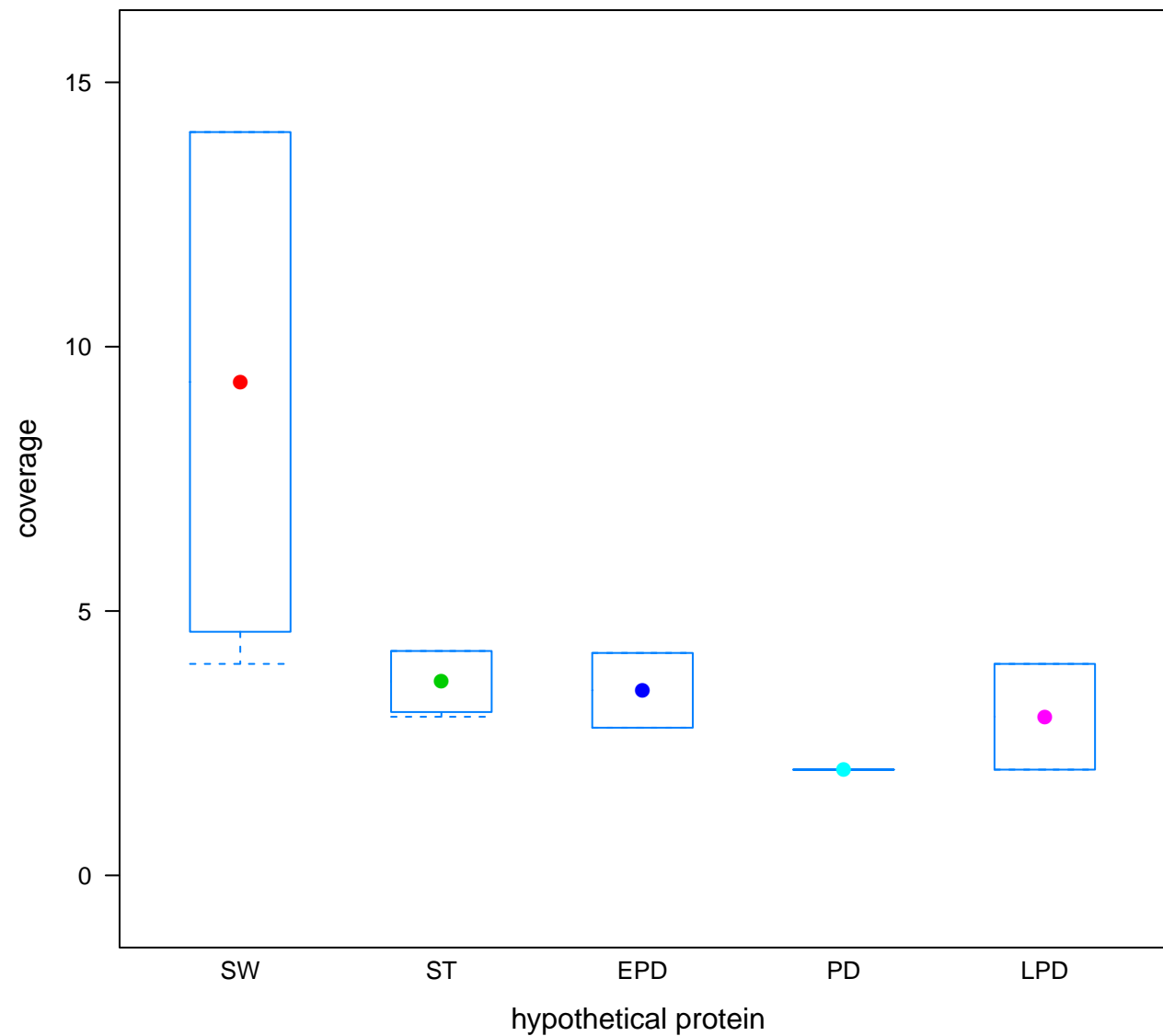

**Fold of change: 6**  
**baySeq likelihood: 0.985**

Supplement: Additional file 9: Figure S2 — Expression profiles of all identified CCR genes. [file 1471-2164-14-450-S9.zip › FigureS2/CCNA_00169.pdf]

# CCNA\_00184

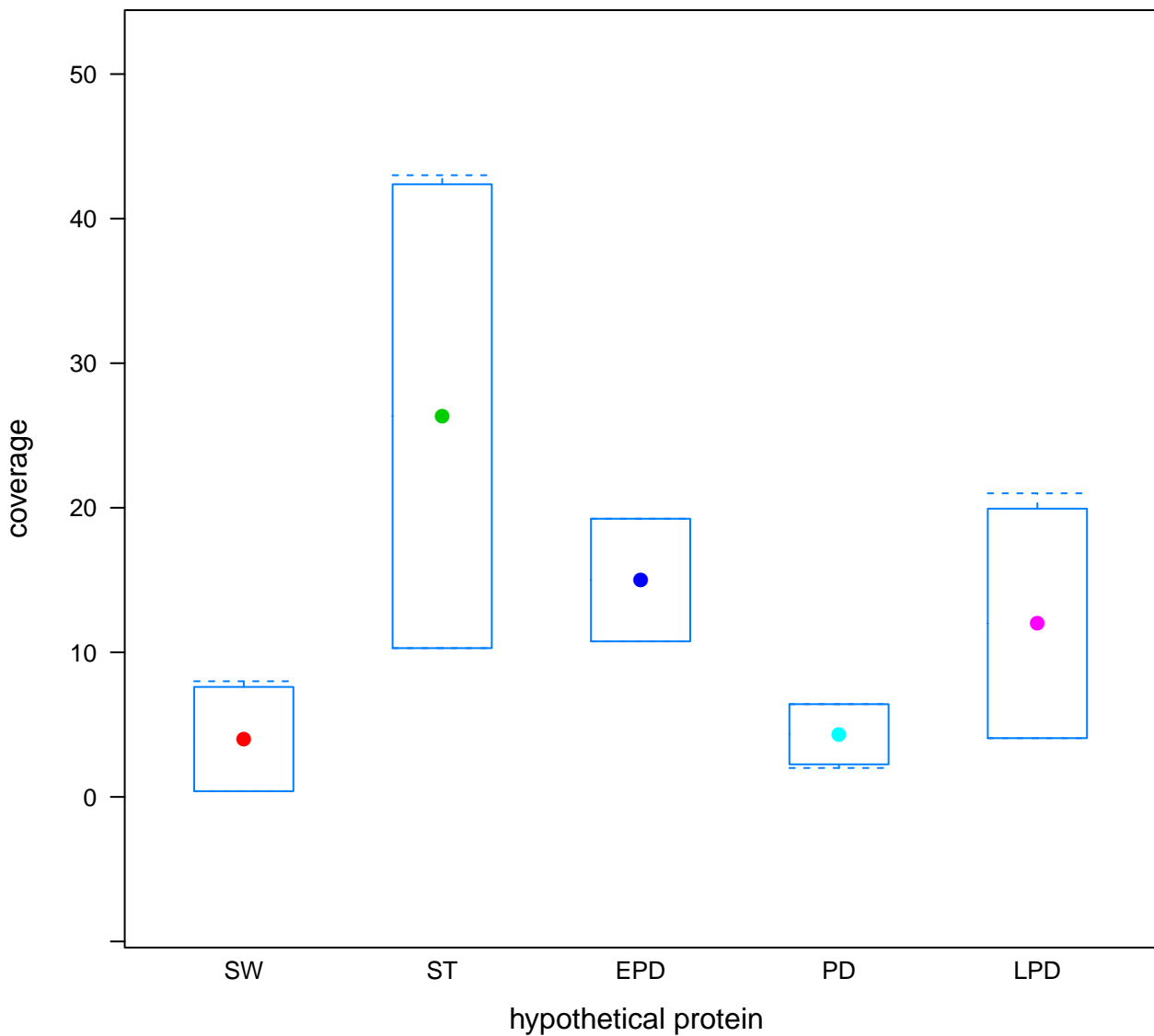

**Fold of change: 17**  
**baySeq likelihood: 0.896**

Supplement: Additional file 9: Figure S2 — Expression profiles of all identified CCR genes. [file 1471-2164-14-450-S9.zip › FigureS2/CCNA_00184.pdf]

# CCNA\_00185

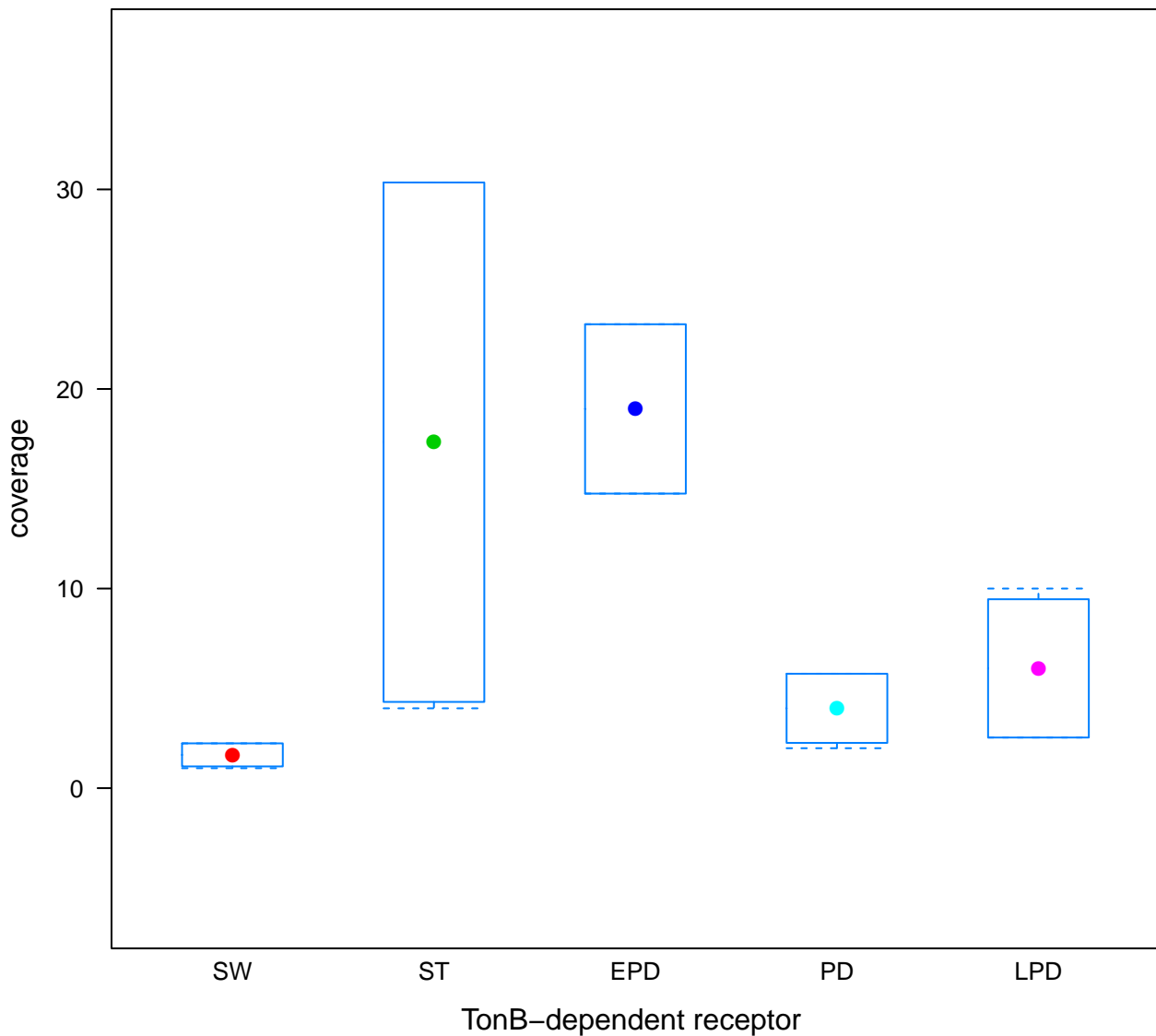

**Fold of change: 16**  
**baySeq likelihood: 0.934**

Supplement: Additional file 9: Figure S2 — Expression profiles of all identified CCR genes. [file 1471-2164-14-450-S9.zip › FigureS2/CCNA_00185.pdf]

# CCNA\_00186

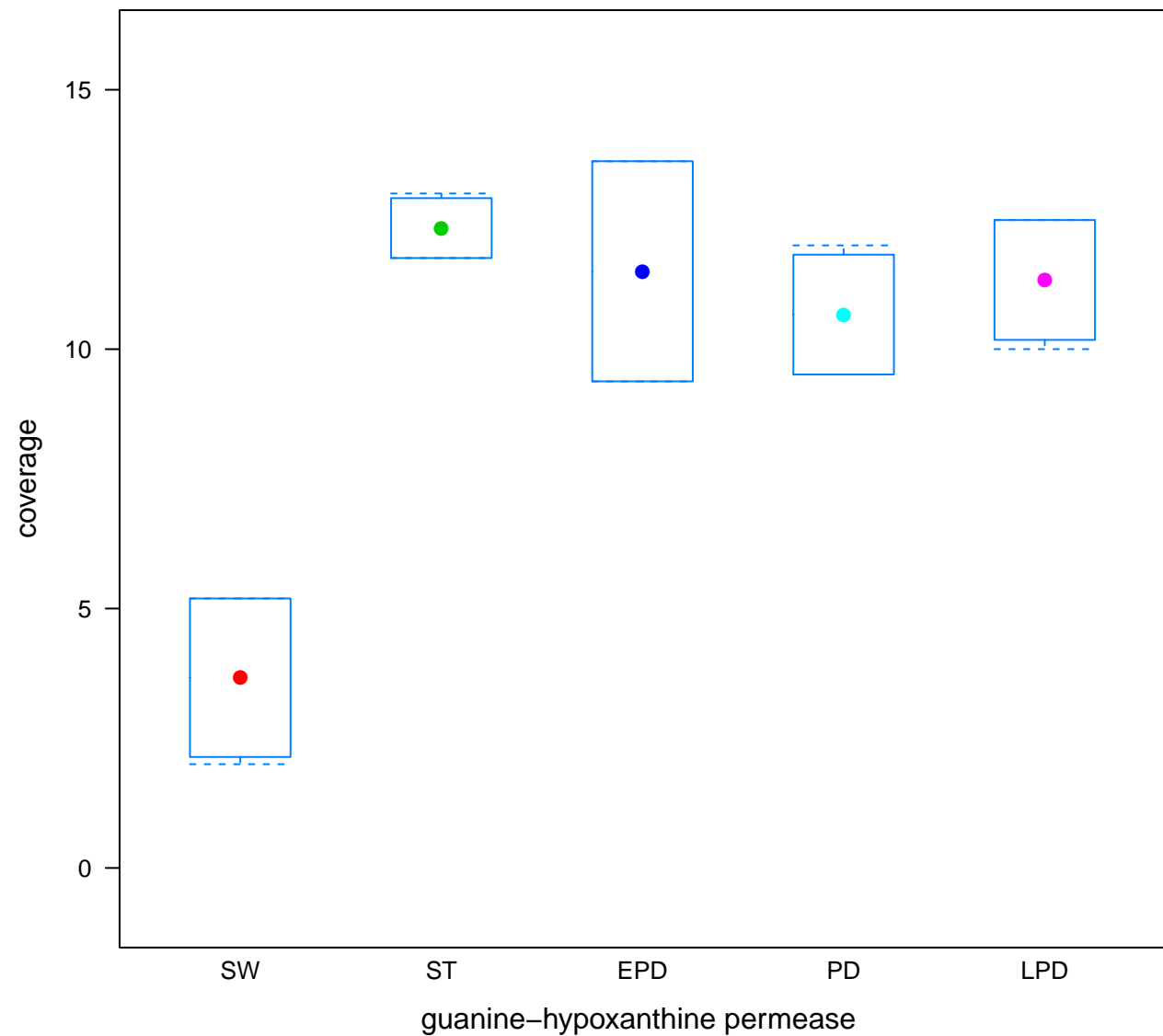

**Fold of change: 4**  
**baySeq likelihood: 0.989**

Supplement: Additional file 9: Figure S2 — Expression profiles of all identified CCR genes. [file 1471-2164-14-450-S9.zip › FigureS2/CCNA_00186.pdf]

# CCNA\_00187

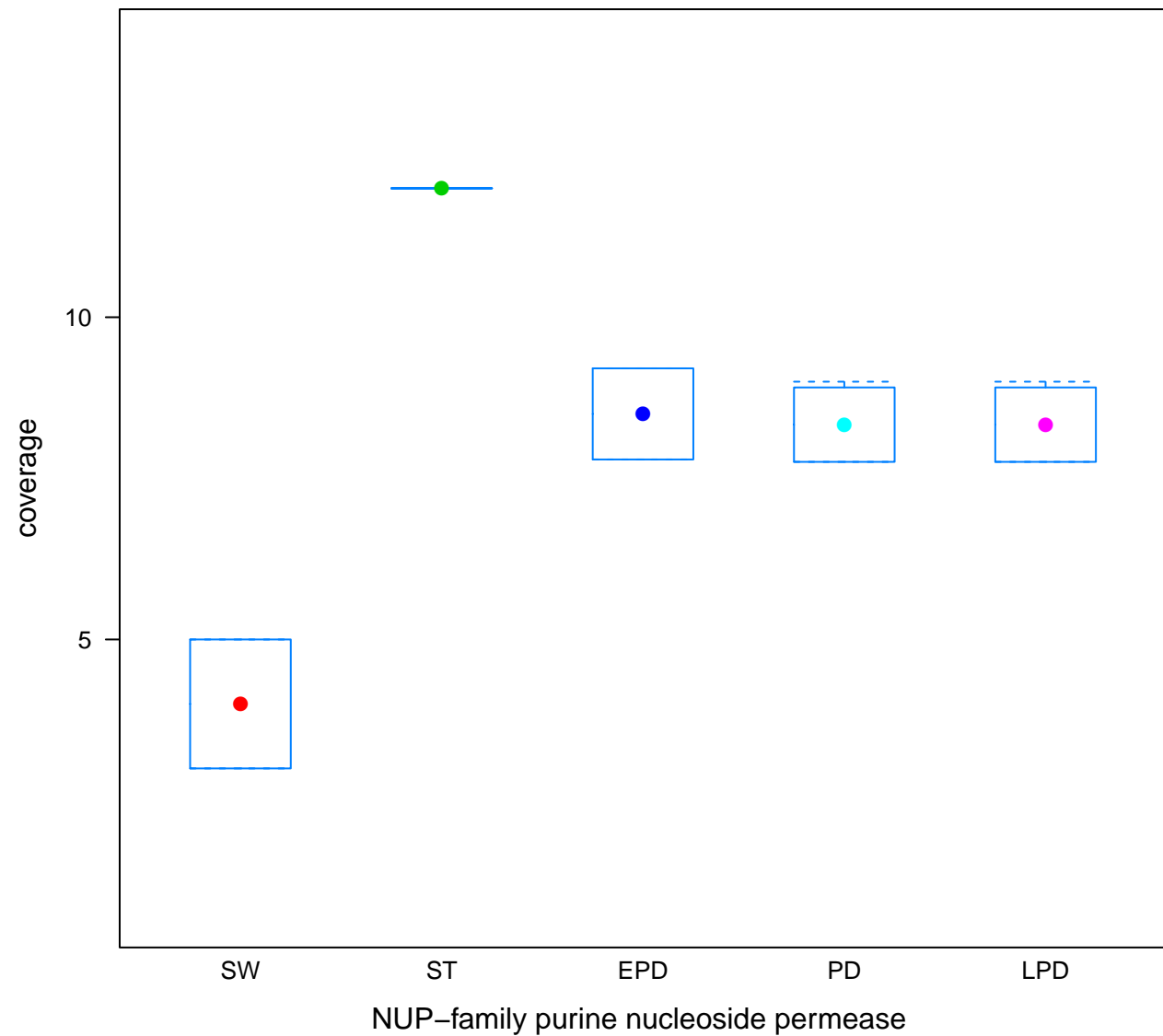

**Fold of change: 3.43**  
**baySeq likelihood: 0.643**

Supplement: Additional file 9: Figure S2 — Expression profiles of all identified CCR genes. [file 1471-2164-14-450-S9.zip › FigureS2/CCNA_00187.pdf]

# CCNA\_00192

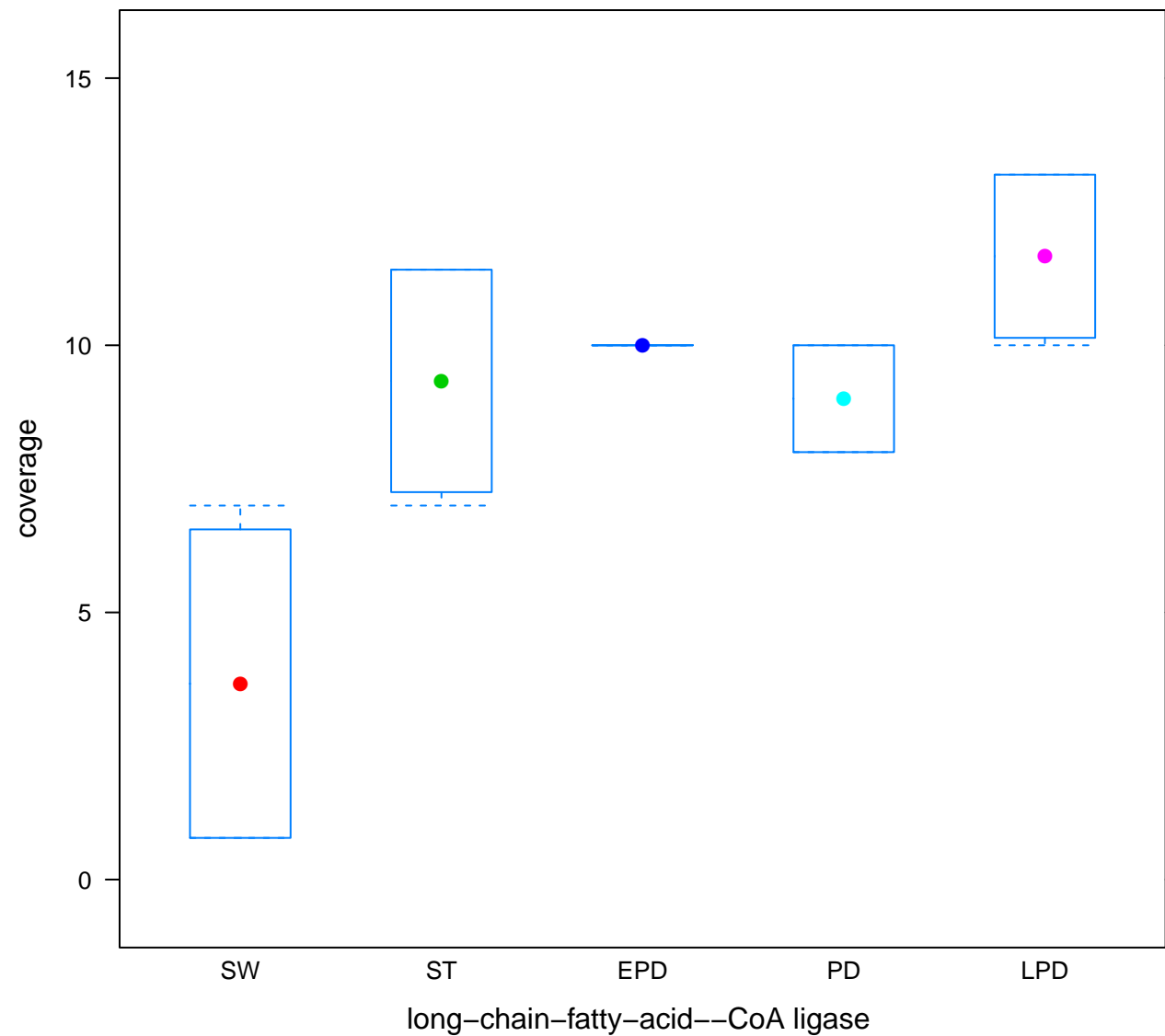

**Fold of change: 5.83**  
**baySeq likelihood: 0.902**

Supplement: Additional file 9: Figure S2 — Expression profiles of all identified CCR genes. [file 1471-2164-14-450-S9.zip › FigureS2/CCNA_00192.pdf]

# leuB;CCNA\_00193

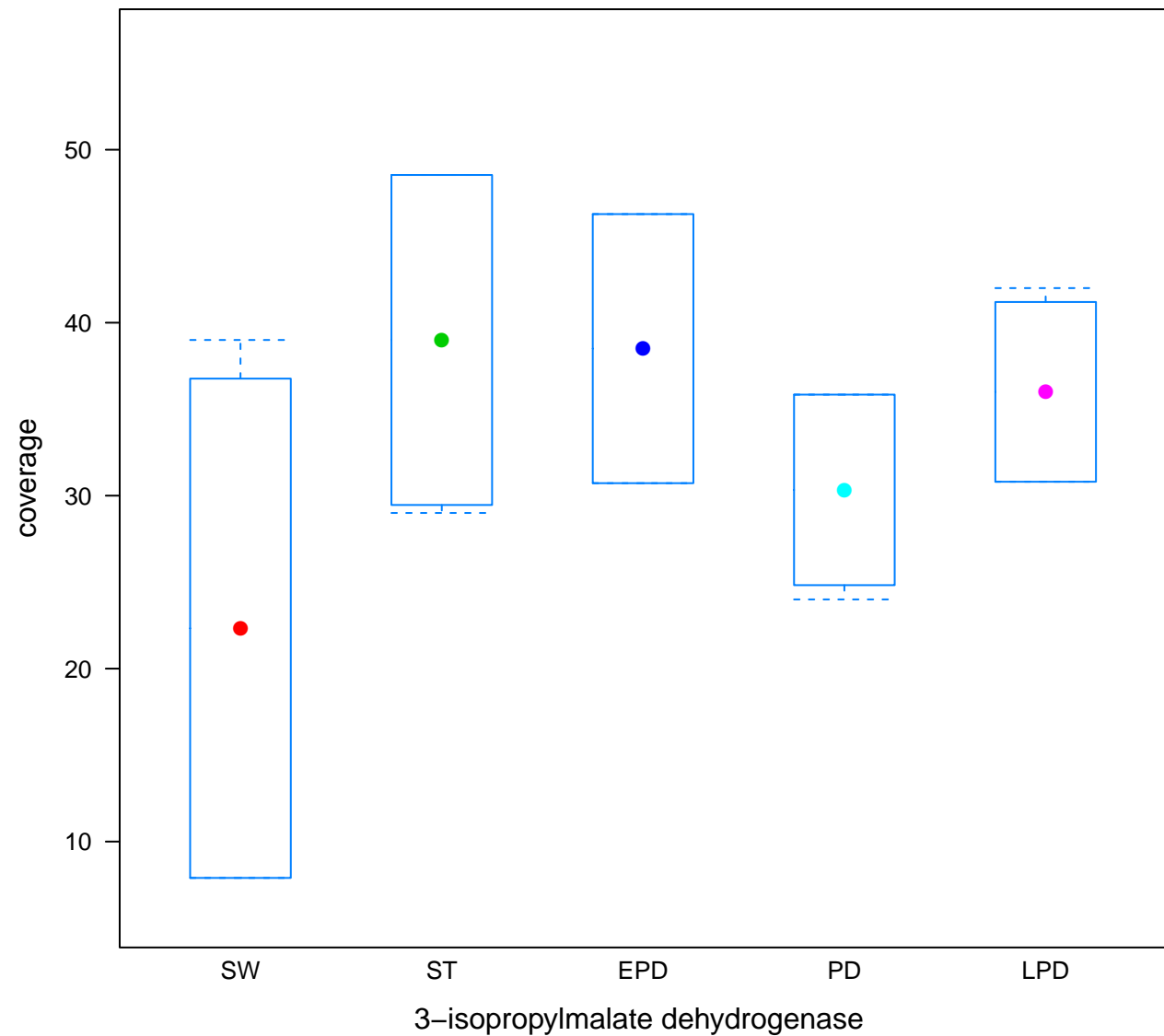

**Fold of change: 3.14**  
**baySeq likelihood: 0.558**

Supplement: Additional file 9: Figure S2 — Expression profiles of all identified CCR genes. [file 1471-2164-14-450-S9.zip › FigureS2/CCNA_00193.pdf]

# CCNA\_00194

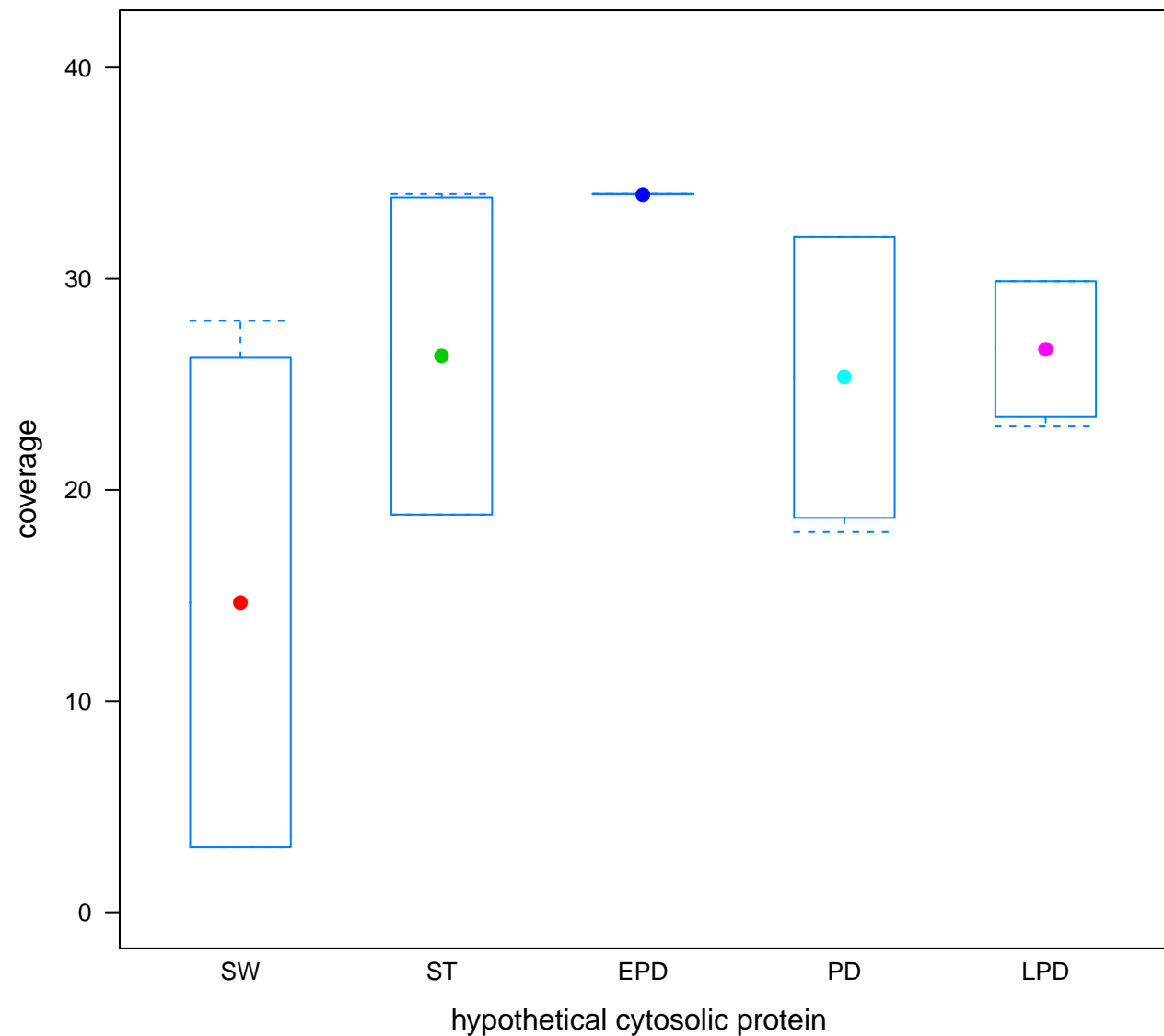

**Fold of change: 4.25**  
**baySeq likelihood: 0.792**

Supplement: Additional file 9: Figure S2 — Expression profiles of all identified CCR genes. [file 1471-2164-14-450-S9.zip › FigureS2/CCNA_00194.pdf]

# CCNA\_00199

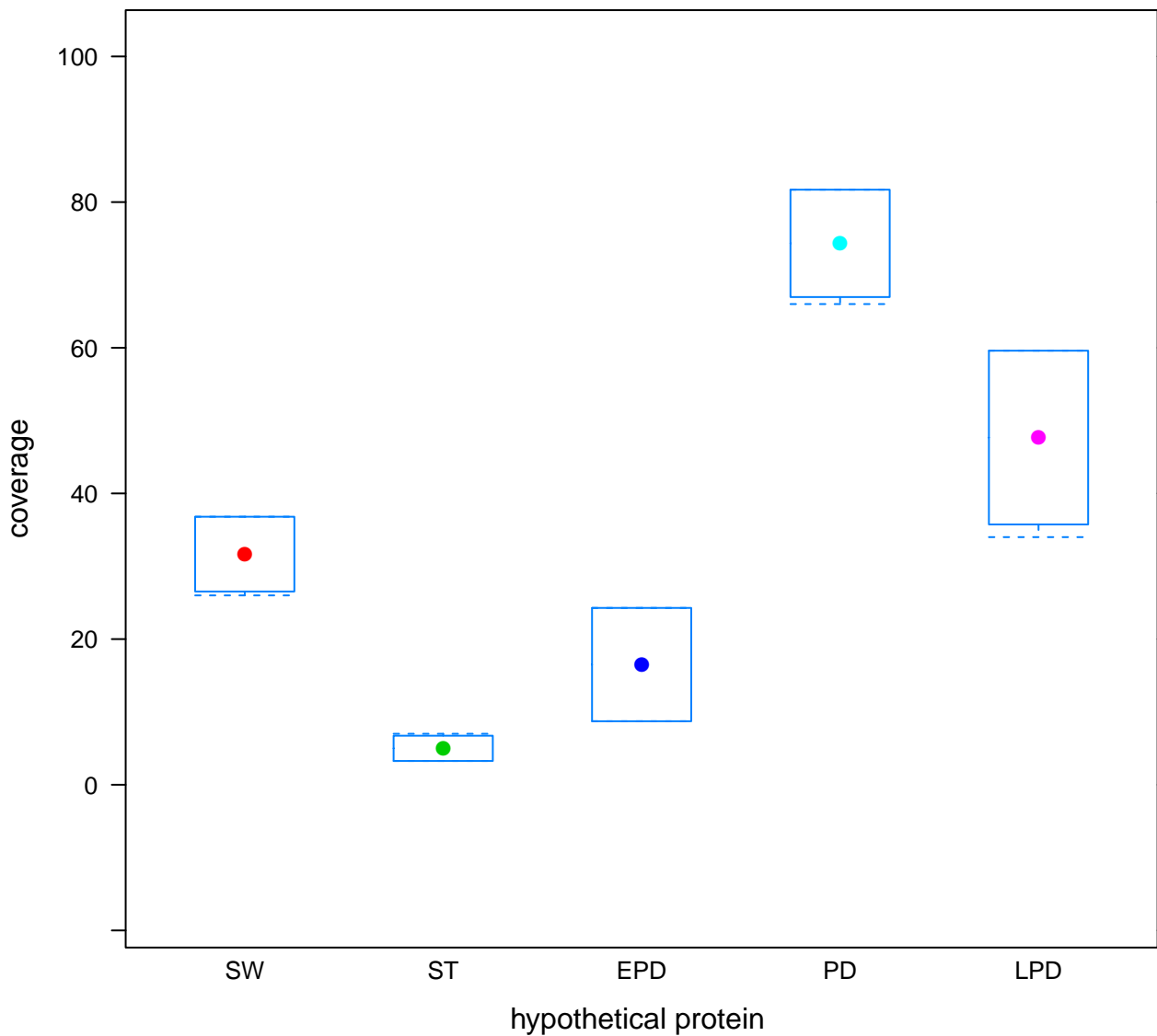

**Fold of change: 13.52**  
**baySeq likelihood: 0.795**

Supplement: Additional file 9: Figure S2 — Expression profiles of all identified CCR genes. [file 1471-2164-14-450-S9.zip › FigureS2/CCNA_00199.pdf]

# CCNA\_00203

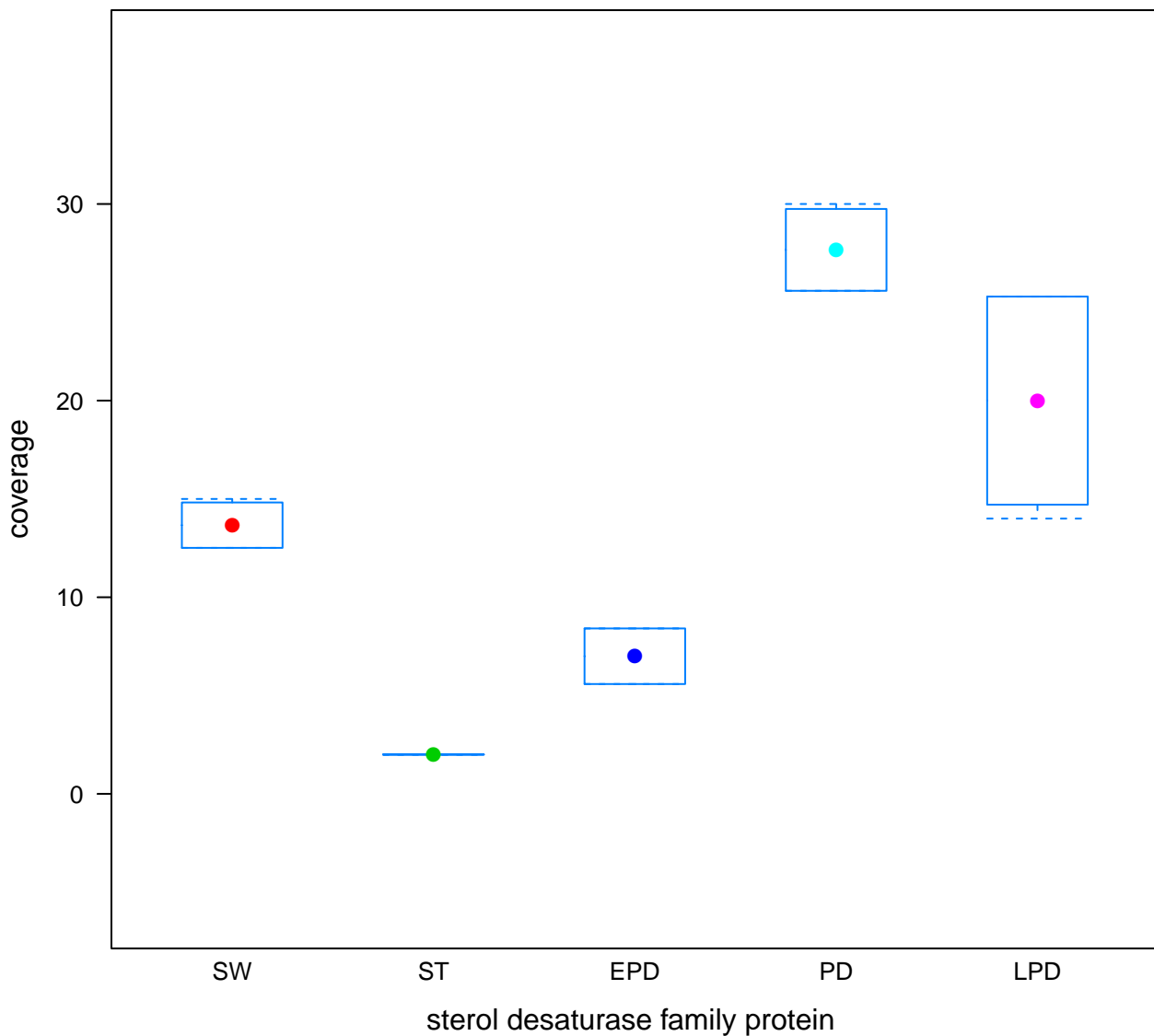

**Fold of change: 13.83**  
**baySeq likelihood: 0.949**

Supplement: Additional file 9: Figure S2 — Expression profiles of all identified CCR genes. [file 1471-2164-14-450-S9.zip › FigureS2/CCNA_00203.pdf]

# CCNA\_00210

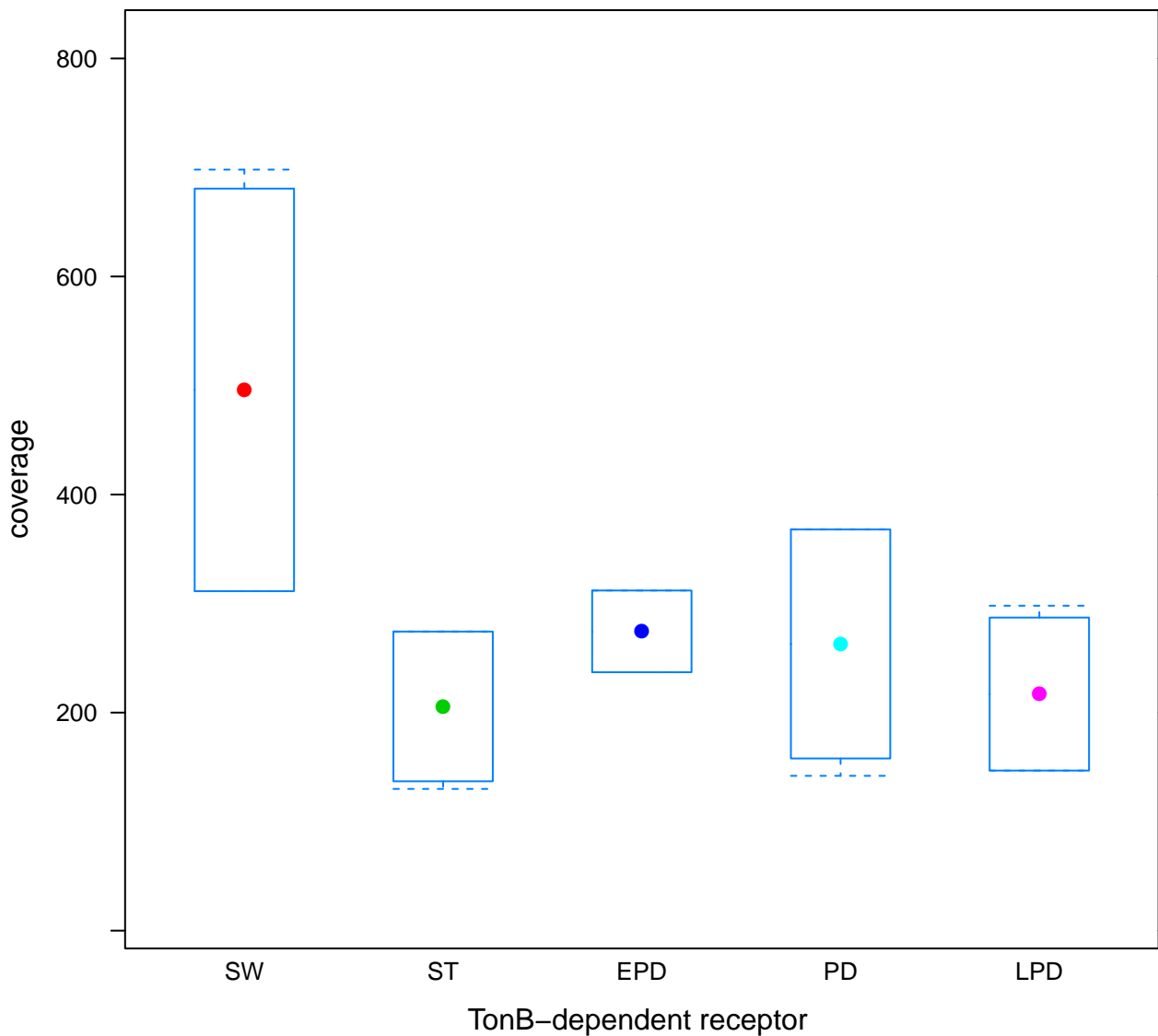

**Fold of change: 2.65**  
**baySeq likelihood: 0.171**

Supplement: Additional file 9: Figure S2 — Expression profiles of all identified CCR genes. [file 1471-2164-14-450-S9.zip › FigureS2/CCNA_00210.pdf]

# CCNA\_00211

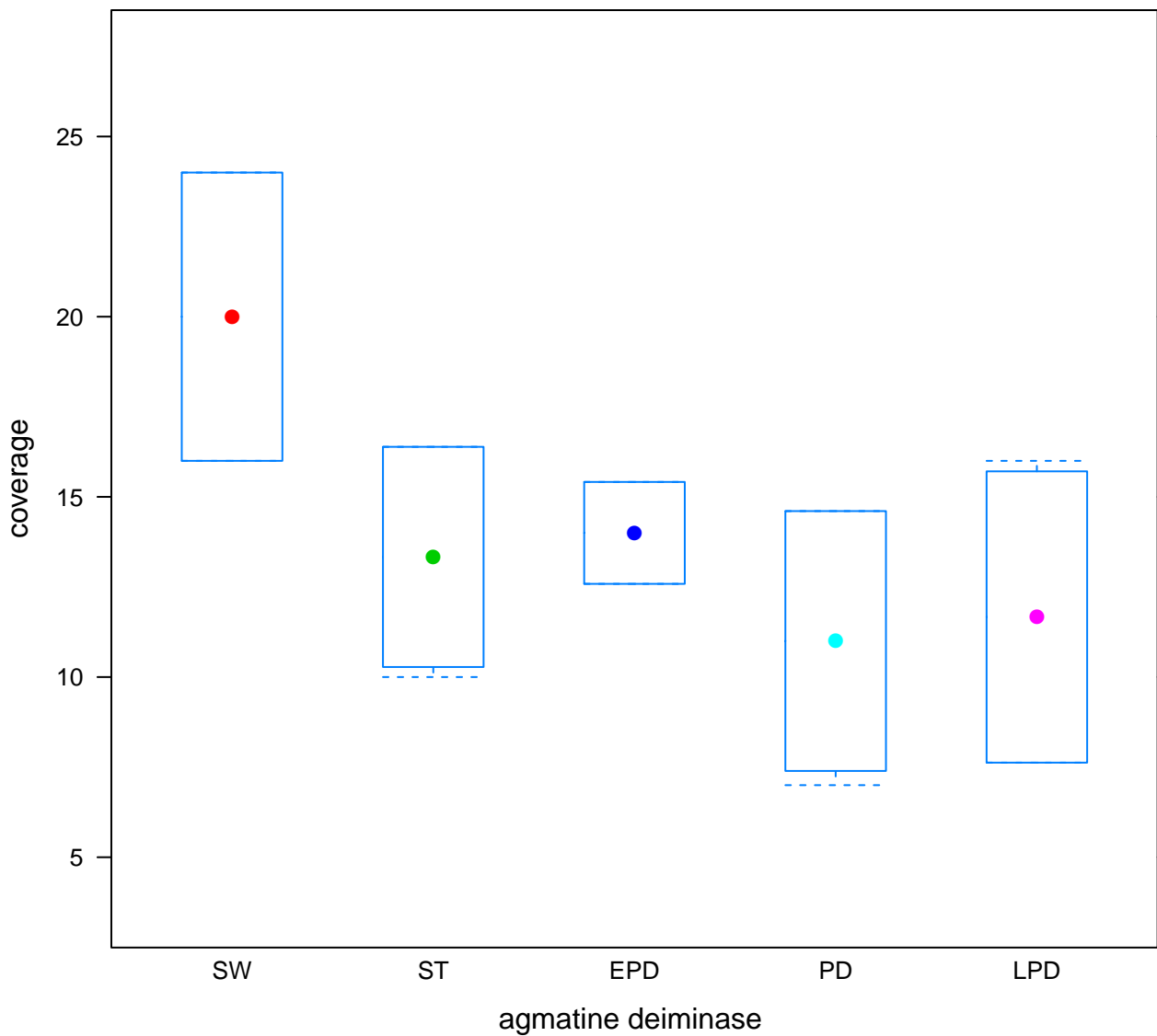

**Fold of change: 2**  
**baySeq likelihood: 0.768**

Supplement: Additional file 9: Figure S2 — Expression profiles of all identified CCR genes. [file 1471-2164-14-450-S9.zip › FigureS2/CCNA_00211.pdf]

# CCNA\_00213

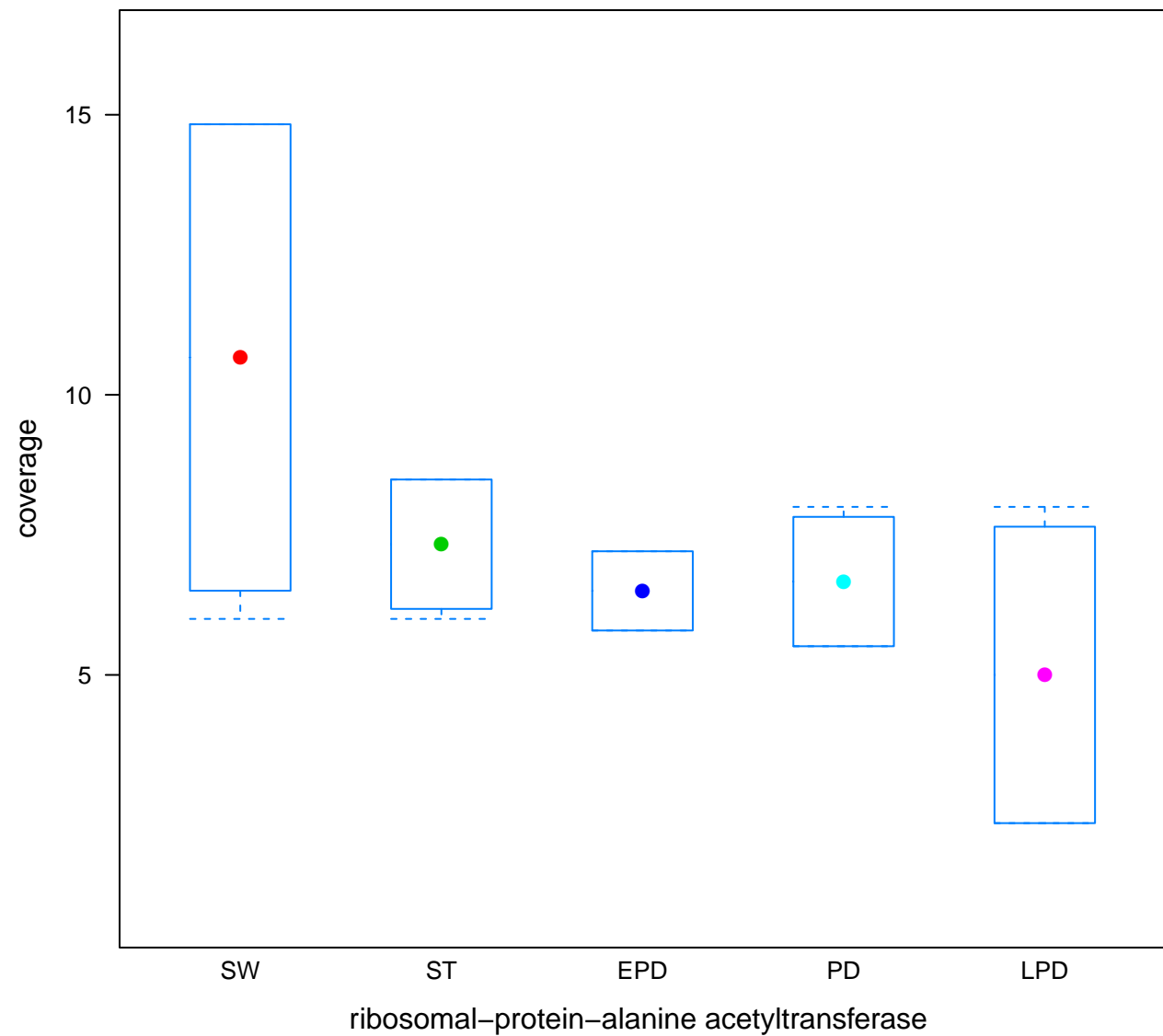

**Fold of change: 2.6**  
**baySeq likelihood: 0.396**

Supplement: Additional file 9: Figure S2 — Expression profiles of all identified CCR genes. [file 1471-2164-14-450-S9.zip › FigureS2/CCNA_00213.pdf]

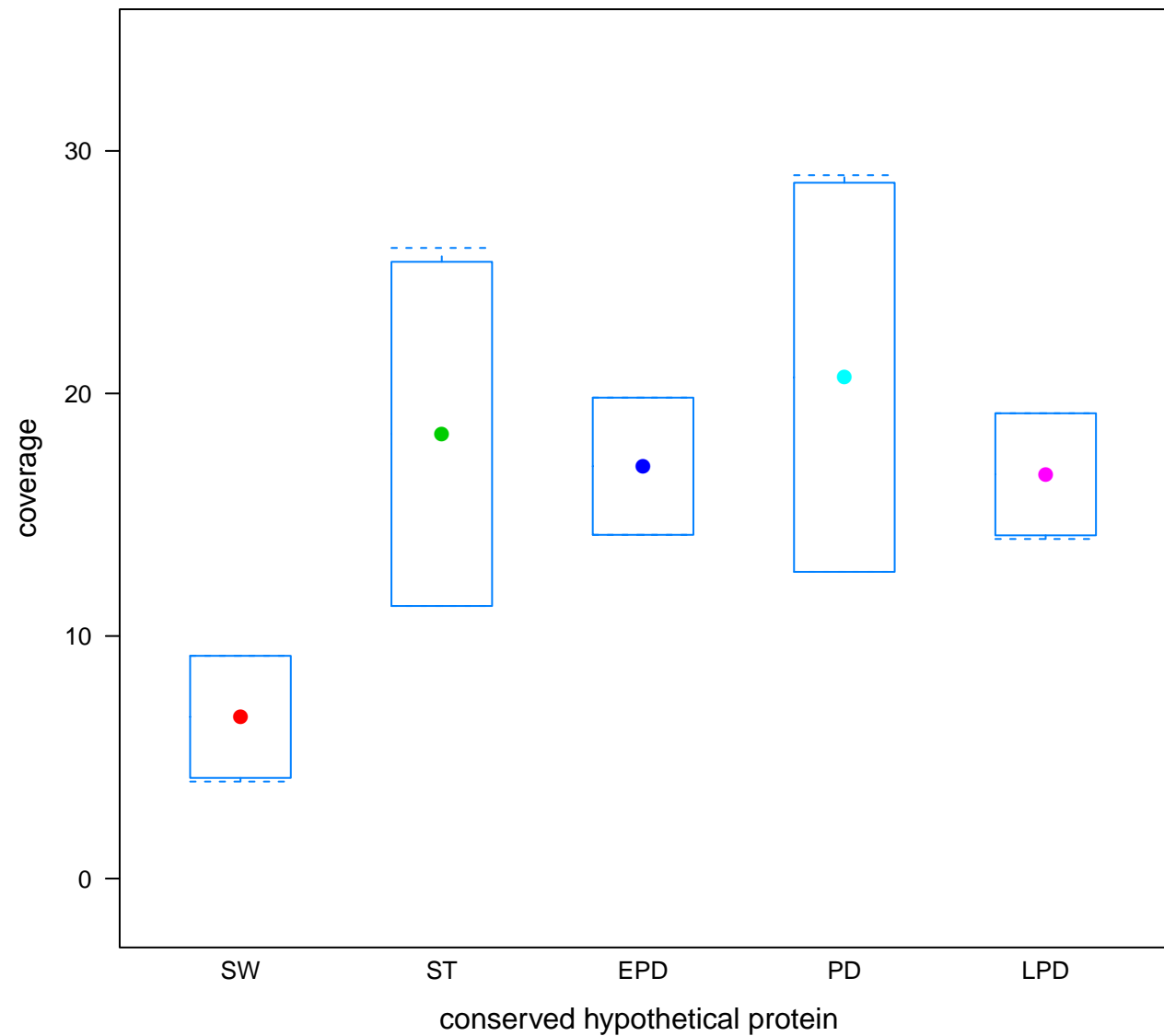

**Fold of change: 3.76**  
**baySeq likelihood: 0.988**

Supplement: Additional file 9: Figure S2 — Expression profiles of all identified CCR genes. [file 1471-2164-14-450-S9.zip › FigureS2/CCNA_00216.pdf]

# CCNA\_00221

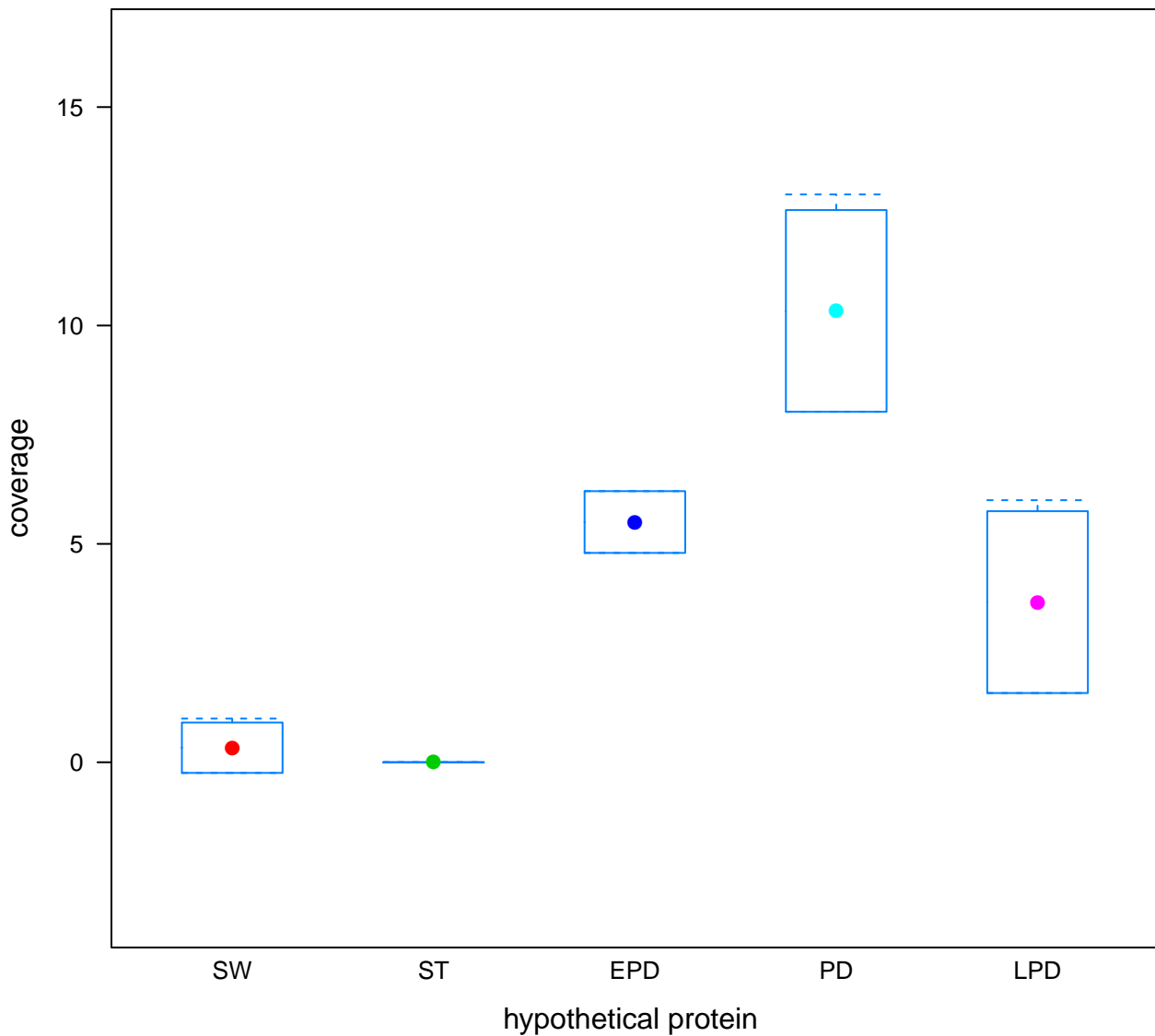

**Fold of change: Inf**  
**baySeq likelihood: 0.999**

Supplement: Additional file 9: Figure S2 — Expression profiles of all identified CCR genes. [file 1471-2164-14-450-S9.zip › FigureS2/CCNA_00221.pdf]

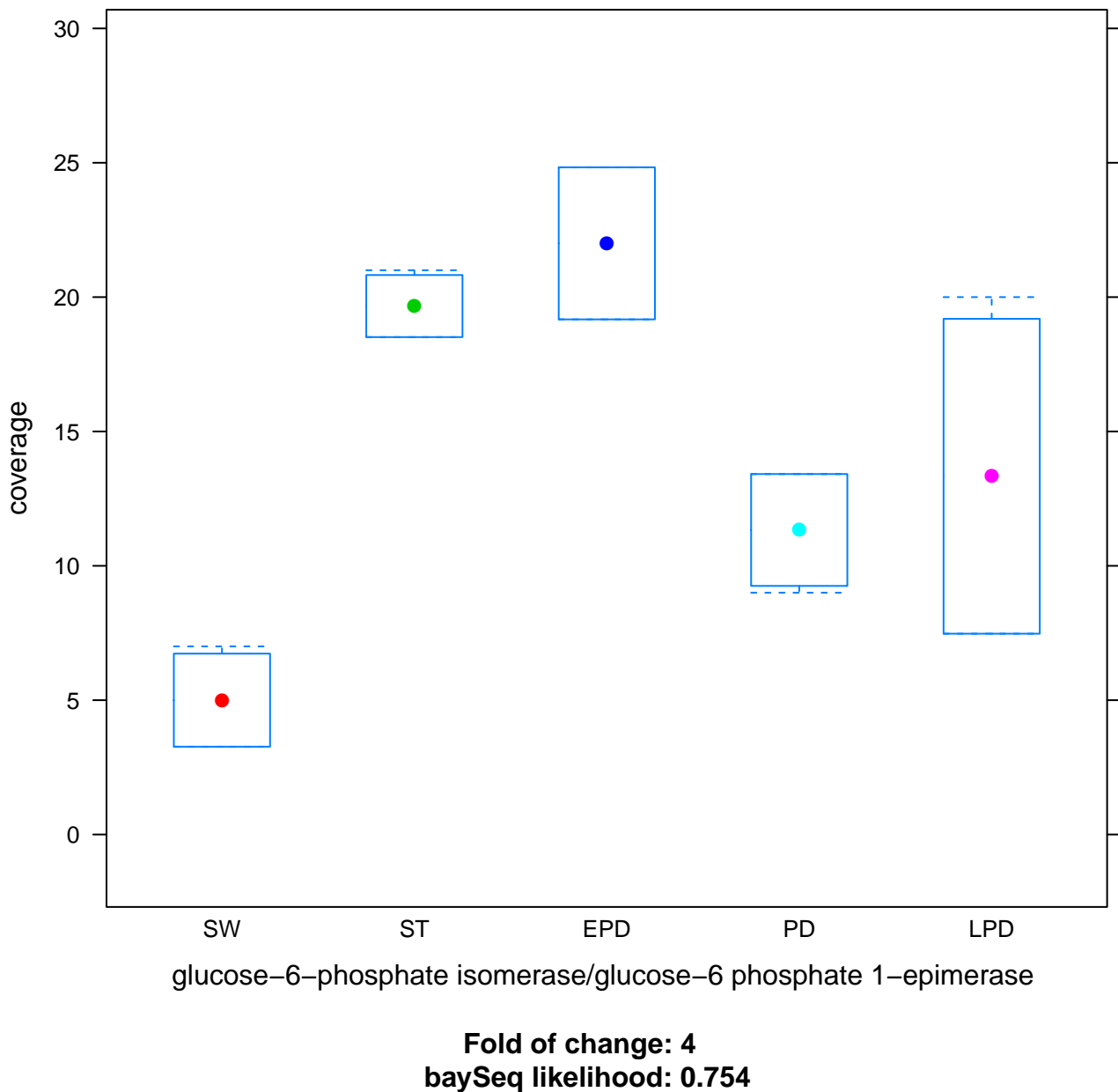

Supplement: Additional file 9: Figure S2 — Expression profiles of all identified CCR genes. [file 1471-2164-14-450-S9.zip › FigureS2/CCNA_00222.pdf]

# CCNA\_00223

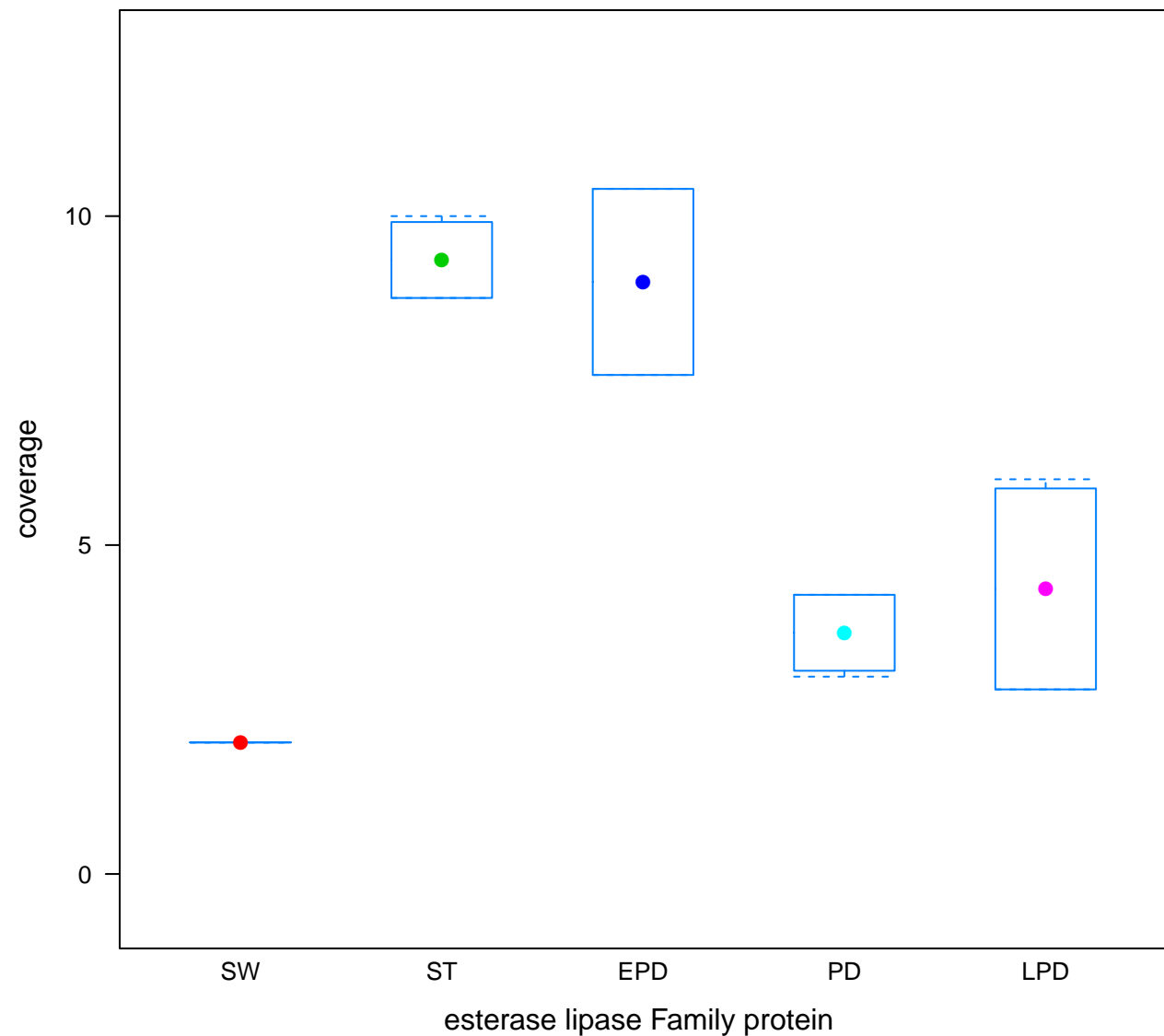

**Fold of change: 4.75**  
**baySeq likelihood: 0.922**

Supplement: Additional file 9: Figure S2 — Expression profiles of all identified CCR genes. [file 1471-2164-14-450-S9.zip › FigureS2/CCNA_00223.pdf]

# CCNA\_00229

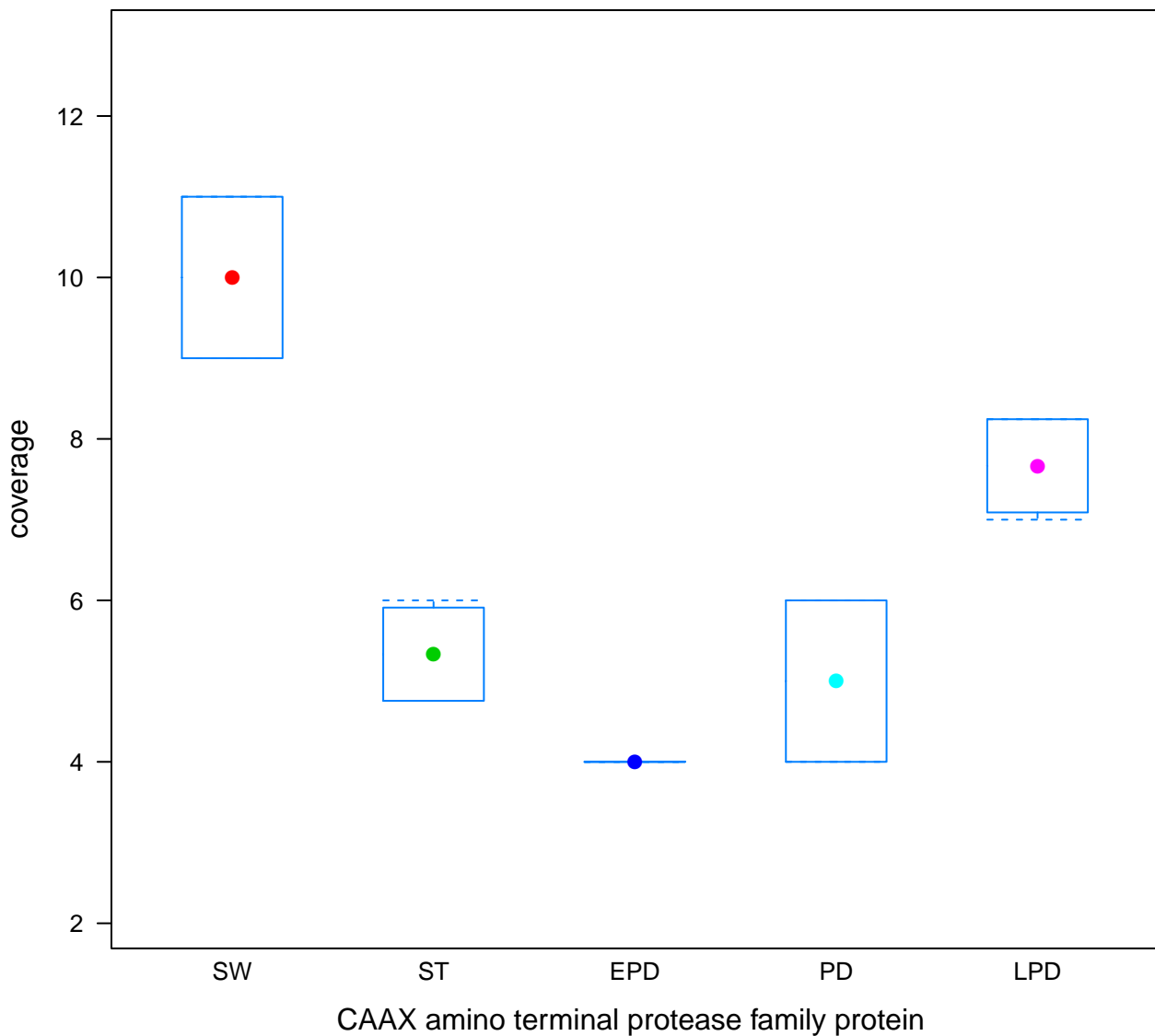

**Fold of change: 2.5**  
**baySeq likelihood: 0.474**

Supplement: Additional file 9: Figure S2 — Expression profiles of all identified CCR genes. [file 1471-2164-14-450-S9.zip › FigureS2/CCNA_00229.pdf]

# CCNA\_00230

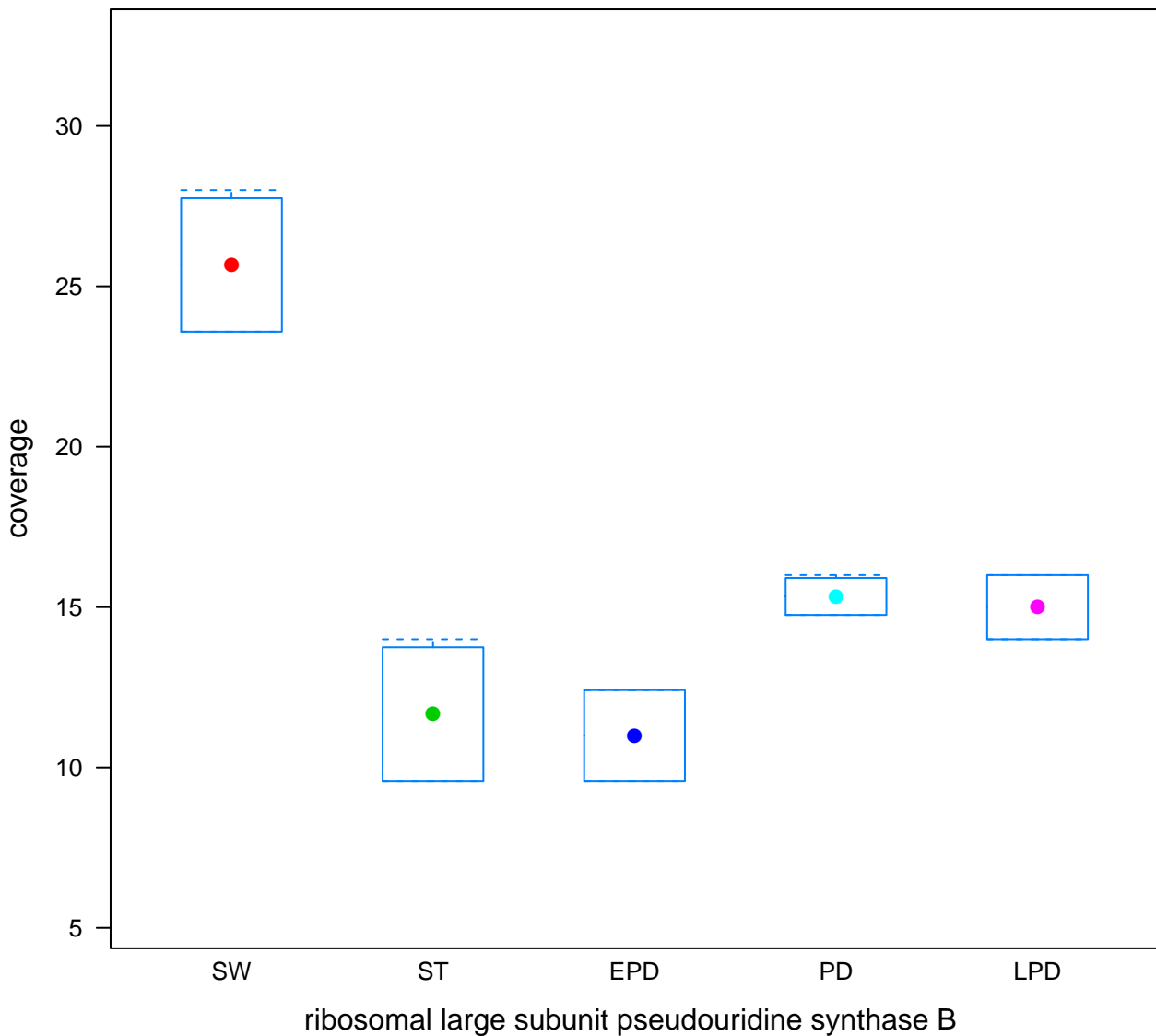

**Fold of change: 2.36**  
**baySeq likelihood: 0.992**

Supplement: Additional file 9: Figure S2 — Expression profiles of all identified CCR genes. [file 1471-2164-14-450-S9.zip › FigureS2/CCNA_00230.pdf]

# CCNA\_00232

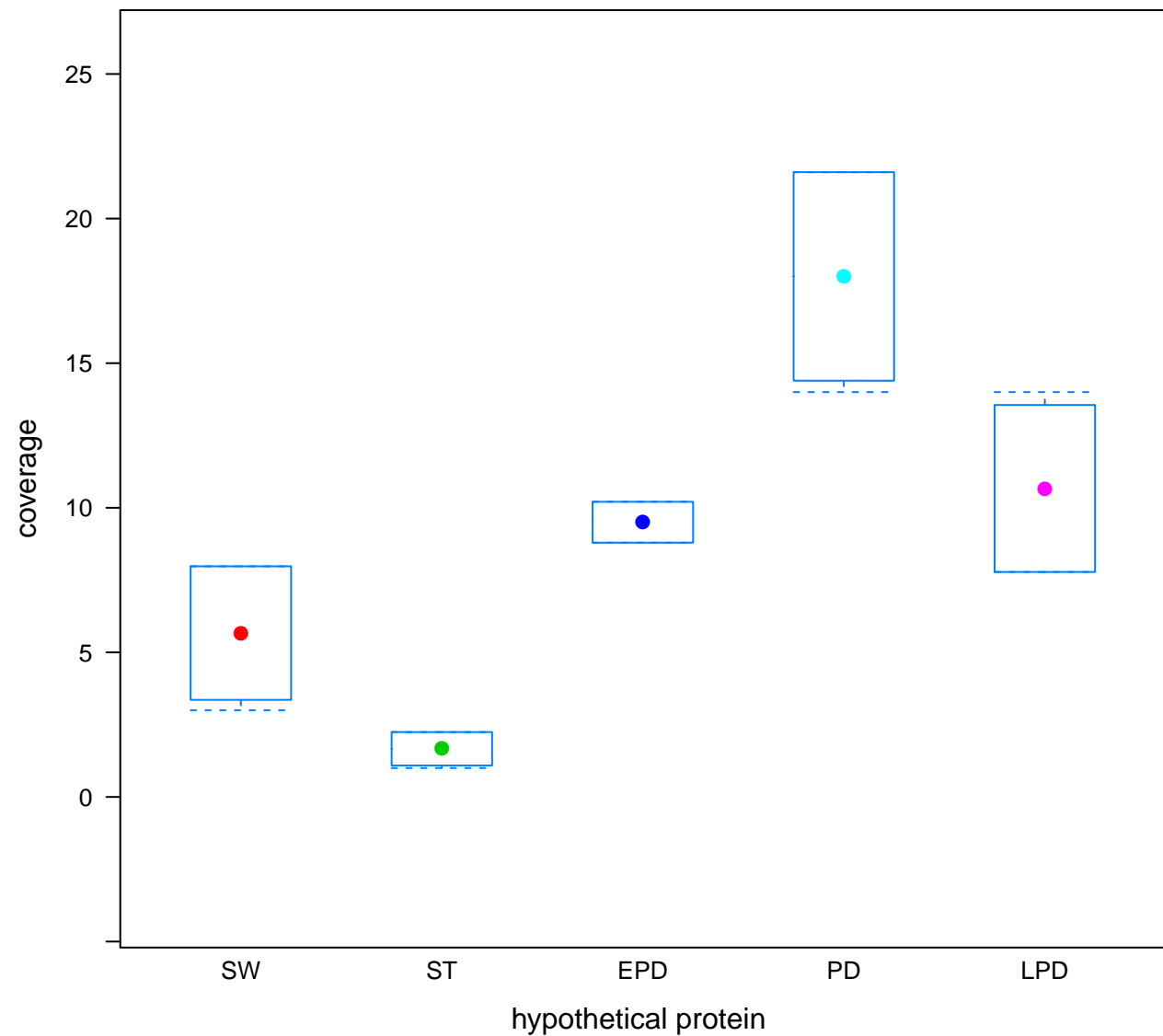

**Fold of change: 12**  
**baySeq likelihood: 0.831**

Supplement: Additional file 9: Figure S2 — Expression profiles of all identified CCR genes. [file 1471-2164-14-450-S9.zip › FigureS2/CCNA_00232.pdf]

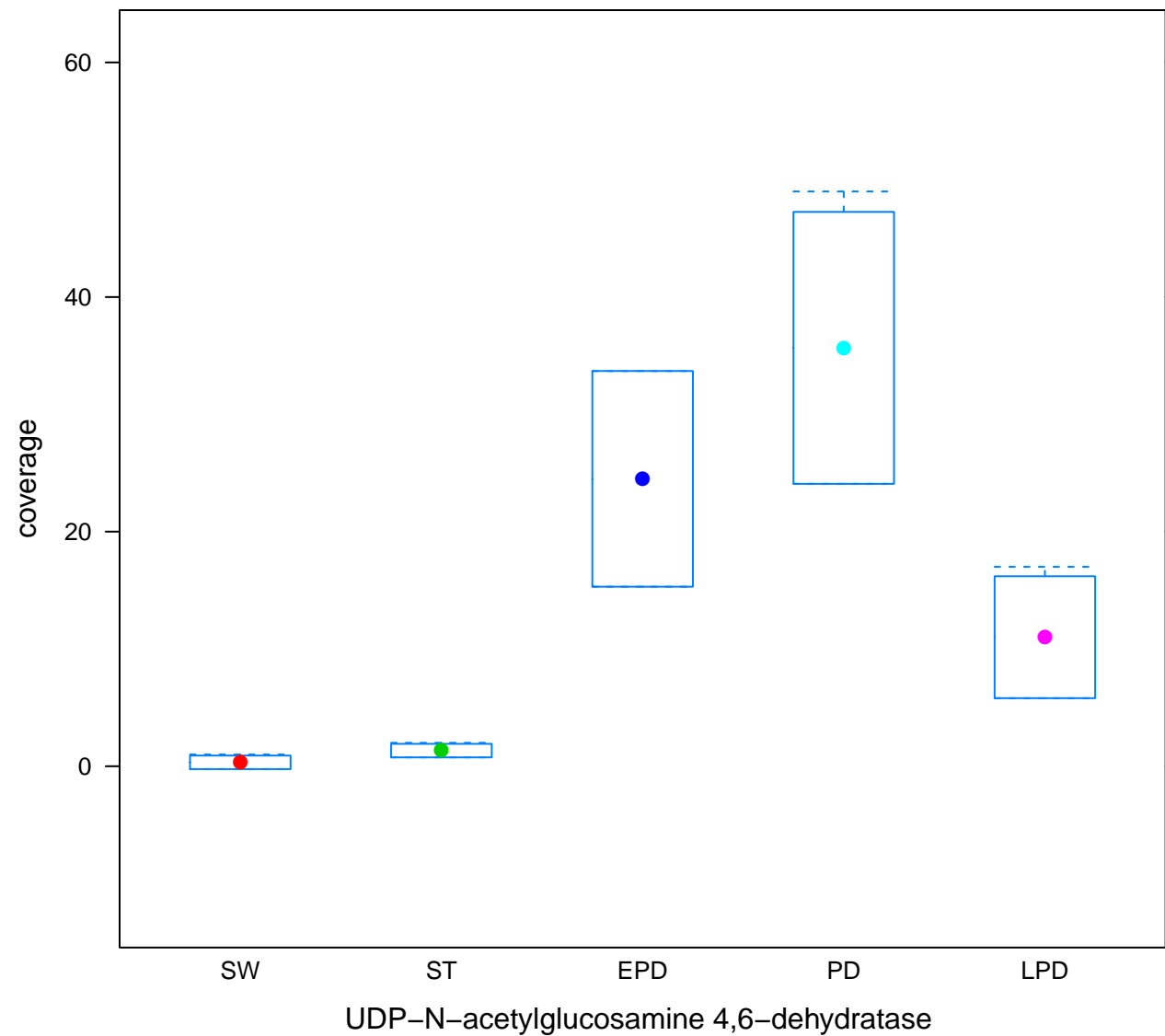

**Fold of change: 71.33**

**baySeq likelihood: 1**

Supplement: Additional file 9: Figure S2 — Expression profiles of all identified CCR genes. [file 1471-2164-14-450-S9.zip › FigureS2/CCNA_00233.pdf]

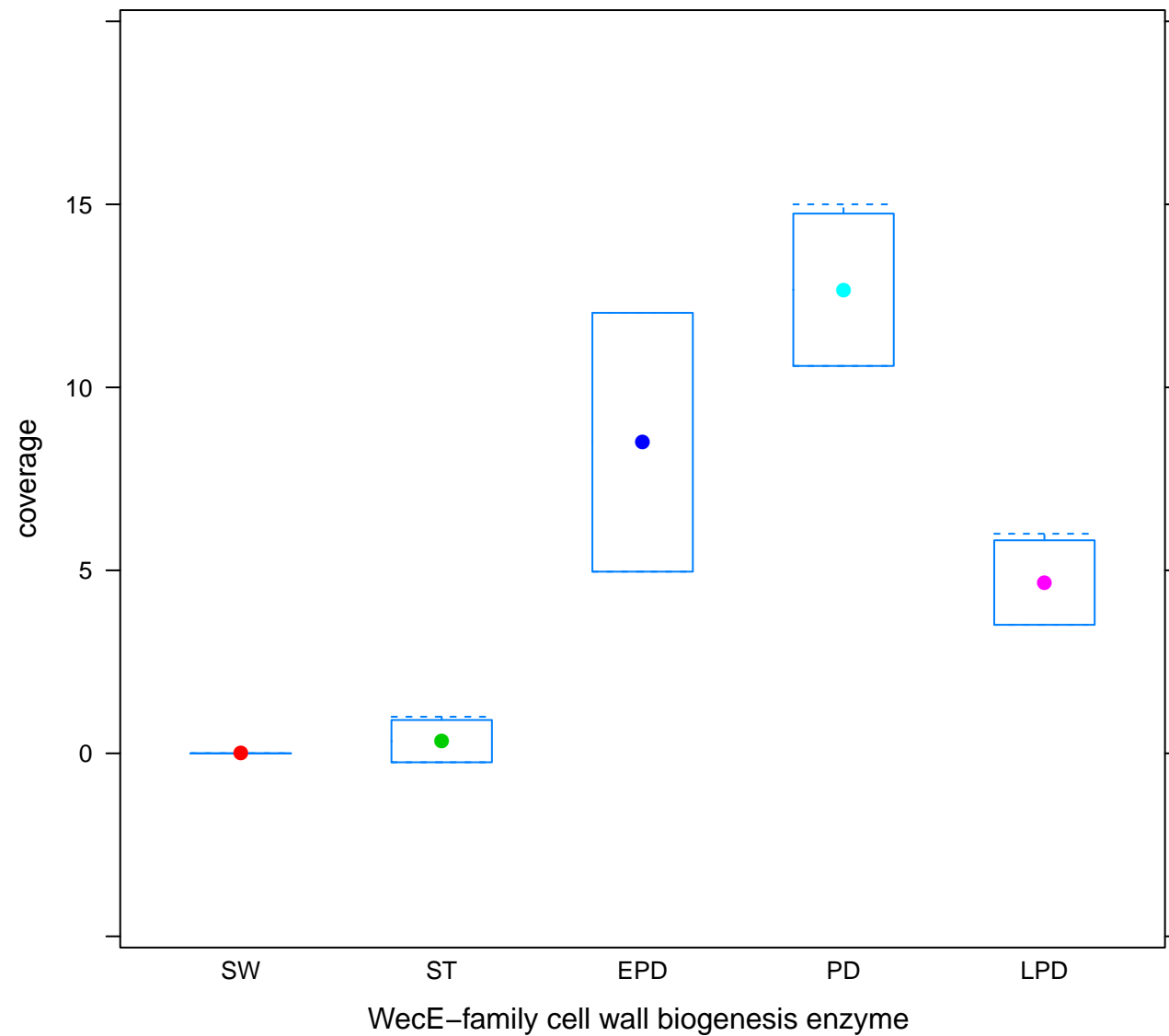

**Fold of change: Inf**  
**baySeq likelihood: 1**

Supplement: Additional file 9: Figure S2 — Expression profiles of all identified CCR genes. [file 1471-2164-14-450-S9.zip › FigureS2/CCNA_00234.pdf]

# CCNA\_00235

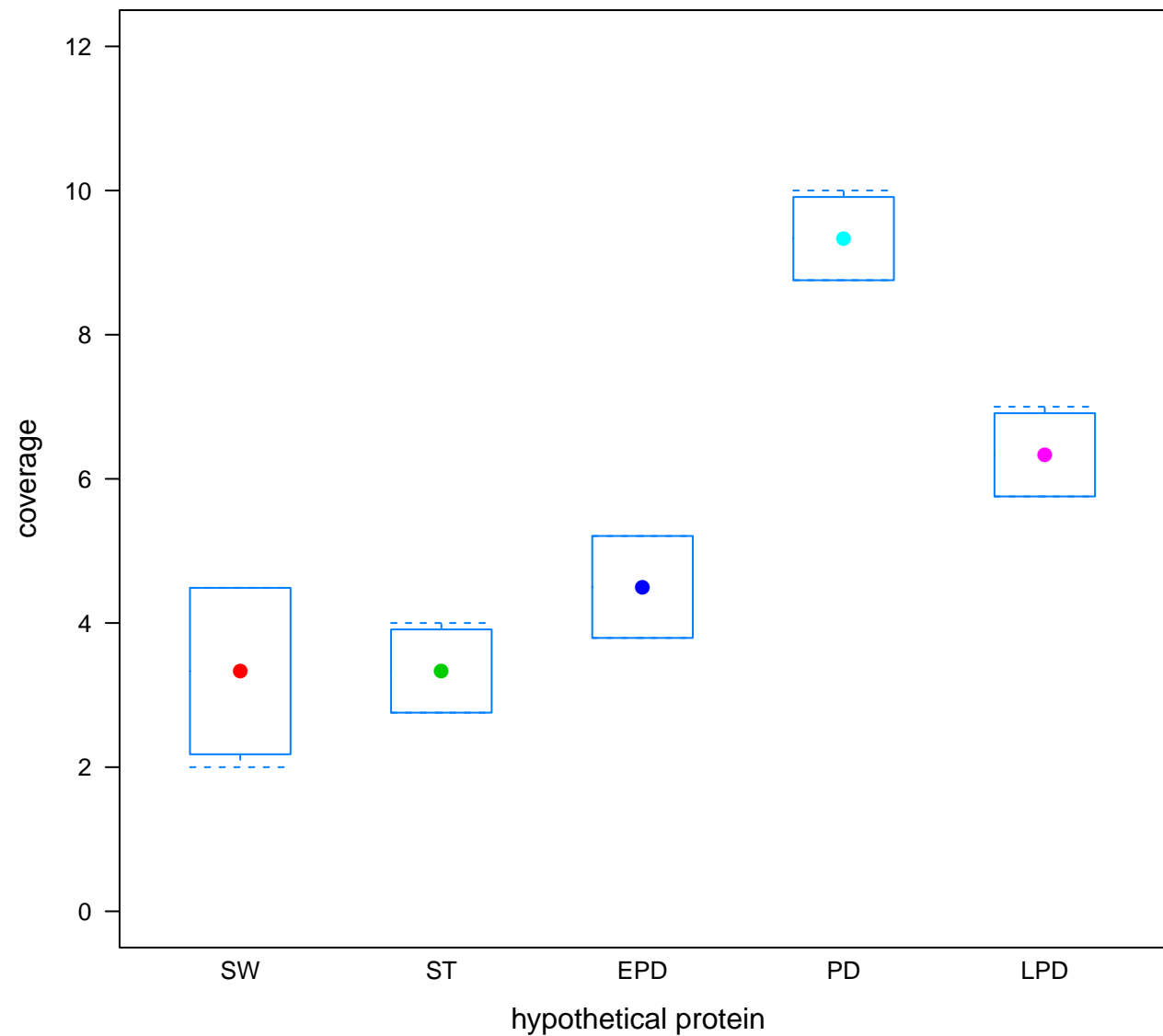

**Fold of change: 3.11**  
**baySeq likelihood: 0.33**

Supplement: Additional file 9: Figure S2 — Expression profiles of all identified CCR genes. [file 1471-2164-14-450-S9.zip › FigureS2/CCNA_00235.pdf]

# CCNA\_00236

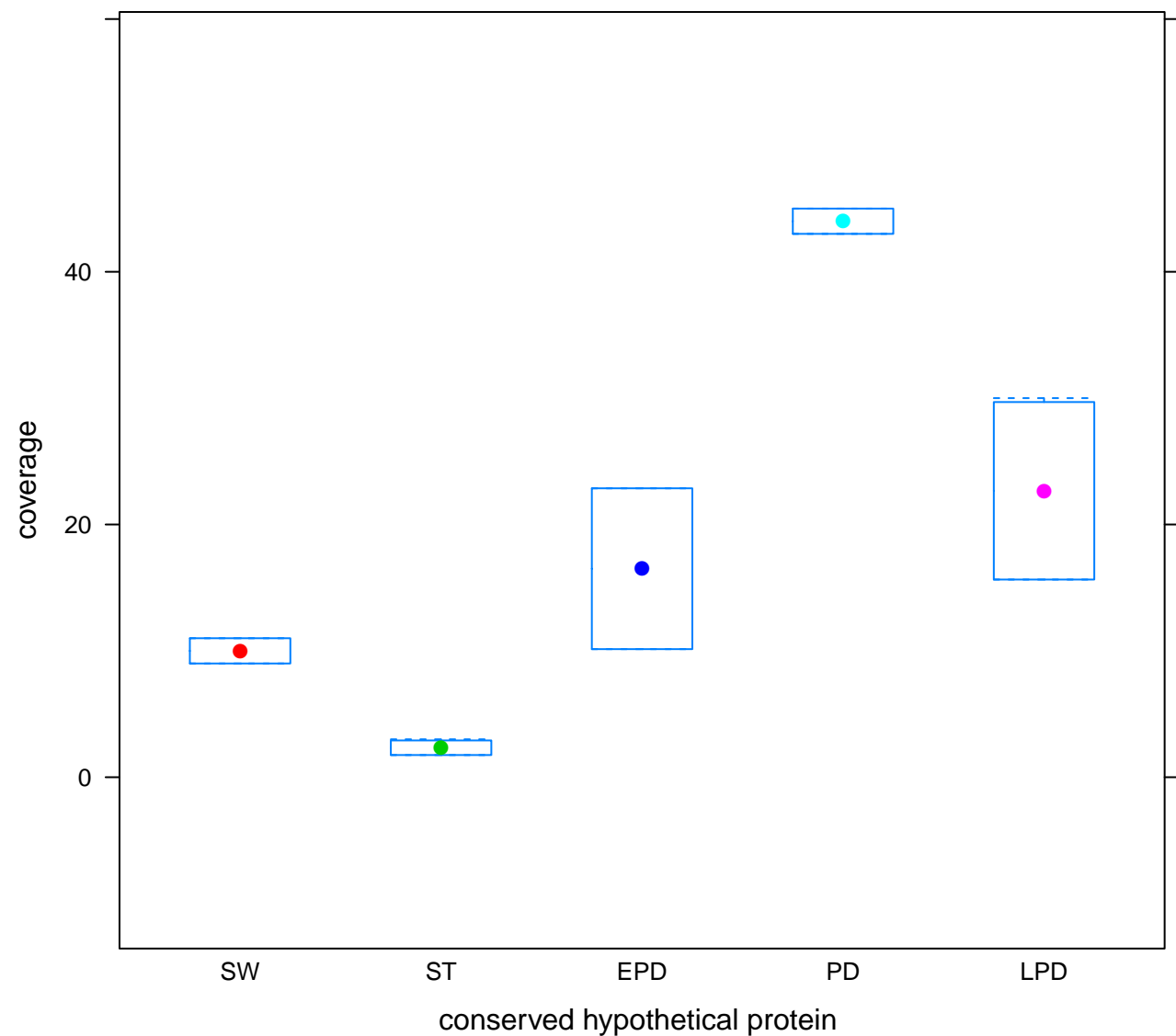

**Fold of change: 17.6**  
**baySeq likelihood: 0.803**

Supplement: Additional file 9: Figure S2 — Expression profiles of all identified CCR genes. [file 1471-2164-14-450-S9.zip › FigureS2/CCNA_00236.pdf]

# CCNA\_00240

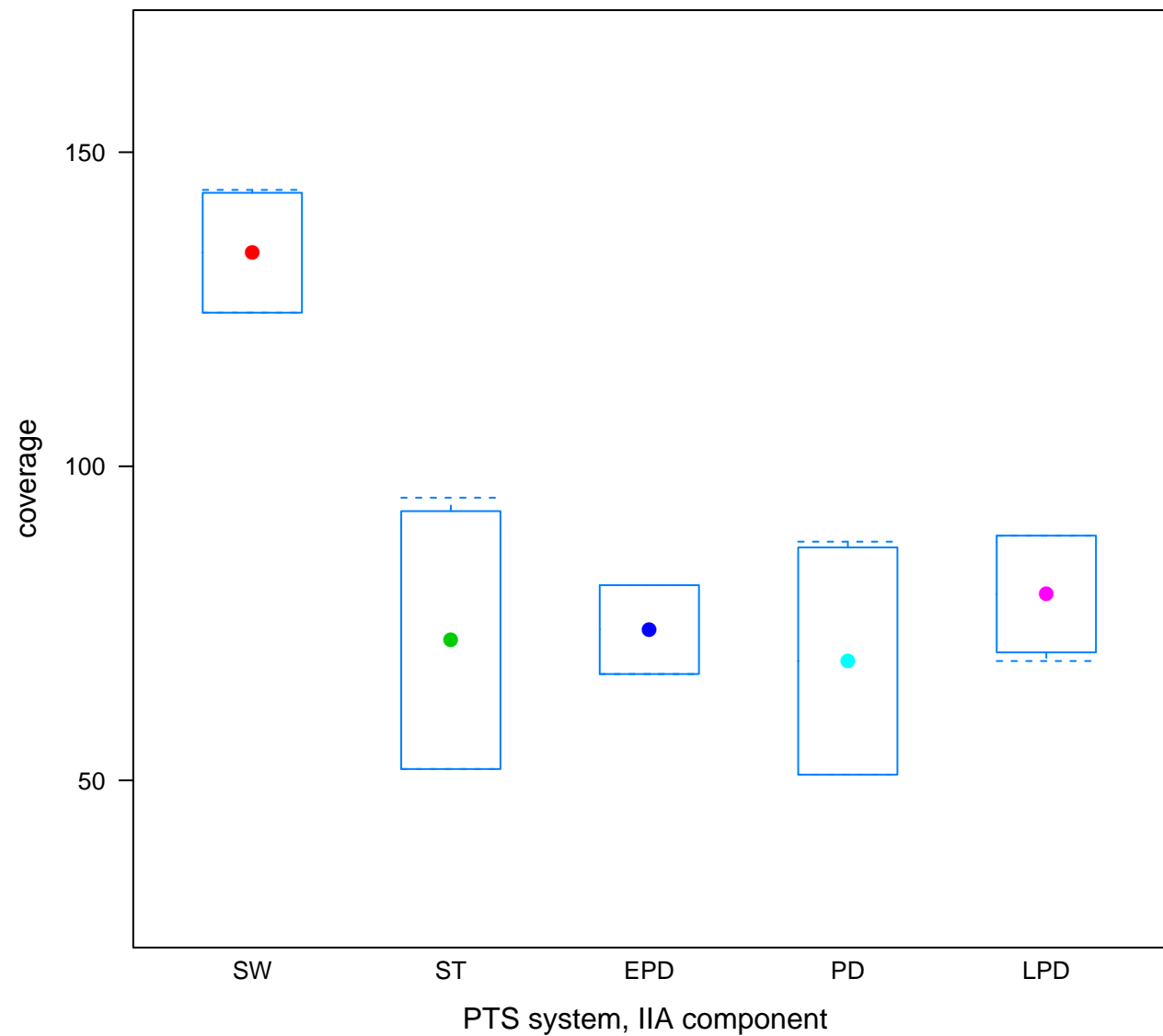

**Fold of change: 2.01**  
**baySeq likelihood: 0.99**

Supplement: Additional file 9: Figure S2 — Expression profiles of all identified CCR genes. [file 1471-2164-14-450-S9.zip › FigureS2/CCNA_00240.pdf]

# CCNA\_00241

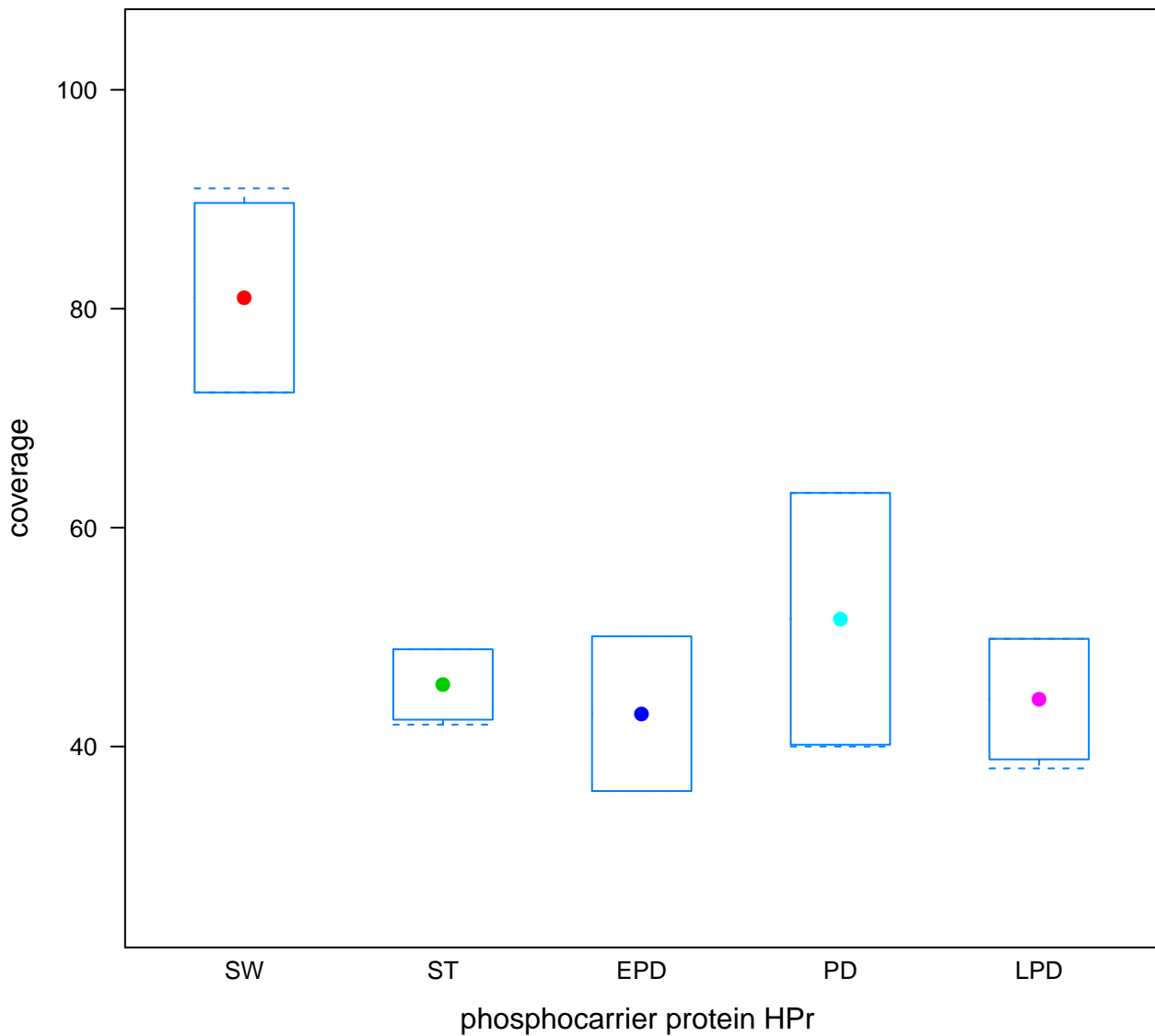

**Fold of change: 1.94**  
**baySeq likelihood: 0.997**

Supplement: Additional file 9: Figure S2 — Expression profiles of all identified CCR genes. [file 1471-2164-14-450-S9.zip › FigureS2/CCNA_00241.pdf]

# CCNA\_00242

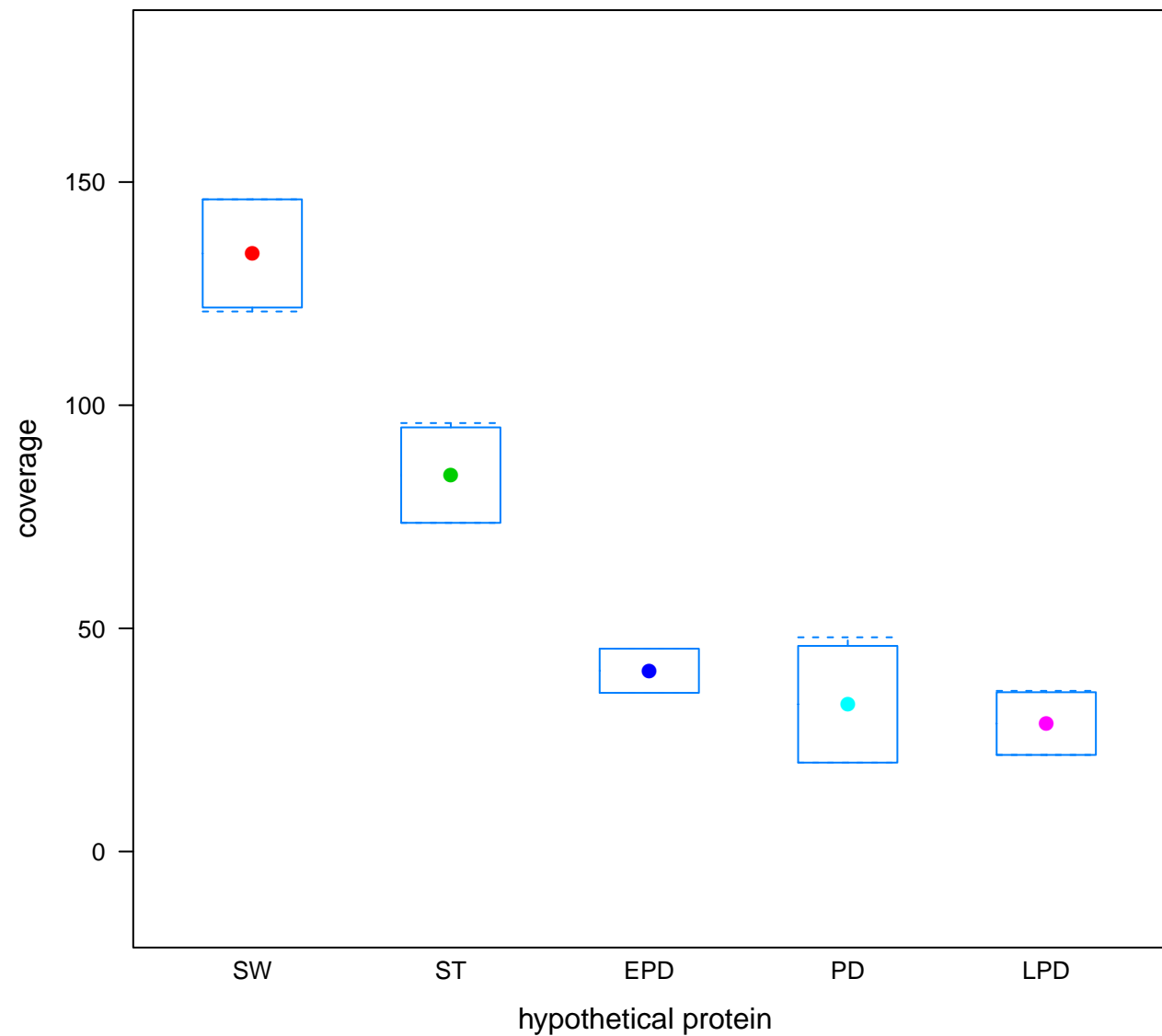

**Fold of change: 4.64**  
**baySeq likelihood: 0.979**

Supplement: Additional file 9: Figure S2 — Expression profiles of all identified CCR genes. [file 1471-2164-14-450-S9.zip › FigureS2/CCNA_00242.pdf]

# CCNA\_00243

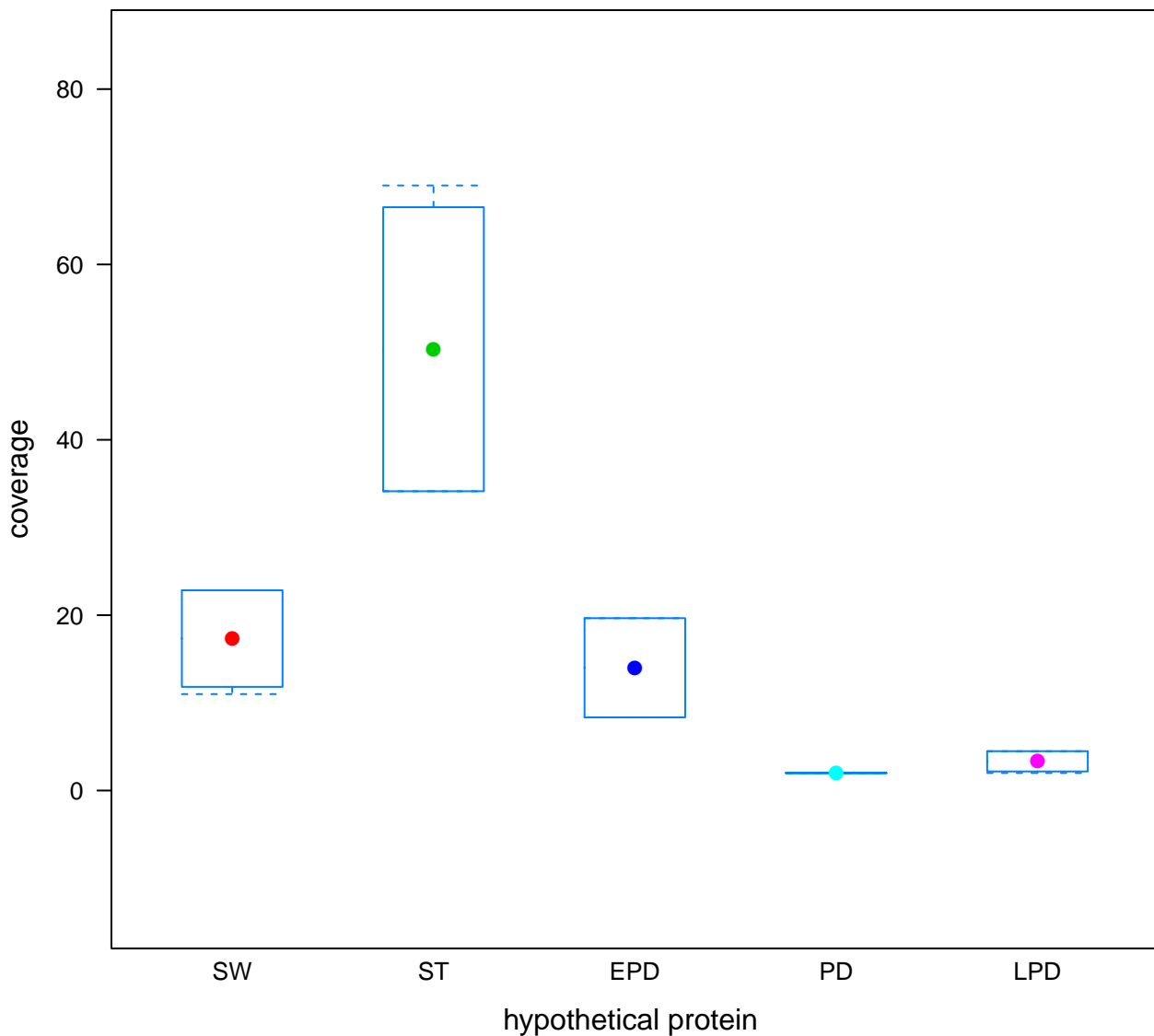

**Fold of change: 27.25**  
**baySeq likelihood: 0.999**

Supplement: Additional file 9: Figure S2 — Expression profiles of all identified CCR genes. [file 1471-2164-14-450-S9.zip › FigureS2/CCNA_00243.pdf]

# CCNA\_00244

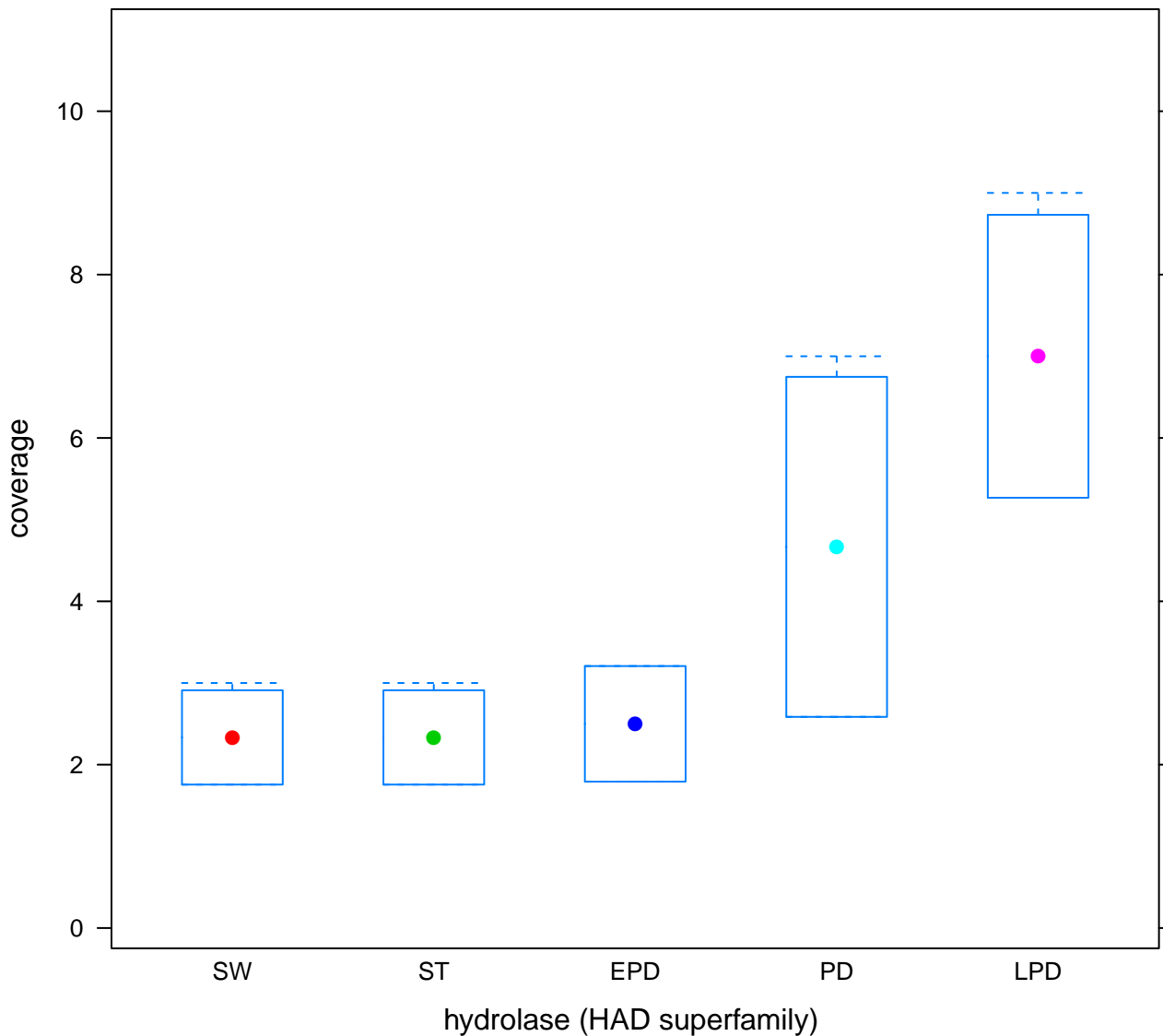

**Fold of change: 3.5**  
**baySeq likelihood: 0.344**

Supplement: Additional file 9: Figure S2 — Expression profiles of all identified CCR genes. [file 1471-2164-14-450-S9.zip › FigureS2/CCNA_00244.pdf]

# CCNA\_00247

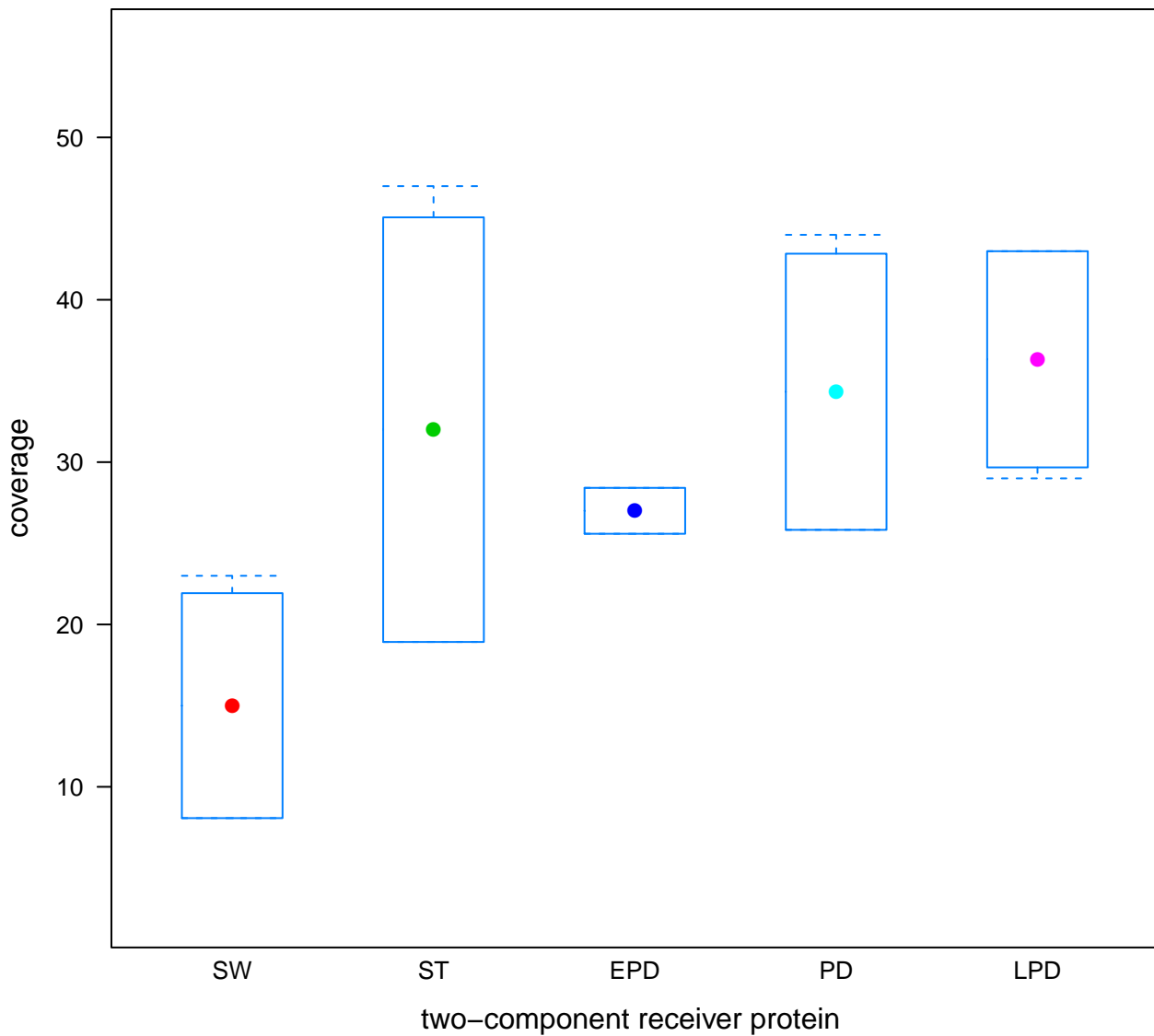

**Fold of change: 3.3**  
**baySeq likelihood: 0.876**

Supplement: Additional file 9: Figure S2 — Expression profiles of all identified CCR genes. [file 1471-2164-14-450-S9.zip › FigureS2/CCNA_00247.pdf]

# CCNA\_00249

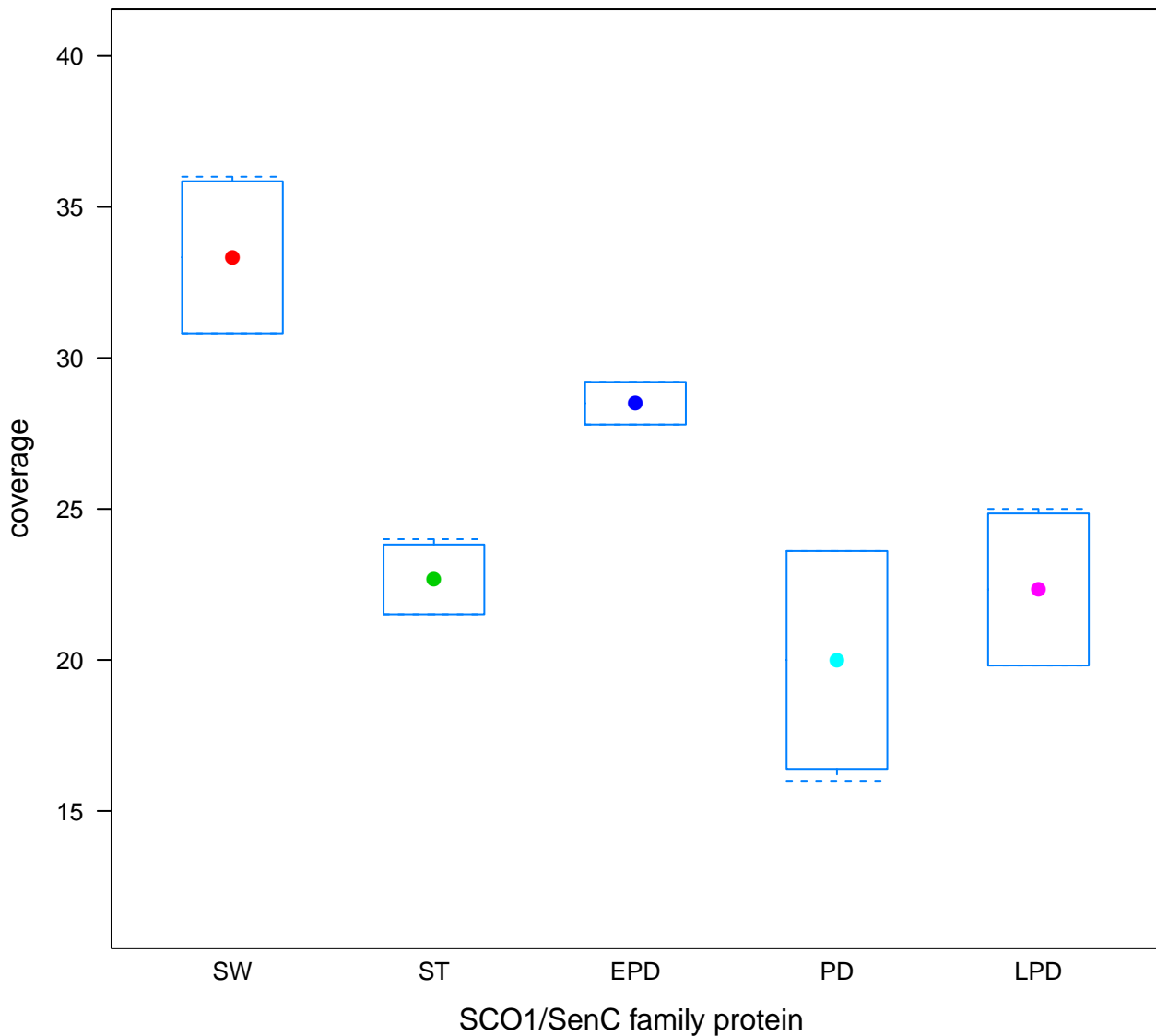

**Fold of change: 1.73**  
**baySeq likelihood: 0.708**

Supplement: Additional file 9: Figure S2 — Expression profiles of all identified CCR genes. [file 1471-2164-14-450-S9.zip › FigureS2/CCNA_00249.pdf]

# CCNA\_00250

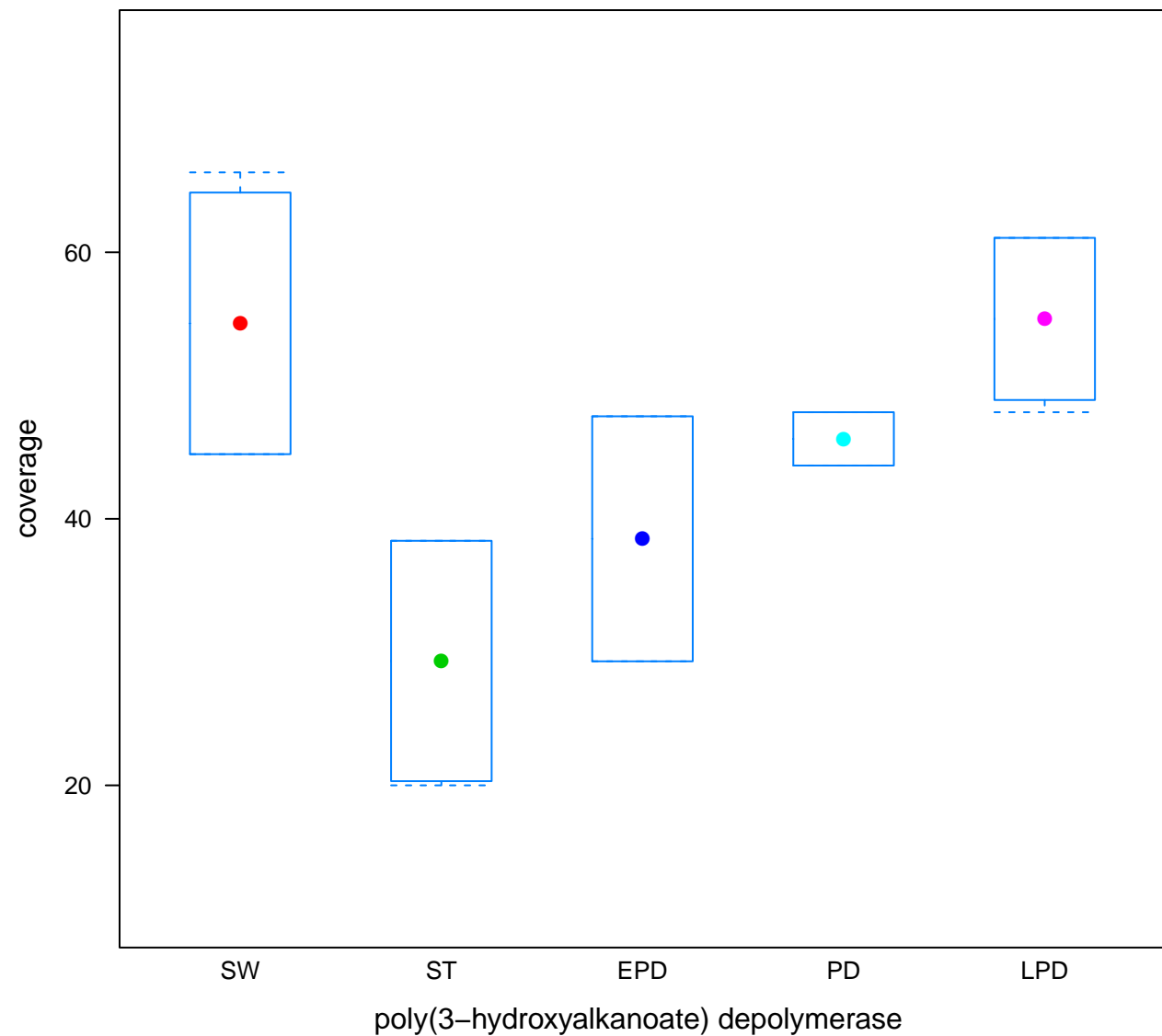

**Fold of change: 2.3**  
**baySeq likelihood: 0.594**

Supplement: Additional file 9: Figure S2 — Expression profiles of all identified CCR genes. [file 1471-2164-14-450-S9.zip › FigureS2/CCNA_00250.pdf]
