# Supplementary material for: Transcriptomic and phylogenetic analysis of a bacterial cell cycle reveals strong associations between gene co-expression and evolution
Source: BMC Genomics. 2013 Jul 5;14:450. doi: 10.1186/1471-2164-14-450 (PMC3829707; doi:10.1186/1471-2164-14-450)
Supplement: Additional file 7 — Additional discussions are in the file of Supplemental Information (SI). Additional files also include supplemental figures S1-S7 with figure legends in file SI. [file 1471-2164-14-450-S7.docx]

**Supplemental information (SI)**

**Transcriptomic and phylogenetic analysis of a bacterial cell cycle reveals strong associations between gene co-expression and evolution**

**Gang Fang ^1, 2^, Karla Passalacqua^3^, Jason Hocking^4^, Paula Montero Llopis^1^, Mark Gerstein^2,5^, Nicholas H. Bergman^3, *^, and Christine Jacobs-Wagner ^1, 4, 6,7^**

1. Department of Molecular, Cellular and Developmental Biology, Yale University, New Haven, CT 06511
2. Department of Molecular Biophysics and Biochemistry, Yale University, New Haven, CT 06511
3. School of Biology, Georgia Institute of Technology, Atlanta, GA 30332
4. Howard Hughes Medical Institute, Yale University, New Haven, CT 06511
5. Program in Computational Biology and Bioinformatics, Yale University, New Haven, CT 06511
6. Department of Microbial Pathogenesis, Yale School of Medicine, New Haven, CT 06511
7. Corresponding author

*Present address: National Biodefense Analysis and Countermeasures Center, Frederick, MD 21702

**1. Potential noise introduced by method-induced genes.**

1.1 Identification of Potential Method-Induced (PMI) genes

We acknowledge that some aspects of the synchronization technique may result in the differential expression of some genes, independently of the cell cycle. For example, the cell cycle synchronization includes a cold shock due to centrifugations and washes at 4°C. Additionally, the washes were done in M2 buffer, which differs from M2G, notably by lacking glucose (to prevent cell growth during the synchronization process). Genes whose transcription is induced by the synchronization method (e.g., cold shock, absence of carbon source) are expected to be up-regulated in the SW (G1) cell stage (t= 0min following synchronization) with a lower expression profile in subsequent time point samples. Hence the genes fitted into the baySeq “up-down-down-down-down” model, which totals 410 genes, may include PMI-induced genes, in addition of bona fide SW-specific genes. Table S9 lists these genes with baySeq likelihood.

1.2 Comparison of PMI and other CCR genes in terms of maximum expression and fold of expression changes.

|  | PMI | Other CCR | KS test |
| --- | --- | --- | --- |
| Maximum expression (mean) | 71 | 195 | p=0.005 |
| Fold of changes (mean) | 4.5 | 9.4 | P<1e-16 |

PMI genes had lower peak expression than other CCR genes, and fold of changes were smaller than other CCR genes.

1.3 GO terms

Among the 1024 annotated CCR genes, 541 were related to primary and cellular metabolic processes, and 175 of them were PMI. Out of 410 PMI genes, 283 had assigned GO terms. Fisher’s exact test indicated that there is no difference between the two groups (p=0.224).

1.4 Gene persistence and co-expression module contribution

Eighty-one PMI genes had PI≥150. After removing all 410 PMI genes, we obtained 95 CCR genes with a PI≥150, giving an average module contribution of 3.1. The average contribution of the remaining CCR genes is 2.98 (KS test p=0.007 and t test p<1e-5). Thus, persistent genes retain a bigger contribution in co-expression modules even when PMI genes are excluded from the analysis.

1.5 PMI WGCNA modules distribution.

| Module ID | PMI counts | Module Size | PMI (%) | MPD-MNTD coords | | |
| --- | --- | --- | --- | --- | --- | --- |
|  |  |  |  | MPD | MNTD | Quadrant |
| skyblue1 | 8 | 8 | 100 | -3.71 | -2.58 | III |
| white | 21 | 22 | 95 | -2.47 | -3.17 | III |
| turquoise | 100 | 107 | 93 | -3.88 | -2.28 | III |
| darkslateblue | 11 | 12 | 92 | -0.79 | 1.01 | I |
| blue | 84 | 93 | 90 | -2.01 | -1.56 | II |
| darkorange | 19 | 22 | 86 | 0.33 | -1.22 | I |
| saddlebrown | 18 | 21 | 86 | -3.20 | -2.20 | III |
| violet | 16 | 19 | 84 | -6.38 | -1.46 | II |
| yellow | 45 | 58 | 78 | -1.58 | -2.03 | IV |
| antiquewhite4 | 6 | 9 | 67 | -6.15 | -1.60 | II |
| darkred | 15 | 25 | 60 | -6.31 | -2.62 | III |
| lightsteelblue | 4 | 7 | 57 | -2.07 | -1.47 | II |
| brown | 33 | 59 | 56 | -2.77 | -2.35 | III |
| maroon | 4 | 11 | 36 | -4.62 | -2.87 | III |
| plum1 | 3 | 16 | 19 | -3.65 | -2.73 | III |
| sienna3 | 3 | 18 | 17 | -5.01 | -0.85 | II |
| floralwhite | 2 | 13 | 15 | -2.05 | -0.40 | II |
| orangered3 | 1 | 7 | 14 | -1.58 | -3.11 | IV |
| orangered4 | 2 | 16 | 13 | -2.91 | -0.96 | II |

As summarized in the above table, 395 PMI genes were found in 19 modules, and the top 13 modules account for 96% (380) of all assigned PMI genes. In total, we had 16 PMI modules in Quadrant II and III, and 2 in each of Quadrant I and IV. On the other hand, we had 49 modules out of the 76 assigned into Quadrant II and III, 11 modules in Quadrant IV, and 16 in Quadrant I. Chi-square and Fisher’s exact tests indicate no difference between PMI and other modules in terms of their evolutionary profiles.

**2. Less stringent gene homolog counts**

See Table S10.

**3. Legends of supplementary figures**

**Figure S1. Frequency distribution of gene expression values**

The frequency distributions of gene expressions for each of the 5 cell cycle time points are shown on the left. The corresponding gene expressions are fitted into power-law distributions. Cumulative distributions with fitted –α+1 values are shown on the right (an example is shown in Figure 2D).

**Figure S2. Expression profiles of all identified CCR genes**

This directory contains the expression profile of the 1586 identified CCR genes during the cell cycle (red, SW; green, ST; dark blue, EPD; light blue, PD; and purple, LPD).

**Figure S3. Directed acyclic graph (DAG) of over- and under-represented gene ontology (GO) terms in CCR genes**

Three DAGs are plotted for the GO term “molecular function”, “biological process” and “cellular component”. Orange (cyan) box indicates that the term is over-represented (under-represented) in CCR genes. The text version of the Fisher’s exact test results is provided in Table S5.

**Figure S4. Co-expression network topologies of all 76 modules**

This is a directory containing the network topologies of all 76 modules (magenta is used as the example in Figure 6A) and a text file Eigen_varExplained.txt that lists the variance explained by 1st eigenvector for each module.

**Figure S5. Module expression profile represented by its 1st eigenvector**

The 1st eigenvector (1st principle component) of each module’s expression matrix (columns are the 5 cell cycle time points, each with 3 replicates; and rows are the expression values of member genes) is used to represent the expression profile of a module. Biological replicates are binned to boxplots that indicate the expression at 5 different cell cycle time points, namely SW, ST, EPD, PD and LPD.

**Figure S6. Phylogenetic profiles and positions in MPD and MNTD coordinates for all modules**

This is a directory containing 77 files, which include the phylogenetic profiles of all 76 modules (examples are in Figure 8B) and a MM_Coordinates.pdf, which is an enlarged and texted version of Figure 8A (right panel) in which modules are plotted according to their positions in MPD and MNTD coordinates.

**Figure S7. Persistent index distributions**

The PI distributions of the CCR genes and of all genes show no difference (t-test p-value is 0.37).
