# Supplementary figures and images for: Transcriptomic and phylogenetic analysis of a bacterial cell cycle reveals strong associations between gene co-expression and evolution (part 3 of 4)
Source: BMC Genomics. 2013 Jul 5;14:450. doi: 10.1186/1471-2164-14-450 (PMC3829707; doi:10.1186/1471-2164-14-450)

# CCNA\_00495

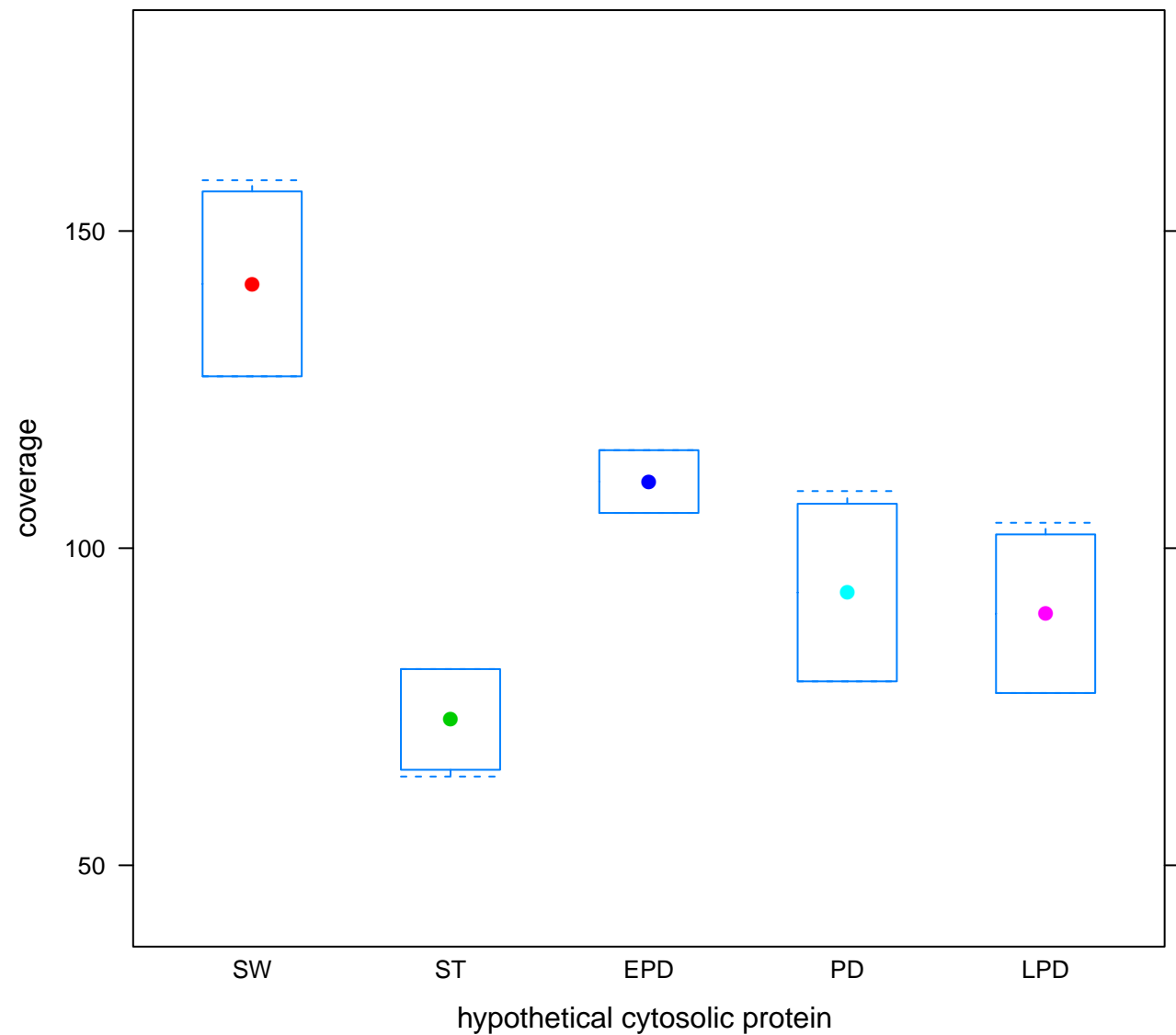

**Fold of change: 2.06**  
**baySeq likelihood: 0.833**

Supplement: Additional file 9: Figure S2 — Expression profiles of all identified CCR genes. [file 1471-2164-14-450-S9.zip › FigureS2/CCNA_00495.pdf]

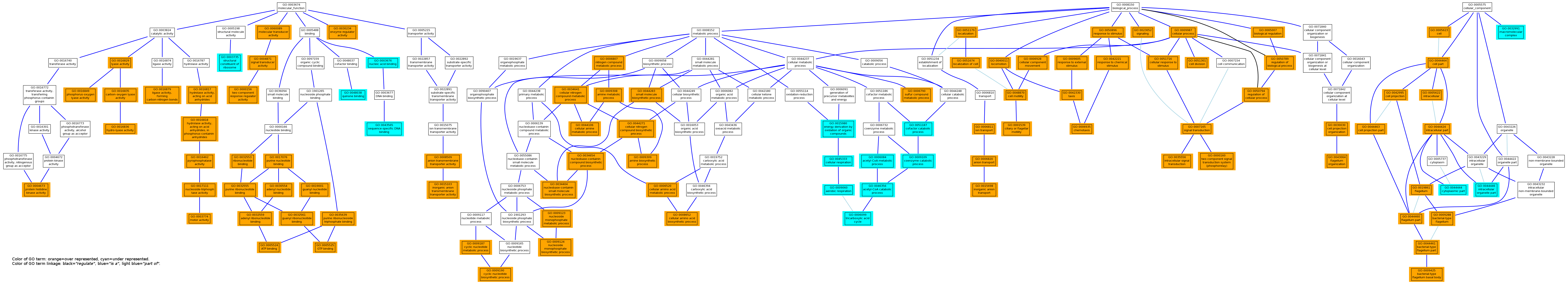

Supplement: Additional file 11: Figure S3 — Directed acyclic graph (DAG) of over- and under-represented gene ontology (GO) terms in CCR genes. [file 1471-2164-14-450-S11.png]

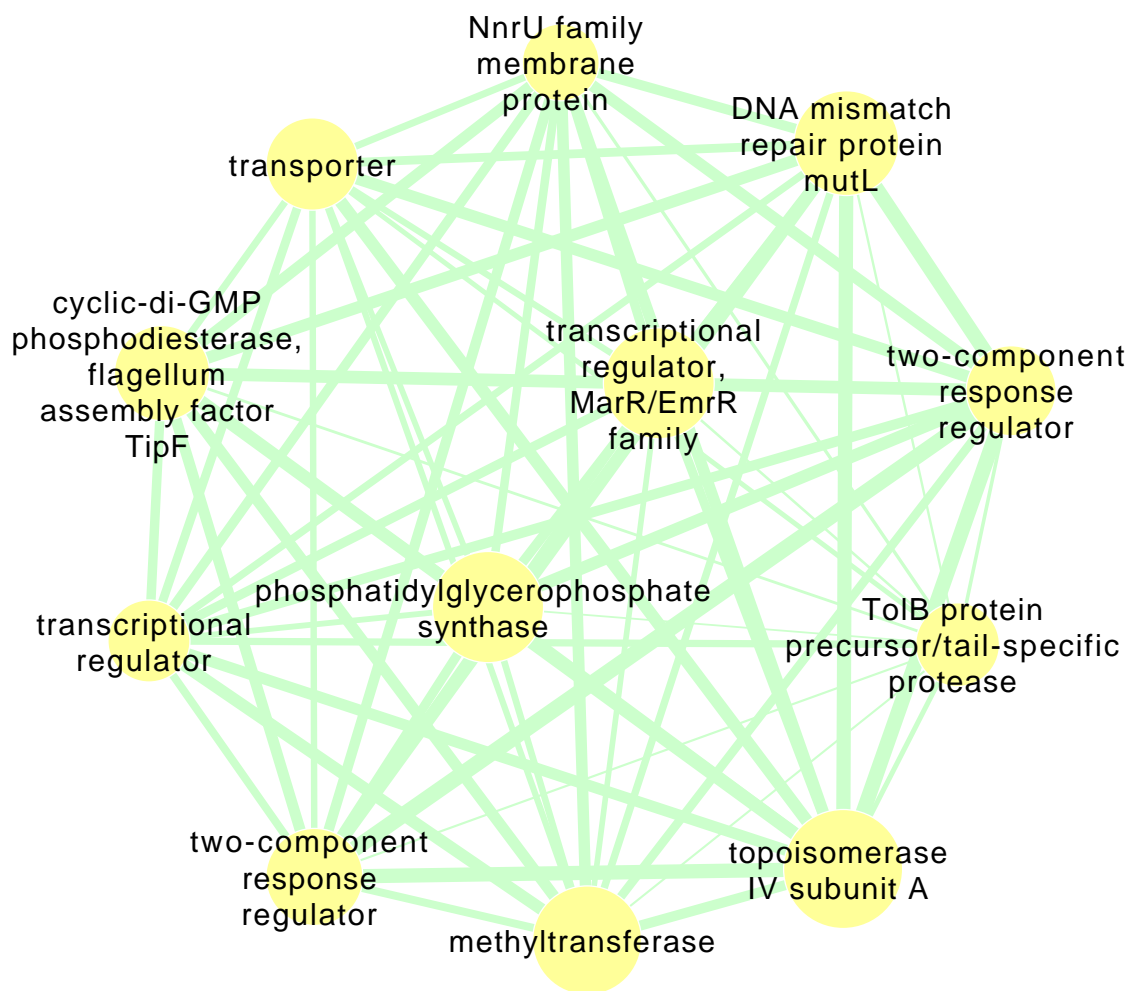

Supplement: Additional file 13: Figure S4 — Co-expression network topologies of all 76 modules. [file 1471-2164-14-450-S13.zip › FigureS4/bisque4.pdf]

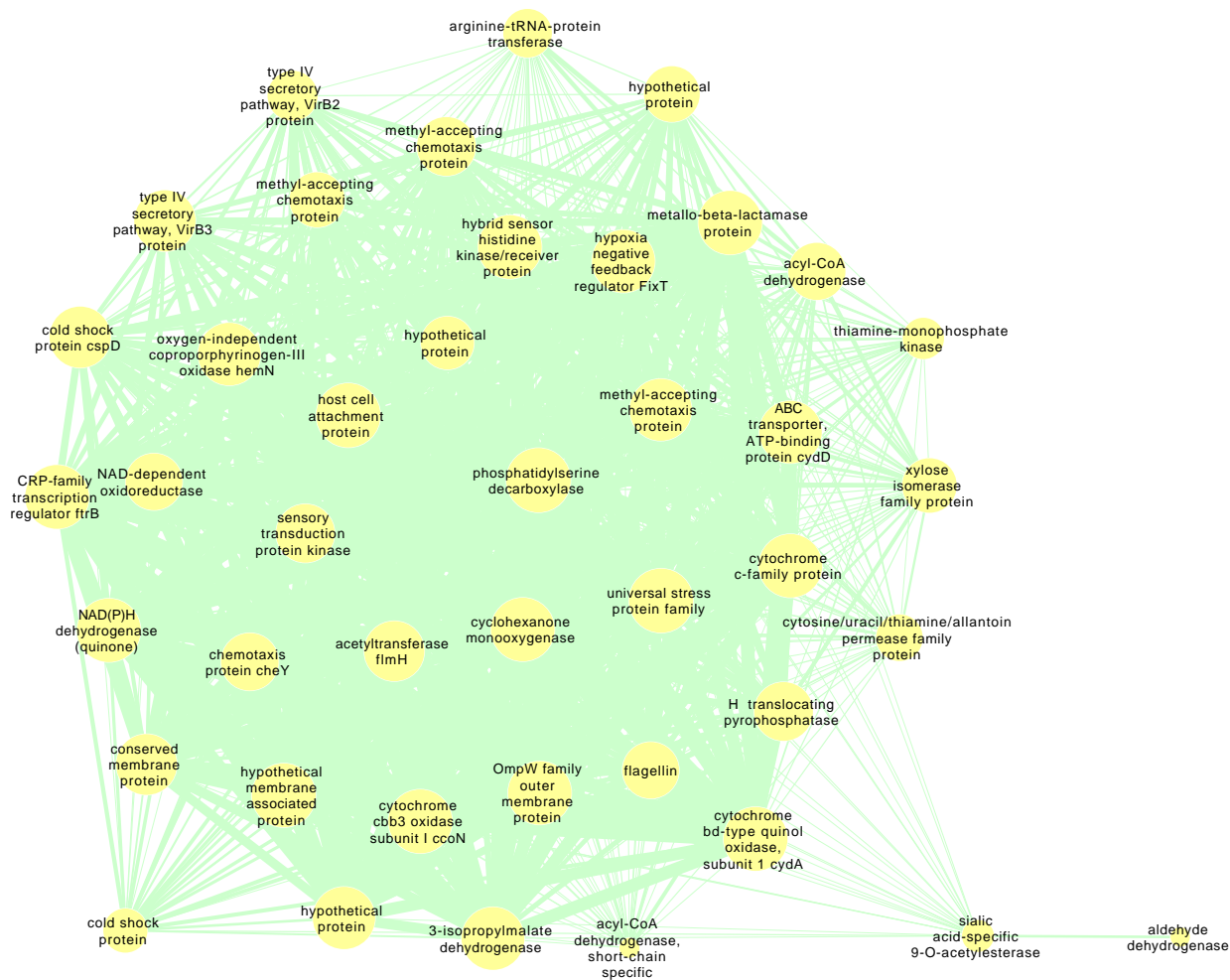

Supplement: Additional file 13: Figure S4 — Co-expression network topologies of all 76 modules. [file 1471-2164-14-450-S13.zip › FigureS4/black.pdf]

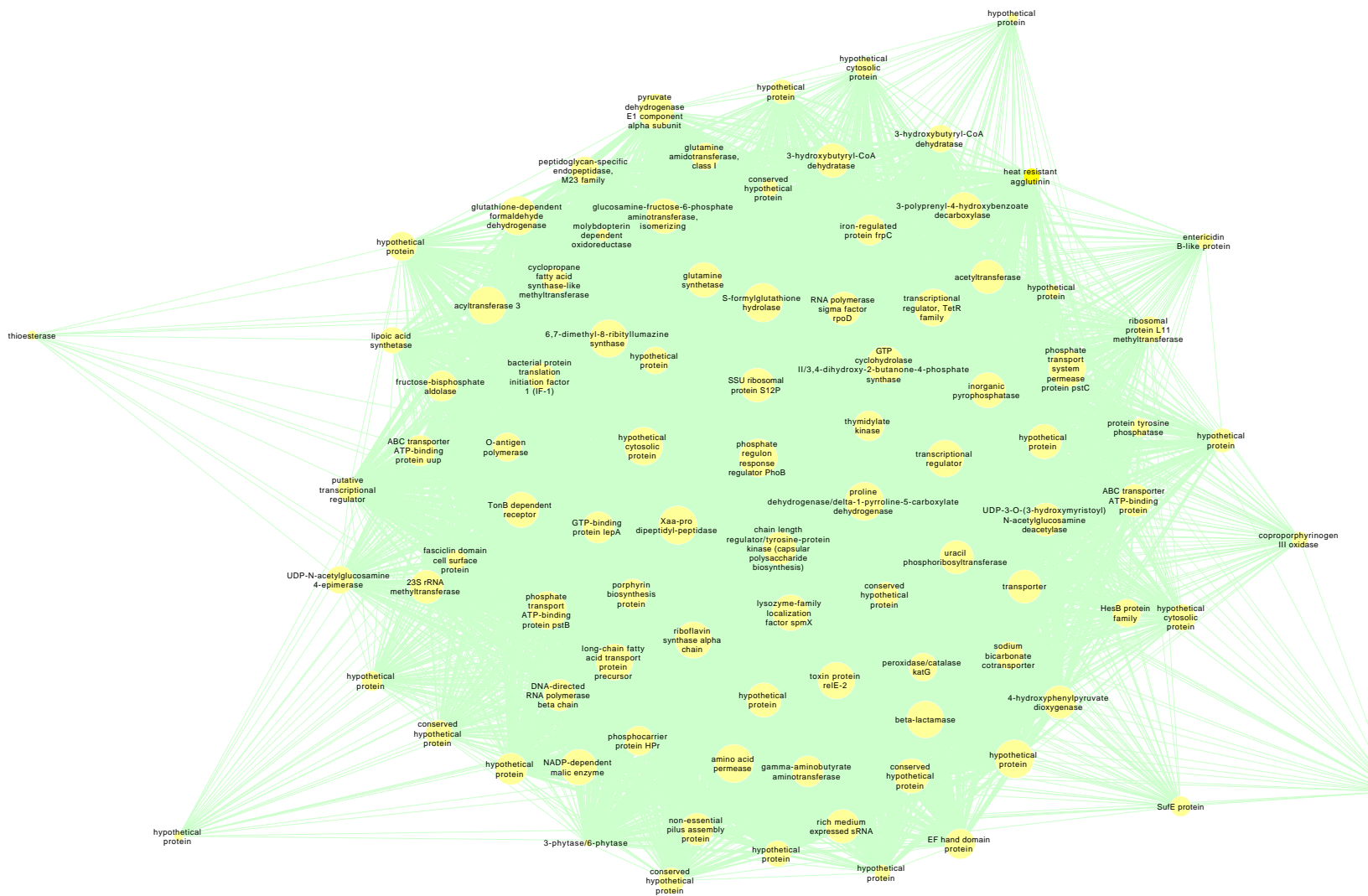

Supplement: Additional file 13: Figure S4 — Co-expression network topologies of all 76 modules. [file 1471-2164-14-450-S13.zip › FigureS4/blue.pdf]

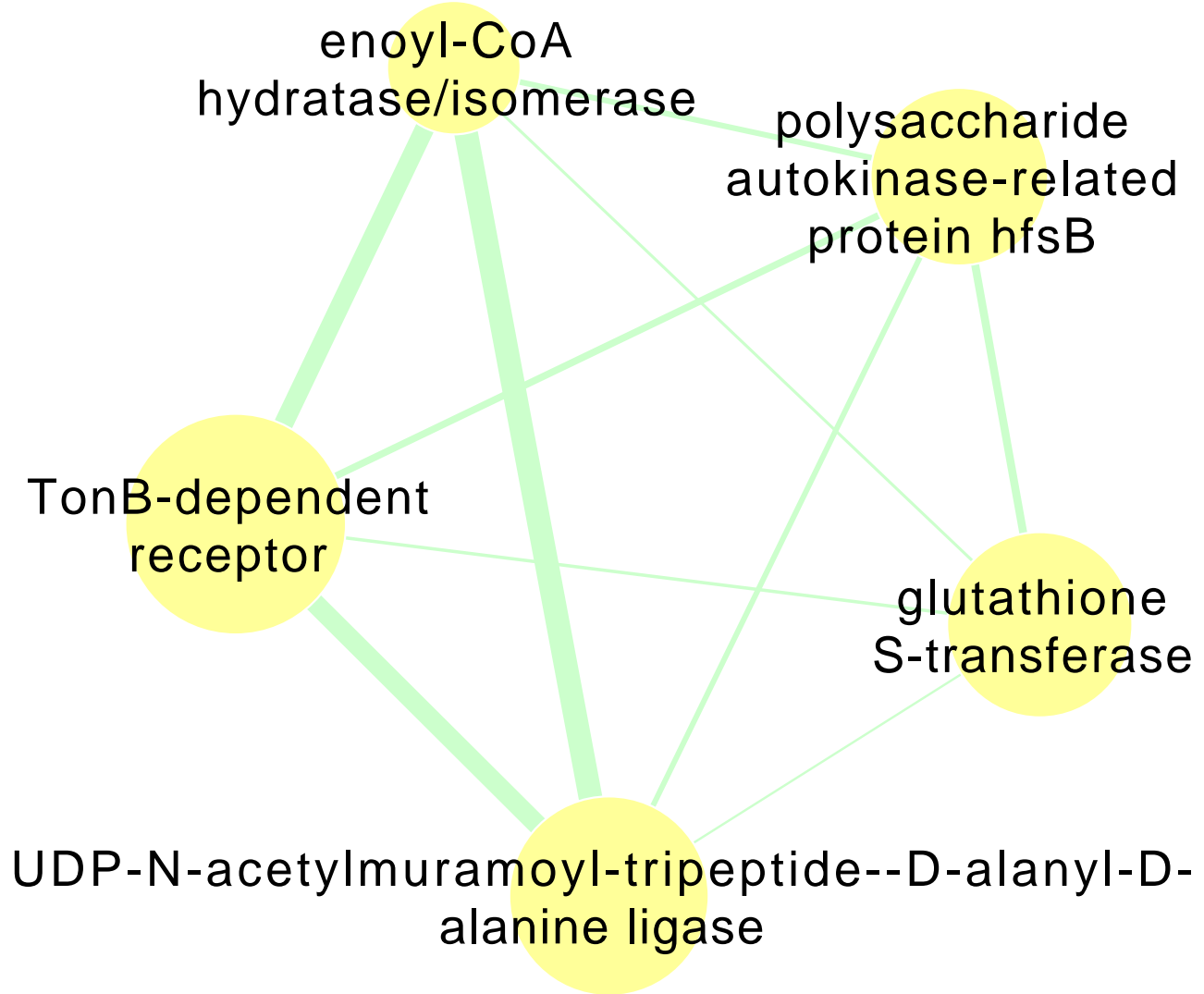

Supplement: Additional file 13: Figure S4 — Co-expression network topologies of all 76 modules. [file 1471-2164-14-450-S13.zip › FigureS4/blue2.pdf]

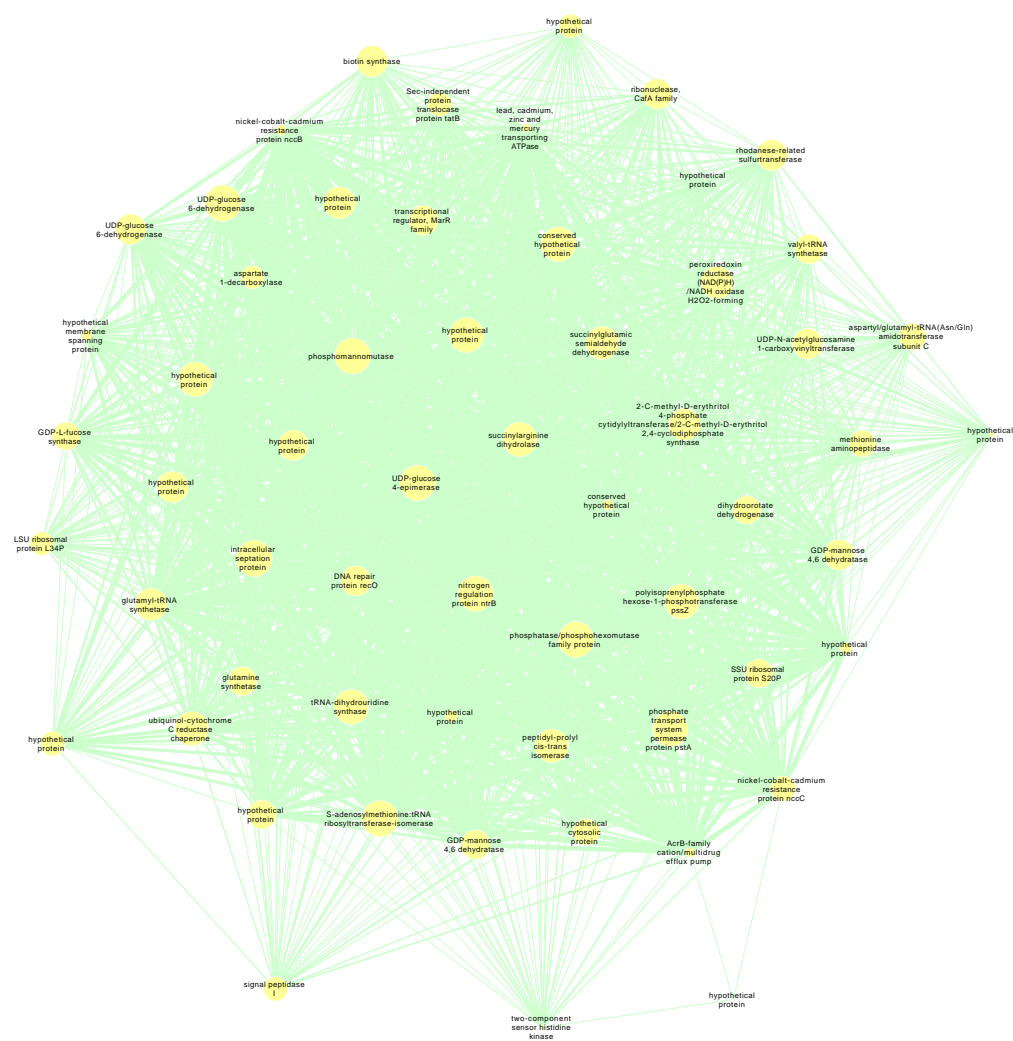

Supplement: Additional file 13: Figure S4 — Co-expression network topologies of all 76 modules. [file 1471-2164-14-450-S13.zip › FigureS4/brown.pdf]

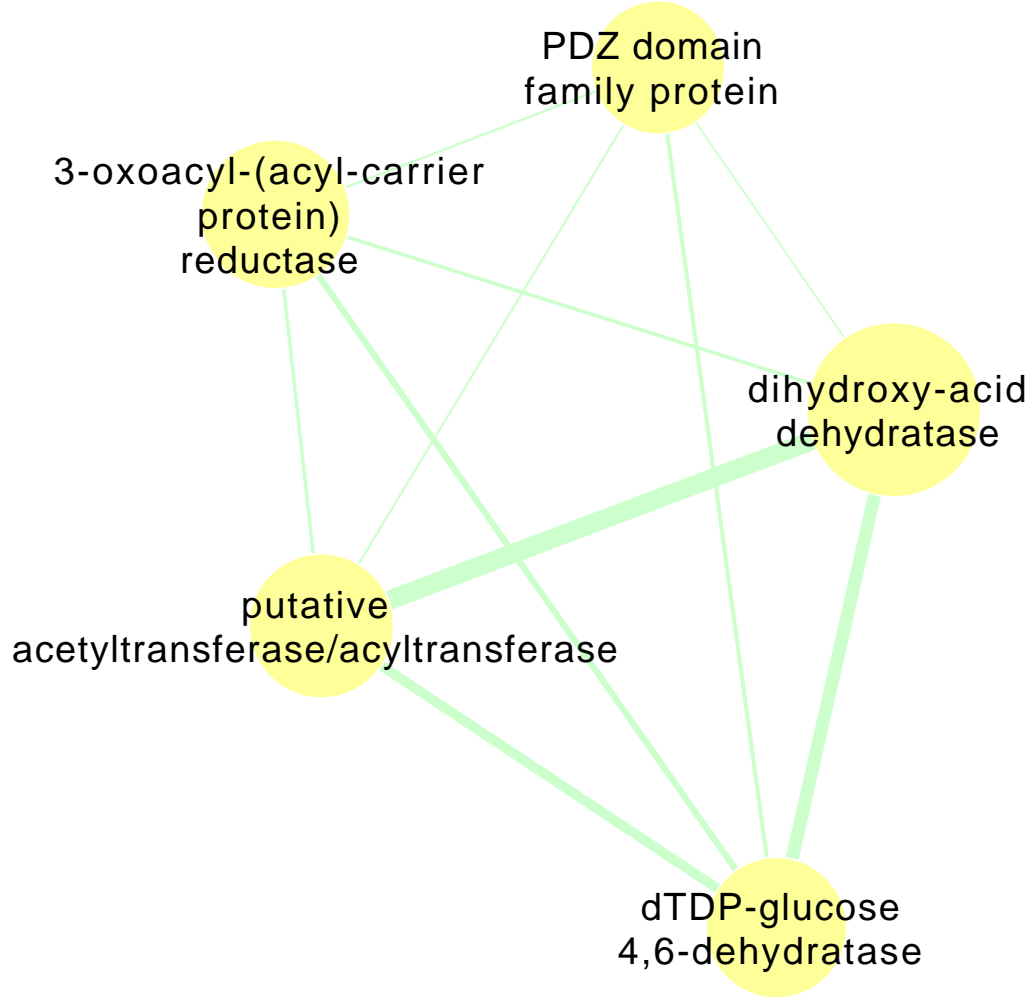

Supplement: Additional file 13: Figure S4 — Co-expression network topologies of all 76 modules. [file 1471-2164-14-450-S13.zip › FigureS4/brown2.pdf]

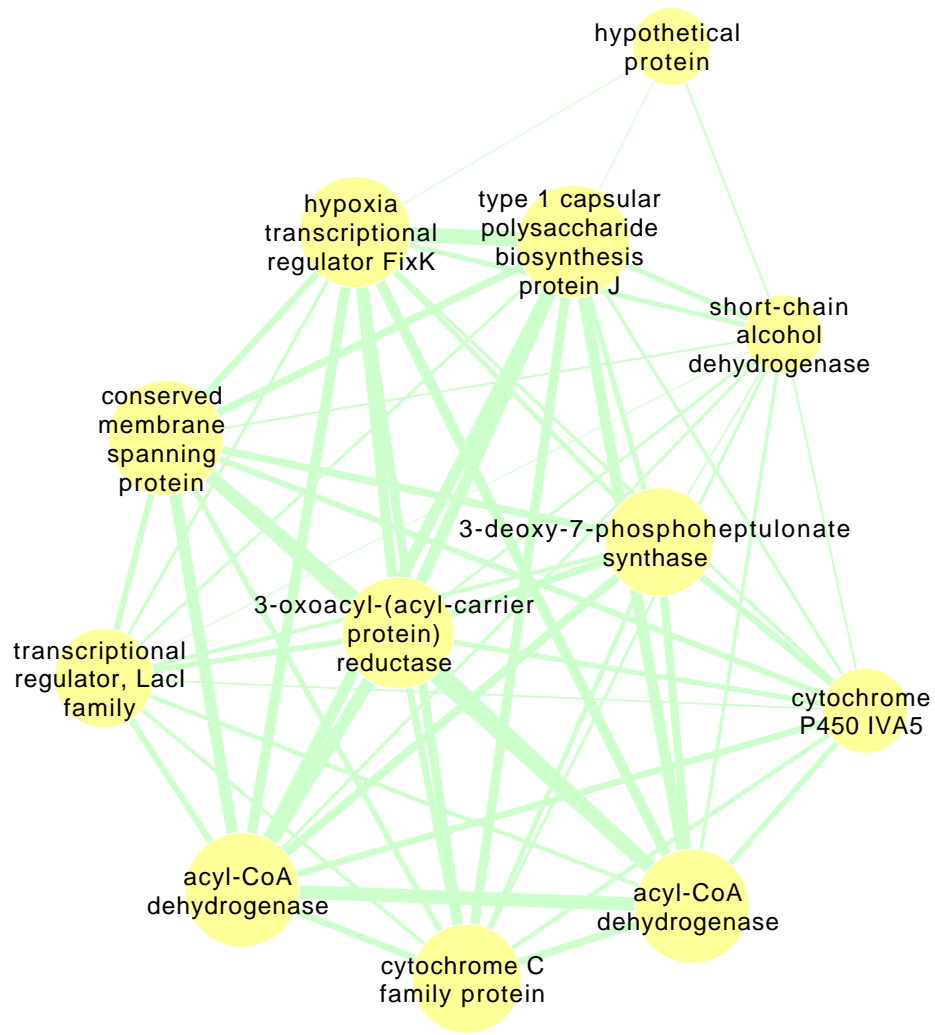

Supplement: Additional file 13: Figure S4 — Co-expression network topologies of all 76 modules. [file 1471-2164-14-450-S13.zip › FigureS4/brown4.pdf]

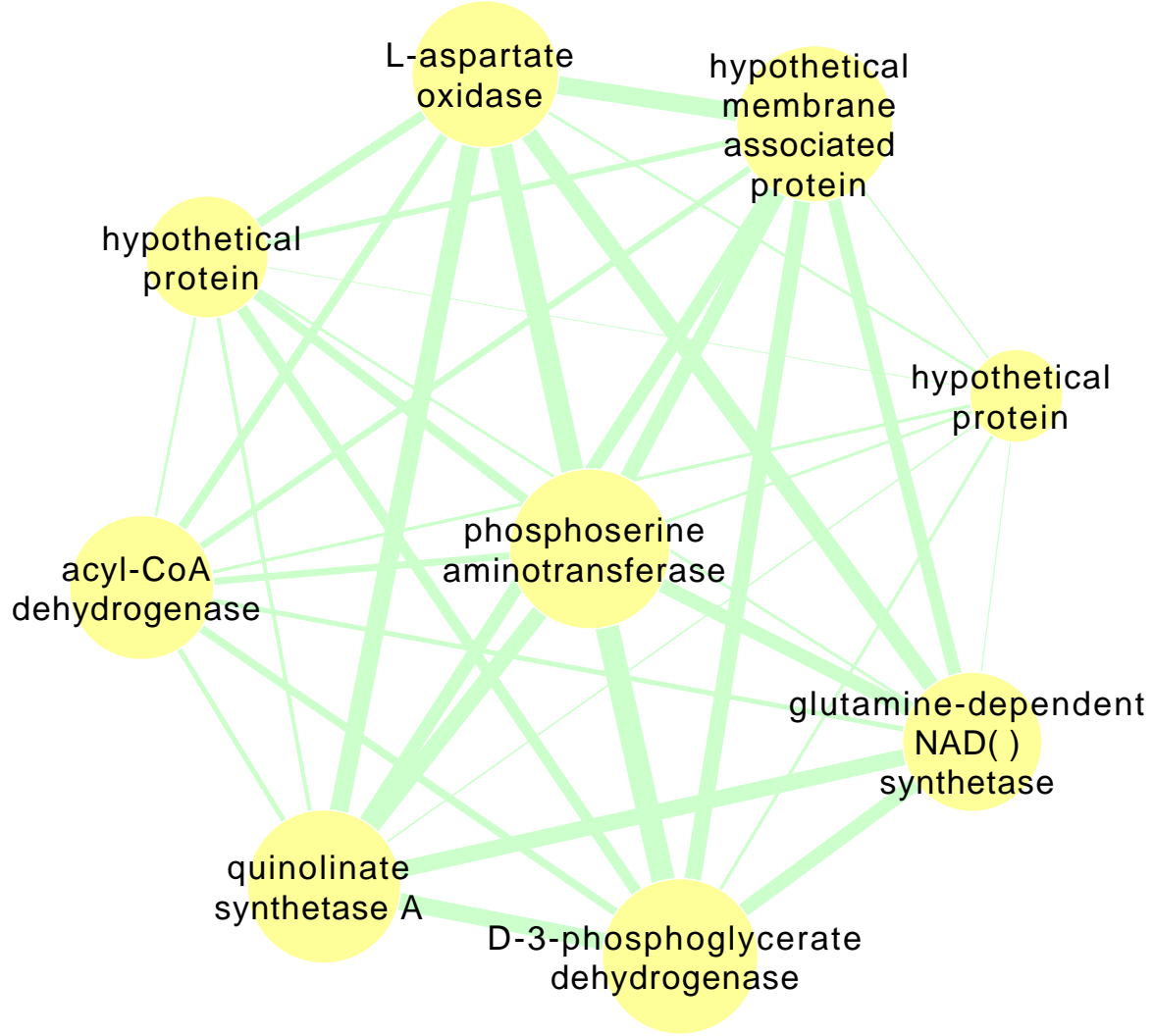

Supplement: Additional file 13: Figure S4 — Co-expression network topologies of all 76 modules. [file 1471-2164-14-450-S13.zip › FigureS4/coral1.pdf]

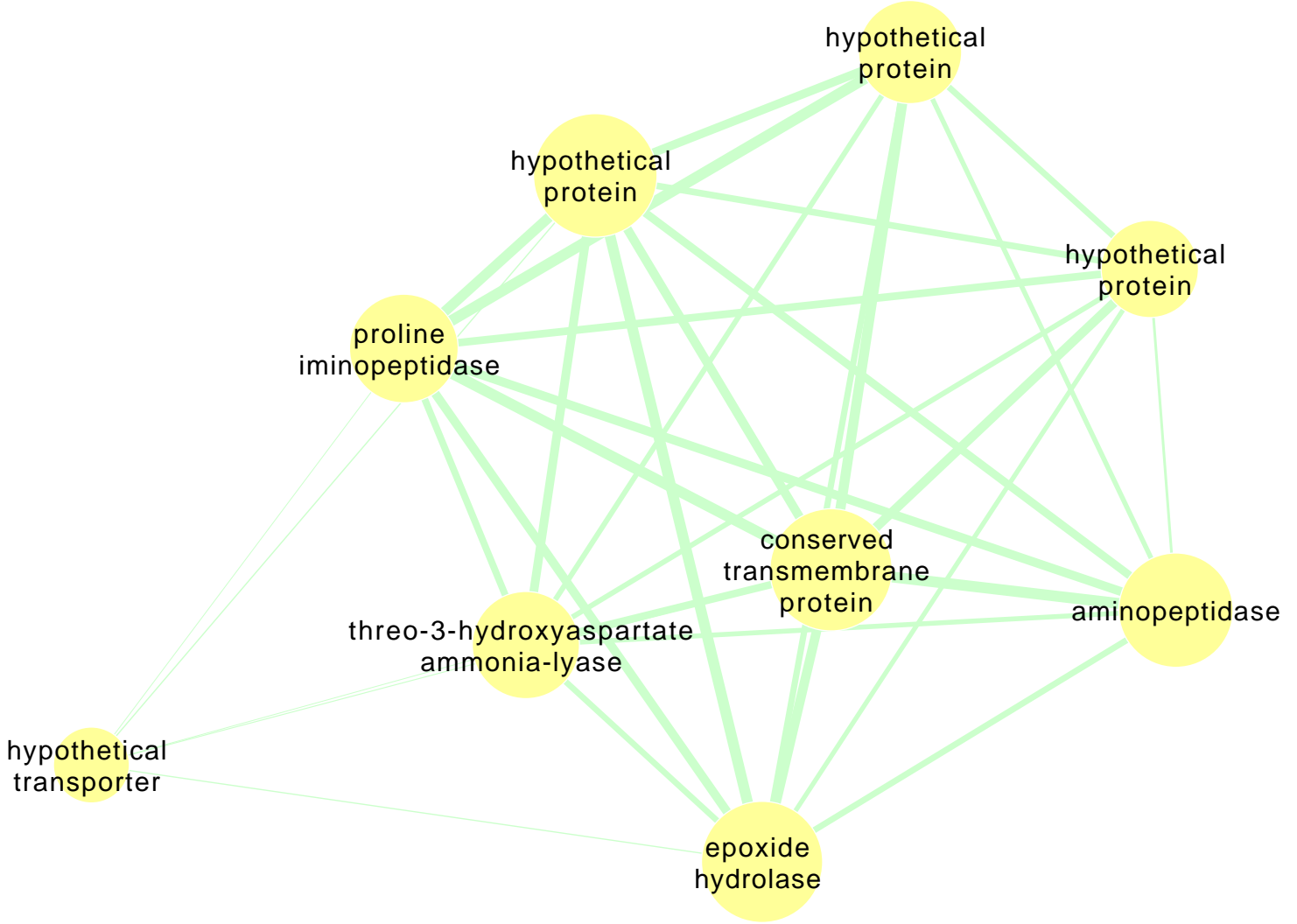

Supplement: Additional file 13: Figure S4 — Co-expression network topologies of all 76 modules. [file 1471-2164-14-450-S13.zip › FigureS4/coral2.pdf]

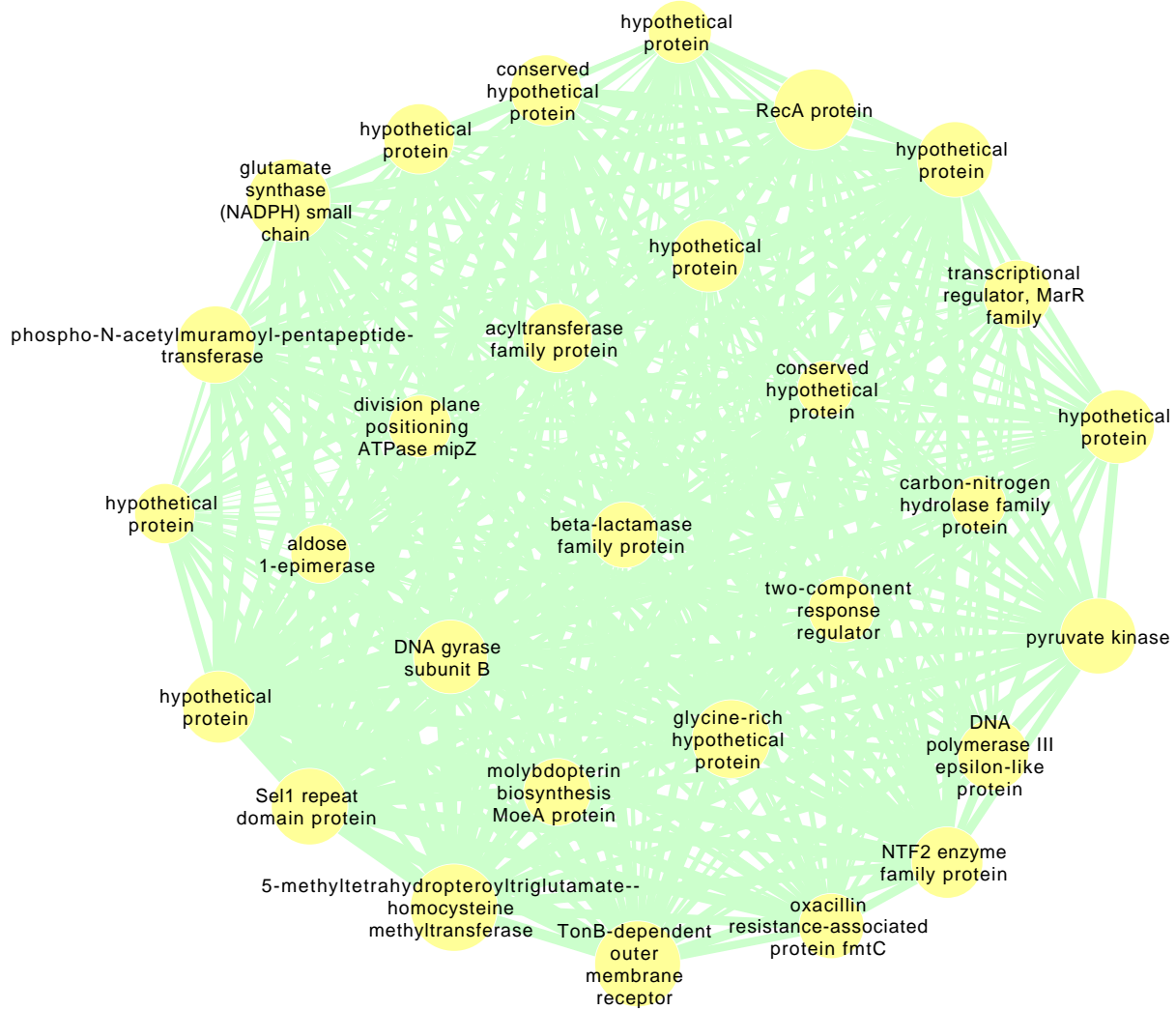

Supplement: Additional file 13: Figure S4 — Co-expression network topologies of all 76 modules. [file 1471-2164-14-450-S13.zip › FigureS4/cyan.pdf]

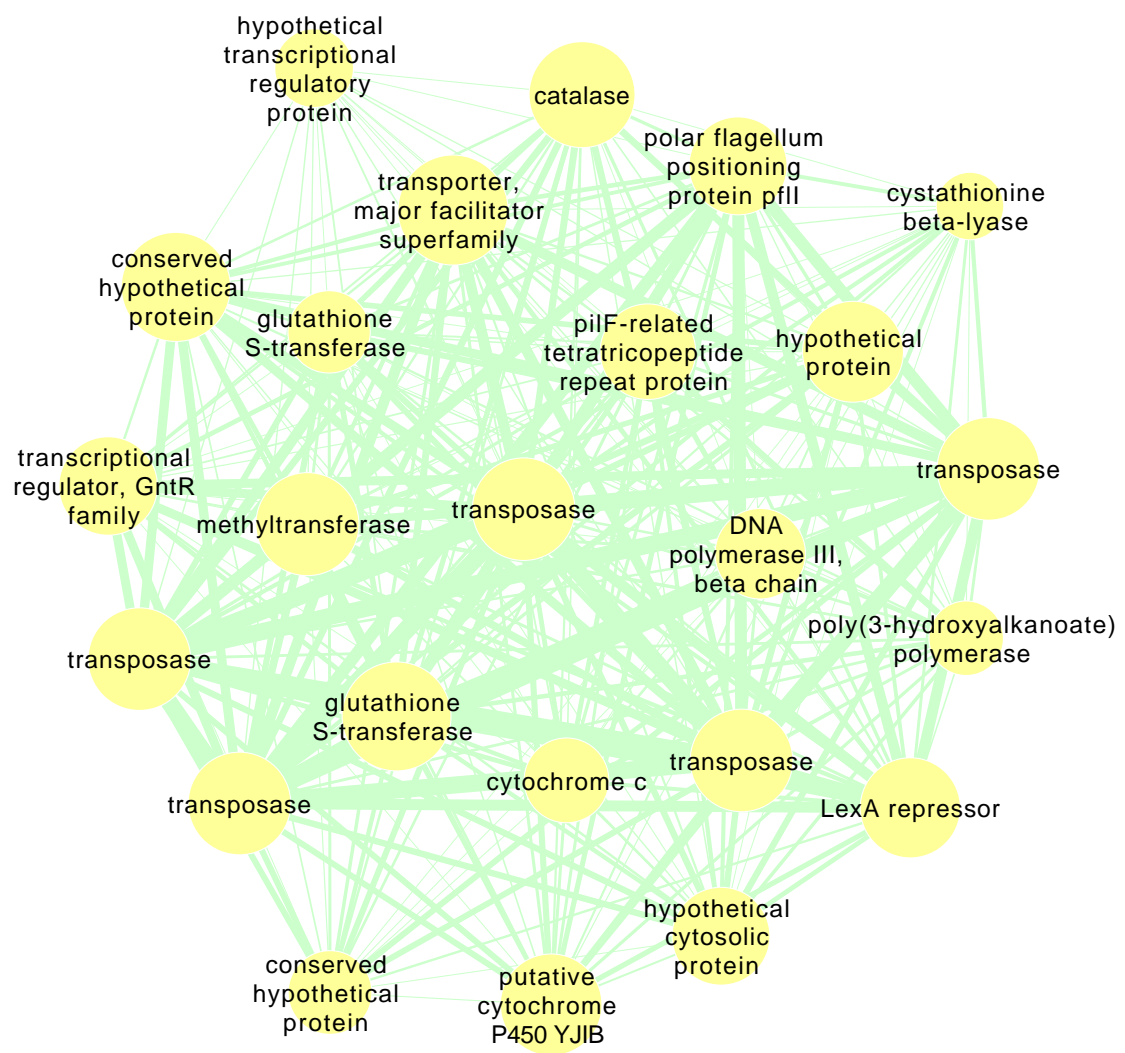

Supplement: Additional file 13: Figure S4 — Co-expression network topologies of all 76 modules. [file 1471-2164-14-450-S13.zip › FigureS4/darkgreen.pdf]

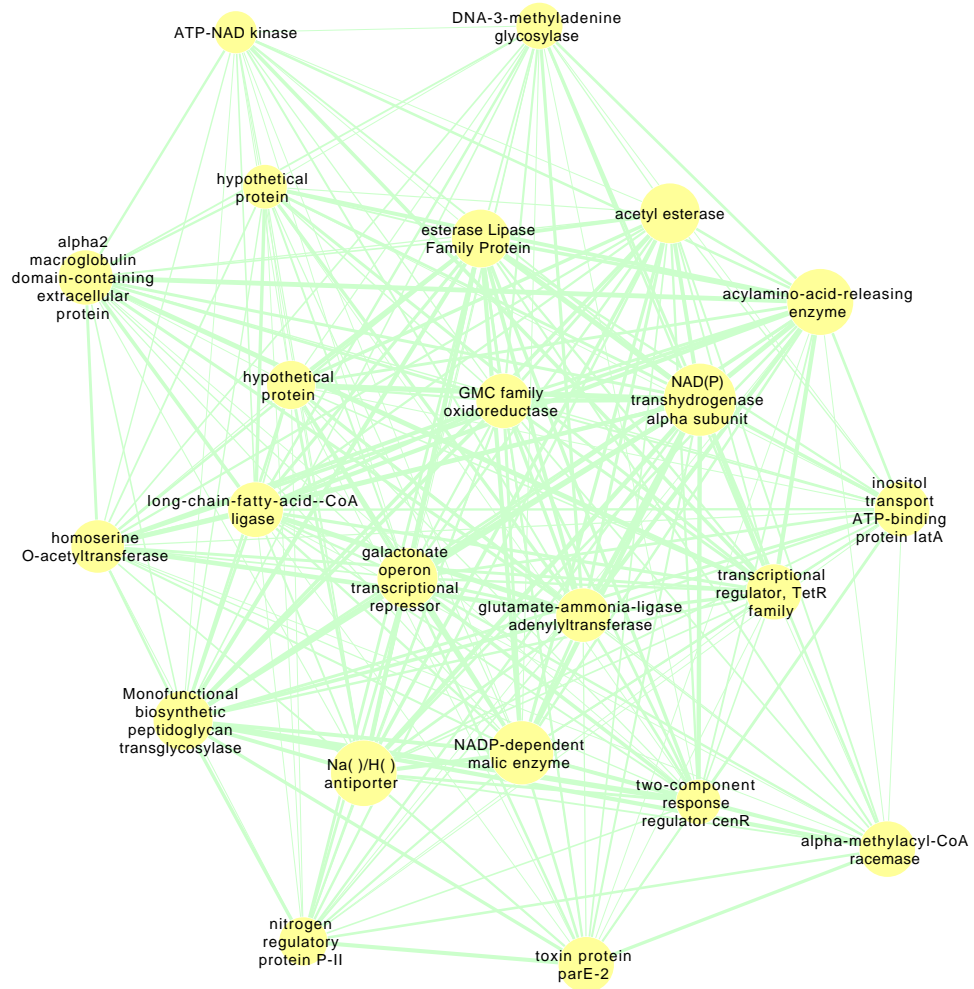

Supplement: Additional file 13: Figure S4 — Co-expression network topologies of all 76 modules. [file 1471-2164-14-450-S13.zip › FigureS4/darkgrey.pdf]

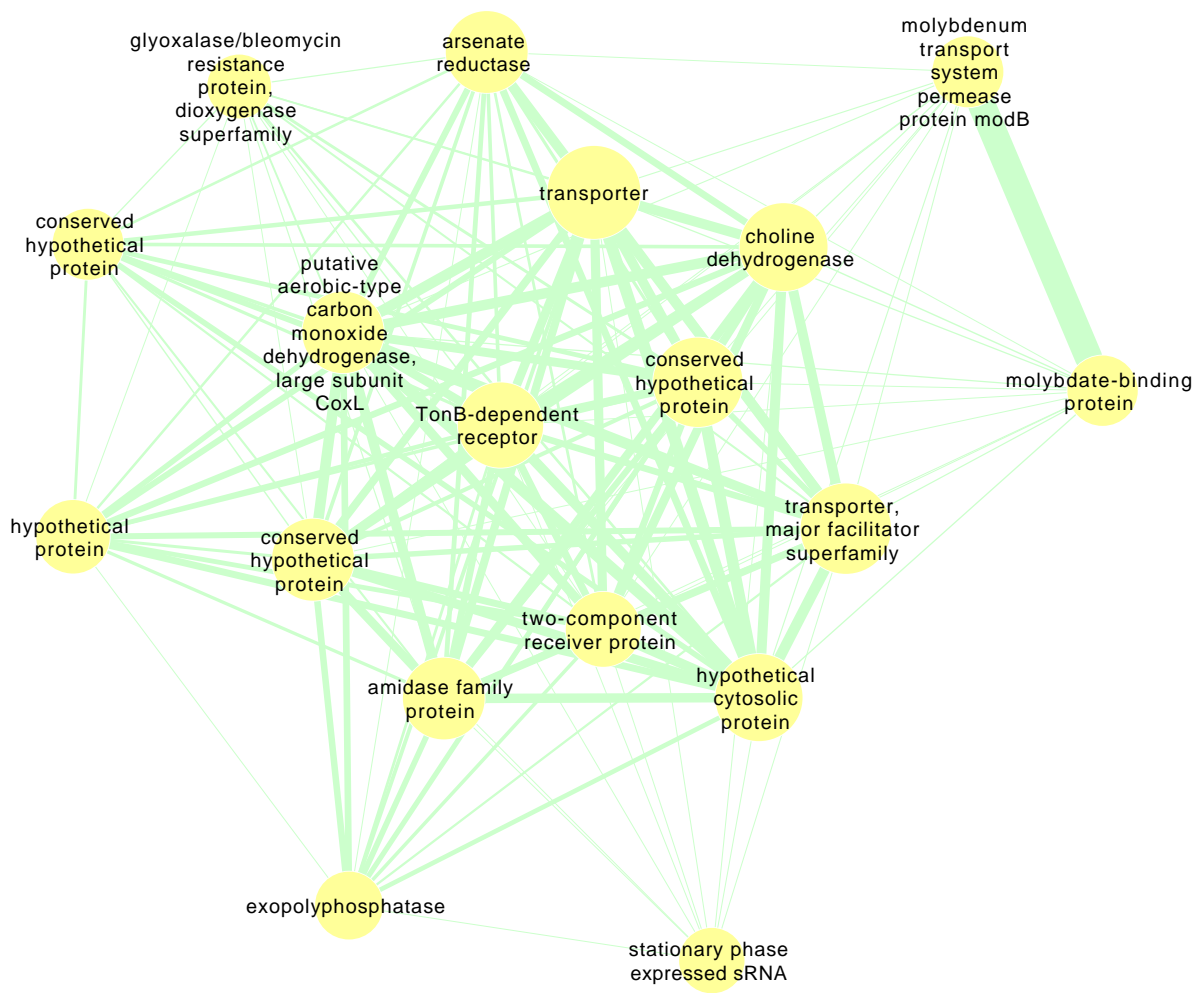

Supplement: Additional file 13: Figure S4 — Co-expression network topologies of all 76 modules. [file 1471-2164-14-450-S13.zip › FigureS4/darkmagenta.pdf]

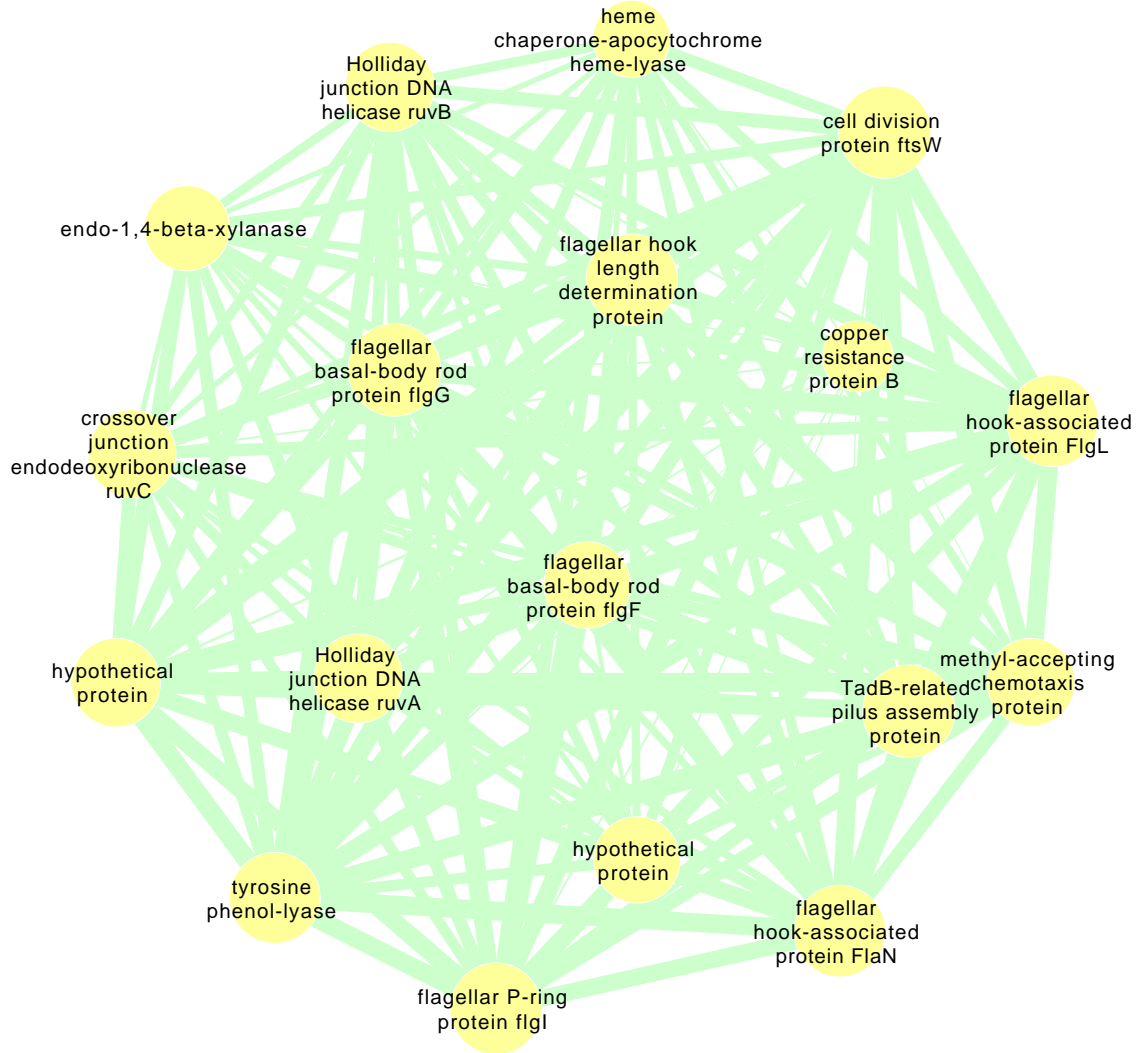

Supplement: Additional file 13: Figure S4 — Co-expression network topologies of all 76 modules. [file 1471-2164-14-450-S13.zip › FigureS4/darkolivegreen.pdf]

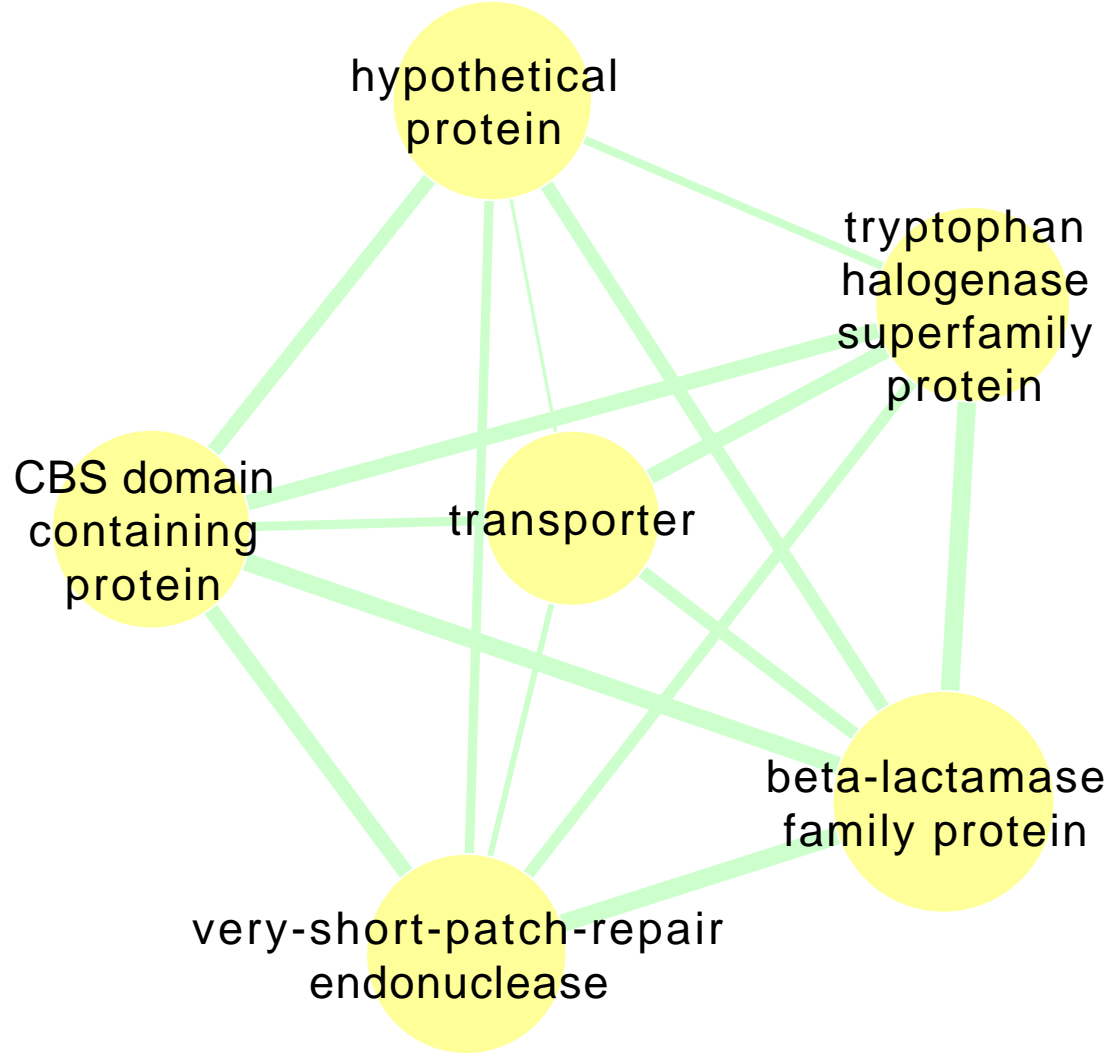

Supplement: Additional file 13: Figure S4 — Co-expression network topologies of all 76 modules. [file 1471-2164-14-450-S13.zip › FigureS4/darkolivegreen4.pdf]

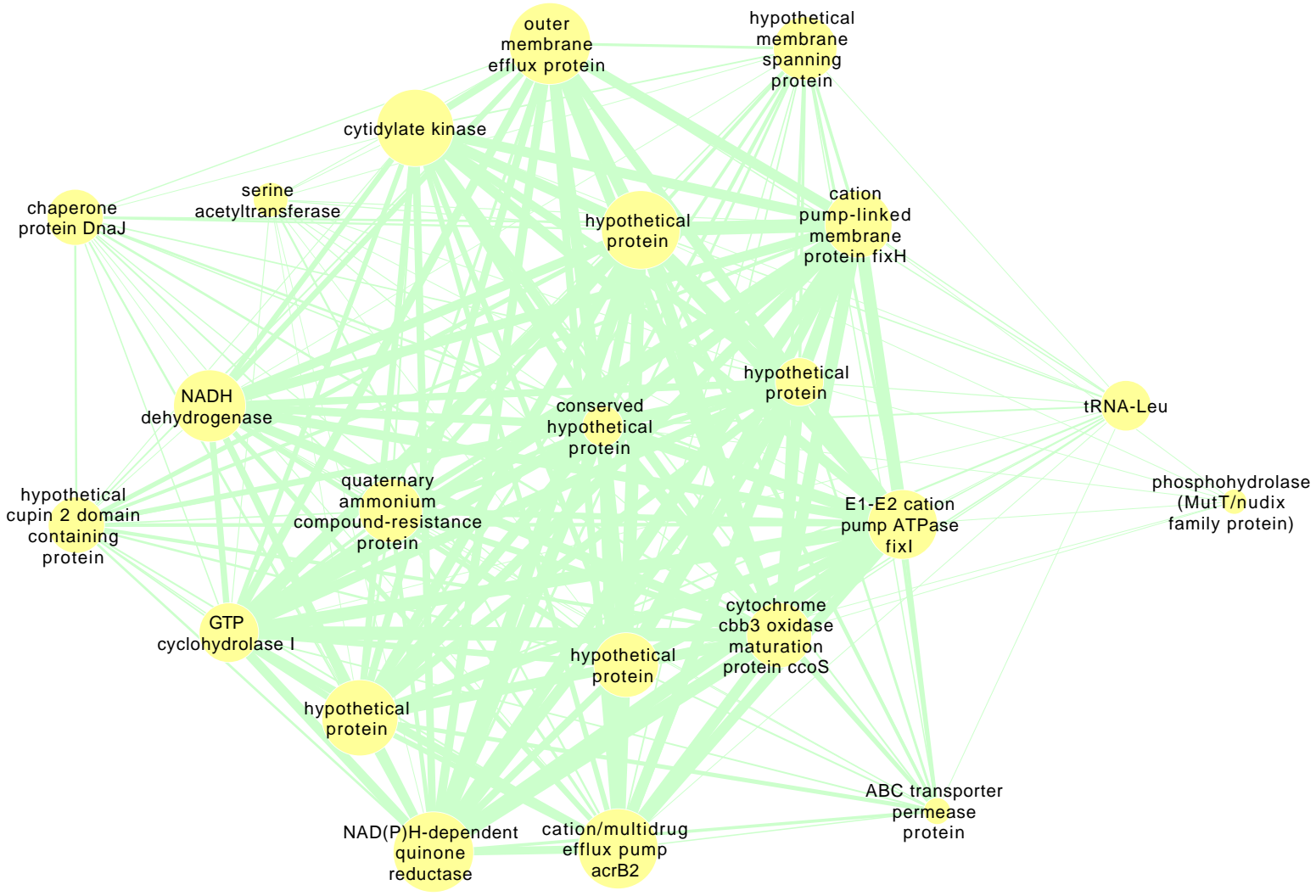

Supplement: Additional file 13: Figure S4 — Co-expression network topologies of all 76 modules. [file 1471-2164-14-450-S13.zip › FigureS4/darkorange.pdf]

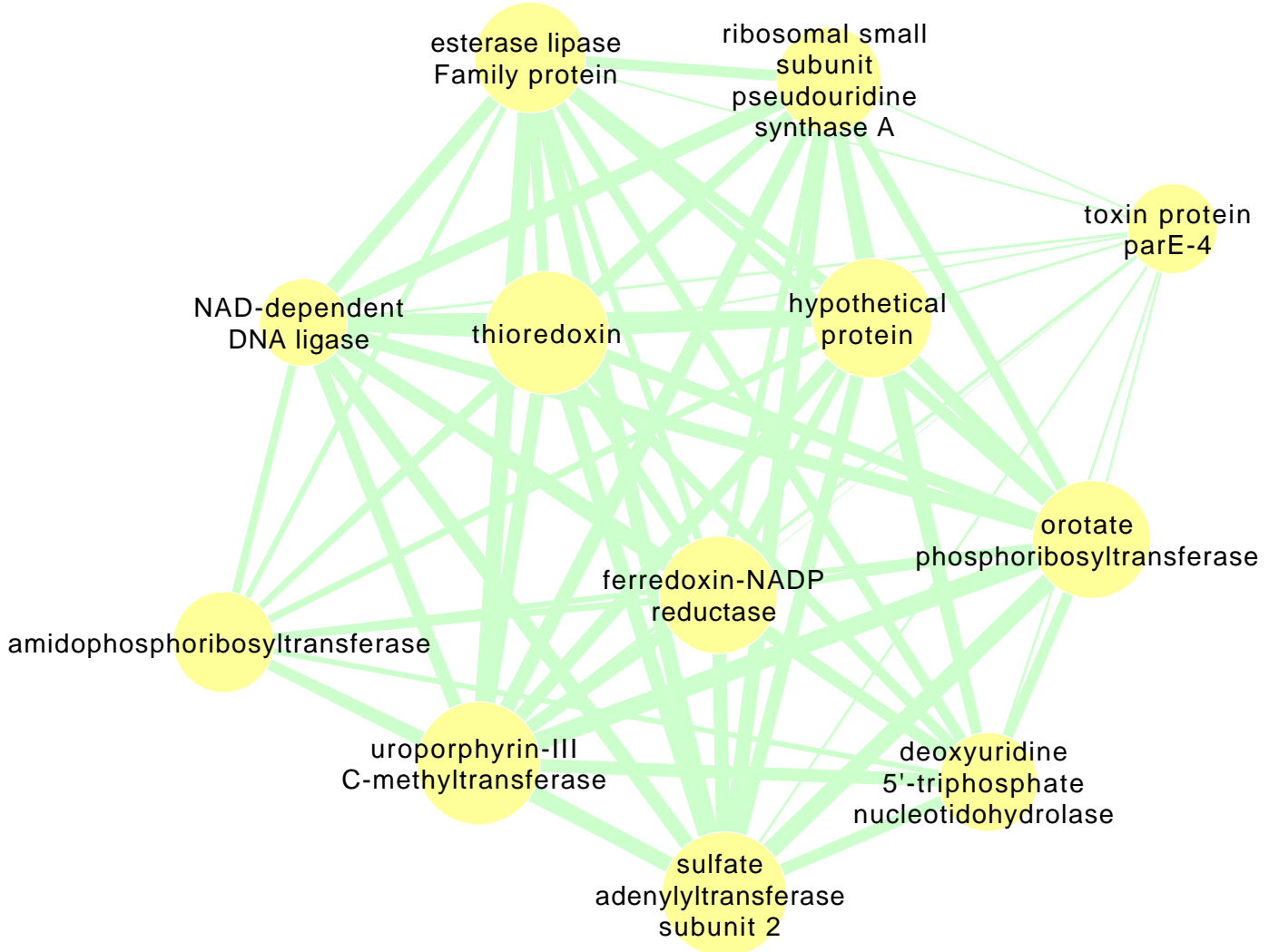

Supplement: Additional file 13: Figure S4 — Co-expression network topologies of all 76 modules. [file 1471-2164-14-450-S13.zip › FigureS4/darkorange2.pdf]

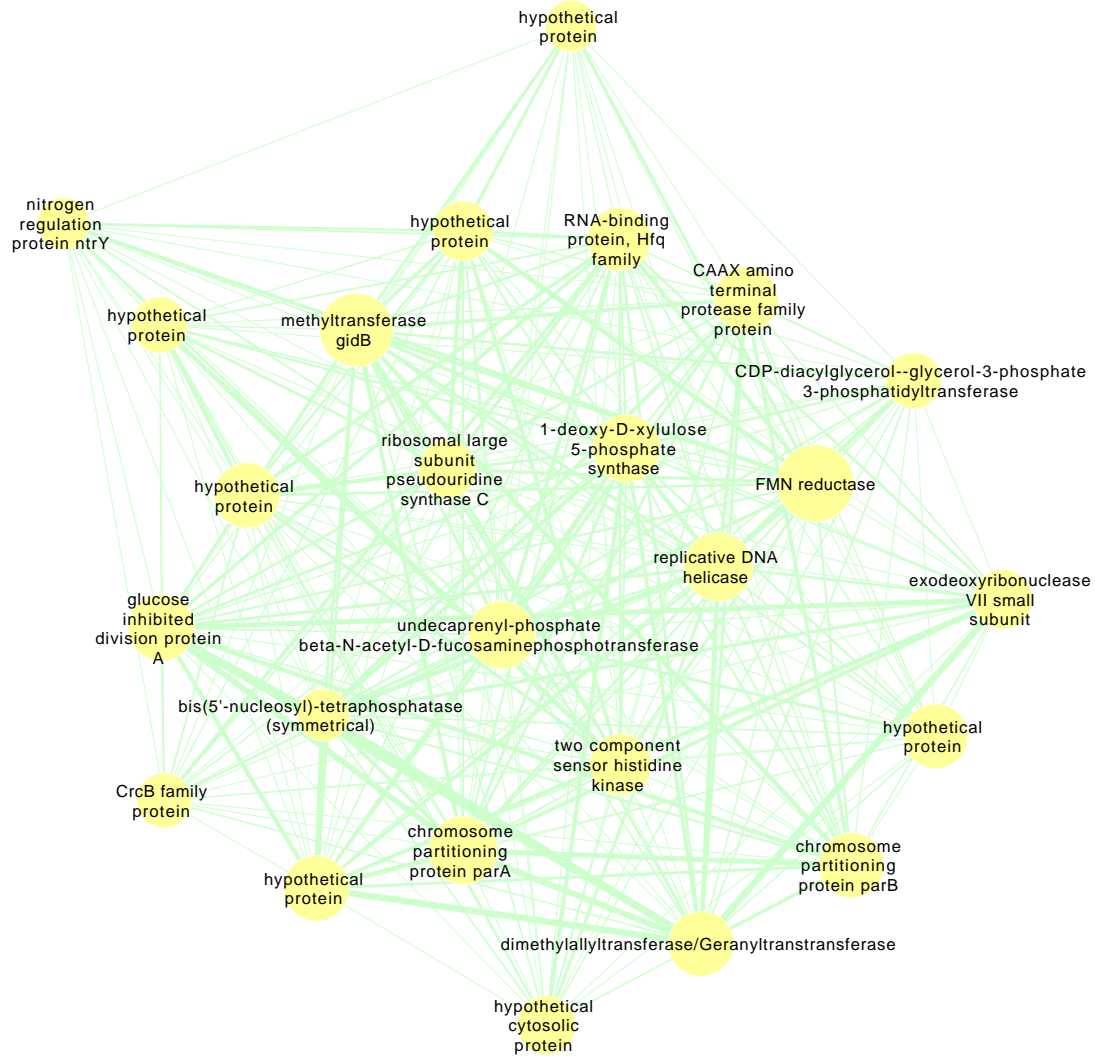

Supplement: Additional file 13: Figure S4 — Co-expression network topologies of all 76 modules. [file 1471-2164-14-450-S13.zip › FigureS4/darkred.pdf]

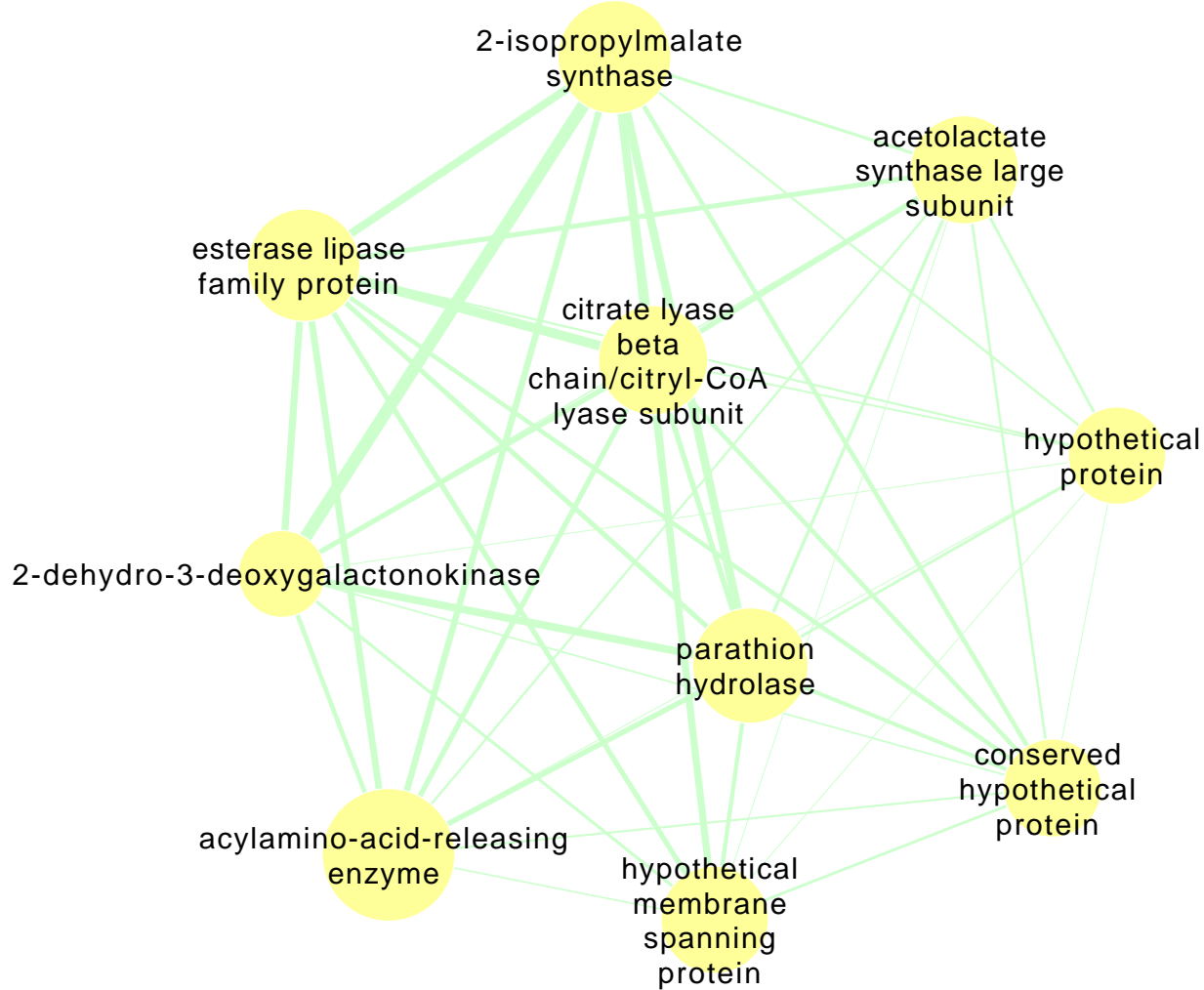

Supplement: Additional file 13: Figure S4 — Co-expression network topologies of all 76 modules. [file 1471-2164-14-450-S13.zip › FigureS4/darkseagreen4.pdf]

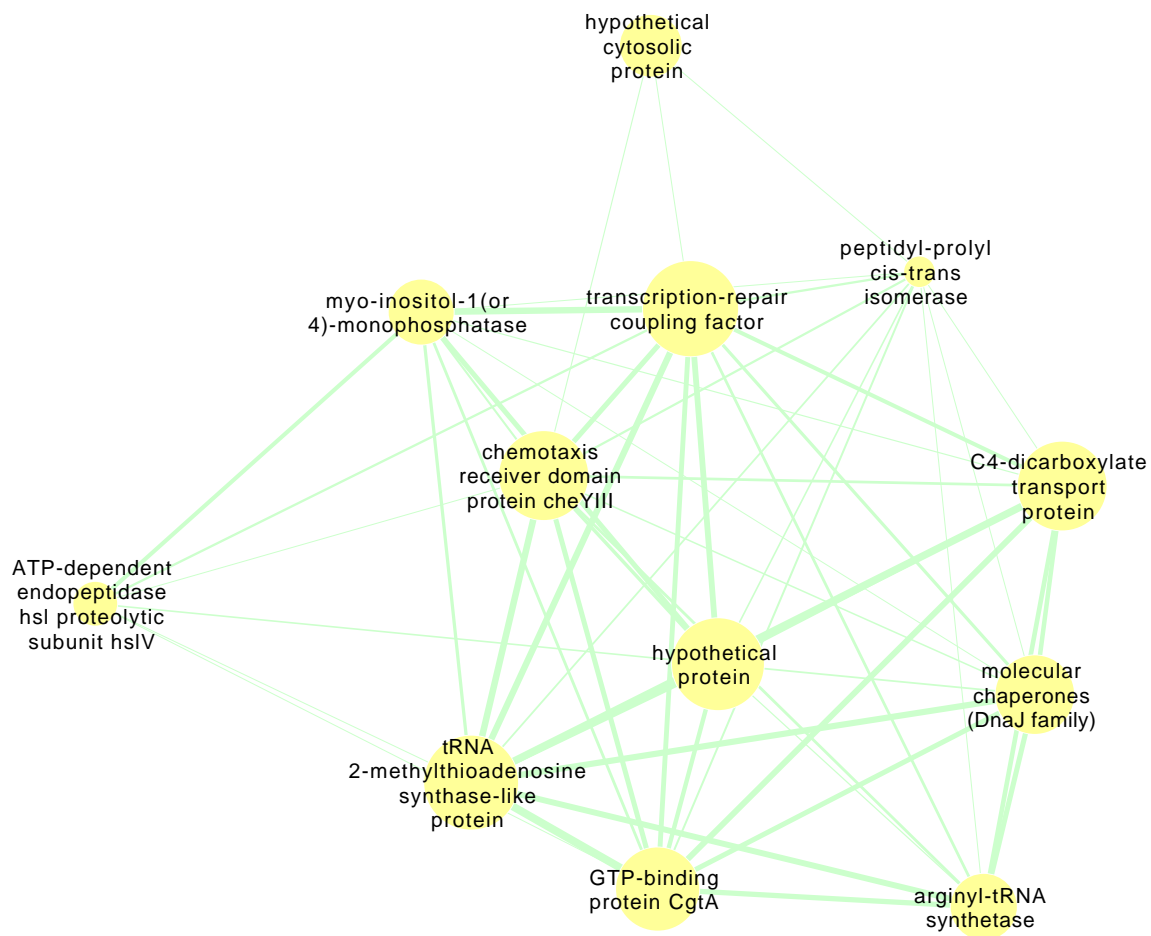

Supplement: Additional file 13: Figure S4 — Co-expression network topologies of all 76 modules. [file 1471-2164-14-450-S13.zip › FigureS4/darkslateblue.pdf]

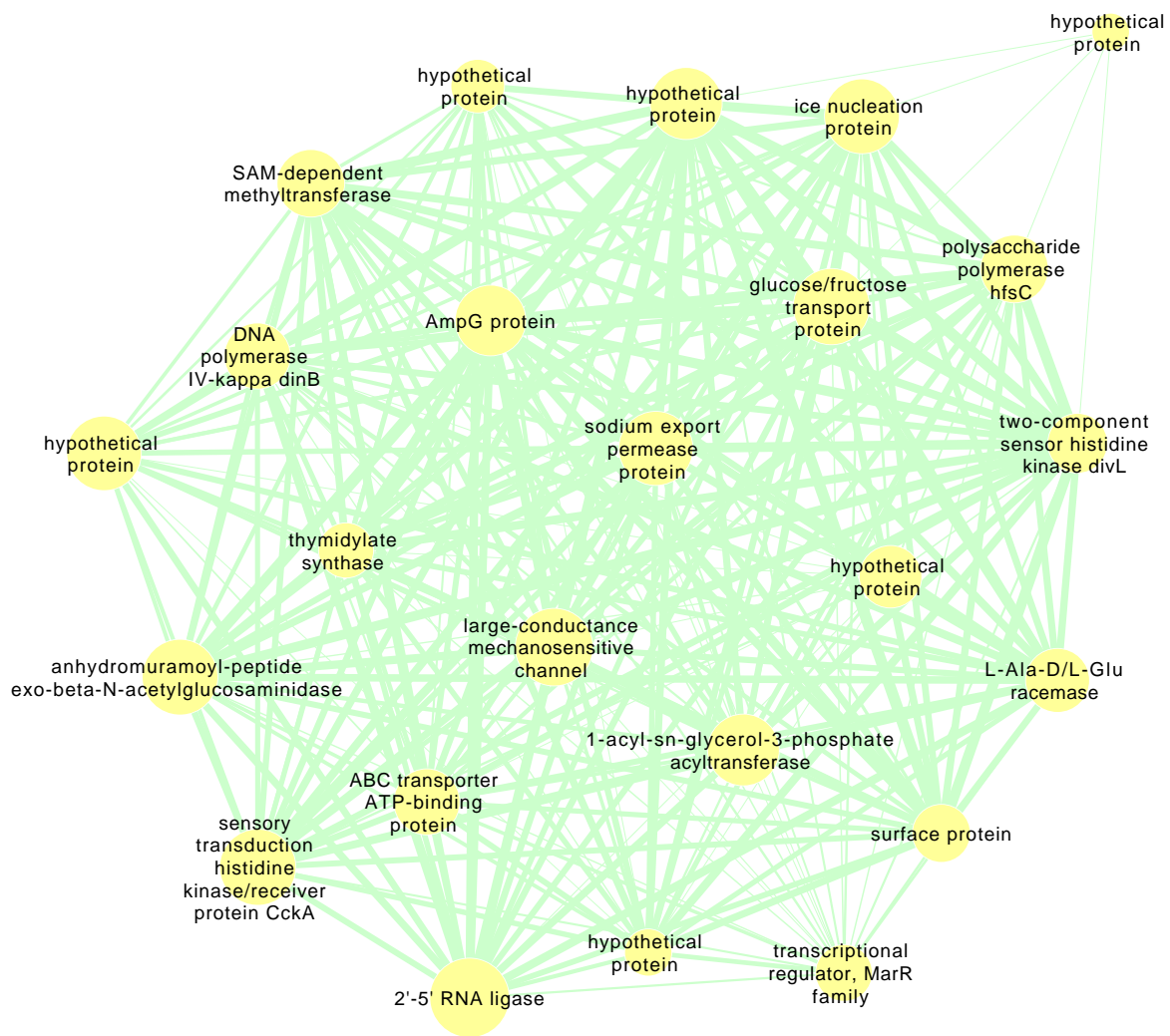

Supplement: Additional file 13: Figure S4 — Co-expression network topologies of all 76 modules. [file 1471-2164-14-450-S13.zip › FigureS4/darkturquoise.pdf]

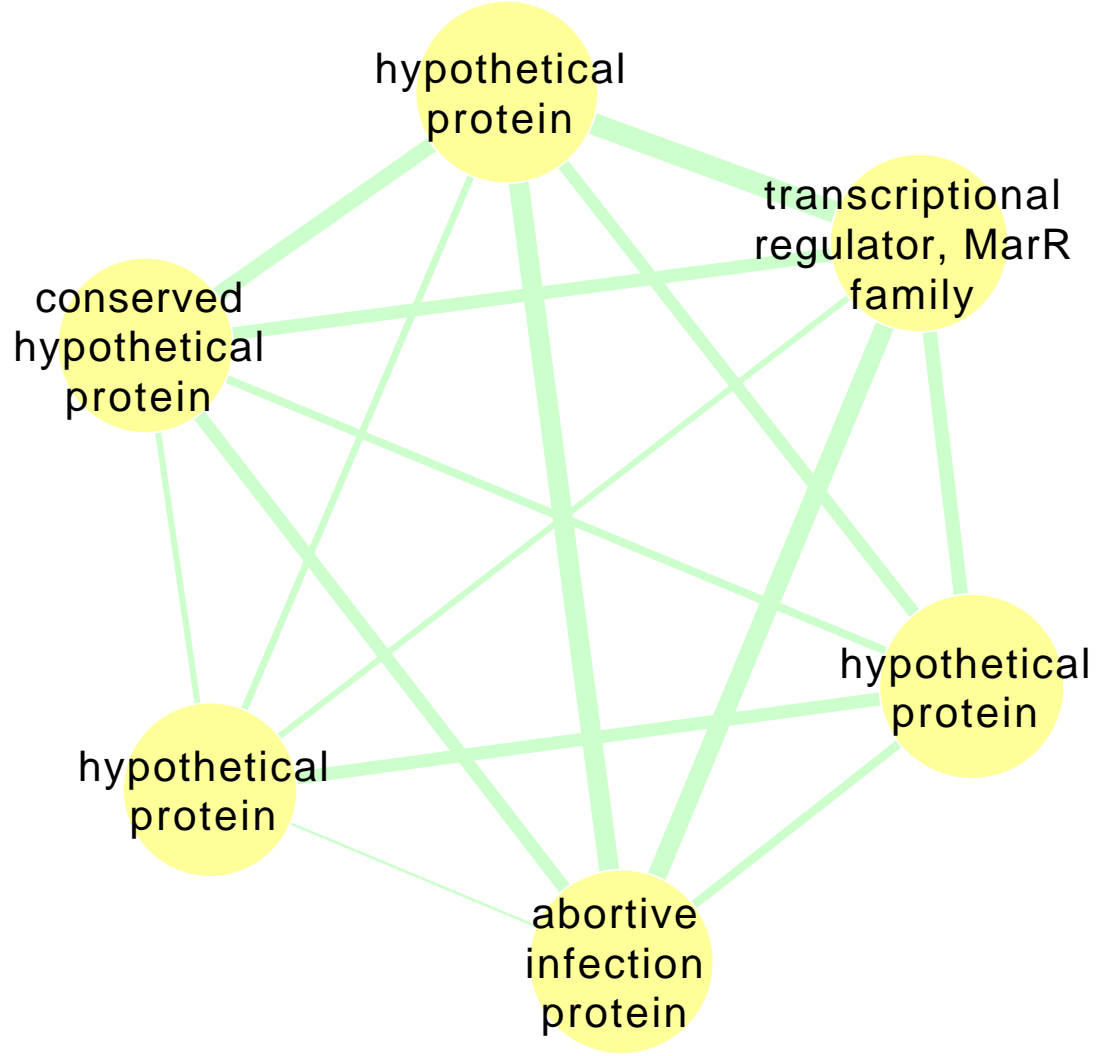

Supplement: Additional file 13: Figure S4 — Co-expression network topologies of all 76 modules. [file 1471-2164-14-450-S13.zip › FigureS4/firebrick4.pdf]

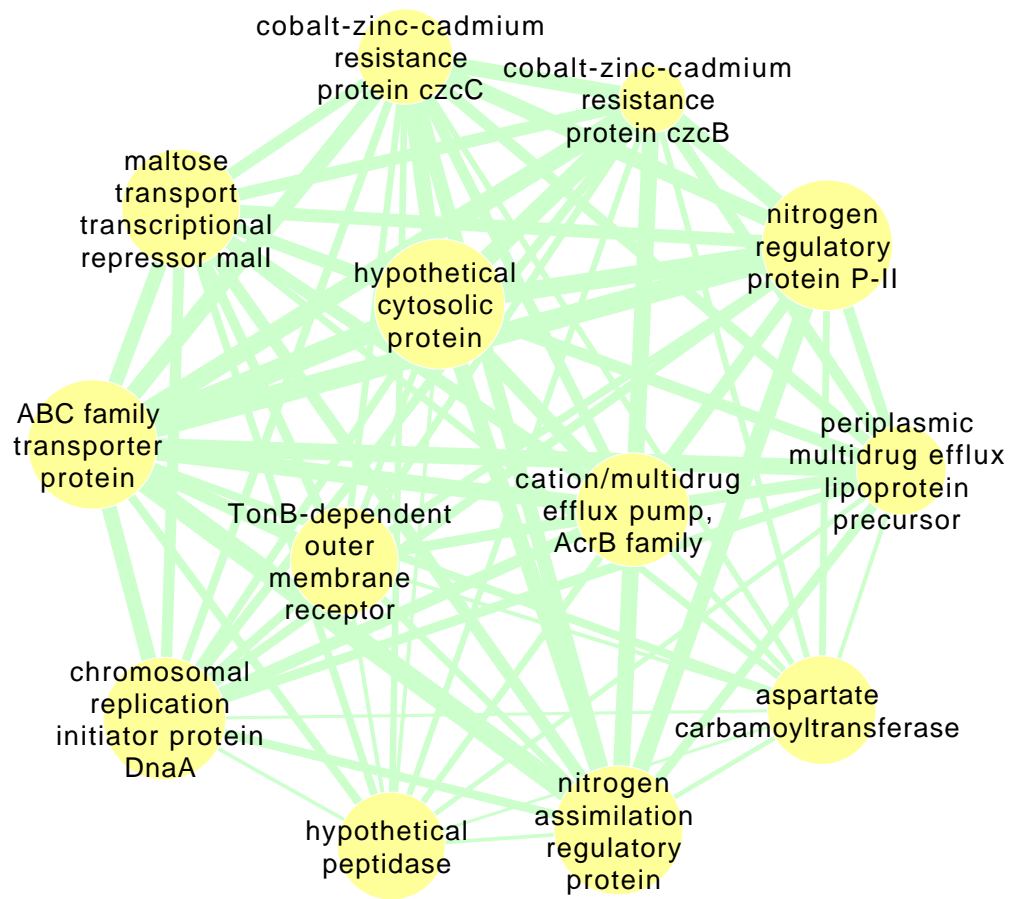

Supplement: Additional file 13: Figure S4 — Co-expression network topologies of all 76 modules. [file 1471-2164-14-450-S13.zip › FigureS4/floralwhite.pdf]

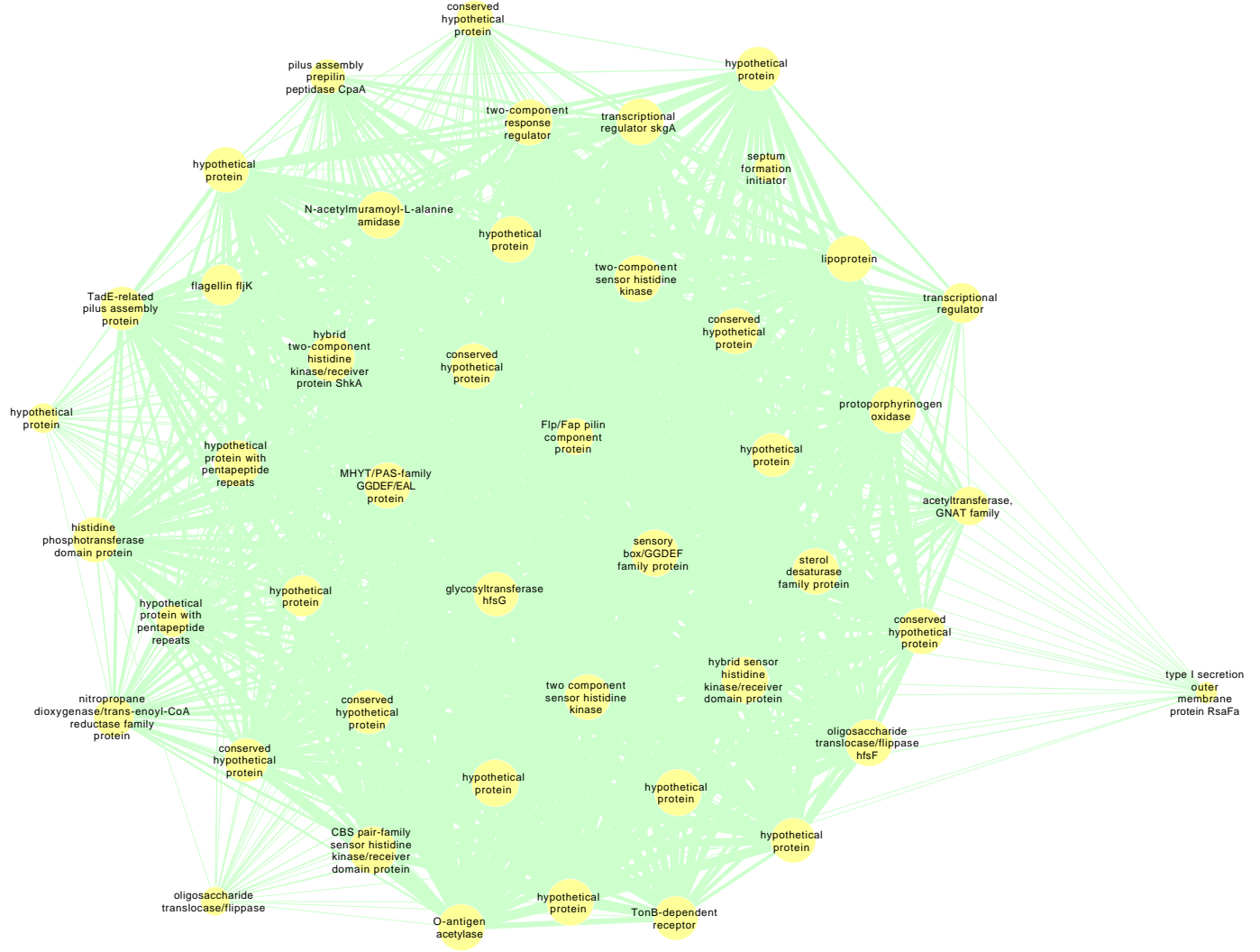

Supplement: Additional file 13: Figure S4 — Co-expression network topologies of all 76 modules. [file 1471-2164-14-450-S13.zip › FigureS4/green.pdf]

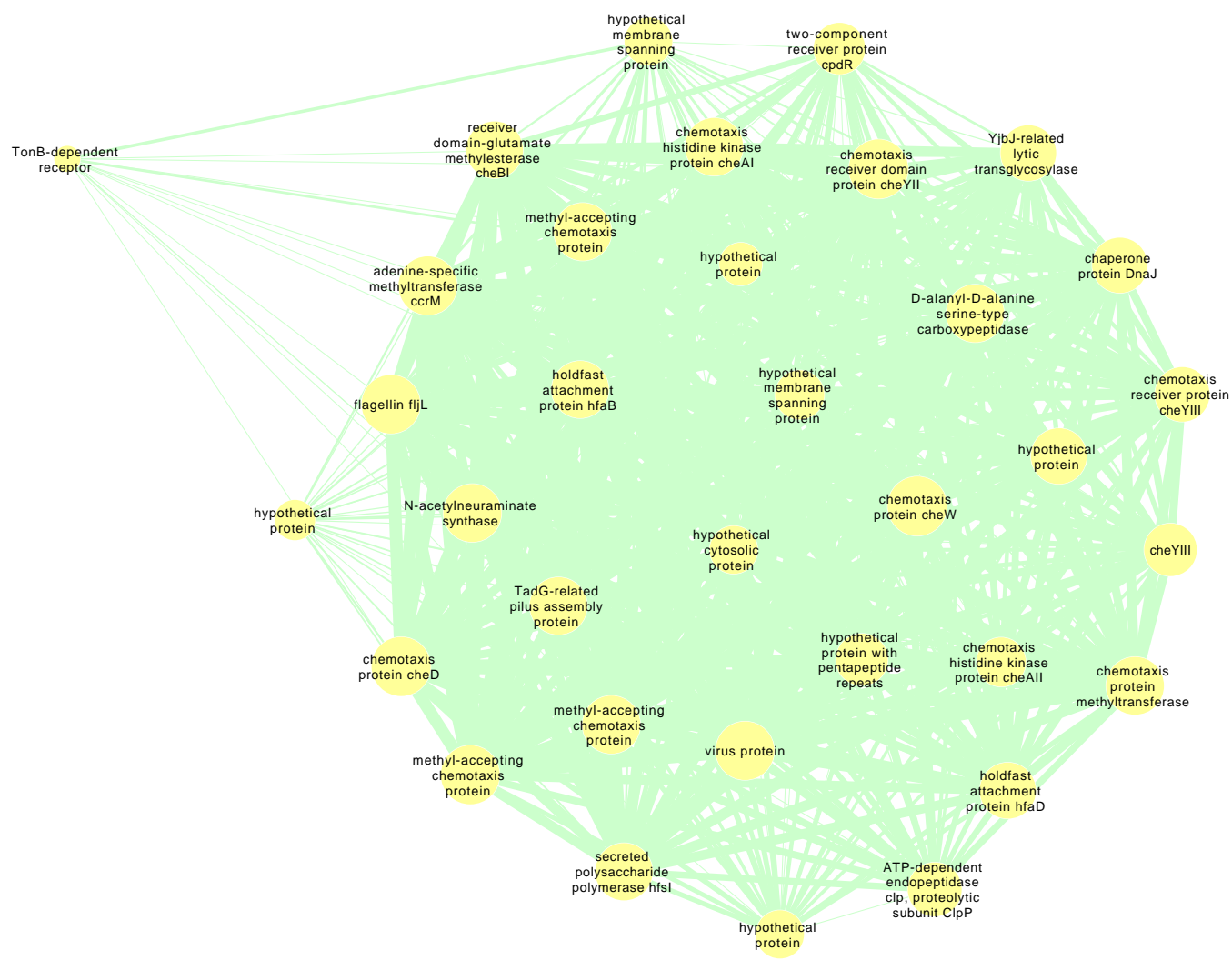

Supplement: Additional file 13: Figure S4 — Co-expression network topologies of all 76 modules. [file 1471-2164-14-450-S13.zip › FigureS4/greenyellow.pdf]

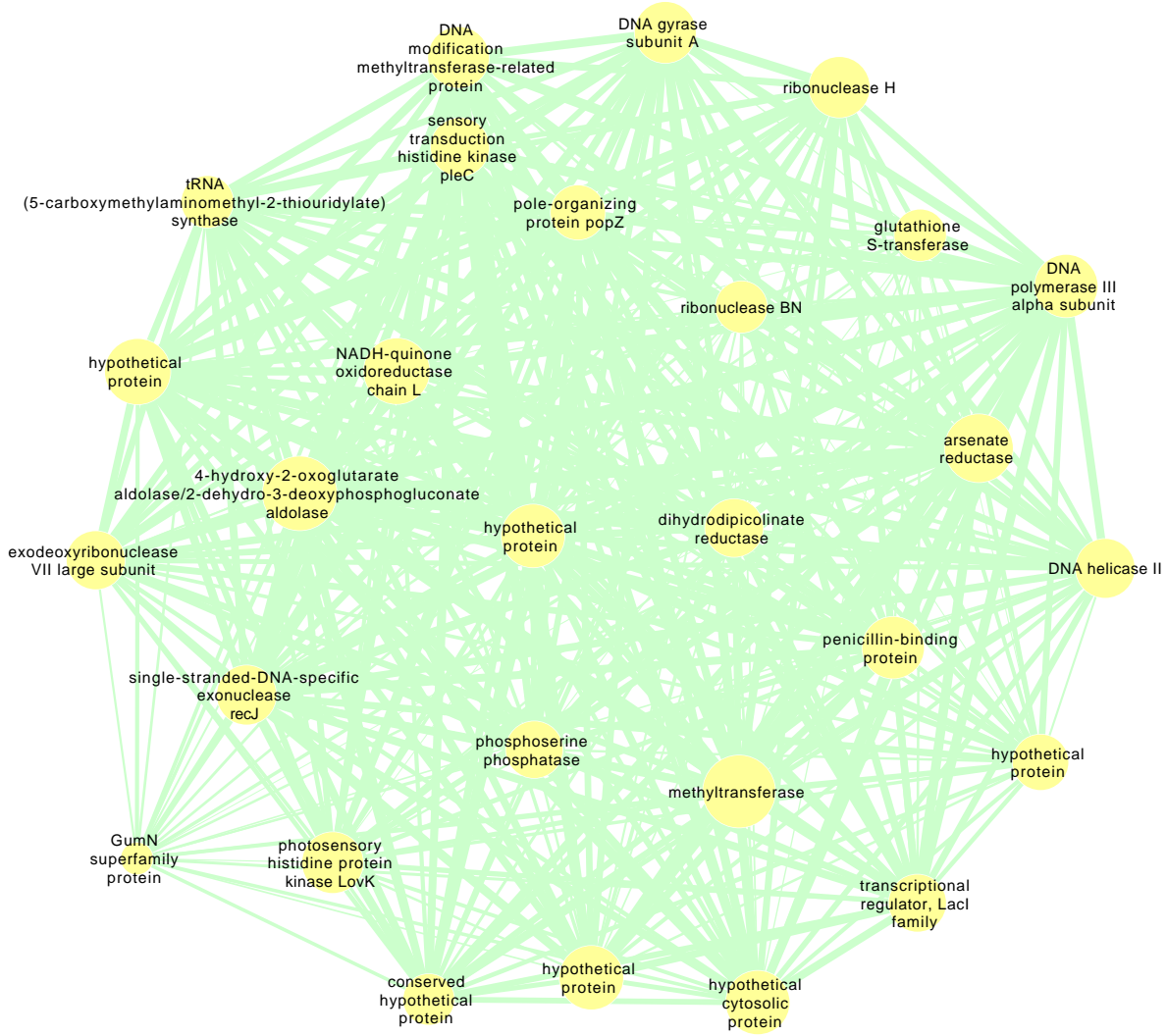

Supplement: Additional file 13: Figure S4 — Co-expression network topologies of all 76 modules. [file 1471-2164-14-450-S13.zip › FigureS4/grey60.pdf]

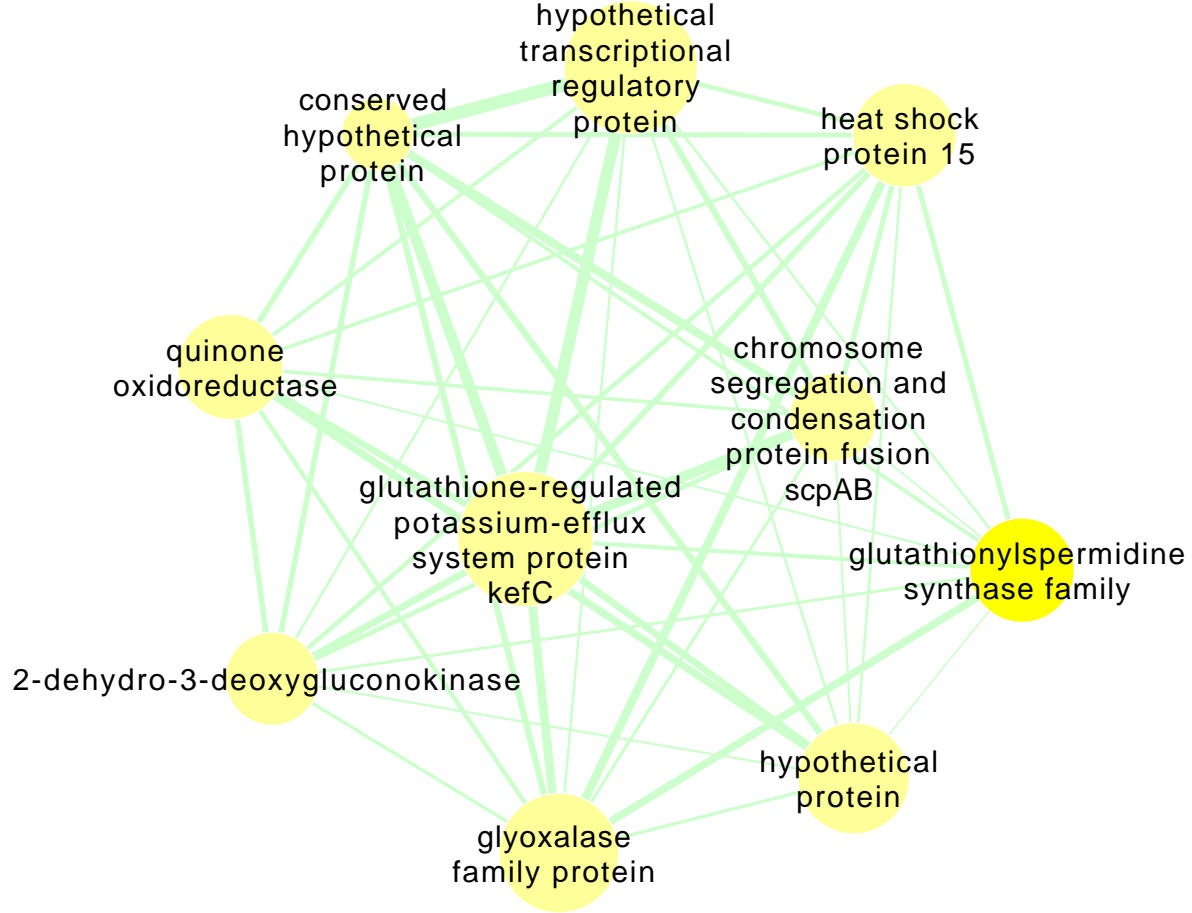

Supplement: Additional file 13: Figure S4 — Co-expression network topologies of all 76 modules. [file 1471-2164-14-450-S13.zip › FigureS4/honeydew1.pdf]

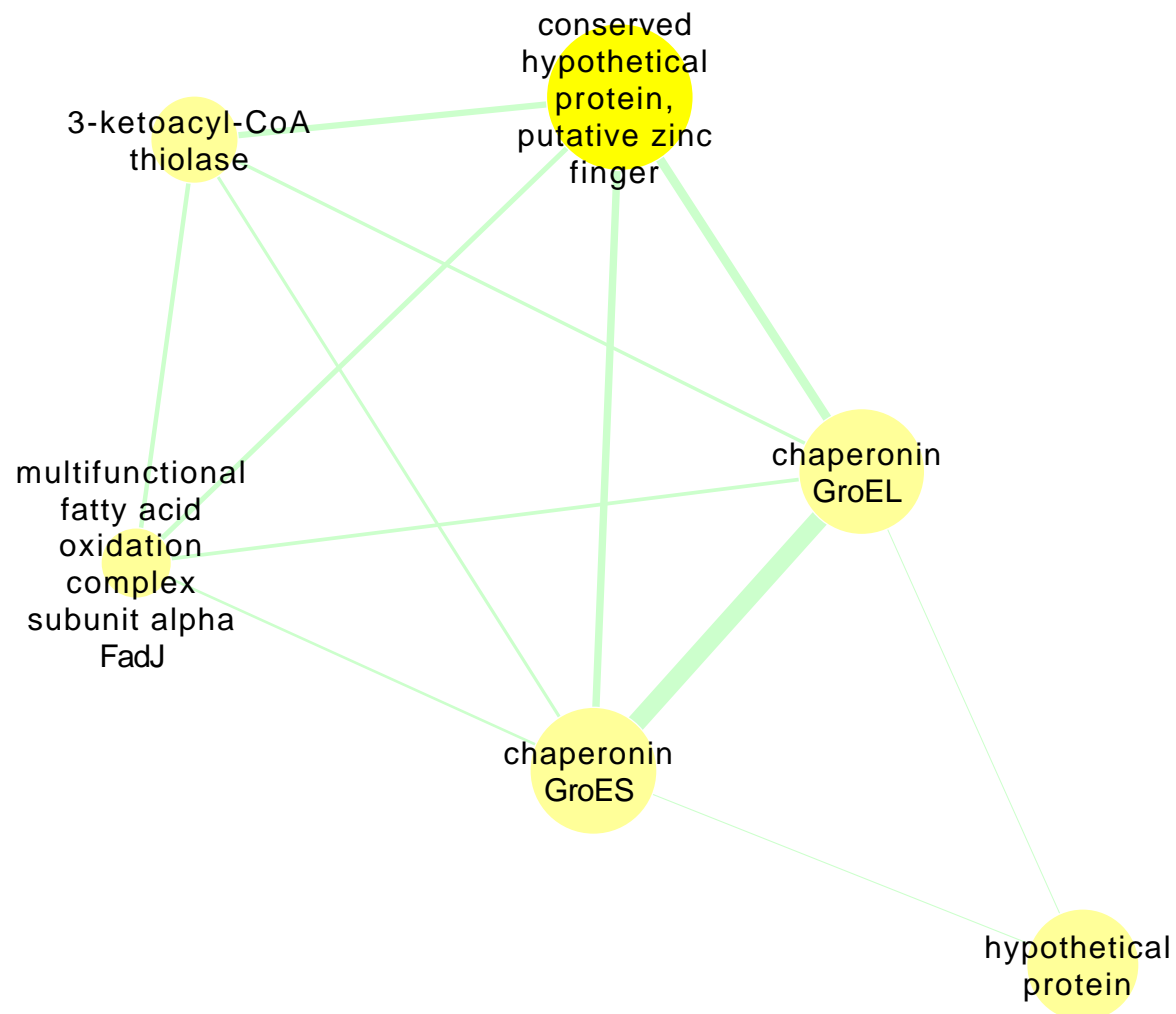

Supplement: Additional file 13: Figure S4 — Co-expression network topologies of all 76 modules. [file 1471-2164-14-450-S13.zip › FigureS4/indianred4.pdf]

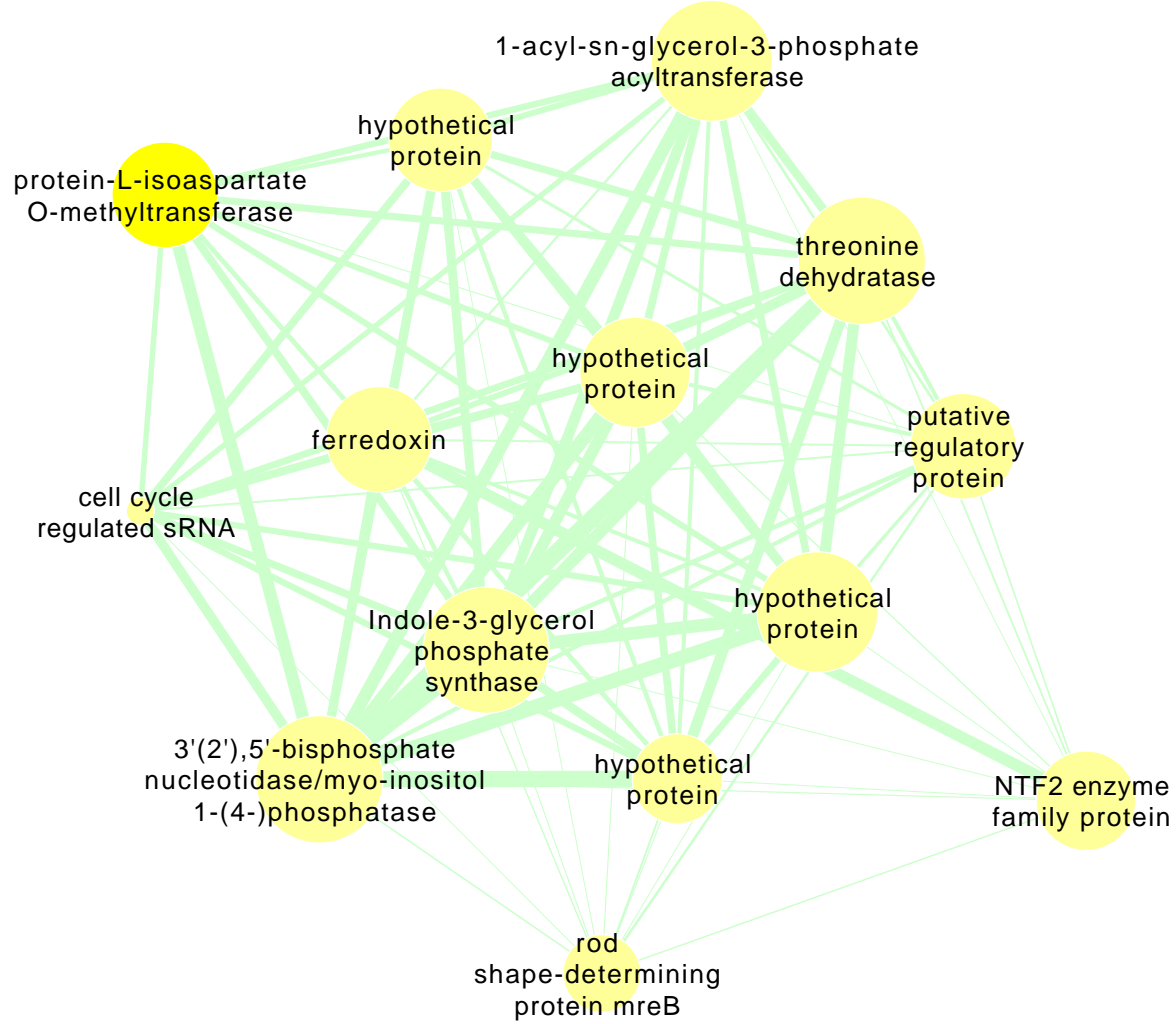

Supplement: Additional file 13: Figure S4 — Co-expression network topologies of all 76 modules. [file 1471-2164-14-450-S13.zip › FigureS4/ivory.pdf]

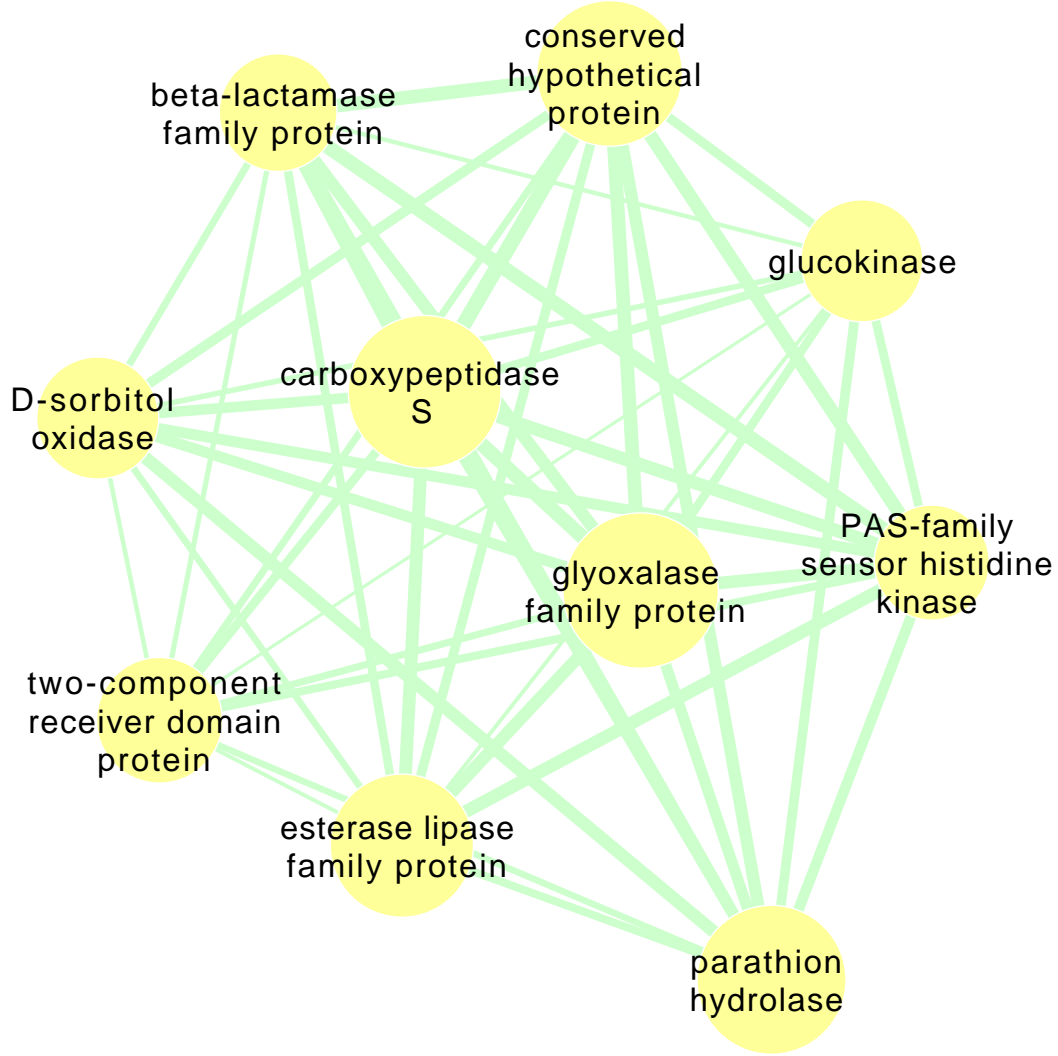

Supplement: Additional file 13: Figure S4 — Co-expression network topologies of all 76 modules. [file 1471-2164-14-450-S13.zip › FigureS4/lavenderblush3.pdf]

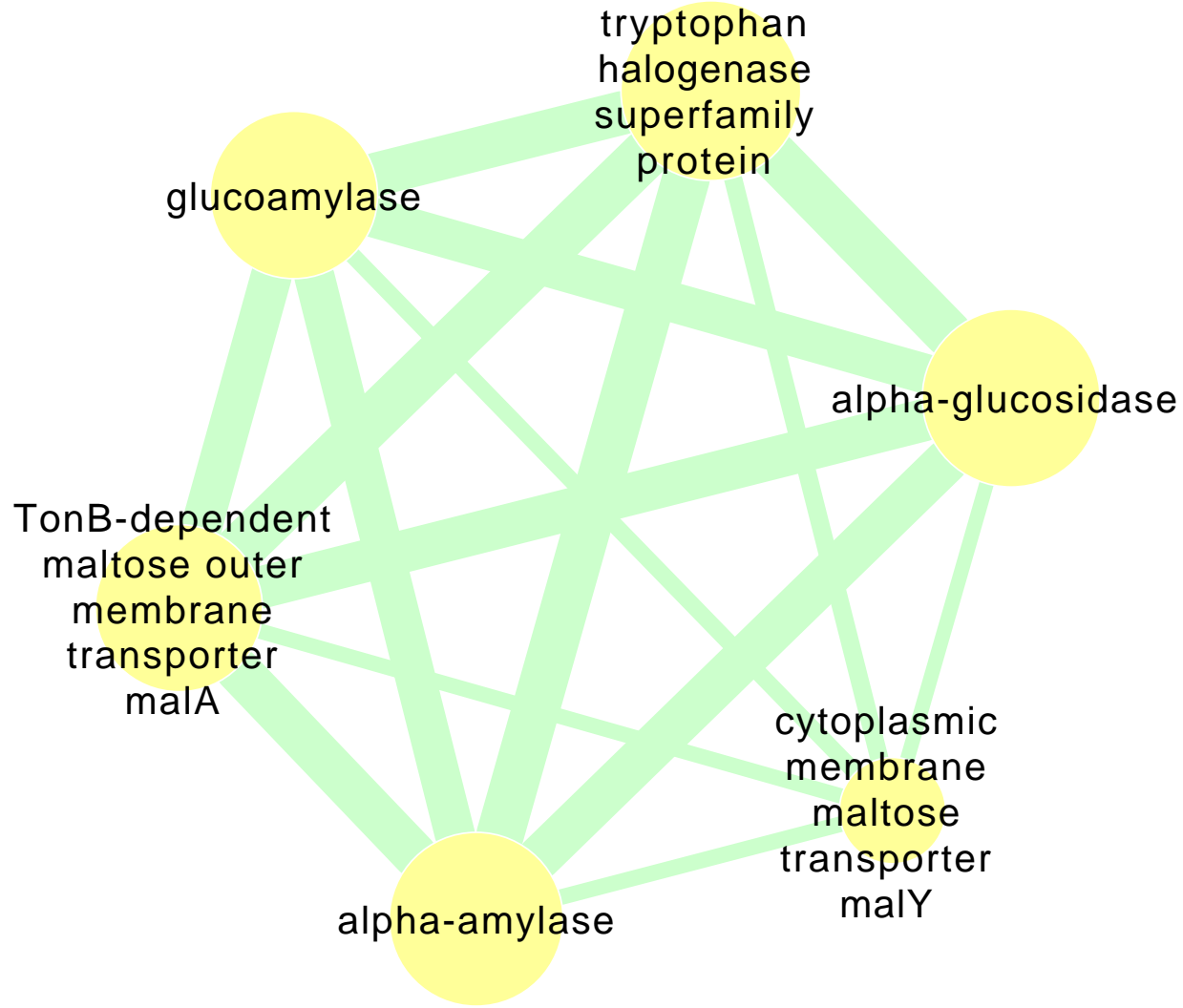

Supplement: Additional file 13: Figure S4 — Co-expression network topologies of all 76 modules. [file 1471-2164-14-450-S13.zip › FigureS4/lightcoral.pdf]

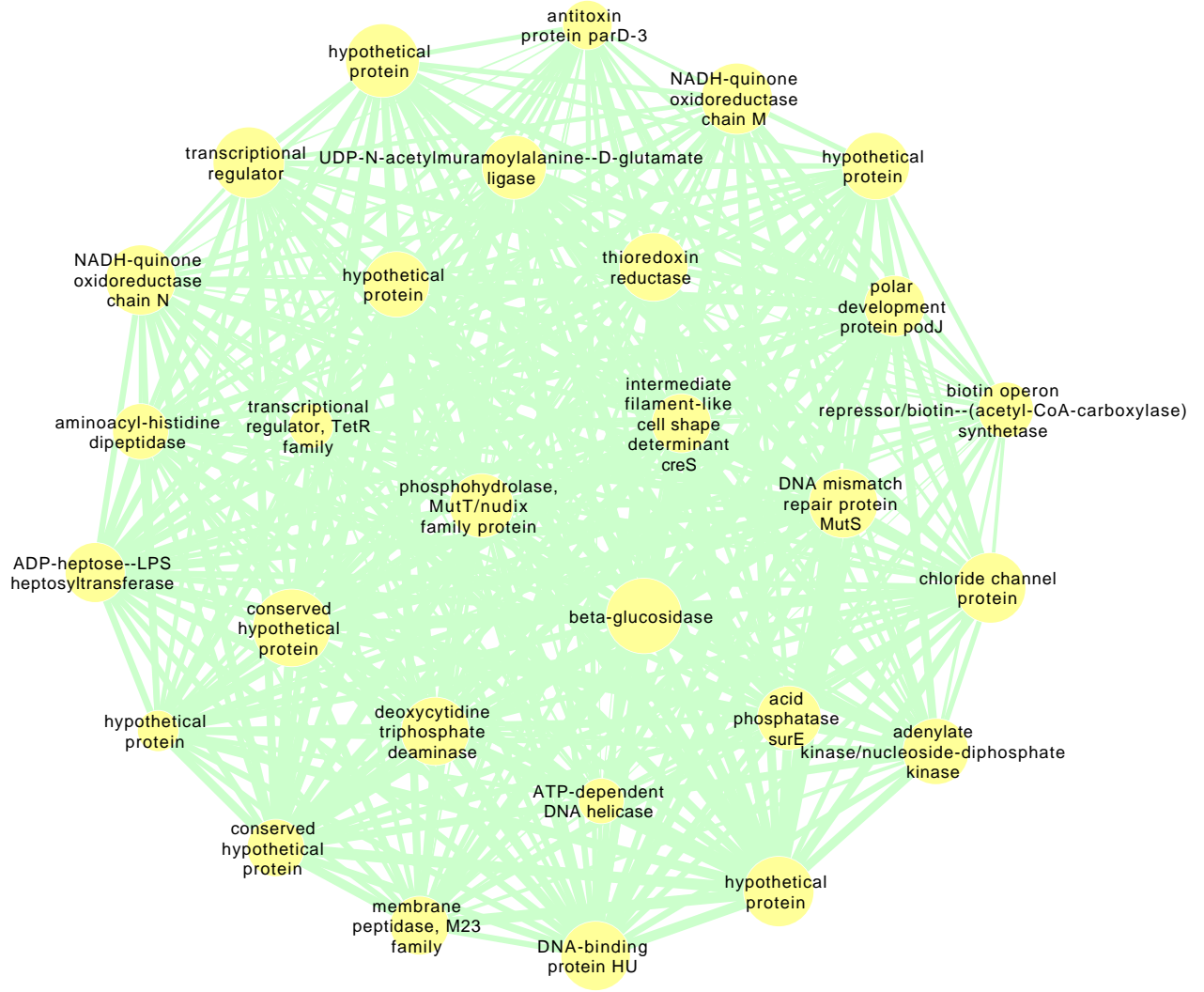

Supplement: Additional file 13: Figure S4 — Co-expression network topologies of all 76 modules. [file 1471-2164-14-450-S13.zip › FigureS4/lightcyan.pdf]

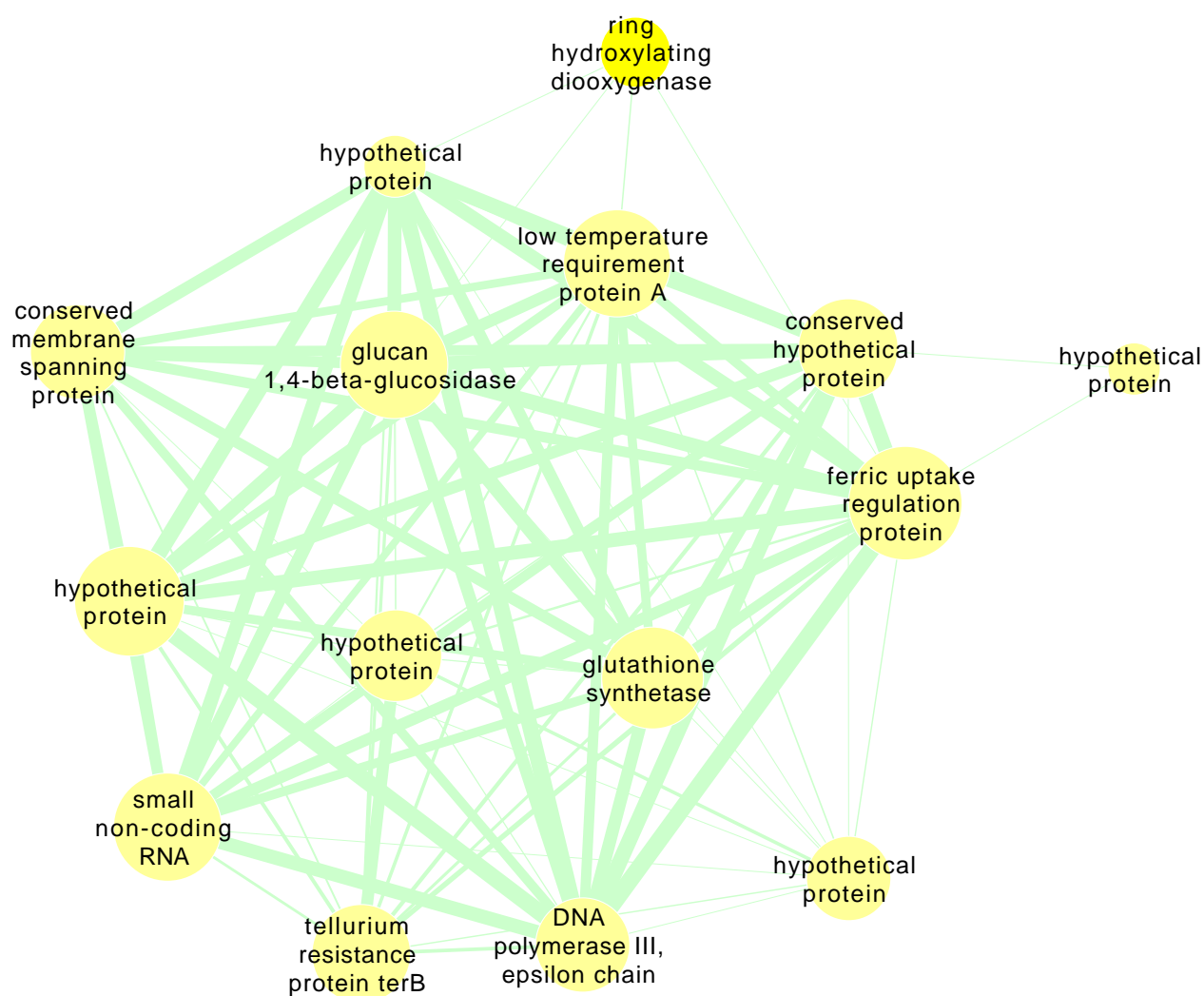

Supplement: Additional file 13: Figure S4 — Co-expression network topologies of all 76 modules. [file 1471-2164-14-450-S13.zip › FigureS4/lightcyan1.pdf]

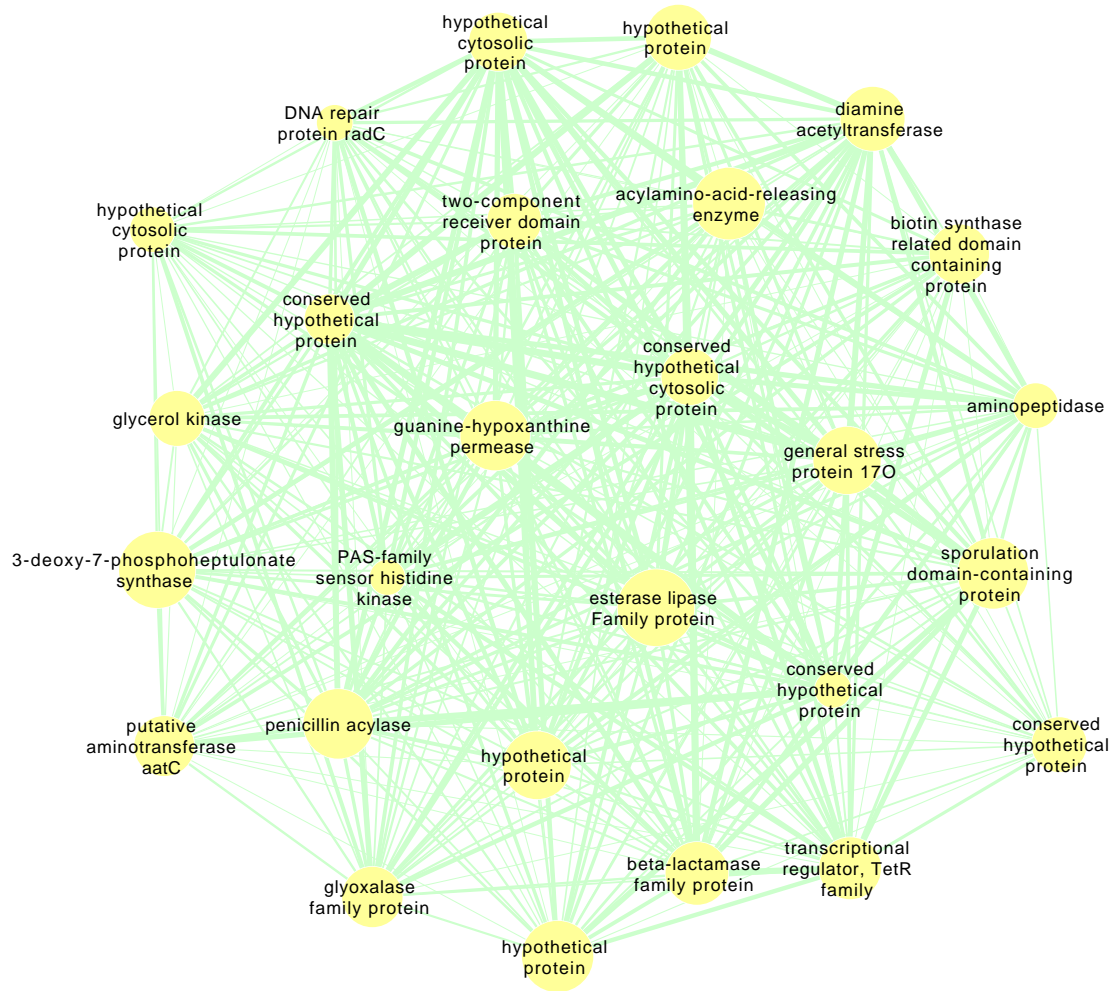

Supplement: Additional file 13: Figure S4 — Co-expression network topologies of all 76 modules. [file 1471-2164-14-450-S13.zip › FigureS4/lightgreen.pdf]

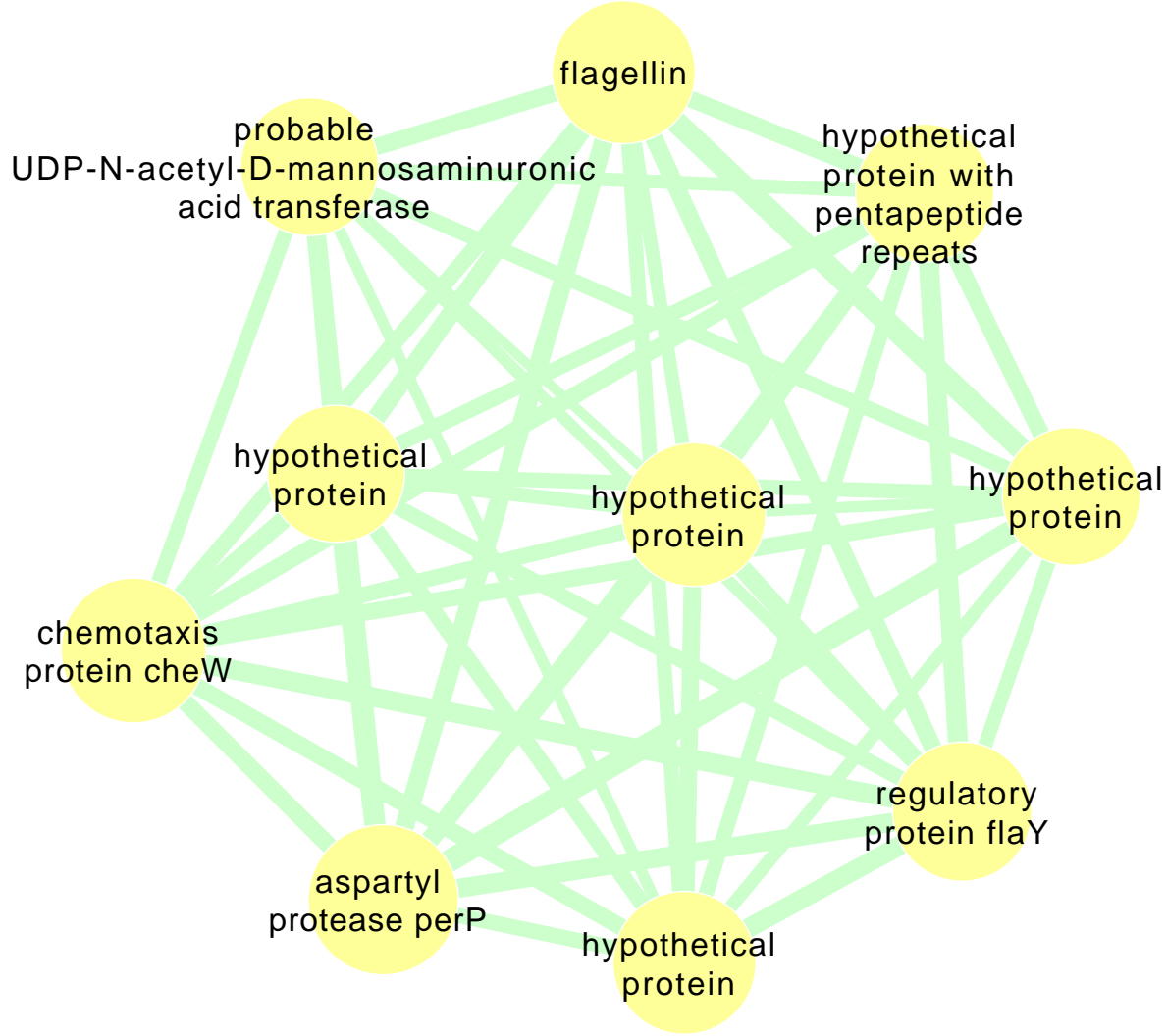

Supplement: Additional file 13: Figure S4 — Co-expression network topologies of all 76 modules. [file 1471-2164-14-450-S13.zip › FigureS4/lightpink4.pdf]

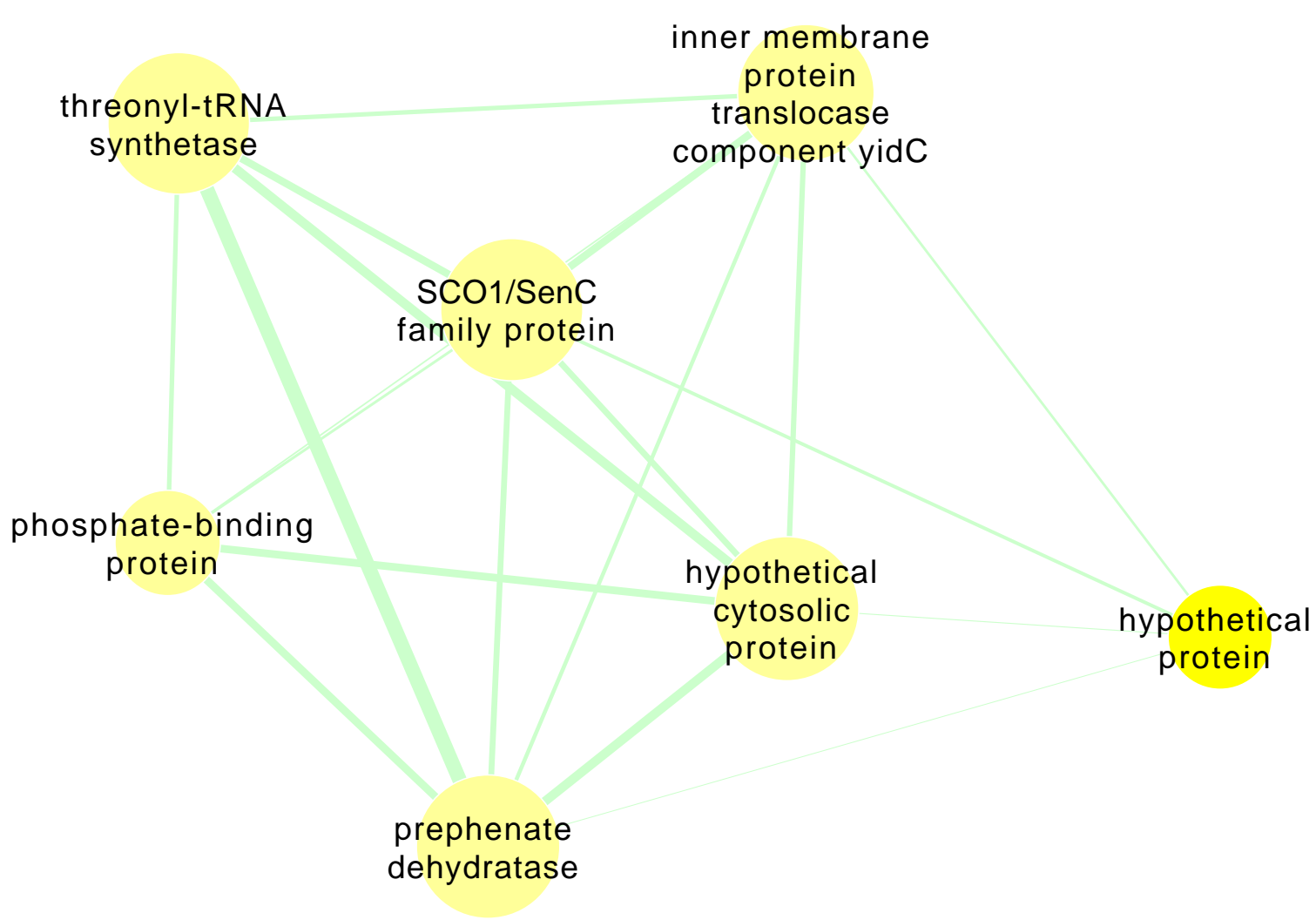

Supplement: Additional file 13: Figure S4 — Co-expression network topologies of all 76 modules. [file 1471-2164-14-450-S13.zip › FigureS4/lightsteelblue.pdf]

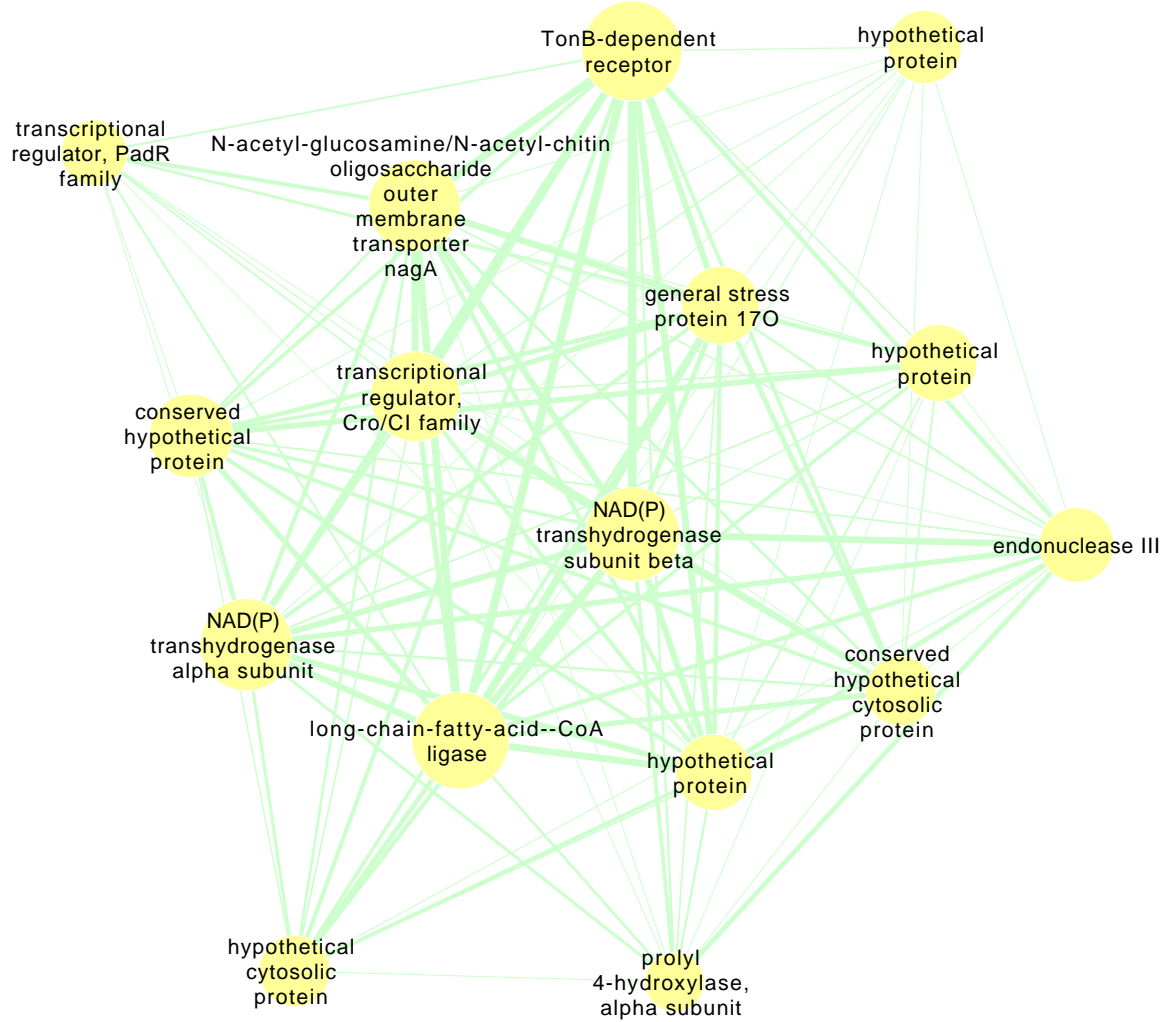

Supplement: Additional file 13: Figure S4 — Co-expression network topologies of all 76 modules. [file 1471-2164-14-450-S13.zip › FigureS4/lightsteelblue1.pdf]

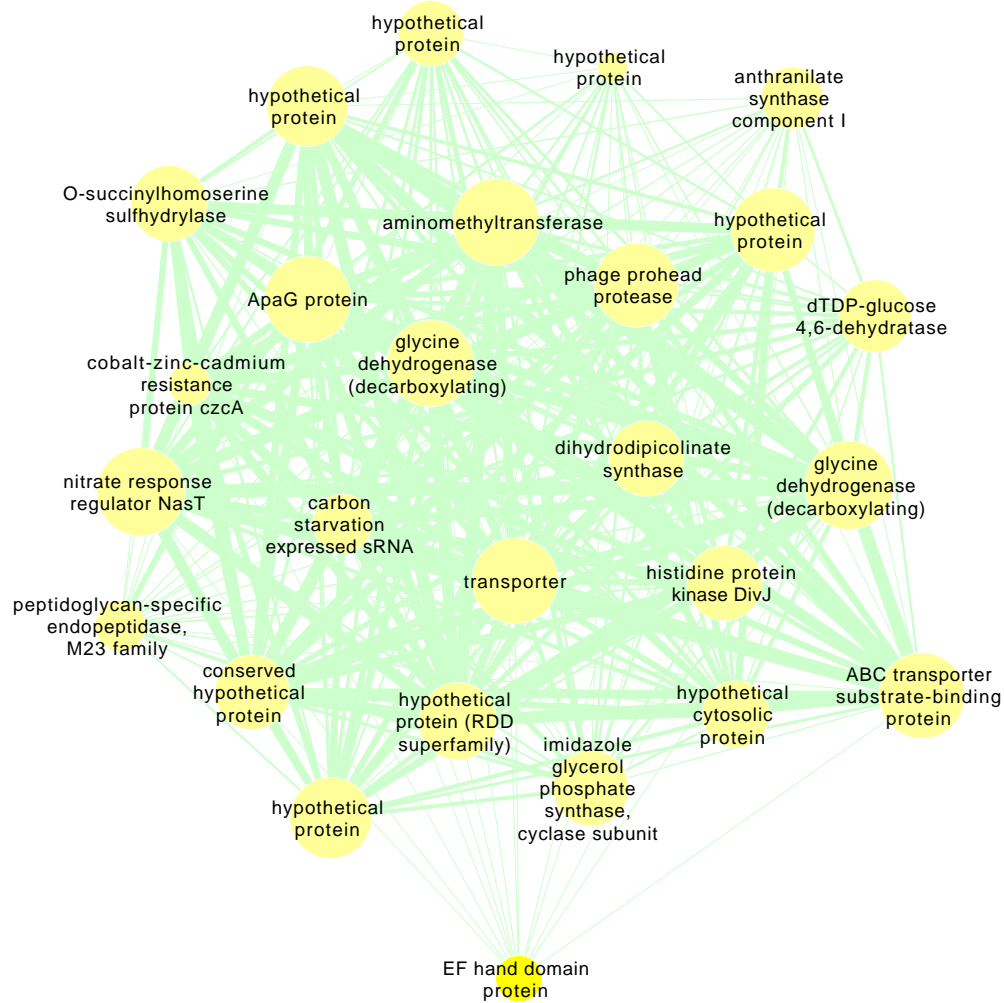

Supplement: Additional file 13: Figure S4 — Co-expression network topologies of all 76 modules. [file 1471-2164-14-450-S13.zip › FigureS4/lightyellow.pdf]

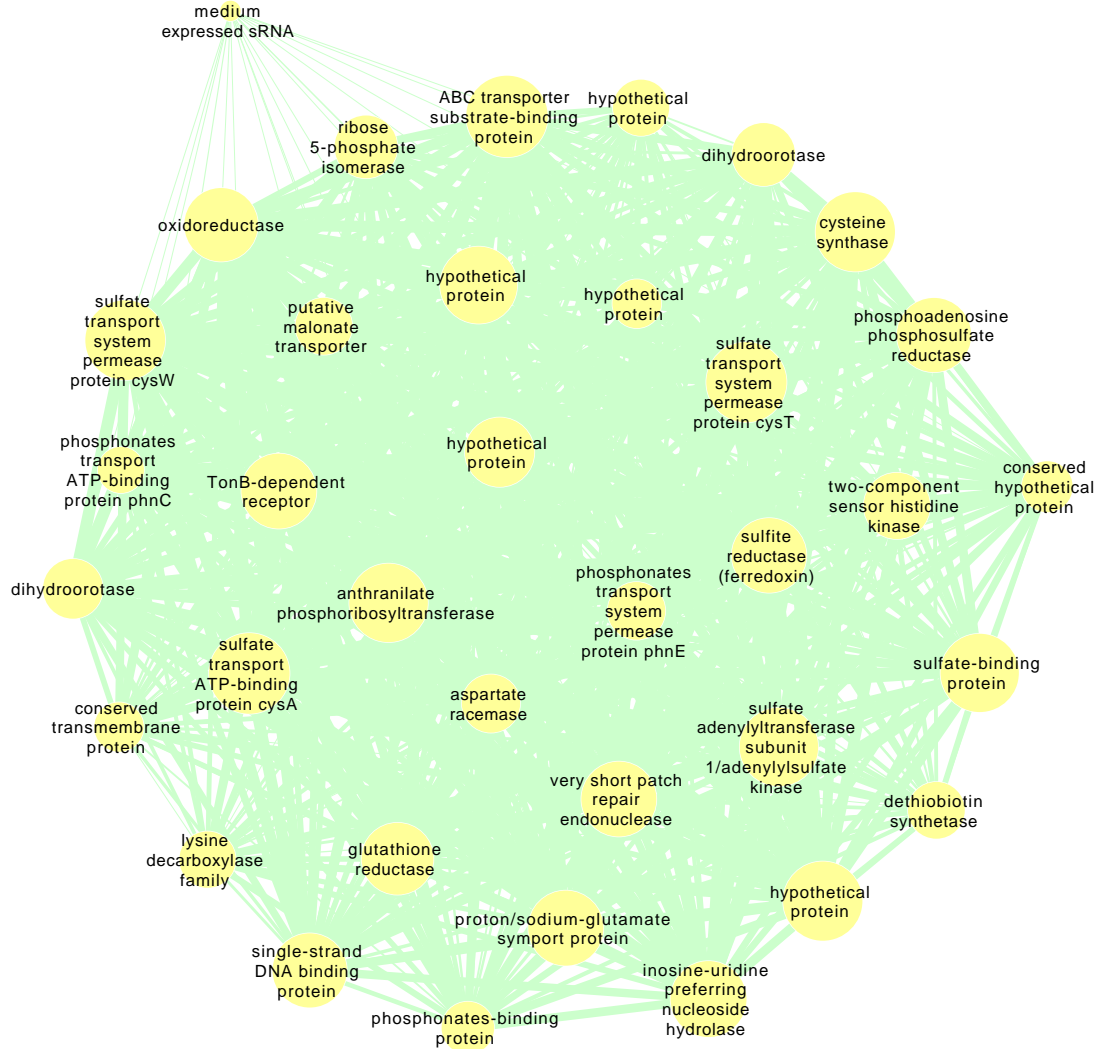

Supplement: Additional file 13: Figure S4 — Co-expression network topologies of all 76 modules. [file 1471-2164-14-450-S13.zip › FigureS4/magenta.pdf]

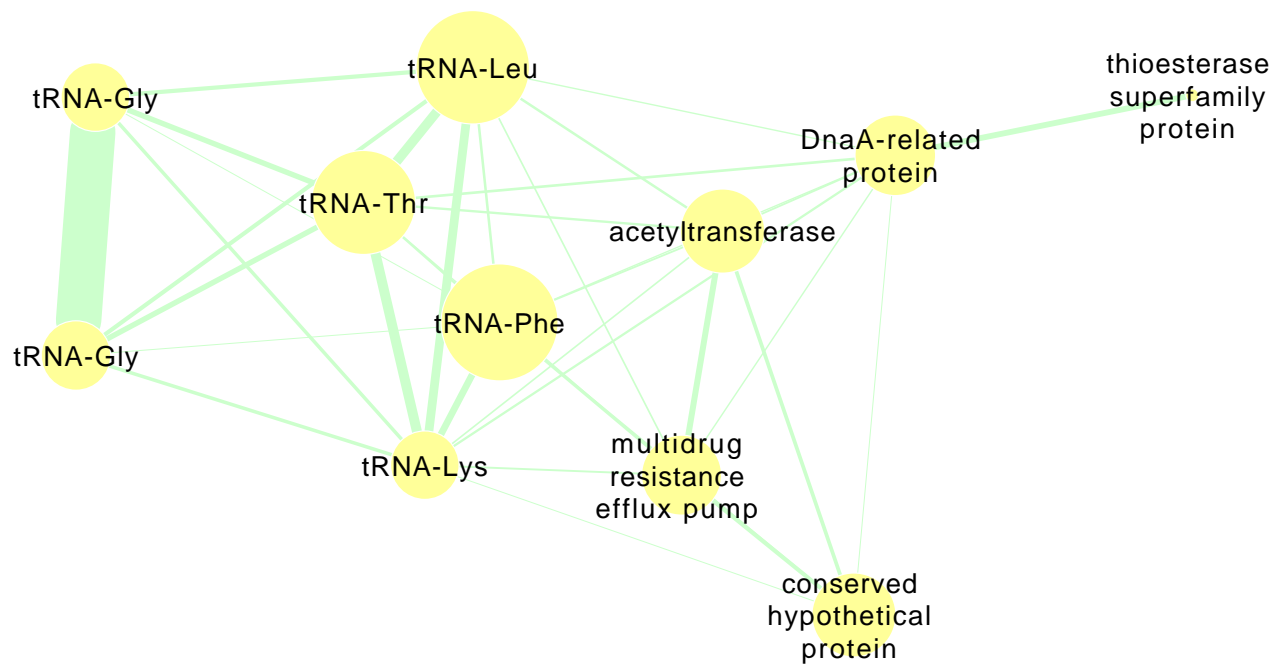

Supplement: Additional file 13: Figure S4 — Co-expression network topologies of all 76 modules. [file 1471-2164-14-450-S13.zip › FigureS4/maroon.pdf]

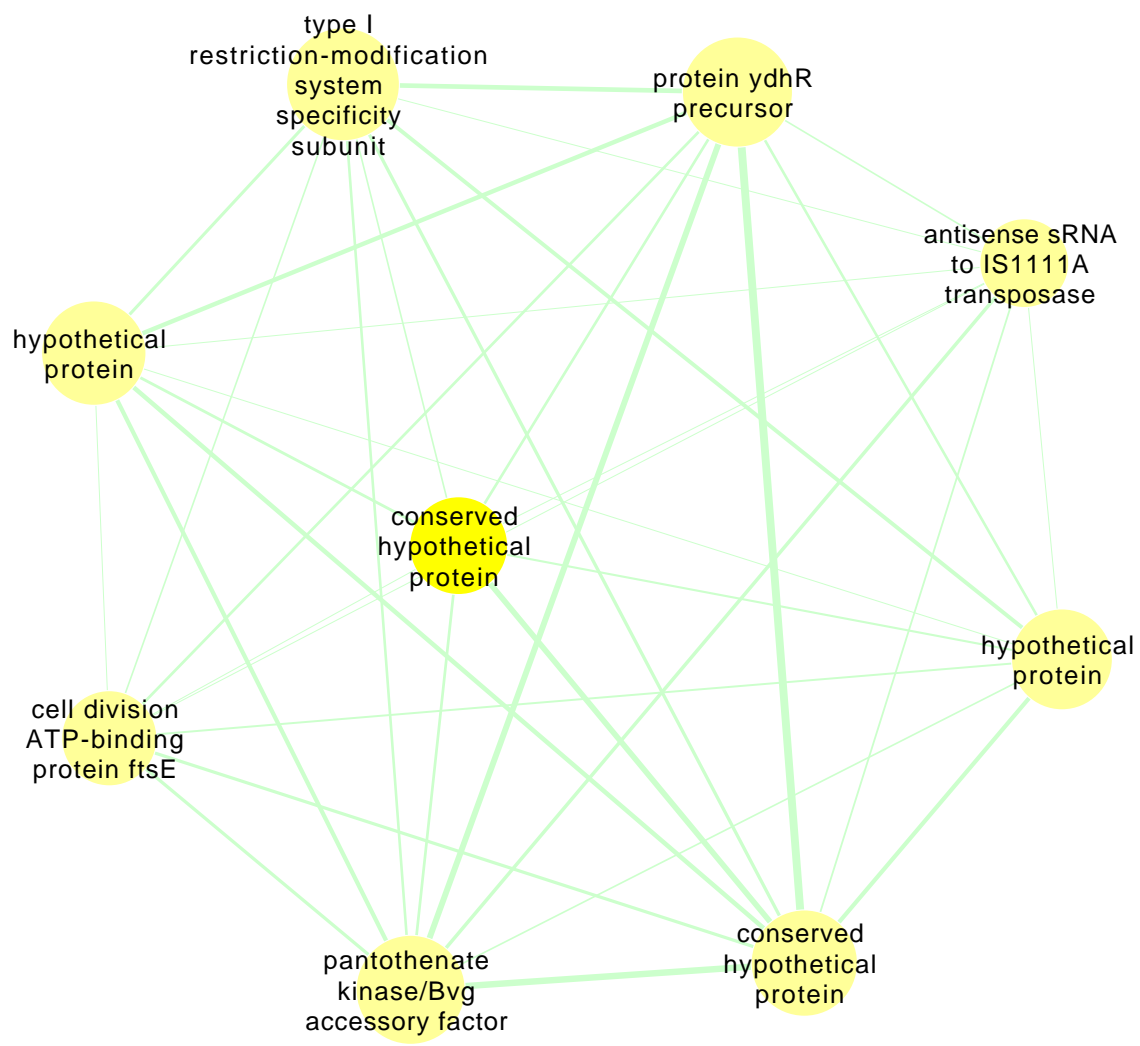

Supplement: Additional file 13: Figure S4 — Co-expression network topologies of all 76 modules. [file 1471-2164-14-450-S13.zip › FigureS4/mediumorchid.pdf]

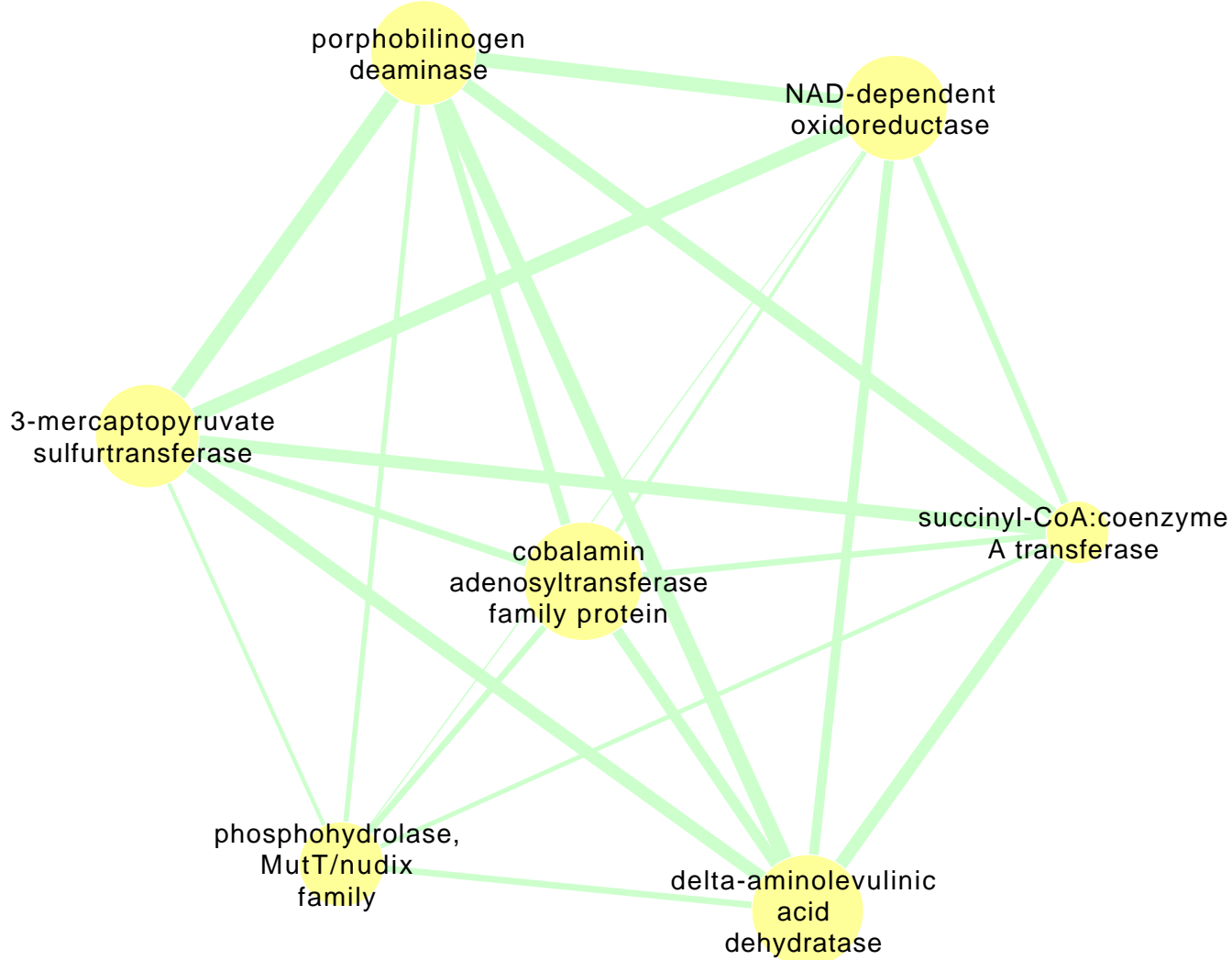

Supplement: Additional file 13: Figure S4 — Co-expression network topologies of all 76 modules. [file 1471-2164-14-450-S13.zip › FigureS4/mediumpurple2.pdf]

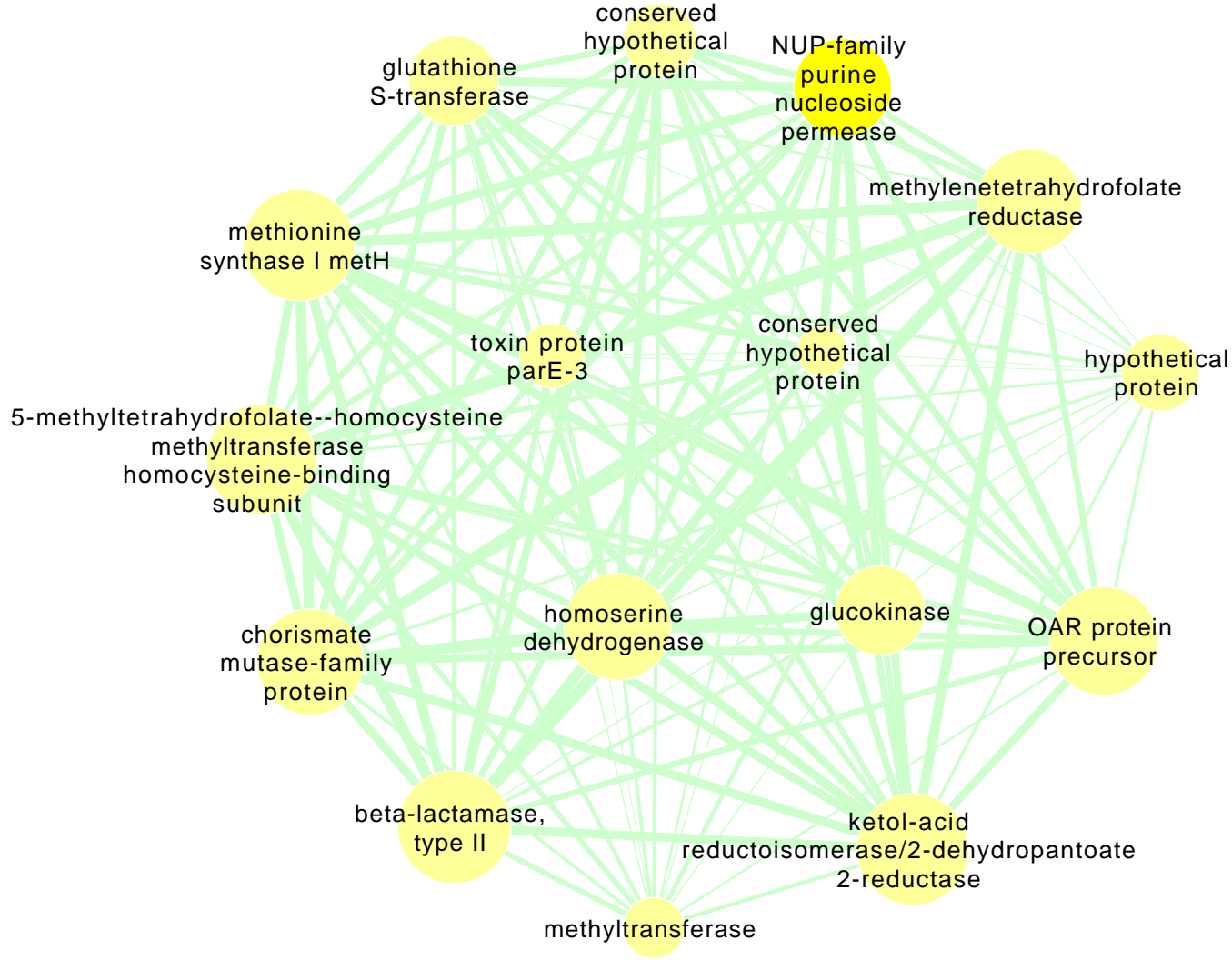

Supplement: Additional file 13: Figure S4 — Co-expression network topologies of all 76 modules. [file 1471-2164-14-450-S13.zip › FigureS4/mediumpurple3.pdf]

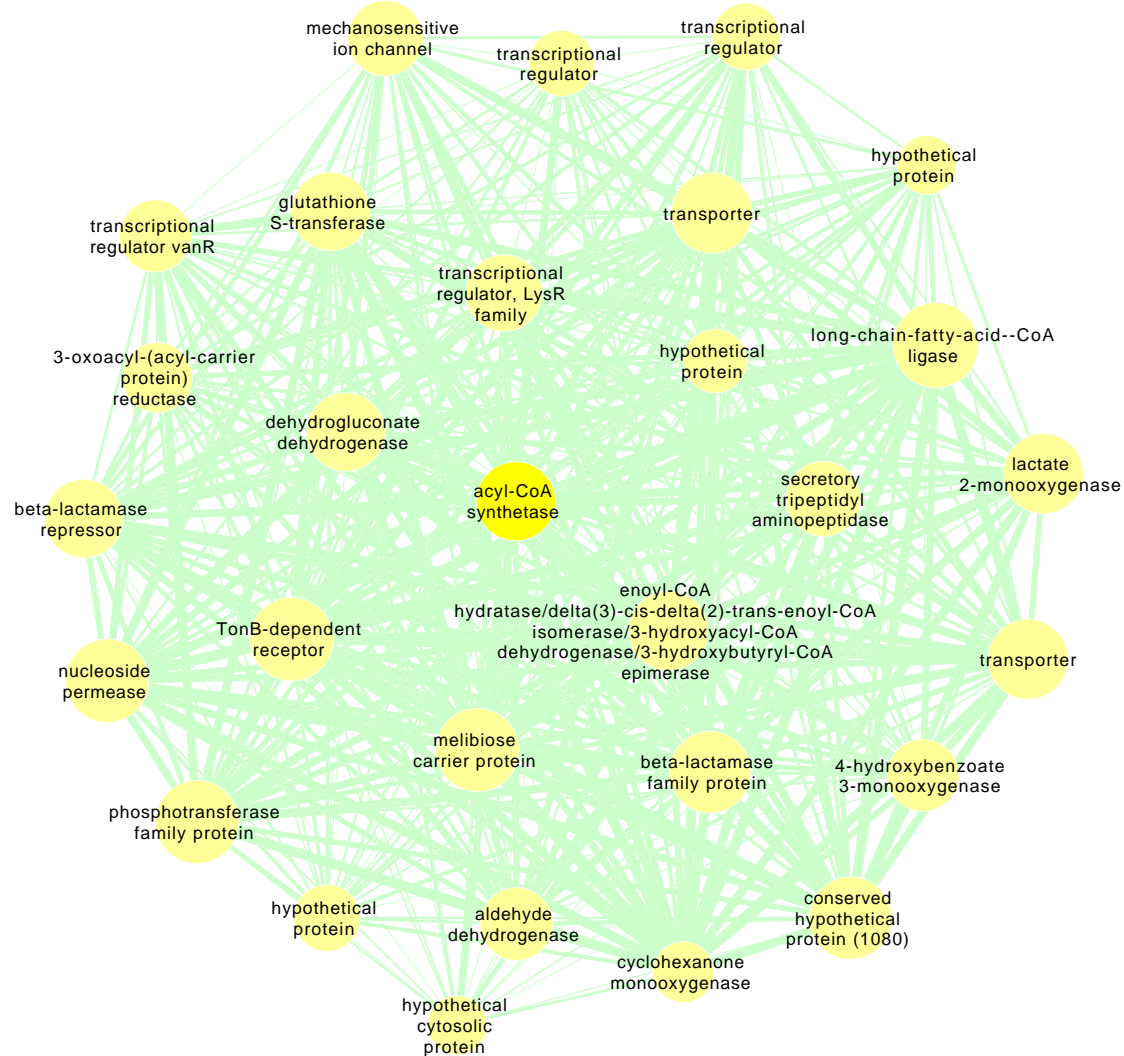

Supplement: Additional file 13: Figure S4 — Co-expression network topologies of all 76 modules. [file 1471-2164-14-450-S13.zip › FigureS4/midnightblue.pdf]

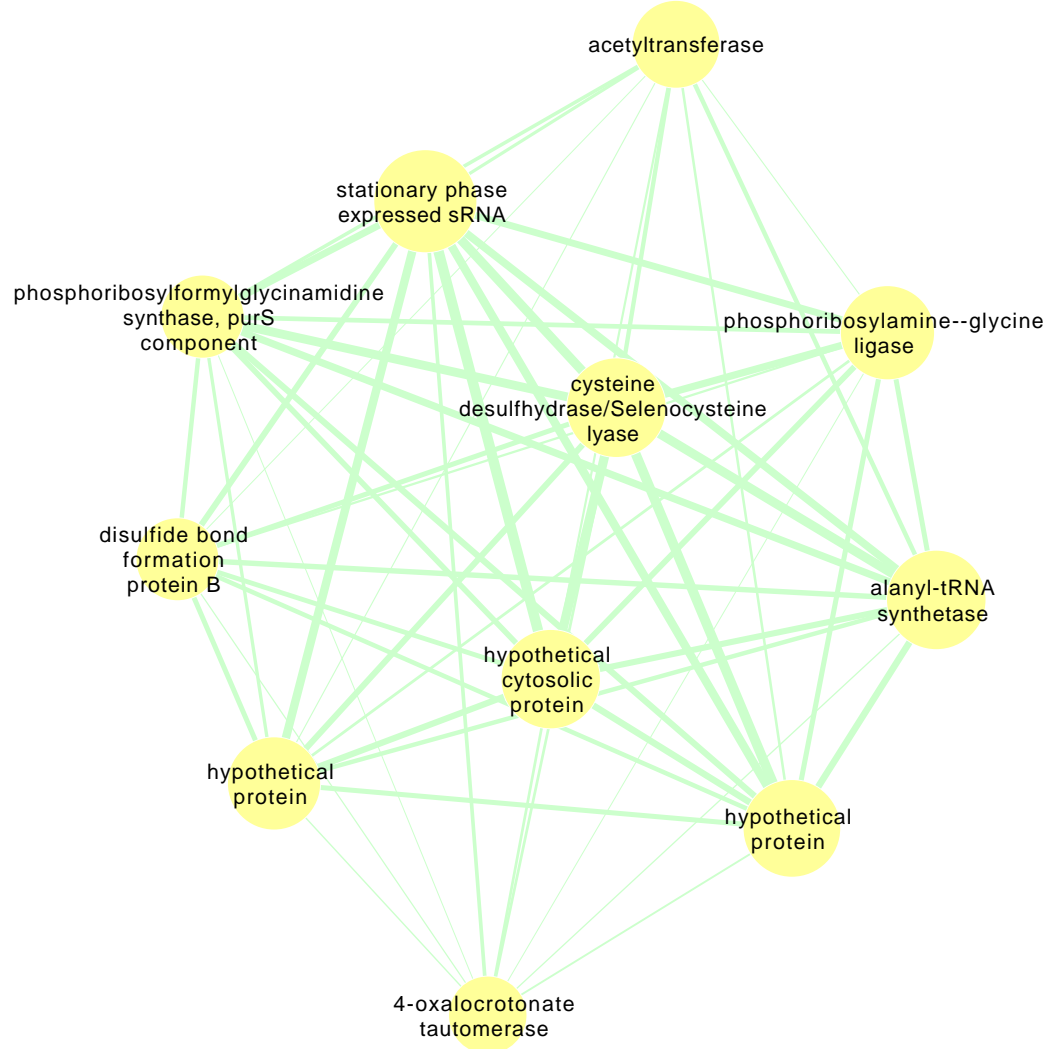

Supplement: Additional file 13: Figure S4 — Co-expression network topologies of all 76 modules. [file 1471-2164-14-450-S13.zip › FigureS4/navajowhite2.pdf]

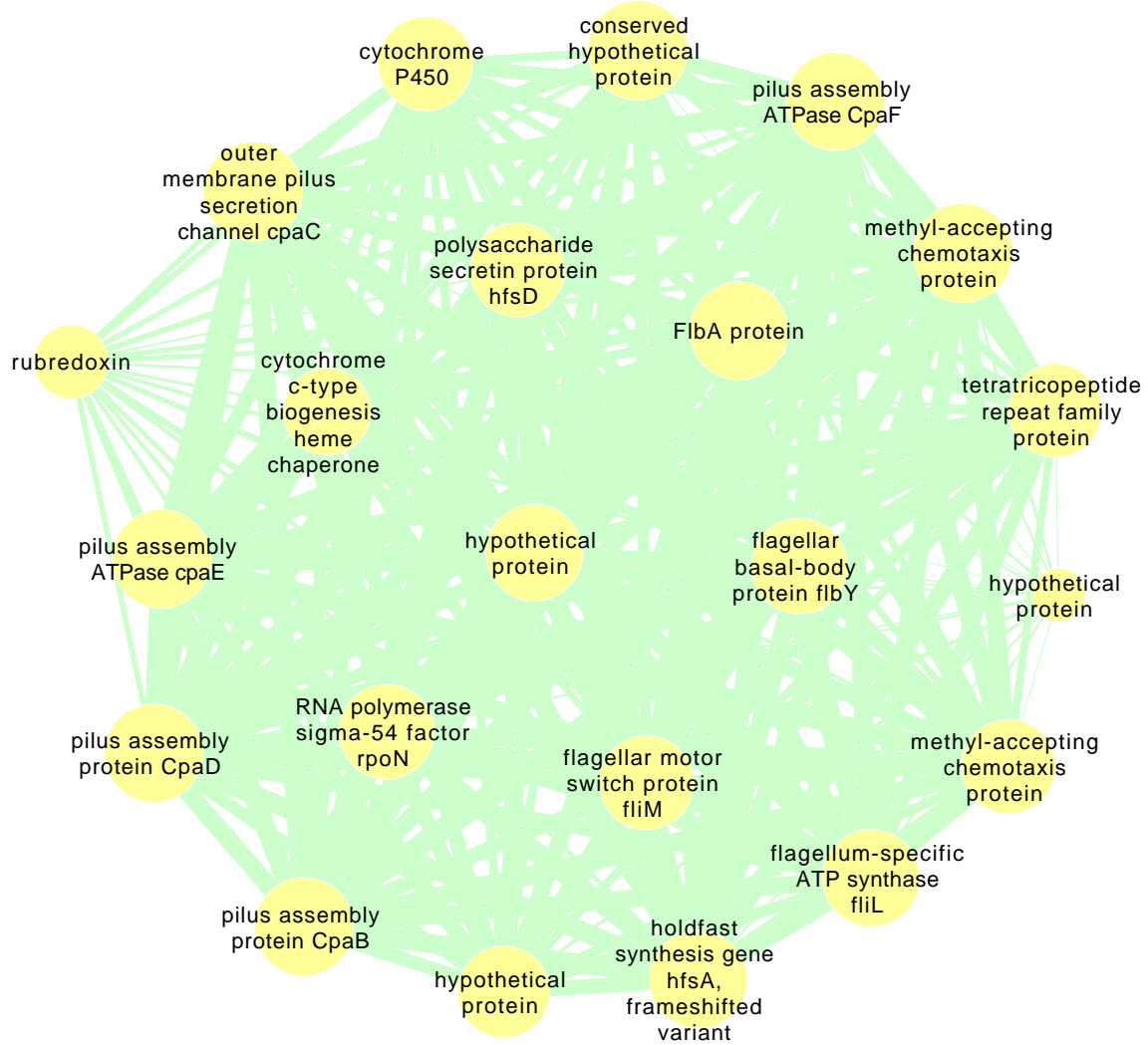

Supplement: Additional file 13: Figure S4 — Co-expression network topologies of all 76 modules. [file 1471-2164-14-450-S13.zip › FigureS4/orange.pdf]

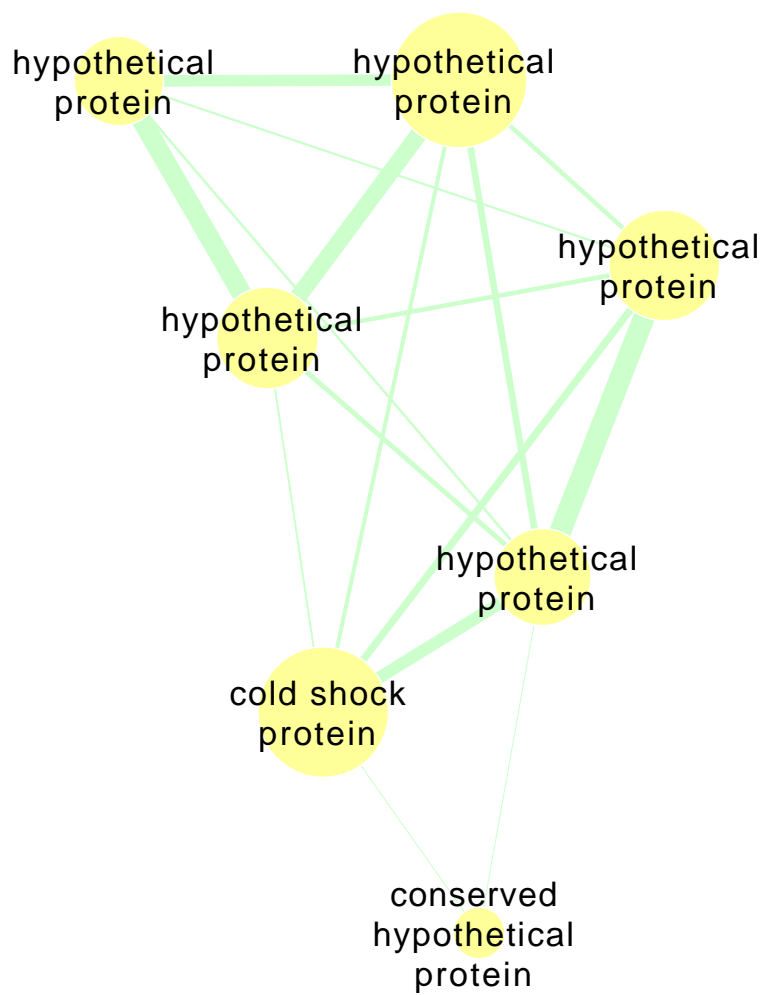

Supplement: Additional file 13: Figure S4 — Co-expression network topologies of all 76 modules. [file 1471-2164-14-450-S13.zip › FigureS4/orangered3.pdf]

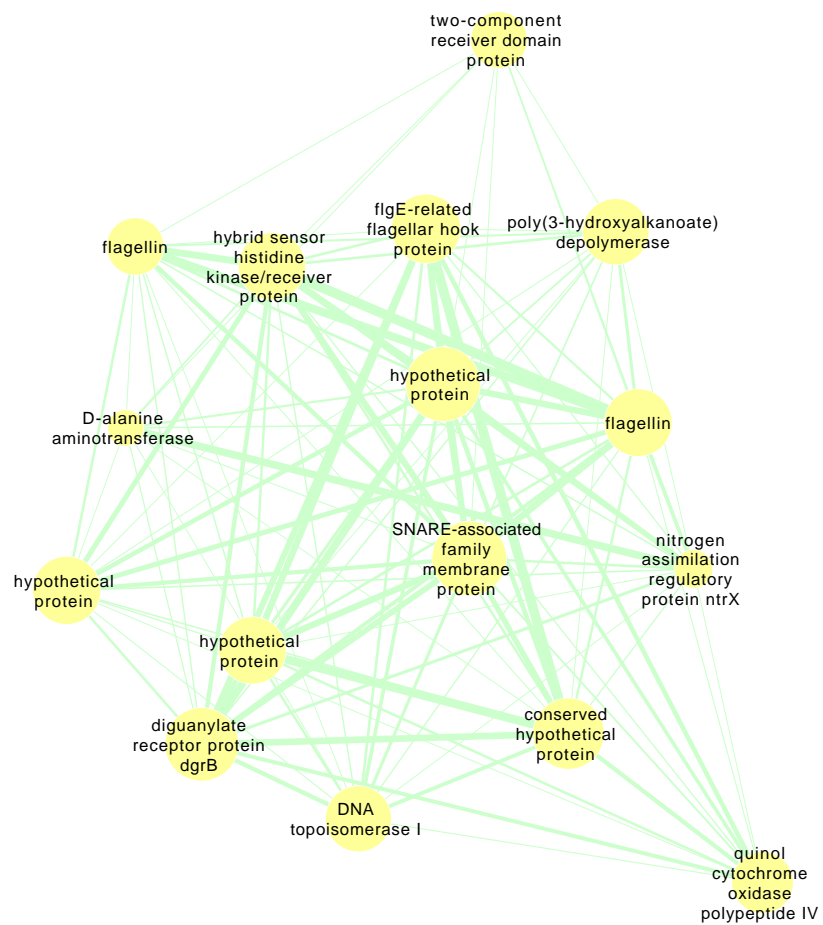

Supplement: Additional file 13: Figure S4 — Co-expression network topologies of all 76 modules. [file 1471-2164-14-450-S13.zip › FigureS4/orangered4.pdf]

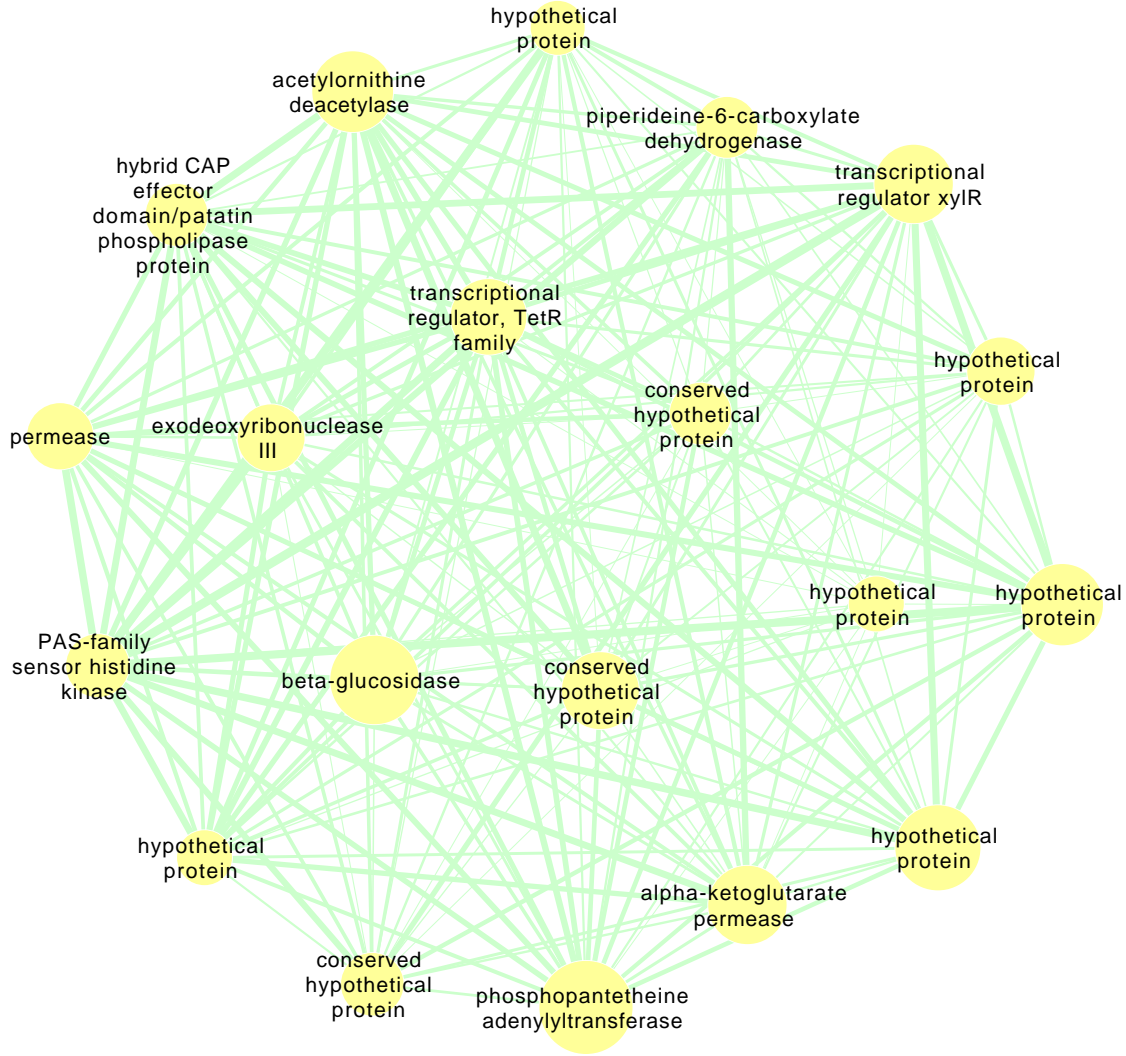

Supplement: Additional file 13: Figure S4 — Co-expression network topologies of all 76 modules. [file 1471-2164-14-450-S13.zip › FigureS4/paleturquoise.pdf]

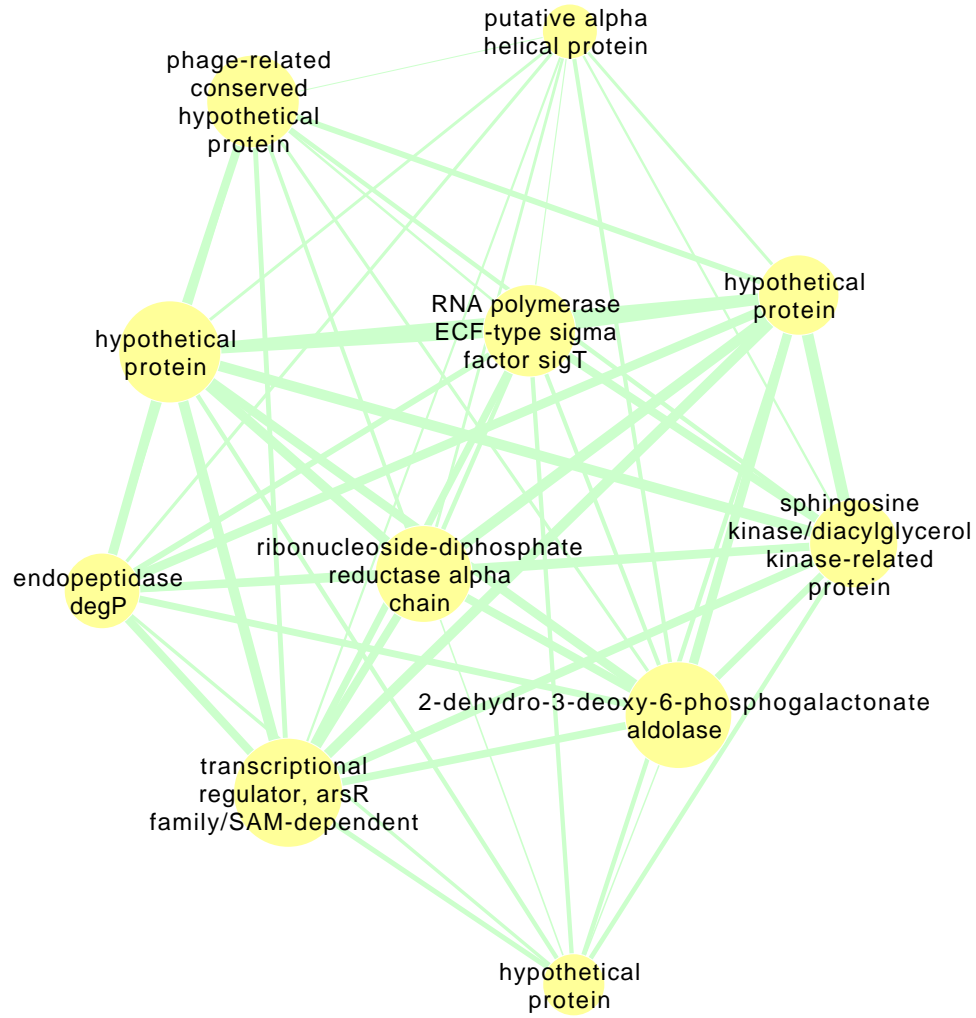

Supplement: Additional file 13: Figure S4 — Co-expression network topologies of all 76 modules. [file 1471-2164-14-450-S13.zip › FigureS4/palevioletred3.pdf]

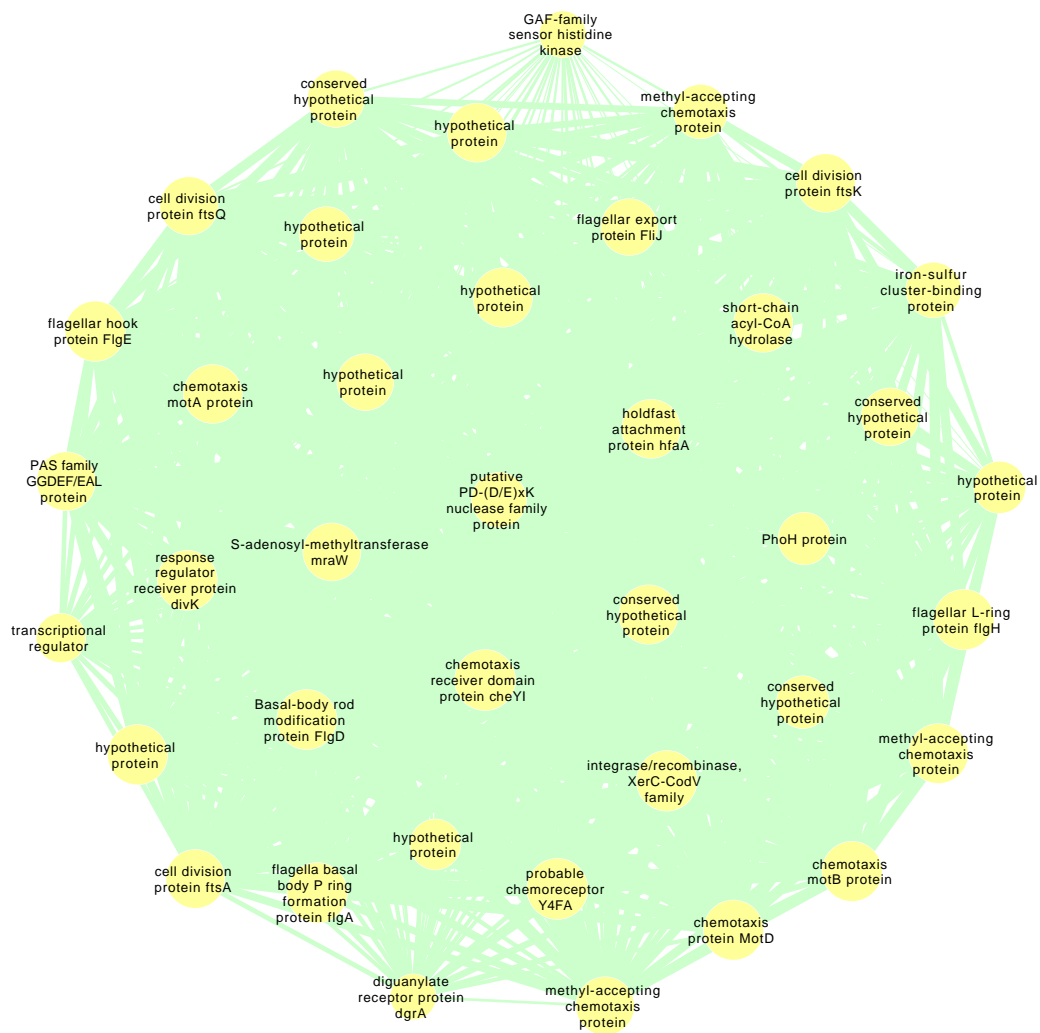

Supplement: Additional file 13: Figure S4 — Co-expression network topologies of all 76 modules. [file 1471-2164-14-450-S13.zip › FigureS4/pink.pdf]

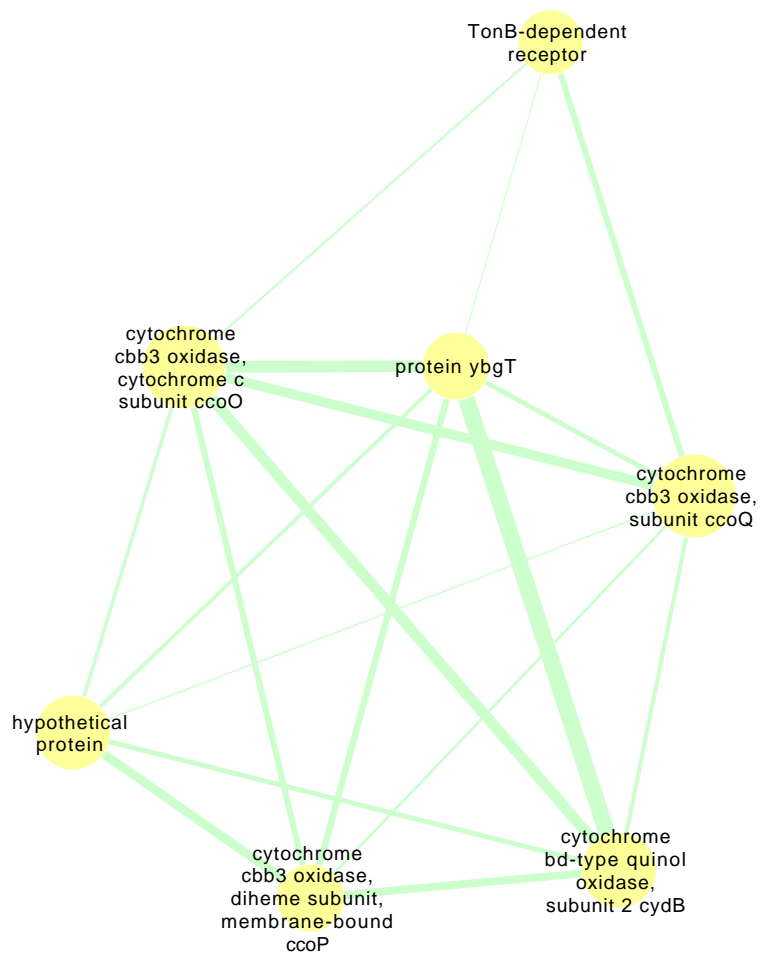

Supplement: Additional file 13: Figure S4 — Co-expression network topologies of all 76 modules. [file 1471-2164-14-450-S13.zip › FigureS4/plum.pdf]

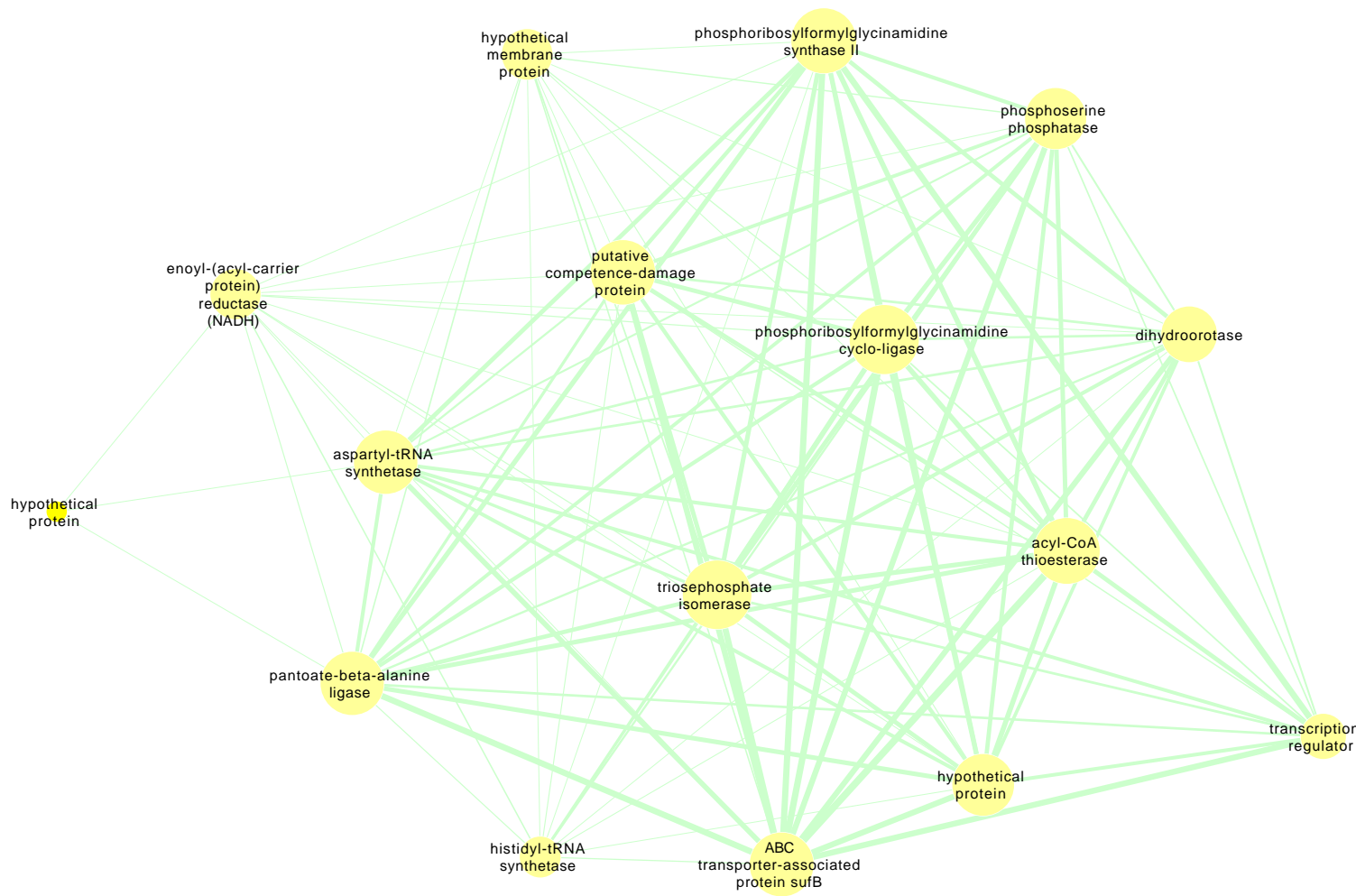

Supplement: Additional file 13: Figure S4 — Co-expression network topologies of all 76 modules. [file 1471-2164-14-450-S13.zip › FigureS4/plum1.pdf]

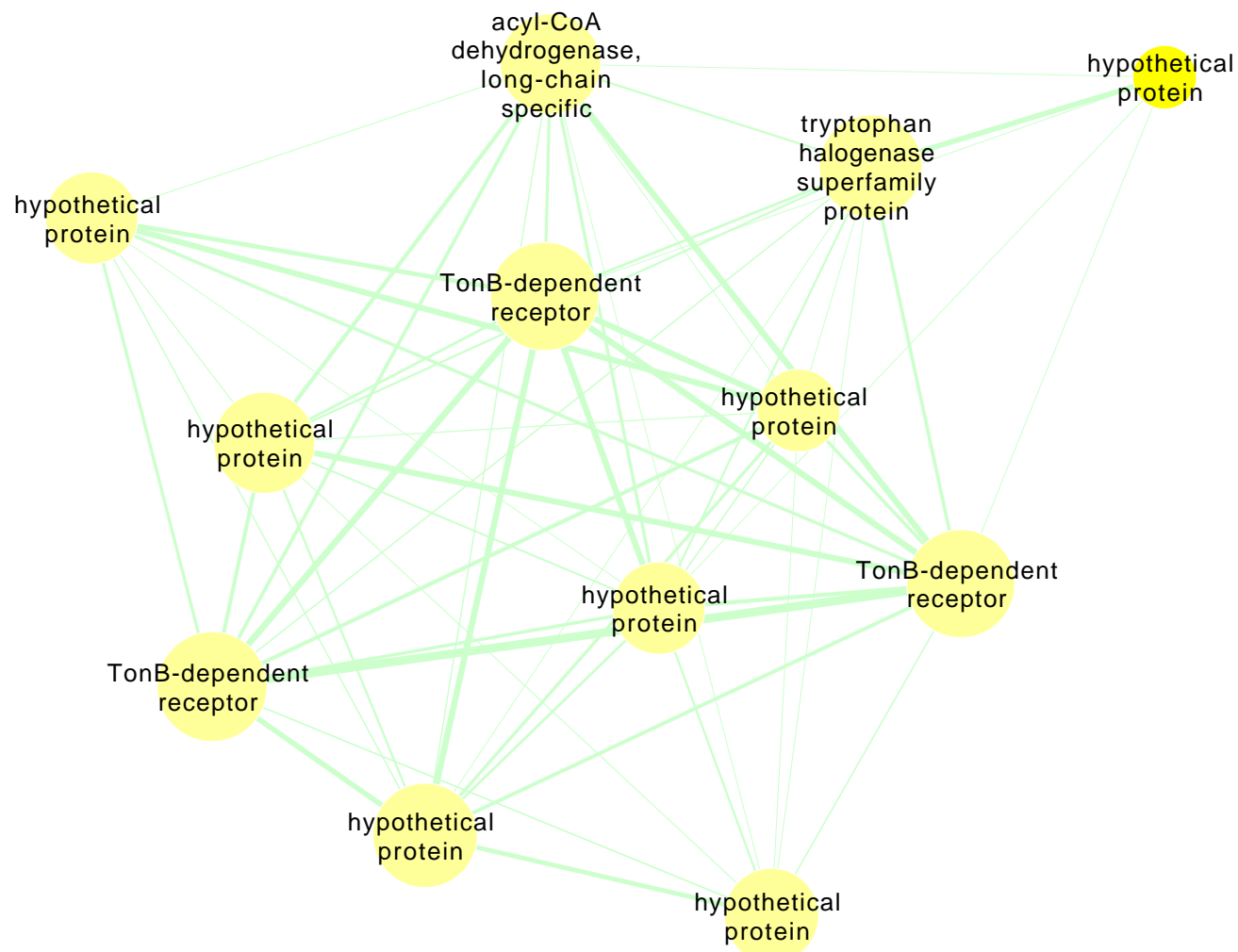

Supplement: Additional file 13: Figure S4 — Co-expression network topologies of all 76 modules. [file 1471-2164-14-450-S13.zip › FigureS4/plum2.pdf]

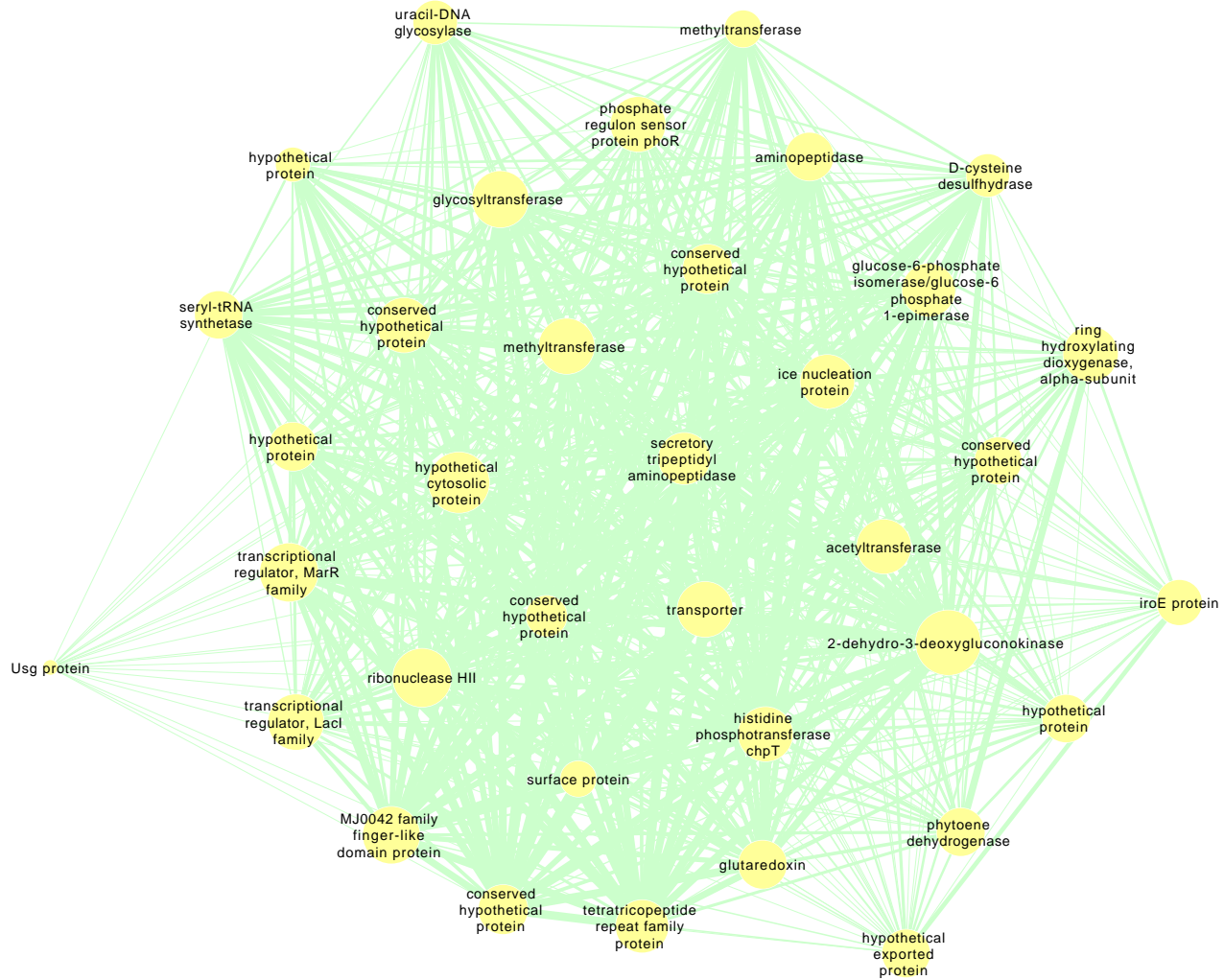

Supplement: Additional file 13: Figure S4 — Co-expression network topologies of all 76 modules. [file 1471-2164-14-450-S13.zip › FigureS4/purple.pdf]

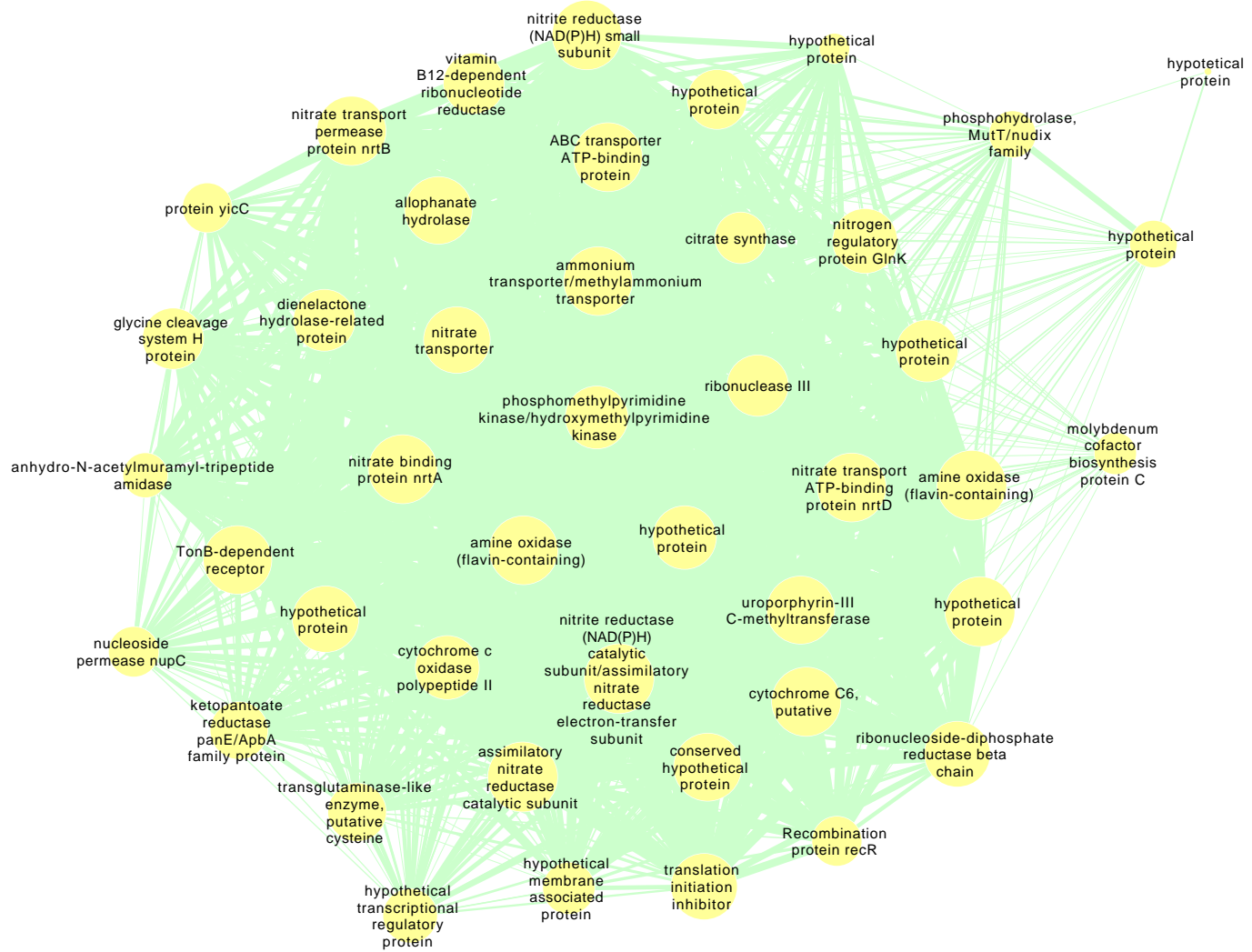

Supplement: Additional file 13: Figure S4 — Co-expression network topologies of all 76 modules. [file 1471-2164-14-450-S13.zip › FigureS4/red.pdf]

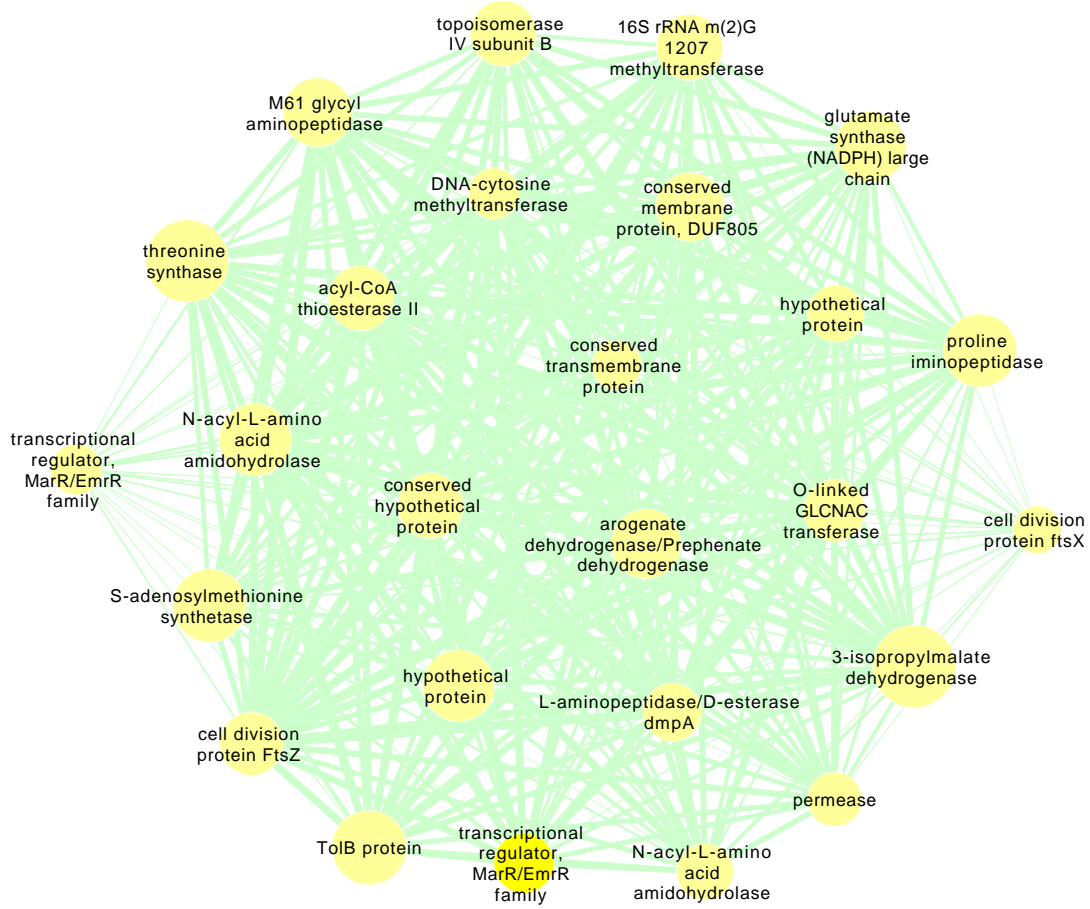

Supplement: Additional file 13: Figure S4 — Co-expression network topologies of all 76 modules. [file 1471-2164-14-450-S13.zip › FigureS4/royalblue.pdf]

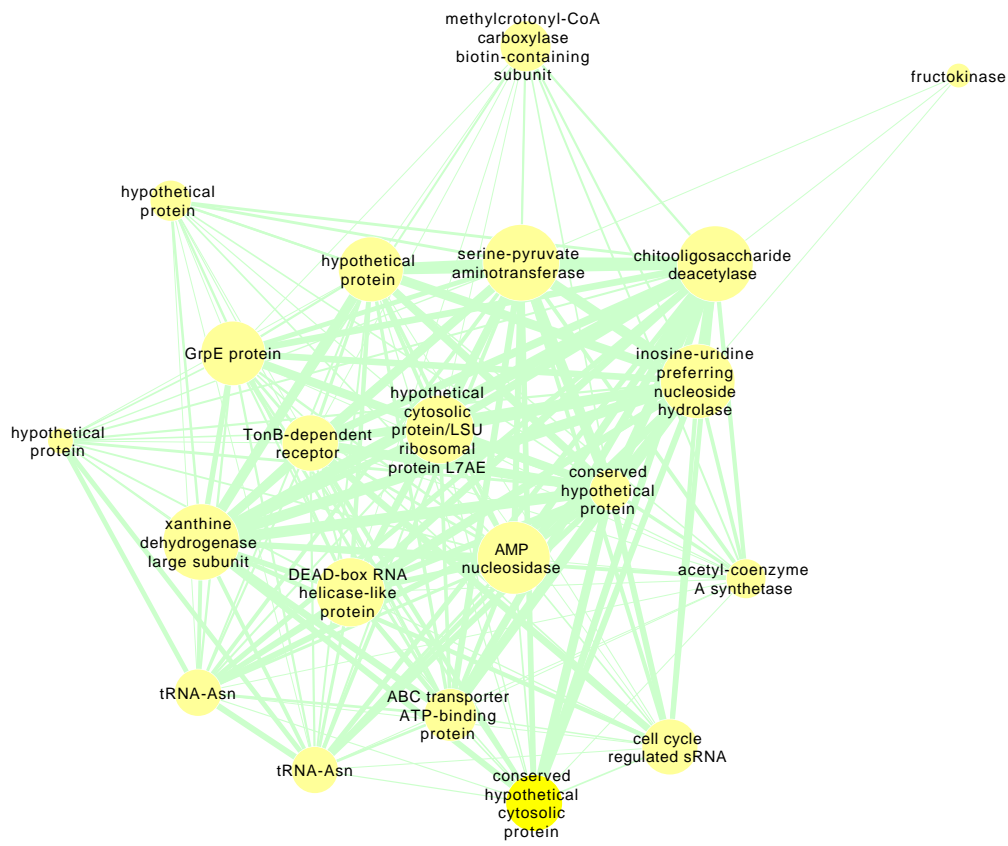

Supplement: Additional file 13: Figure S4 — Co-expression network topologies of all 76 modules. [file 1471-2164-14-450-S13.zip › FigureS4/saddlebrown.pdf]

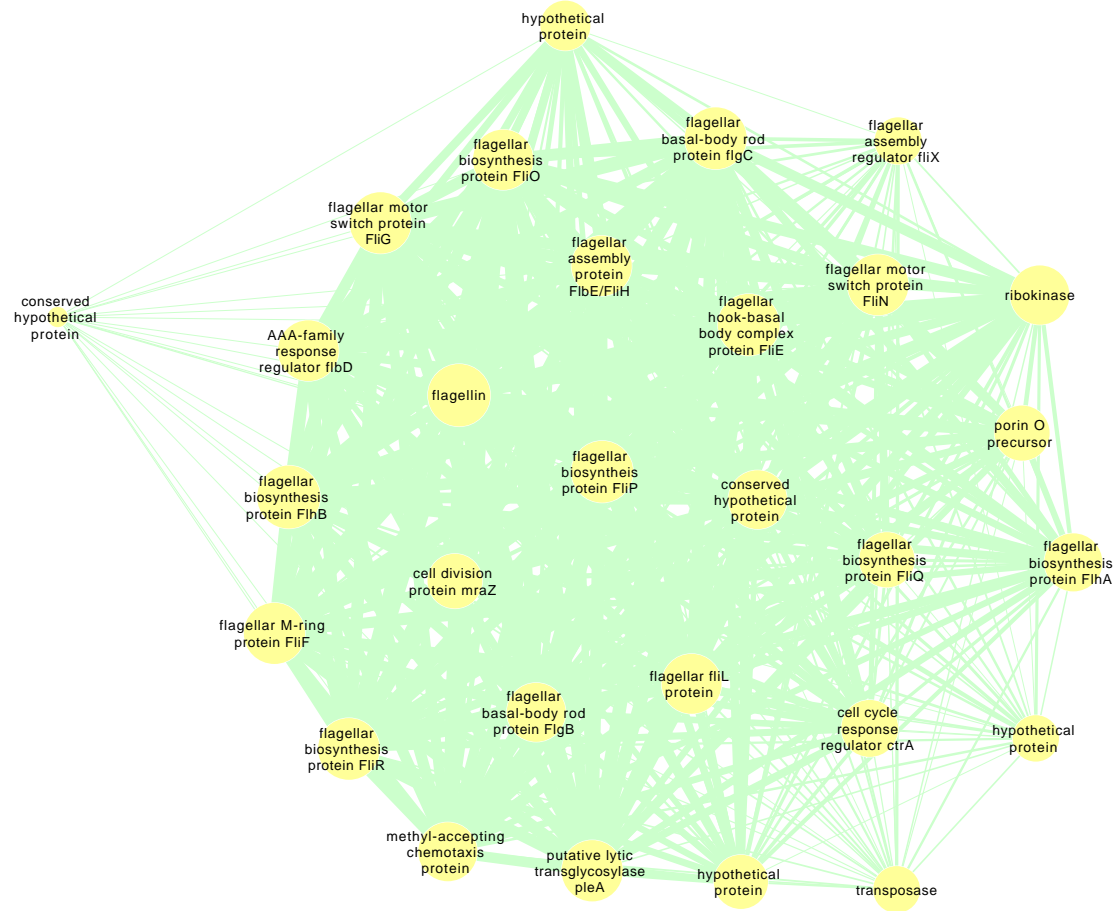

Supplement: Additional file 13: Figure S4 — Co-expression network topologies of all 76 modules. [file 1471-2164-14-450-S13.zip › FigureS4/salmon.pdf]

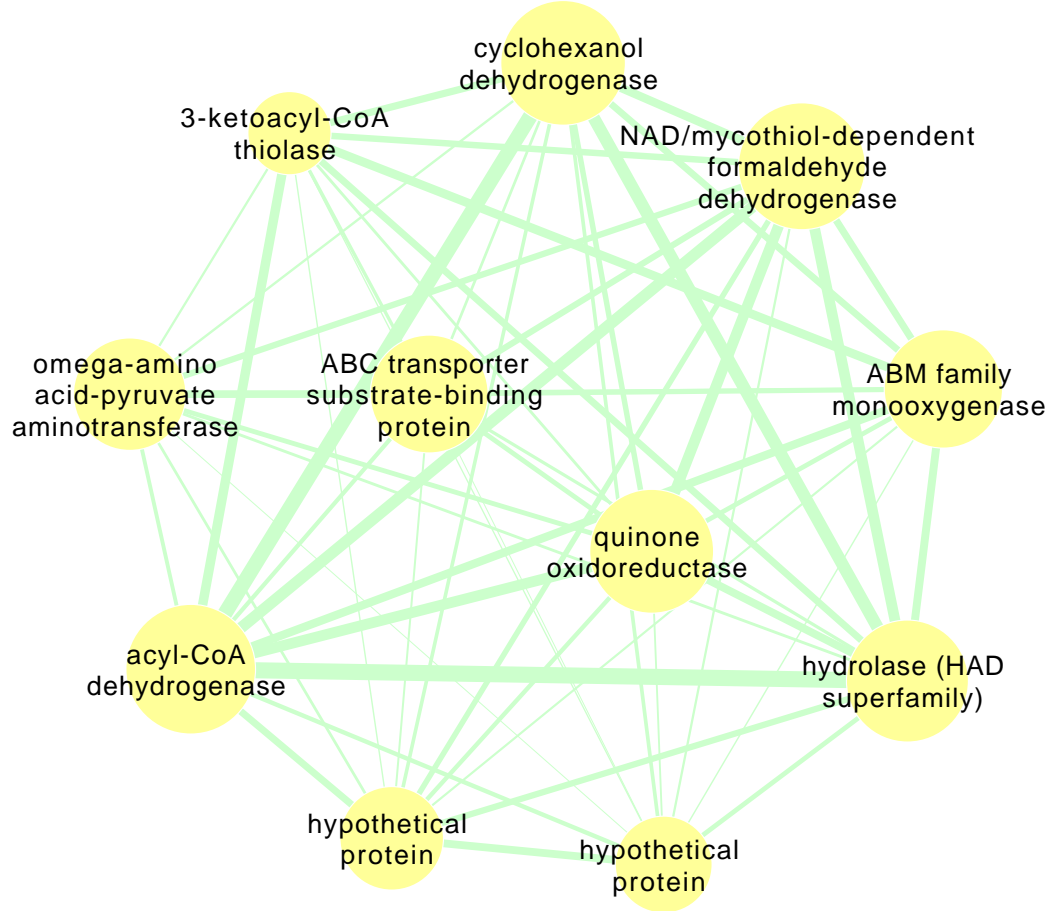

Supplement: Additional file 13: Figure S4 — Co-expression network topologies of all 76 modules. [file 1471-2164-14-450-S13.zip › FigureS4/salmon4.pdf]

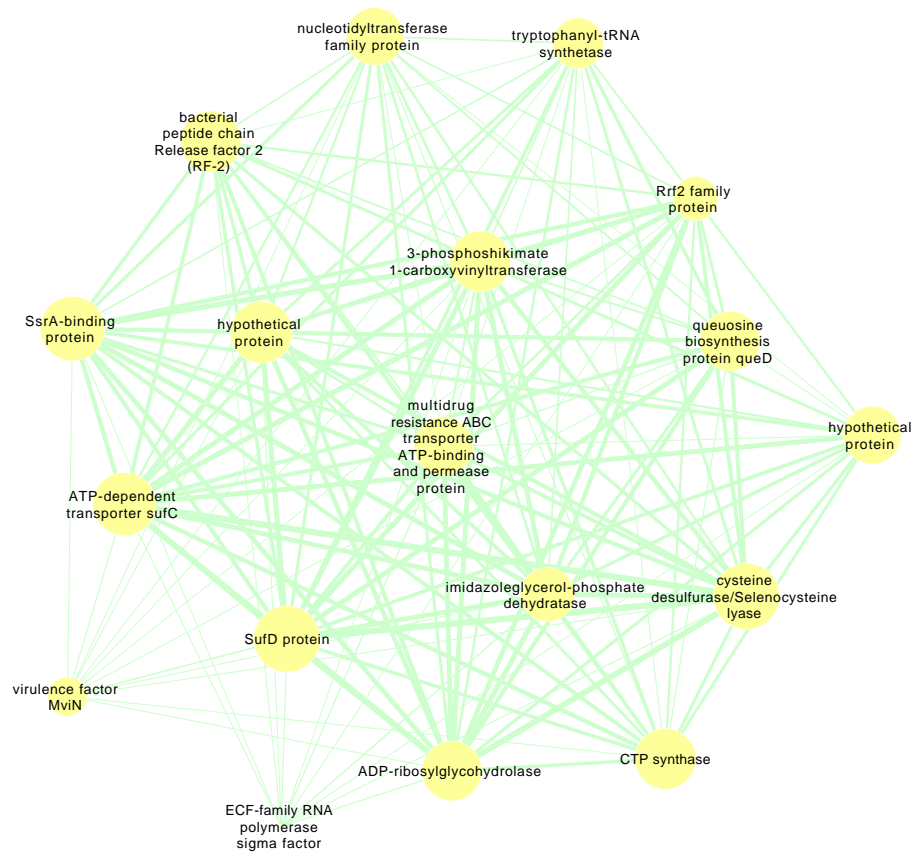

Supplement: Additional file 13: Figure S4 — Co-expression network topologies of all 76 modules. [file 1471-2164-14-450-S13.zip › FigureS4/sienna3.pdf]

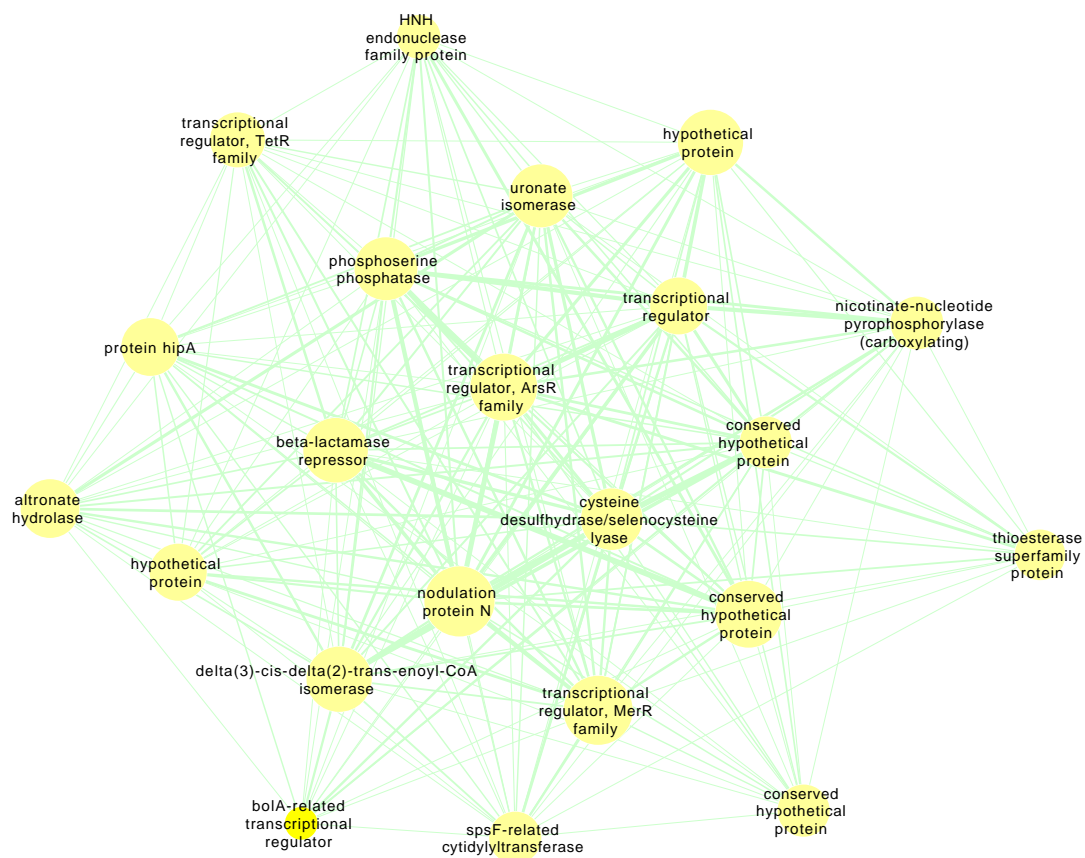

Supplement: Additional file 13: Figure S4 — Co-expression network topologies of all 76 modules. [file 1471-2164-14-450-S13.zip › FigureS4/skyblue.pdf]

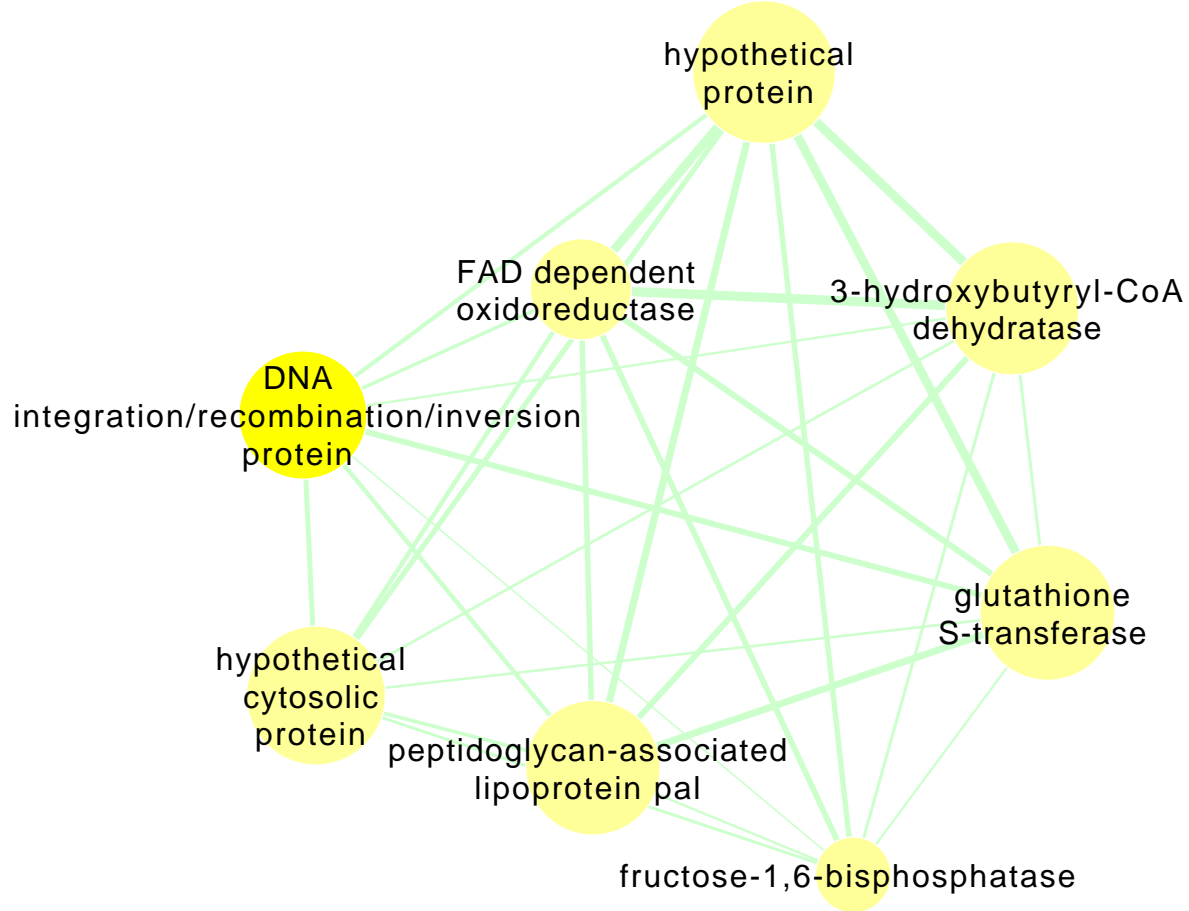

Supplement: Additional file 13: Figure S4 — Co-expression network topologies of all 76 modules. [file 1471-2164-14-450-S13.zip › FigureS4/skyblue1.pdf]

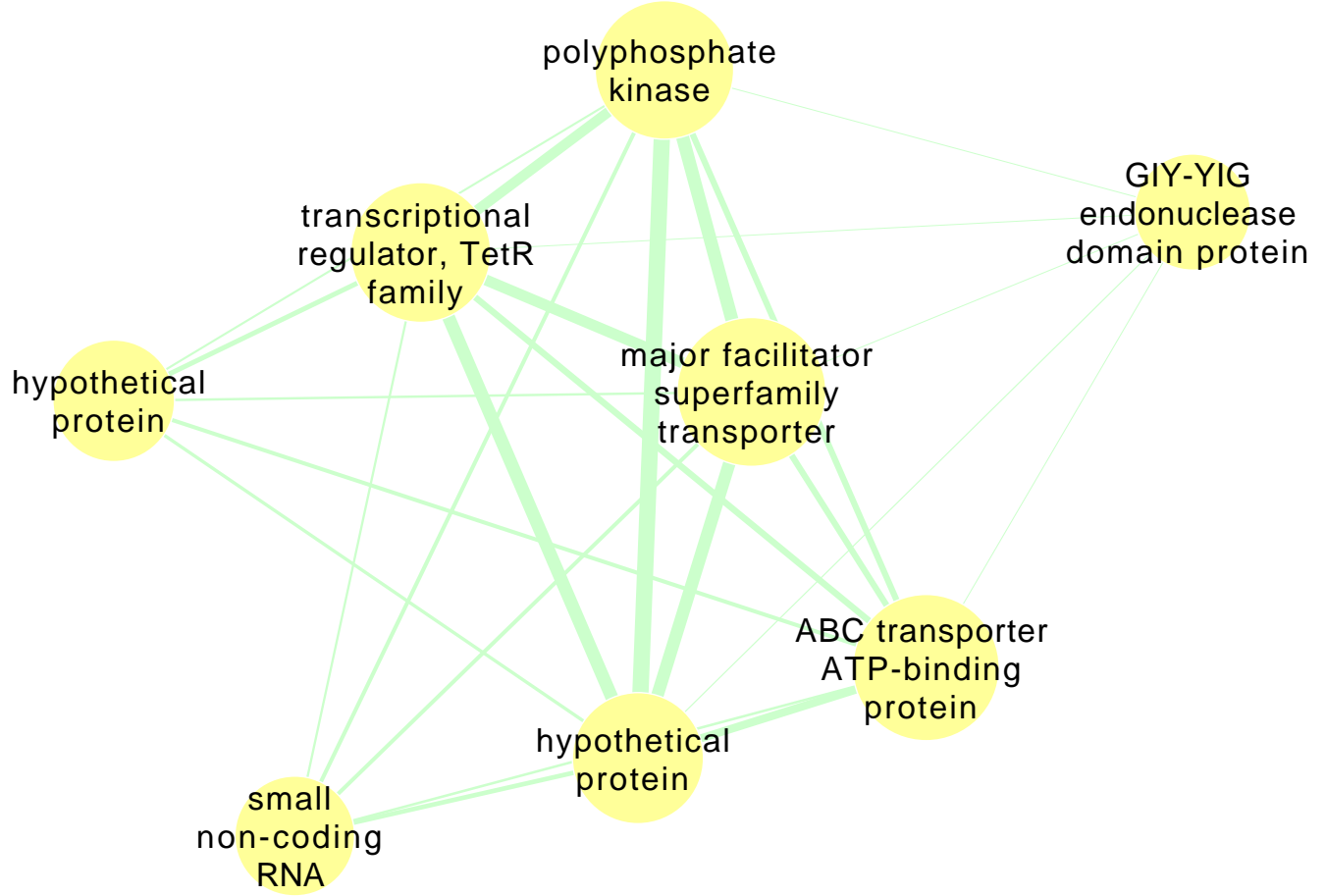

Supplement: Additional file 13: Figure S4 — Co-expression network topologies of all 76 modules. [file 1471-2164-14-450-S13.zip › FigureS4/skyblue2.pdf]

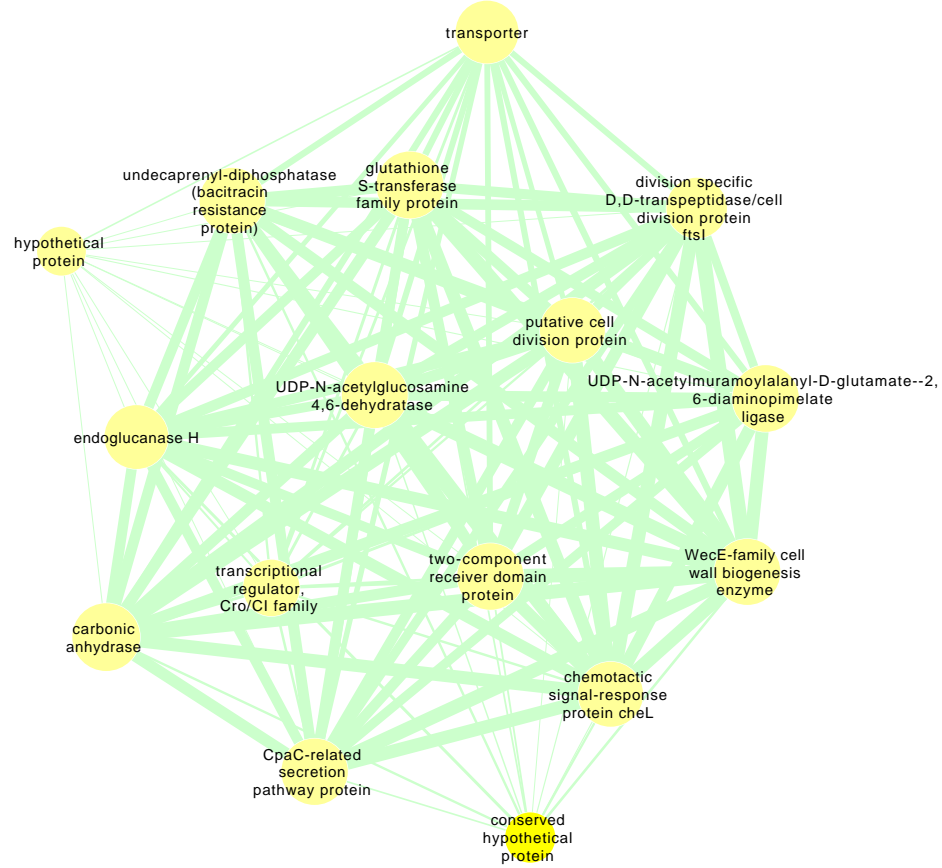

Supplement: Additional file 13: Figure S4 — Co-expression network topologies of all 76 modules. [file 1471-2164-14-450-S13.zip › FigureS4/skyblue3.pdf]

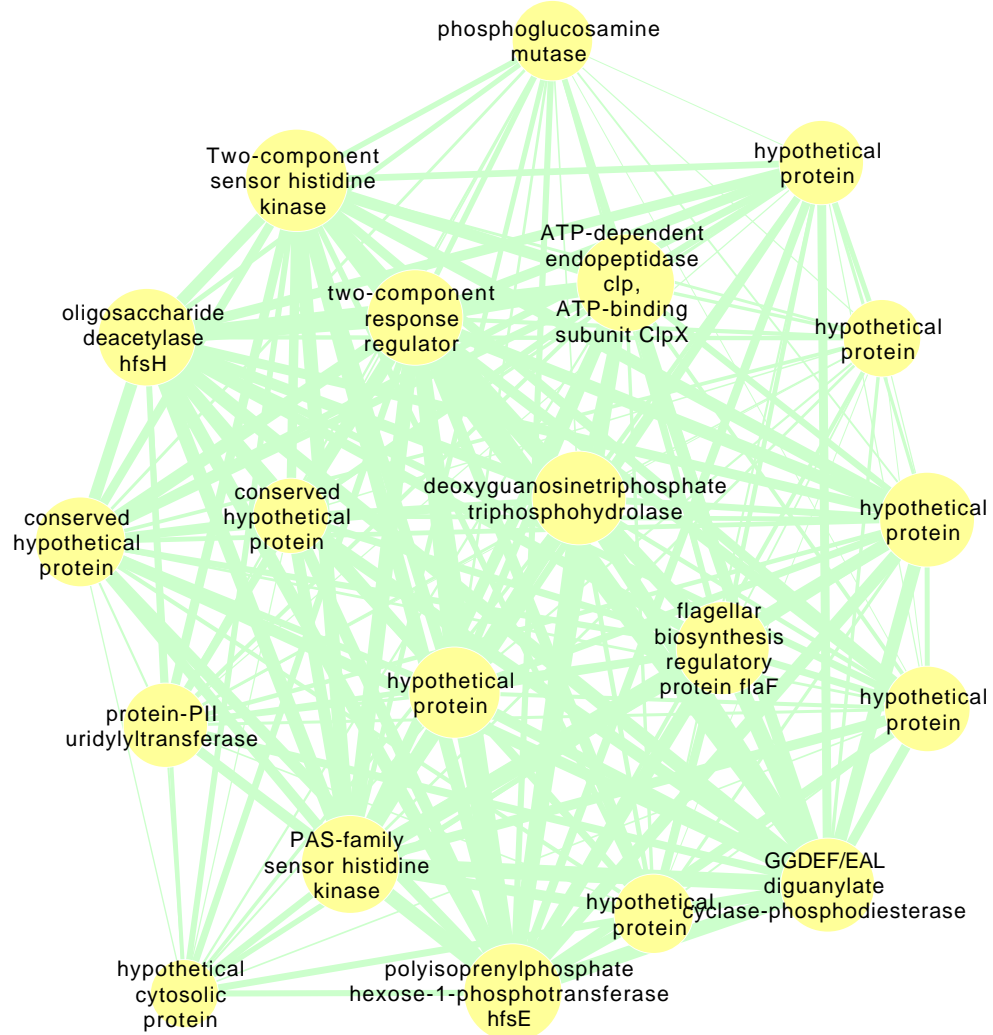

Supplement: Additional file 13: Figure S4 — Co-expression network topologies of all 76 modules. [file 1471-2164-14-450-S13.zip › FigureS4/steelblue.pdf]

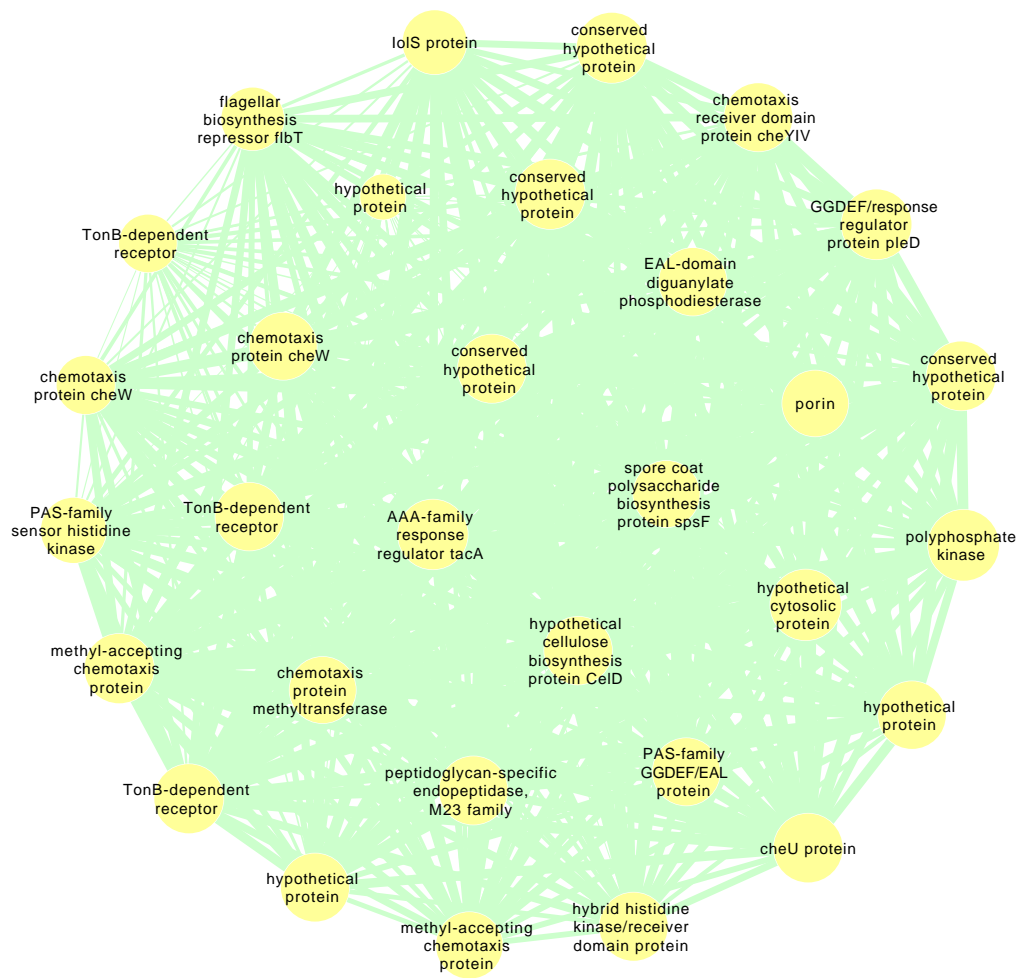

Supplement: Additional file 13: Figure S4 — Co-expression network topologies of all 76 modules. [file 1471-2164-14-450-S13.zip › FigureS4/tan.pdf]

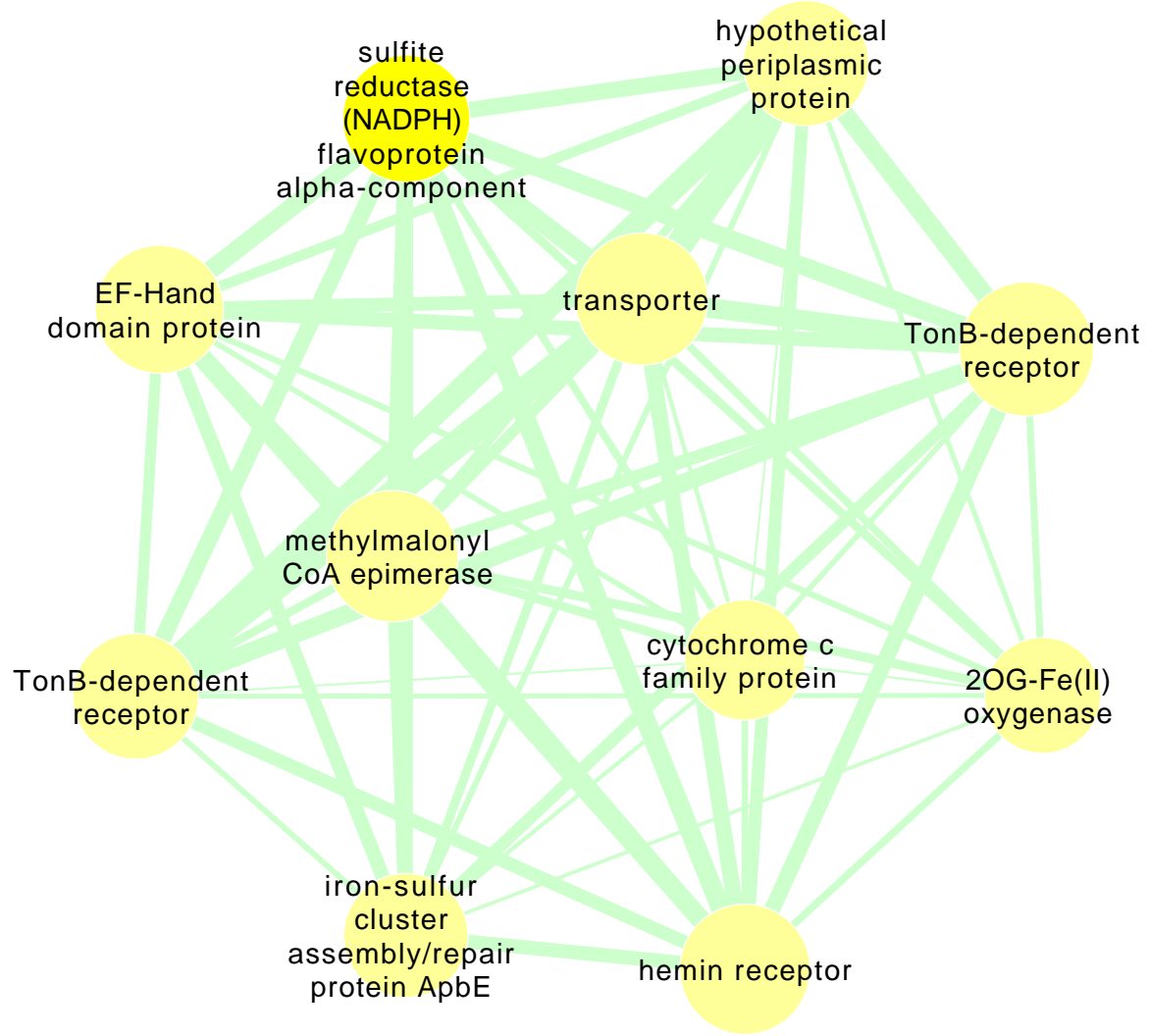

Supplement: Additional file 13: Figure S4 — Co-expression network topologies of all 76 modules. [file 1471-2164-14-450-S13.zip › FigureS4/thistle1.pdf]

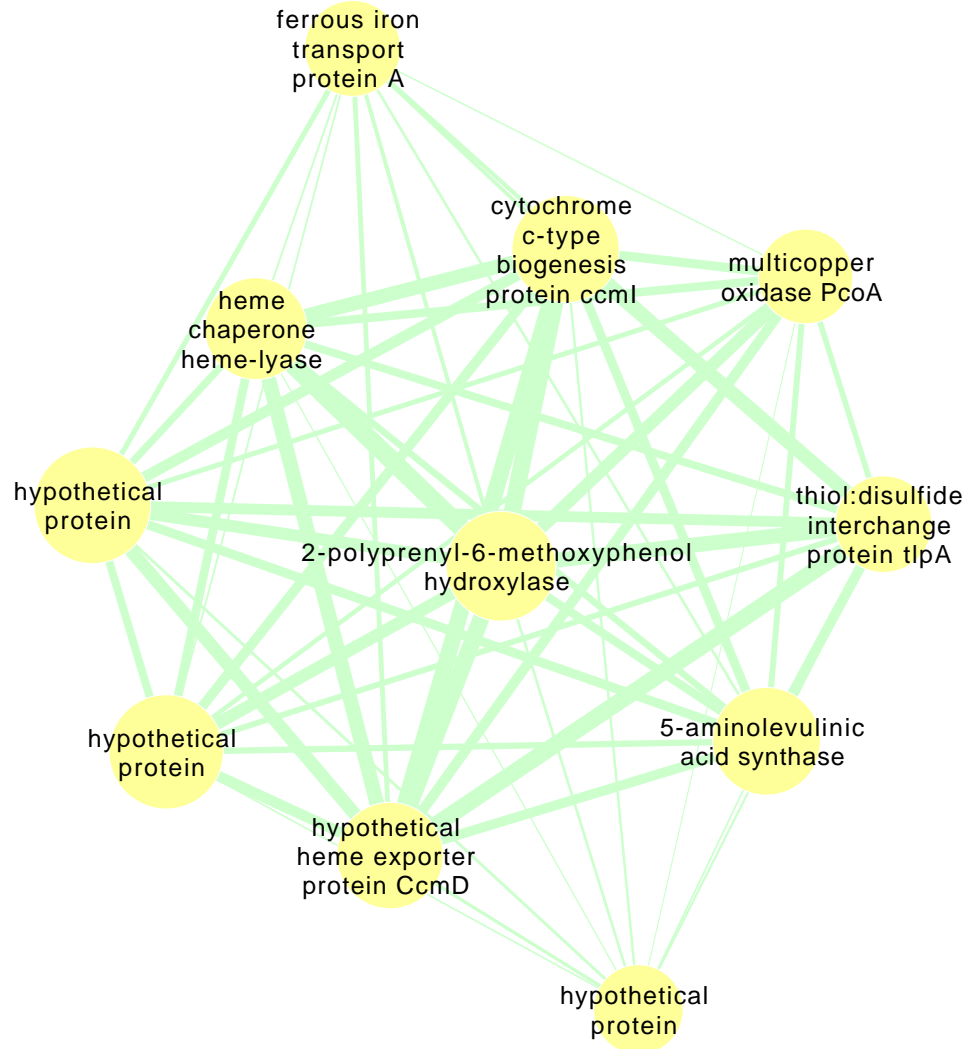

Supplement: Additional file 13: Figure S4 — Co-expression network topologies of all 76 modules. [file 1471-2164-14-450-S13.zip › FigureS4/thistle2.pdf]

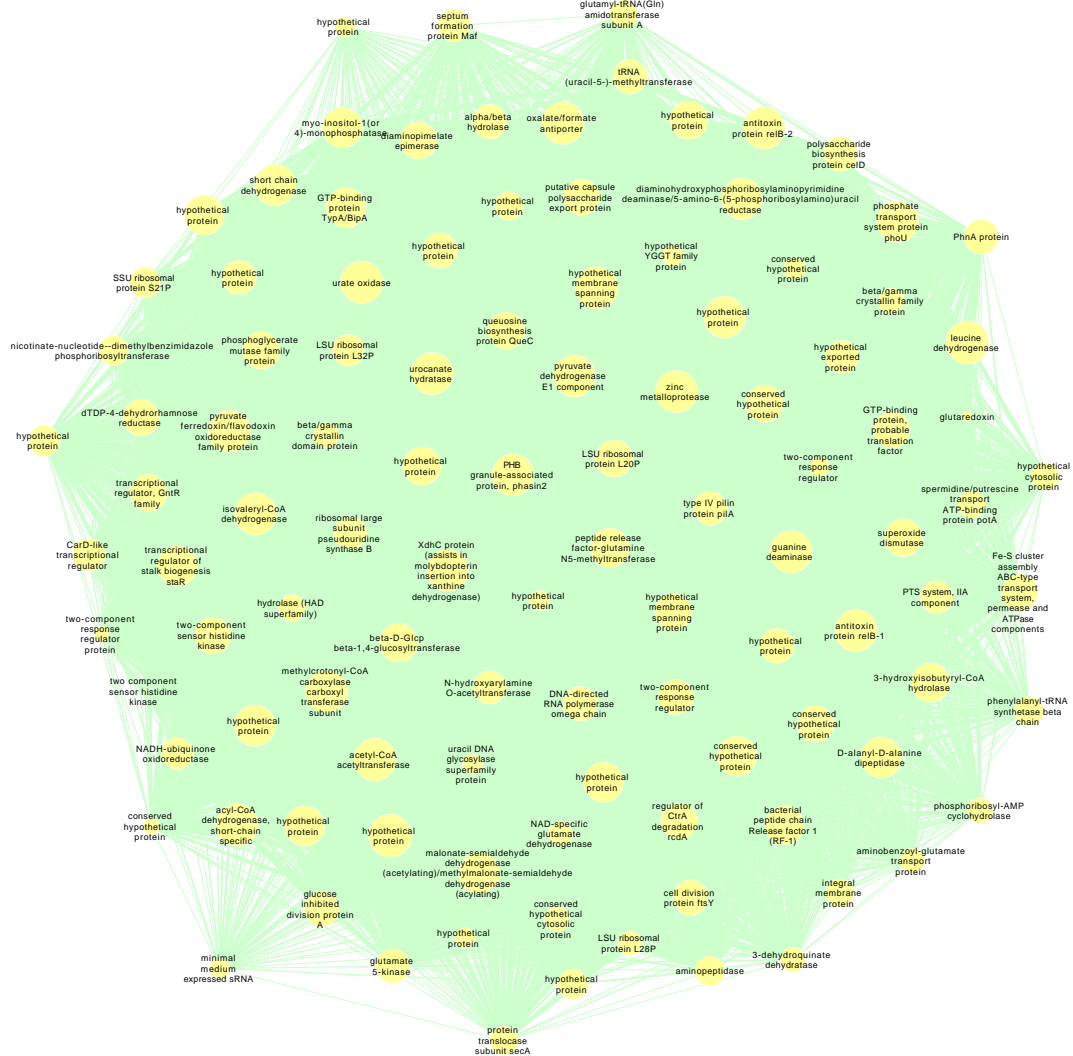

Supplement: Additional file 13: Figure S4 — Co-expression network topologies of all 76 modules. [file 1471-2164-14-450-S13.zip › FigureS4/turquoise.pdf]

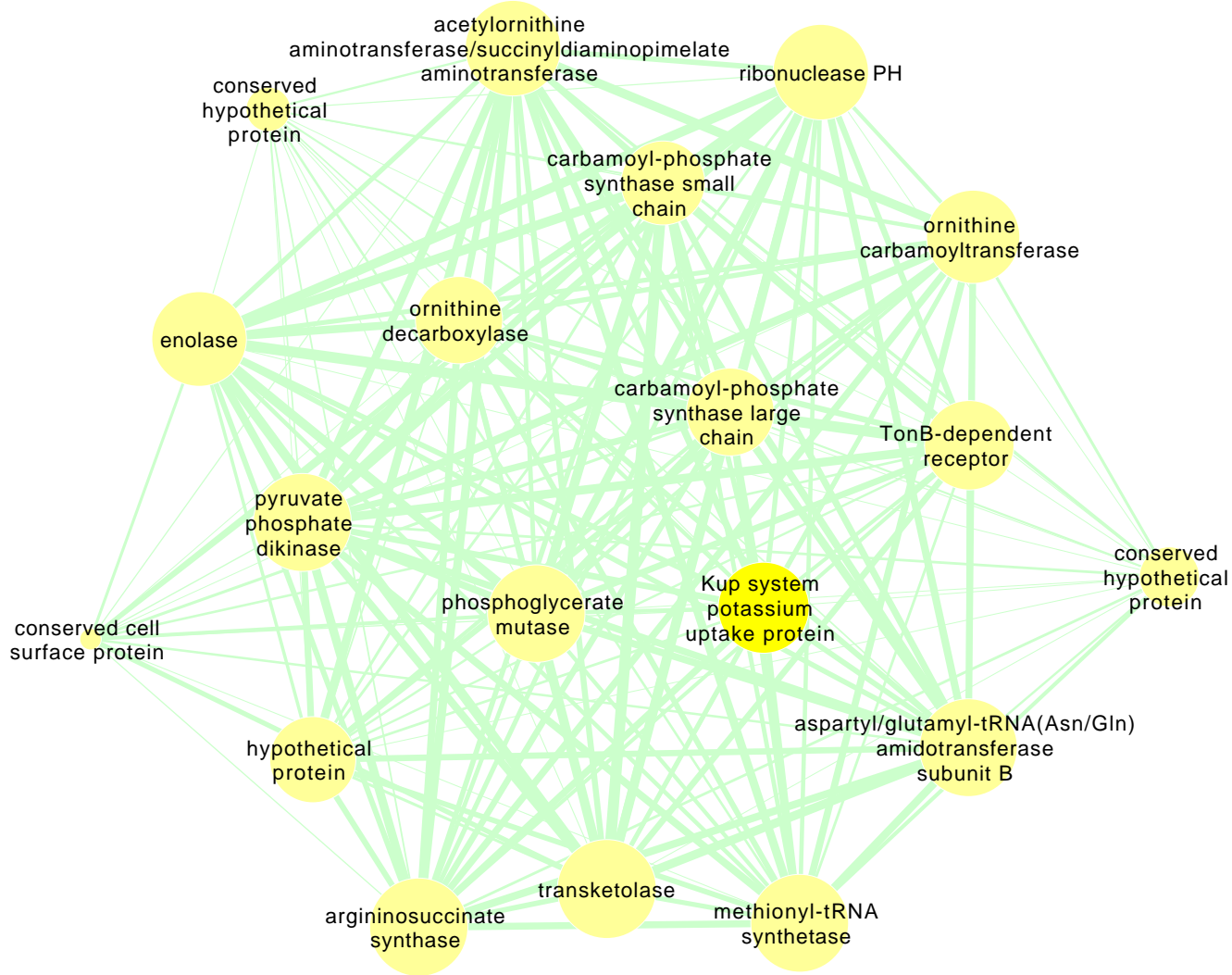

Supplement: Additional file 13: Figure S4 — Co-expression network topologies of all 76 modules. [file 1471-2164-14-450-S13.zip › FigureS4/violet.pdf]

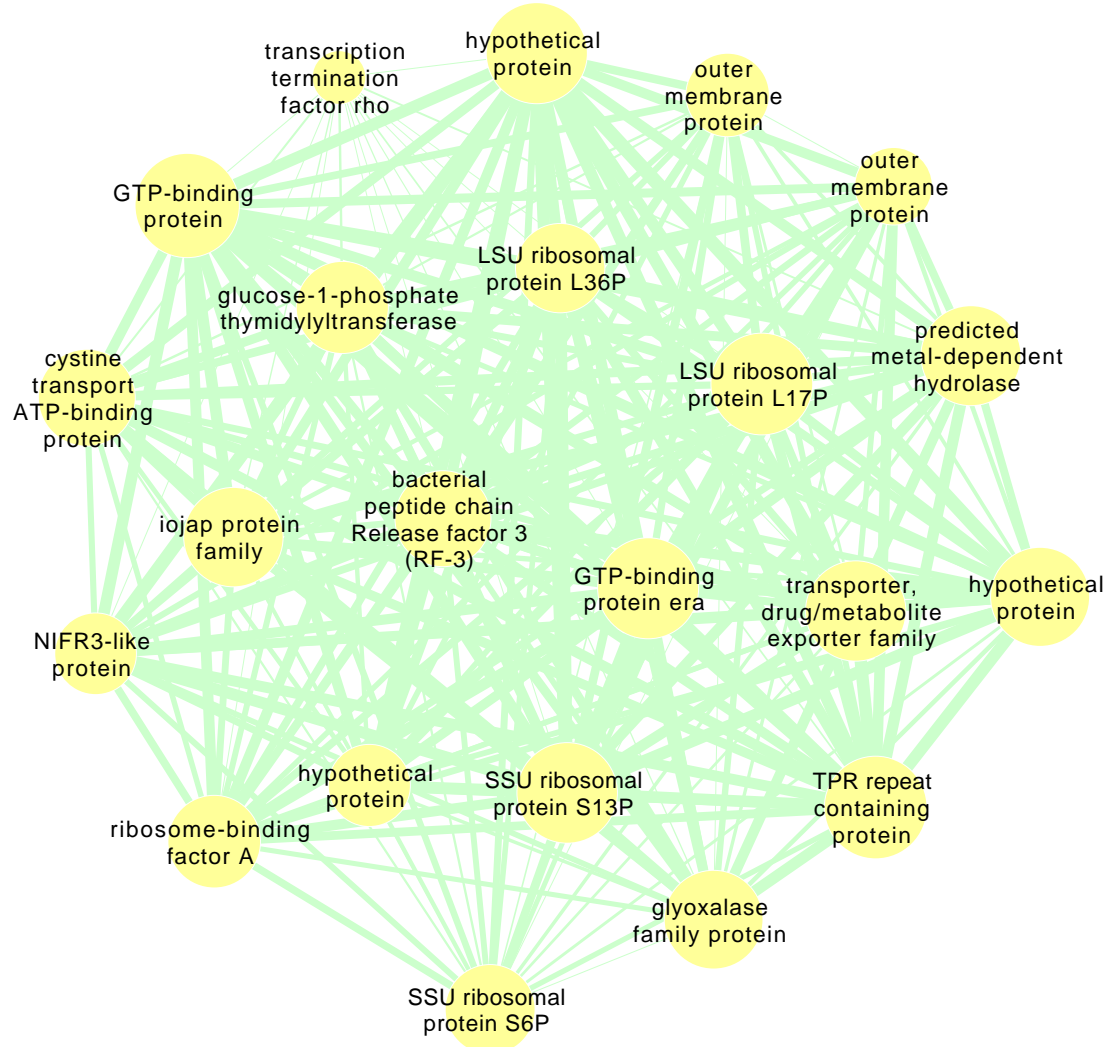

Supplement: Additional file 13: Figure S4 — Co-expression network topologies of all 76 modules. [file 1471-2164-14-450-S13.zip › FigureS4/white.pdf]

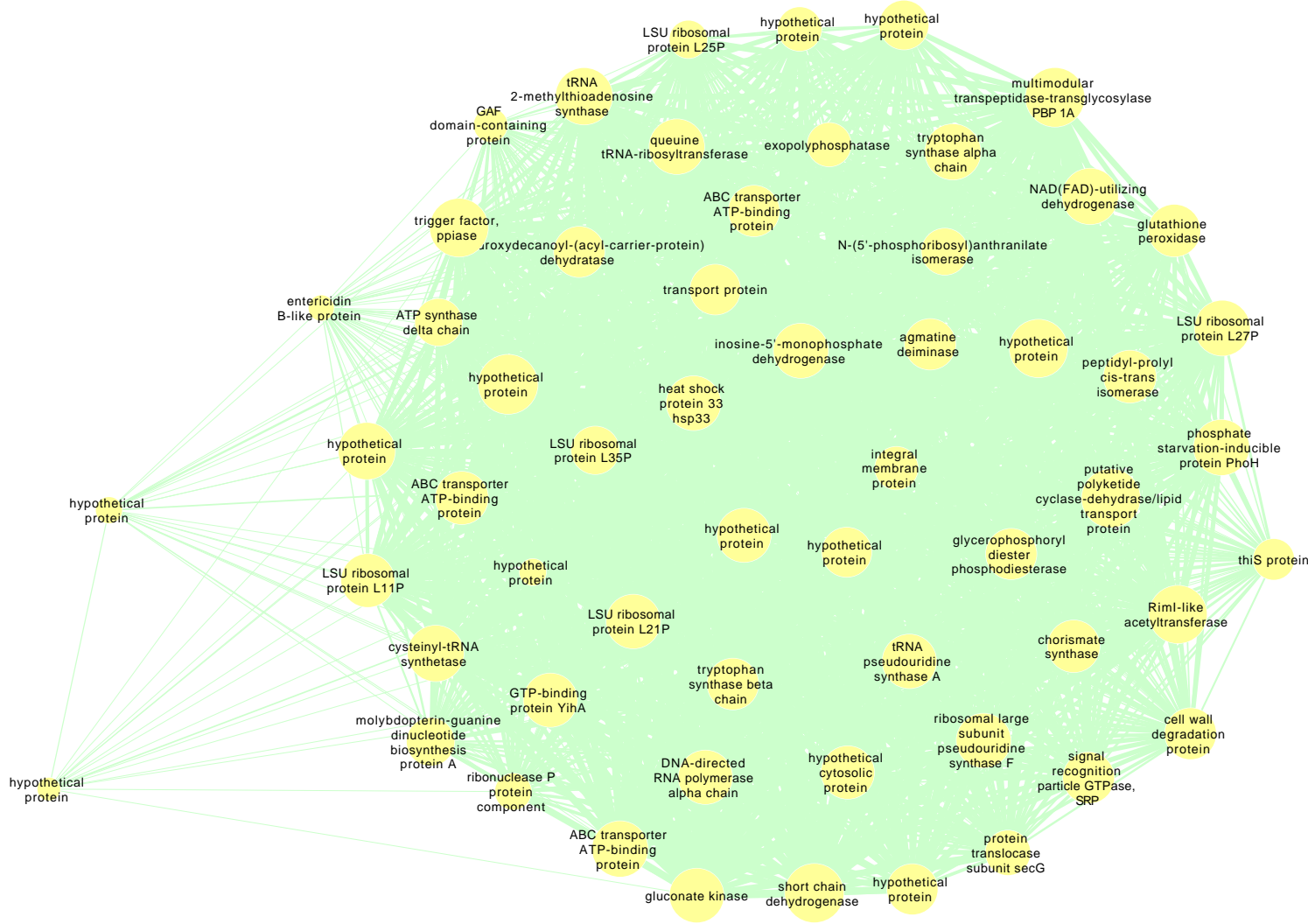

Supplement: Additional file 13: Figure S4 — Co-expression network topologies of all 76 modules. [file 1471-2164-14-450-S13.zip › FigureS4/yellow.pdf]

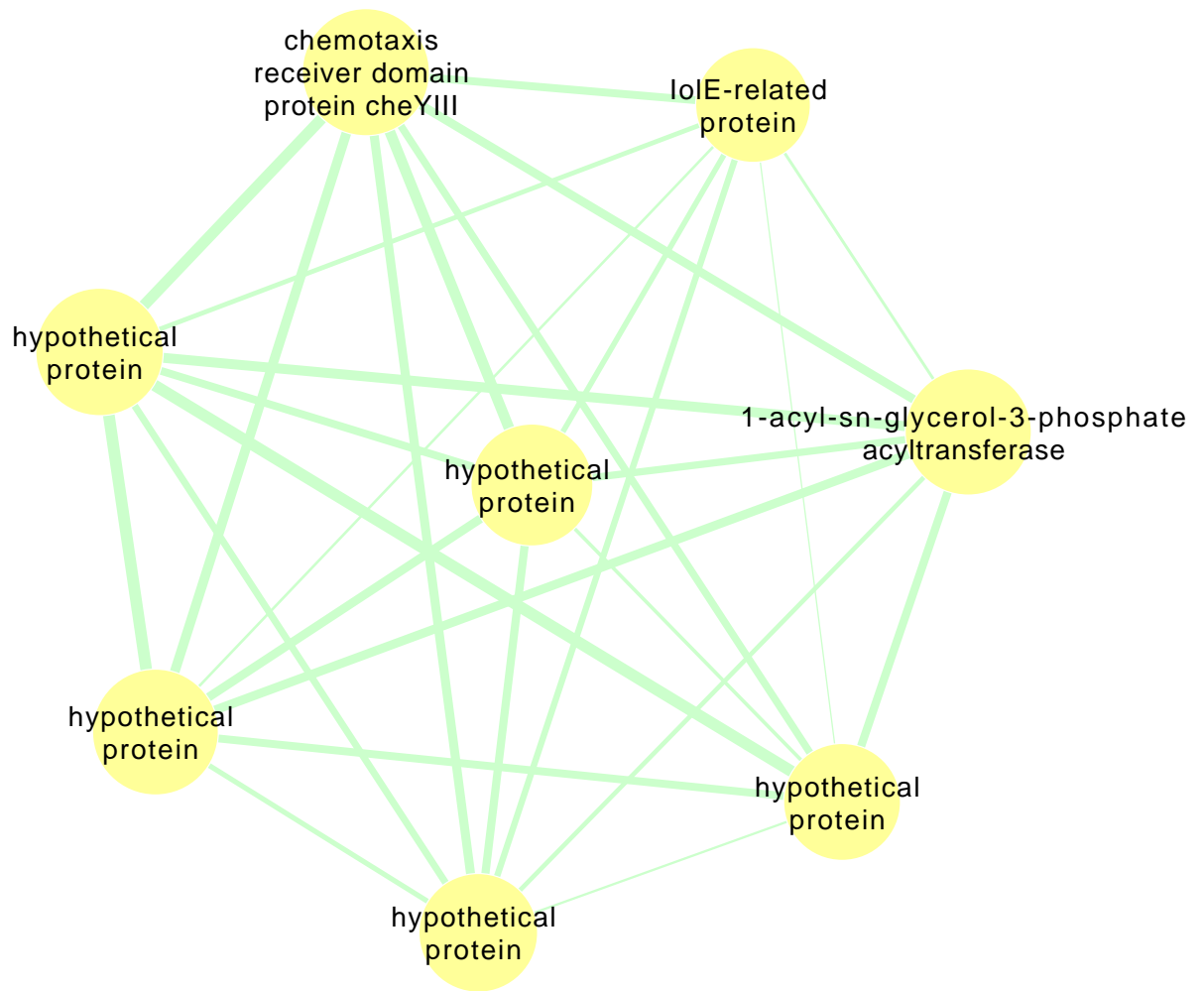

Supplement: Additional file 13: Figure S4 — Co-expression network topologies of all 76 modules. [file 1471-2164-14-450-S13.zip › FigureS4/yellow4.pdf]

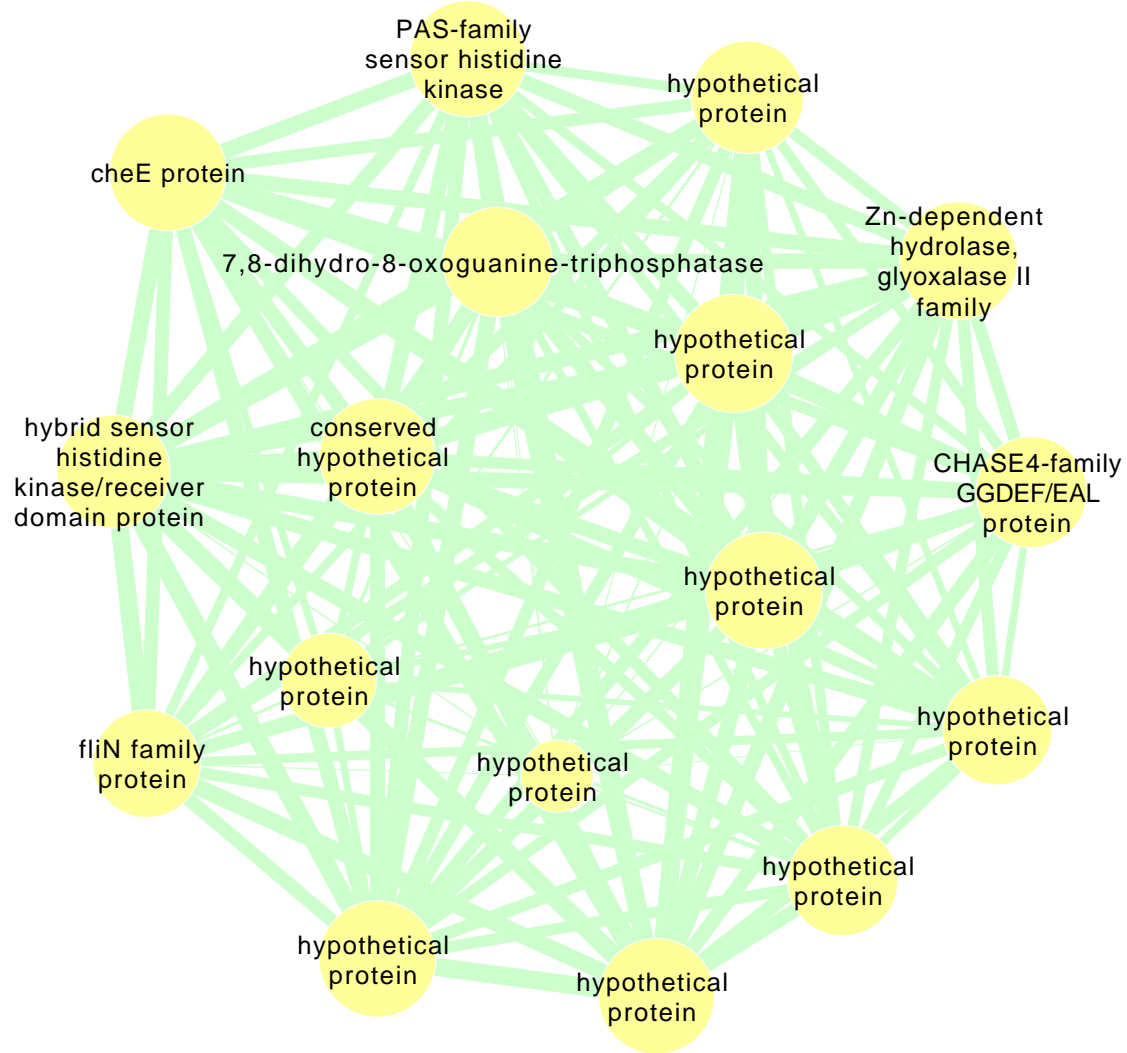

Supplement: Additional file 13: Figure S4 — Co-expression network topologies of all 76 modules. [file 1471-2164-14-450-S13.zip › FigureS4/yellowgreen.pdf]

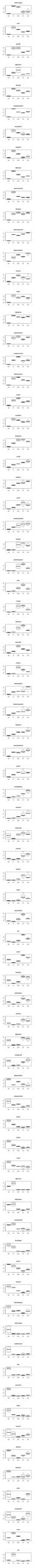

Supplement: Additional file 15: Figure S5 — Module expression profile represented by its 1st eigenvector. [file 1471-2164-14-450-S15.png]

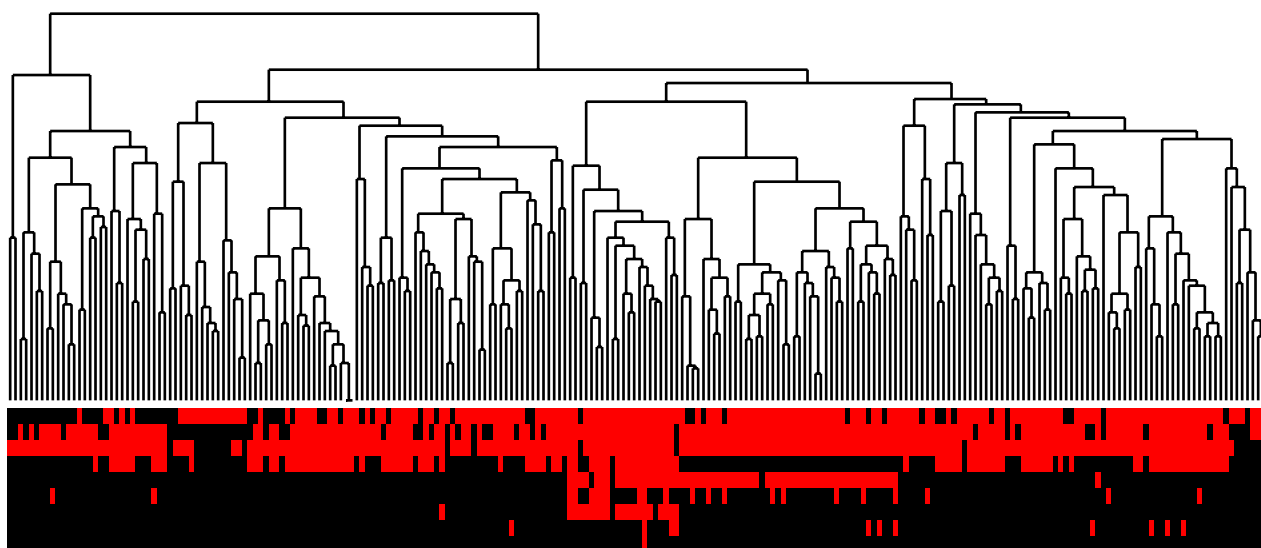

Supplement: Additional file 19: Figure S6 — Phylogenetic profiles and positions in MPD and MNTD coordinates for all modules. [file 1471-2164-14-450-S19.zip › FigureS6/antiquewhite4.pdf]

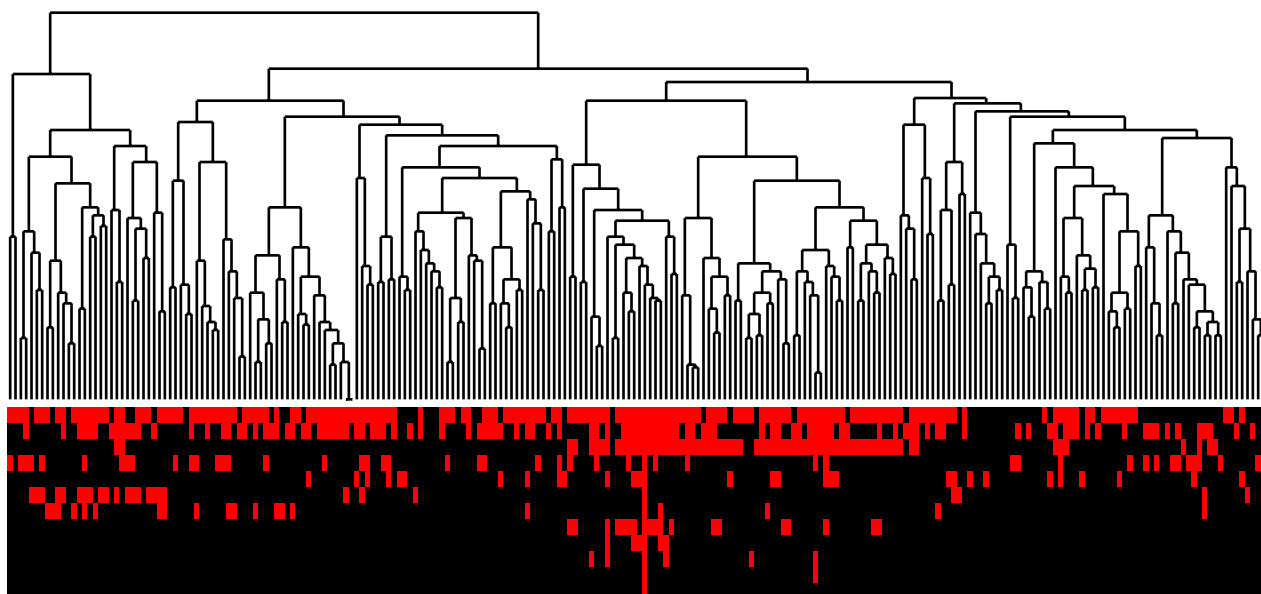

Supplement: Additional file 19: Figure S6 — Phylogenetic profiles and positions in MPD and MNTD coordinates for all modules. [file 1471-2164-14-450-S19.zip › FigureS6/bisque4.pdf]

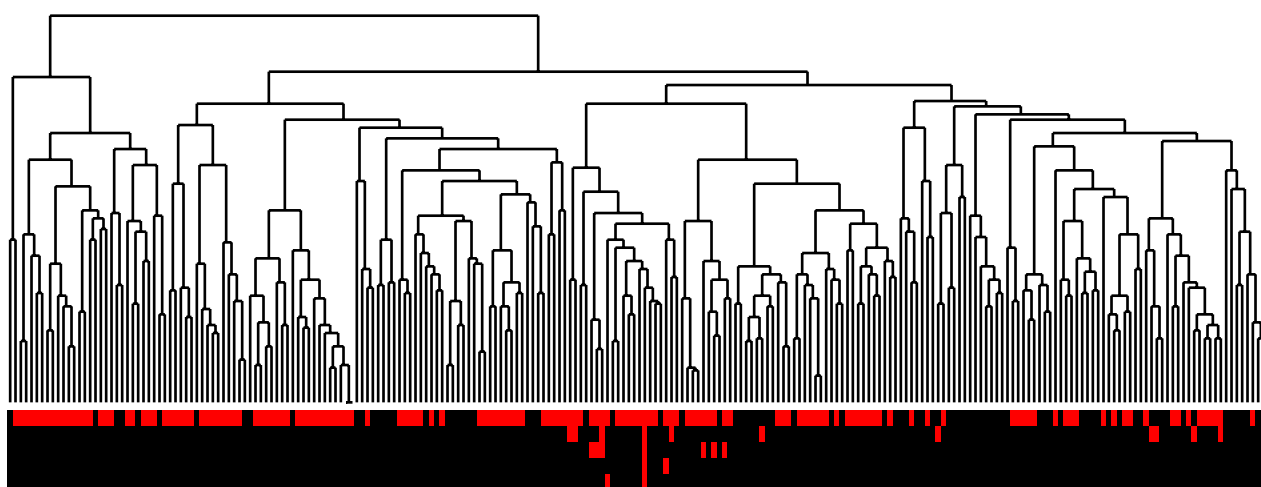

CCNA\_02641  
CCNA\_03180  
CCNA\_03227  
CCNA\_00260  
CCNA\_02512

Supplement: Additional file 19: Figure S6 — Phylogenetic profiles and positions in MPD and MNTD coordinates for all modules. [file 1471-2164-14-450-S19.zip › FigureS6/blue2.pdf]

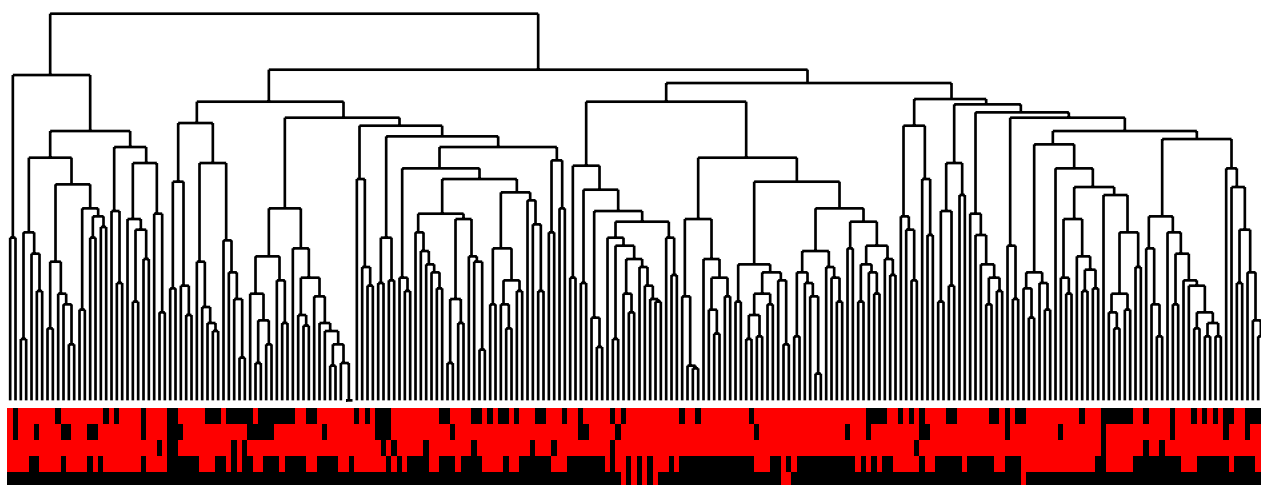

Supplement: Additional file 19: Figure S6 — Phylogenetic profiles and positions in MPD and MNTD coordinates for all modules. [file 1471-2164-14-450-S19.zip › FigureS6/brown2.pdf]

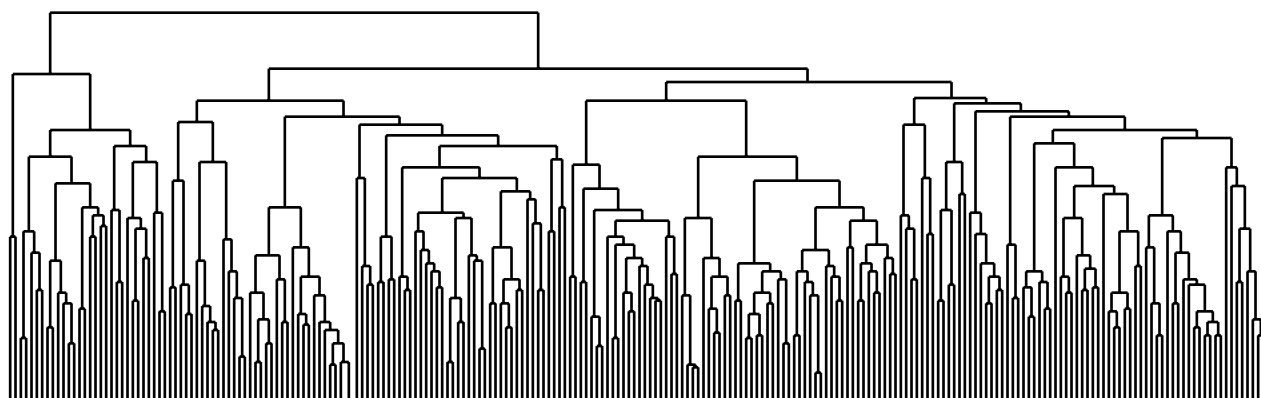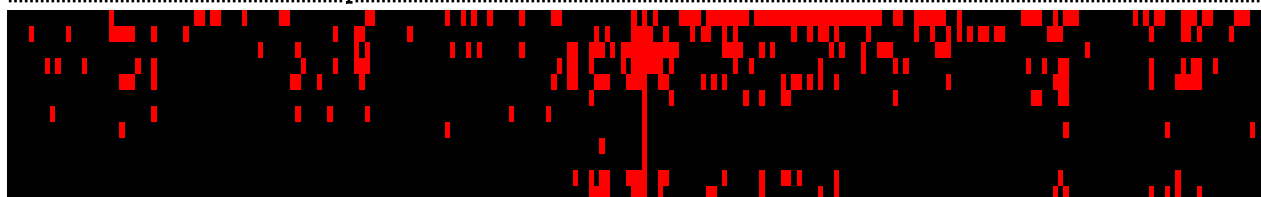

CCNA\_01463  
CCNA\_03172  
CCNA\_00789  
CCNA\_03092  
CCNA\_00726  
CCNA\_03153  
CCNA\_03122  
CCNA\_01020  
CCNA\_00433  
CCNA\_01767  
CCNA\_01368  
CCNA\_02178

Supplement: Additional file 19: Figure S6 — Phylogenetic profiles and positions in MPD and MNTD coordinates for all modules. [file 1471-2164-14-450-S19.zip › FigureS6/brown4.pdf]

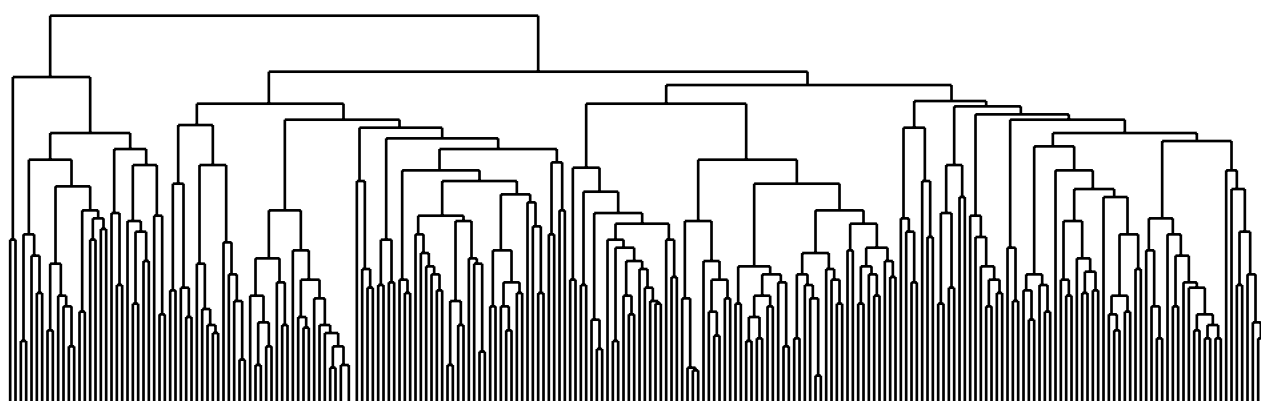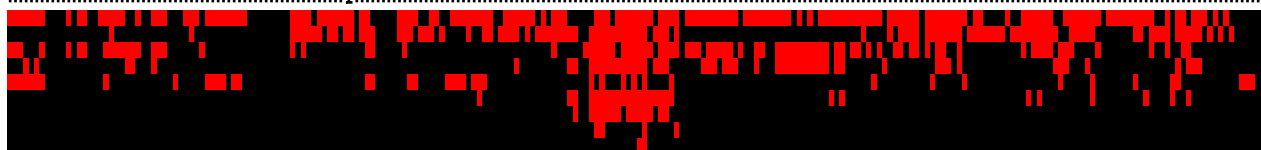

CCNA\_03007  
CCNA\_03322  
CCNA\_03006  
CCNA\_00078  
CCNA\_03734  
CCNA\_03323  
CCNA\_02010  
CCNA\_00354  
CCNA\_02452

Supplement: Additional file 19: Figure S6 — Phylogenetic profiles and positions in MPD and MNTD coordinates for all modules. [file 1471-2164-14-450-S19.zip › FigureS6/coral1.pdf]

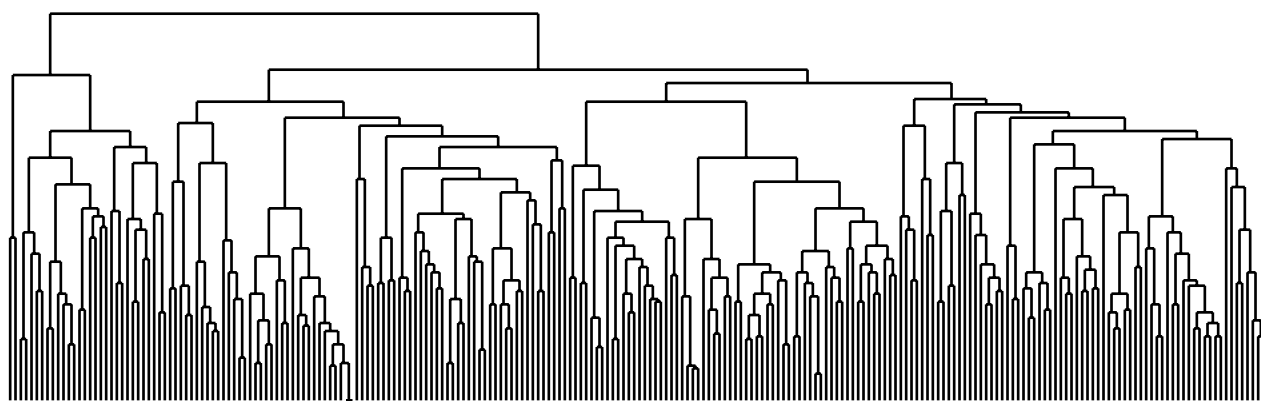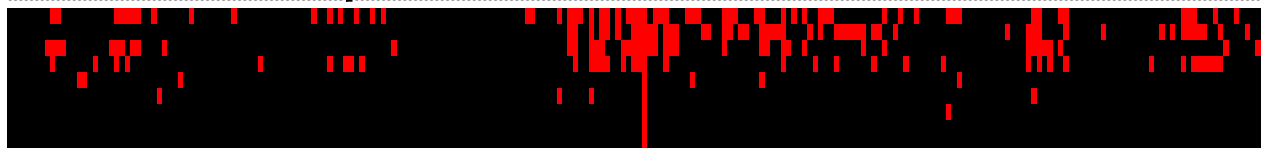

CCNA\_03203  
CCNA\_01285  
CCNA\_01452  
CCNA\_01287  
CCNA\_02761  
CCNA\_02662  
CCNA\_00427  
CCNA\_02680  
CCNA\_03286

Supplement: Additional file 19: Figure S6 — Phylogenetic profiles and positions in MPD and MNTD coordinates for all modules. [file 1471-2164-14-450-S19.zip › FigureS6/coral2.pdf]

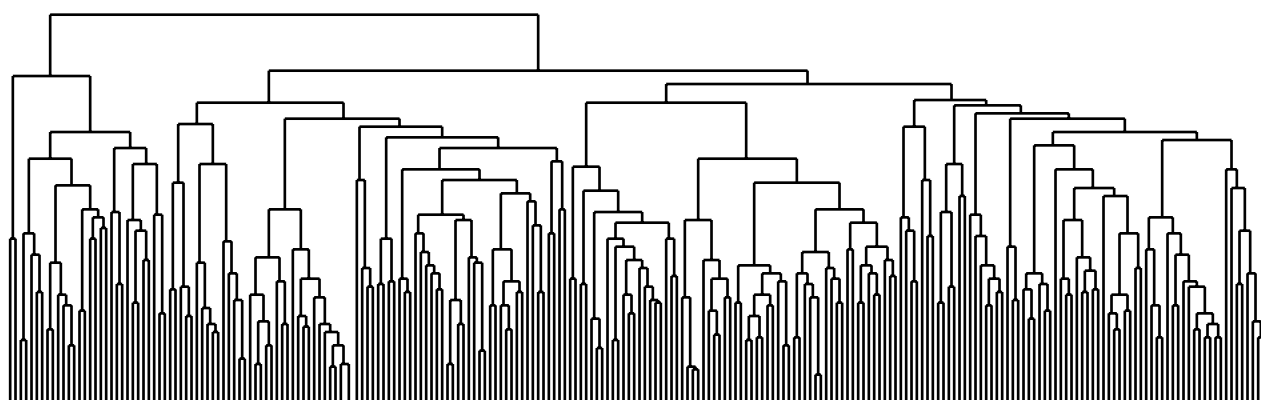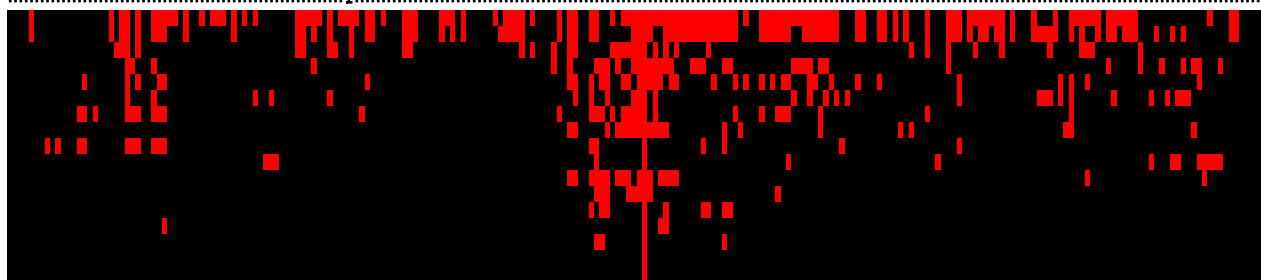

CCNA\_00332  
CCNA\_00331  
CCNA\_02429  
CCNA\_02725  
CCNA\_00021  
CCNA\_01384  
CCNA\_01031  
CCNA\_00247  
CCNA\_01707  
CCNA\_02400  
CCNA\_01686  
CCNA\_03572  
CCNA\_00338  
CCNA\_02873  
CCNA\_00396  
CCNA\_01120  
CCNA\_02820

Supplement: Additional file 19: Figure S6 — Phylogenetic profiles and positions in MPD and MNTD coordinates for all modules. [file 1471-2164-14-450-S19.zip › FigureS6/darkmagenta.pdf]

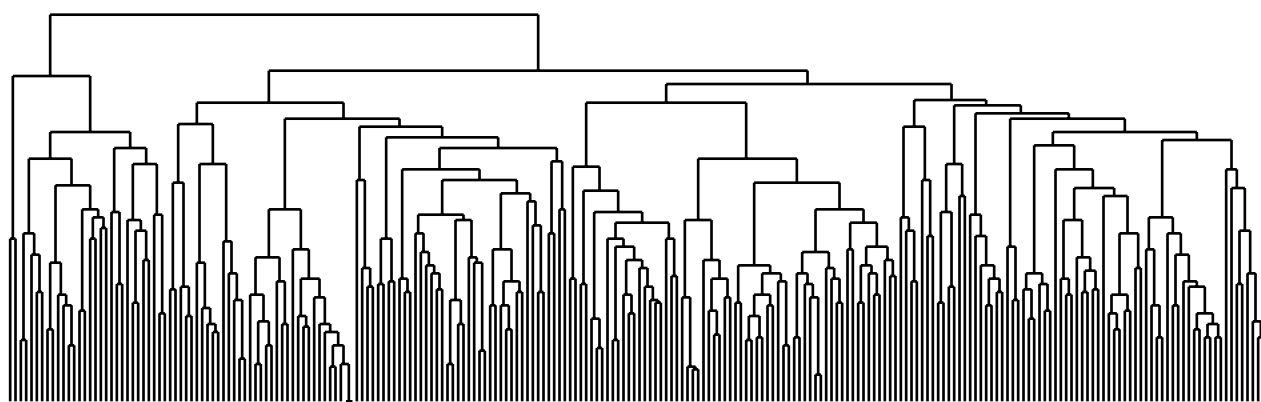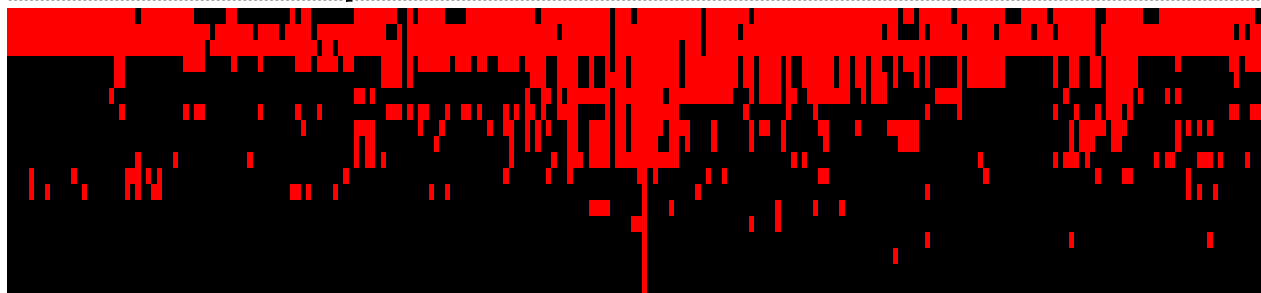

CCNA\_03346  
CCNA\_03345  
CCNA\_03344  
CCNA\_02143  
CCNA\_02665  
CCNA\_02848  
CCNA\_02142  
CCNA\_03036  
CCNA\_03035  
CCNA\_02635  
CCNA\_02951  
CCNA\_03137  
CCNA\_01016  
CCNA\_00943  
CCNA\_00944  
CCNA\_00942  
CCNA\_02852  
CCNA\_02901

Supplement: Additional file 19: Figure S6 — Phylogenetic profiles and positions in MPD and MNTD coordinates for all modules. [file 1471-2164-14-450-S19.zip › FigureS6/darkolivegreen.pdf]

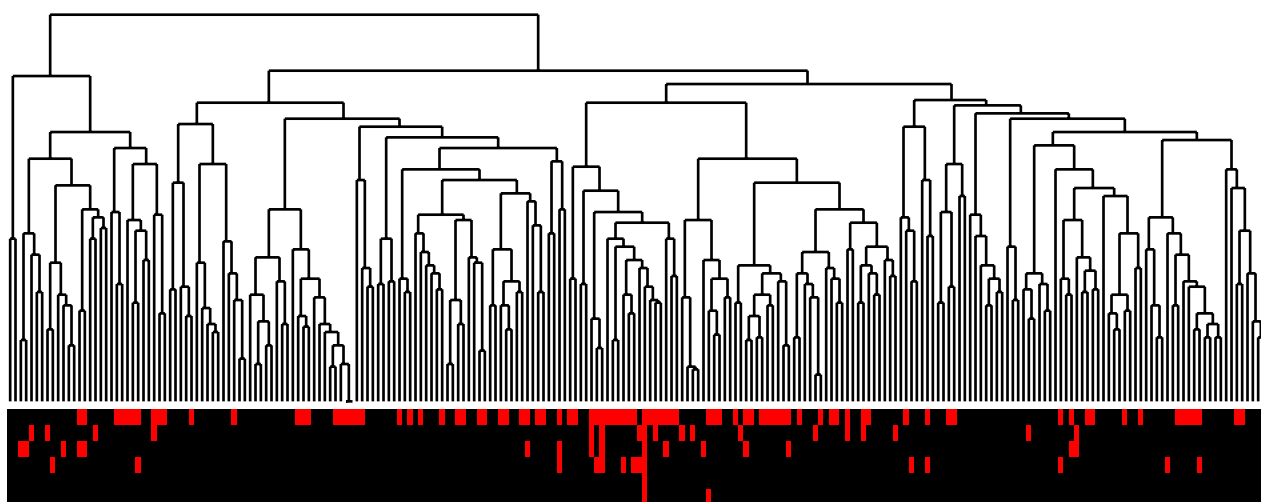

CCNA\_02709  
CCNA\_00426  
CCNA\_00339  
CCNA\_03294  
CCNA\_01657  
CCNA\_01022

Supplement: Additional file 19: Figure S6 — Phylogenetic profiles and positions in MPD and MNTD coordinates for all modules. [file 1471-2164-14-450-S19.zip › FigureS6/darkolivegreen4.pdf]

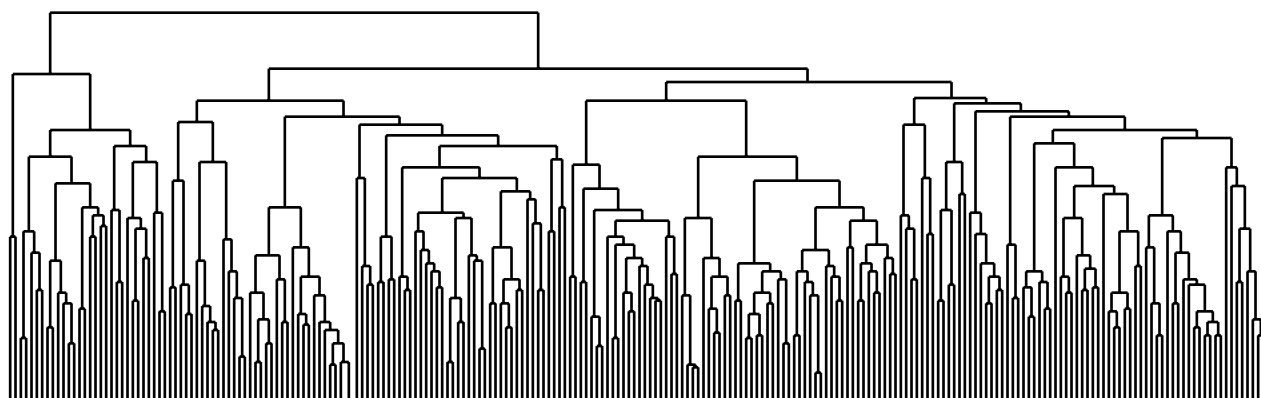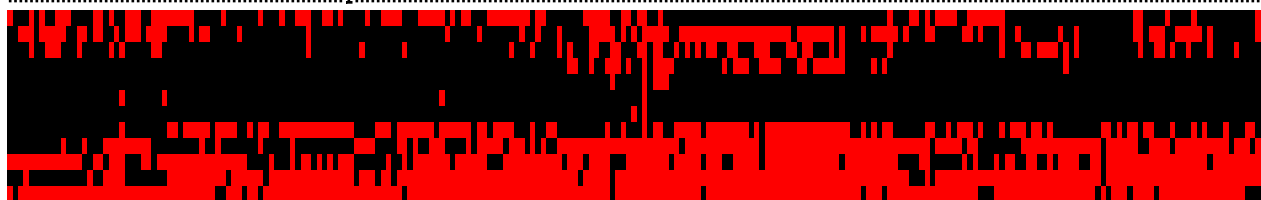

CCNA\_01624  
CCNA\_01550  
CCNA\_01175  
CCNA\_03314  
CCNA\_01176  
CCNA\_00223  
CCNA\_03079  
CCNA\_01915  
CCNA\_03653  
CCNA\_03828  
CCNA\_01730  
CCNA\_01590

Supplement: Additional file 19: Figure S6 — Phylogenetic profiles and positions in MPD and MNTD coordinates for all modules. [file 1471-2164-14-450-S19.zip › FigureS6/darkorange2.pdf]

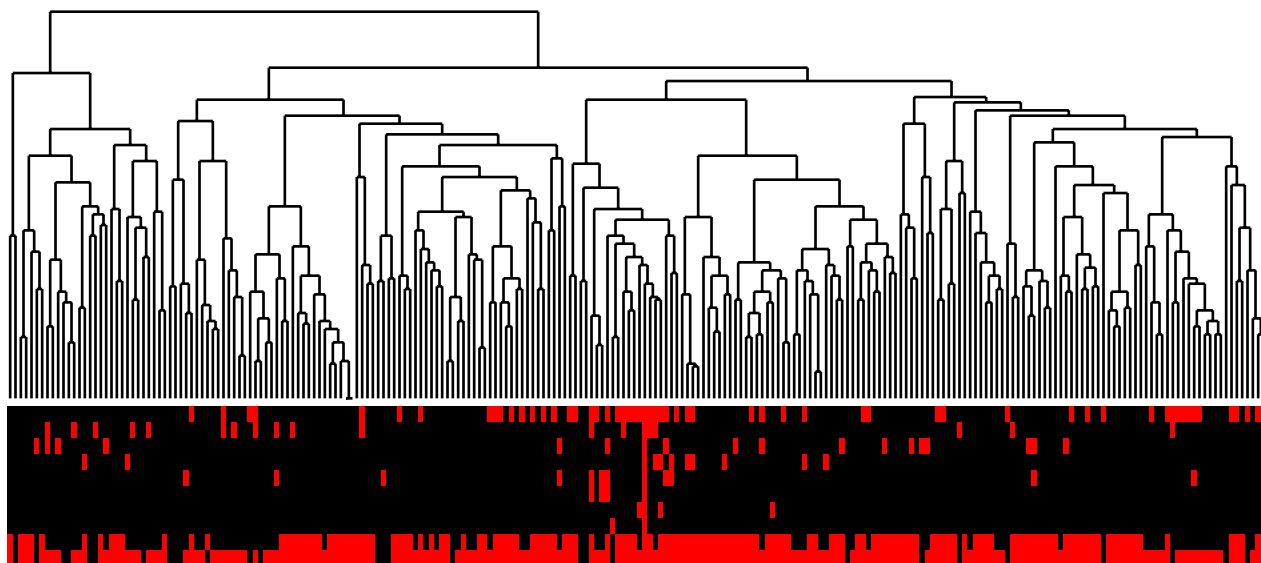

Supplement: Additional file 19: Figure S6 — Phylogenetic profiles and positions in MPD and MNTD coordinates for all modules. [file 1471-2164-14-450-S19.zip › FigureS6/darkseagreen4.pdf]

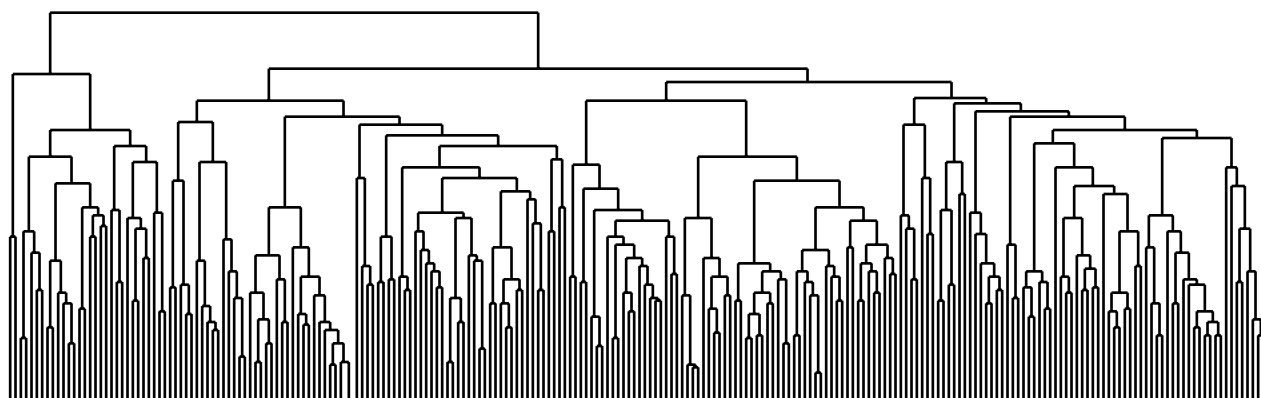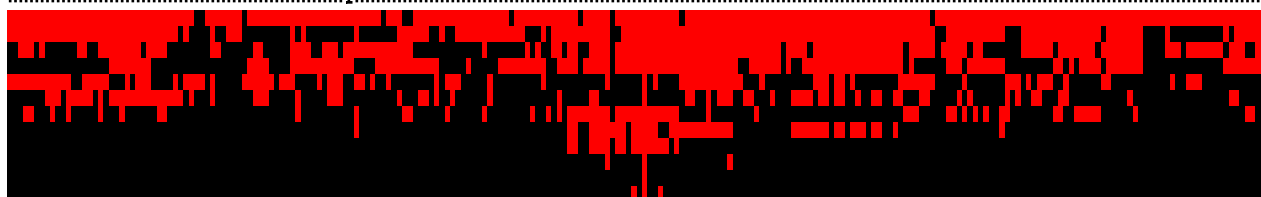

CCNA\_01920  
CCNA\_00317  
CCNA\_03378  
CCNA\_03843  
CCNA\_03469  
CCNA\_00264  
CCNA\_03798  
CCNA\_03385  
CCNA\_03105  
CCNA\_01653  
CCNA\_01007  
CCNA\_03257

Supplement: Additional file 19: Figure S6 — Phylogenetic profiles and positions in MPD and MNTD coordinates for all modules. [file 1471-2164-14-450-S19.zip › FigureS6/darkslateblue.pdf]

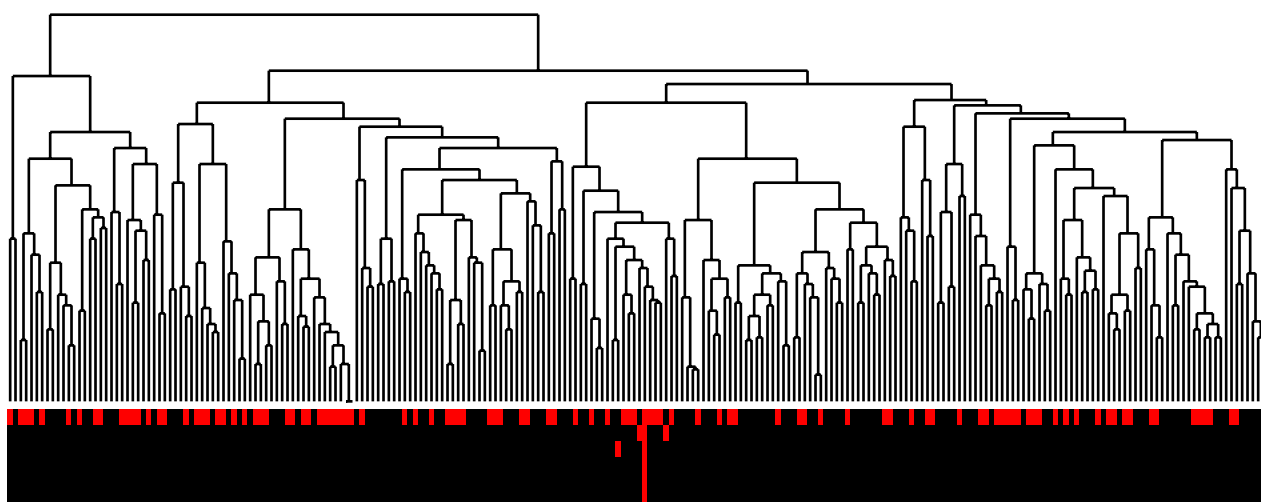

CCNA\_01913  
CCNA\_00999  
CCNA\_03630  
CCNA\_03182  
CCNA\_00718  
CCNA\_03629

Supplement: Additional file 19: Figure S6 — Phylogenetic profiles and positions in MPD and MNTD coordinates for all modules. [file 1471-2164-14-450-S19.zip › FigureS6/firebrick4.pdf]

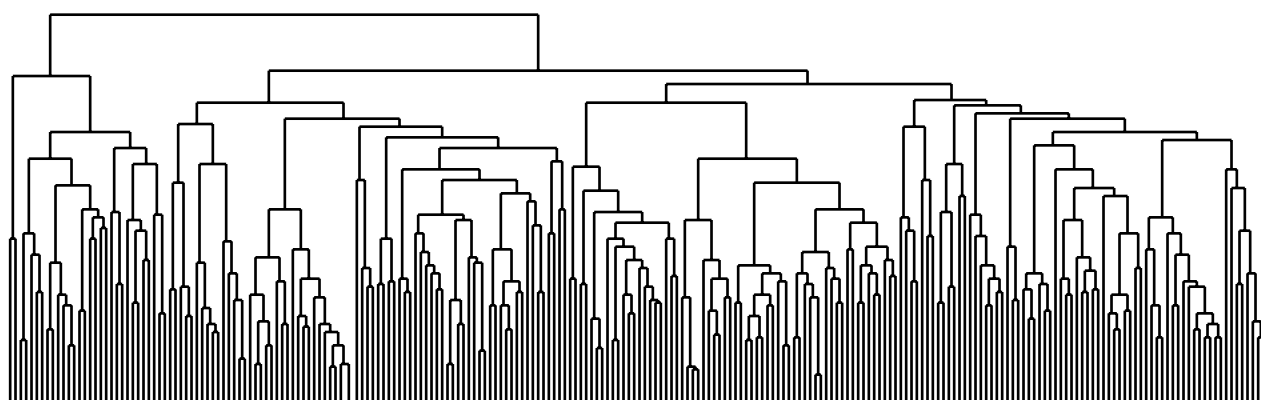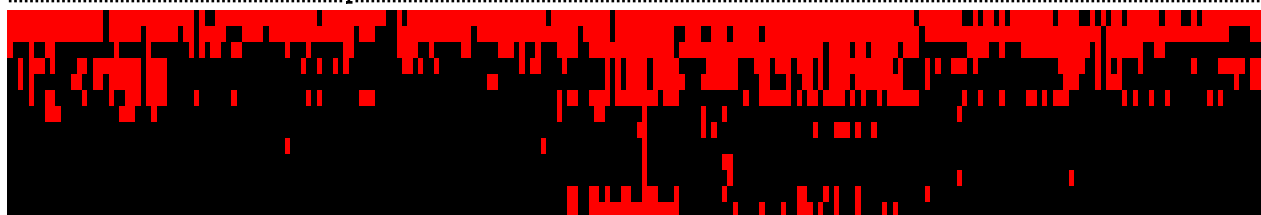

CCNA\_00008  
CCNA\_02525  
CCNA\_02046  
CCNA\_01262  
CCNA\_01261  
CCNA\_01674  
CCNA\_00933  
CCNA\_02472  
CCNA\_02367  
CCNA\_01034  
CCNA\_02471  
CCNA\_00649  
CCNA\_01815

Supplement: Additional file 19: Figure S6 — Phylogenetic profiles and positions in MPD and MNTD coordinates for all modules. [file 1471-2164-14-450-S19.zip › FigureS6/floralwhite.pdf]

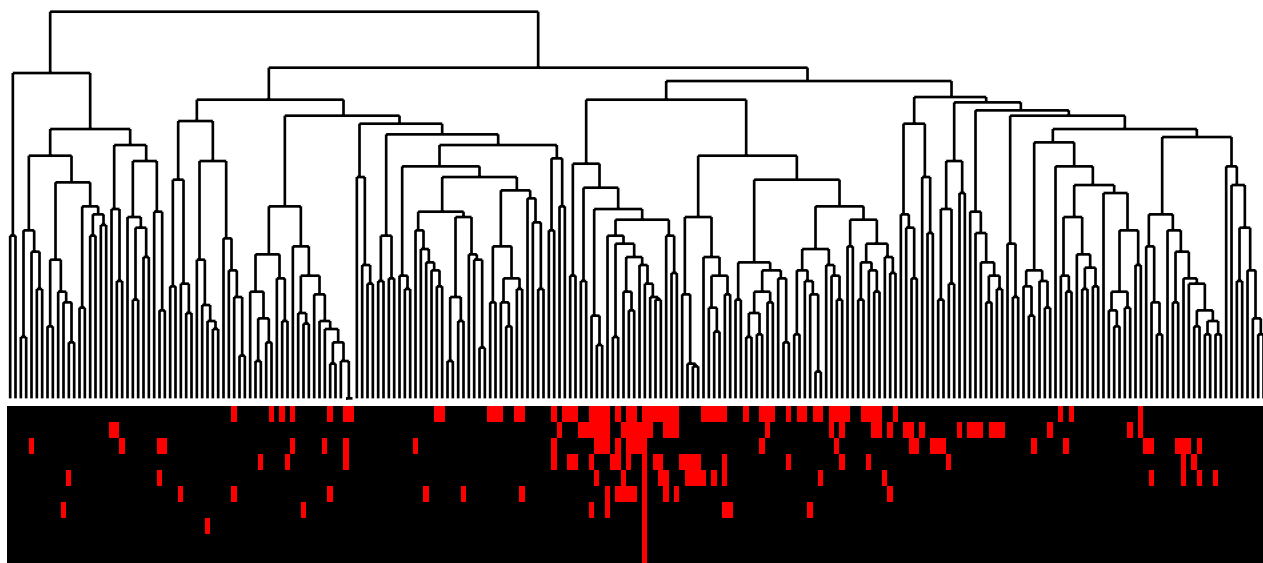

Supplement: Additional file 19: Figure S6 — Phylogenetic profiles and positions in MPD and MNTD coordinates for all modules. [file 1471-2164-14-450-S19.zip › FigureS6/honeydew1.pdf]

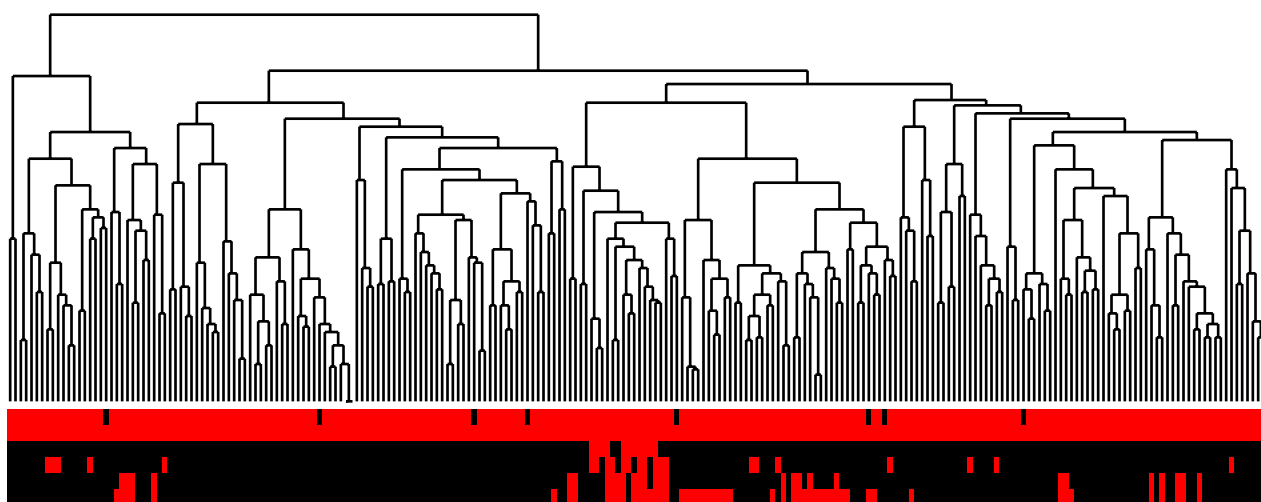

Supplement: Additional file 19: Figure S6 — Phylogenetic profiles and positions in MPD and MNTD coordinates for all modules. [file 1471-2164-14-450-S19.zip › FigureS6/indianred4.pdf]

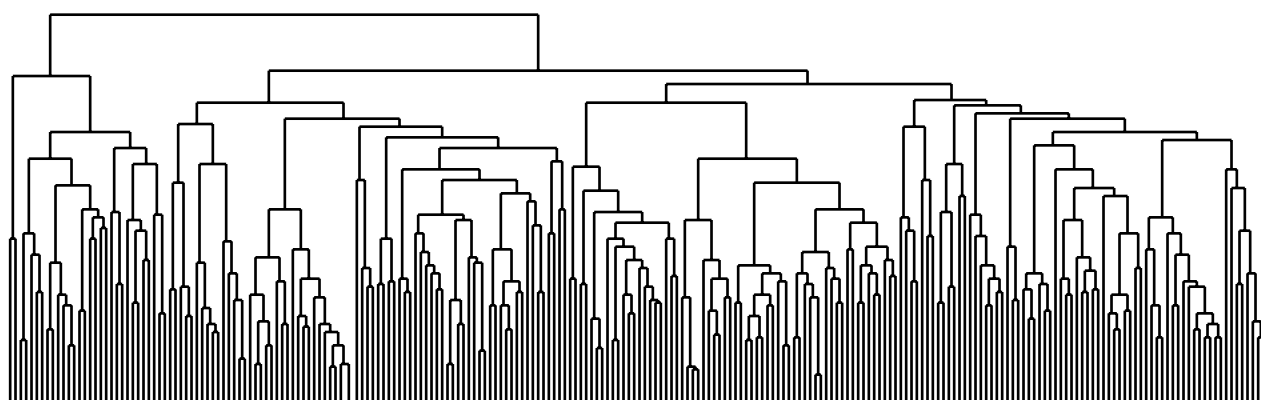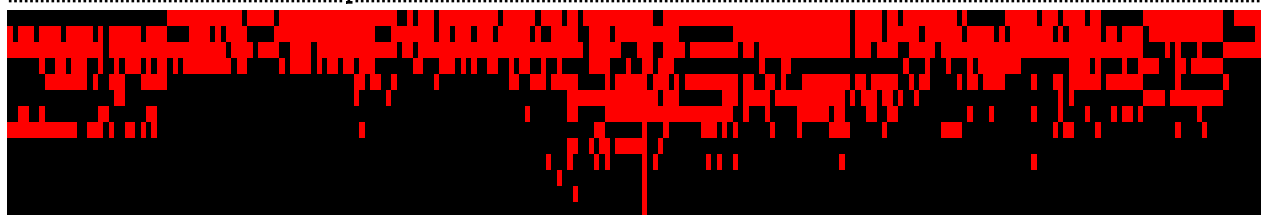

CCNA\_01420  
CCNA\_01976  
CCNA\_01612  
CCNA\_03750  
CCNA\_02076  
CCNA\_00691  
CCNA\_03582  
CCNA\_01977  
CCNA\_02328  
CCNA\_02028  
CCNA\_01371  
CCNA\_03583  
CCNA\_03082

Supplement: Additional file 19: Figure S6 — Phylogenetic profiles and positions in MPD and MNTD coordinates for all modules. [file 1471-2164-14-450-S19.zip › FigureS6/ivory.pdf]

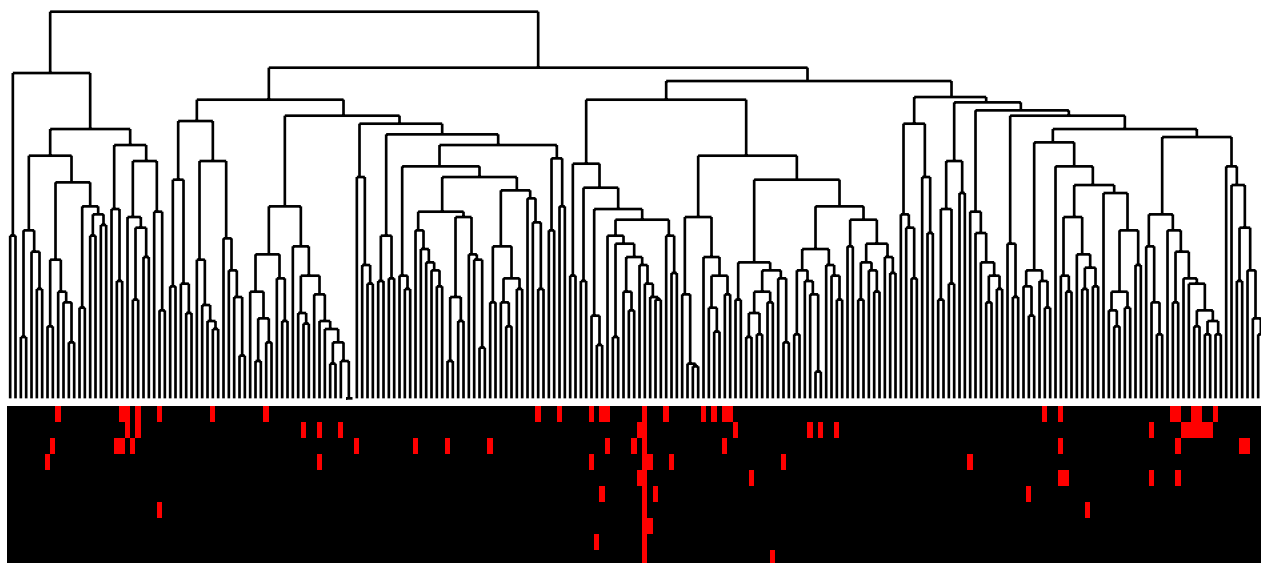

Supplement: Additional file 19: Figure S6 — Phylogenetic profiles and positions in MPD and MNTD coordinates for all modules. [file 1471-2164-14-450-S19.zip › FigureS6/lavenderblush3.pdf]

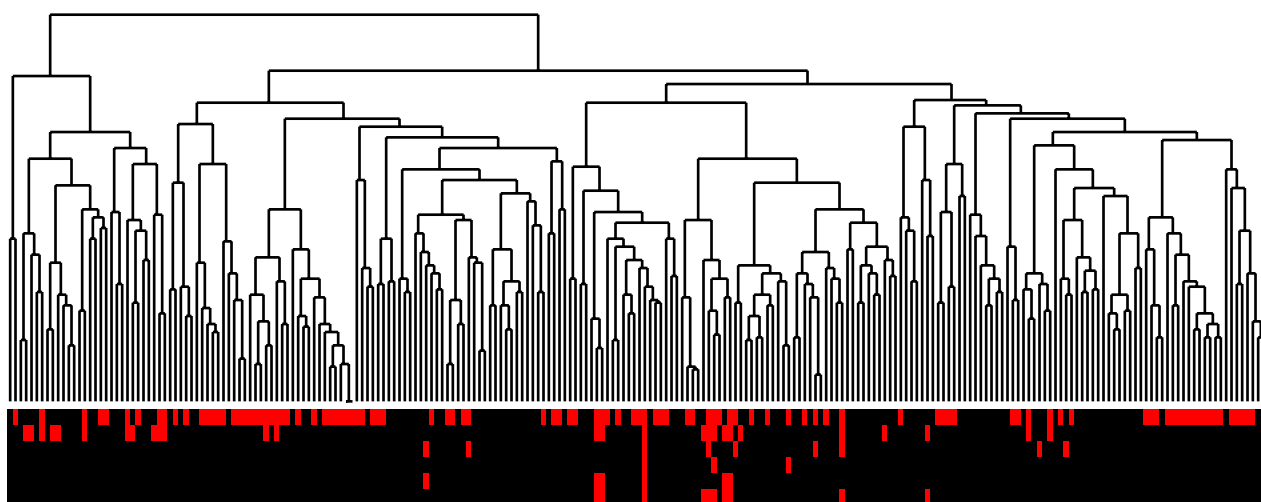

Supplement: Additional file 19: Figure S6 — Phylogenetic profiles and positions in MPD and MNTD coordinates for all modules. [file 1471-2164-14-450-S19.zip › FigureS6/lightcoral.pdf]

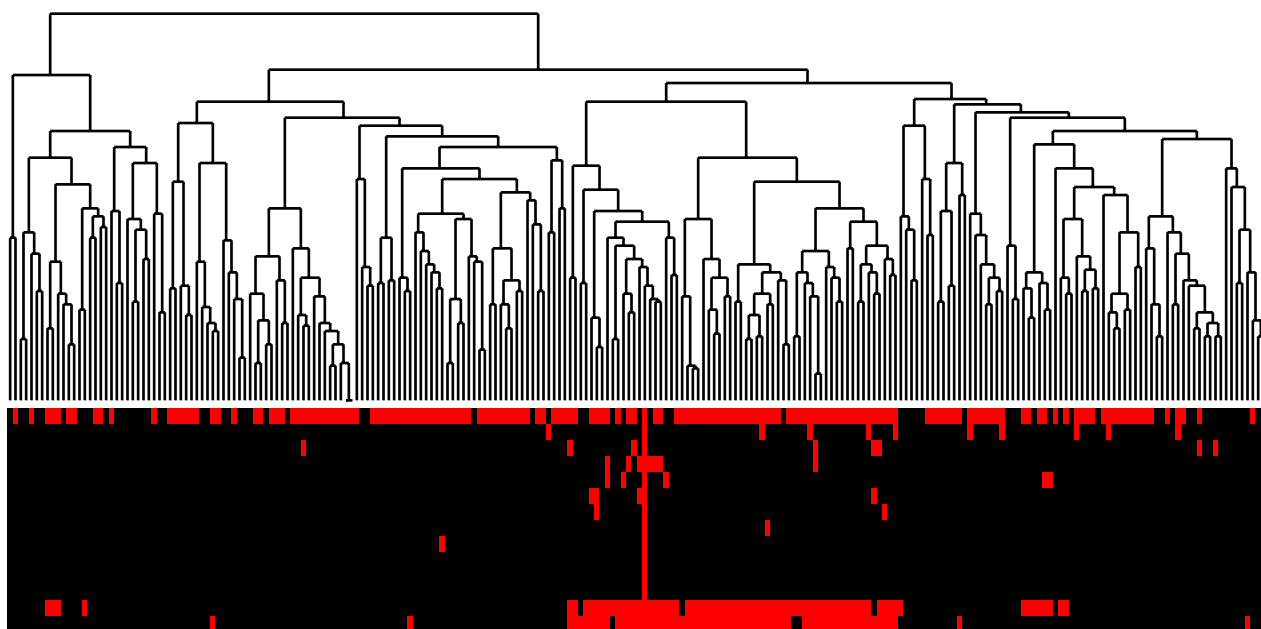

CCNA\_00055  
CCNA\_00521  
CCNA\_03795  
CCNA\_00503  
CCNA\_00774  
CCNA\_02067  
CCNA\_02131  
CCNA\_01973  
CCNA\_00756  
CCNA\_02461  
CCNA\_00697  
CCNA\_01286  
CCNA\_00140  
CCNA\_00005

Supplement: Additional file 19: Figure S6 — Phylogenetic profiles and positions in MPD and MNTD coordinates for all modules. [file 1471-2164-14-450-S19.zip › FigureS6/lightcyan1.pdf]

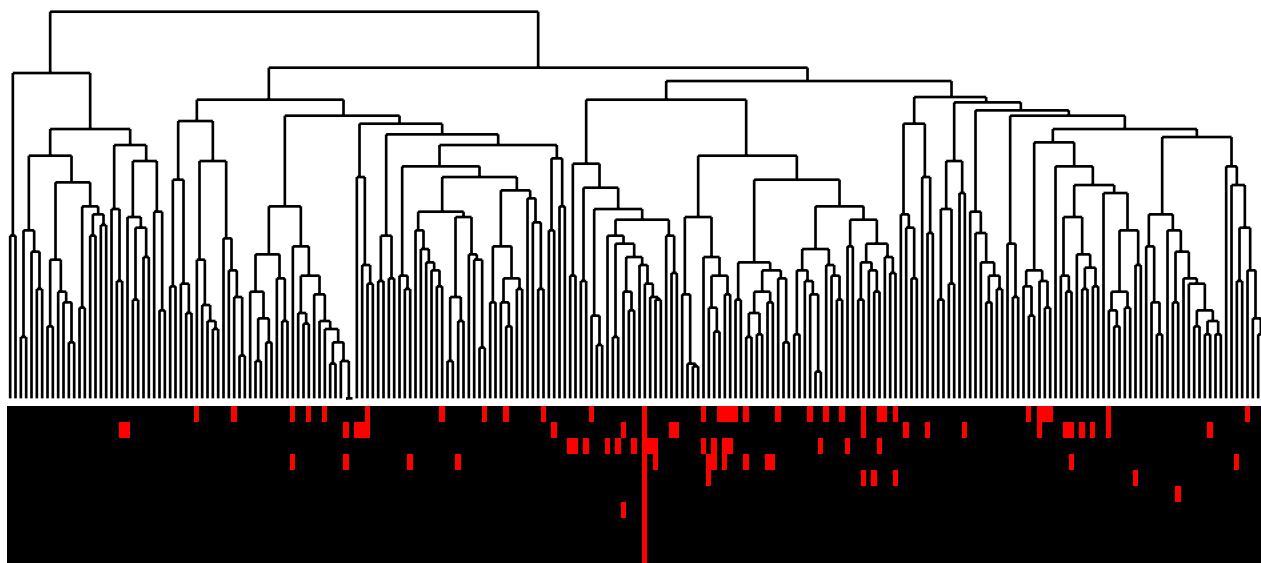

Supplement: Additional file 19: Figure S6 — Phylogenetic profiles and positions in MPD and MNTD coordinates for all modules. [file 1471-2164-14-450-S19.zip › FigureS6/lightpink4.pdf]
