# Supplementary figures and images for: Transcriptomic and phylogenetic analysis of a bacterial cell cycle reveals strong associations between gene co-expression and evolution (part 2 of 4)
Source: BMC Genomics. 2013 Jul 5;14:450. doi: 10.1186/1471-2164-14-450 (PMC3829707; doi:10.1186/1471-2164-14-450)

# CCNA\_00260

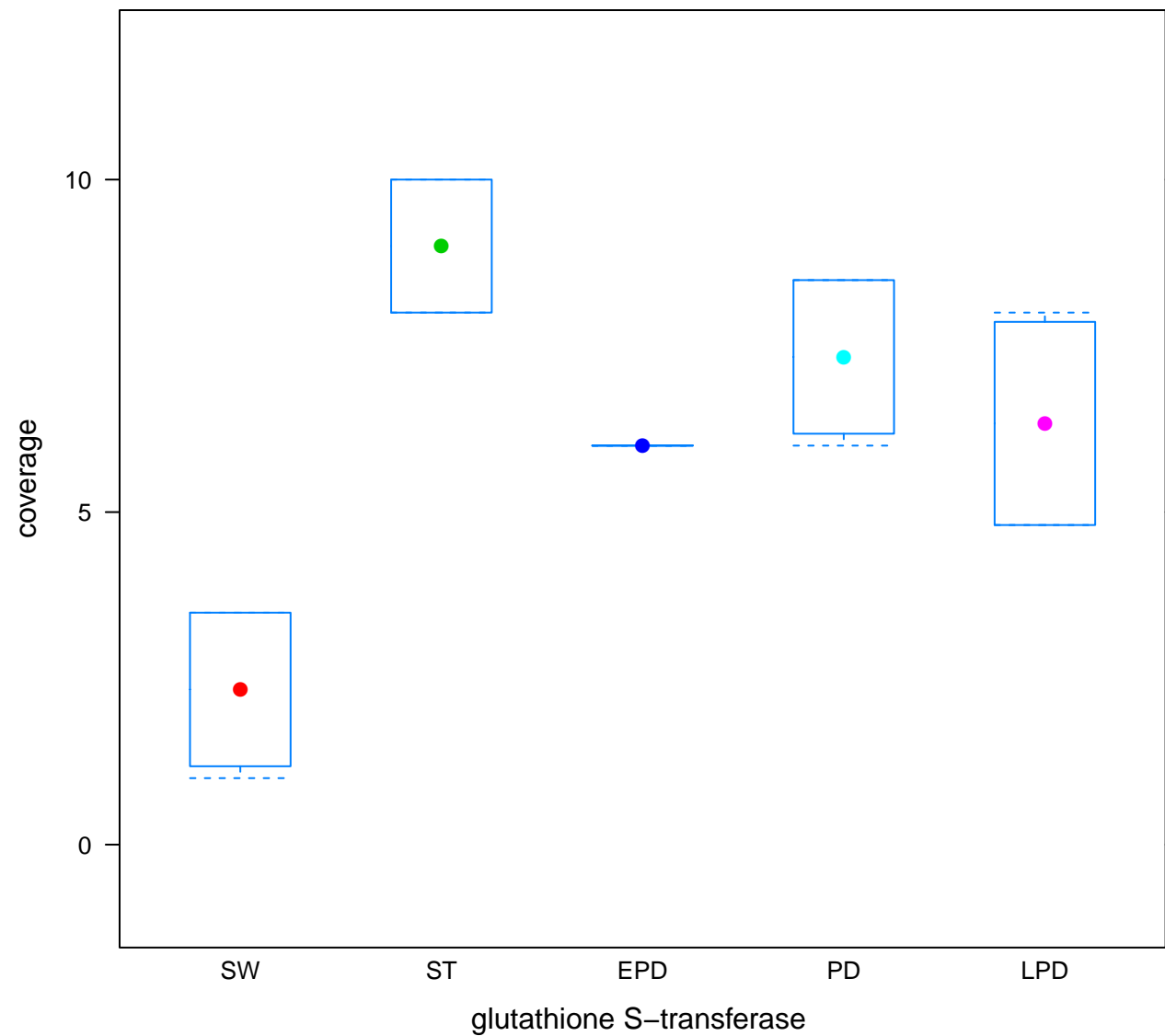

**Fold of change: 4.5**  
**baySeq likelihood: 0.831**

Supplement: Additional file 9: Figure S2 — Expression profiles of all identified CCR genes. [file 1471-2164-14-450-S9.zip › FigureS2/CCNA_00260.pdf]

# CCNA\_00261

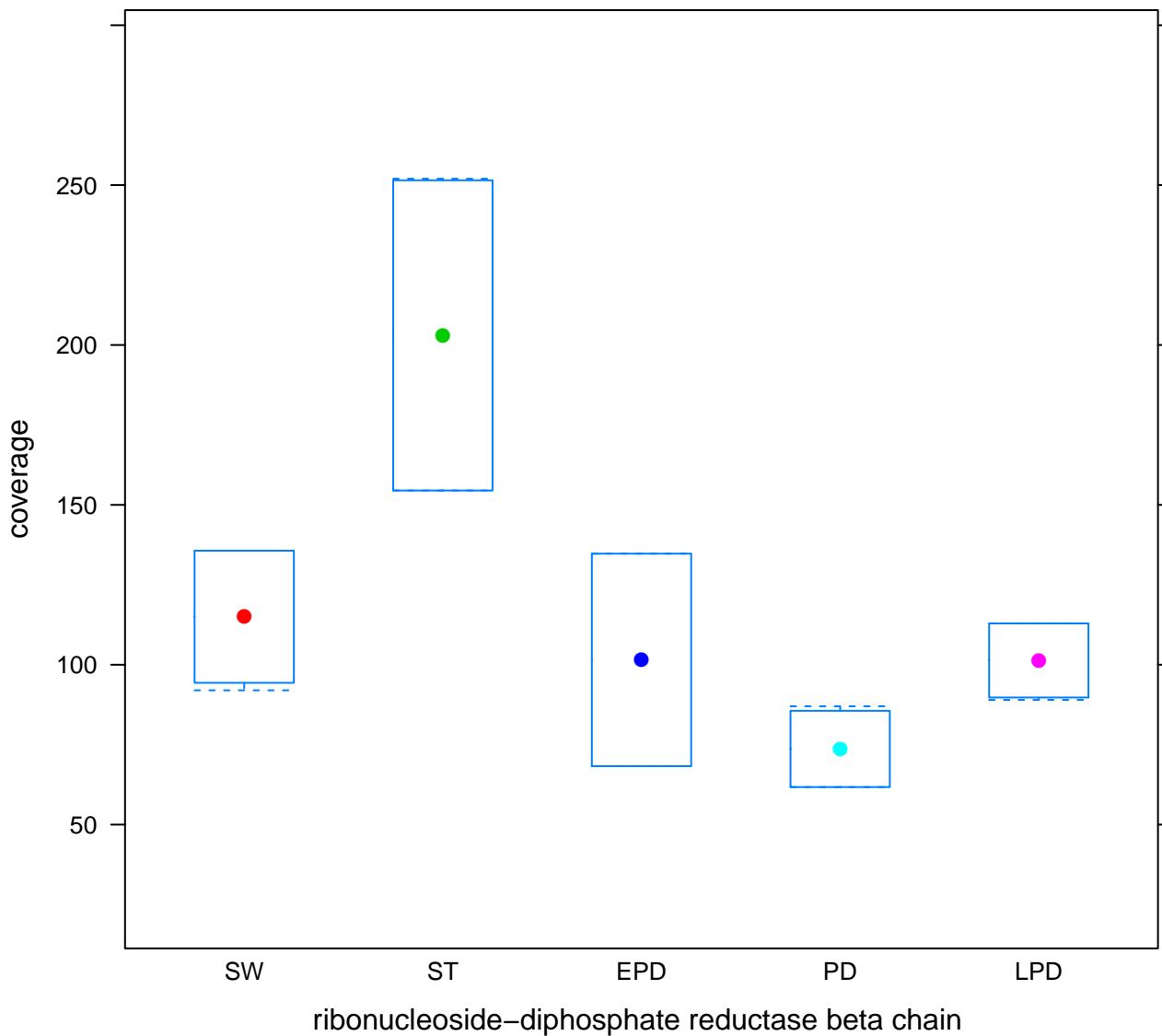

**Fold of change: 3.08**  
**baySeq likelihood: 0.581**

Supplement: Additional file 9: Figure S2 — Expression profiles of all identified CCR genes. [file 1471-2164-14-450-S9.zip › FigureS2/CCNA_00261.pdf]

# CCNA\_00262

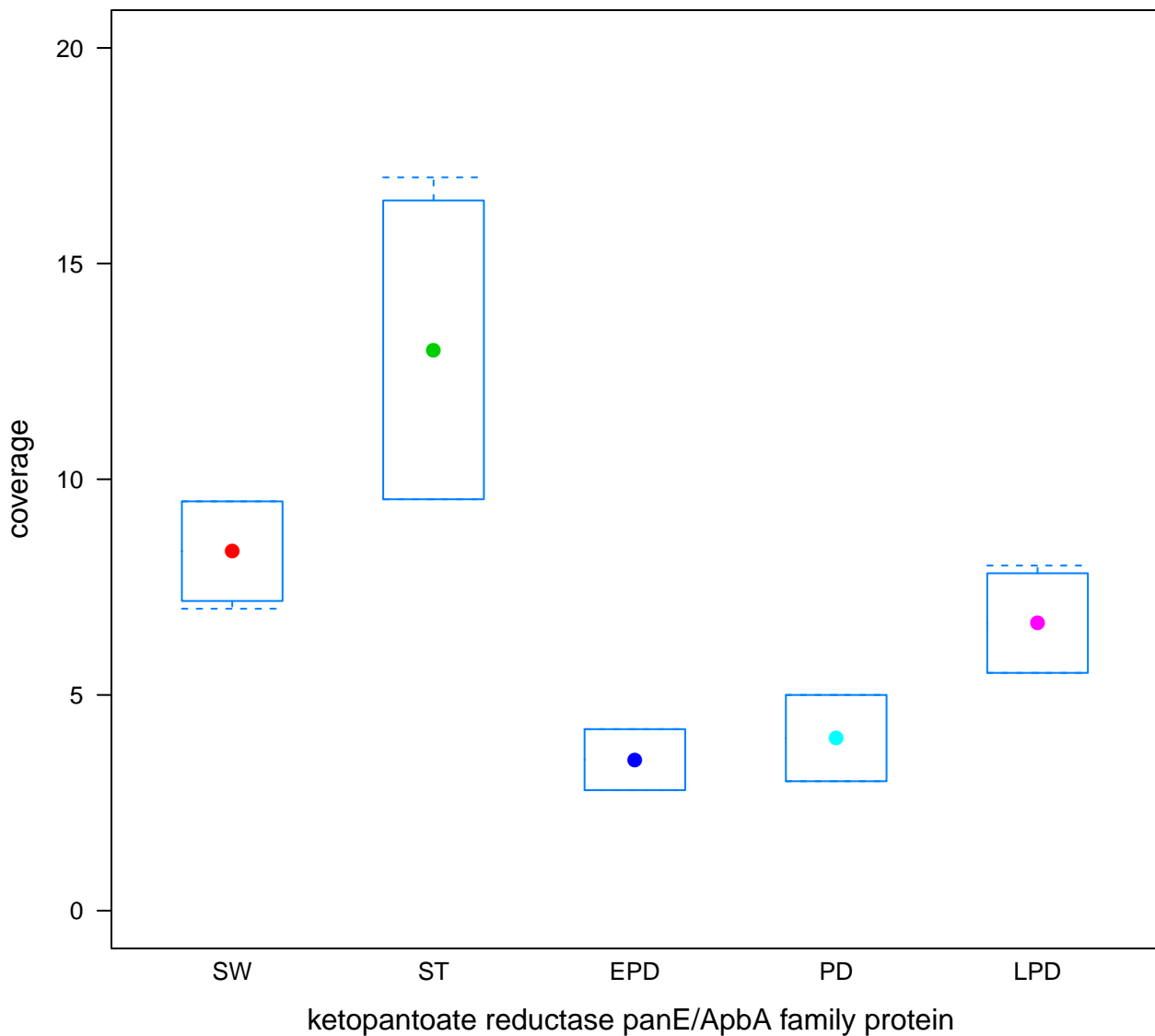

**Fold of change: 4**  
**baySeq likelihood: 0.773**

Supplement: Additional file 9: Figure S2 — Expression profiles of all identified CCR genes. [file 1471-2164-14-450-S9.zip › FigureS2/CCNA_00262.pdf]

# CCNA\_00264

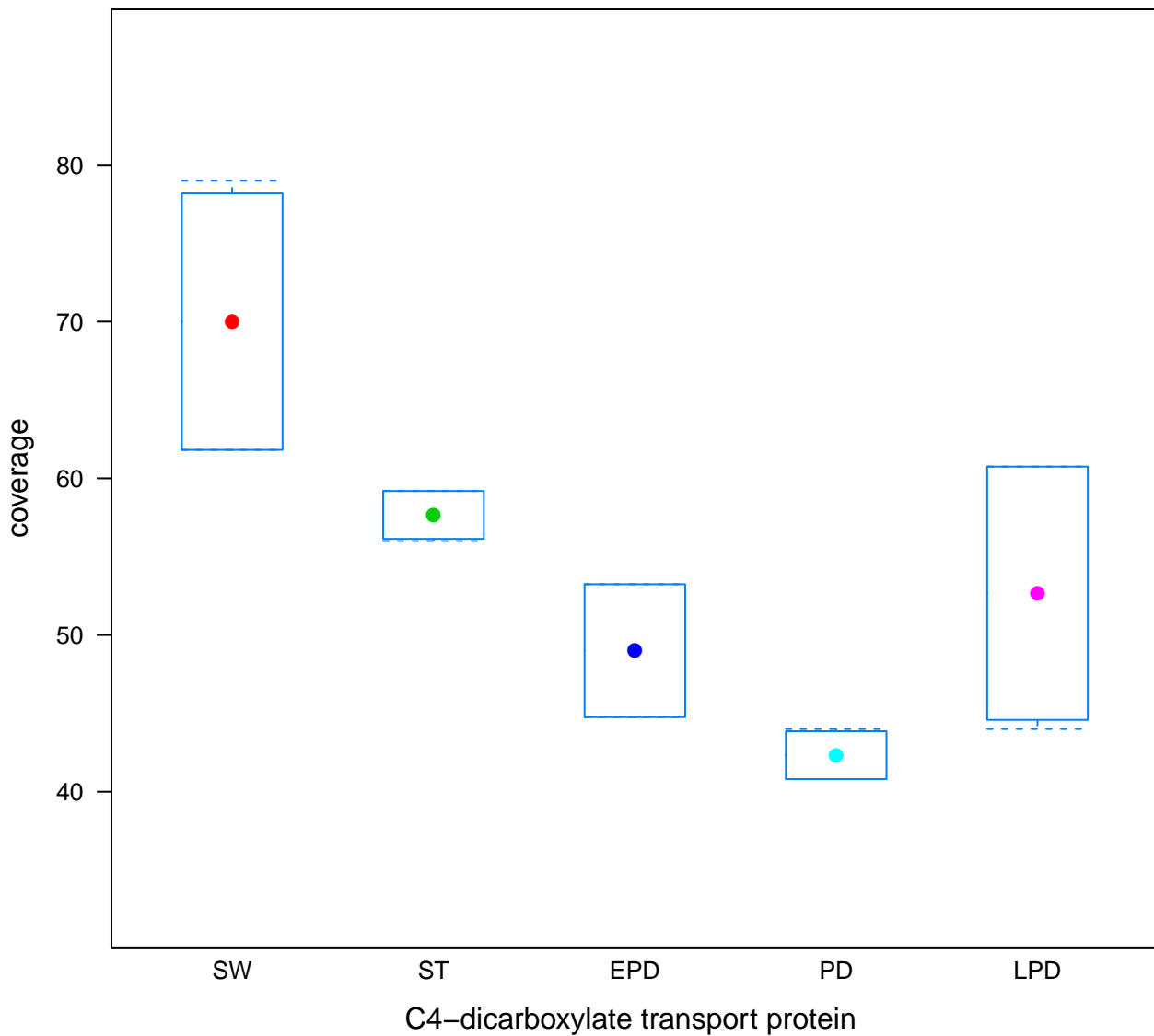

**Fold of change: 1.74**  
**baySeq likelihood: 0.699**

Supplement: Additional file 9: Figure S2 — Expression profiles of all identified CCR genes. [file 1471-2164-14-450-S9.zip › FigureS2/CCNA_00264.pdf]

# CCNA\_00265

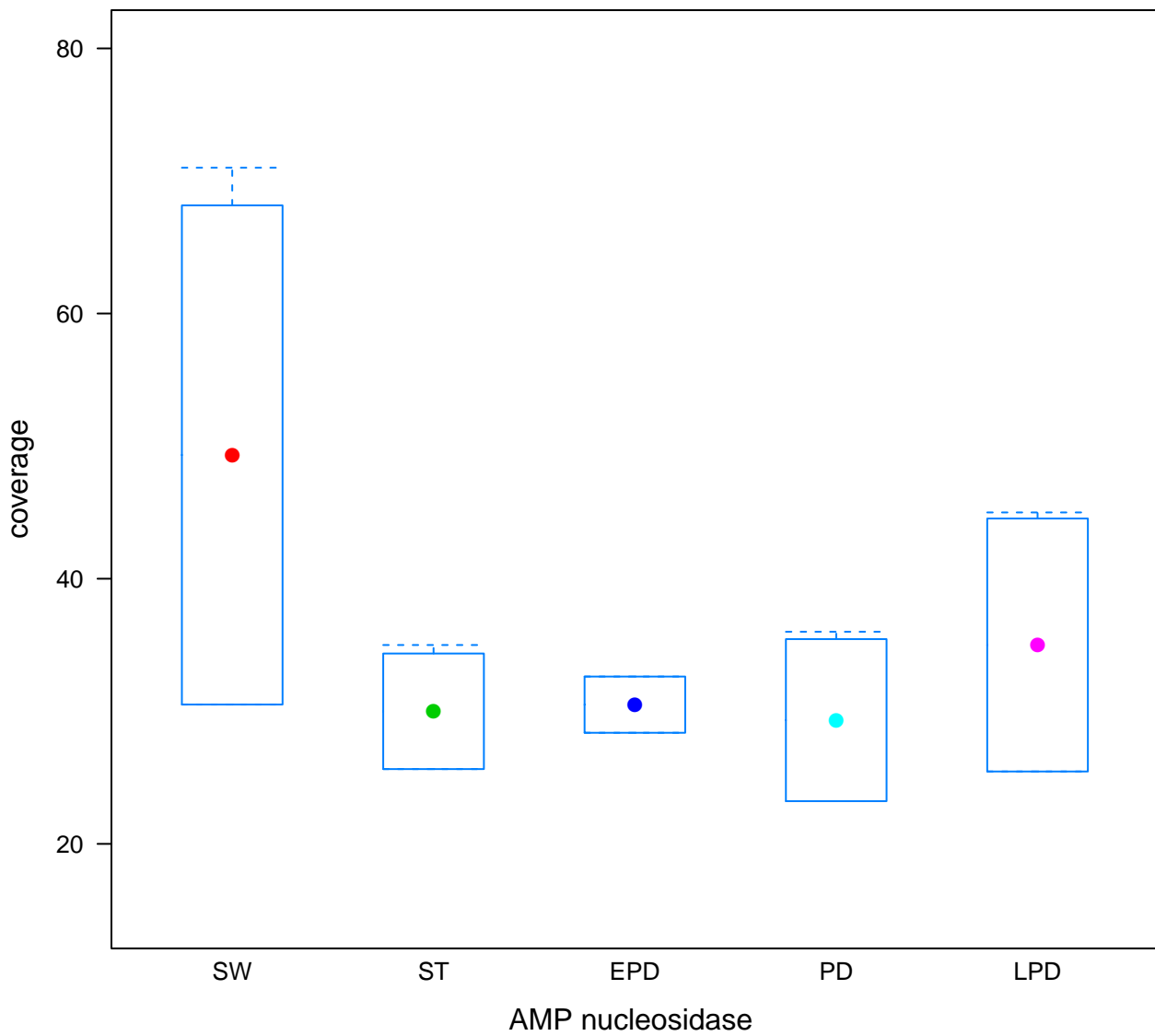

**Fold of change: 2.02**  
**baySeq likelihood: 0.794**

Supplement: Additional file 9: Figure S2 — Expression profiles of all identified CCR genes. [file 1471-2164-14-450-S9.zip › FigureS2/CCNA_00265.pdf]

# CCNA\_00269

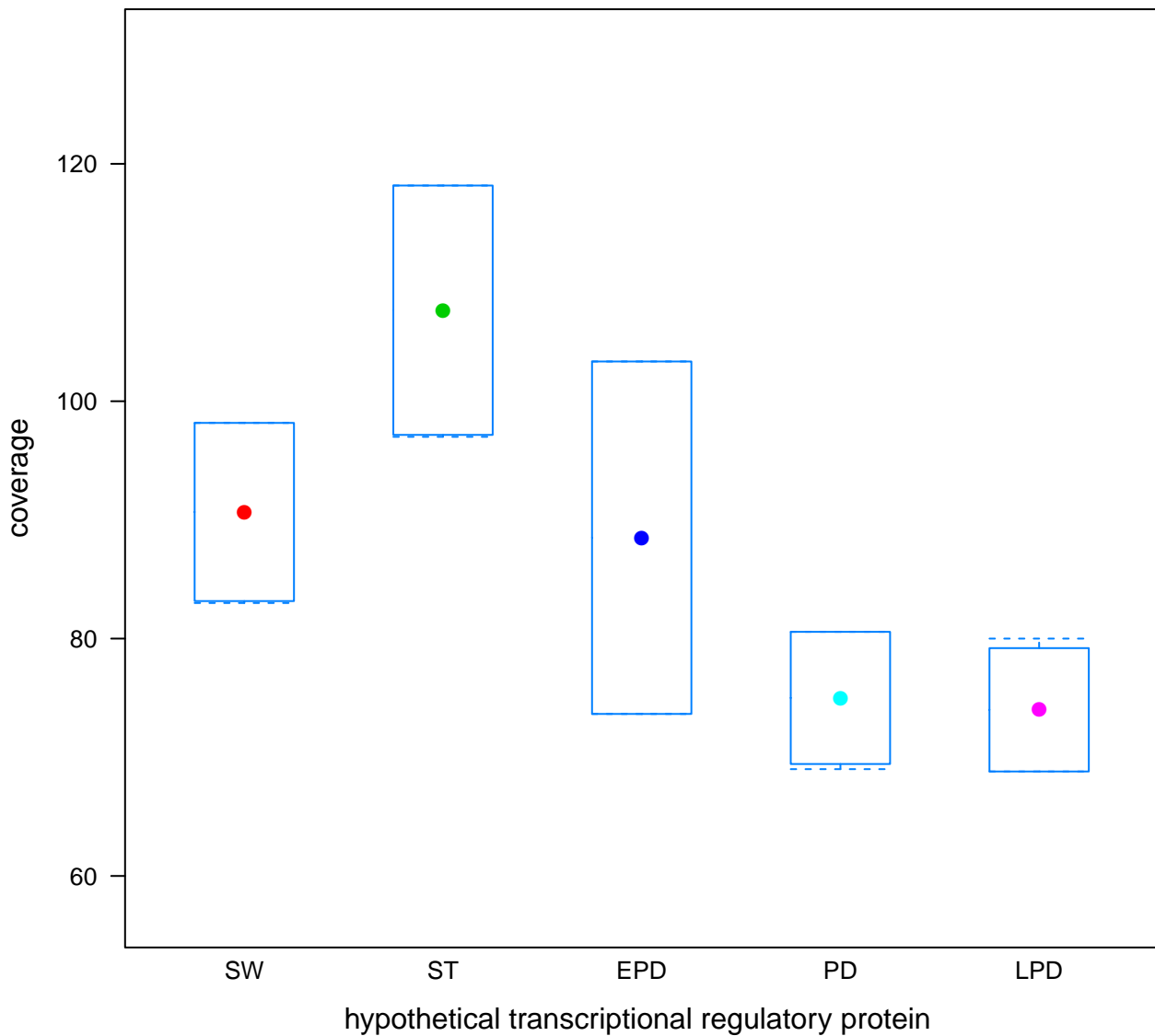

**Fold of change: 1.53**  
**baySeq likelihood: 0.279**

Supplement: Additional file 9: Figure S2 — Expression profiles of all identified CCR genes. [file 1471-2164-14-450-S9.zip › FigureS2/CCNA_00269.pdf]

# recR;CCNA\_00270

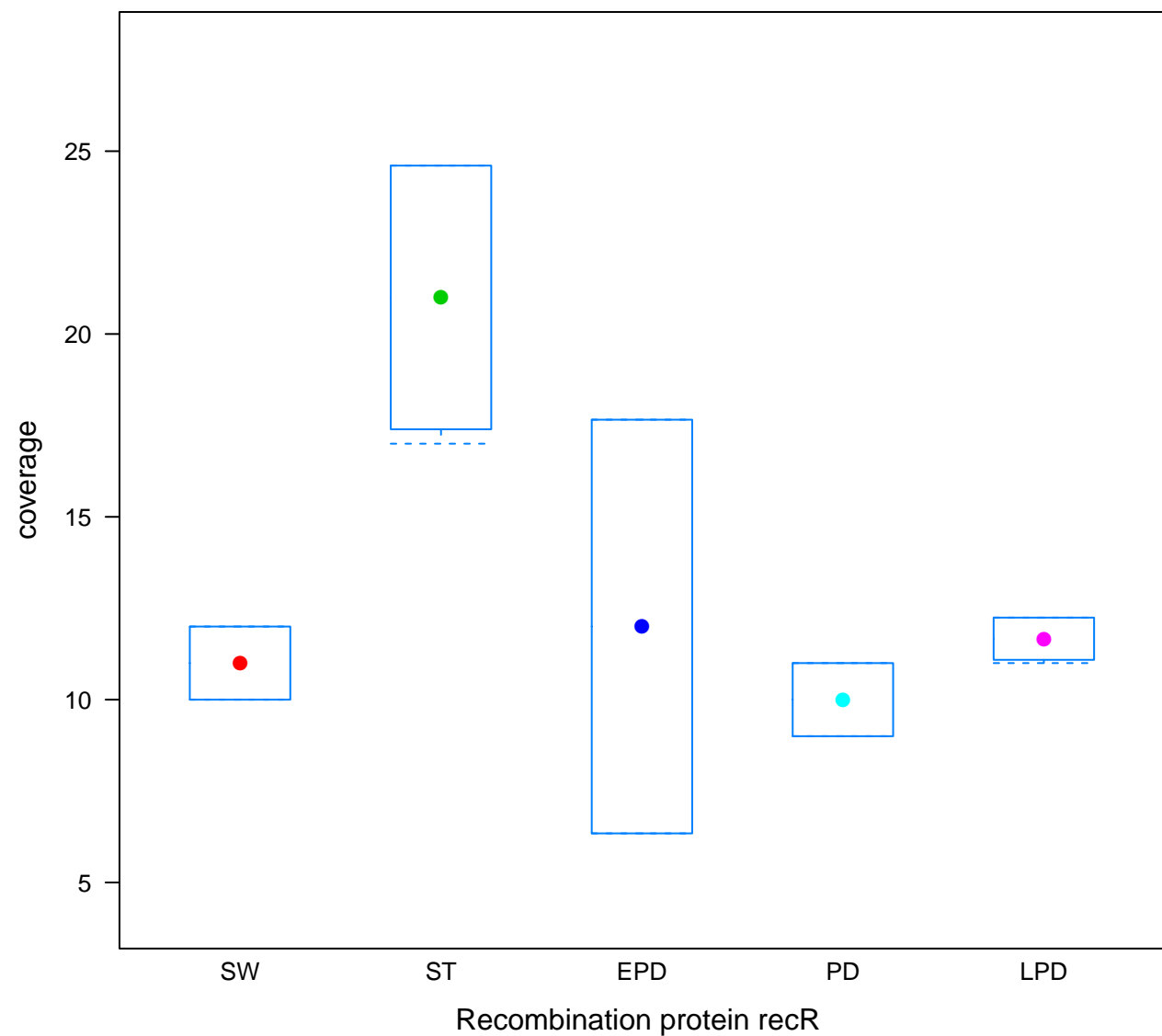

**Fold of change: 2.05**  
**baySeq likelihood: 0.721**

Supplement: Additional file 9: Figure S2 — Expression profiles of all identified CCR genes. [file 1471-2164-14-450-S9.zip › FigureS2/CCNA_00270.pdf]

# CCNA\_00279

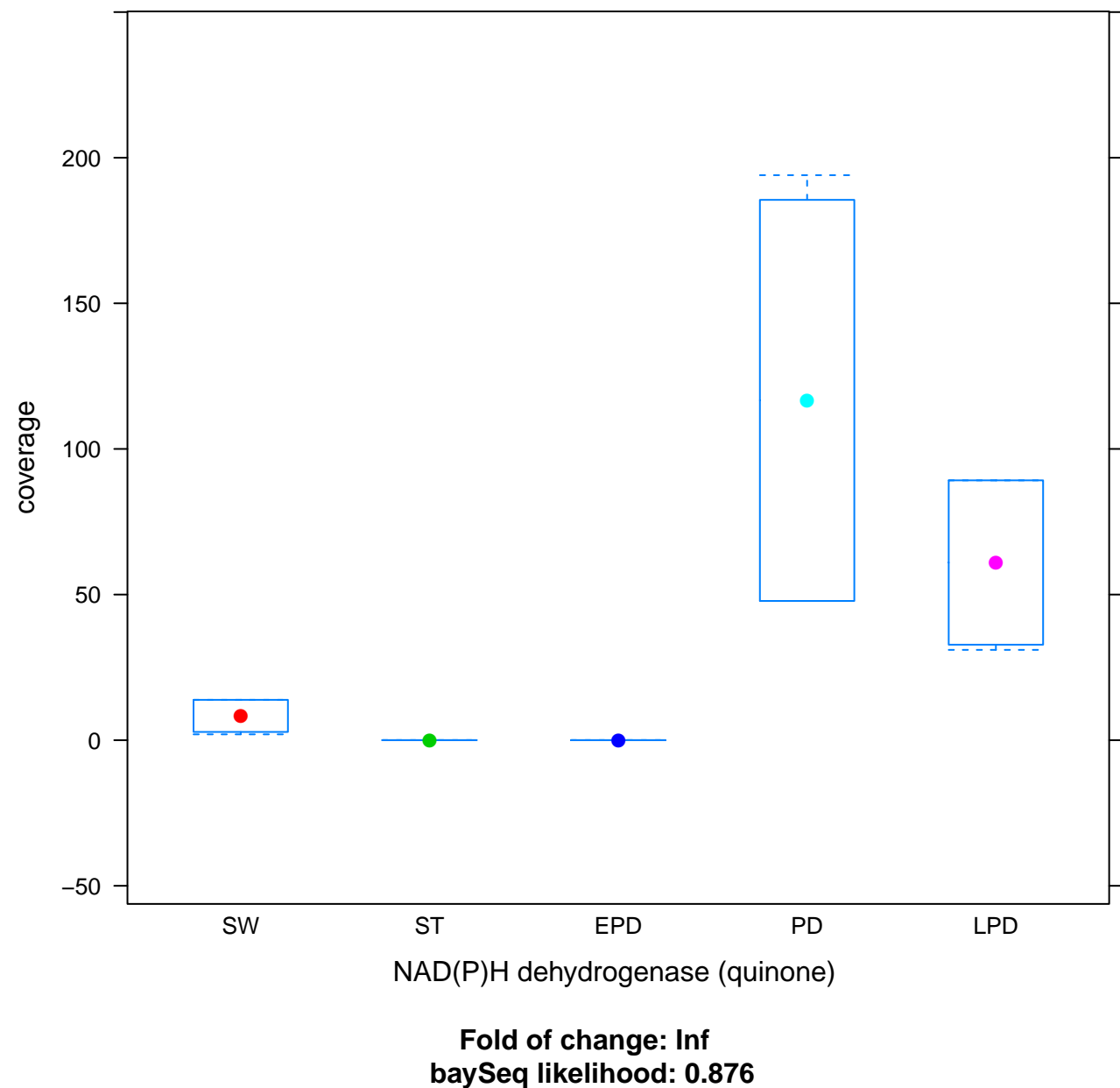

Supplement: Additional file 9: Figure S2 — Expression profiles of all identified CCR genes. [file 1471-2164-14-450-S9.zip › FigureS2/CCNA_00279.pdf]

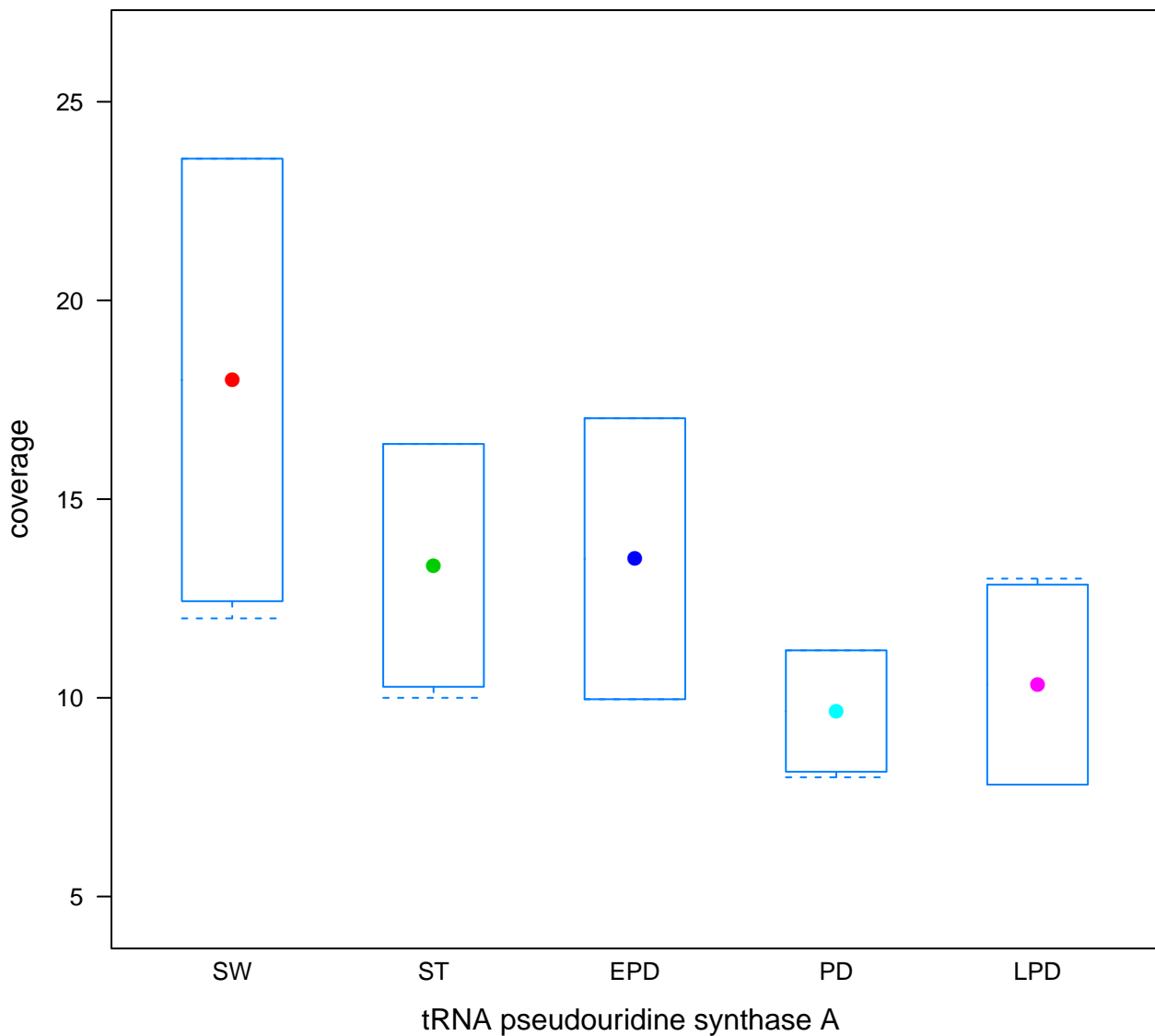

**Fold of change: 2.17**  
**baySeq likelihood: 0.516**

Supplement: Additional file 9: Figure S2 — Expression profiles of all identified CCR genes. [file 1471-2164-14-450-S9.zip › FigureS2/CCNA_00280.pdf]

# CCNA\_00282

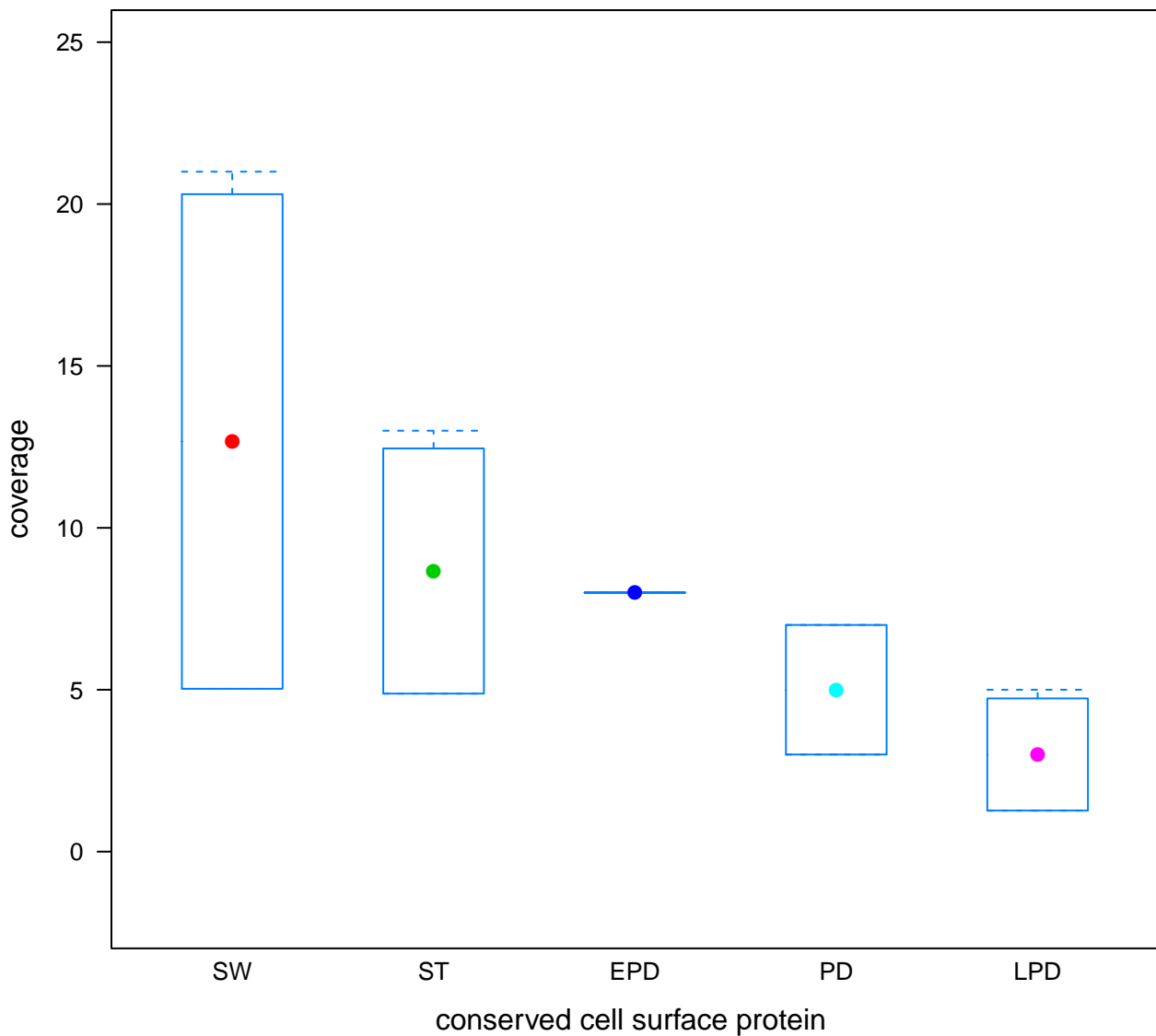

**Fold of change: 2.83**  
**baySeq likelihood: 0.439**

Supplement: Additional file 9: Figure S2 — Expression profiles of all identified CCR genes. [file 1471-2164-14-450-S9.zip › FigureS2/CCNA_00282.pdf]

# CCNA\_00285

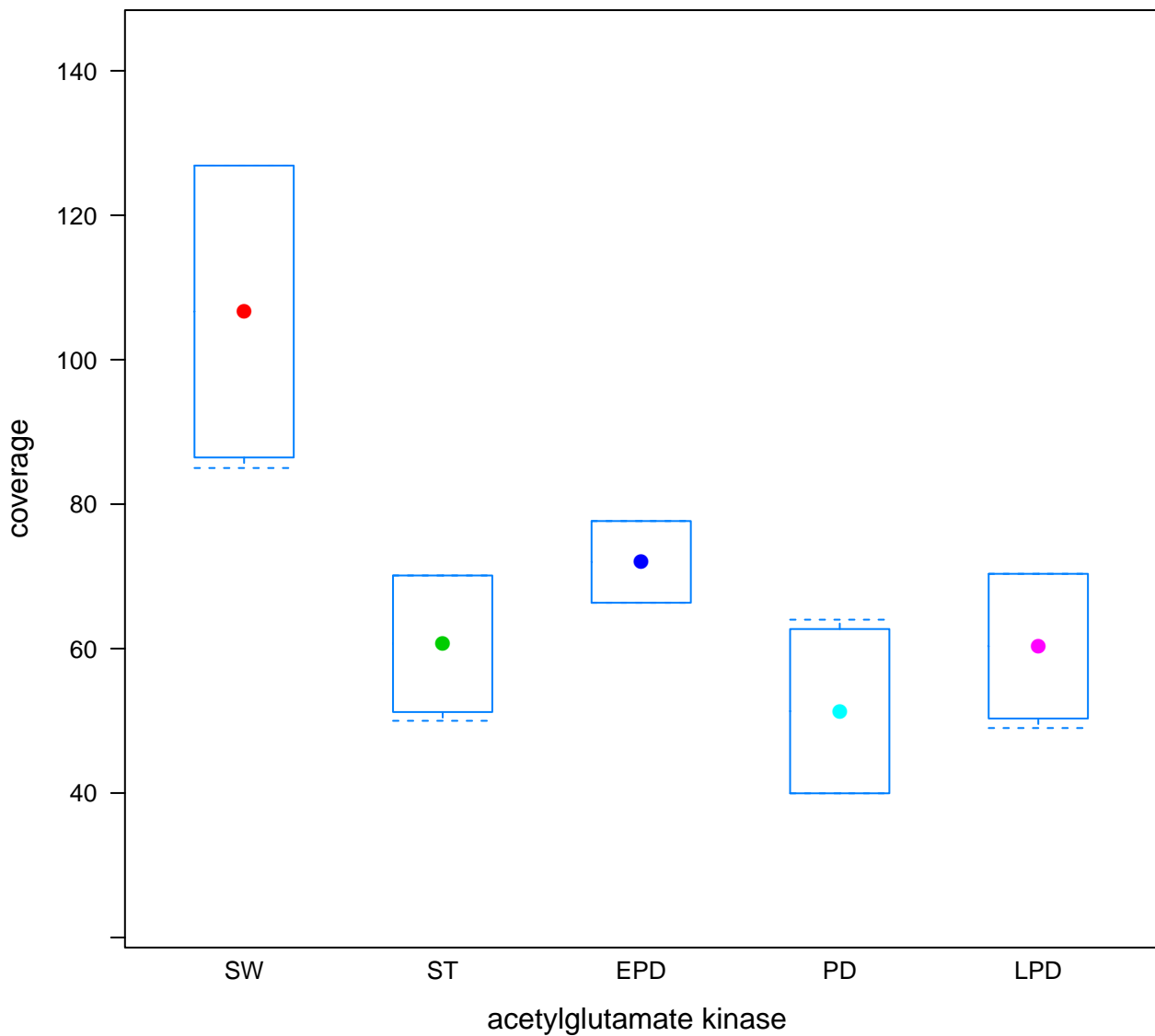

**Fold of change: 2.05**  
**baySeq likelihood: 0.966**

Supplement: Additional file 9: Figure S2 — Expression profiles of all identified CCR genes. [file 1471-2164-14-450-S9.zip › FigureS2/CCNA_00285.pdf]

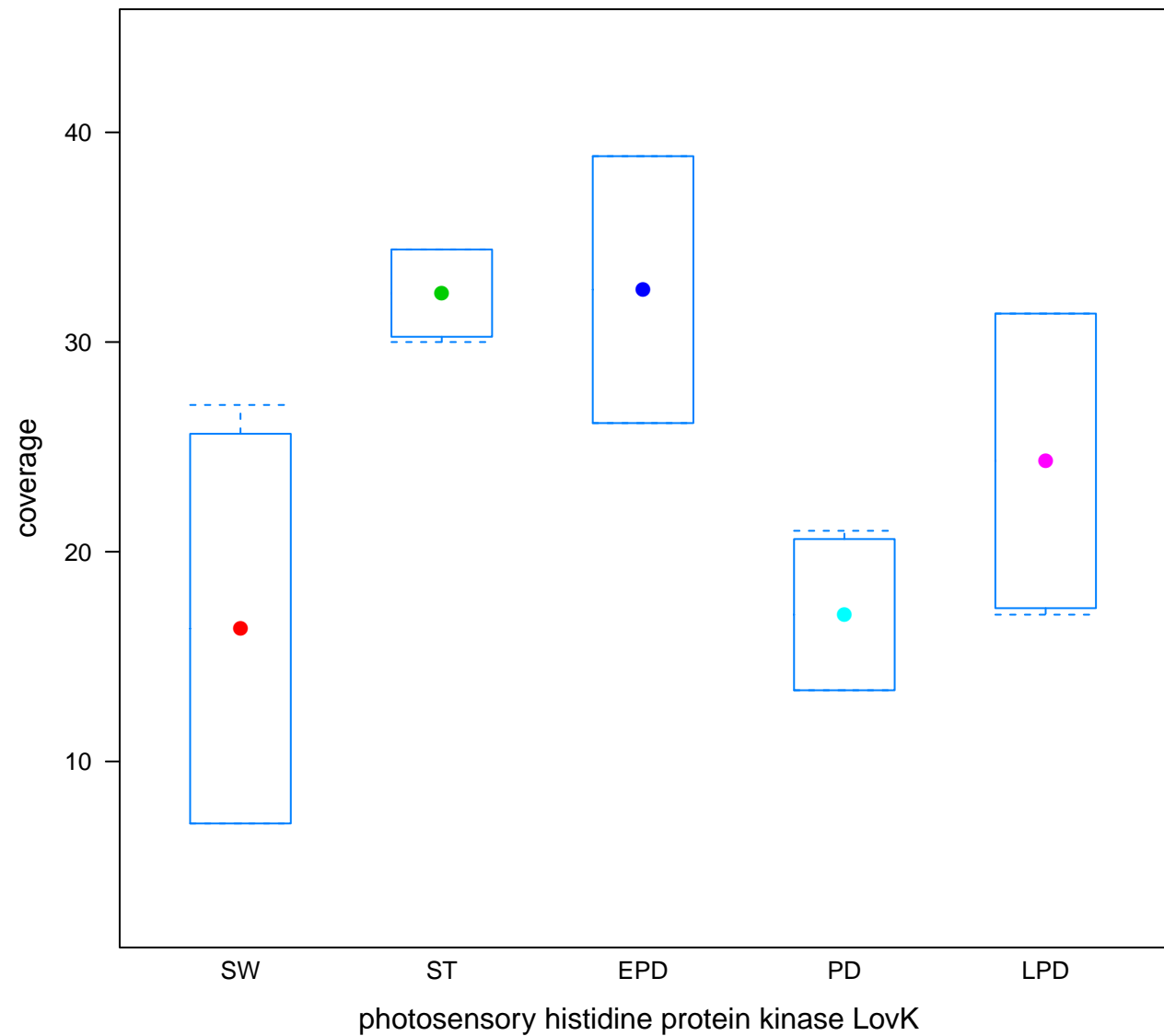

**Fold of change: 2.95**  
**baySeq likelihood: 0.599**

Supplement: Additional file 9: Figure S2 — Expression profiles of all identified CCR genes. [file 1471-2164-14-450-S9.zip › FigureS2/CCNA_00287.pdf]

# CCNA\_00288

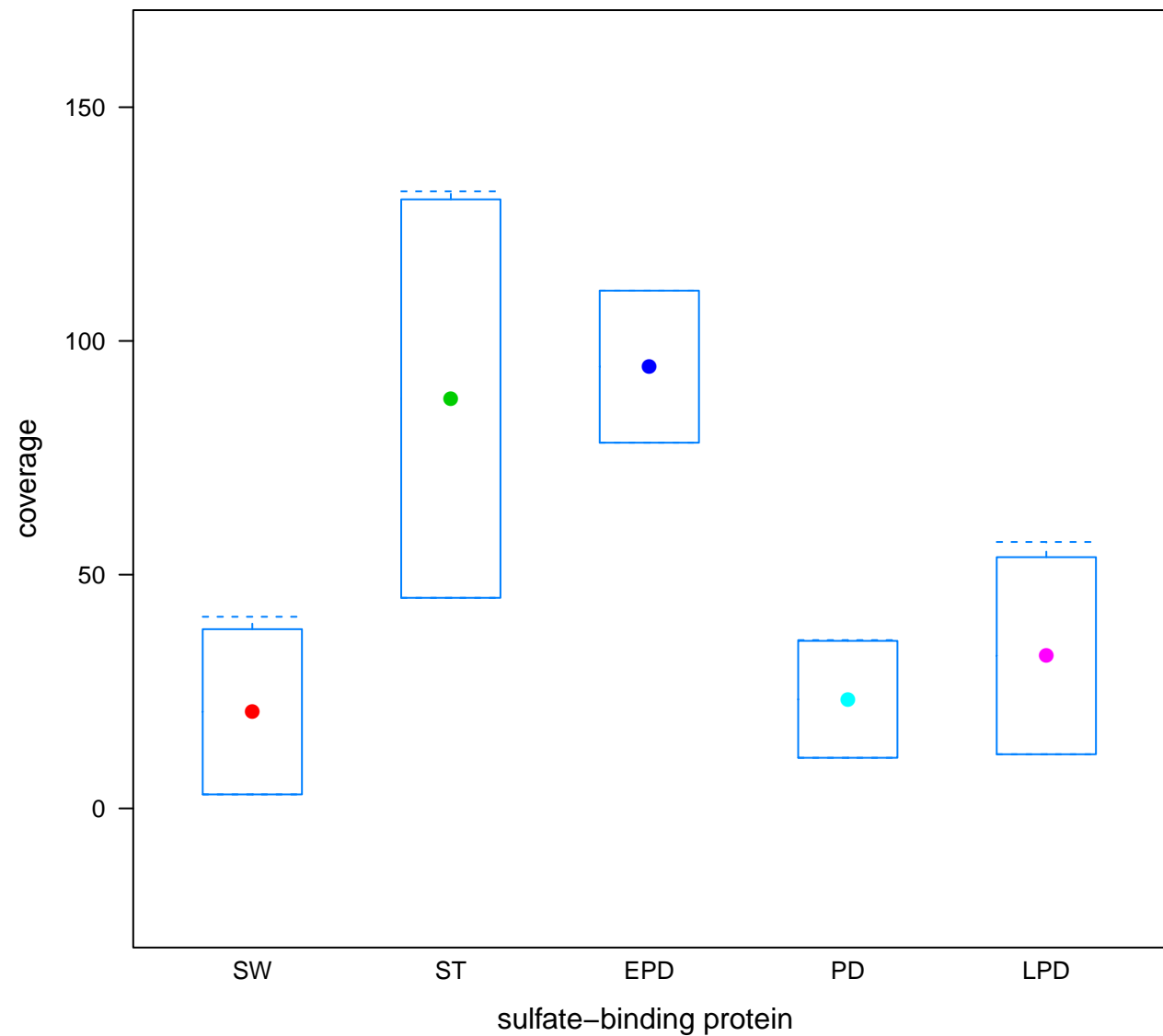

**Fold of change: 10.29**  
**baySeq likelihood: 0.946**

Supplement: Additional file 9: Figure S2 — Expression profiles of all identified CCR genes. [file 1471-2164-14-450-S9.zip › FigureS2/CCNA_00288.pdf]

# CCNA\_00289

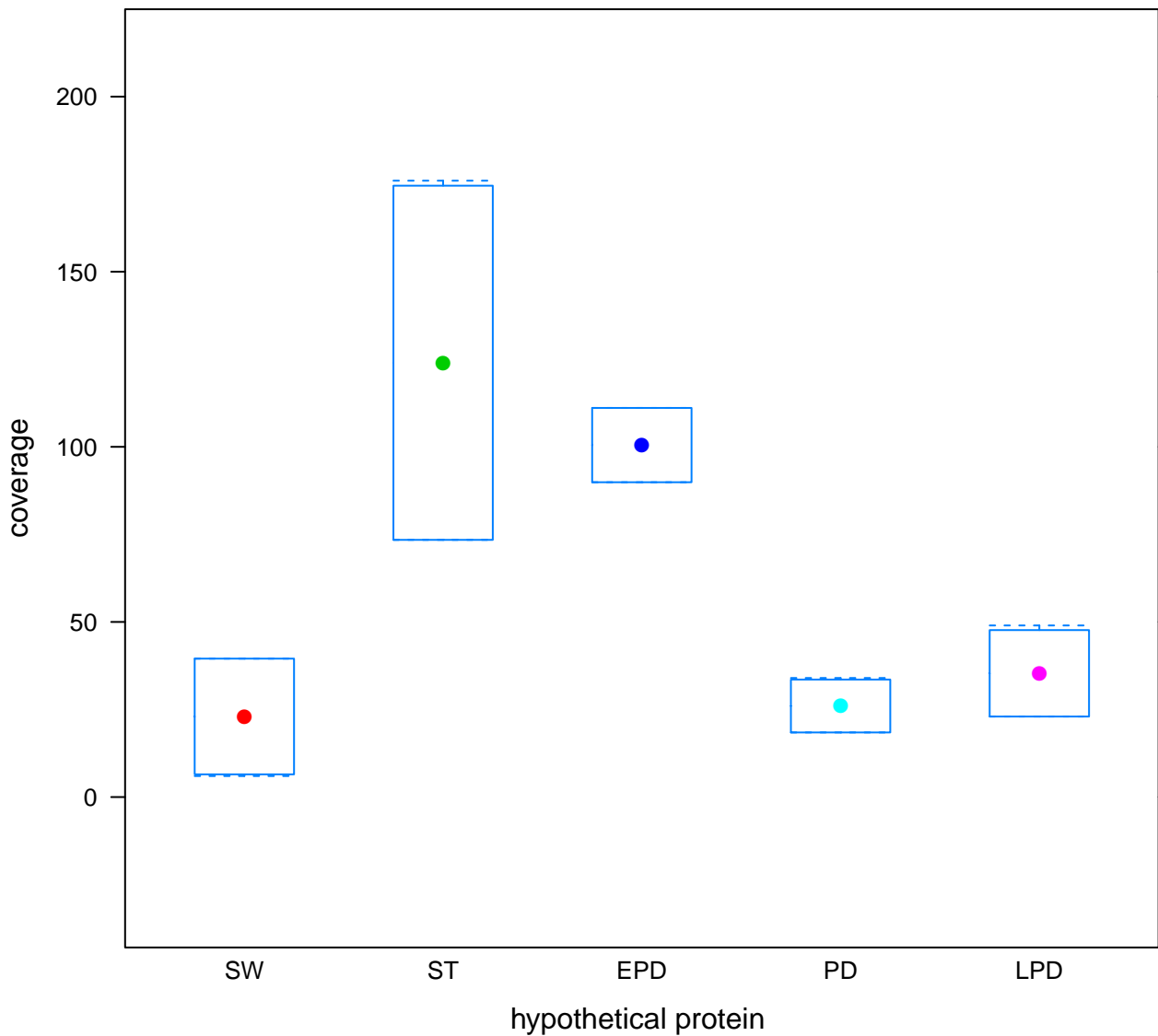

**Fold of change: 9.9**  
**baySeq likelihood: 0.994**

Supplement: Additional file 9: Figure S2 — Expression profiles of all identified CCR genes. [file 1471-2164-14-450-S9.zip › FigureS2/CCNA_00289.pdf]

# phoR;CCNA\_00291

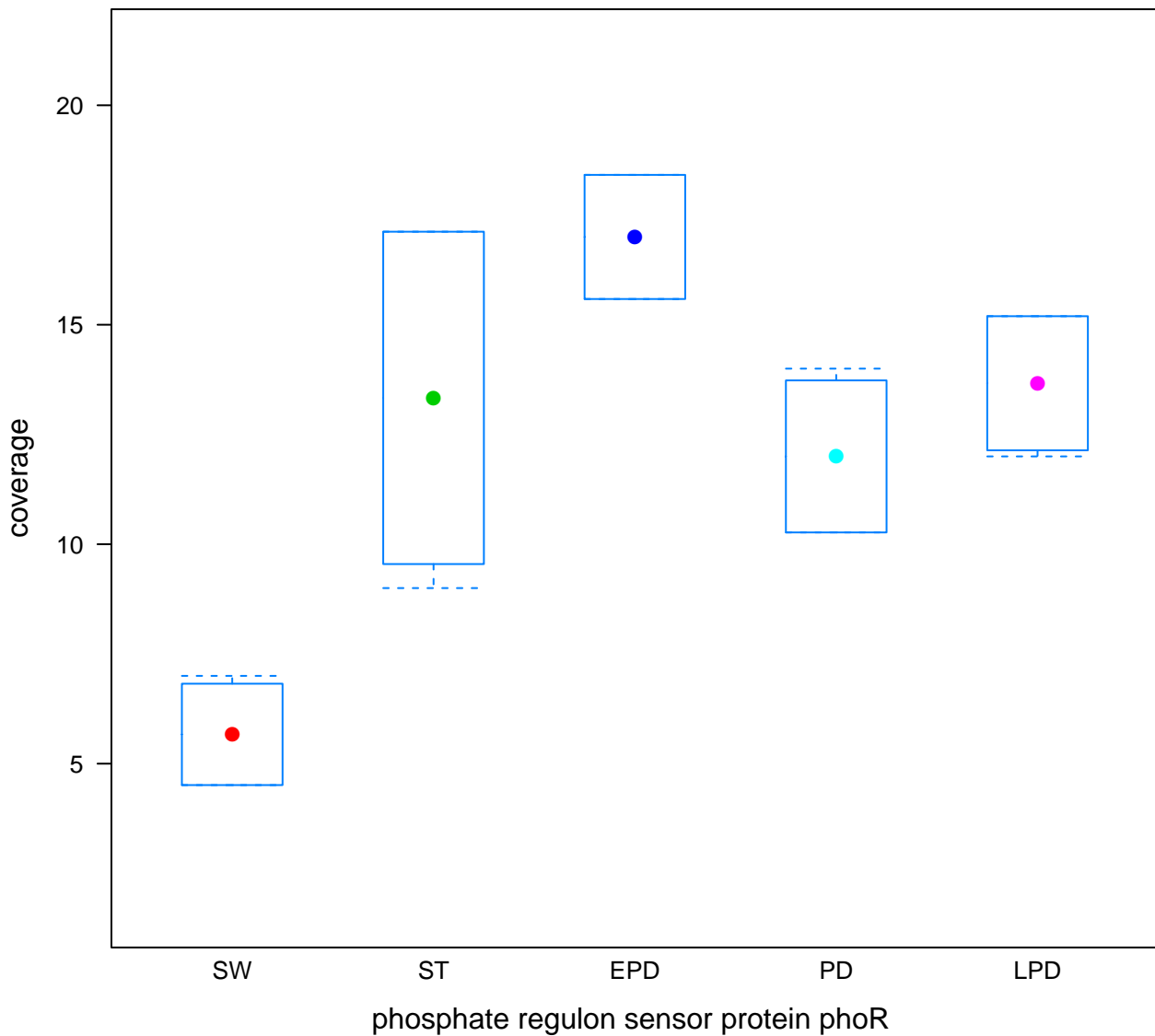

**Fold of change: 3.4**  
**baySeq likelihood: 0.949**

Supplement: Additional file 9: Figure S2 — Expression profiles of all identified CCR genes. [file 1471-2164-14-450-S9.zip › FigureS2/CCNA_00291.pdf]

# pstC;CCNA\_00292

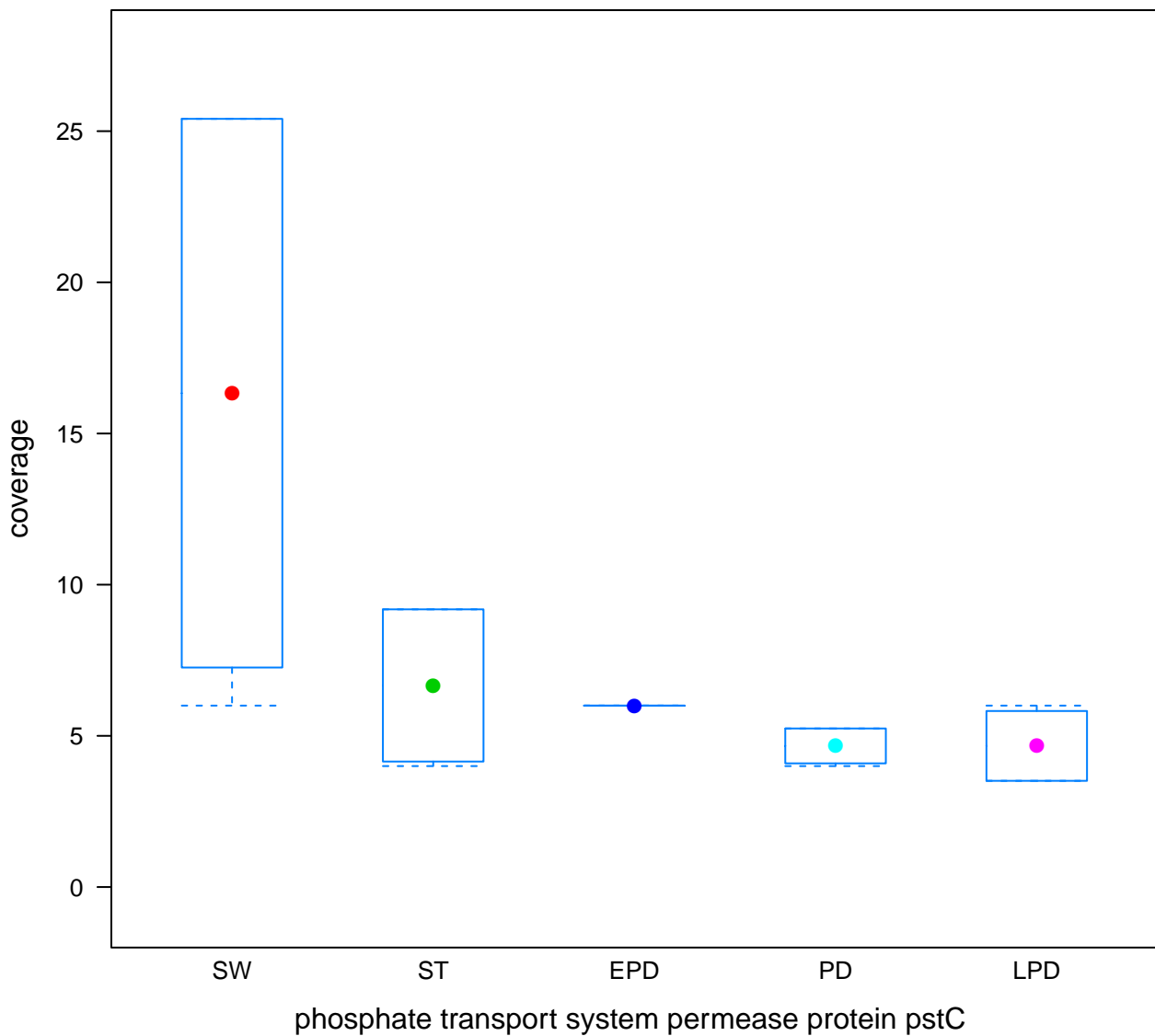

**Fold of change: 4.61**  
**baySeq likelihood: 0.987**

Supplement: Additional file 9: Figure S2 — Expression profiles of all identified CCR genes. [file 1471-2164-14-450-S9.zip › FigureS2/CCNA_00292.pdf]

# pstA;CCNA\_00293

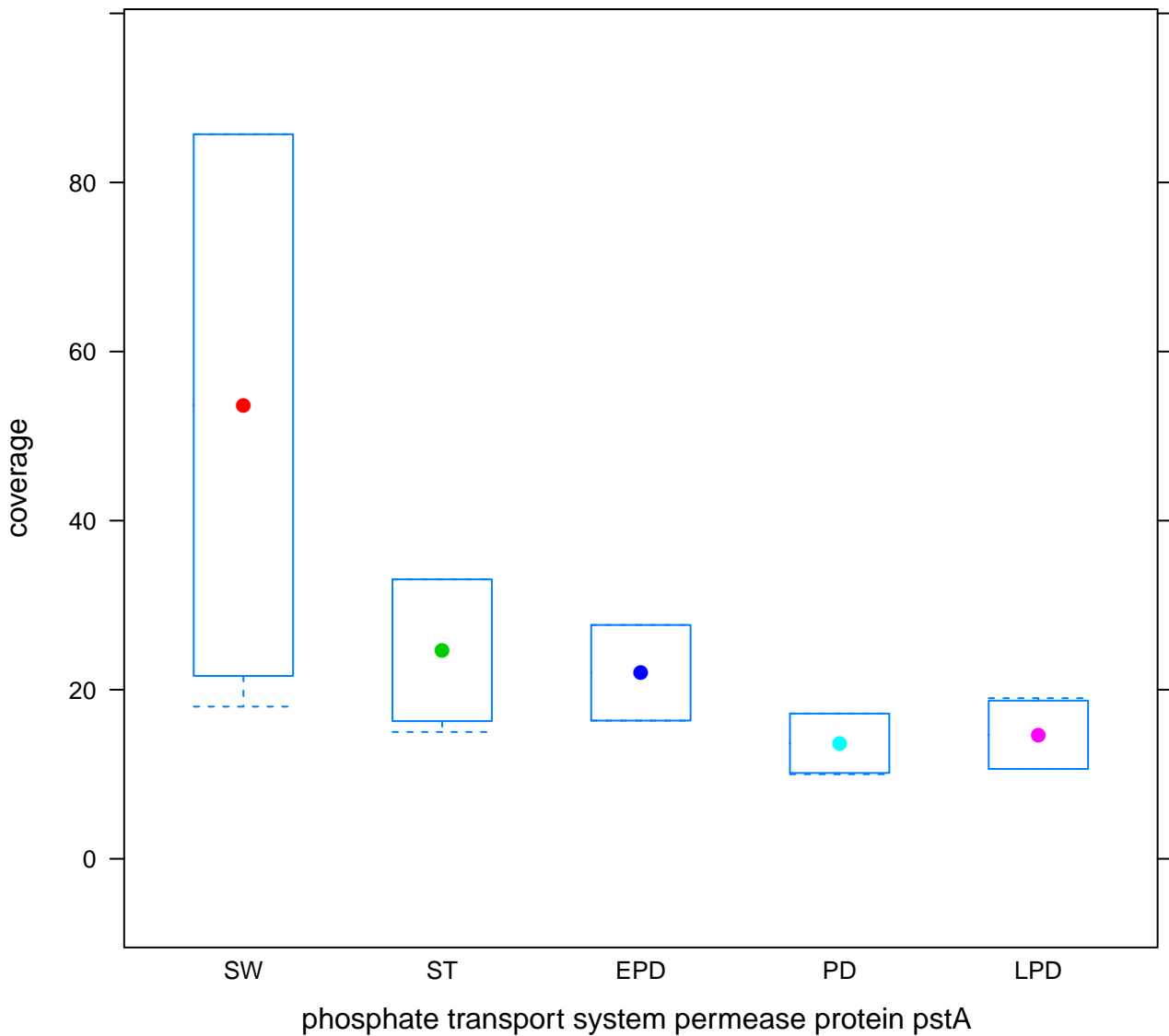

**Fold of change: 5.23**  
**baySeq likelihood: 0.437**

Supplement: Additional file 9: Figure S2 — Expression profiles of all identified CCR genes. [file 1471-2164-14-450-S9.zip › FigureS2/CCNA_00293.pdf]

# pstB;CCNA\_00294

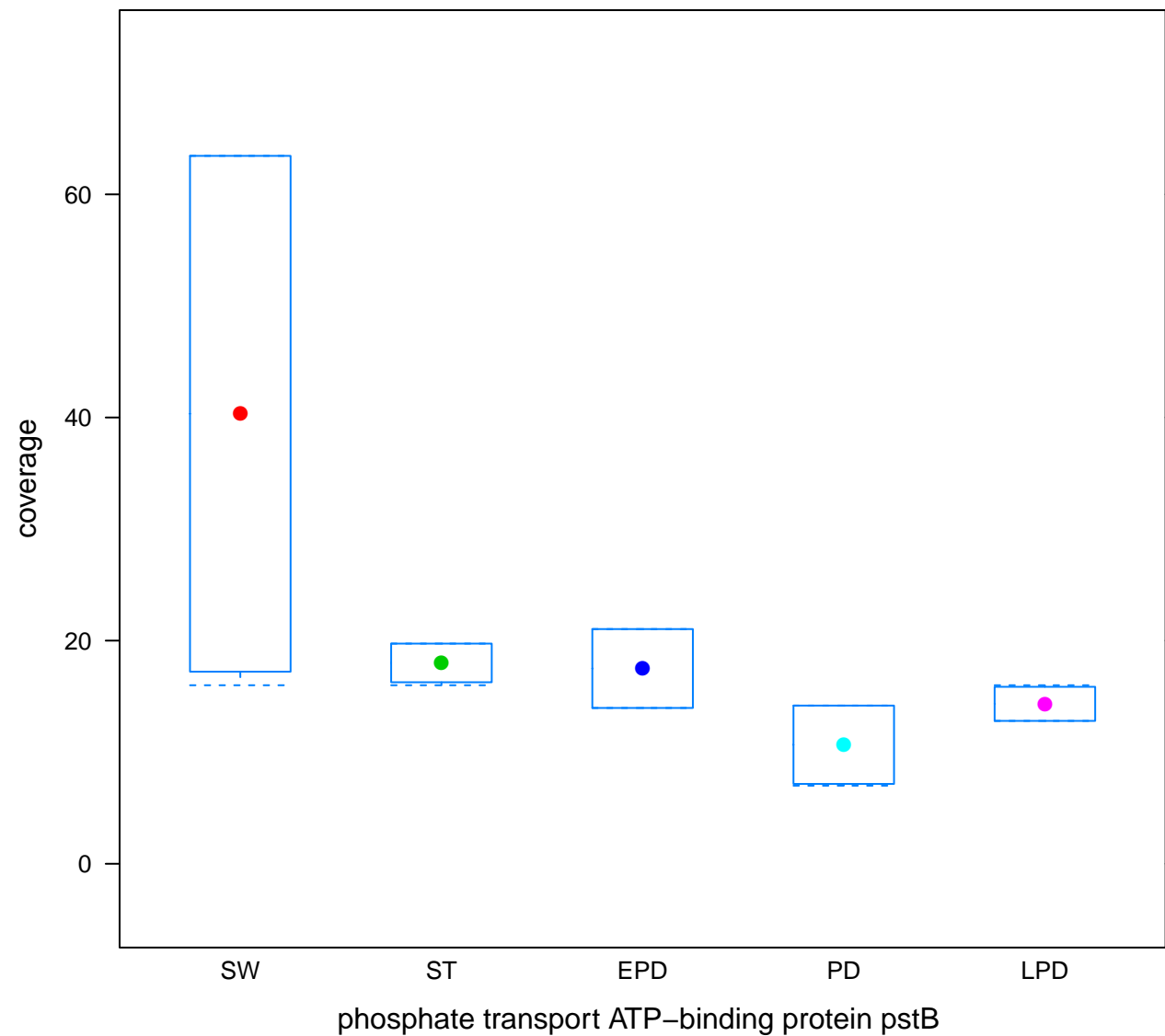

**Fold of change: 4.92**  
**baySeq likelihood: 0.978**

Supplement: Additional file 9: Figure S2 — Expression profiles of all identified CCR genes. [file 1471-2164-14-450-S9.zip › FigureS2/CCNA_00294.pdf]

# phoU;CCNA\_00295

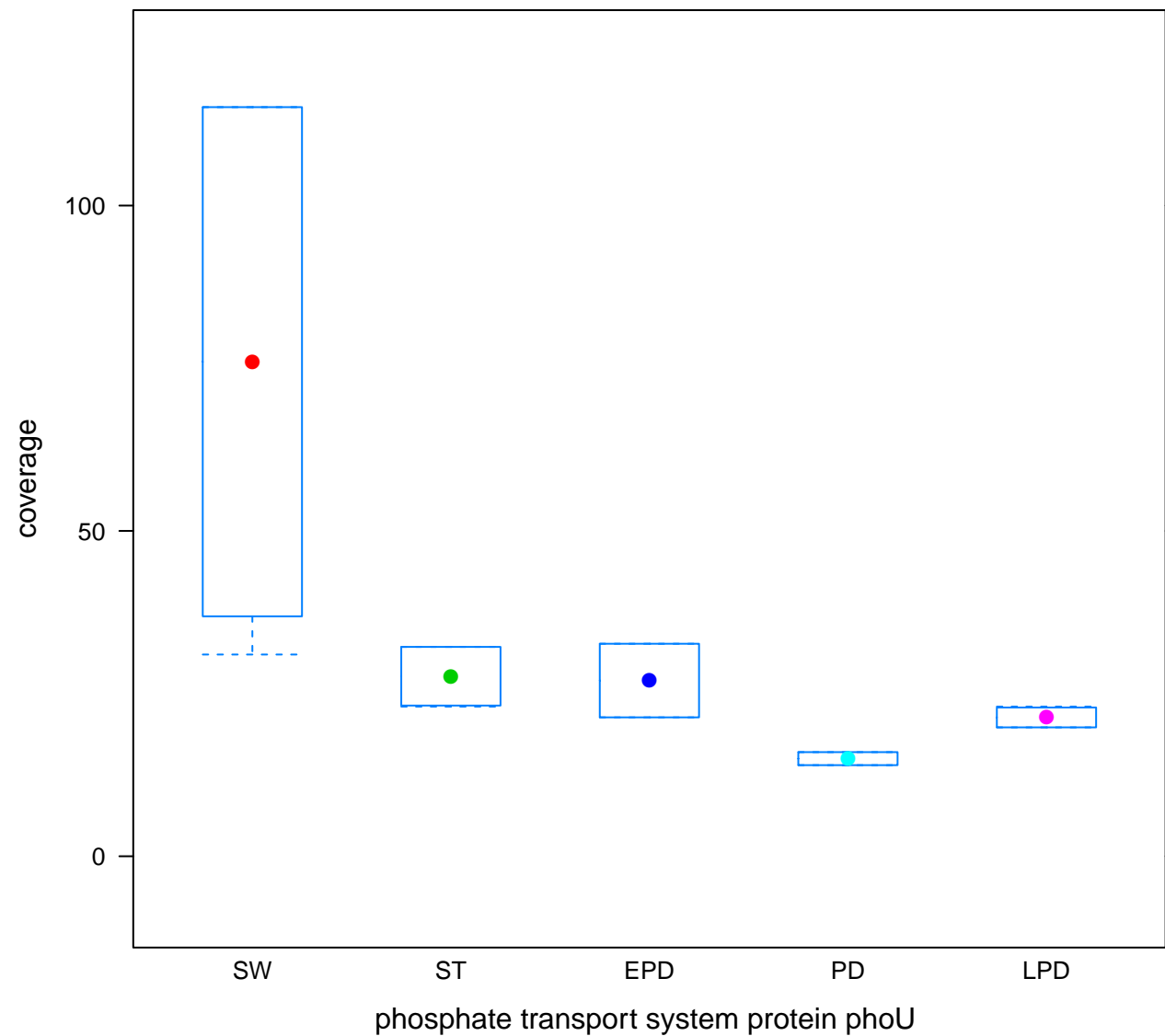

**Fold of change: 6.57**  
**baySeq likelihood: 0.985**

Supplement: Additional file 9: Figure S2 — Expression profiles of all identified CCR genes. [file 1471-2164-14-450-S9.zip › FigureS2/CCNA_00295.pdf]

# phoB;CCNA\_00296

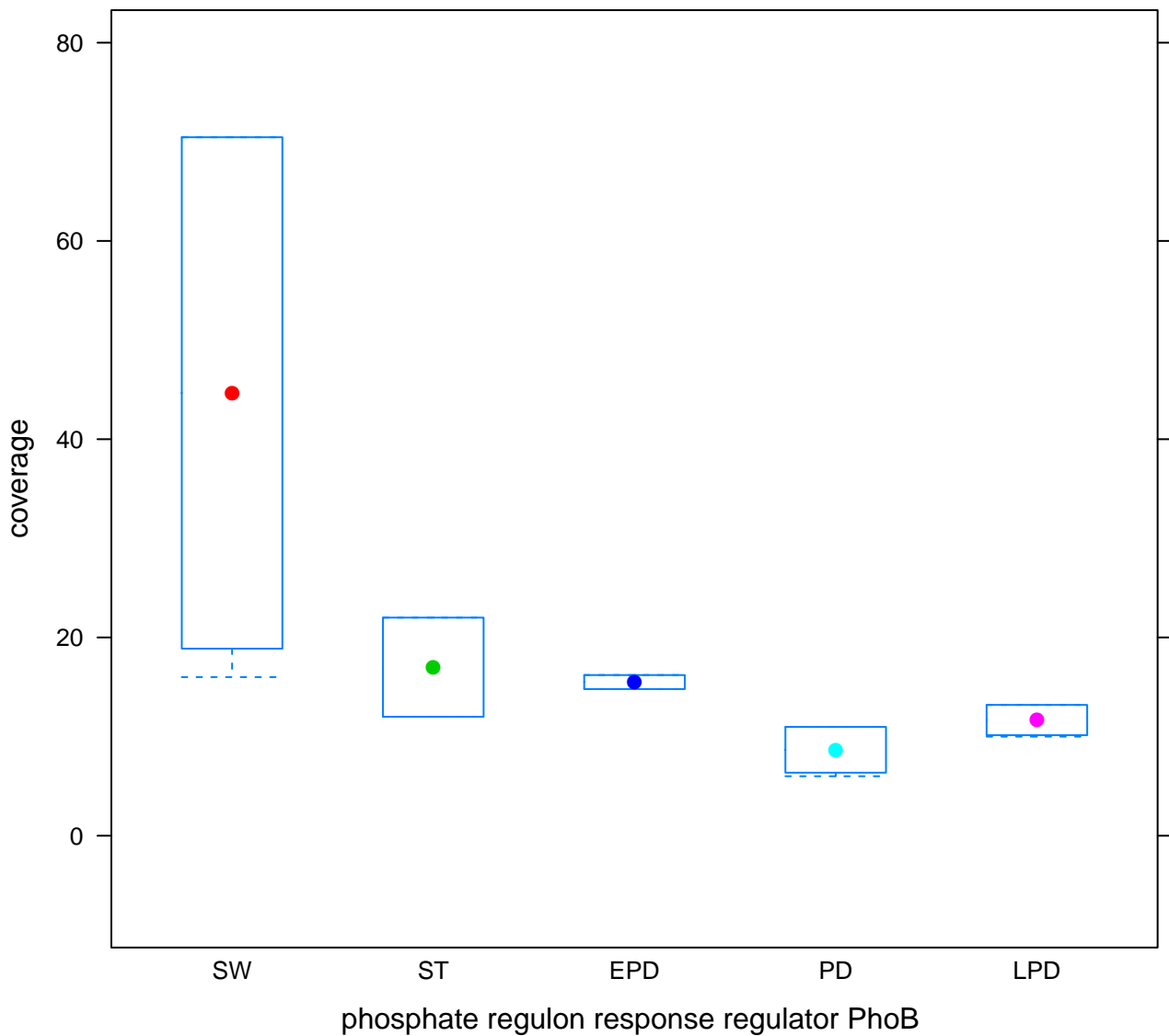

**Fold of change: 6.81**  
**baySeq likelihood: 0.944**

Supplement: Additional file 9: Figure S2 — Expression profiles of all identified CCR genes. [file 1471-2164-14-450-S9.zip › FigureS2/CCNA_00296.pdf]

# CCNA\_00299

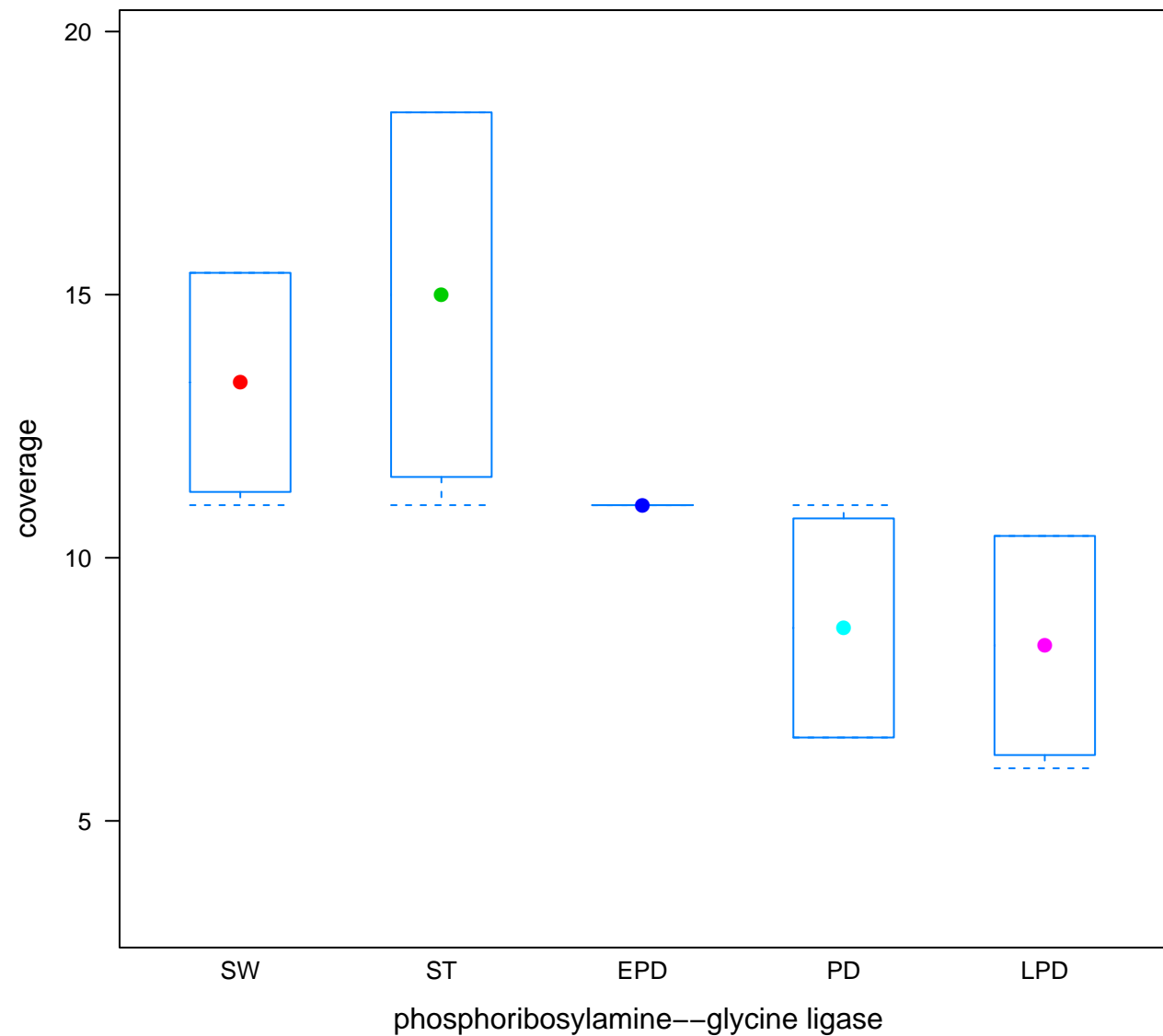

**Fold of change: 2.04**  
**baySeq likelihood: 0.31**

Supplement: Additional file 9: Figure S2 — Expression profiles of all identified CCR genes. [file 1471-2164-14-450-S9.zip › FigureS2/CCNA_00299.pdf]

# CCNA\_00301

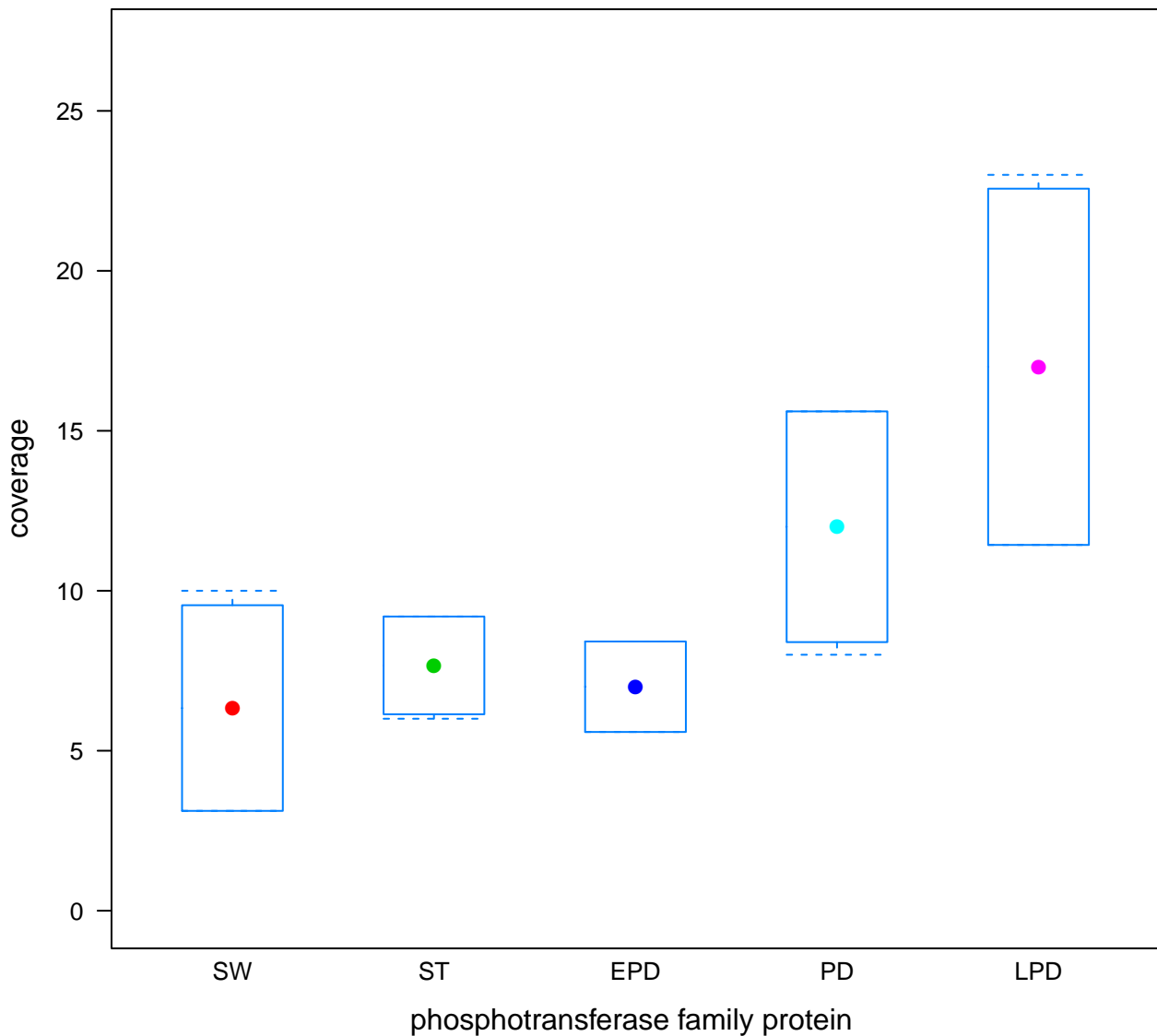

**Fold of change: 3.78**  
**baySeq likelihood: 0.817**

Supplement: Additional file 9: Figure S2 — Expression profiles of all identified CCR genes. [file 1471-2164-14-450-S9.zip › FigureS2/CCNA_00301.pdf]

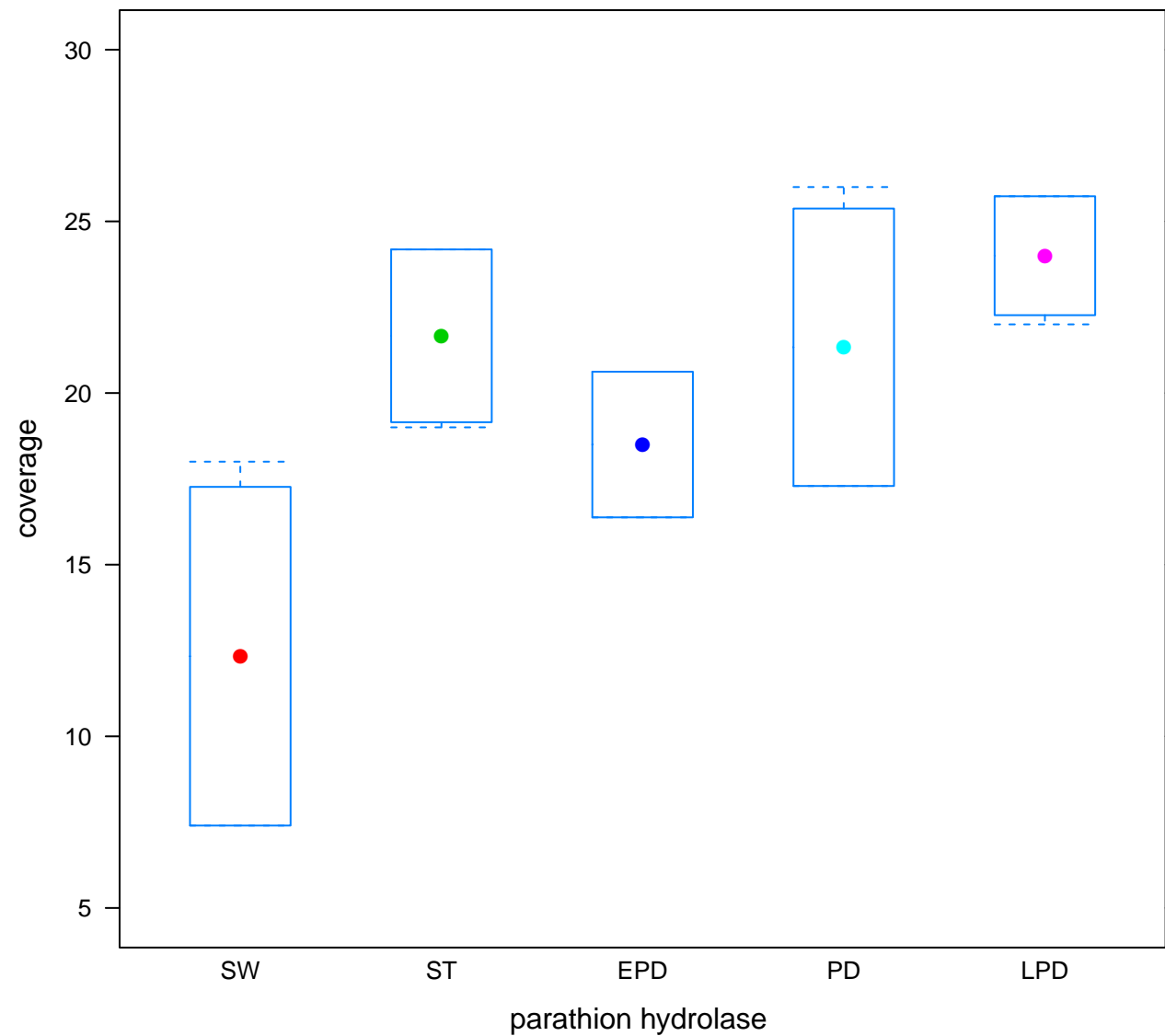

**Fold of change: 2.53**  
**baySeq likelihood: 0.729**

Supplement: Additional file 9: Figure S2 — Expression profiles of all identified CCR genes. [file 1471-2164-14-450-S9.zip › FigureS2/CCNA_00302.pdf]

# CCNA\_00309

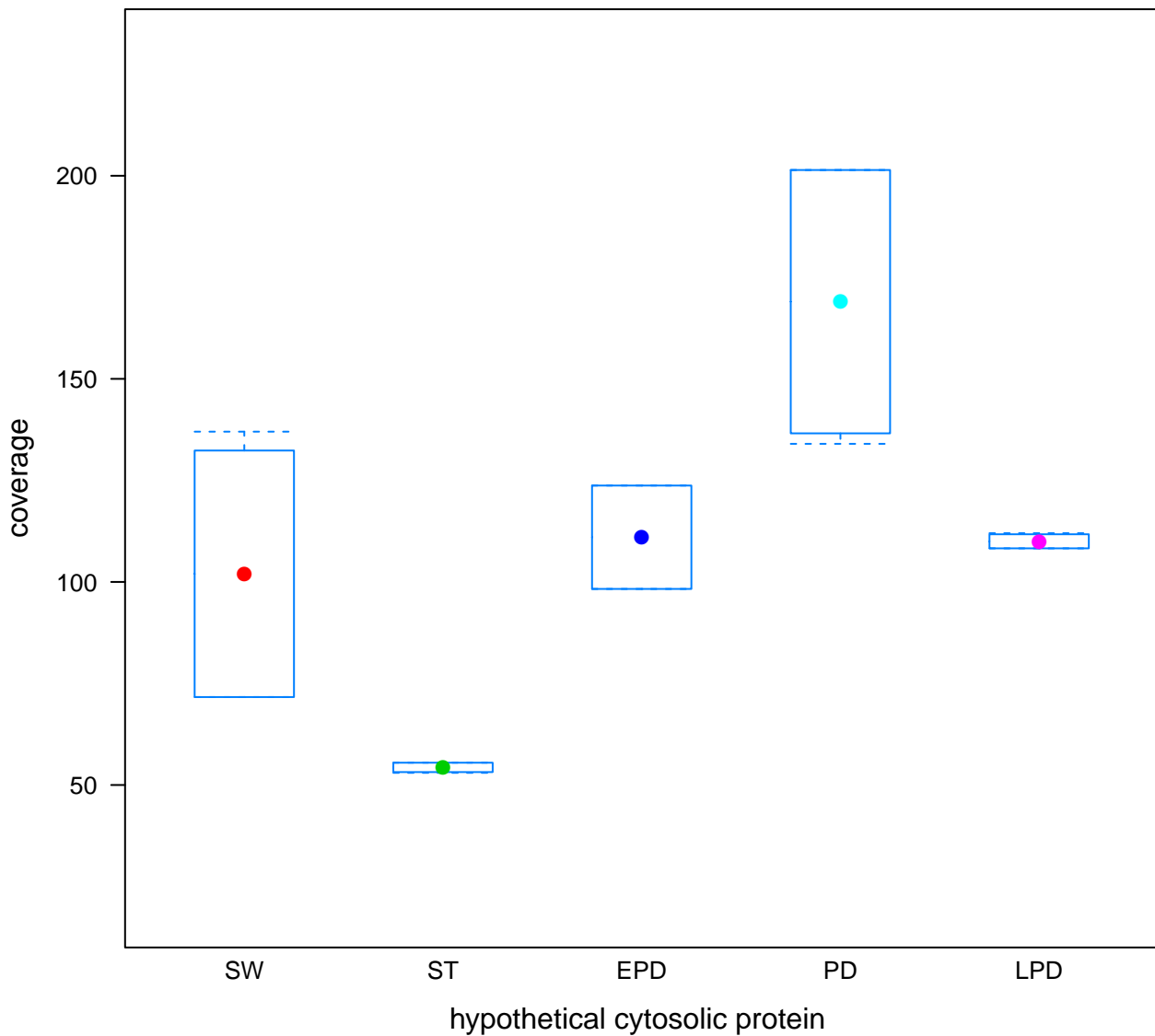

**Fold of change: 3.07**  
**baySeq likelihood: 0.935**

Supplement: Additional file 9: Figure S2 — Expression profiles of all identified CCR genes. [file 1471-2164-14-450-S9.zip › FigureS2/CCNA_00309.pdf]

# CCNA\_00315

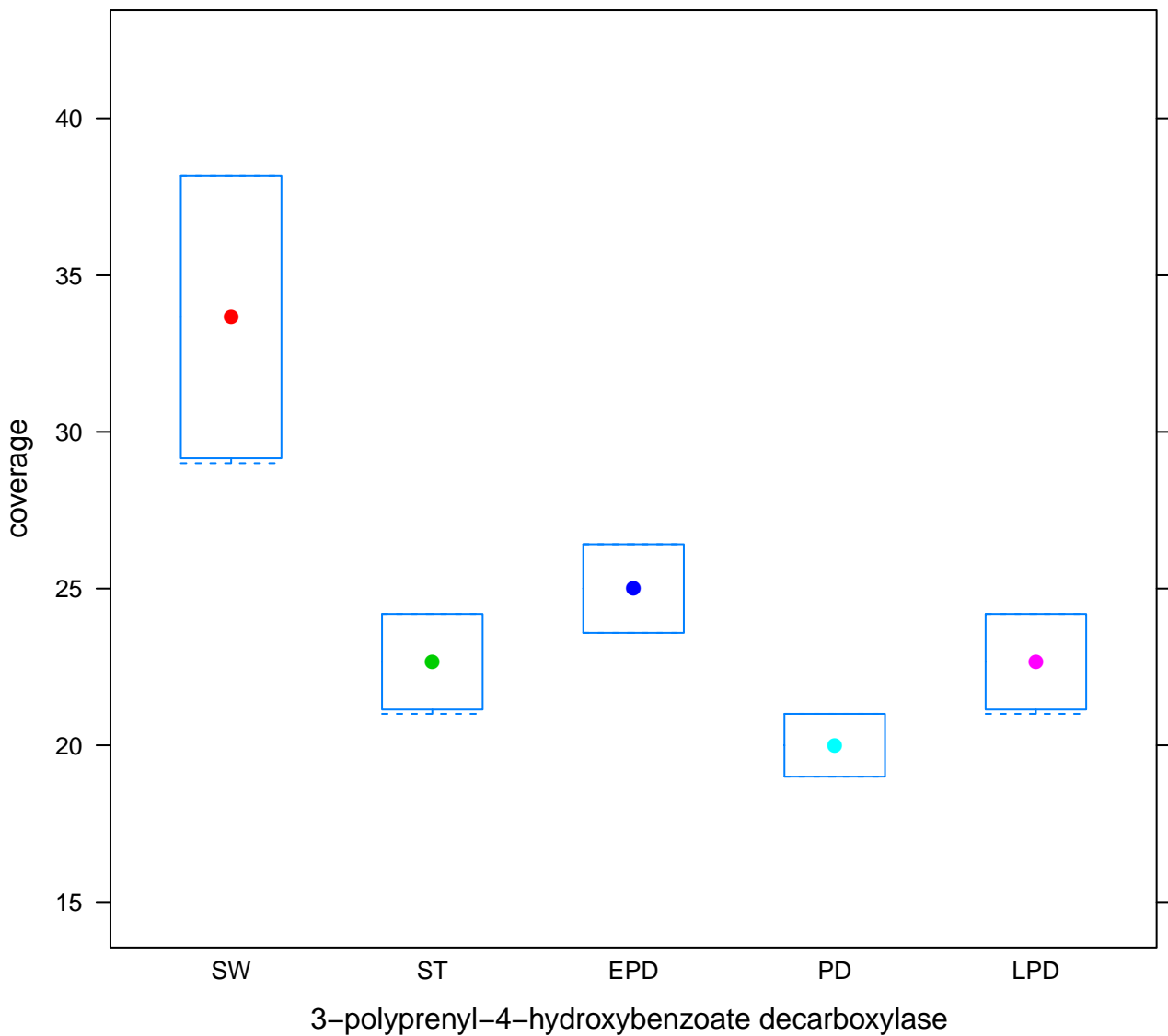

**Fold of change: 1.8**  
**baySeq likelihood: 0.897**

Supplement: Additional file 9: Figure S2 — Expression profiles of all identified CCR genes. [file 1471-2164-14-450-S9.zip › FigureS2/CCNA_00315.pdf]

# CCNA\_00316

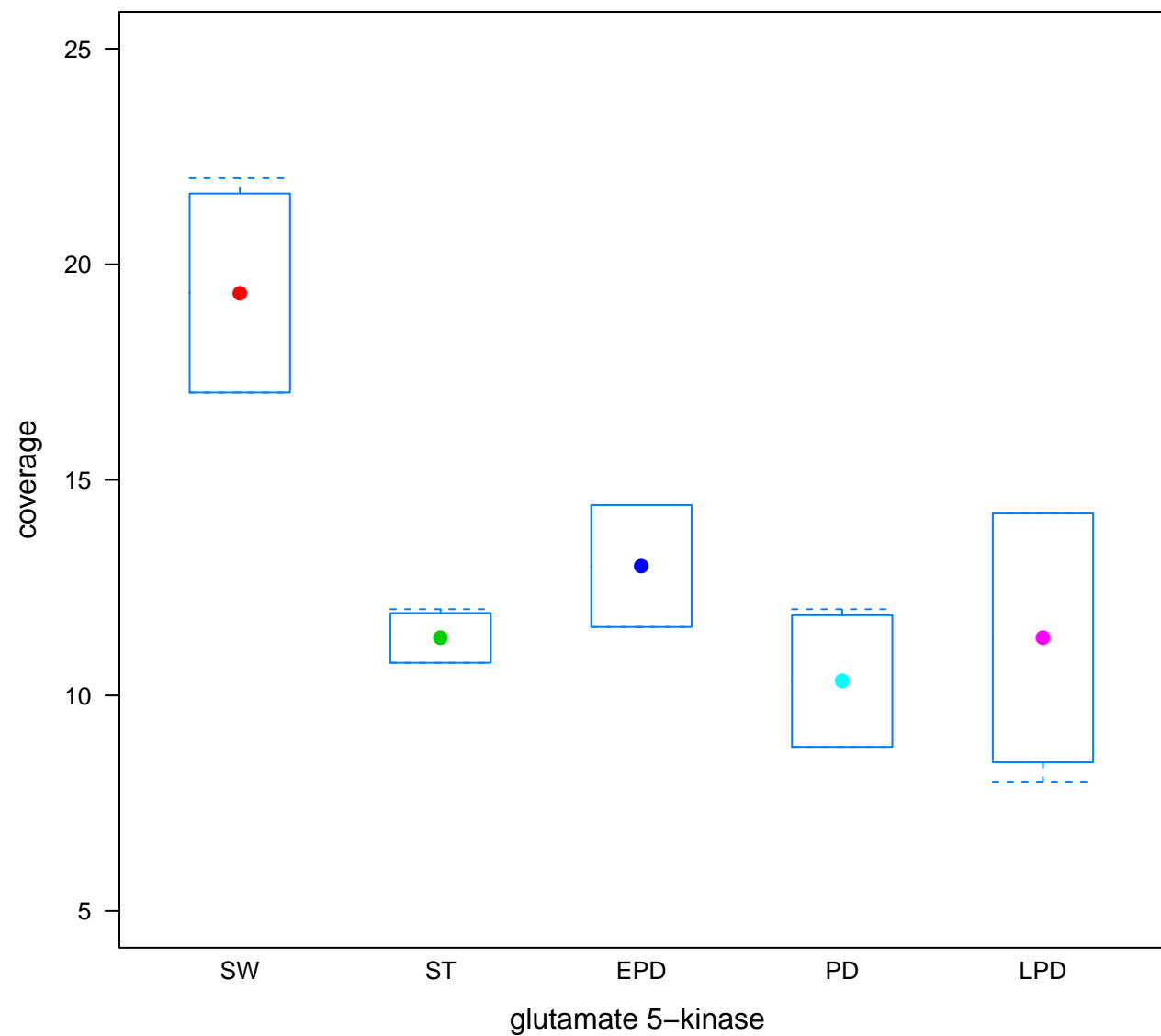

**Fold of change: 1.94**  
**baySeq likelihood: 0.879**

Supplement: Additional file 9: Figure S2 — Expression profiles of all identified CCR genes. [file 1471-2164-14-450-S9.zip › FigureS2/CCNA_00316.pdf]

# cgfA;CCNA\_00317

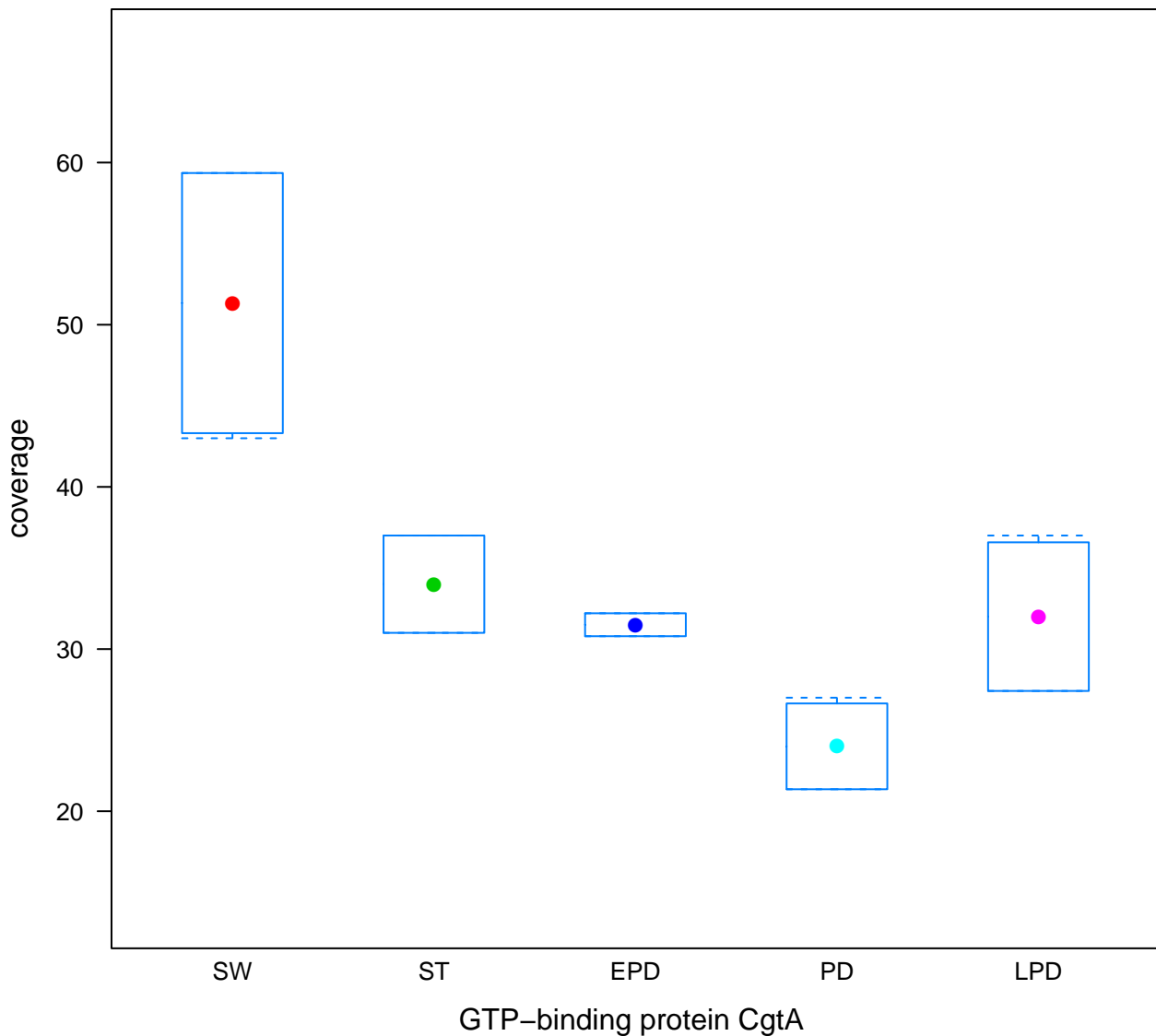

**Fold of change: 2.12**  
**baySeq likelihood: 0.976**

Supplement: Additional file 9: Figure S2 — Expression profiles of all identified CCR genes. [file 1471-2164-14-450-S9.zip › FigureS2/CCNA_00317.pdf]

# CCNA\_00318

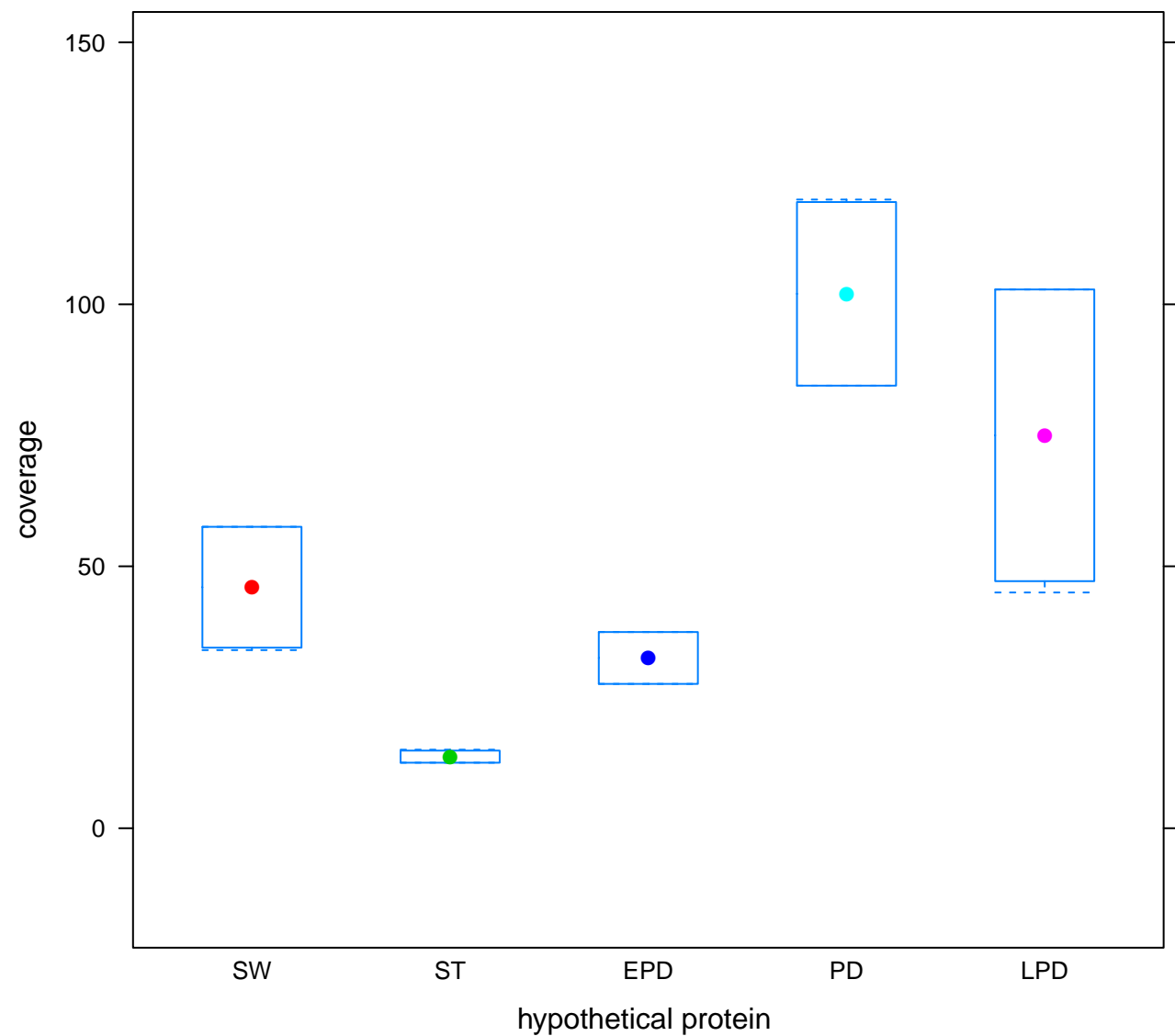

**Fold of change: 7.85**  
**baySeq likelihood: 0.911**

Supplement: Additional file 9: Figure S2 — Expression profiles of all identified CCR genes. [file 1471-2164-14-450-S9.zip › FigureS2/CCNA_00318.pdf]

# CCNA\_00321

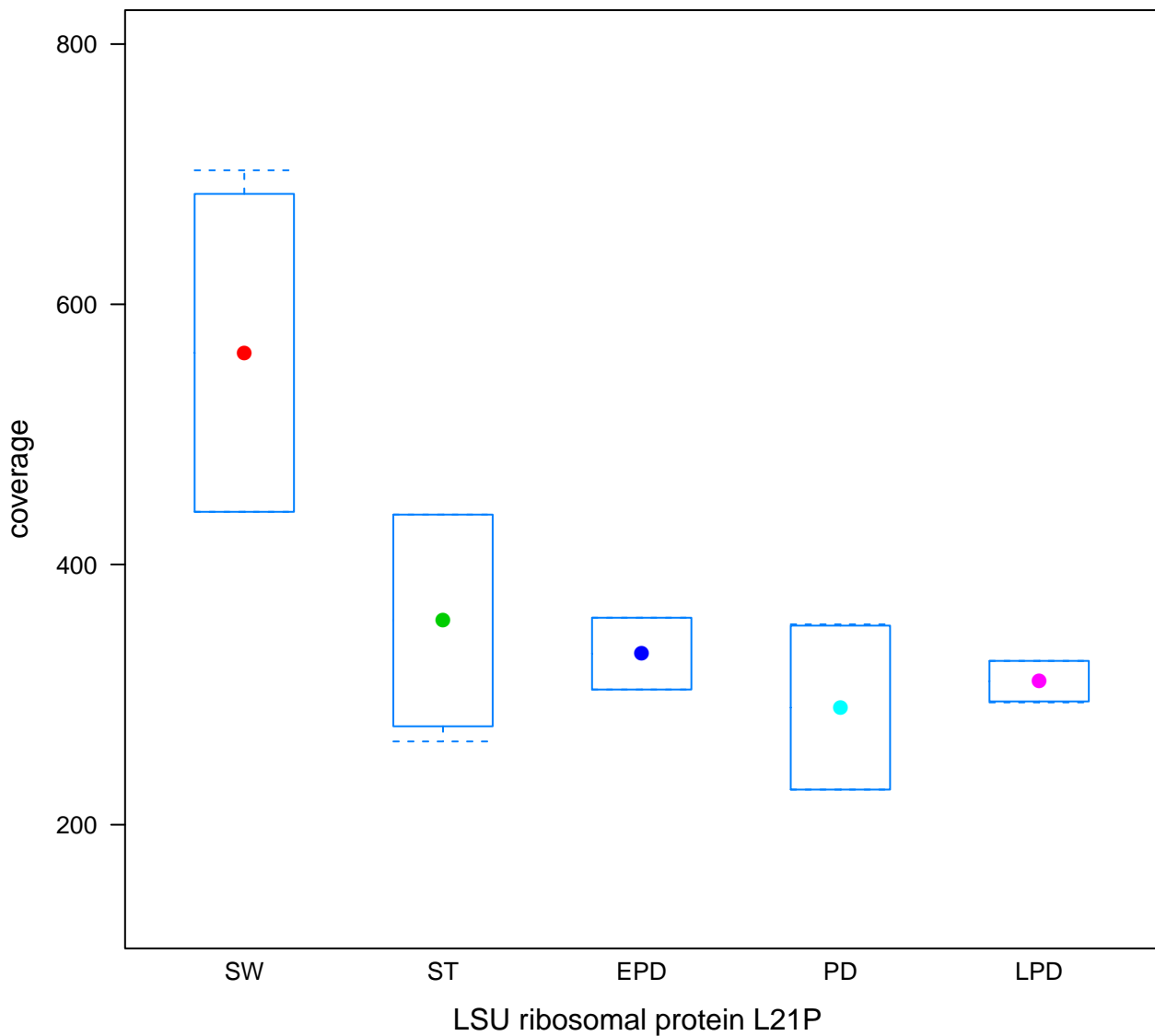

**Fold of change: 2.08**  
**baySeq likelihood: 0.479**

Supplement: Additional file 9: Figure S2 — Expression profiles of all identified CCR genes. [file 1471-2164-14-450-S9.zip › FigureS2/CCNA_00321.pdf]

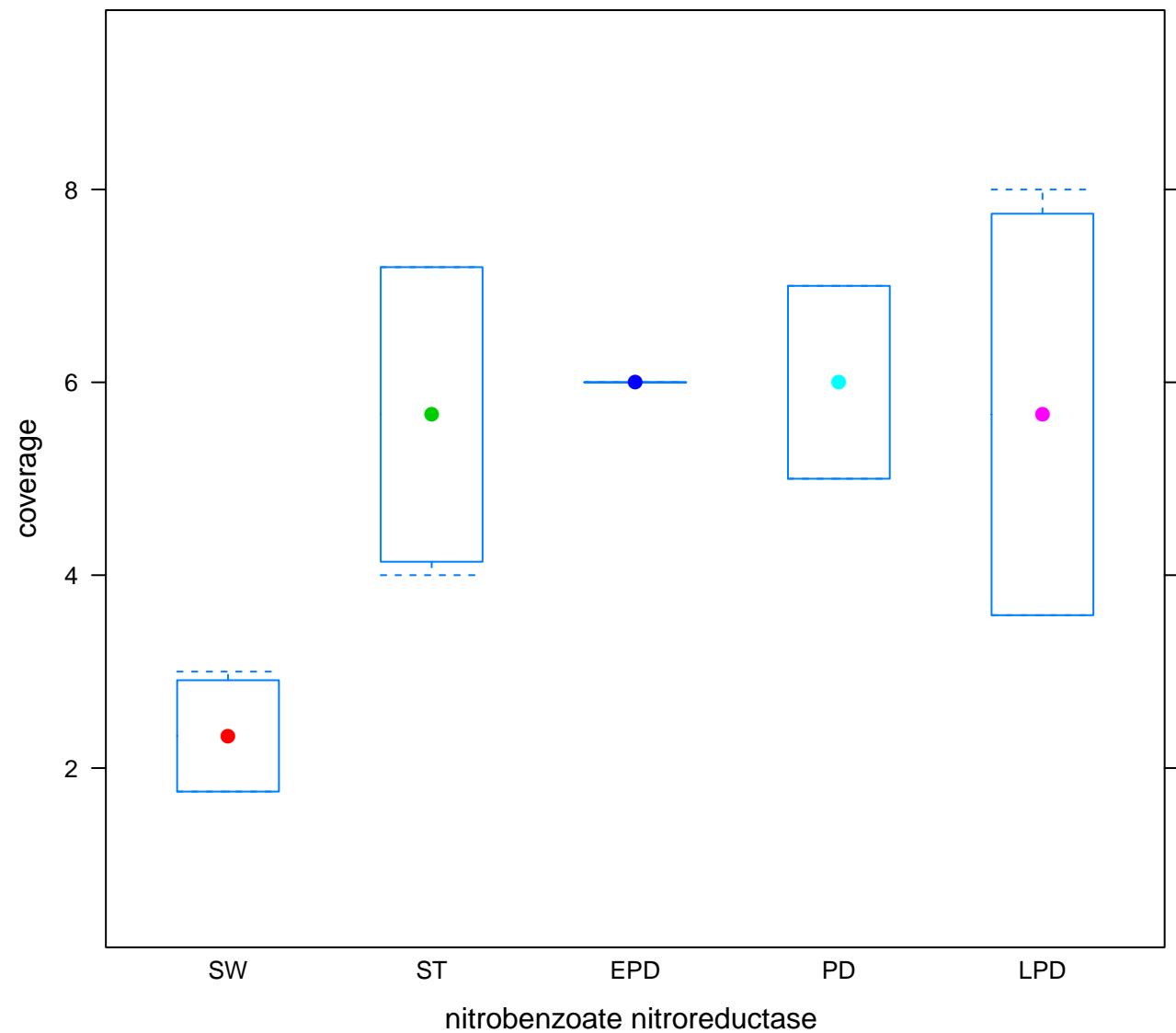

**Fold of change: 3**  
**baySeq likelihood: 0.386**

Supplement: Additional file 9: Figure S2 — Expression profiles of all identified CCR genes. [file 1471-2164-14-450-S9.zip › FigureS2/CCNA_00327.pdf]

# CCNA\_00328

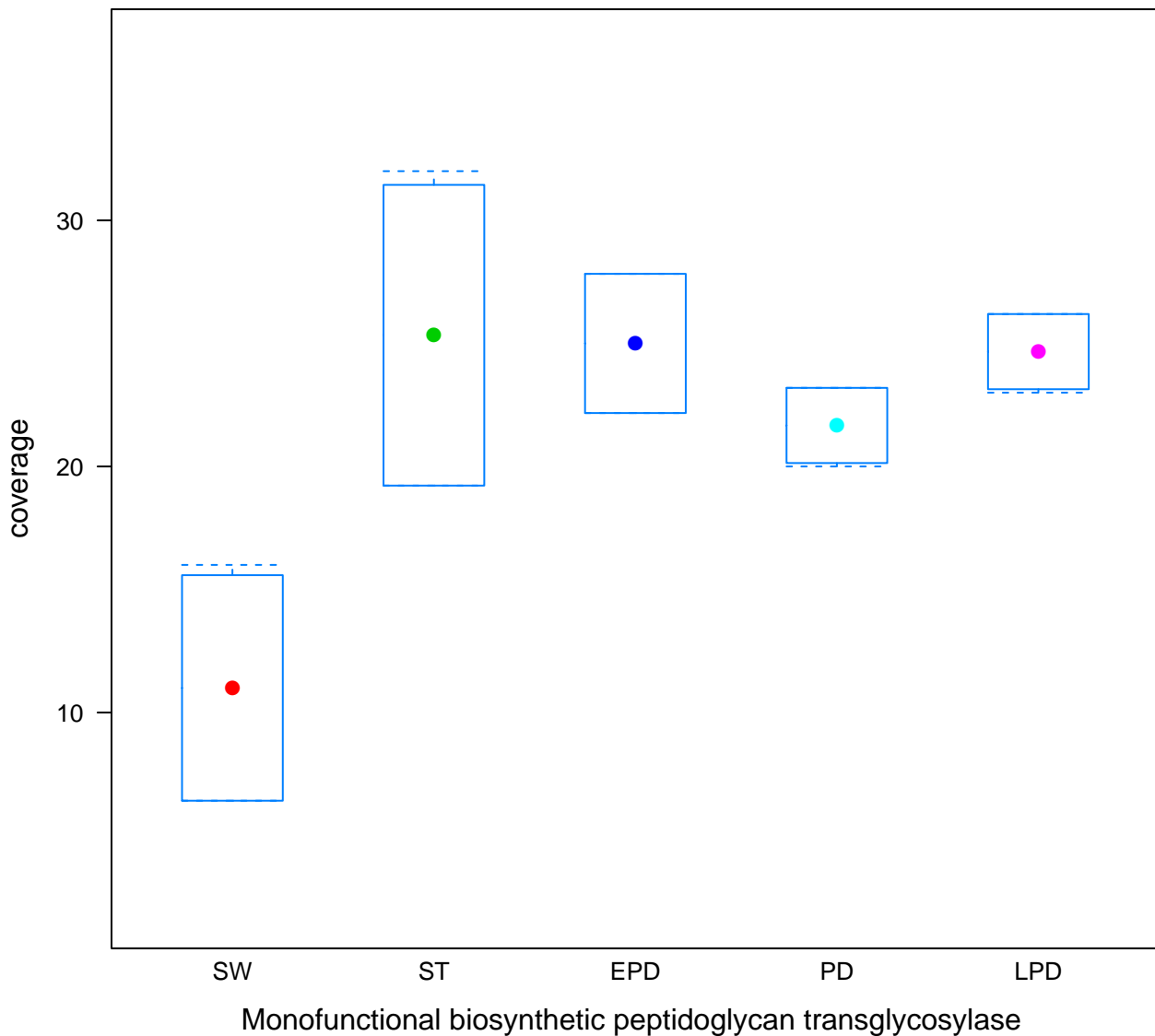

**Fold of change: 2.94**  
**baySeq likelihood: 0.991**

Supplement: Additional file 9: Figure S2 — Expression profiles of all identified CCR genes. [file 1471-2164-14-450-S9.zip › FigureS2/CCNA_00328.pdf]

# CCNA\_00329

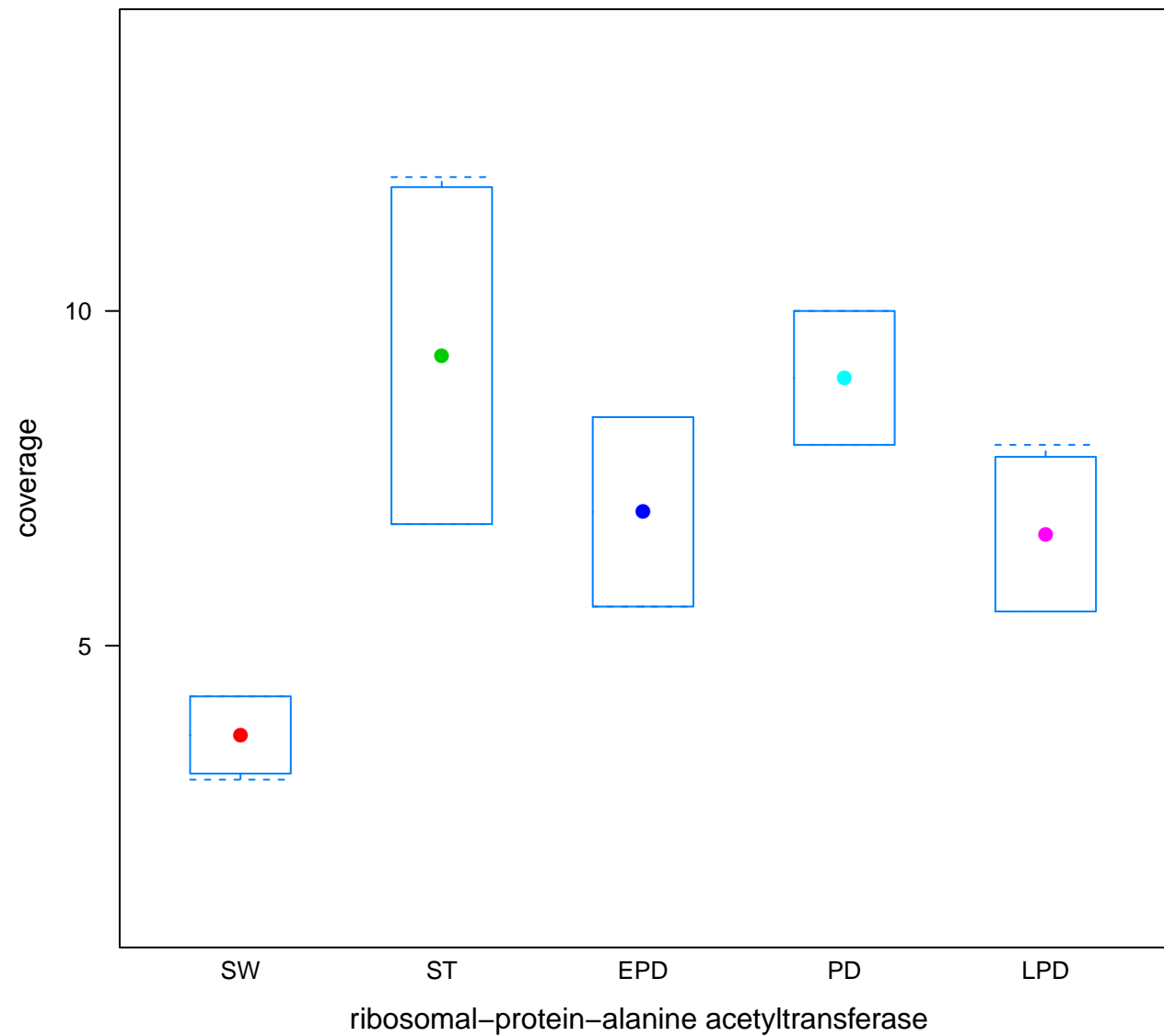

**Fold of change: 2.57**  
**baySeq likelihood: 0.45**

Supplement: Additional file 9: Figure S2 — Expression profiles of all identified CCR genes. [file 1471-2164-14-450-S9.zip › FigureS2/CCNA_00329.pdf]

# CCNA\_00331

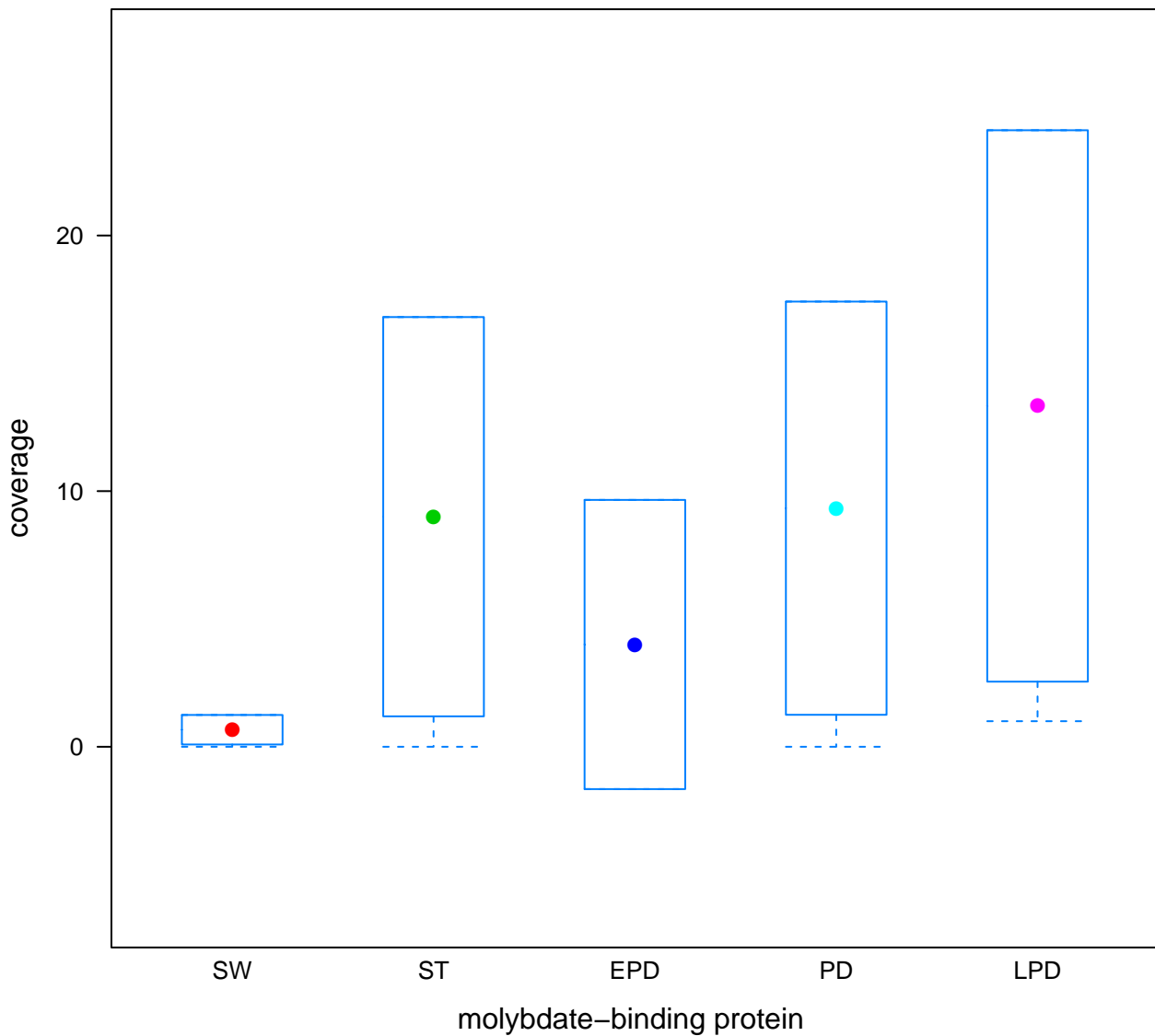

**Fold of change: 26.67**  
**baySeq likelihood: 0.995**

Supplement: Additional file 9: Figure S2 — Expression profiles of all identified CCR genes. [file 1471-2164-14-450-S9.zip › FigureS2/CCNA_00331.pdf]

# CCNA\_00332

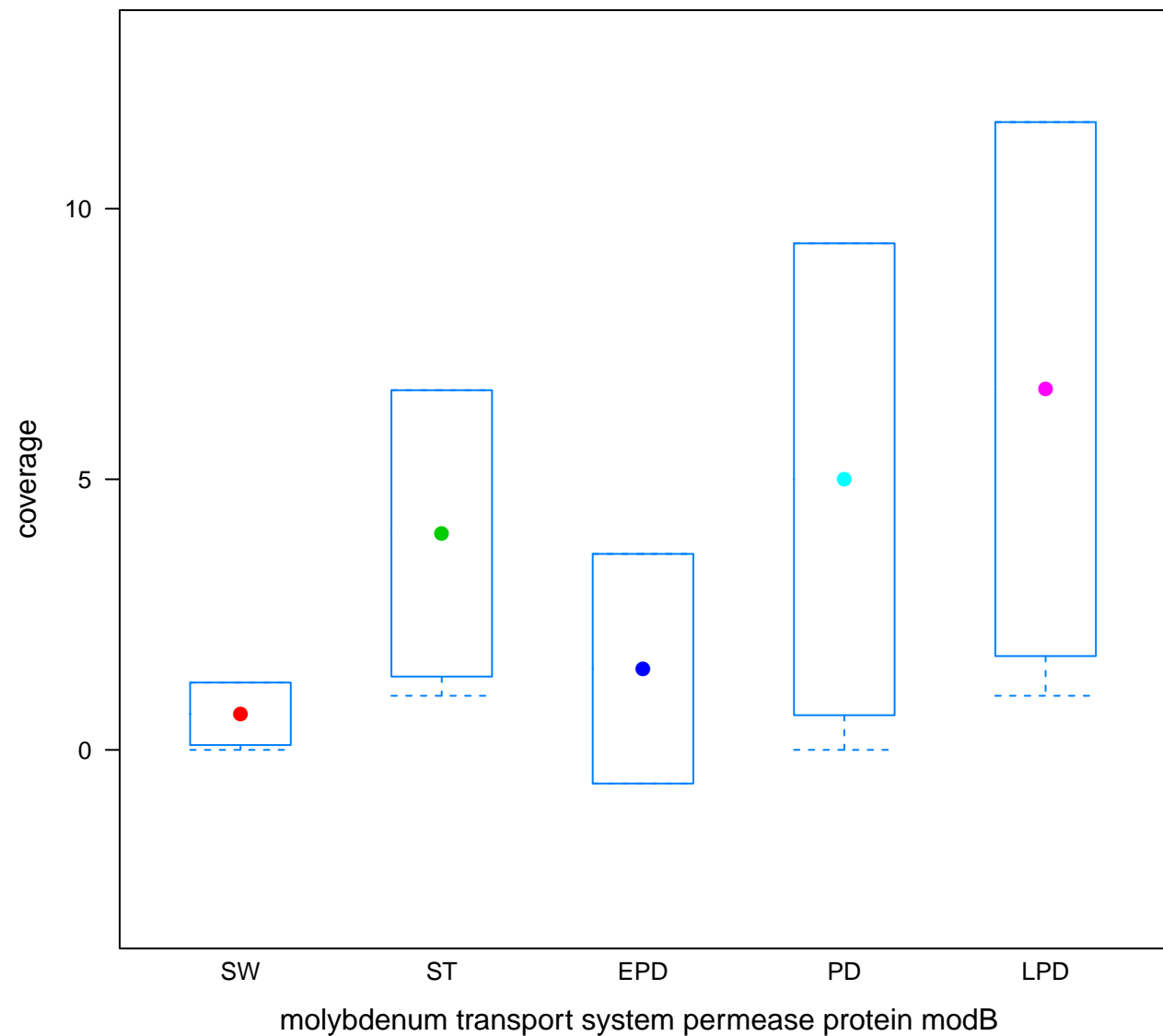

**Fold of change: 13.33**  
**baySeq likelihood: 0.54**

Supplement: Additional file 9: Figure S2 — Expression profiles of all identified CCR genes. [file 1471-2164-14-450-S9.zip › FigureS2/CCNA_00332.pdf]

# CCNA\_00338

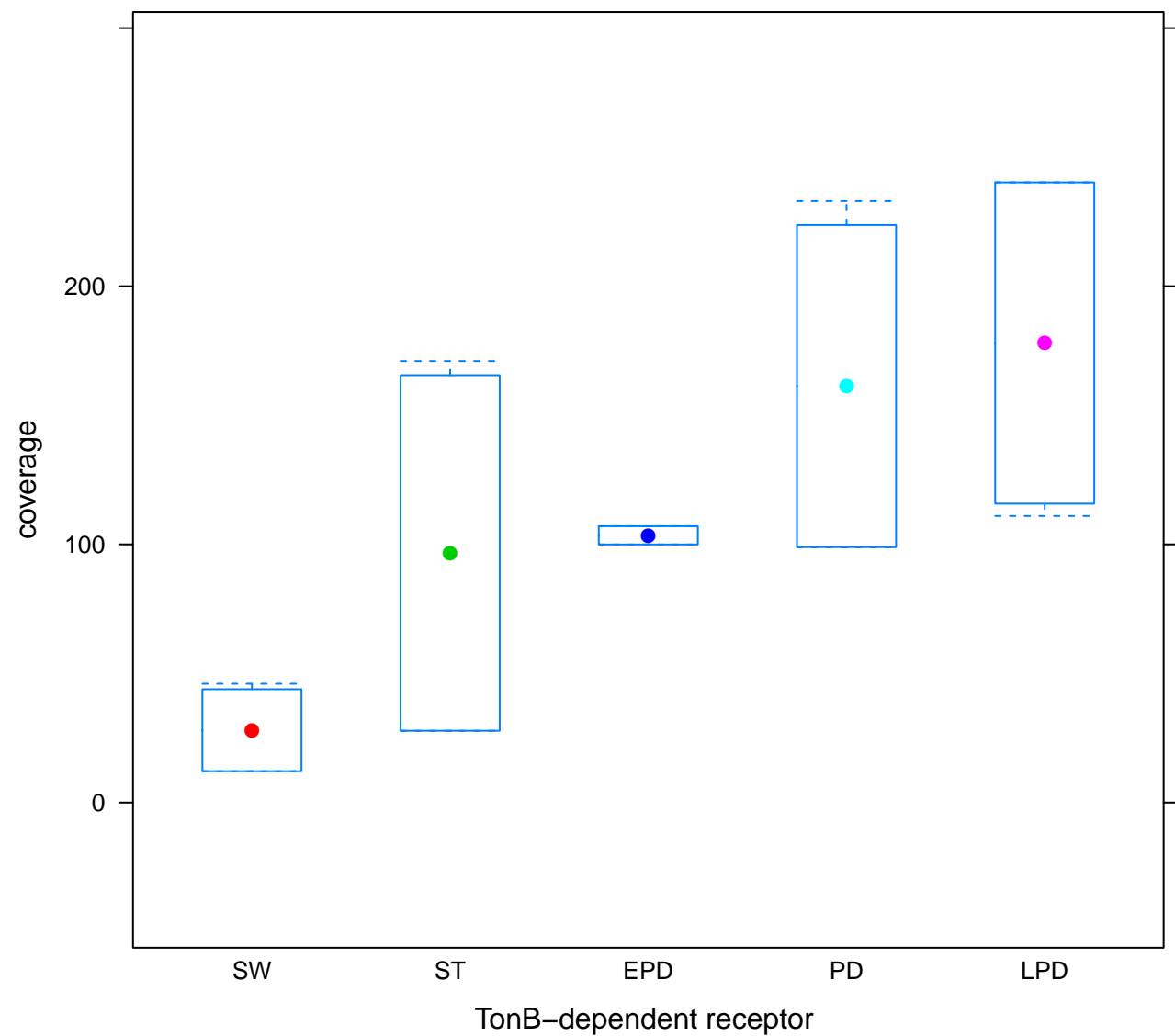

**Fold of change: 9.37**  
**baySeq likelihood: 0.93**

Supplement: Additional file 9: Figure S2 — Expression profiles of all identified CCR genes. [file 1471-2164-14-450-S9.zip › FigureS2/CCNA_00338.pdf]

# CCNA\_00339

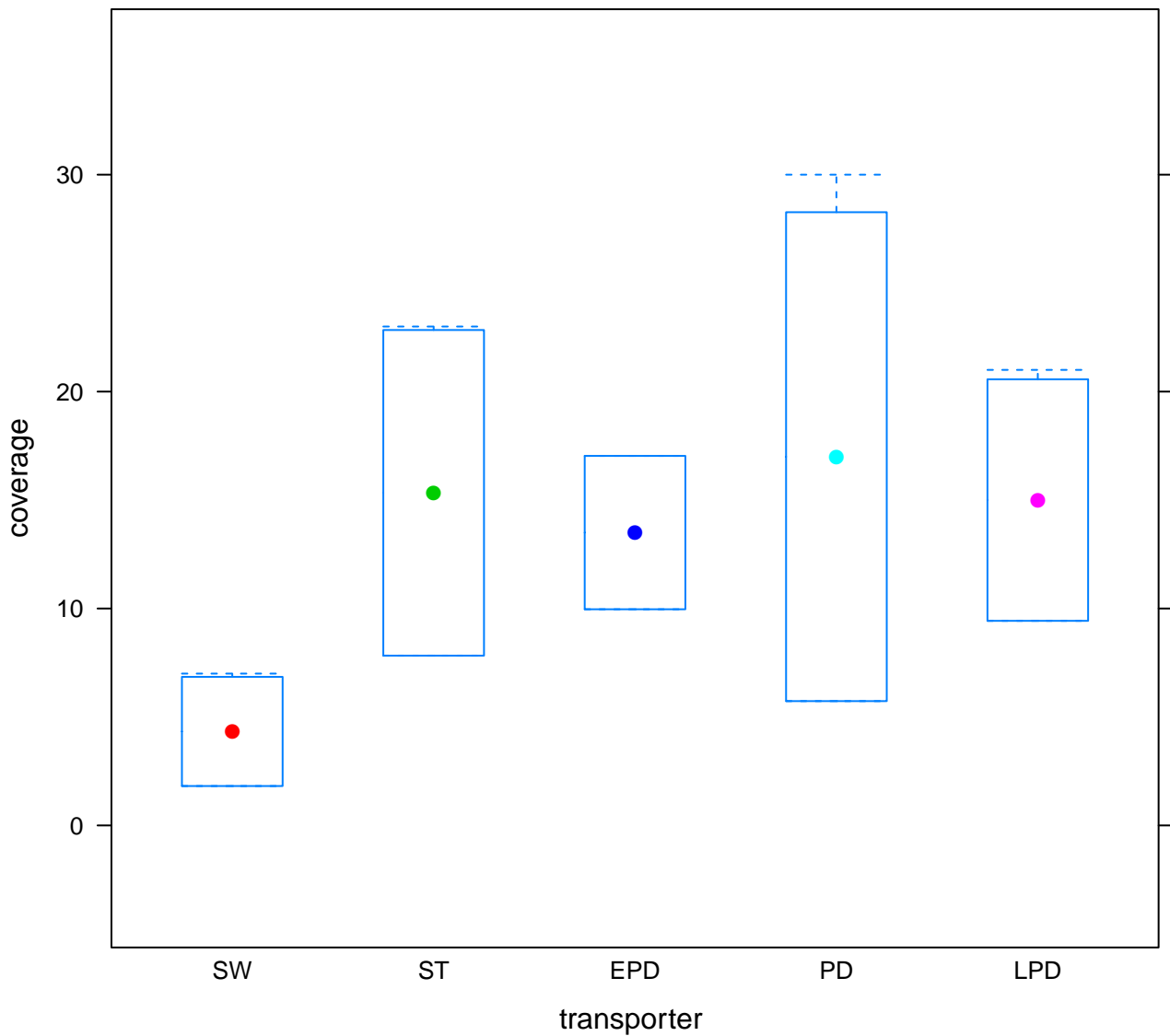

**Fold of change: 5.67**  
**baySeq likelihood: 0.937**

Supplement: Additional file 9: Figure S2 — Expression profiles of all identified CCR genes. [file 1471-2164-14-450-S9.zip › FigureS2/CCNA_00339.pdf]

# CCNA\_00345

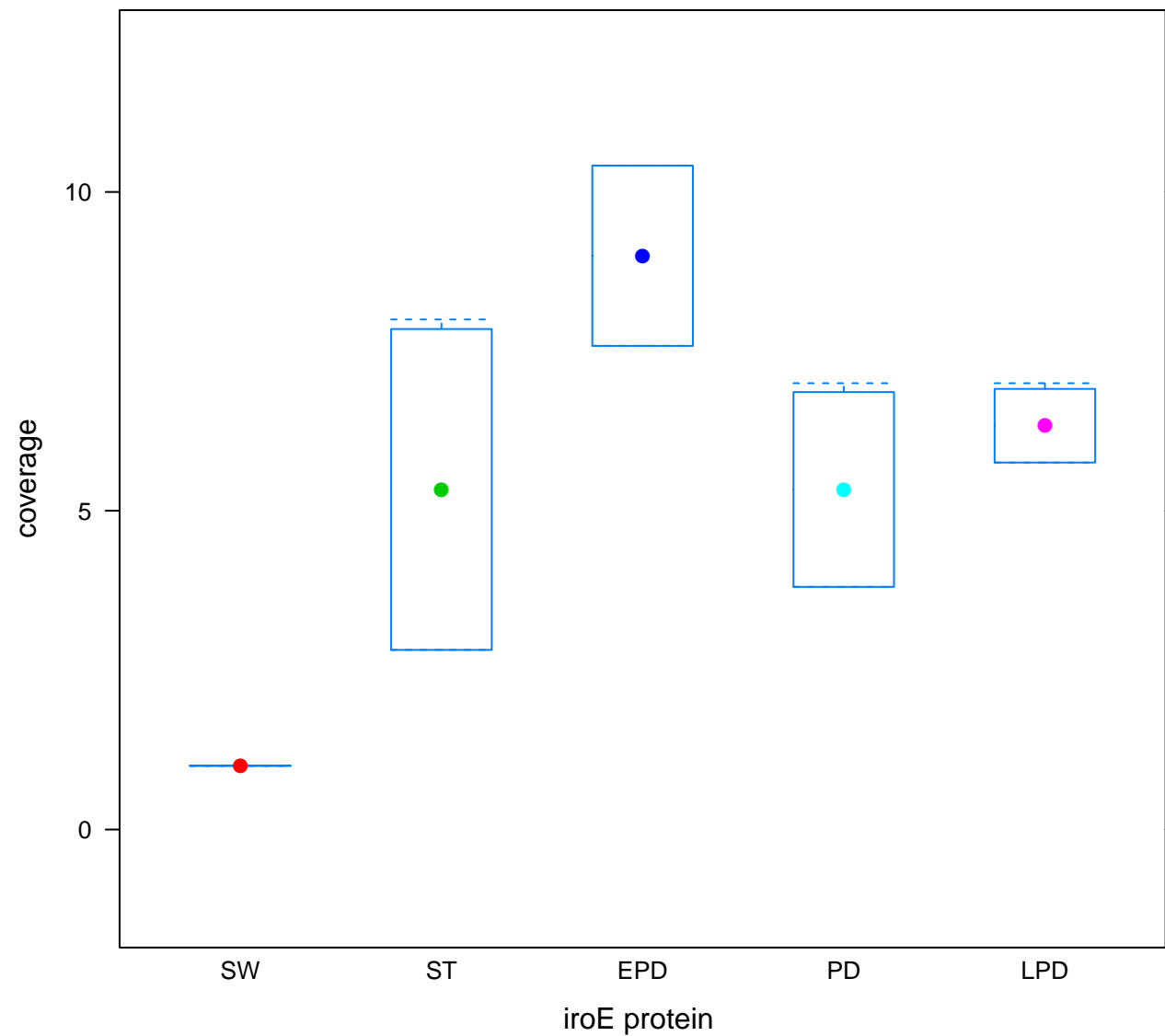

**Fold of change: 9**  
**baySeq likelihood: 0.983**

Supplement: Additional file 9: Figure S2 — Expression profiles of all identified CCR genes. [file 1471-2164-14-450-S9.zip › FigureS2/CCNA_00345.pdf]

# CCNA\_00348

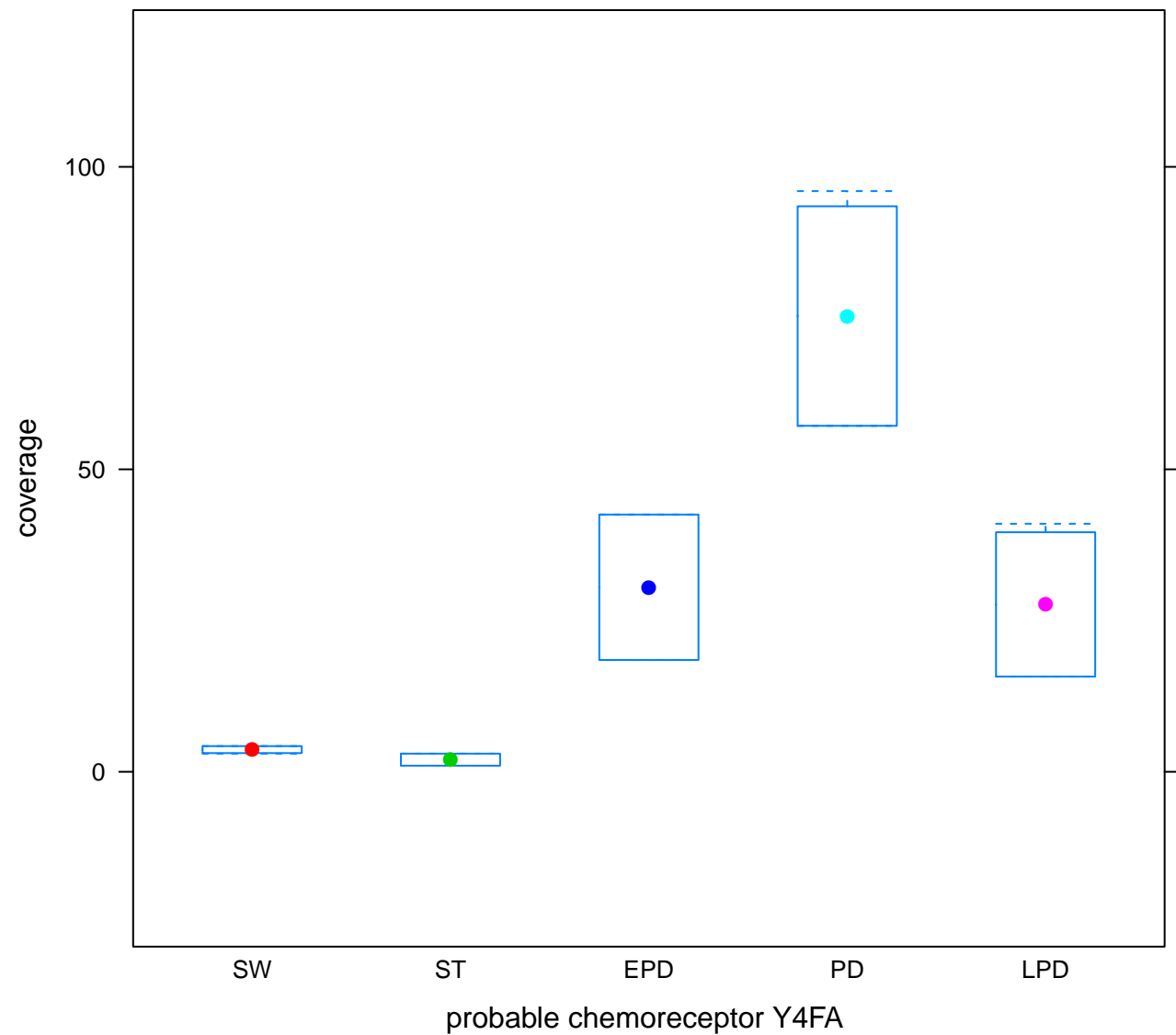

**Fold of change: 50.22**

**baySeq likelihood: 1**

Supplement: Additional file 9: Figure S2 — Expression profiles of all identified CCR genes. [file 1471-2164-14-450-S9.zip › FigureS2/CCNA_00348.pdf]

# CCNA\_00349

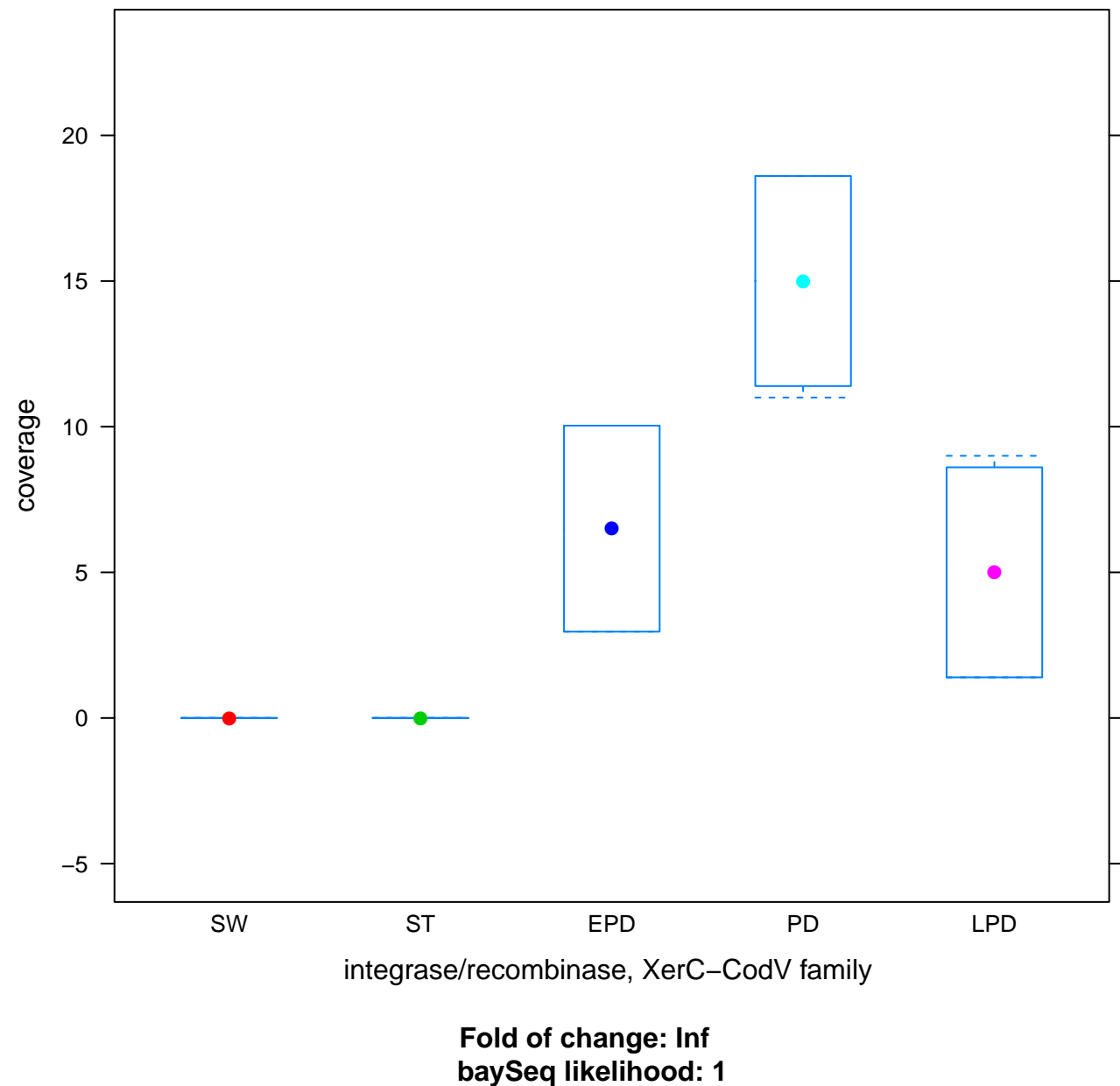

Supplement: Additional file 9: Figure S2 — Expression profiles of all identified CCR genes. [file 1471-2164-14-450-S9.zip › FigureS2/CCNA_00349.pdf]

# CCNA\_00350

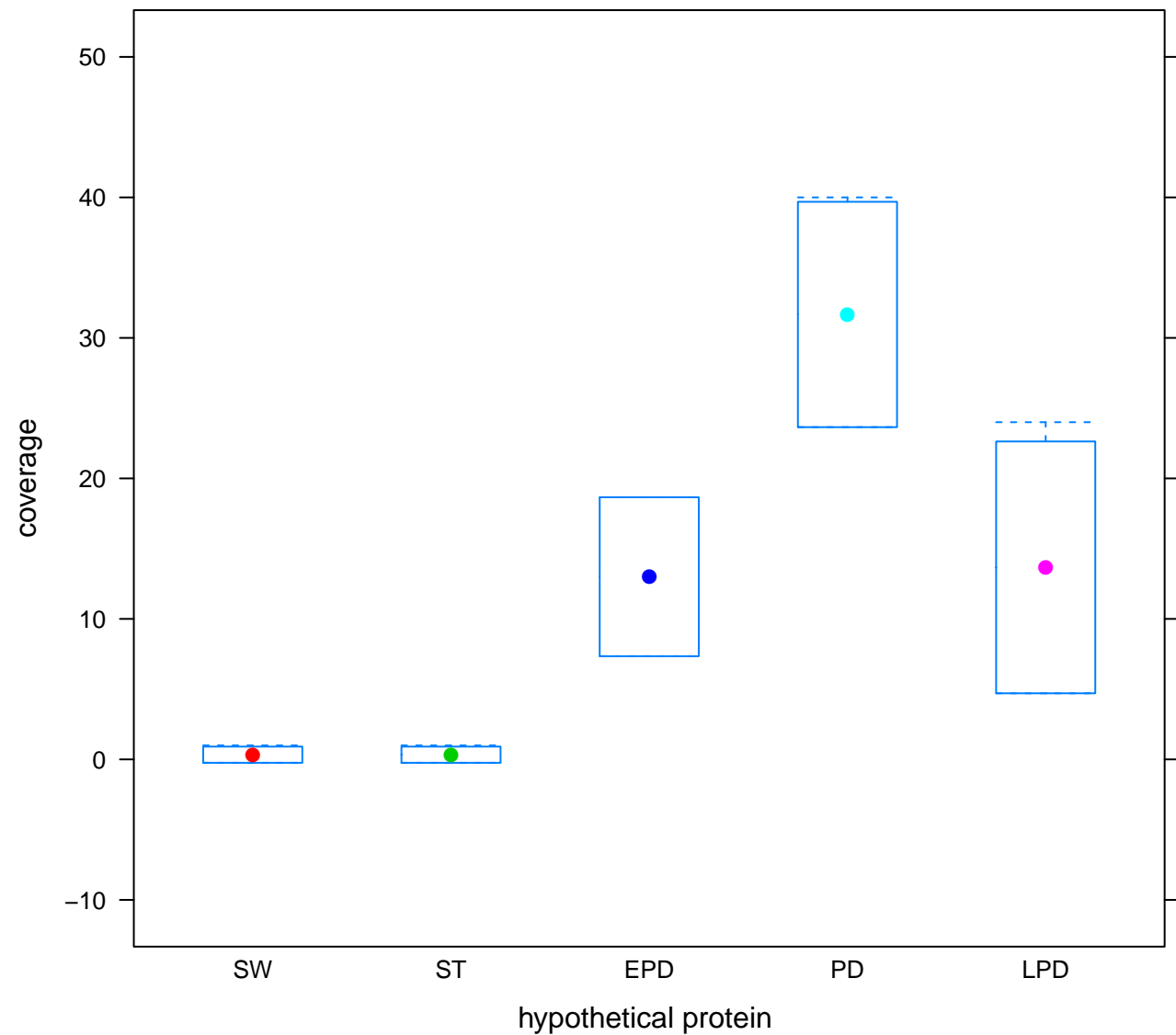

**Fold of change: Inf**  
**baySeq likelihood: 1**

Supplement: Additional file 9: Figure S2 — Expression profiles of all identified CCR genes. [file 1471-2164-14-450-S9.zip › FigureS2/CCNA_00350.pdf]

# CCNA\_00352

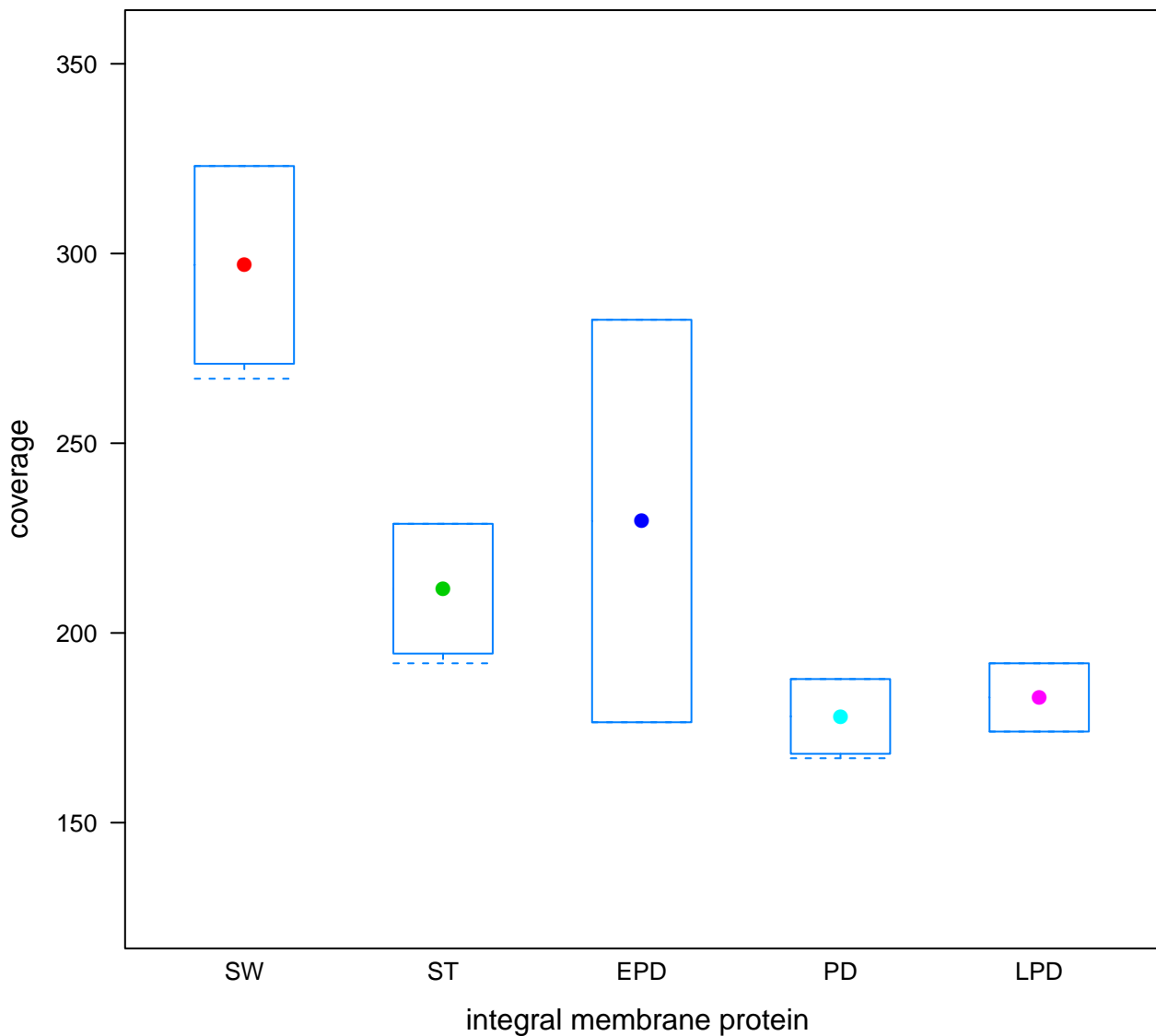

**Fold of change: 1.63**  
**baySeq likelihood: 0.803**

Supplement: Additional file 9: Figure S2 — Expression profiles of all identified CCR genes. [file 1471-2164-14-450-S9.zip › FigureS2/CCNA_00352.pdf]

# CCNA\_00353

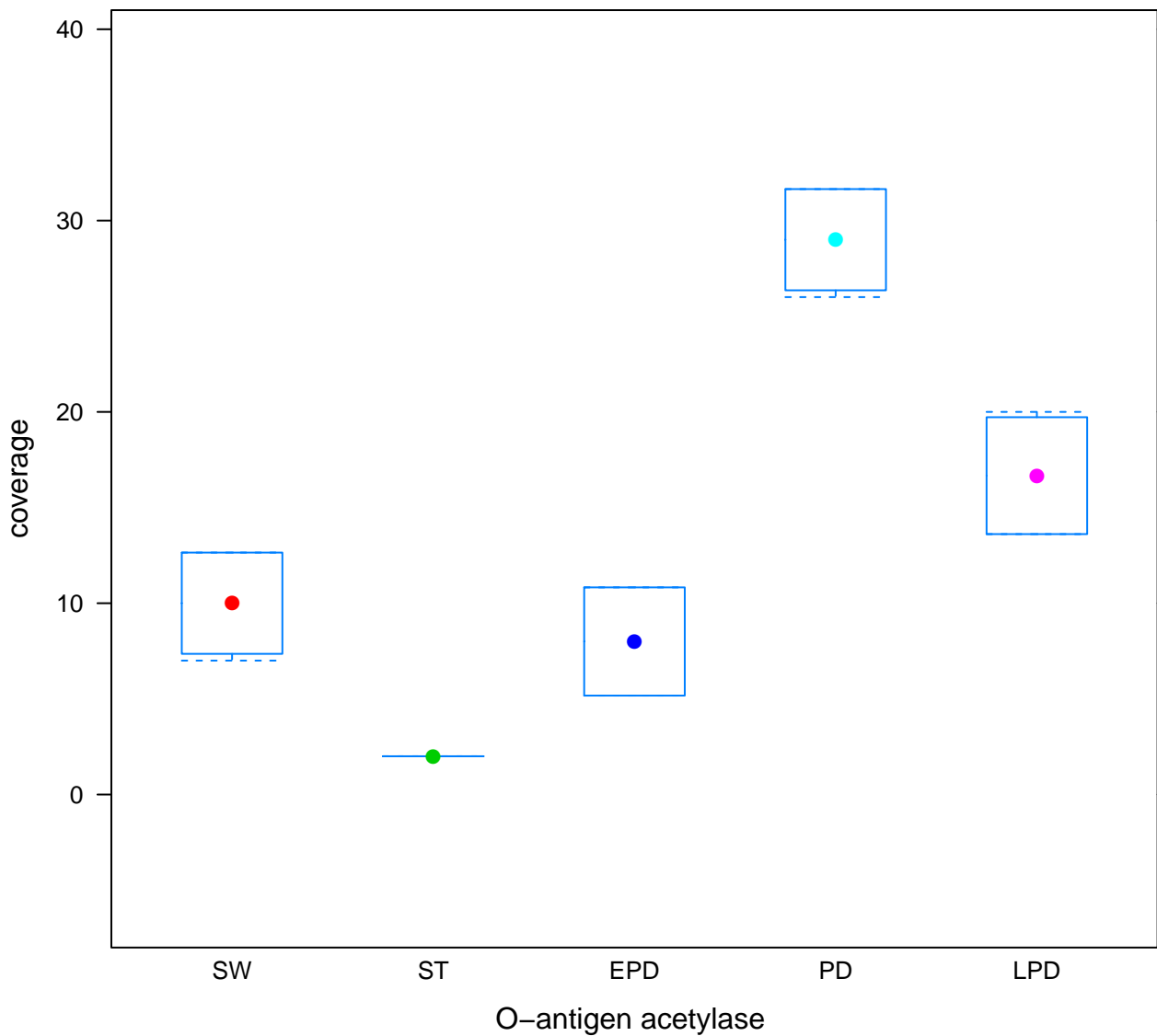

**Fold of change: 14.5**  
**baySeq likelihood: 0.578**

Supplement: Additional file 9: Figure S2 — Expression profiles of all identified CCR genes. [file 1471-2164-14-450-S9.zip › FigureS2/CCNA_00353.pdf]

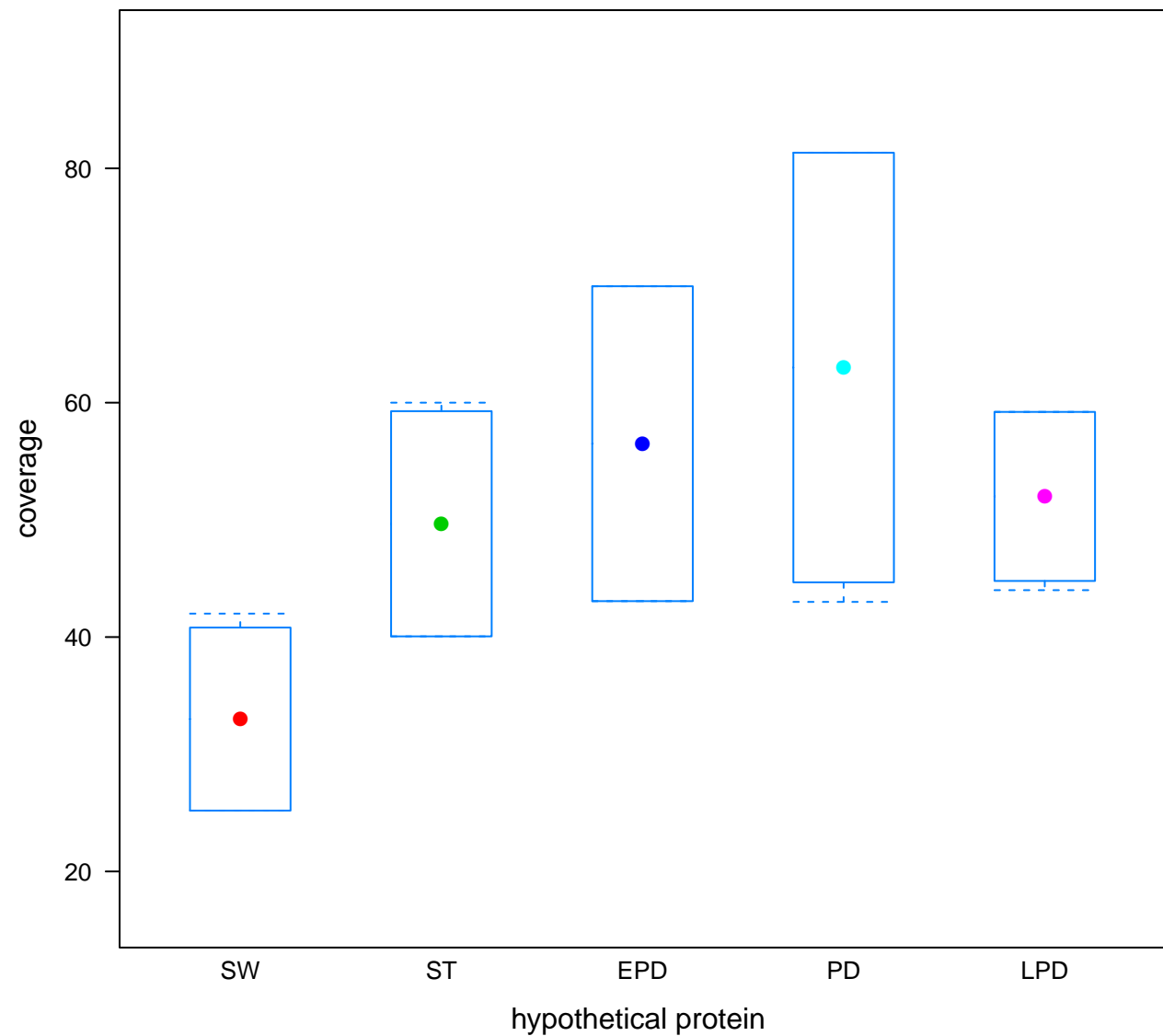

**Fold of change: 1.8**  
**baySeq likelihood: 0.549**

Supplement: Additional file 9: Figure S2 — Expression profiles of all identified CCR genes. [file 1471-2164-14-450-S9.zip › FigureS2/CCNA_00354.pdf]

# CCNA\_00355

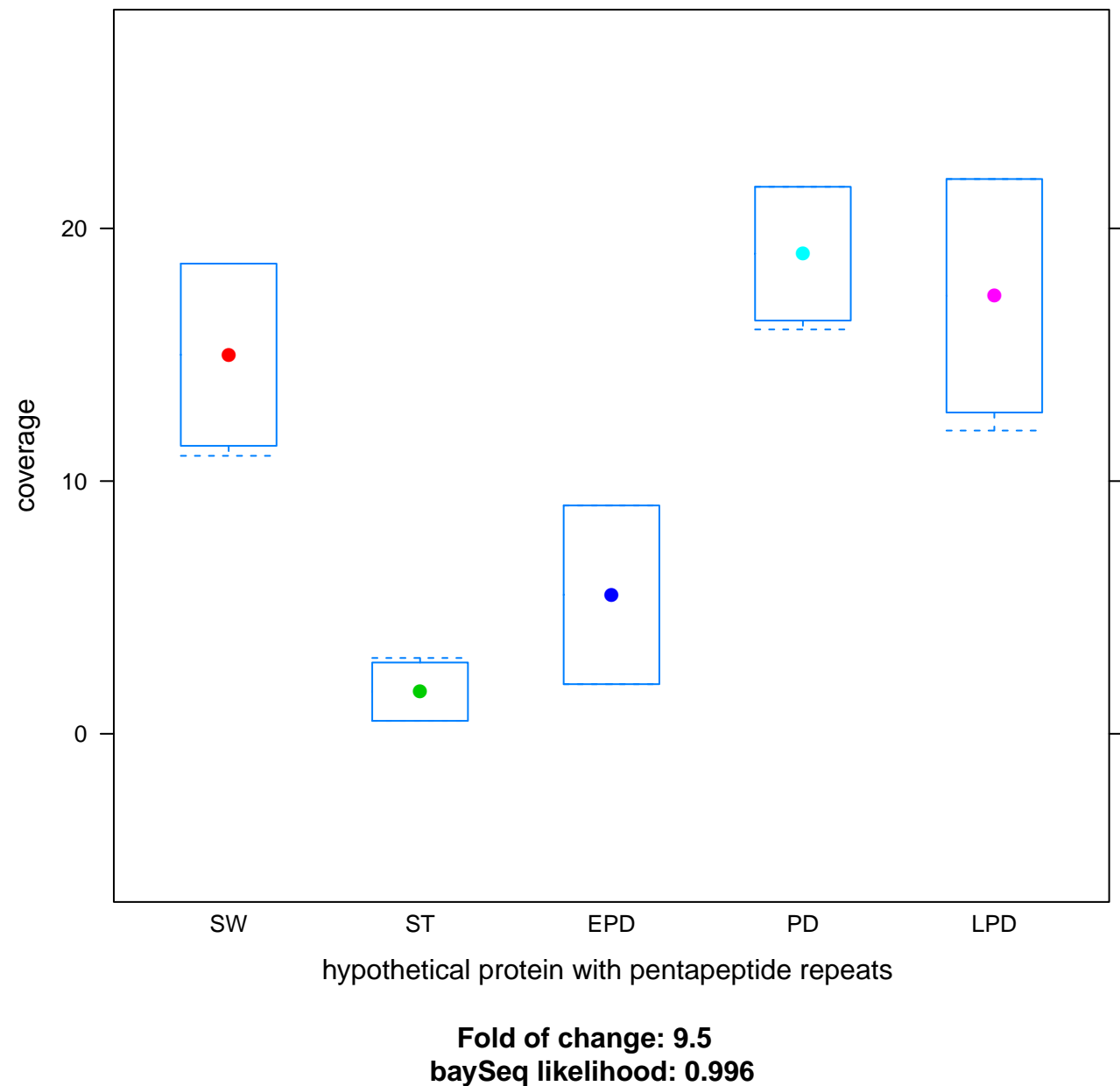

Supplement: Additional file 9: Figure S2 — Expression profiles of all identified CCR genes. [file 1471-2164-14-450-S9.zip › FigureS2/CCNA_00355.pdf]

# CCNA\_00357

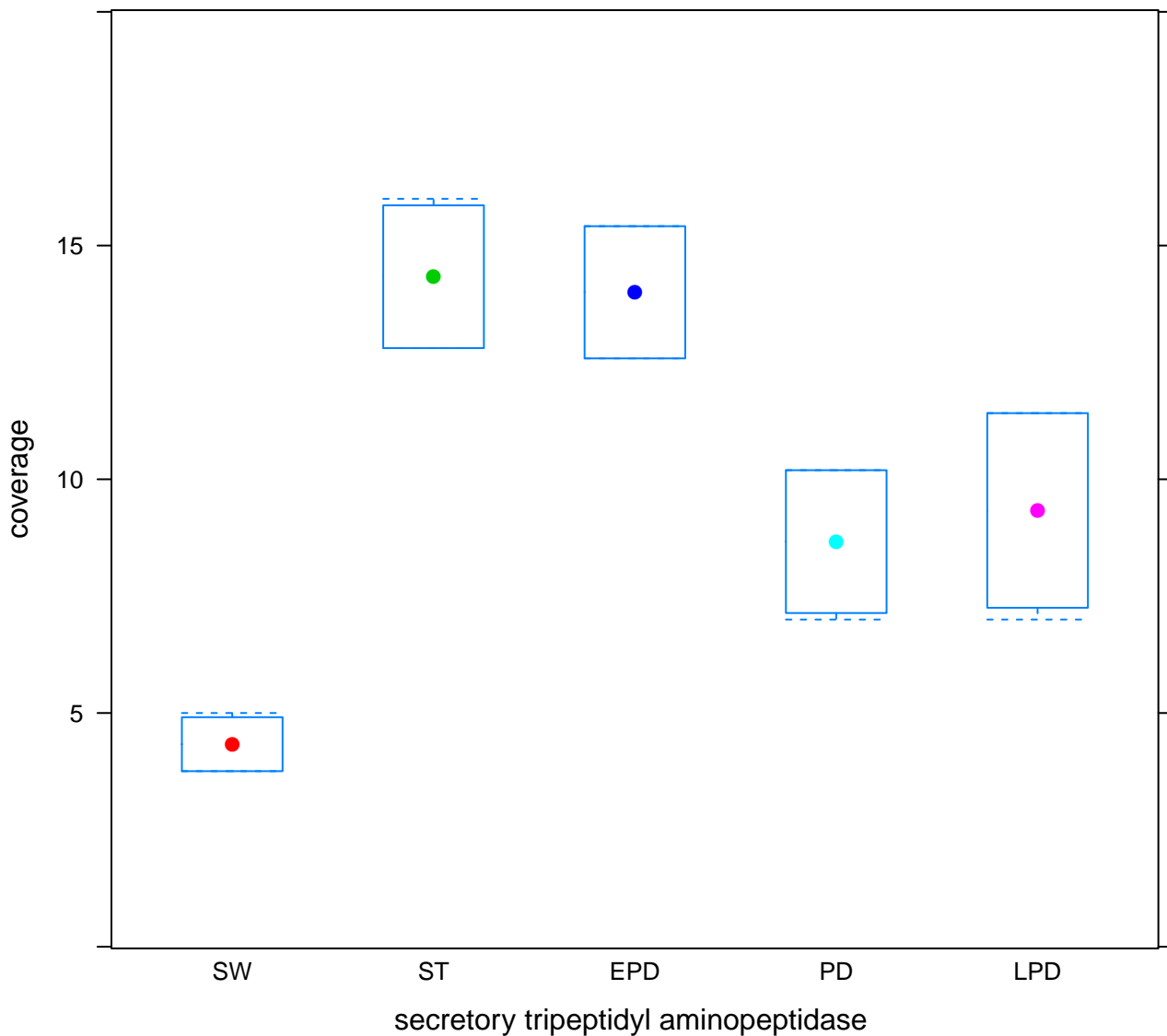

**Fold of change: 3.5**  
**baySeq likelihood: 0.683**

Supplement: Additional file 9: Figure S2 — Expression profiles of all identified CCR genes. [file 1471-2164-14-450-S9.zip › FigureS2/CCNA_00357.pdf]

# CCNA\_00361

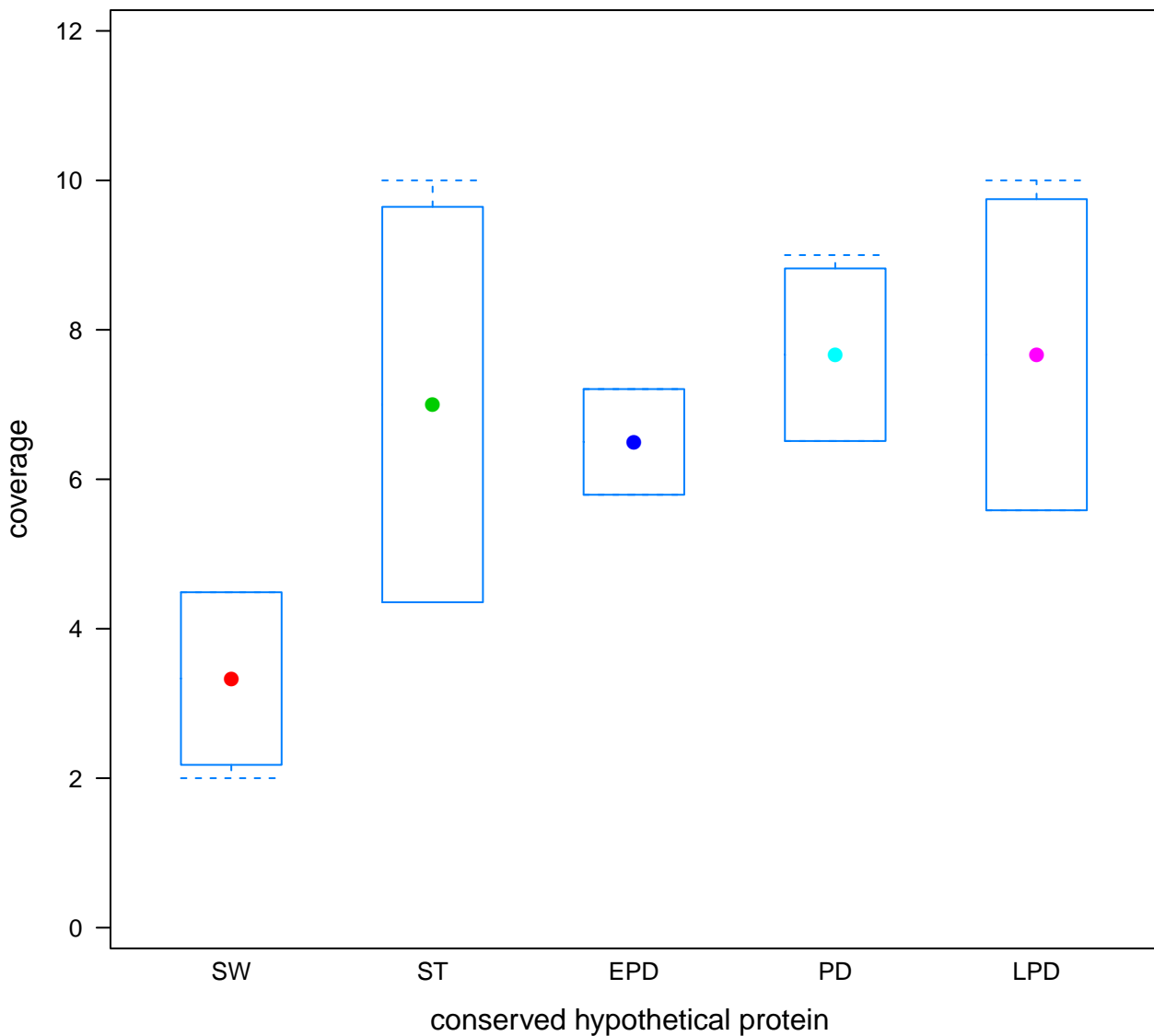

**Fold of change: 2.56**  
**baySeq likelihood: 0.361**

Supplement: Additional file 9: Figure S2 — Expression profiles of all identified CCR genes. [file 1471-2164-14-450-S9.zip › FigureS2/CCNA_00361.pdf]

# CCNA\_00365

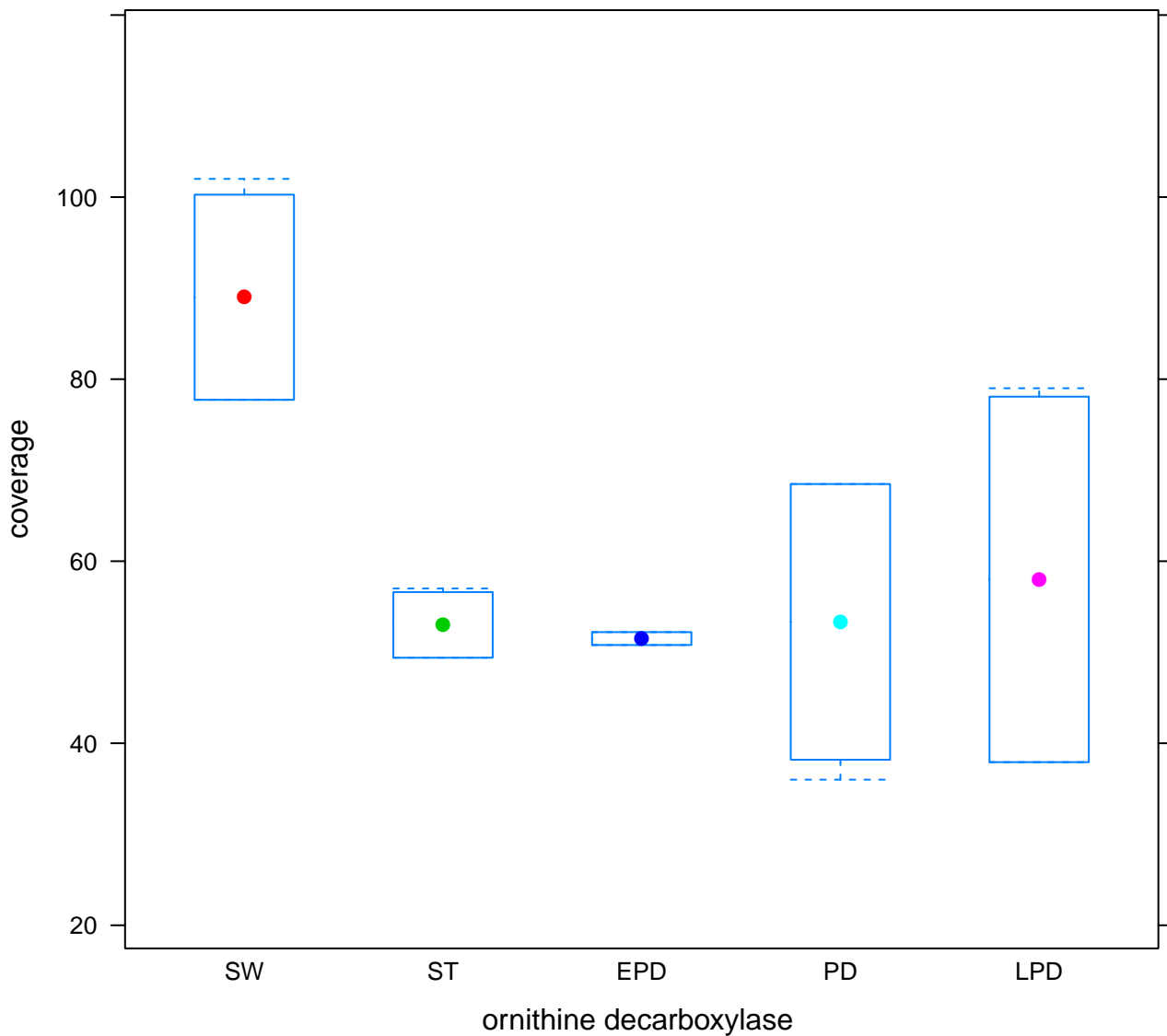

**Fold of change: 1.79**  
**baySeq likelihood: 0.909**

Supplement: Additional file 9: Figure S2 — Expression profiles of all identified CCR genes. [file 1471-2164-14-450-S9.zip › FigureS2/CCNA_00365.pdf]

# CCNA\_00366

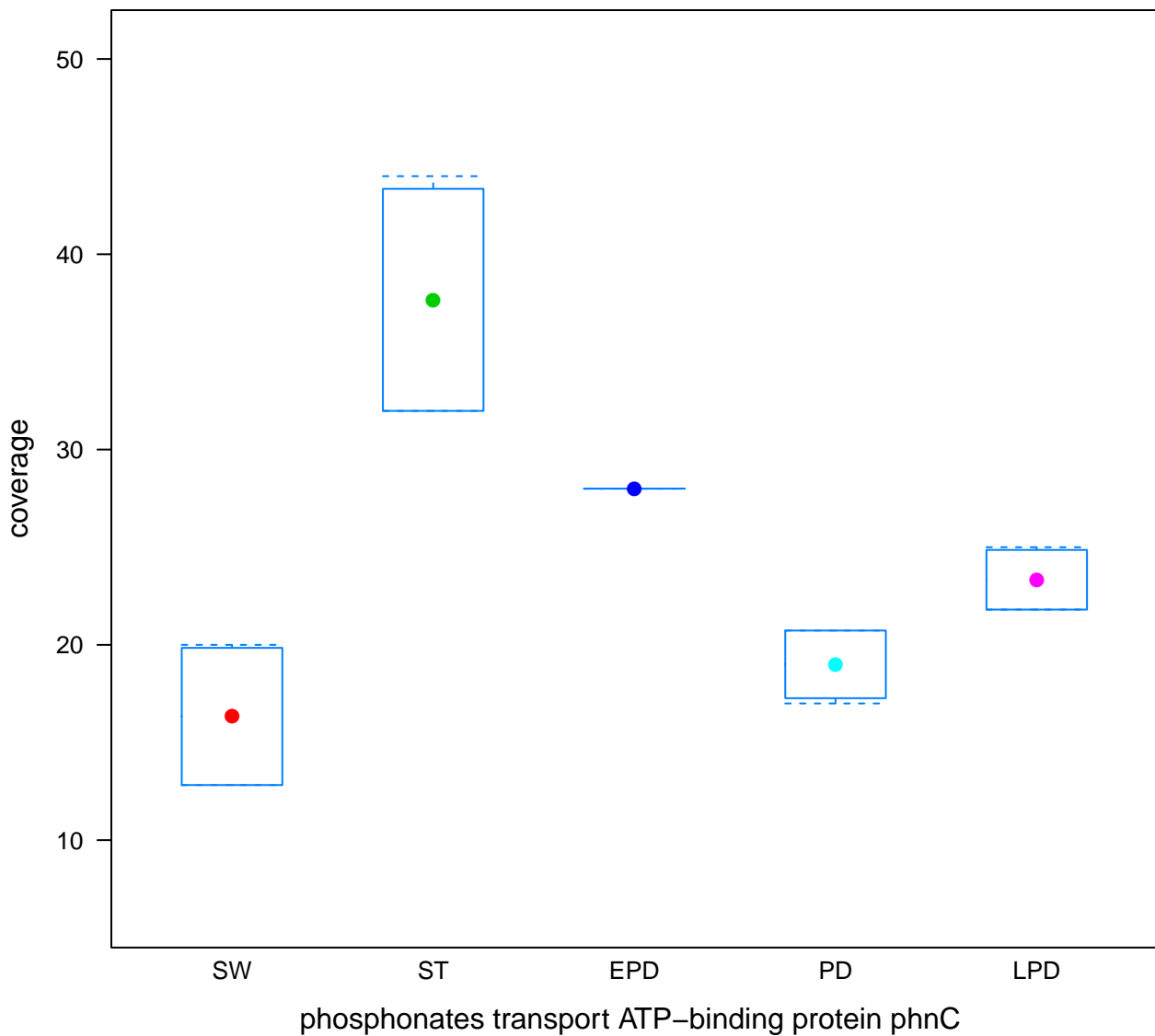

**Fold of change: 1.92**  
**baySeq likelihood: 0.739**

Supplement: Additional file 9: Figure S2 — Expression profiles of all identified CCR genes. [file 1471-2164-14-450-S9.zip › FigureS2/CCNA_00366.pdf]

# CCNA\_00367

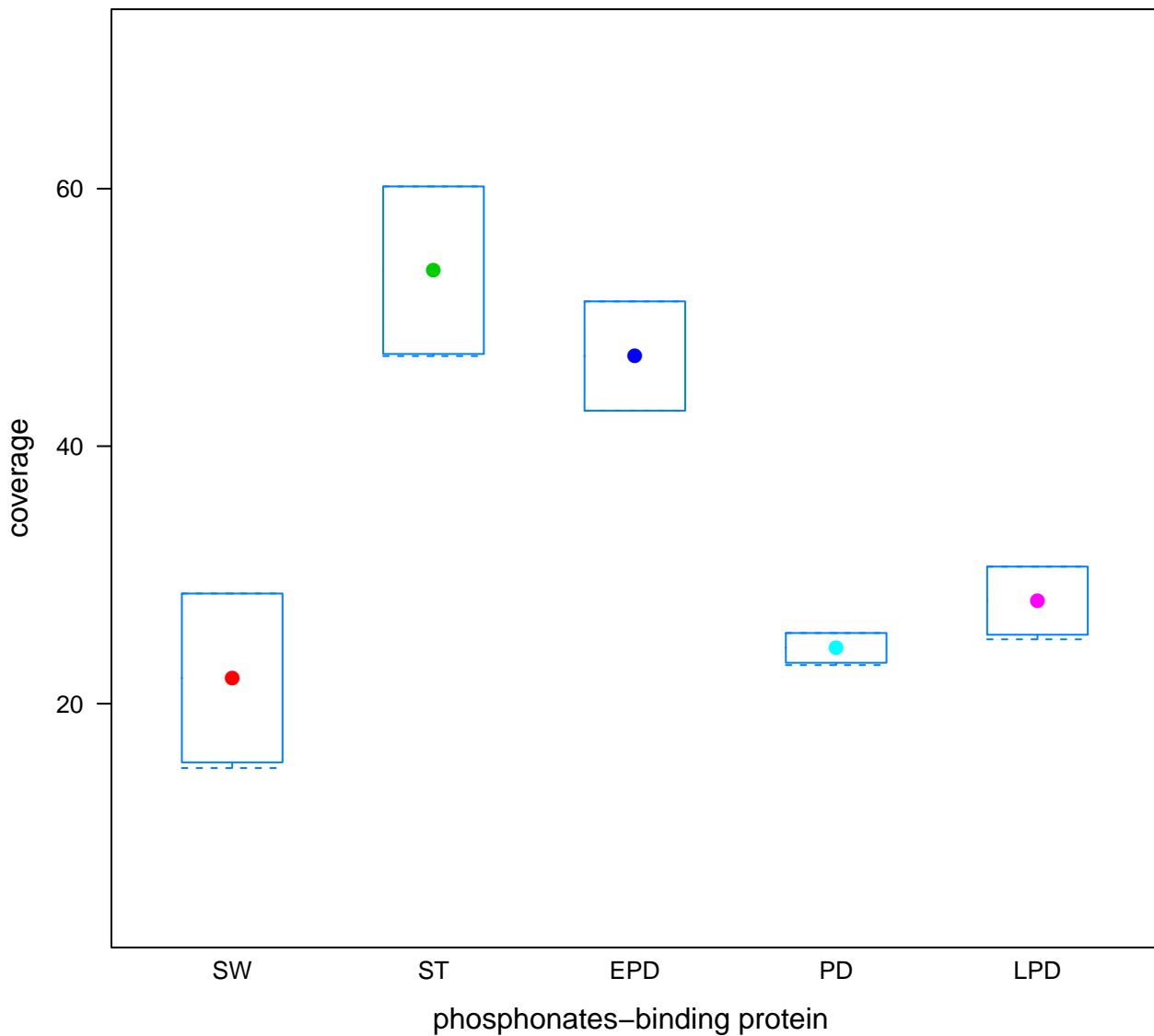

**Fold of change: 2.08**  
**baySeq likelihood: 0.999**

Supplement: Additional file 9: Figure S2 — Expression profiles of all identified CCR genes. [file 1471-2164-14-450-S9.zip › FigureS2/CCNA_00367.pdf]

# CCNA\_00368

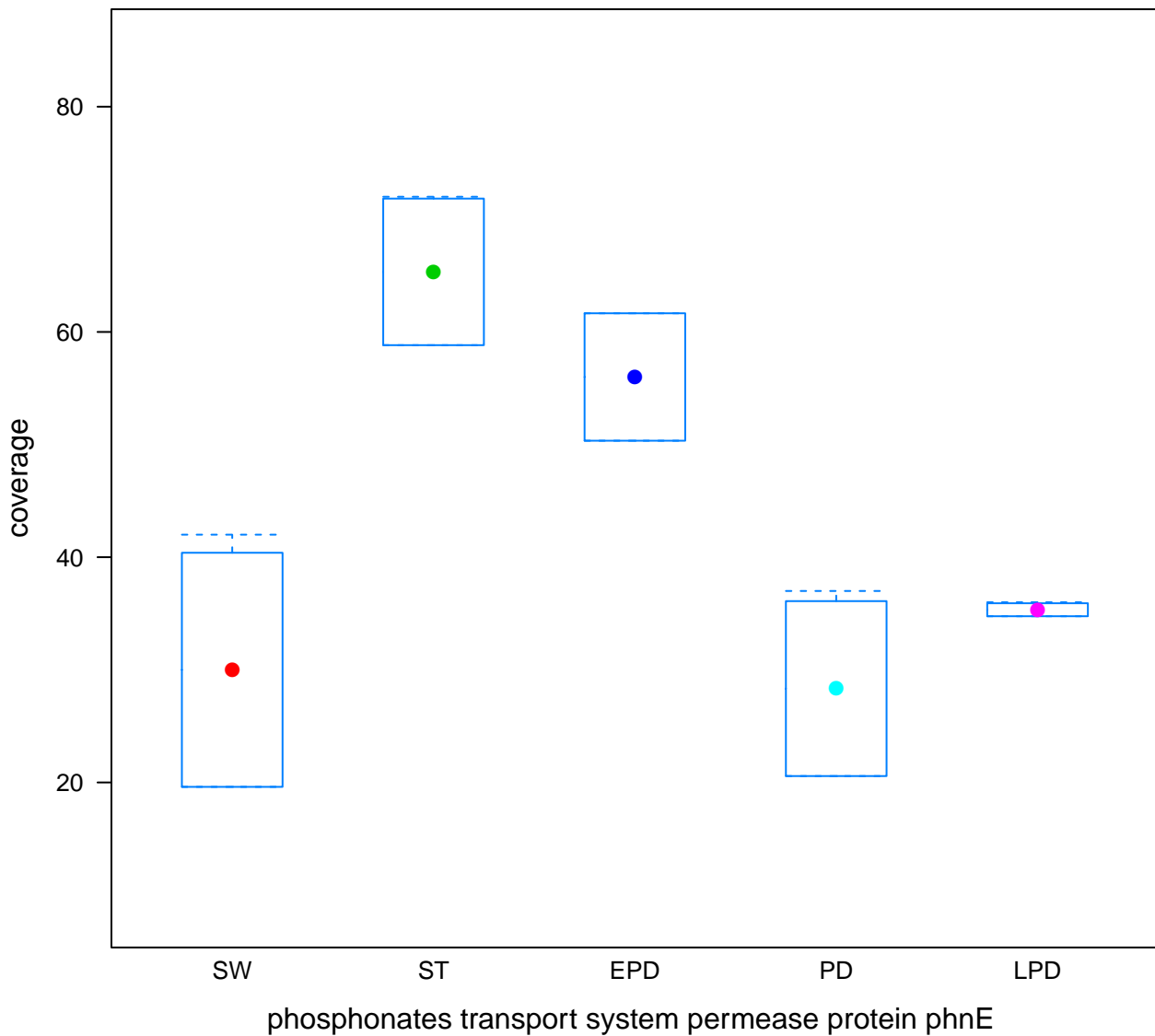

**Fold of change: 2.31**  
**baySeq likelihood: 0.991**

Supplement: Additional file 9: Figure S2 — Expression profiles of all identified CCR genes. [file 1471-2164-14-450-S9.zip › FigureS2/CCNA_00368.pdf]

# CCNA\_00375

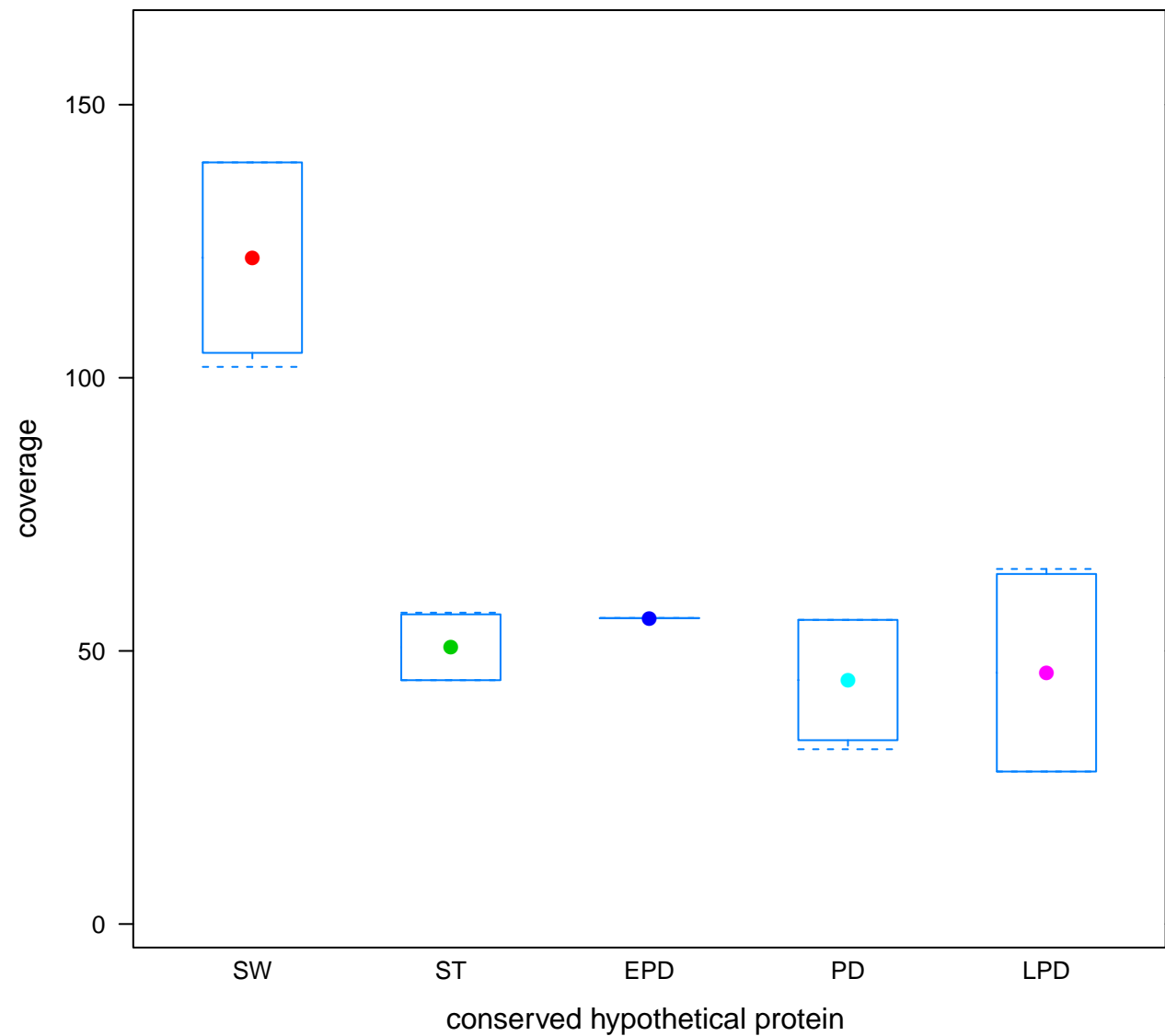

**Fold of change: 2.96**  
**baySeq likelihood: 0.999**

Supplement: Additional file 9: Figure S2 — Expression profiles of all identified CCR genes. [file 1471-2164-14-450-S9.zip › FigureS2/CCNA_00375.pdf]

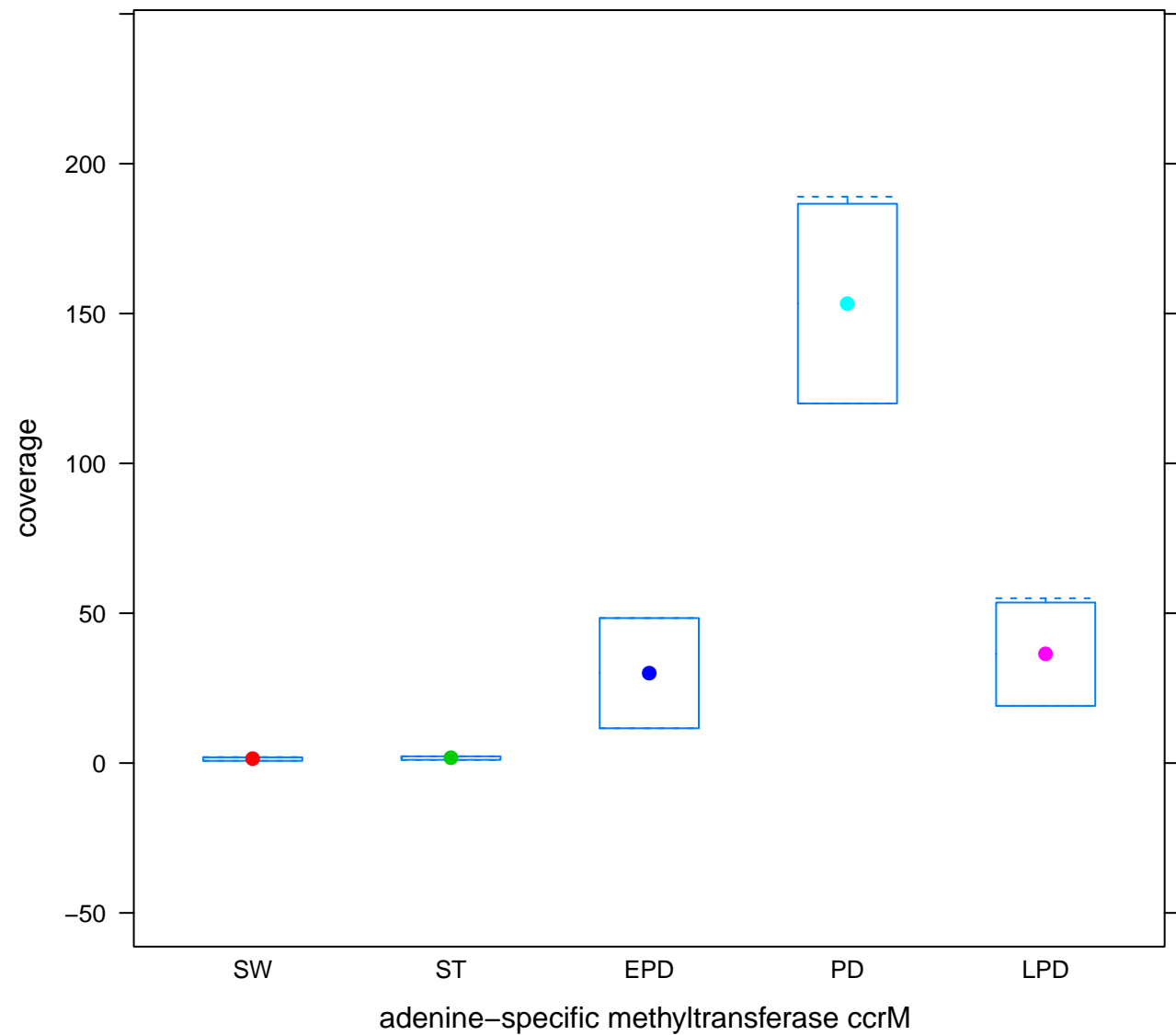

**Fold of change: 153.33**

**baySeq likelihood: 1**

Supplement: Additional file 9: Figure S2 — Expression profiles of all identified CCR genes. [file 1471-2164-14-450-S9.zip › FigureS2/CCNA_00382.pdf]

# CCNA\_00383

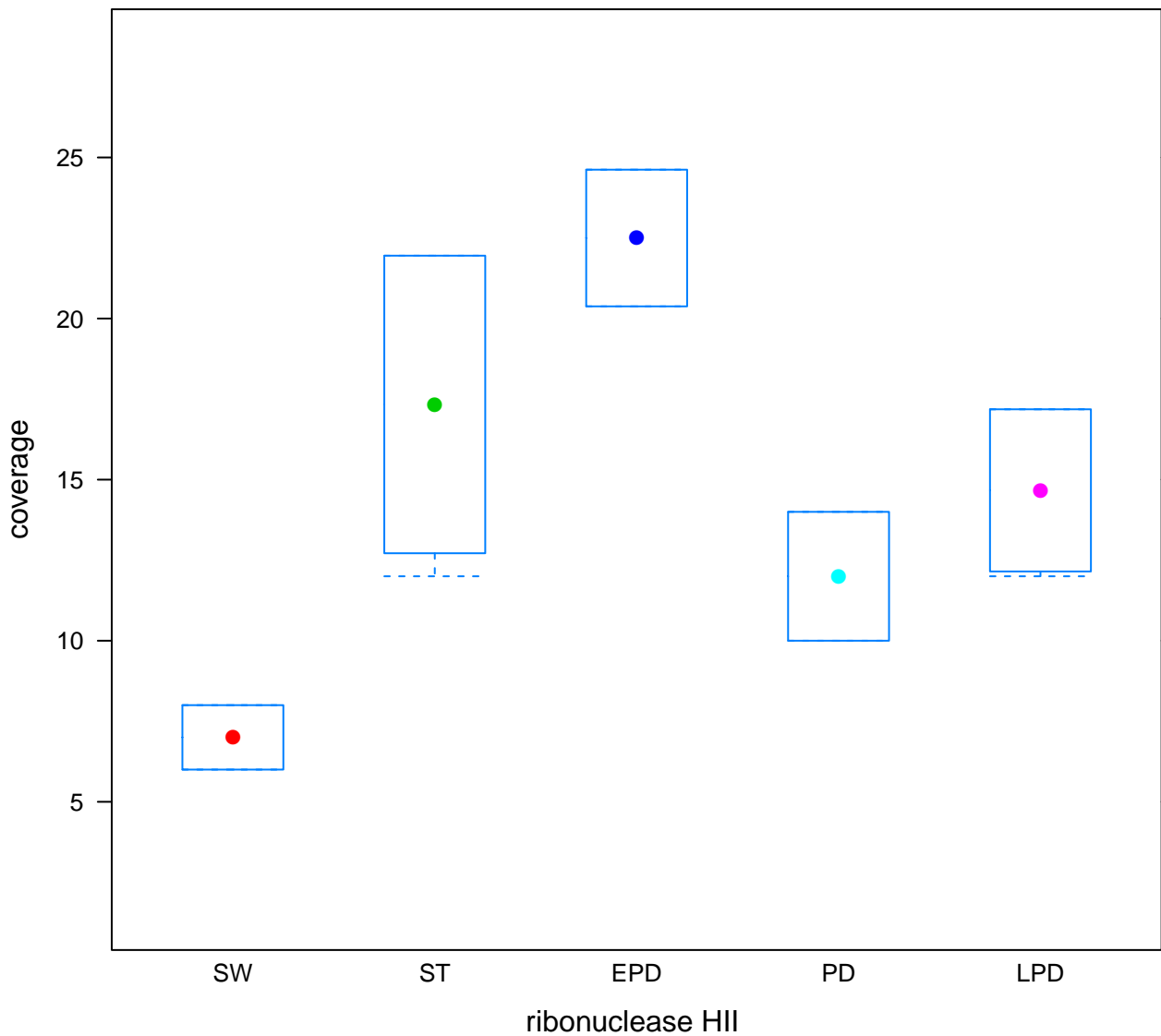

**Fold of change: 3.46**  
**baySeq likelihood: 0.75**

Supplement: Additional file 9: Figure S2 — Expression profiles of all identified CCR genes. [file 1471-2164-14-450-S9.zip › FigureS2/CCNA_00383.pdf]

# CCNA\_00386

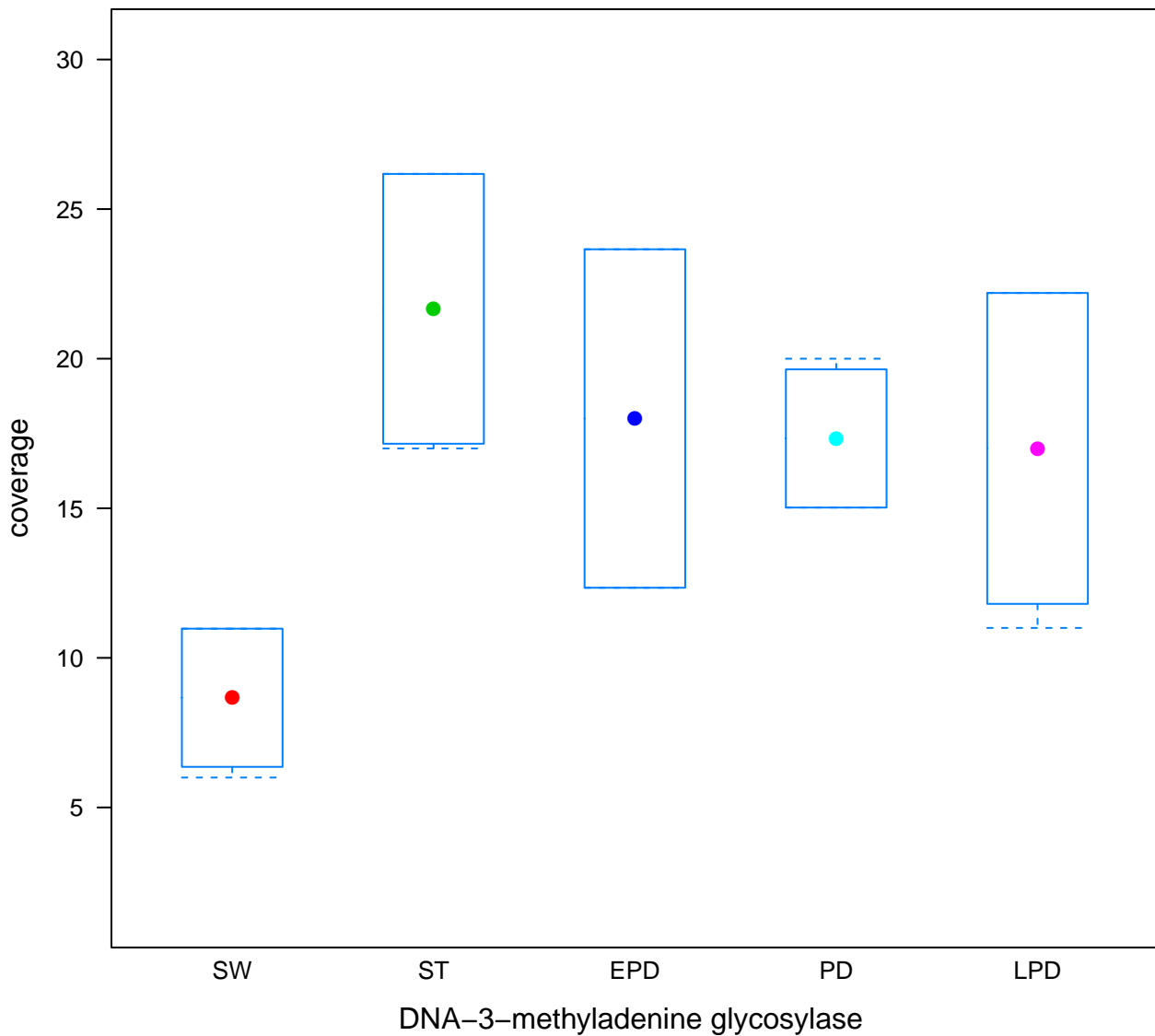

**Fold of change: 2.44**  
**baySeq likelihood: 0.942**

Supplement: Additional file 9: Figure S2 — Expression profiles of all identified CCR genes. [file 1471-2164-14-450-S9.zip › FigureS2/CCNA_00386.pdf]

# CCNA\_00388

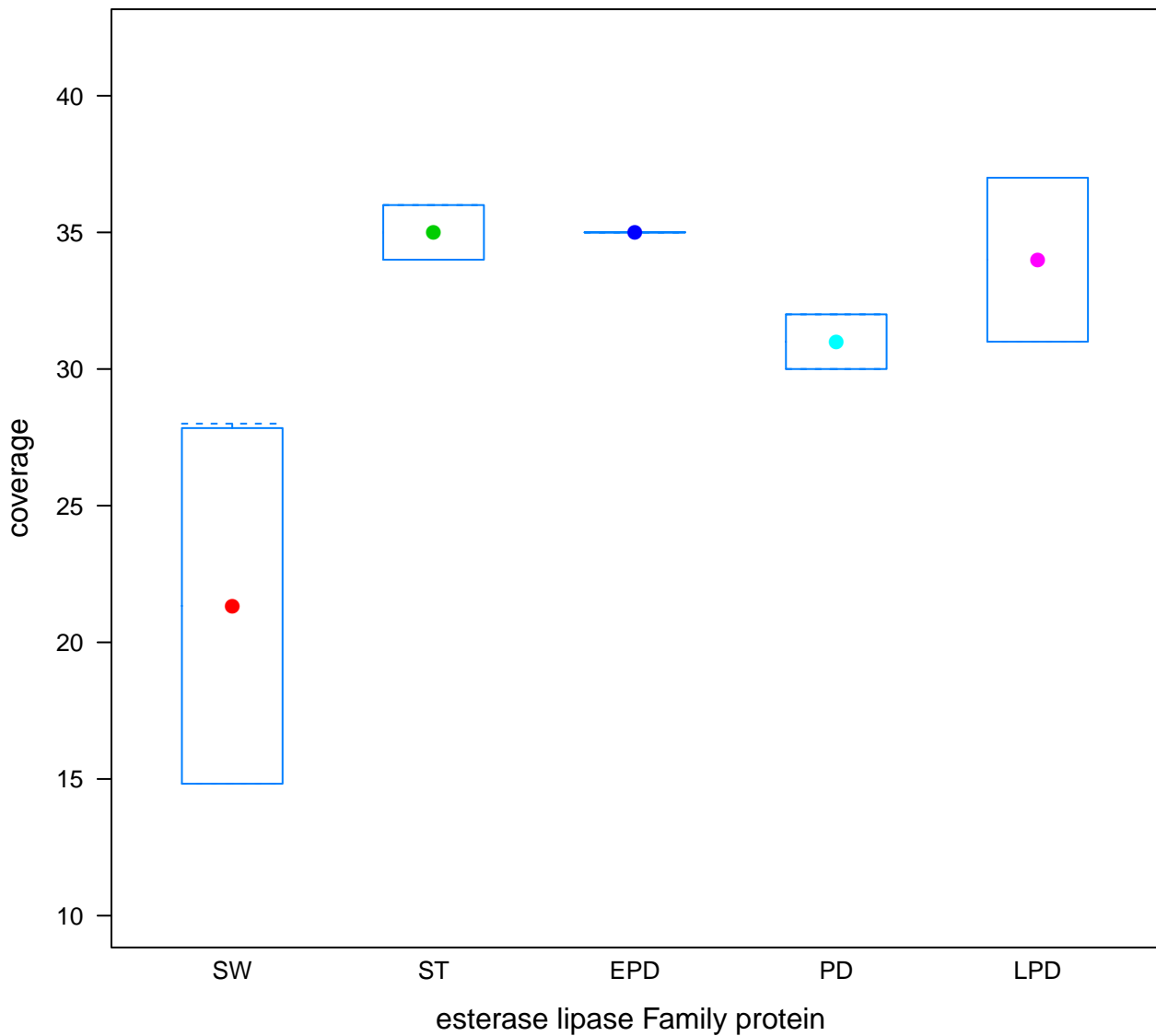

**Fold of change: 1.94**  
**baySeq likelihood: 0.729**

Supplement: Additional file 9: Figure S2 — Expression profiles of all identified CCR genes. [file 1471-2164-14-450-S9.zip › FigureS2/CCNA_00388.pdf]

# CCNA\_00390

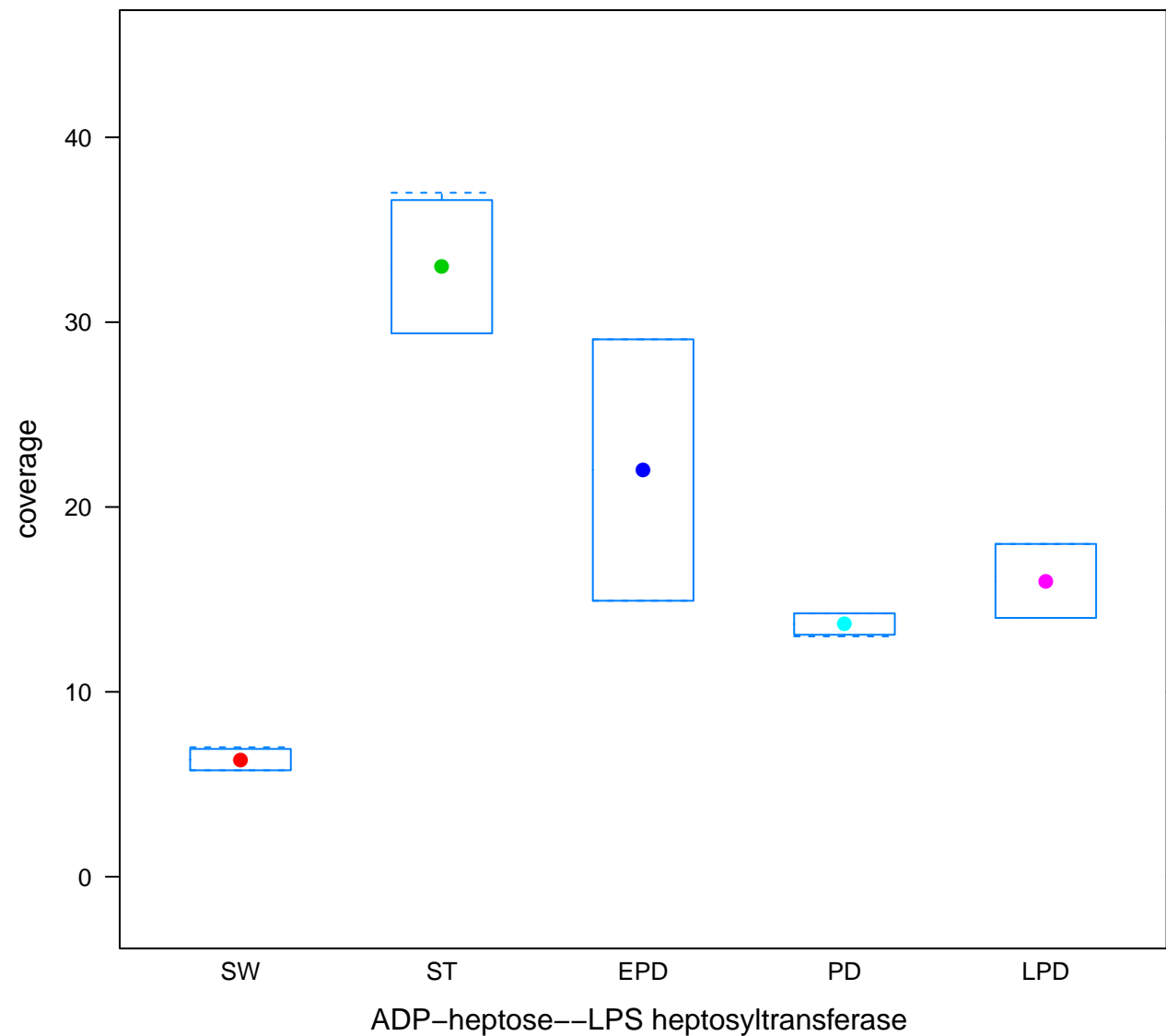

**Fold of change: 5.17**  
**baySeq likelihood: 0.637**

Supplement: Additional file 9: Figure S2 — Expression profiles of all identified CCR genes. [file 1471-2164-14-450-S9.zip › FigureS2/CCNA_00390.pdf]

# CCNA\_00391

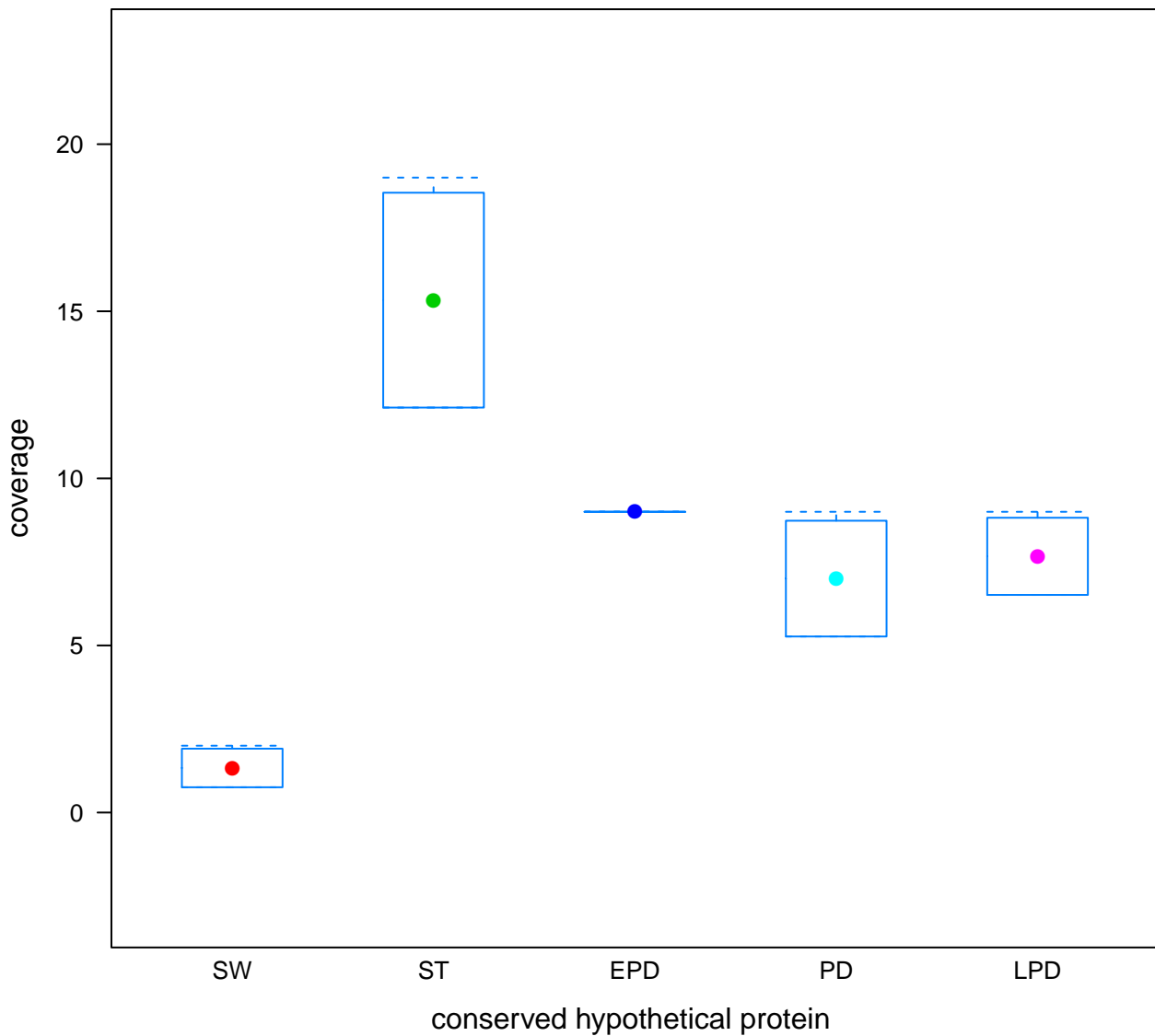

**Fold of change: 13.5**  
**baySeq likelihood: 0.985**

Supplement: Additional file 9: Figure S2 — Expression profiles of all identified CCR genes. [file 1471-2164-14-450-S9.zip › FigureS2/CCNA_00391.pdf]

# CCNA\_00396

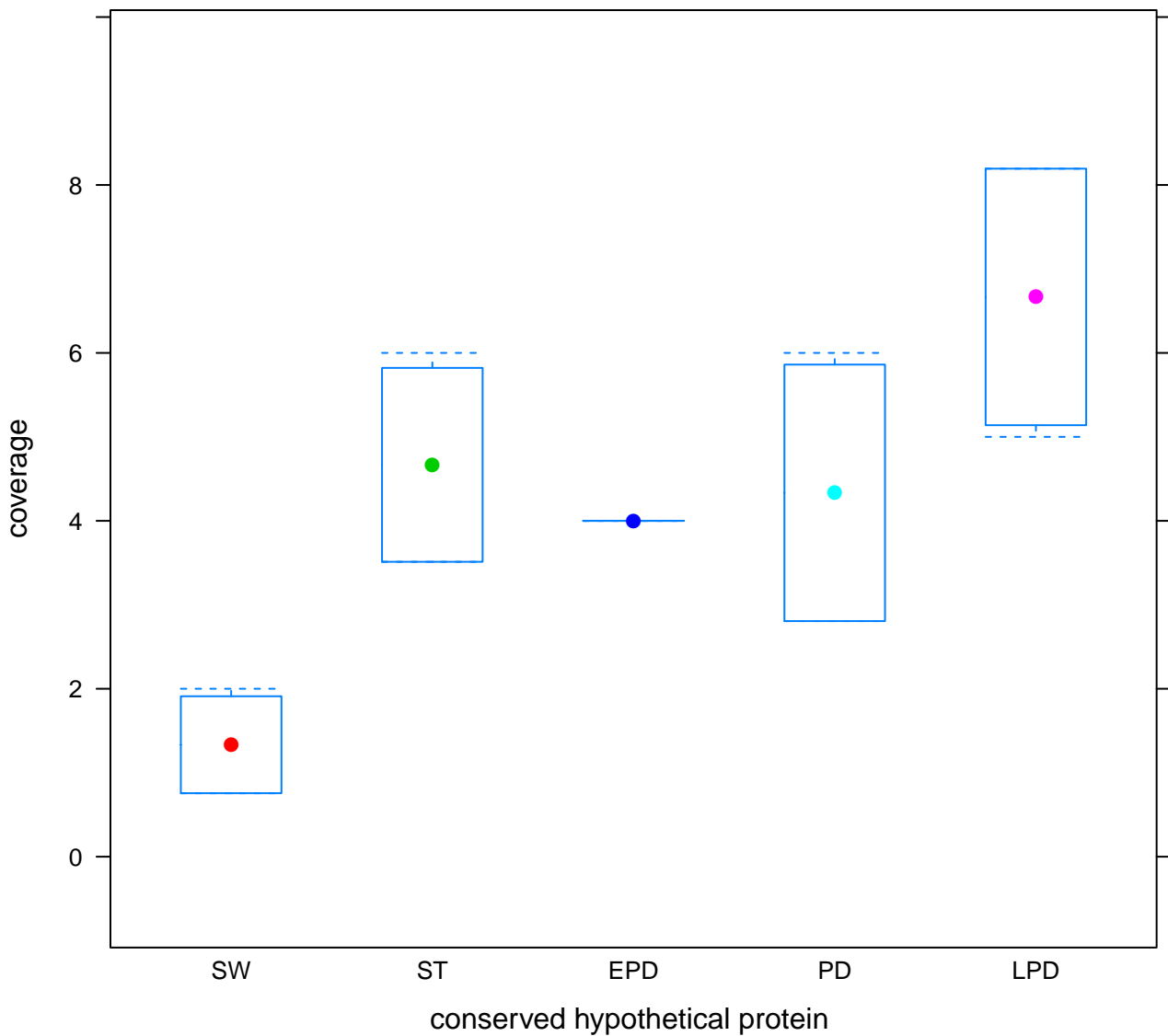

**Fold of change: 6.67**  
**baySeq likelihood: 0.674**

Supplement: Additional file 9: Figure S2 — Expression profiles of all identified CCR genes. [file 1471-2164-14-450-S9.zip › FigureS2/CCNA_00396.pdf]

# CCNA\_00401

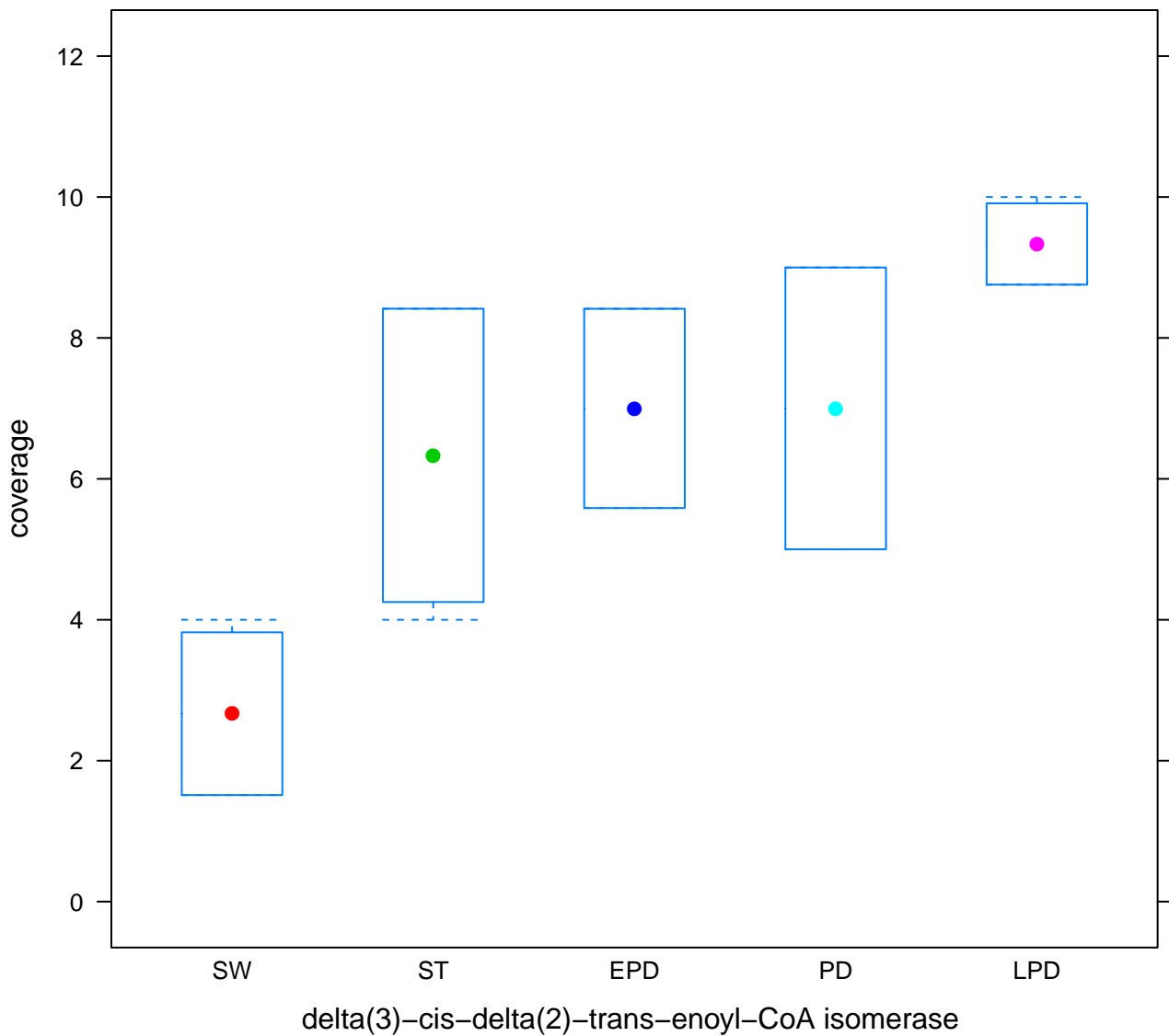

**Fold of change: 4.67**  
**baySeq likelihood: 0.712**

Supplement: Additional file 9: Figure S2 — Expression profiles of all identified CCR genes. [file 1471-2164-14-450-S9.zip › FigureS2/CCNA_00401.pdf]

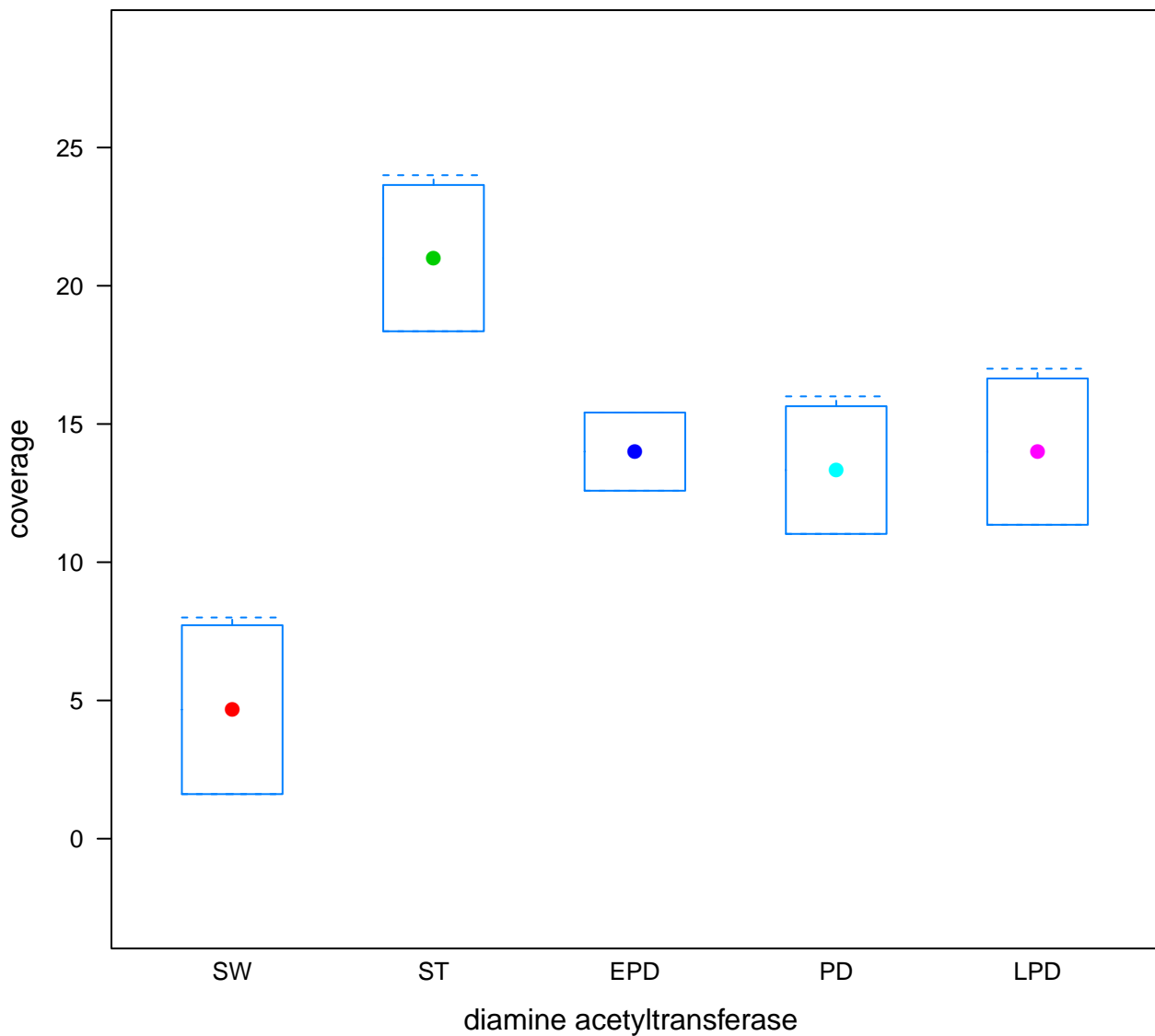

**Fold of change: 6.5**  
**baySeq likelihood: 0.995**

Supplement: Additional file 9: Figure S2 — Expression profiles of all identified CCR genes. [file 1471-2164-14-450-S9.zip › FigureS2/CCNA_00407.pdf]

# CCNA\_00410

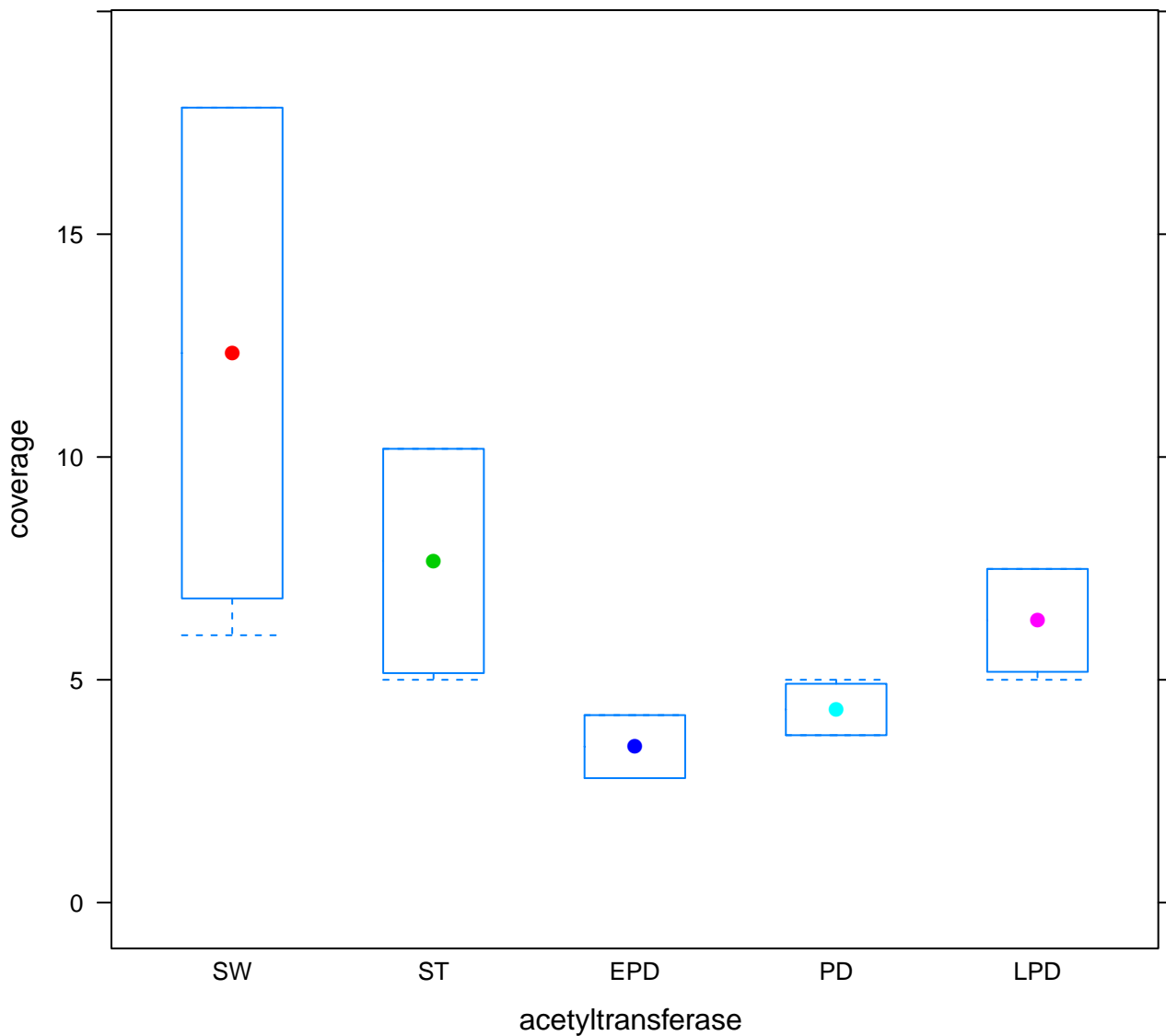

**Fold of change: 3**  
**baySeq likelihood: 0.783**

Supplement: Additional file 9: Figure S2 — Expression profiles of all identified CCR genes. [file 1471-2164-14-450-S9.zip › FigureS2/CCNA_00410.pdf]

# CCNA\_00415

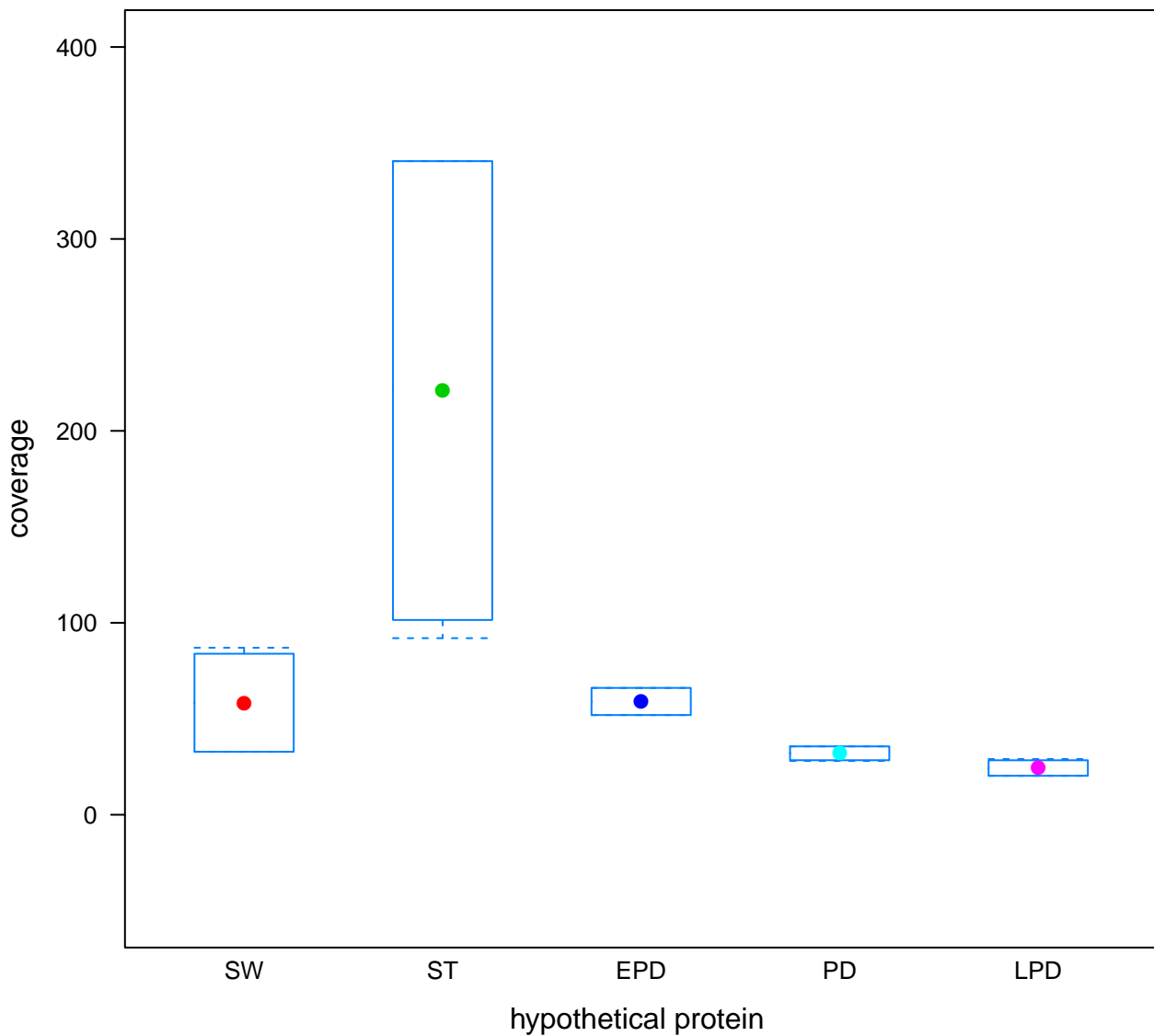

**Fold of change: 11.73**  
**baySeq likelihood: 0.91**

Supplement: Additional file 9: Figure S2 — Expression profiles of all identified CCR genes. [file 1471-2164-14-450-S9.zip › FigureS2/CCNA_00415.pdf]

# CCNA\_00418

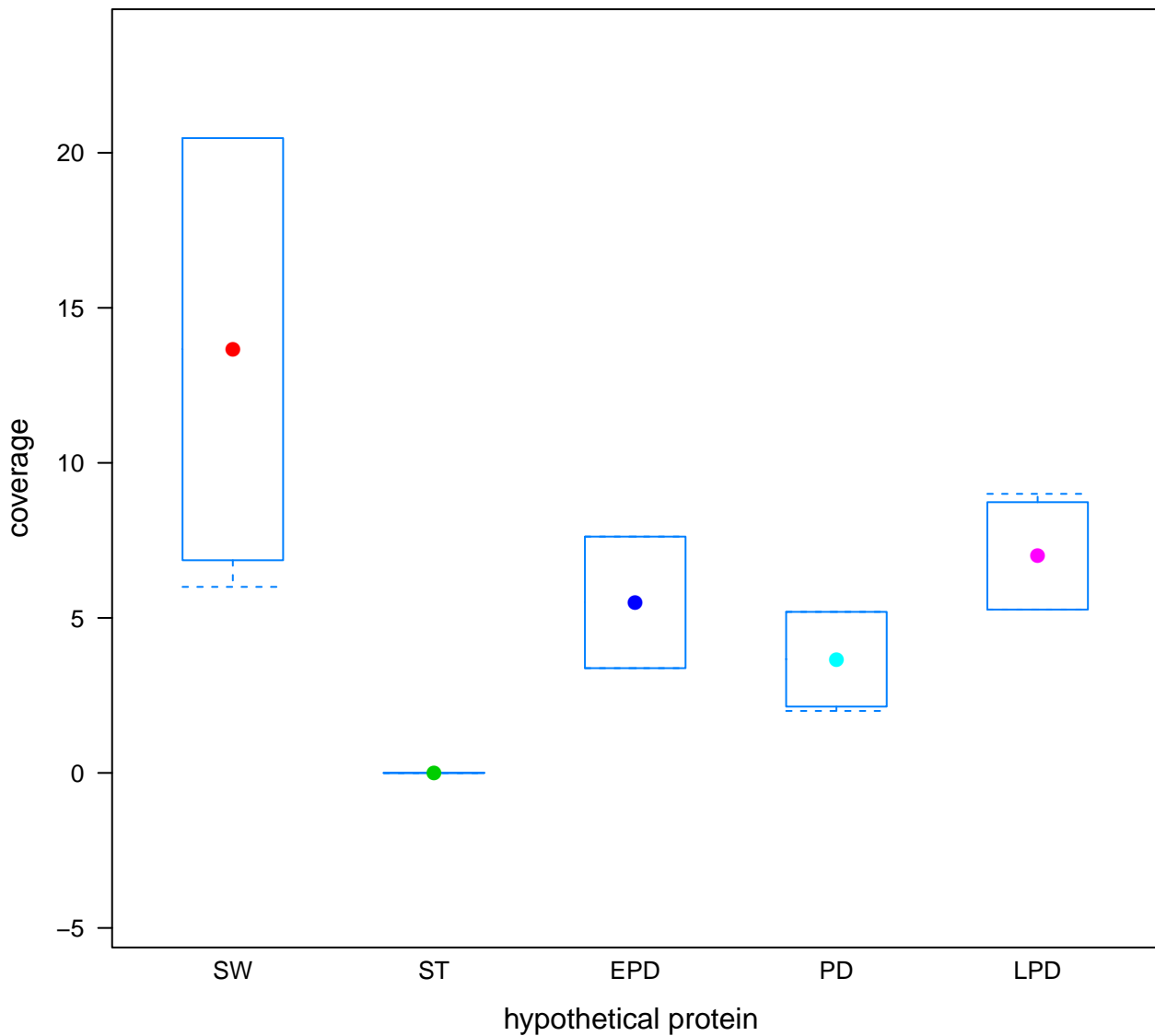

**Fold of change: Inf**  
**baySeq likelihood: 0.865**

Supplement: Additional file 9: Figure S2 — Expression profiles of all identified CCR genes. [file 1471-2164-14-450-S9.zip › FigureS2/CCNA_00418.pdf]

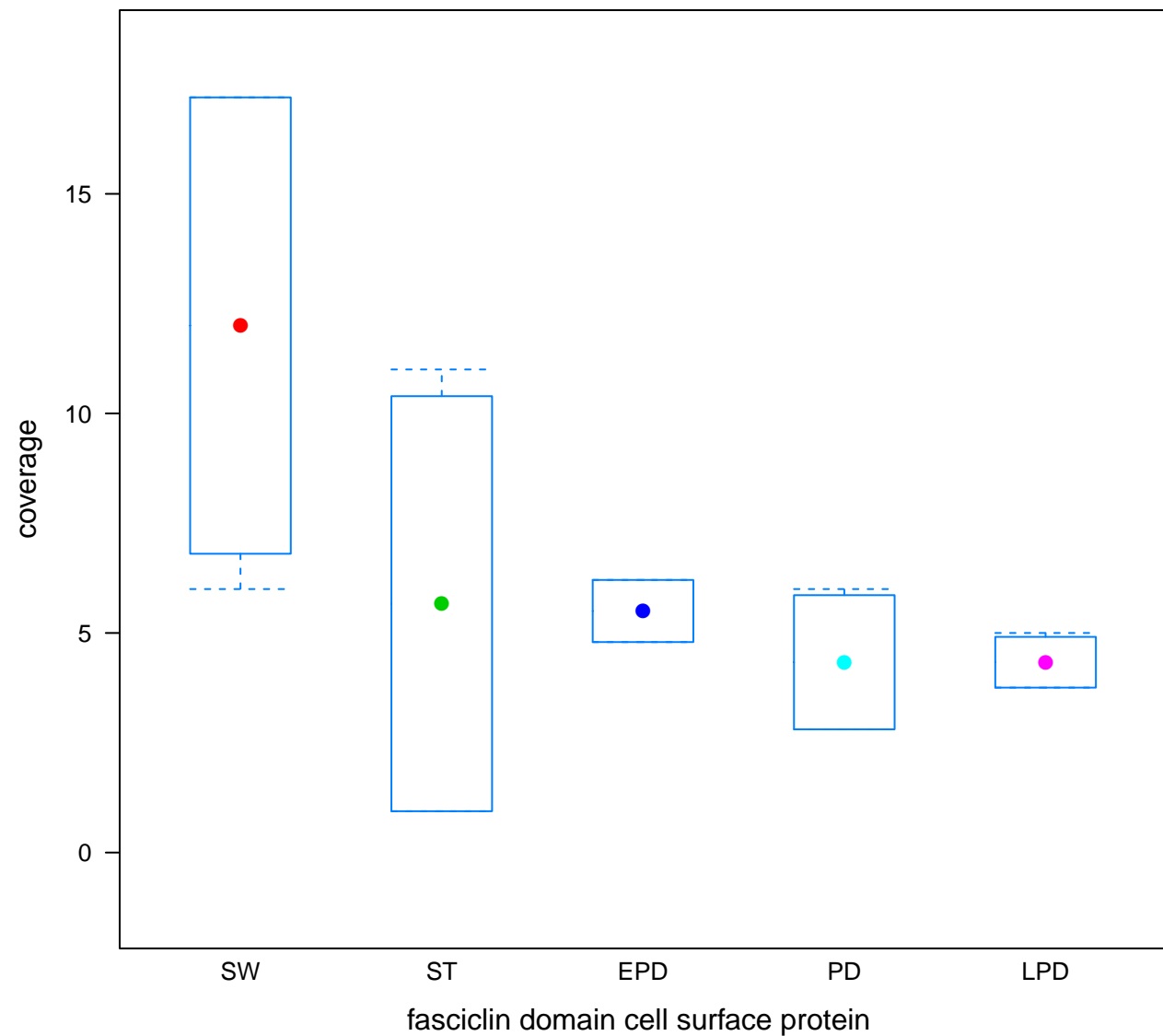

**Fold of change: 3.5**  
**baySeq likelihood: 0.956**

Supplement: Additional file 9: Figure S2 — Expression profiles of all identified CCR genes. [file 1471-2164-14-450-S9.zip › FigureS2/CCNA_00419.pdf]

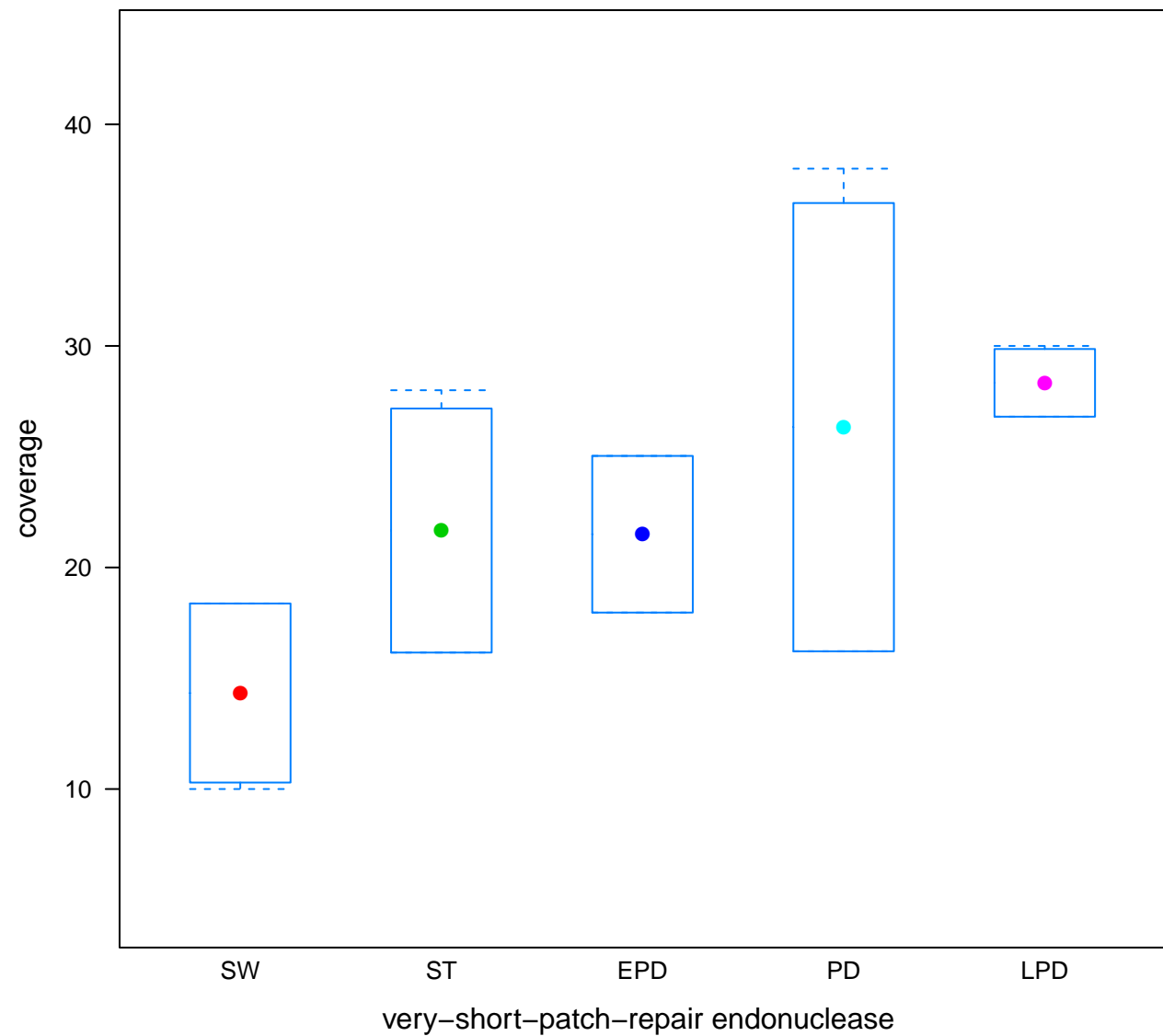

**Fold of change: 2.27**  
**baySeq likelihood: 0.579**

Supplement: Additional file 9: Figure S2 — Expression profiles of all identified CCR genes. [file 1471-2164-14-450-S9.zip › FigureS2/CCNA_00426.pdf]

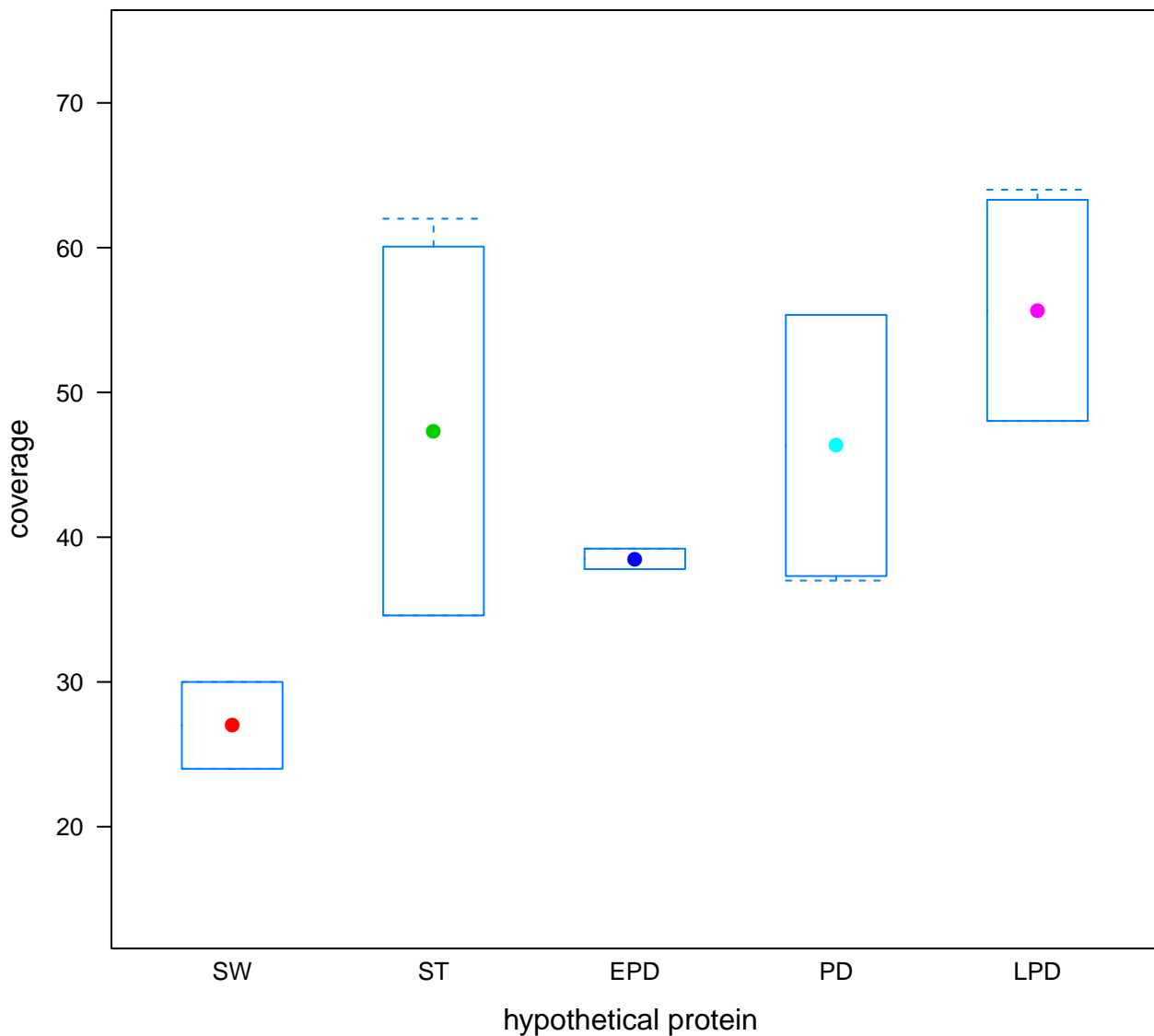

**Fold of change: 2.18**  
**baySeq likelihood: 0.834**

Supplement: Additional file 9: Figure S2 — Expression profiles of all identified CCR genes. [file 1471-2164-14-450-S9.zip › FigureS2/CCNA_00427.pdf]

# CCNA\_00428

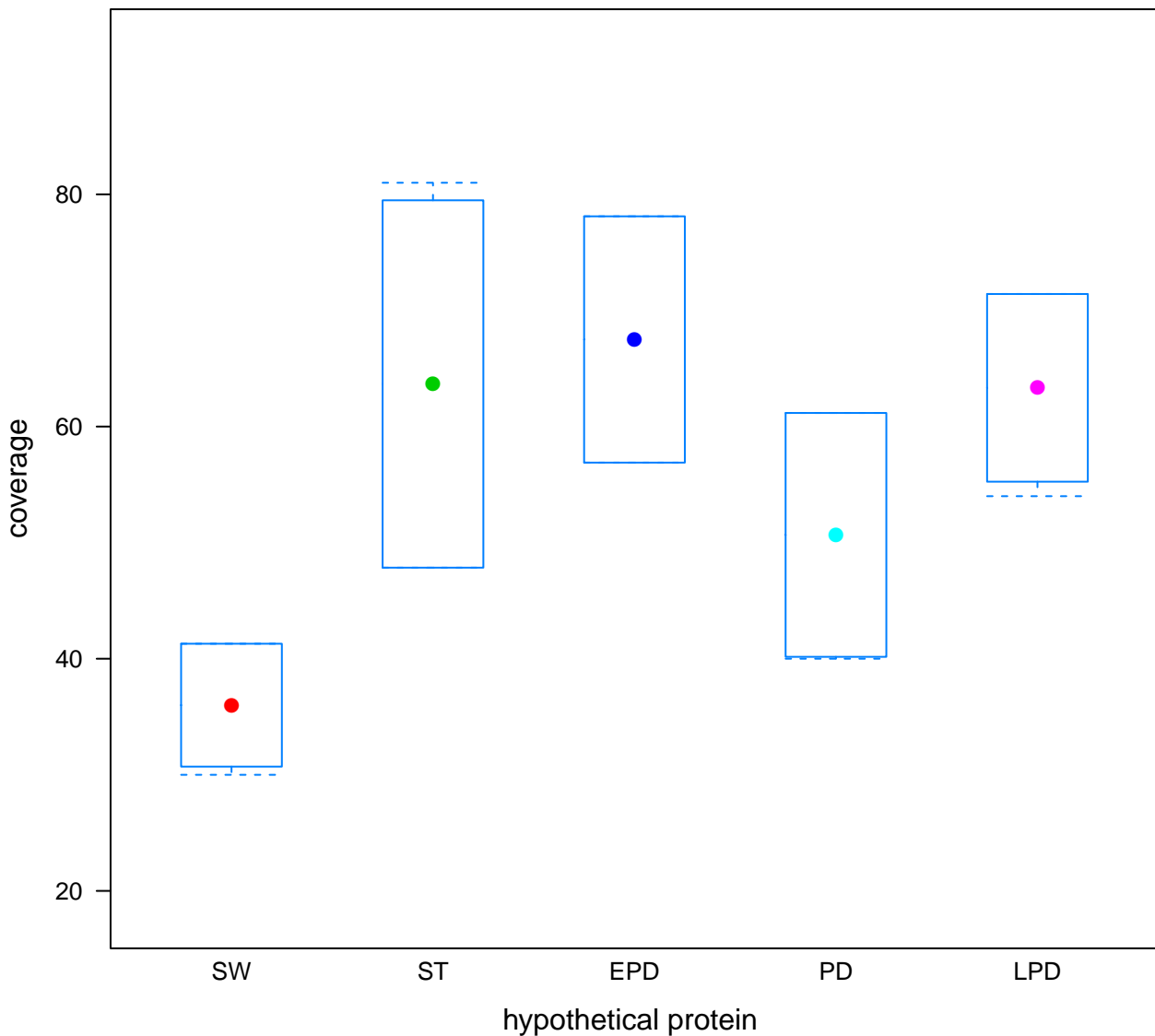

**Fold of change: 1.99**  
**baySeq likelihood: 0.684**

Supplement: Additional file 9: Figure S2 — Expression profiles of all identified CCR genes. [file 1471-2164-14-450-S9.zip › FigureS2/CCNA_00428.pdf]

# CCNA\_00431

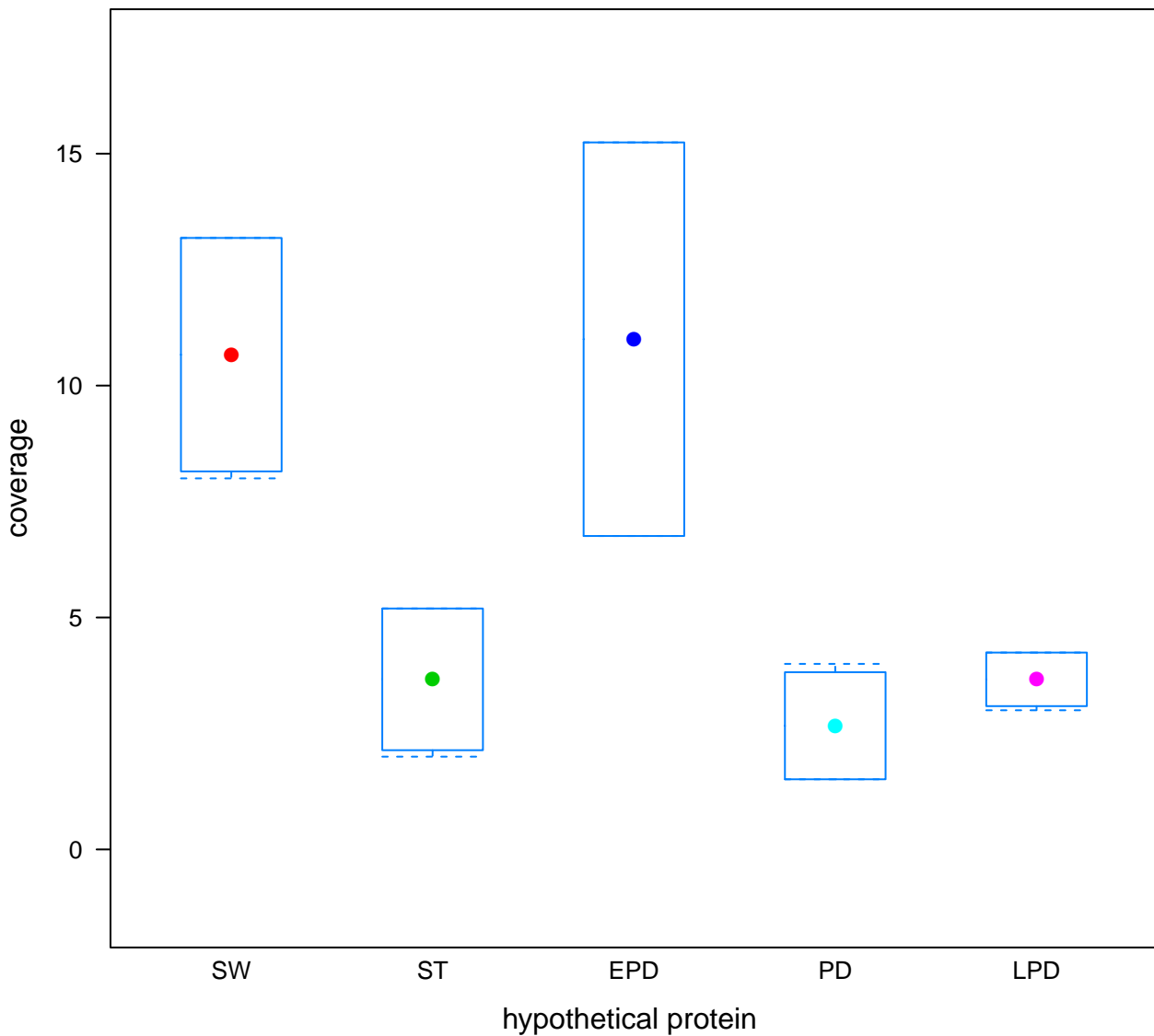

**Fold of change: 4.12**  
**baySeq likelihood: 0.986**

Supplement: Additional file 9: Figure S2 — Expression profiles of all identified CCR genes. [file 1471-2164-14-450-S9.zip › FigureS2/CCNA_00431.pdf]

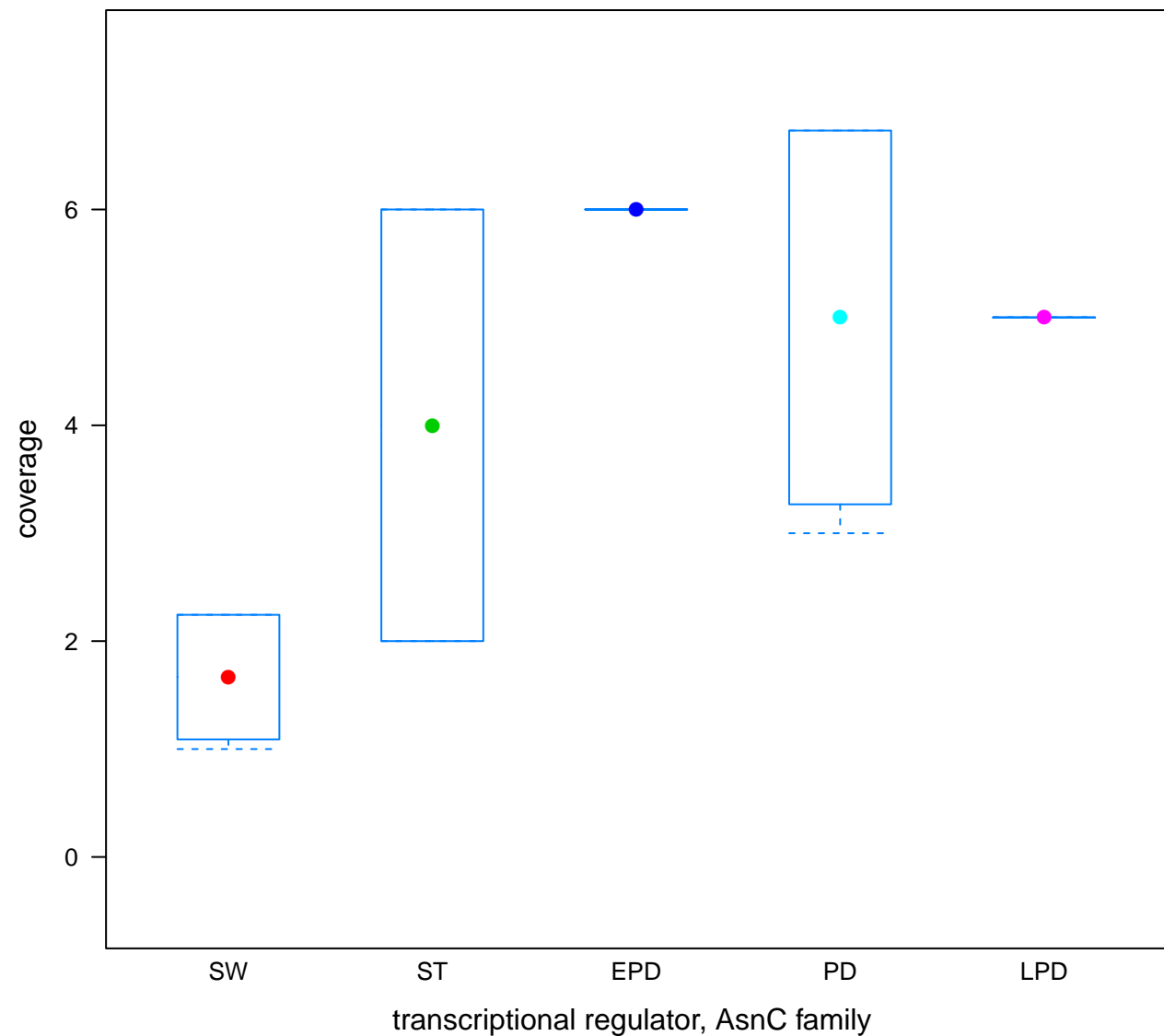

**Fold of change: 4**  
**baySeq likelihood: 0.417**

Supplement: Additional file 9: Figure S2 — Expression profiles of all identified CCR genes. [file 1471-2164-14-450-S9.zip › FigureS2/CCNA_00432.pdf]

# CCNA\_00433

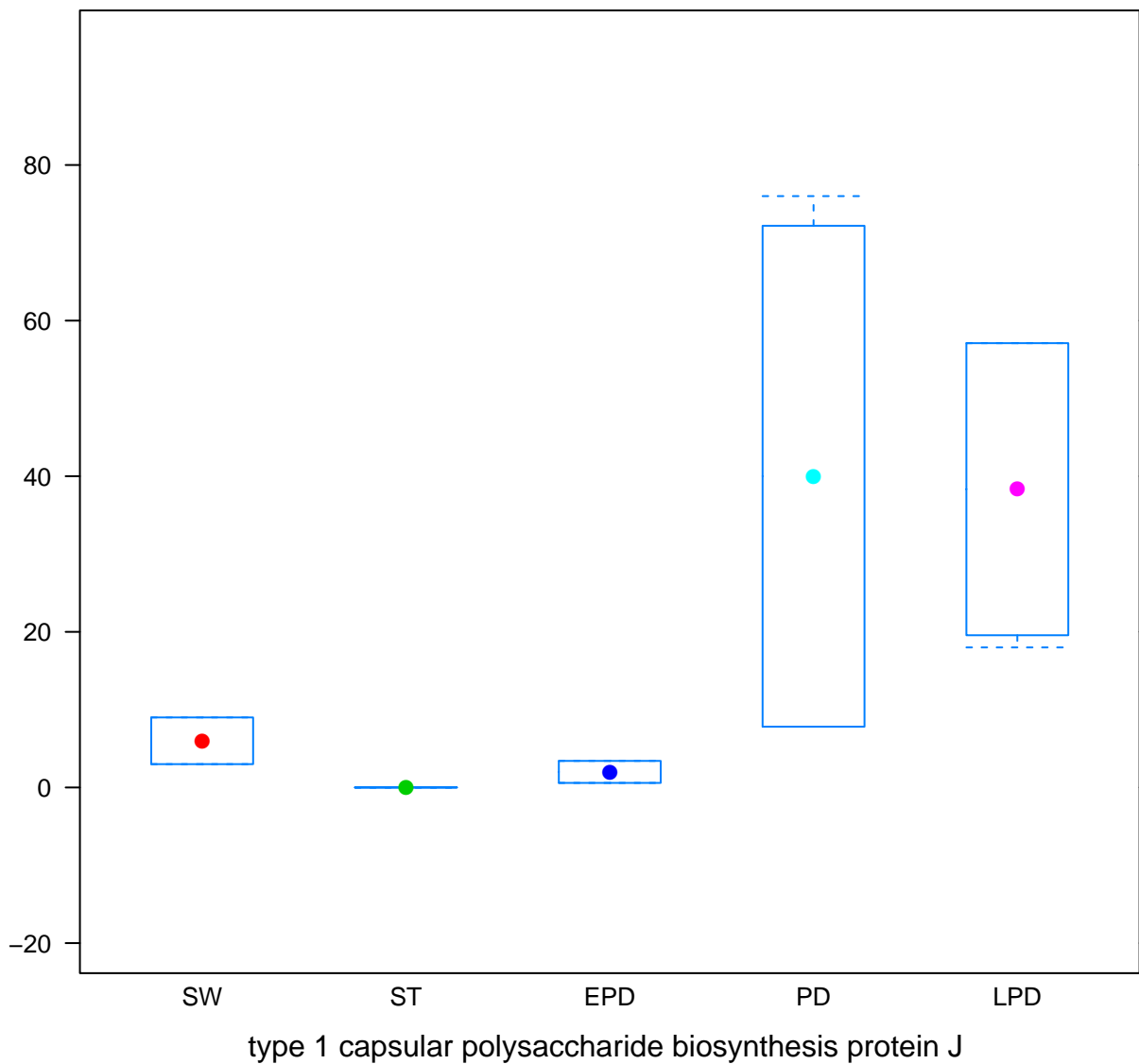

**Fold of change: Inf**  
**baySeq likelihood: 0.706**

Supplement: Additional file 9: Figure S2 — Expression profiles of all identified CCR genes. [file 1471-2164-14-450-S9.zip › FigureS2/CCNA_00433.pdf]

# CCNA\_00437

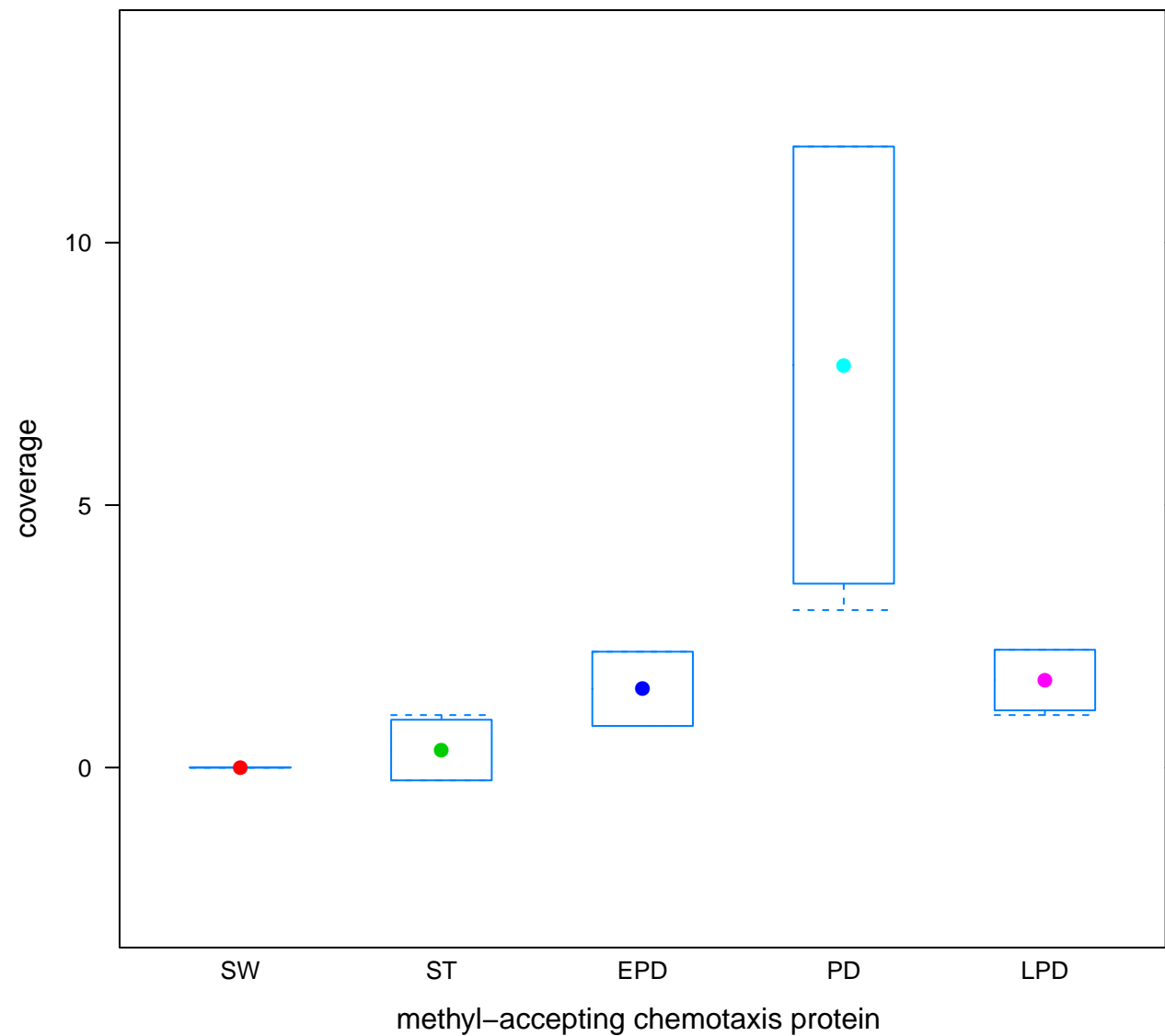

**Fold of change: Inf**  
**baySeq likelihood: 0.921**

Supplement: Additional file 9: Figure S2 — Expression profiles of all identified CCR genes. [file 1471-2164-14-450-S9.zip › FigureS2/CCNA_00437.pdf]

# CCNA\_00438

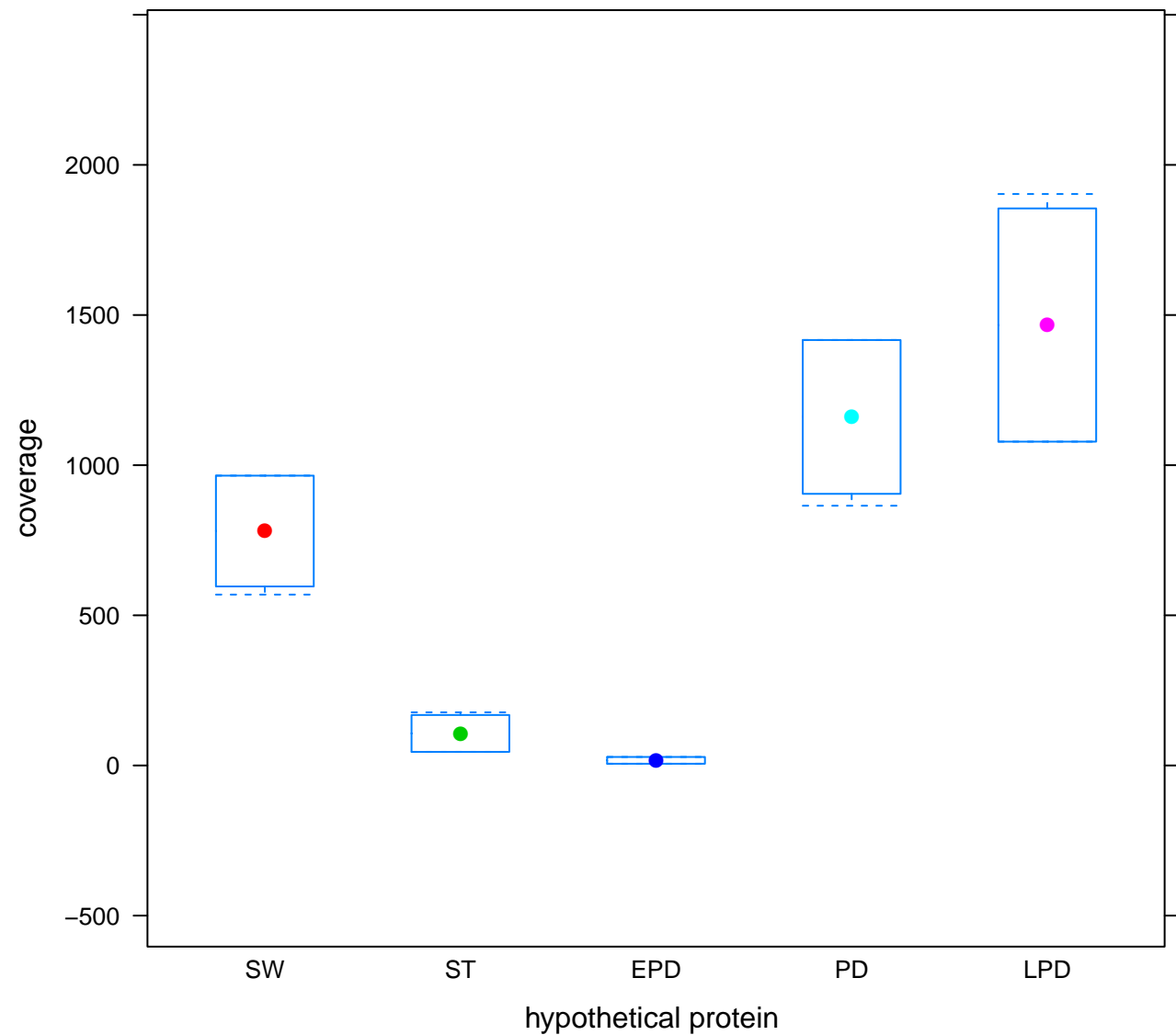

**Fold of change: 86.27**  
**baySeq likelihood: 0.984**

Supplement: Additional file 9: Figure S2 — Expression profiles of all identified CCR genes. [file 1471-2164-14-450-S9.zip › FigureS2/CCNA_00438.pdf]

# CCNA\_00439

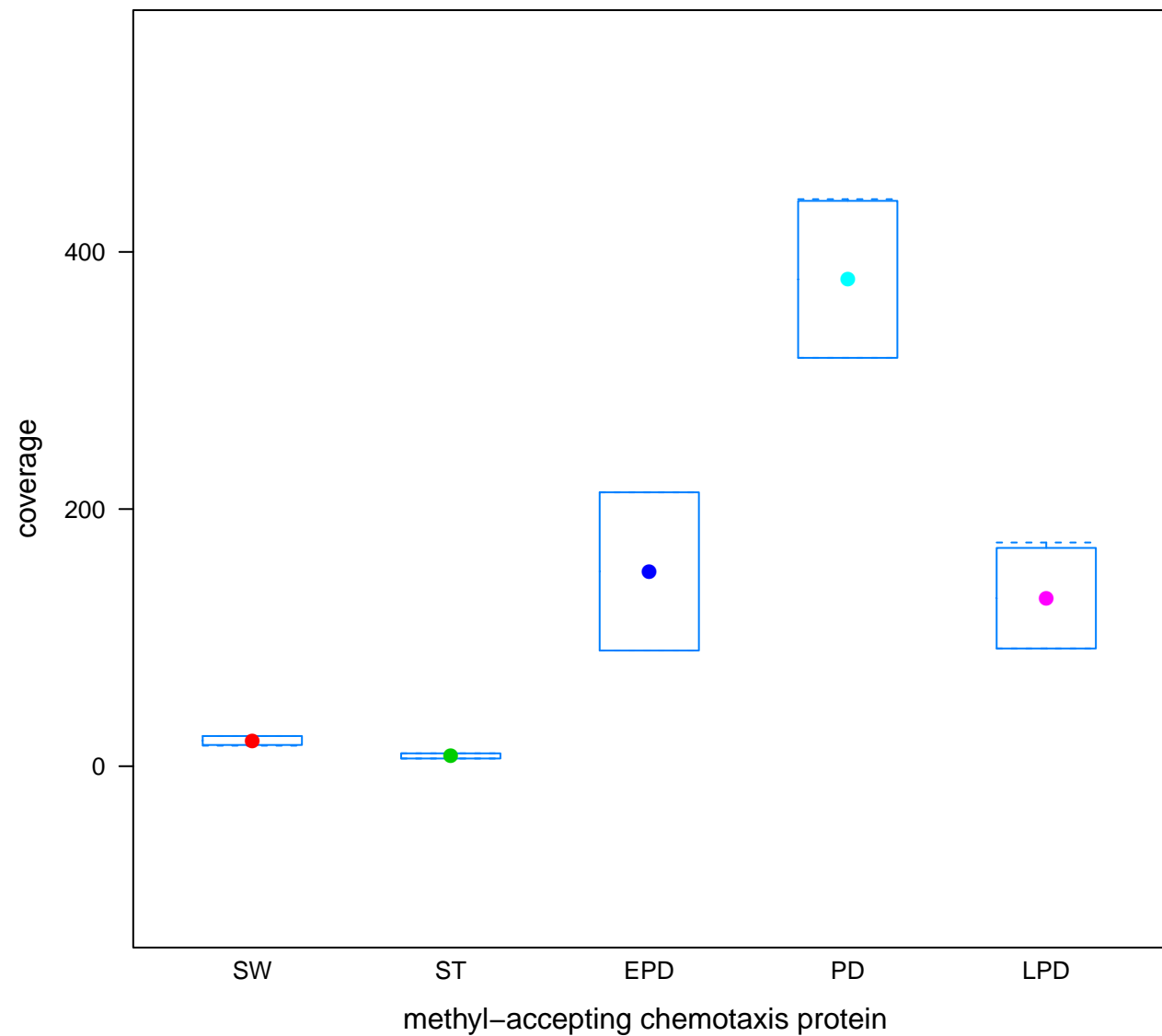

**Fold of change: 54.1**

**baySeq likelihood: 1**

Supplement: Additional file 9: Figure S2 — Expression profiles of all identified CCR genes. [file 1471-2164-14-450-S9.zip › FigureS2/CCNA_00439.pdf]

# CCNA\_00440

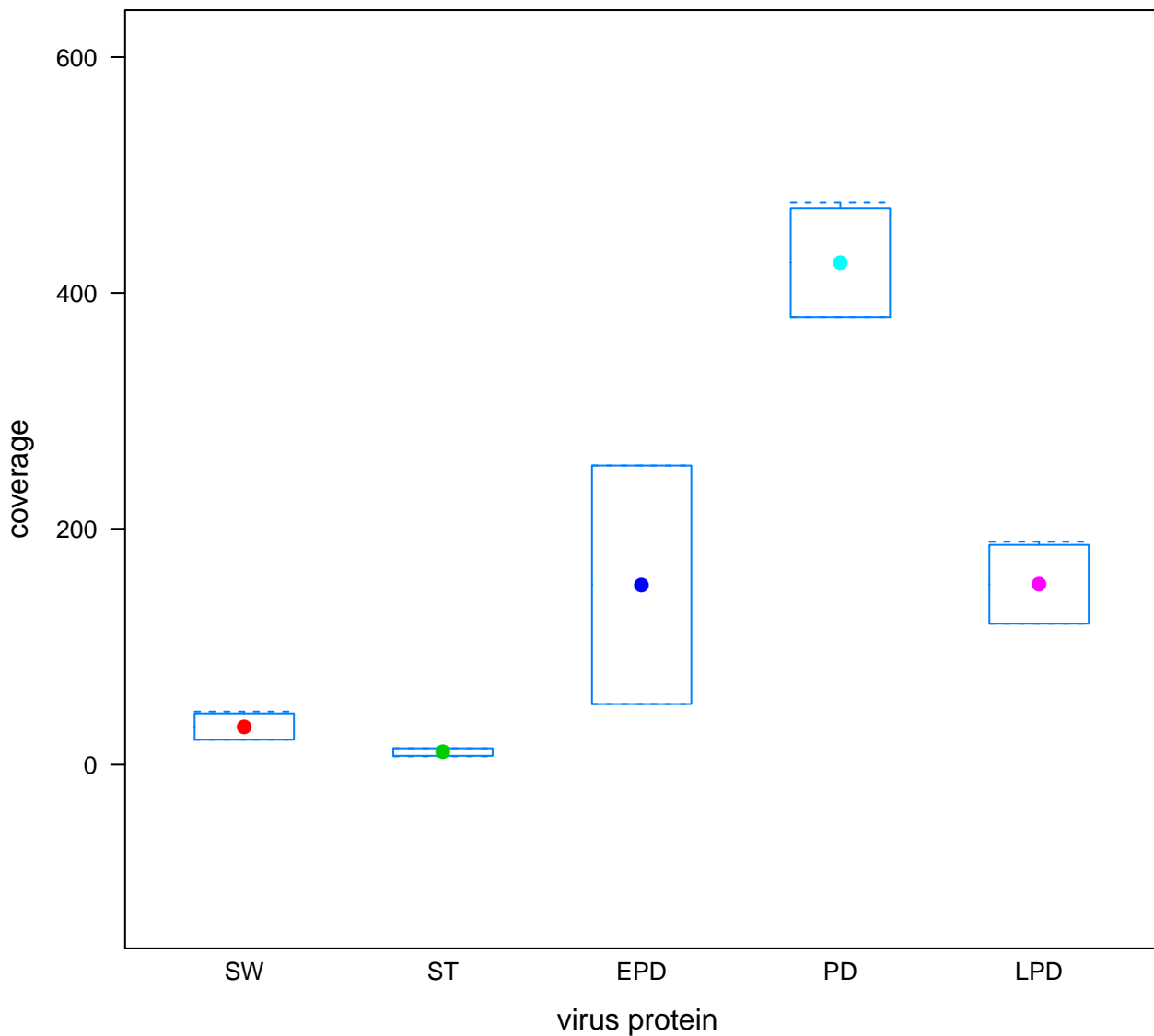

**Fold of change: 44.81**  
**baySeq likelihood: 0.985**

Supplement: Additional file 9: Figure S2 — Expression profiles of all identified CCR genes. [file 1471-2164-14-450-S9.zip › FigureS2/CCNA_00440.pdf]

# cheYI;CCNA\_00441

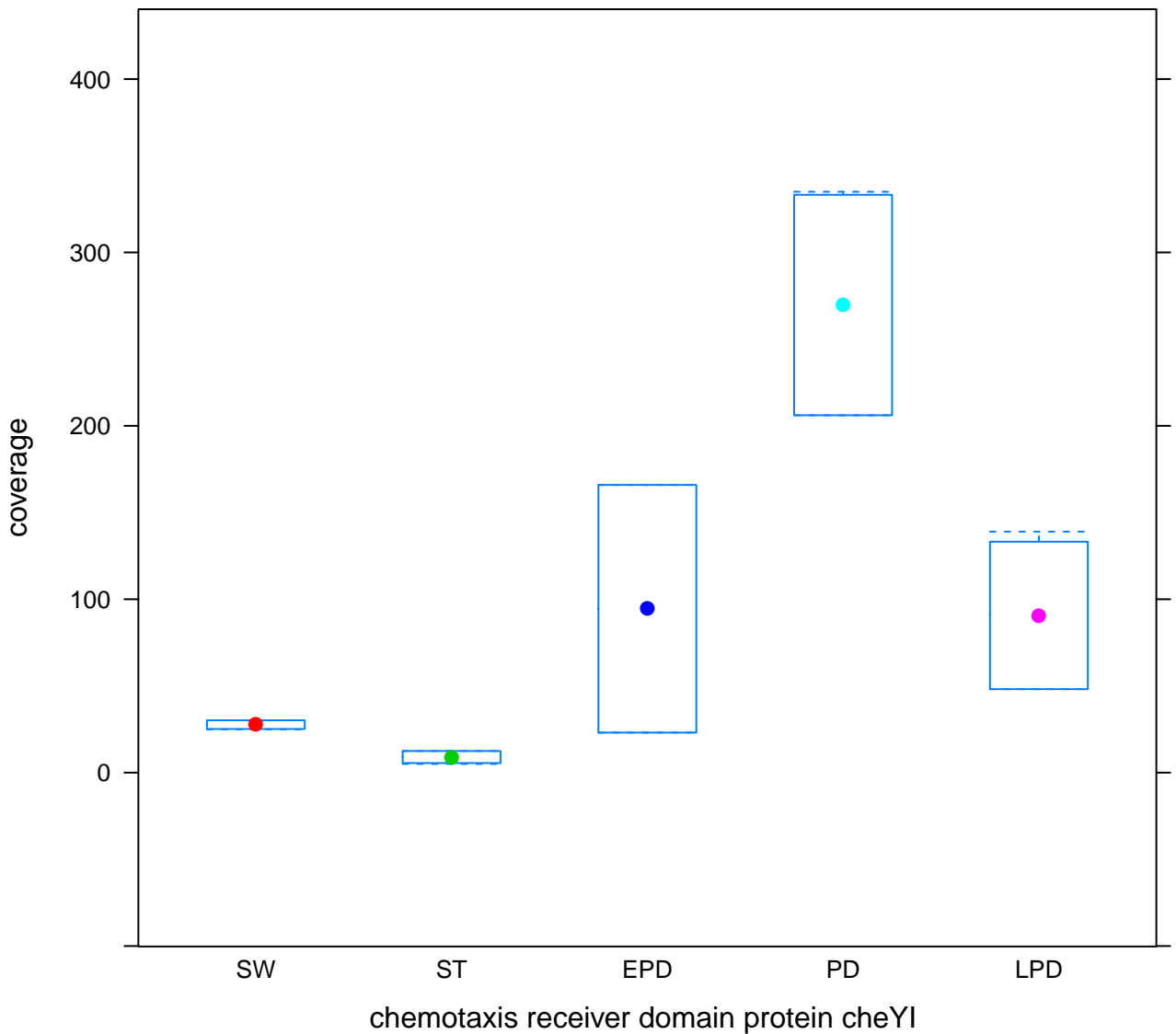

**Fold of change: 33.71**  
**baySeq likelihood: 0.628**

Supplement: Additional file 9: Figure S2 — Expression profiles of all identified CCR genes. [file 1471-2164-14-450-S9.zip › FigureS2/CCNA_00441.pdf]

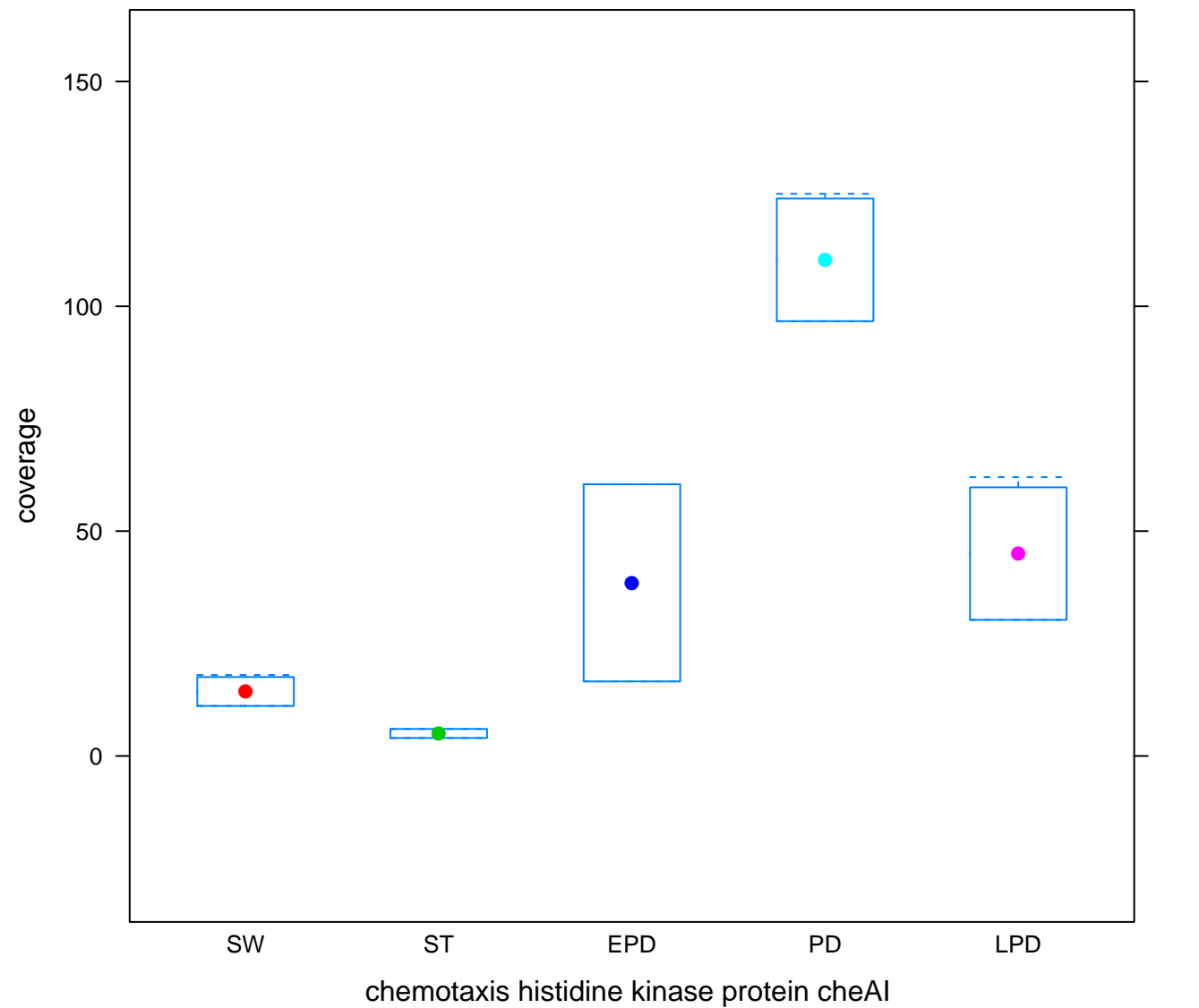

**Fold of change: 22.07**  
**baySeq likelihood: 0.769**

Supplement: Additional file 9: Figure S2 — Expression profiles of all identified CCR genes. [file 1471-2164-14-450-S9.zip › FigureS2/CCNA_00442.pdf]

# CCNA\_00443

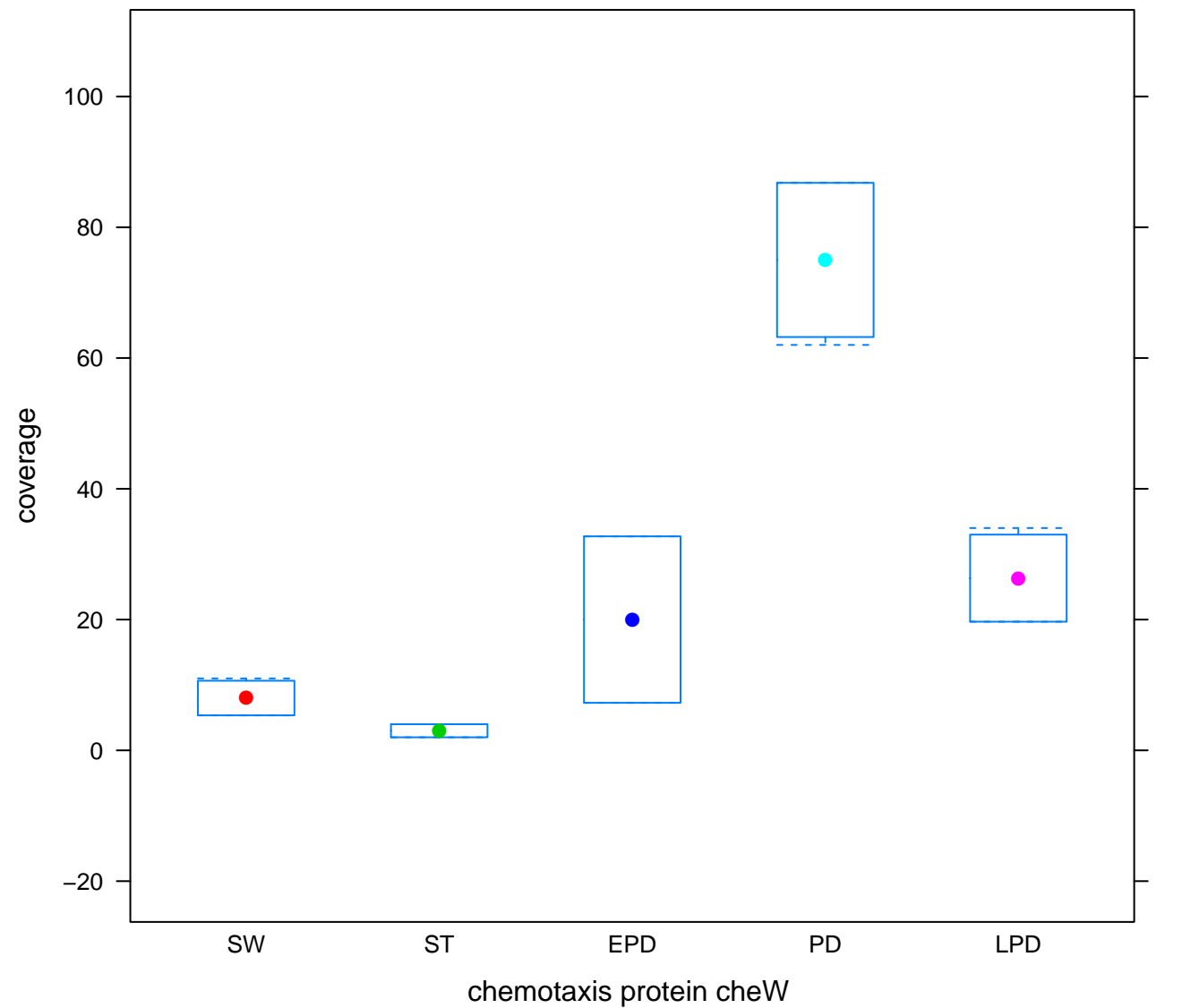

**Fold of change: 21.43**  
**baySeq likelihood: 0.968**

Supplement: Additional file 9: Figure S2 — Expression profiles of all identified CCR genes. [file 1471-2164-14-450-S9.zip › FigureS2/CCNA_00443.pdf]

# CCNA\_00444

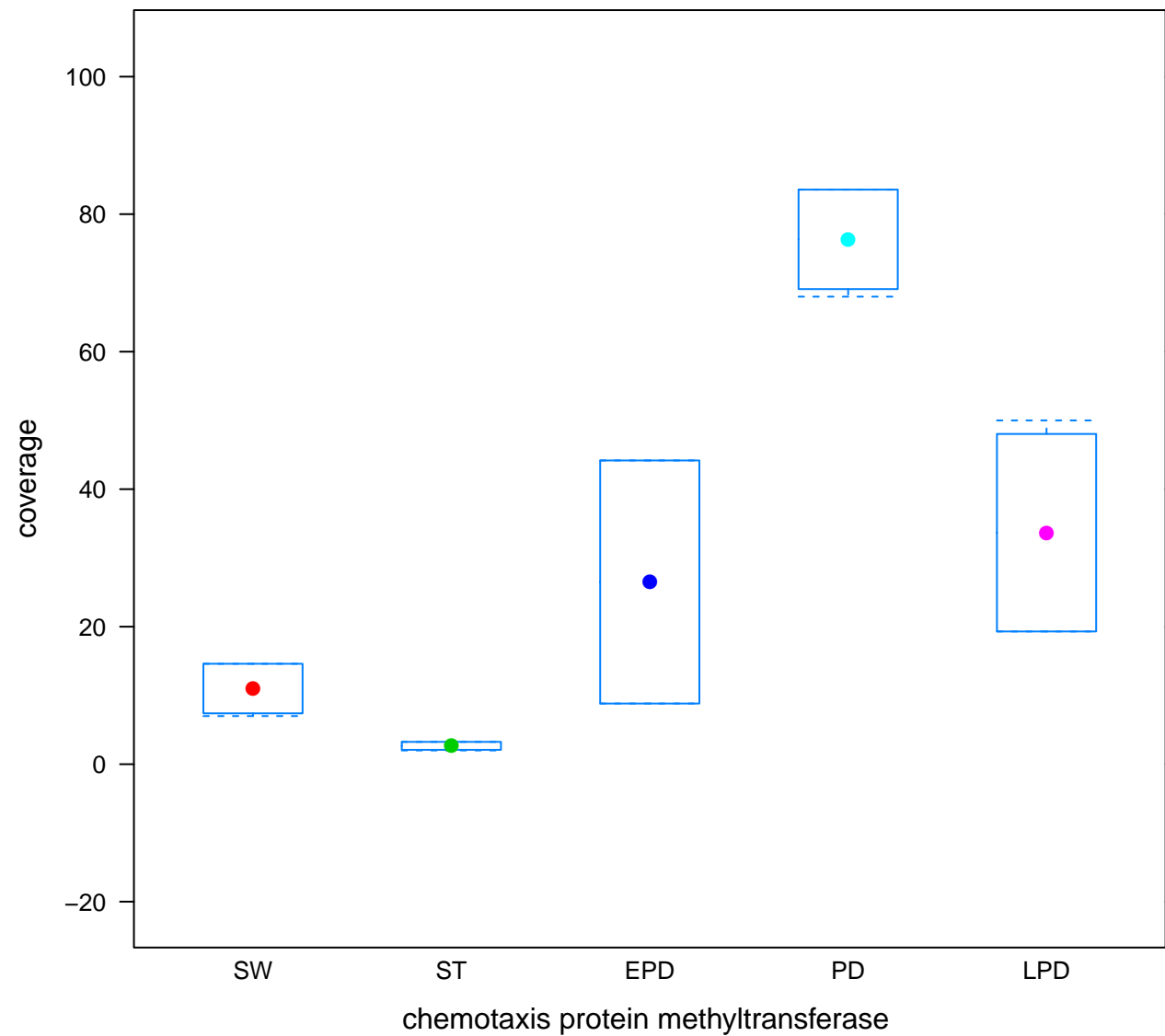

**Fold of change: 30.53**  
**baySeq likelihood: 0.634**

Supplement: Additional file 9: Figure S2 — Expression profiles of all identified CCR genes. [file 1471-2164-14-450-S9.zip › FigureS2/CCNA_00444.pdf]

# CCNA\_00445

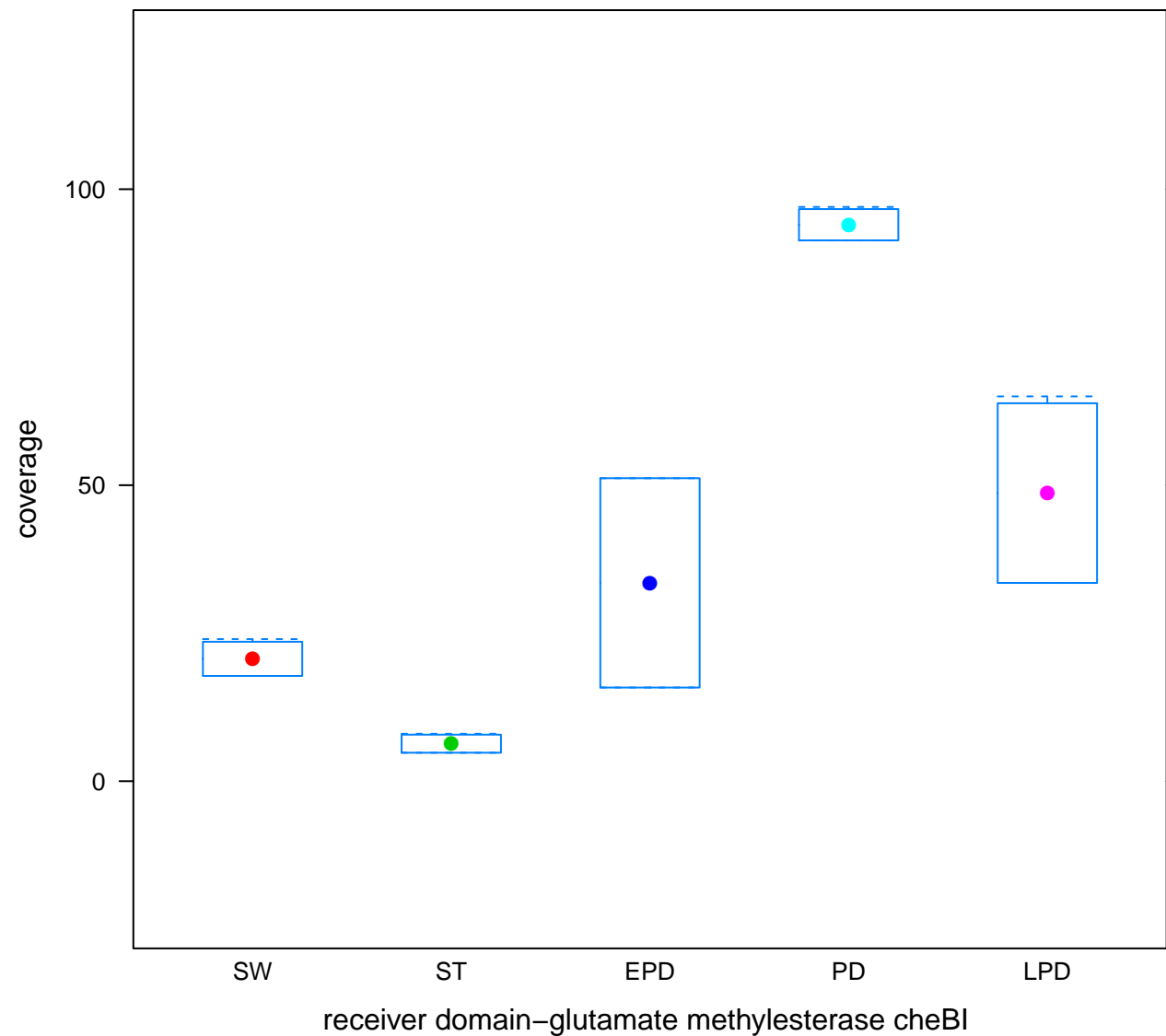

**Fold of change: 14.46**  
**baySeq likelihood: 0.869**

Supplement: Additional file 9: Figure S2 — Expression profiles of all identified CCR genes. [file 1471-2164-14-450-S9.zip › FigureS2/CCNA_00445.pdf]

CCNA\_00446

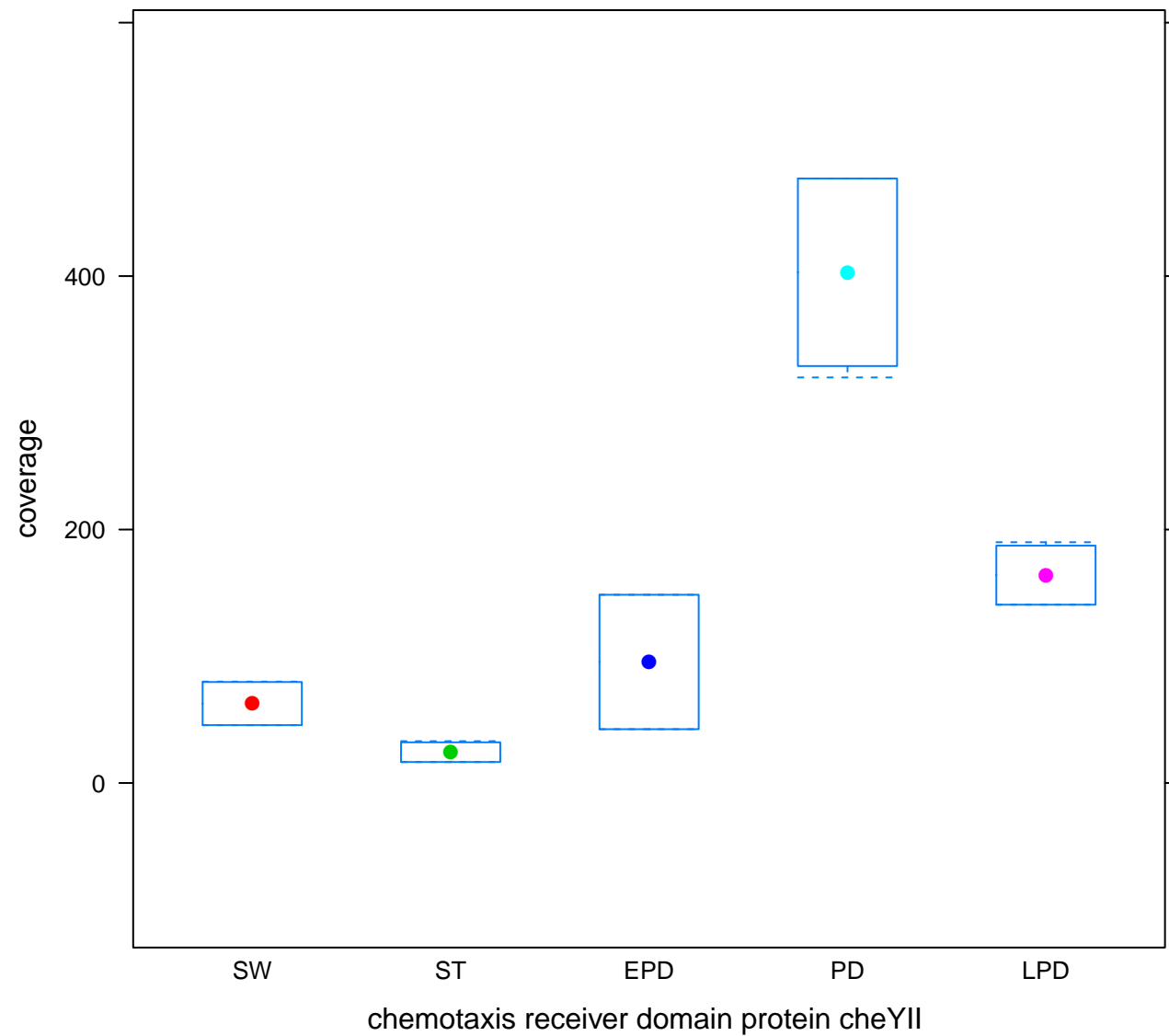

**Fold of change: 15.8**  
**baySeq likelihood: 0.604**

Supplement: Additional file 9: Figure S2 — Expression profiles of all identified CCR genes. [file 1471-2164-14-450-S9.zip › FigureS2/CCNA_00446.pdf]

# cheD;CCNA\_00447

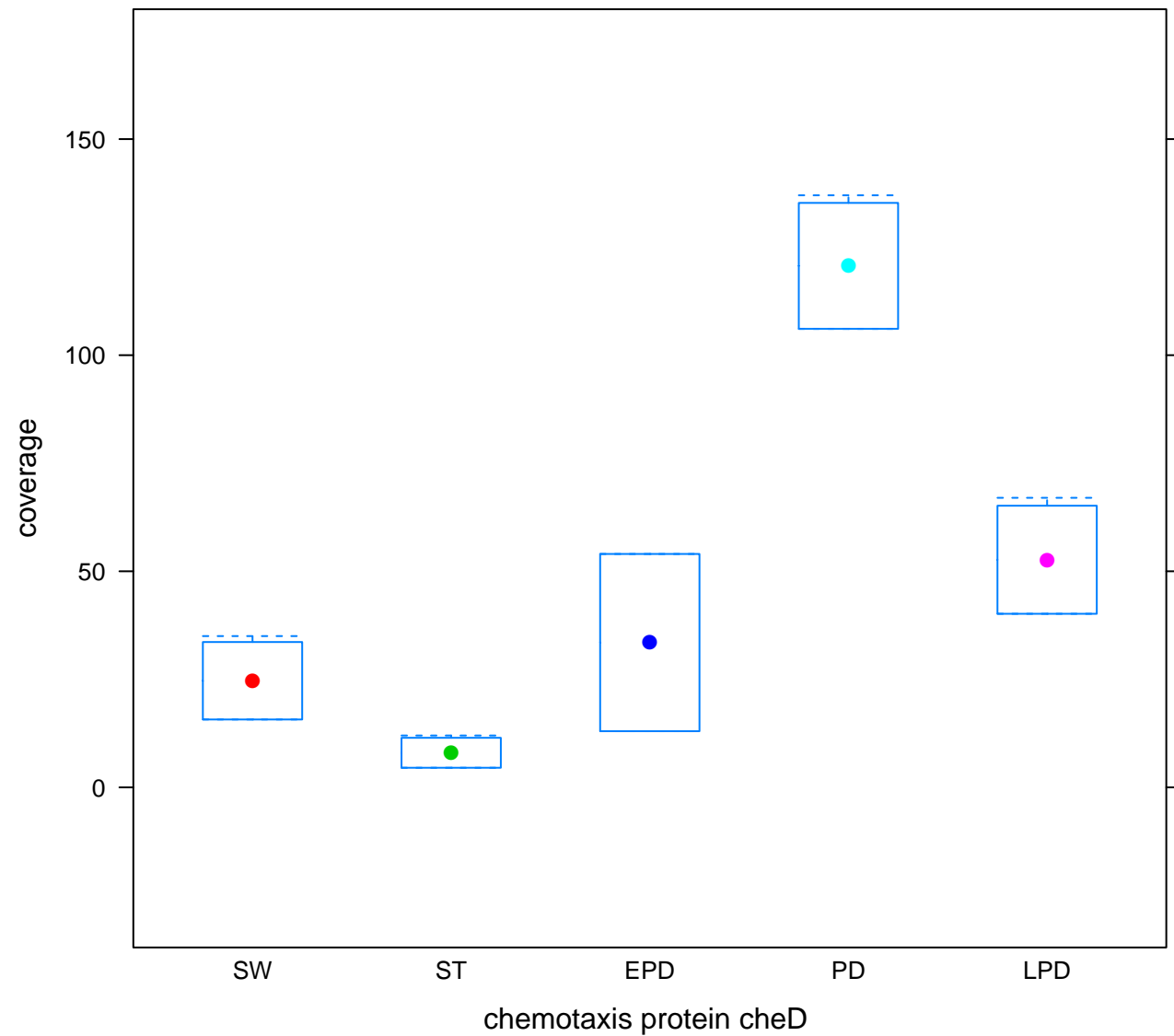

**Fold of change: 13.41**  
**baySeq likelihood: 0.64**

Supplement: Additional file 9: Figure S2 — Expression profiles of all identified CCR genes. [file 1471-2164-14-450-S9.zip › FigureS2/CCNA_00447.pdf]

# cheU;CCNA\_00448

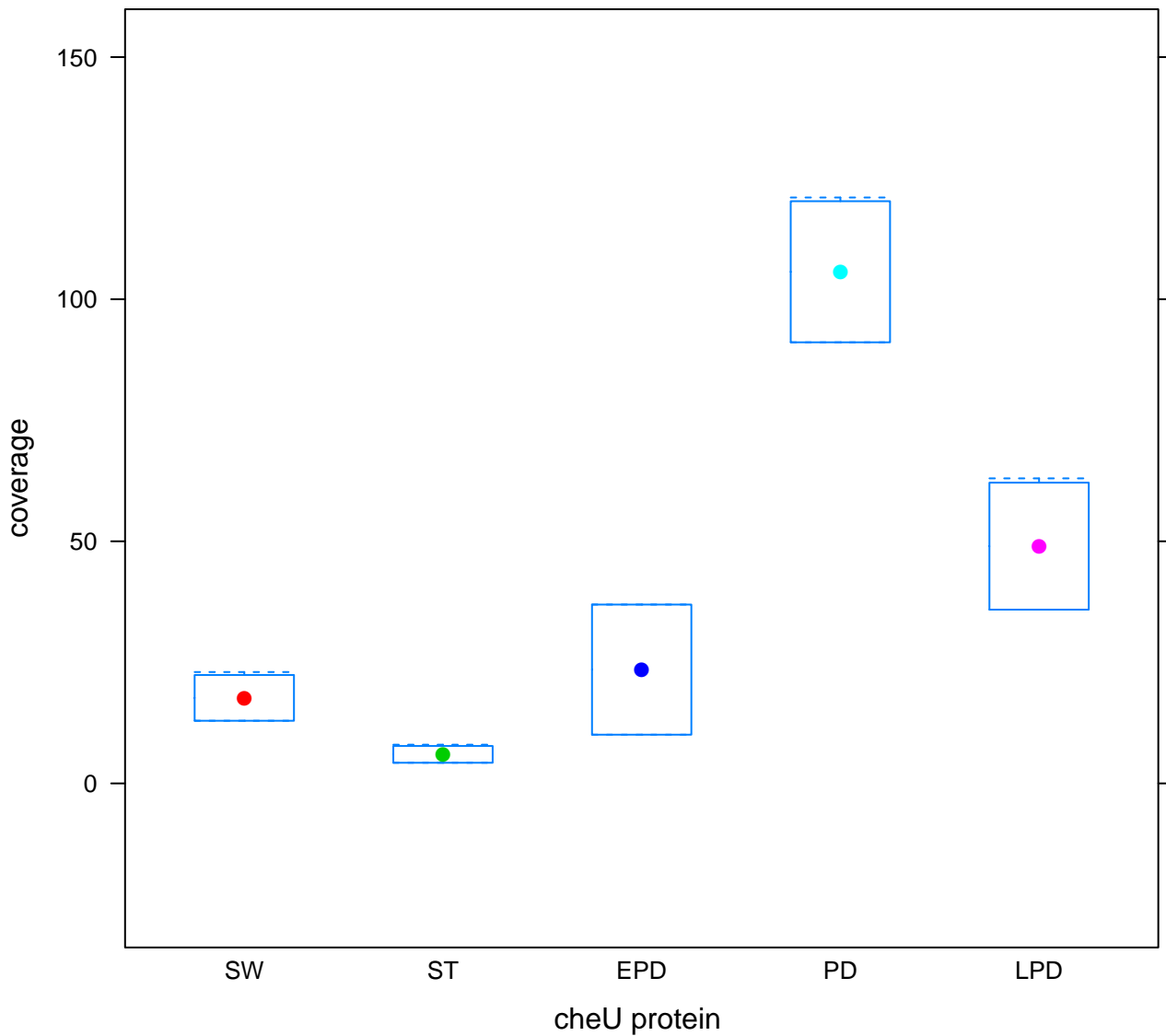

**Fold of change: 16.26**  
**baySeq likelihood: 0.512**

Supplement: Additional file 9: Figure S2 — Expression profiles of all identified CCR genes. [file 1471-2164-14-450-S9.zip › FigureS2/CCNA_00448.pdf]

# cheYIII;CCNA\_00449

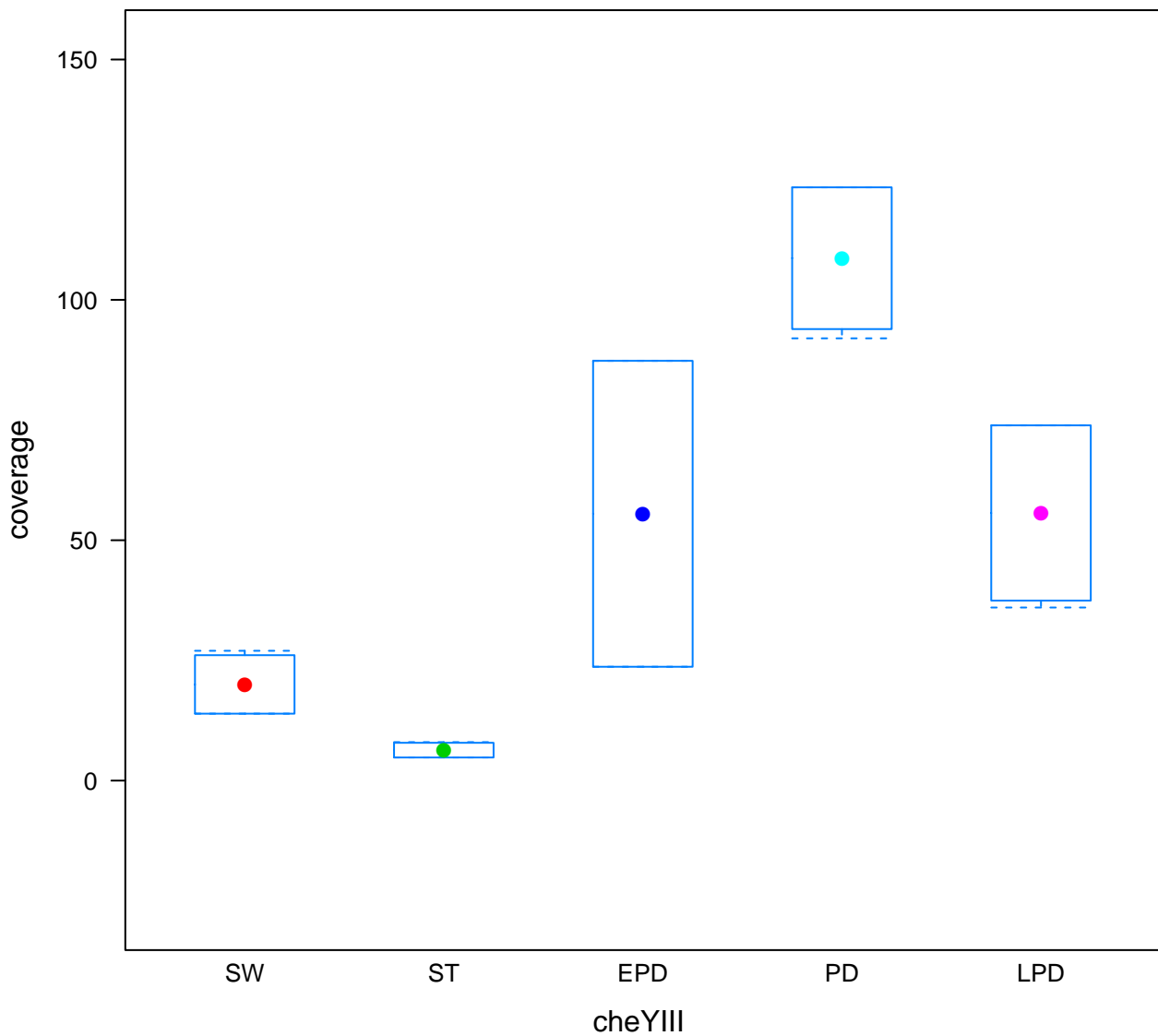

**Fold of change: 15.52**  
**baySeq likelihood: 0.628**

Supplement: Additional file 9: Figure S2 — Expression profiles of all identified CCR genes. [file 1471-2164-14-450-S9.zip › FigureS2/CCNA_00449.pdf]

# cheE;CCNA\_00450

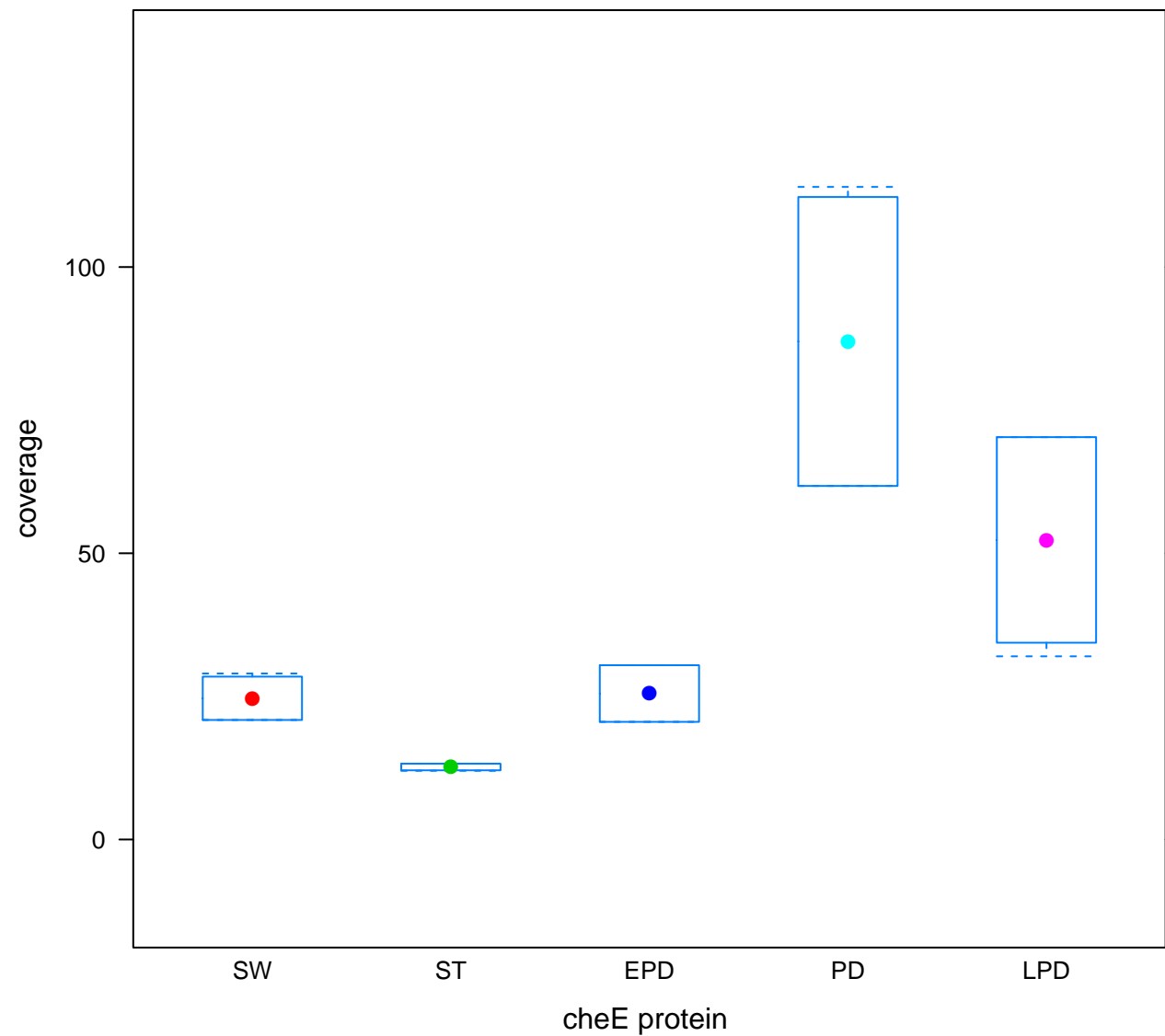

**Fold of change: 6.96**  
**baySeq likelihood: 0.815**

Supplement: Additional file 9: Figure S2 — Expression profiles of all identified CCR genes. [file 1471-2164-14-450-S9.zip › FigureS2/CCNA_00450.pdf]

# CCNA\_00451

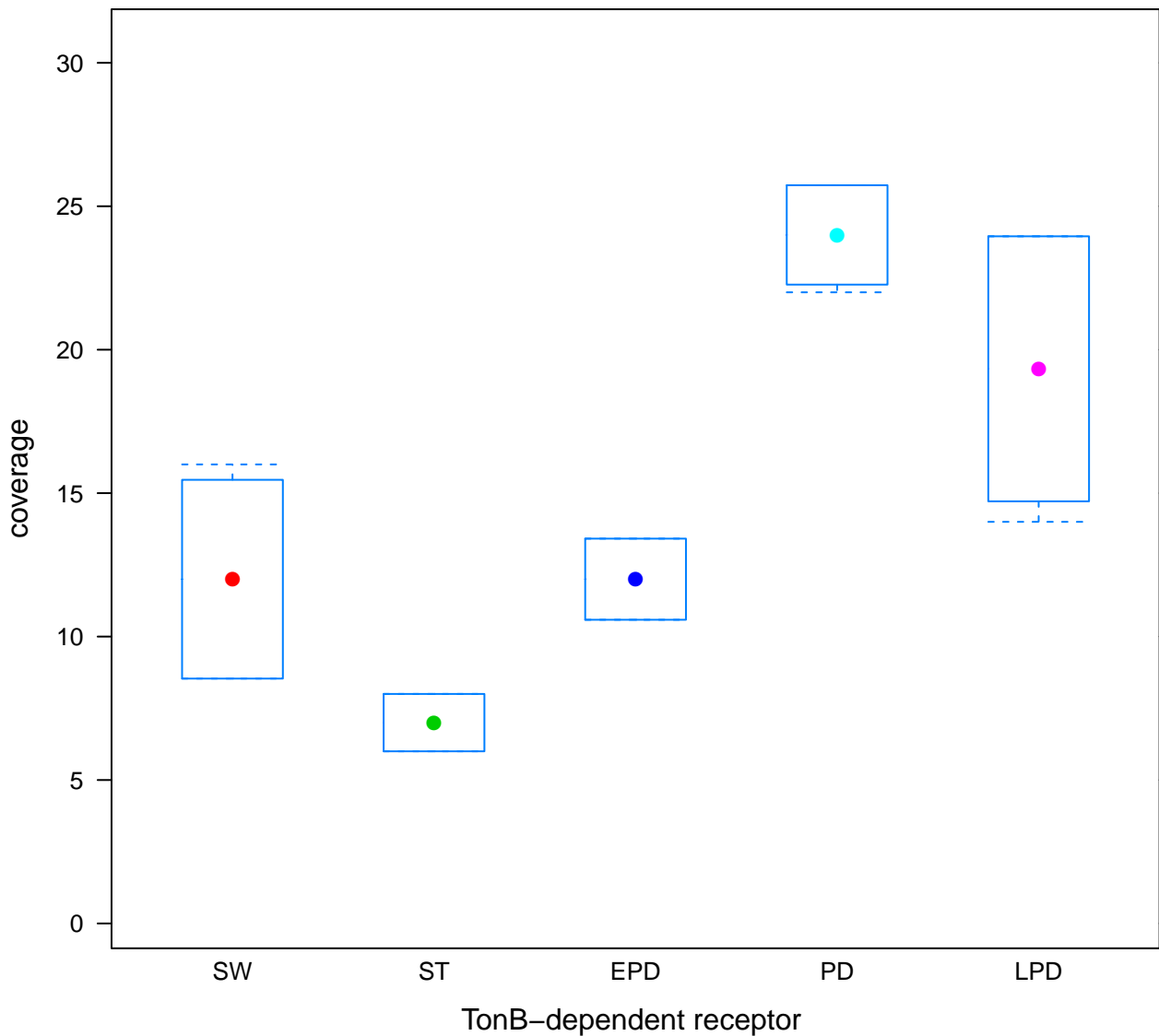

**Fold of change: 3.43**  
**baySeq likelihood: 0.821**

Supplement: Additional file 9: Figure S2 — Expression profiles of all identified CCR genes. [file 1471-2164-14-450-S9.zip › FigureS2/CCNA_00451.pdf]

# nagA;CCNA\_00455

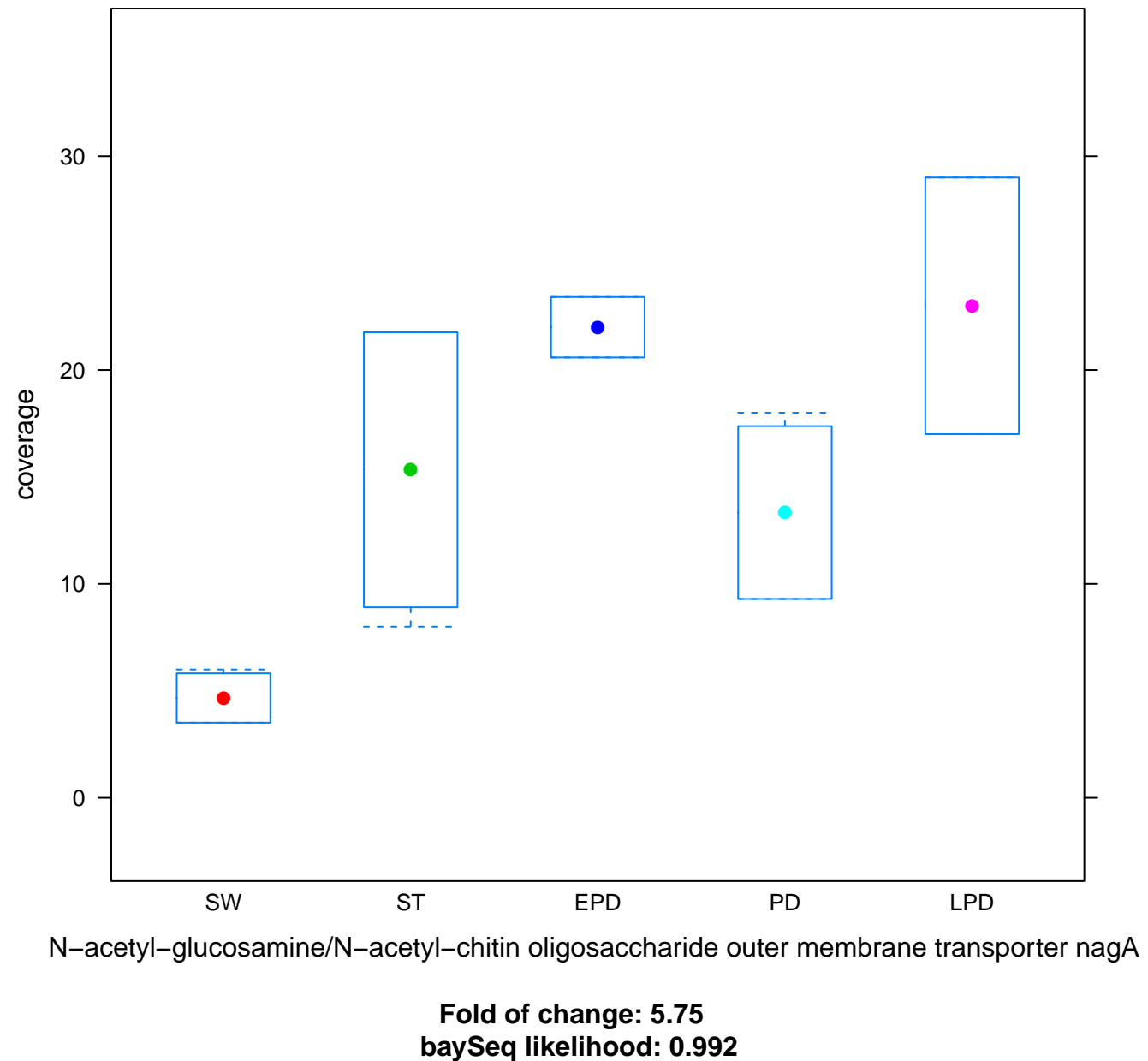

Supplement: Additional file 9: Figure S2 — Expression profiles of all identified CCR genes. [file 1471-2164-14-450-S9.zip › FigureS2/CCNA_00455.pdf]

# CCNA\_00464

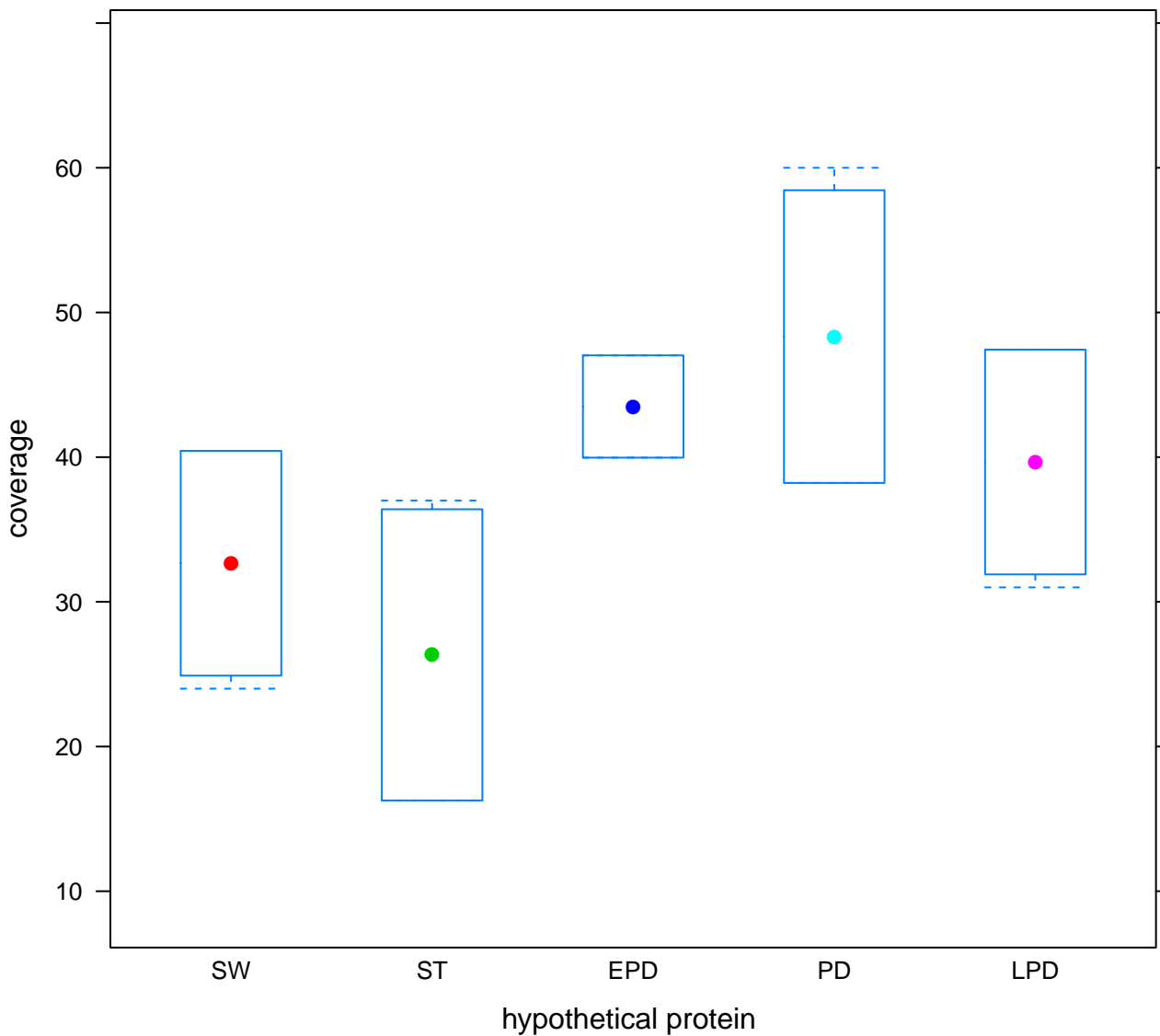

**Fold of change: 2.3**  
**baySeq likelihood: 0.256**

Supplement: Additional file 9: Figure S2 — Expression profiles of all identified CCR genes. [file 1471-2164-14-450-S9.zip › FigureS2/CCNA_00464.pdf]

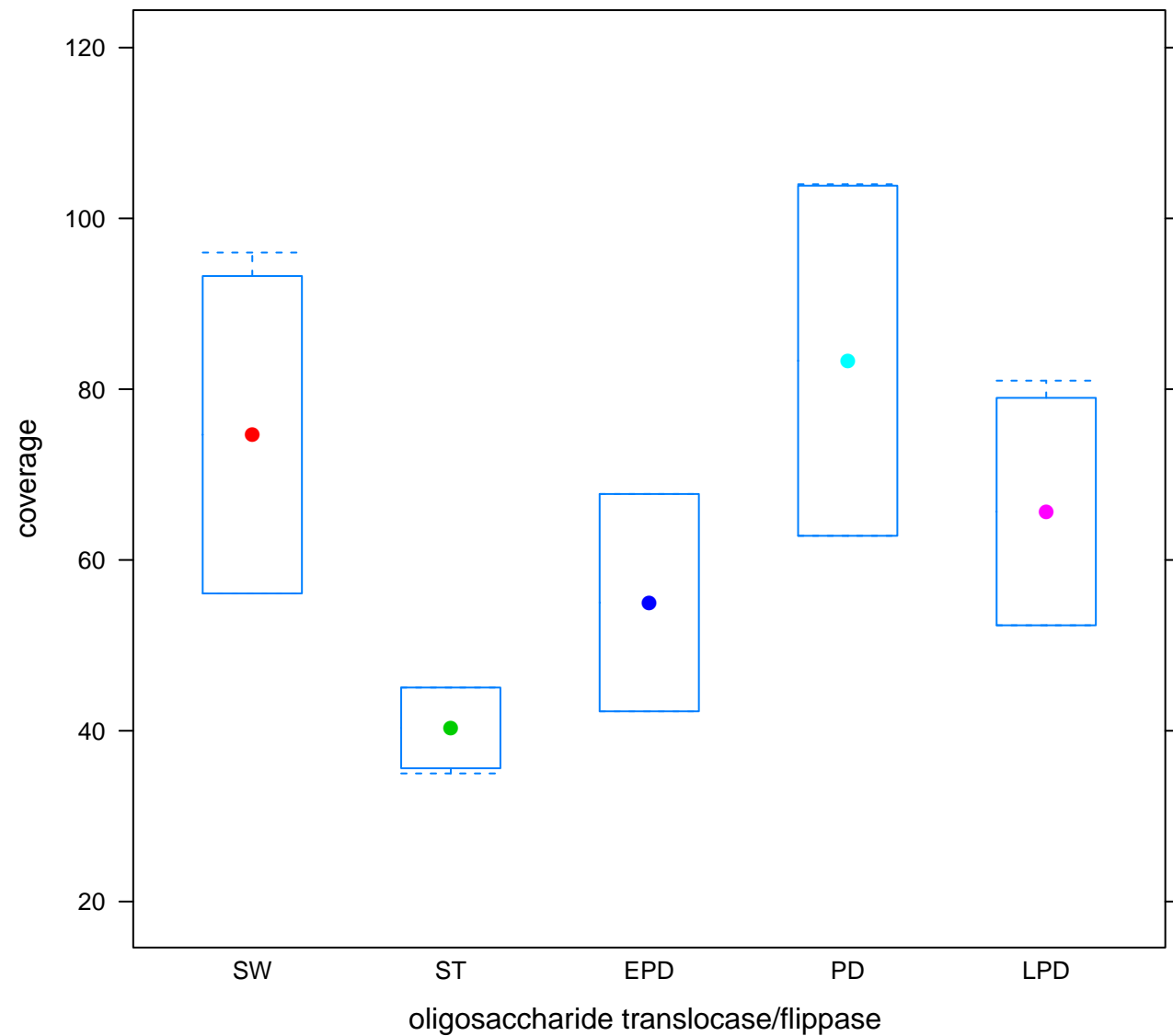

**Fold of change: 2.16**  
**baySeq likelihood: 0.367**

Supplement: Additional file 9: Figure S2 — Expression profiles of all identified CCR genes. [file 1471-2164-14-450-S9.zip › FigureS2/CCNA_00467.pdf]

# CCNA\_00468

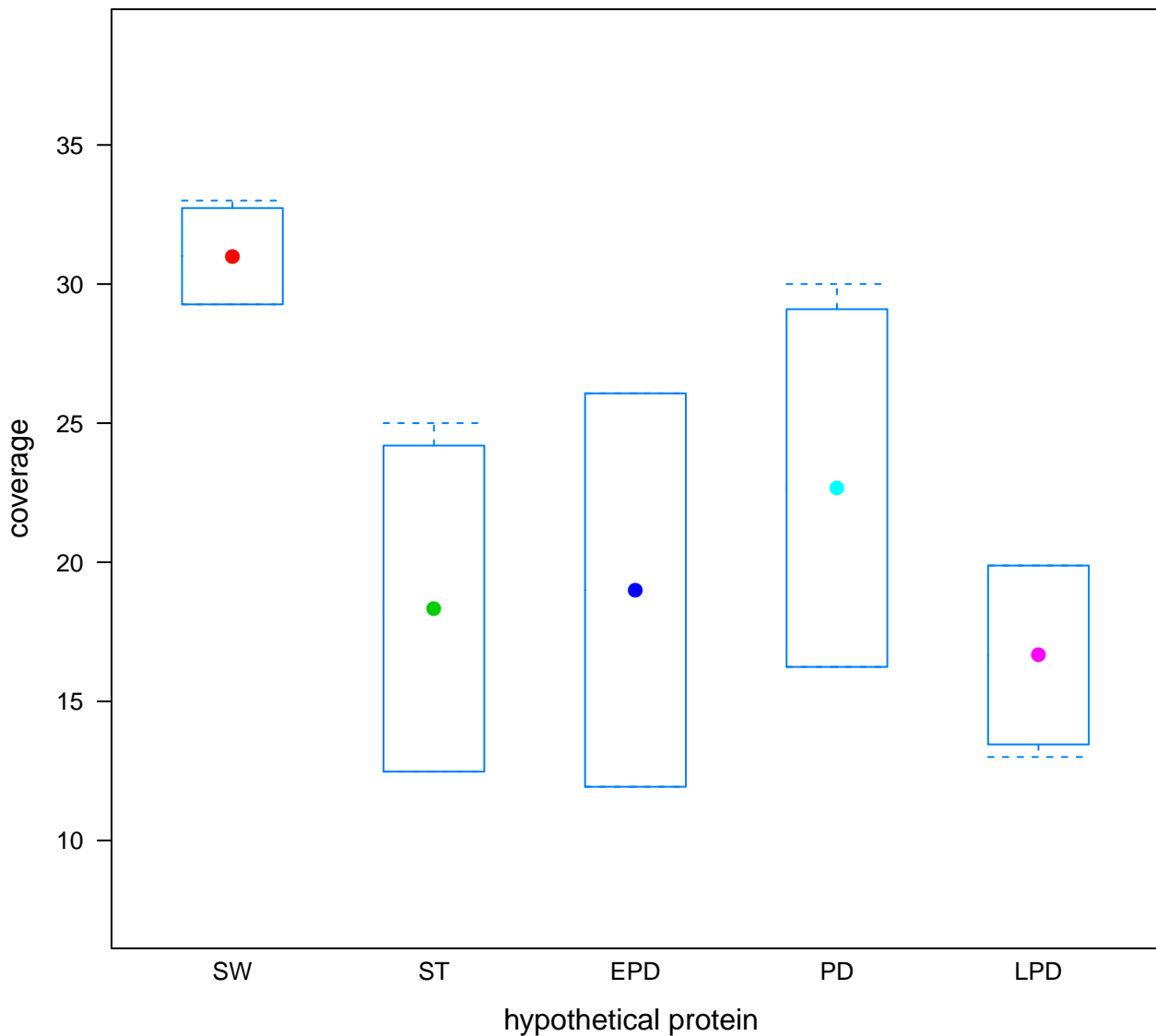

**Fold of change: 2**  
**baySeq likelihood: 0.897**

Supplement: Additional file 9: Figure S2 — Expression profiles of all identified CCR genes. [file 1471-2164-14-450-S9.zip › FigureS2/CCNA_00468.pdf]

# CCNA\_00470

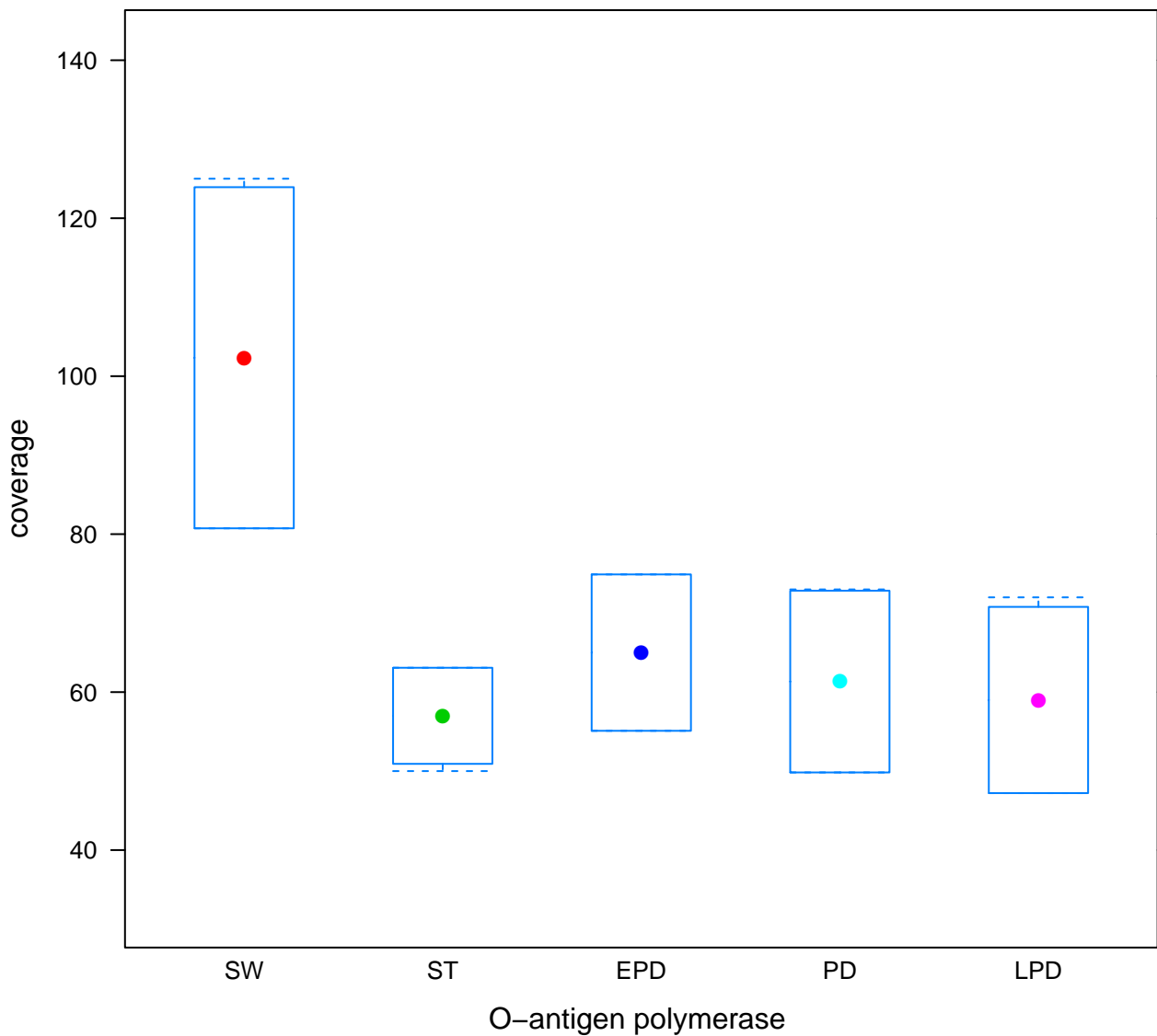

**Fold of change: 1.75**  
**baySeq likelihood: 0.985**

Supplement: Additional file 9: Figure S2 — Expression profiles of all identified CCR genes. [file 1471-2164-14-450-S9.zip › FigureS2/CCNA_00470.pdf]

# CCNA\_00471

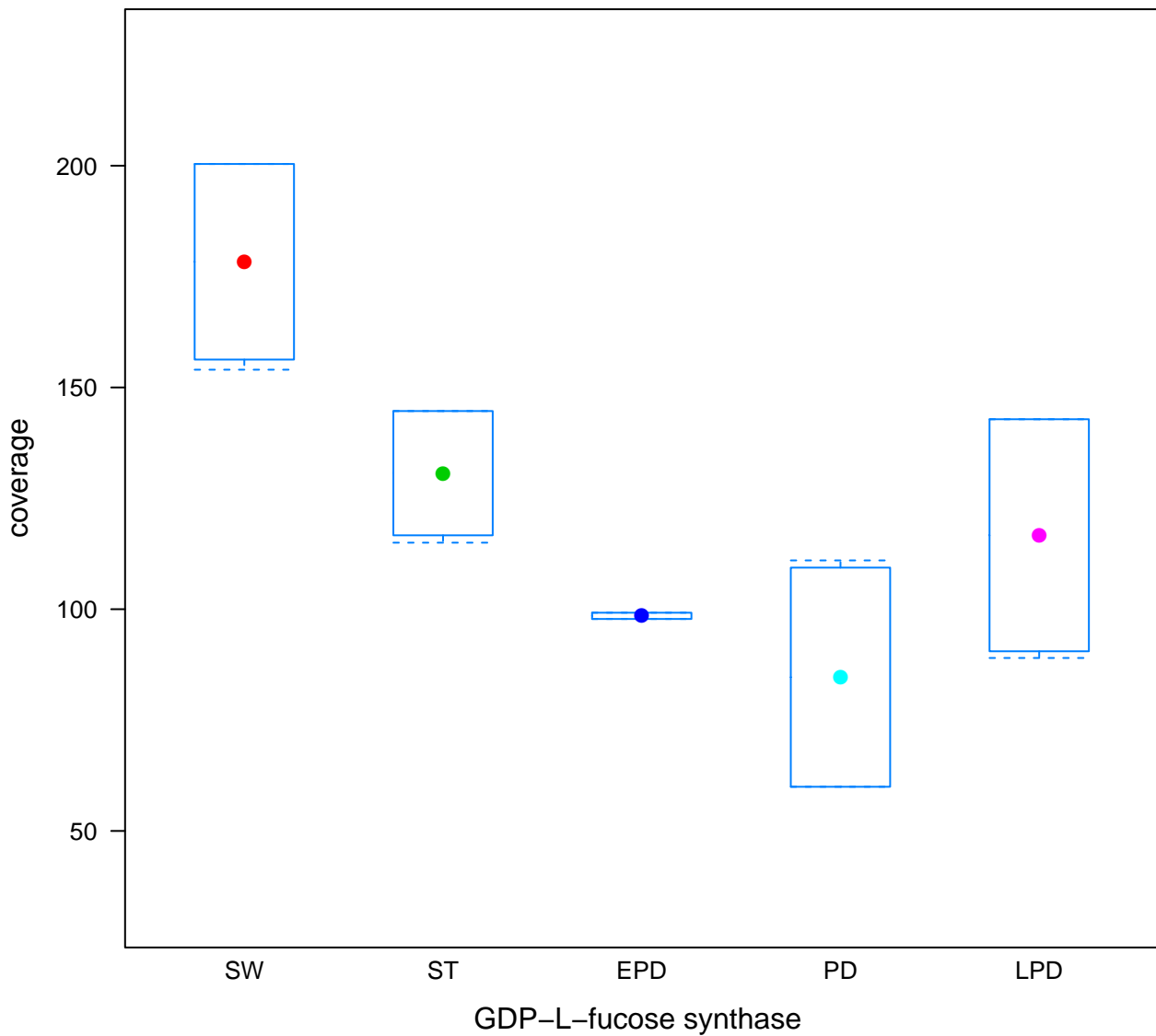

**Fold of change: 2.07**  
**baySeq likelihood: 0.714**

Supplement: Additional file 9: Figure S2 — Expression profiles of all identified CCR genes. [file 1471-2164-14-450-S9.zip › FigureS2/CCNA_00471.pdf]

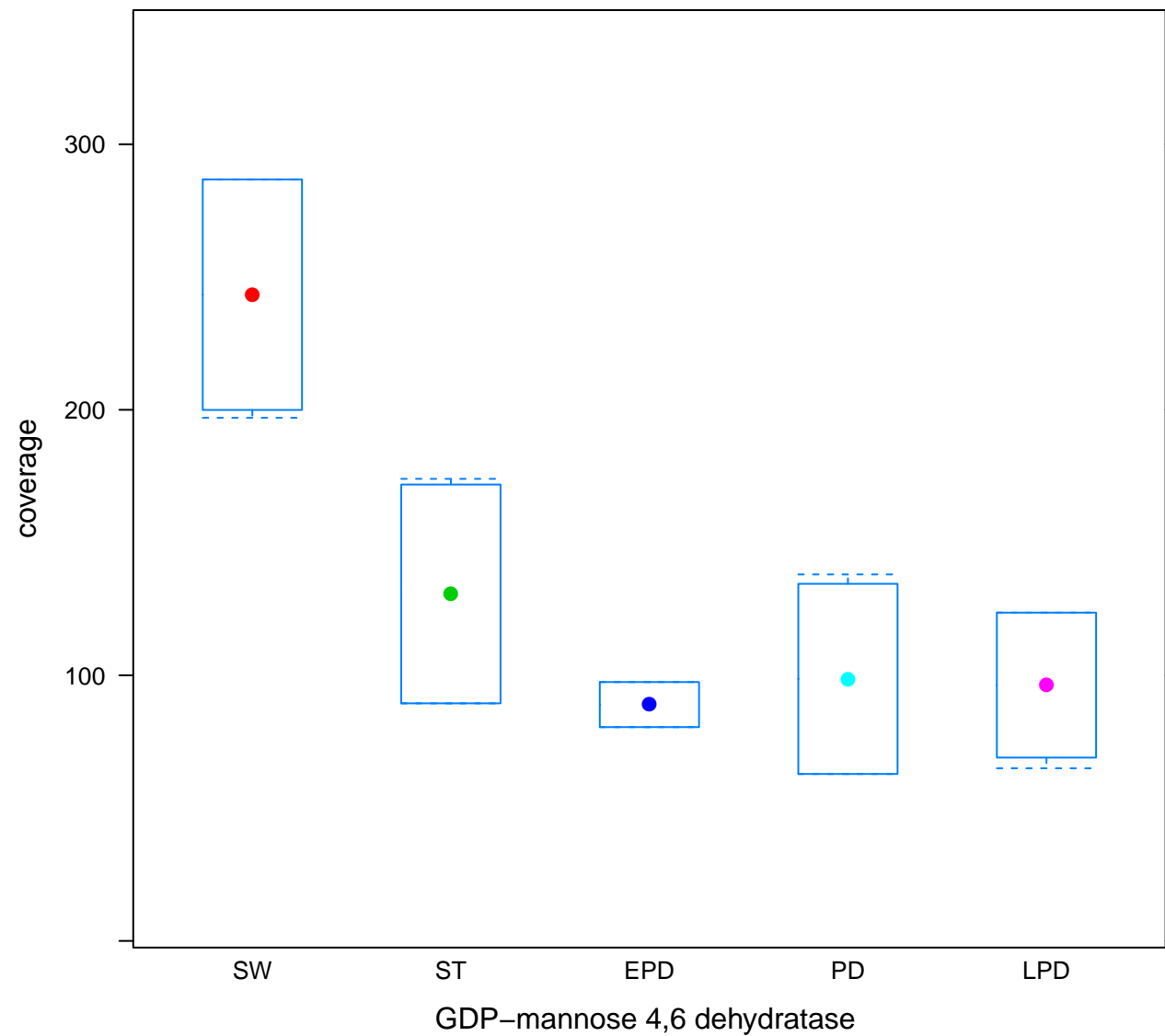

**Fold of change: 2.7**  
**baySeq likelihood: 0.974**

Supplement: Additional file 9: Figure S2 — Expression profiles of all identified CCR genes. [file 1471-2164-14-450-S9.zip › FigureS2/CCNA_00472.pdf]

# CCNA\_00481

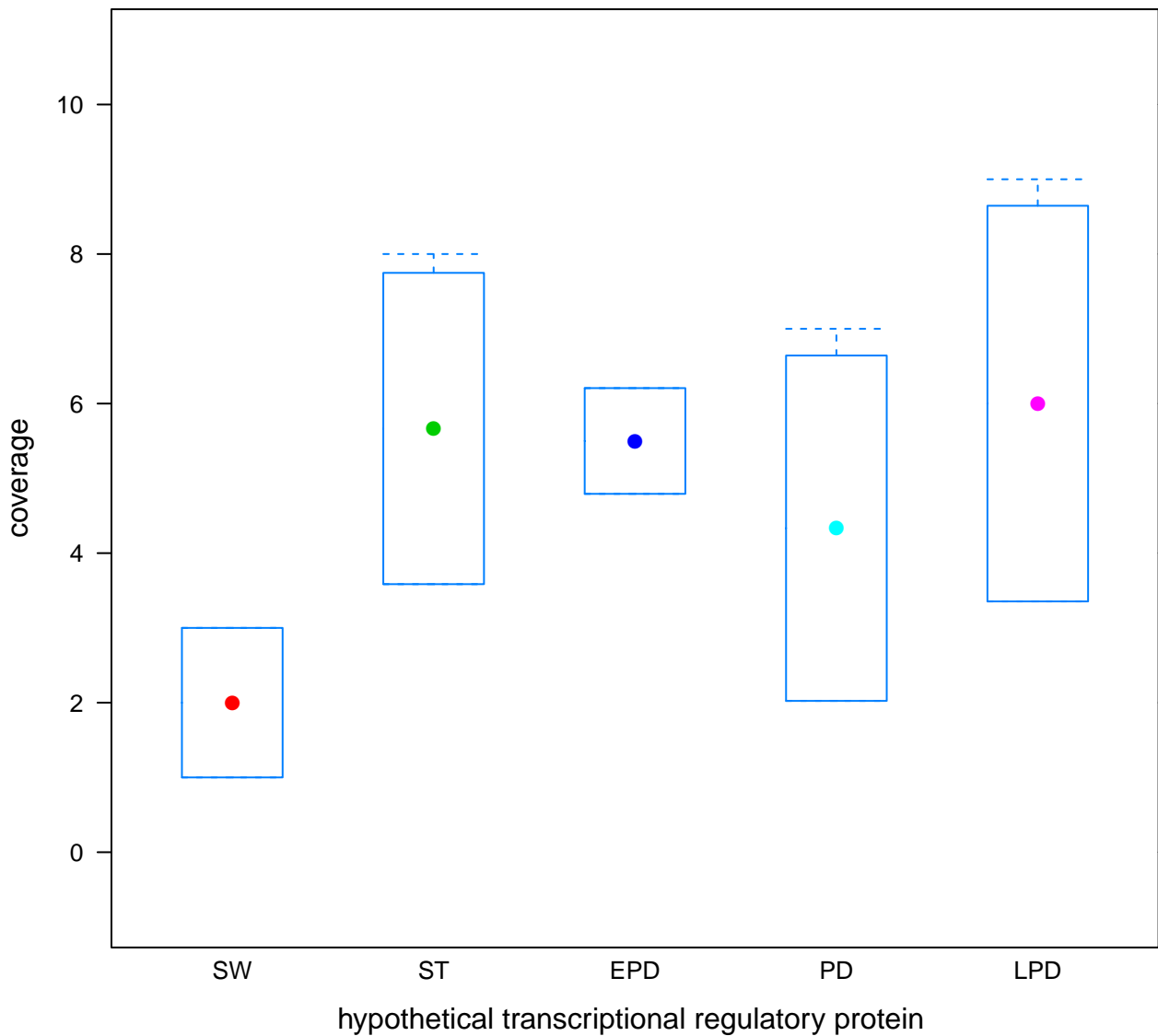

**Fold of change: 4**  
**baySeq likelihood: 0.404**

Supplement: Additional file 9: Figure S2 — Expression profiles of all identified CCR genes. [file 1471-2164-14-450-S9.zip › FigureS2/CCNA_00481.pdf]

# CCNA\_00482

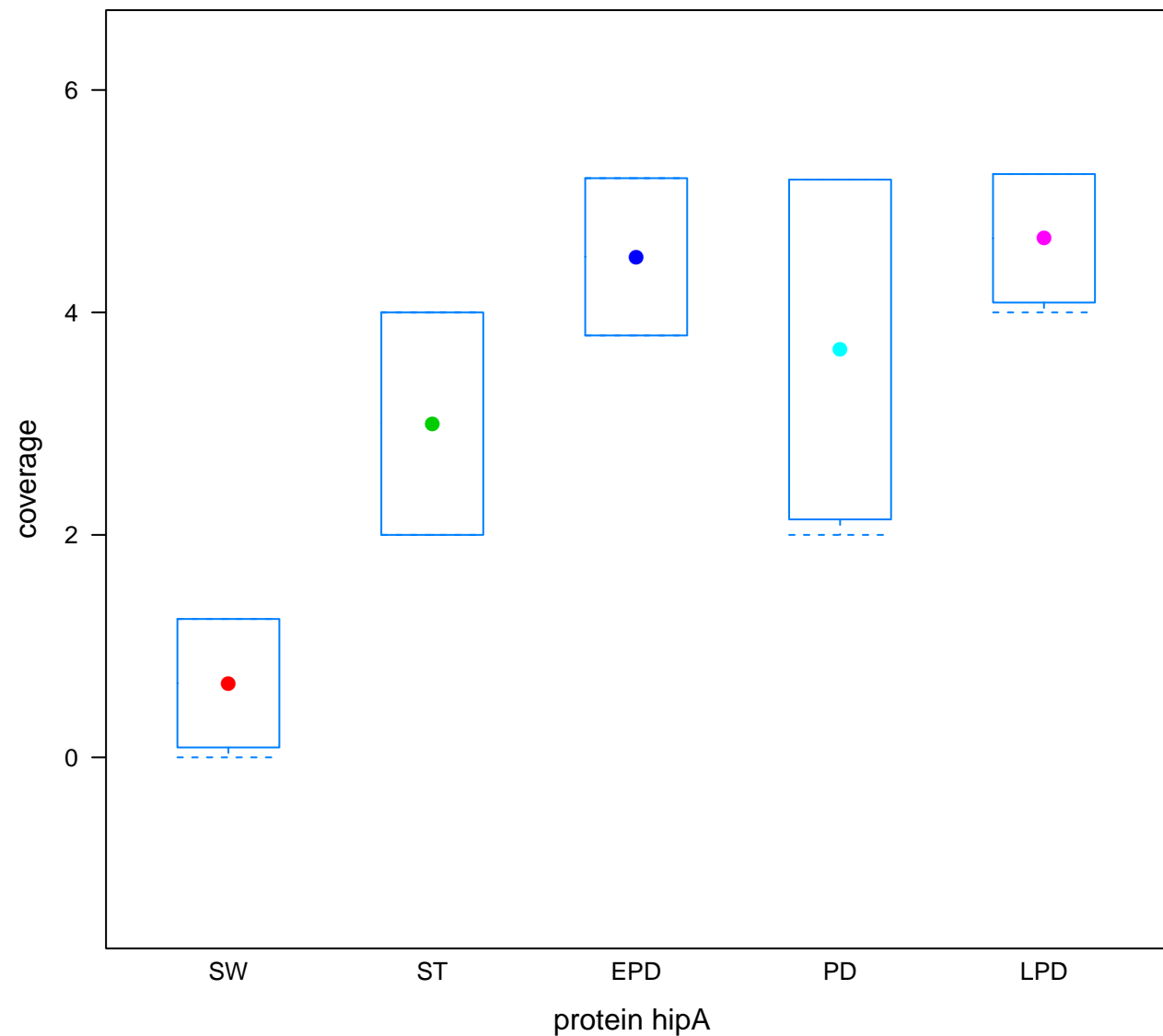

**Fold of change: 9.33**  
**baySeq likelihood: 0.772**

Supplement: Additional file 9: Figure S2 — Expression profiles of all identified CCR genes. [file 1471-2164-14-450-S9.zip › FigureS2/CCNA_00482.pdf]

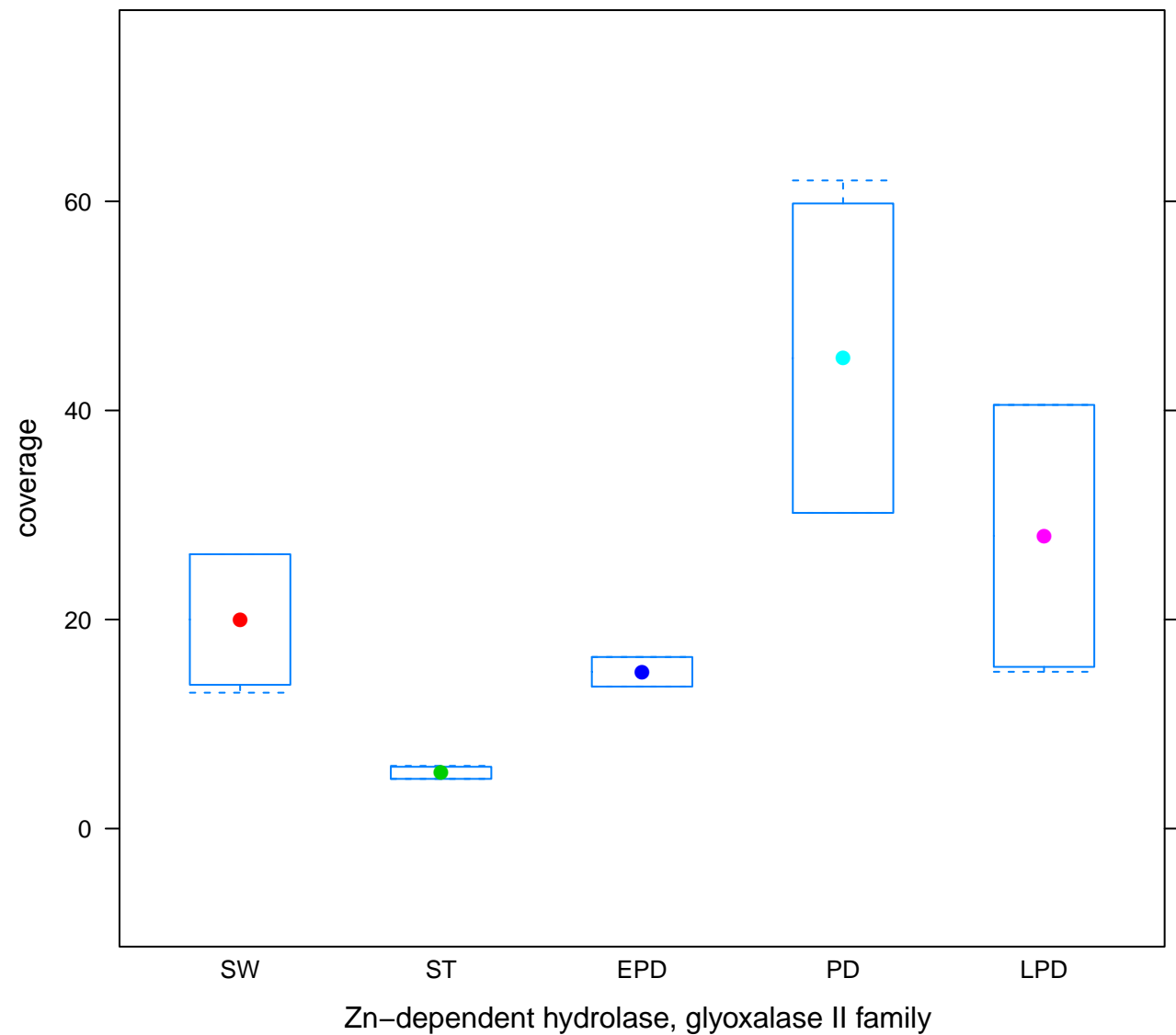

**Fold of change: 9**  
**baySeq likelihood: 0.897**

Supplement: Additional file 9: Figure S2 — Expression profiles of all identified CCR genes. [file 1471-2164-14-450-S9.zip › FigureS2/CCNA_00483.pdf]

# CCNA\_00489

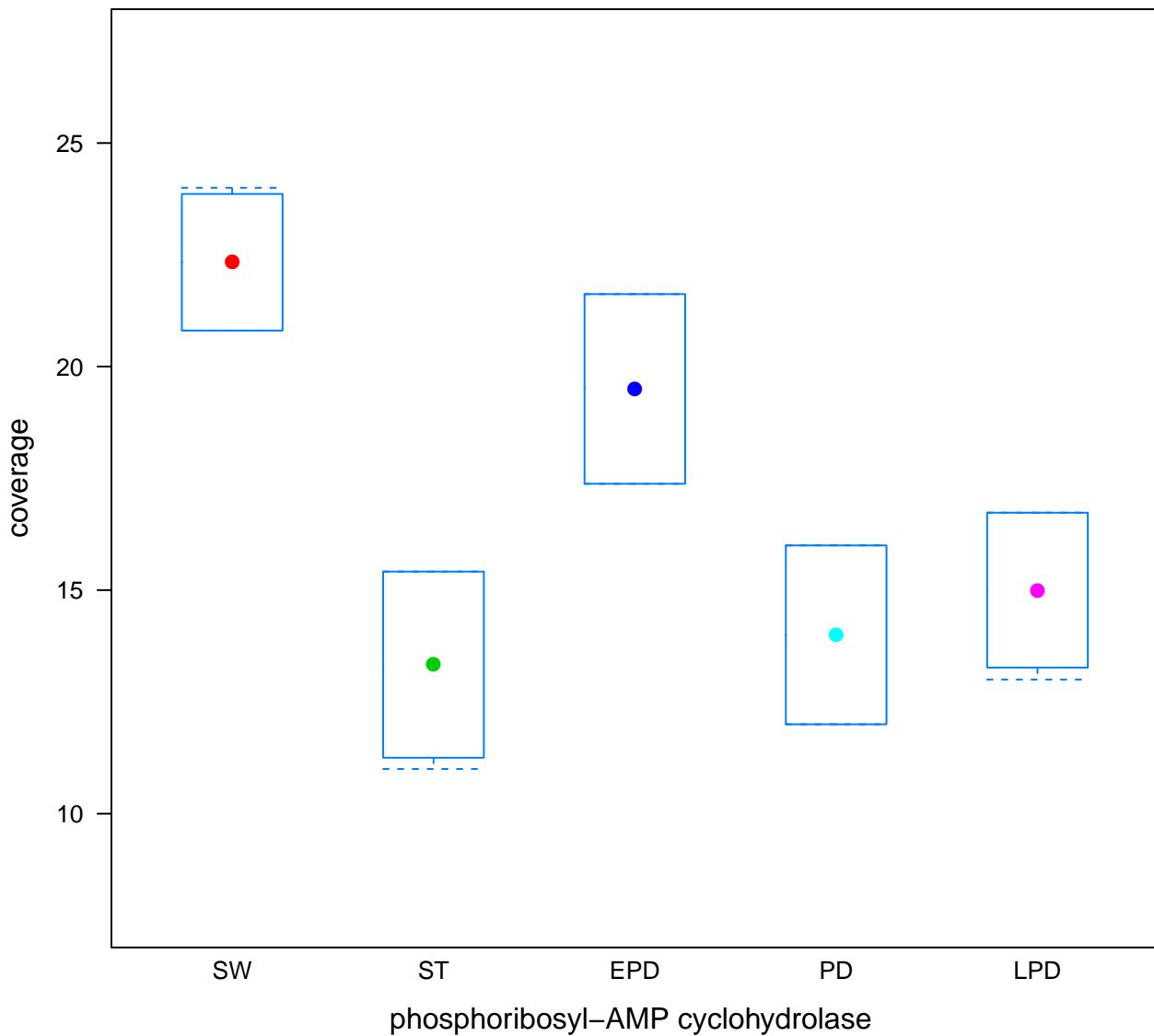

**Fold of change: 1.64**  
**baySeq likelihood: 0.512**

Supplement: Additional file 9: Figure S2 — Expression profiles of all identified CCR genes. [file 1471-2164-14-450-S9.zip › FigureS2/CCNA_00489.pdf]

# CCNA\_00491

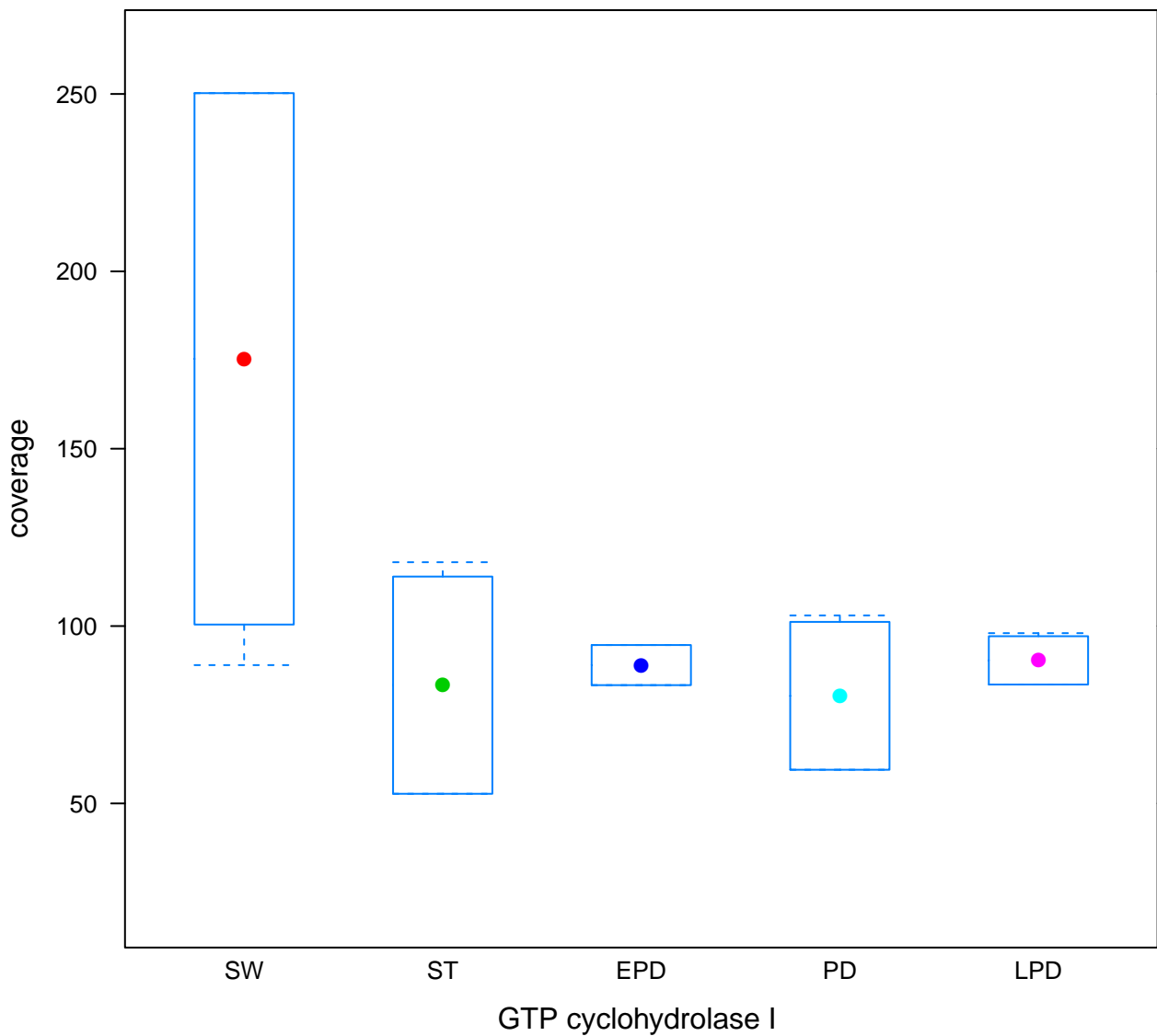

**Fold of change: 2.36**  
**baySeq likelihood: 0.873**

Supplement: Additional file 9: Figure S2 — Expression profiles of all identified CCR genes. [file 1471-2164-14-450-S9.zip › FigureS2/CCNA_00491.pdf]

# CCNA\_00492

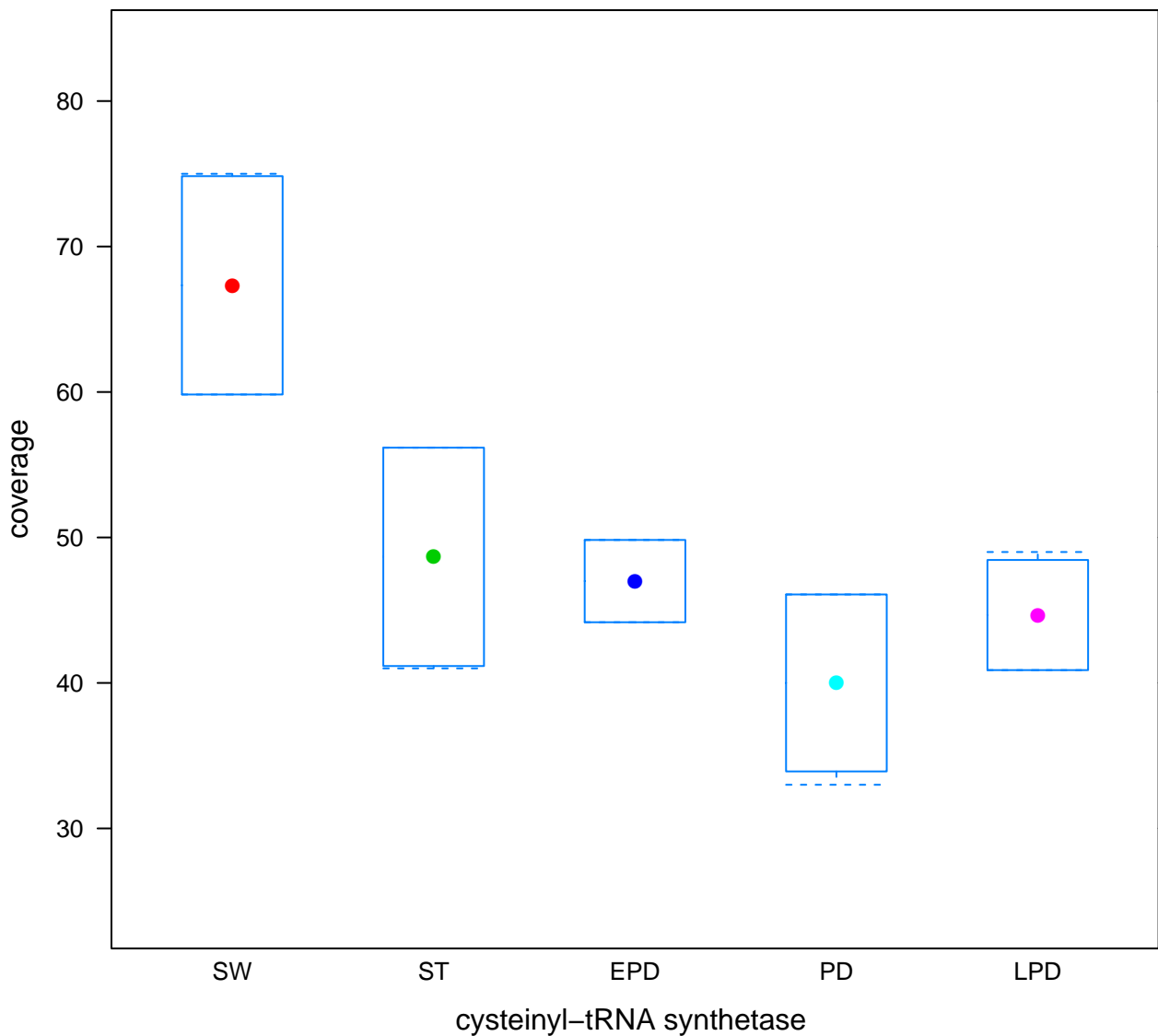

**Fold of change: 1.77**  
**baySeq likelihood: 0.965**

Supplement: Additional file 9: Figure S2 — Expression profiles of all identified CCR genes. [file 1471-2164-14-450-S9.zip › FigureS2/CCNA_00492.pdf]

# CCNA\_00493

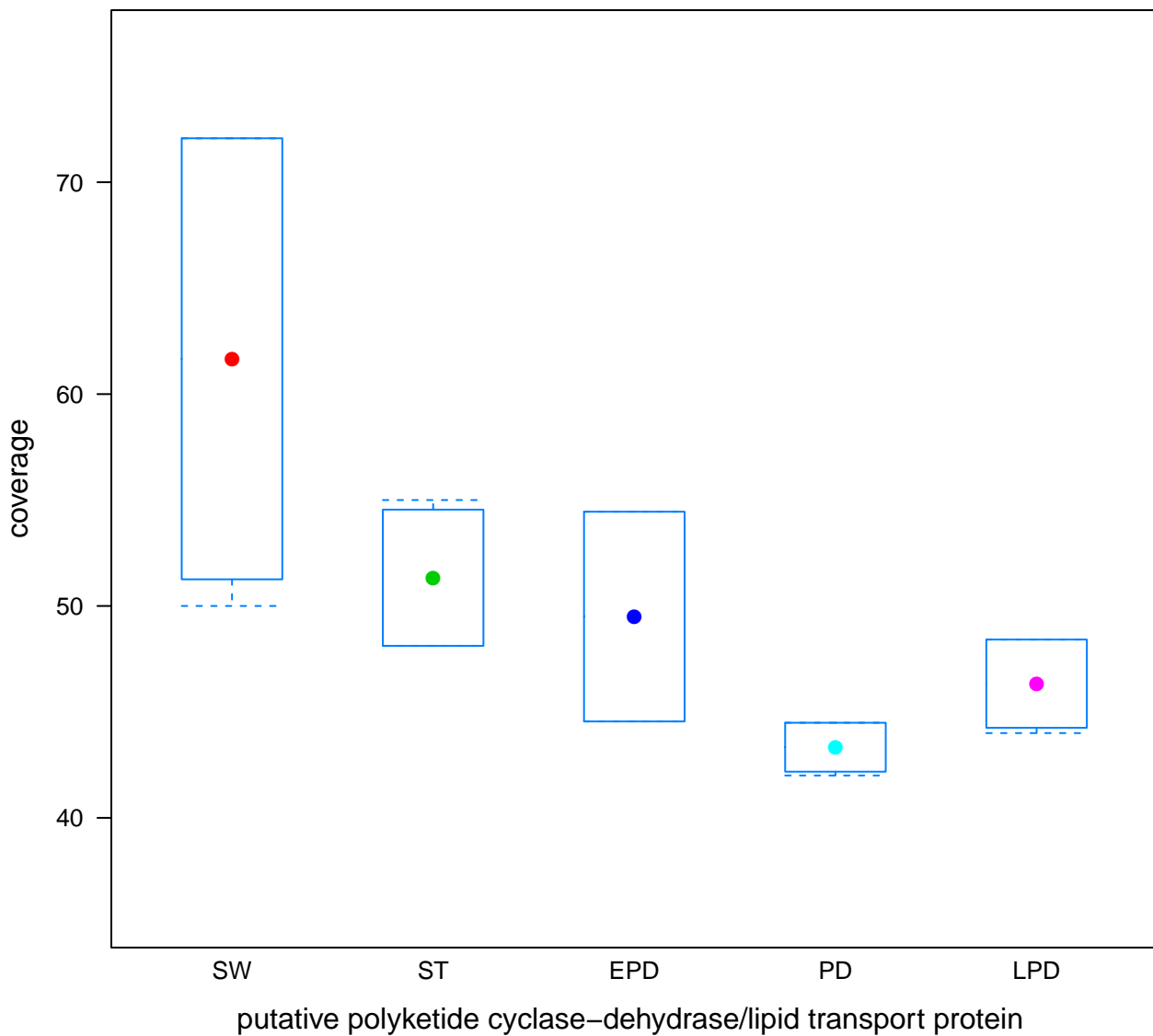

**Fold of change: 1.56**  
**baySeq likelihood: 0.66**

Supplement: Additional file 9: Figure S2 — Expression profiles of all identified CCR genes. [file 1471-2164-14-450-S9.zip › FigureS2/CCNA_00493.pdf]

# CCNA\_00494

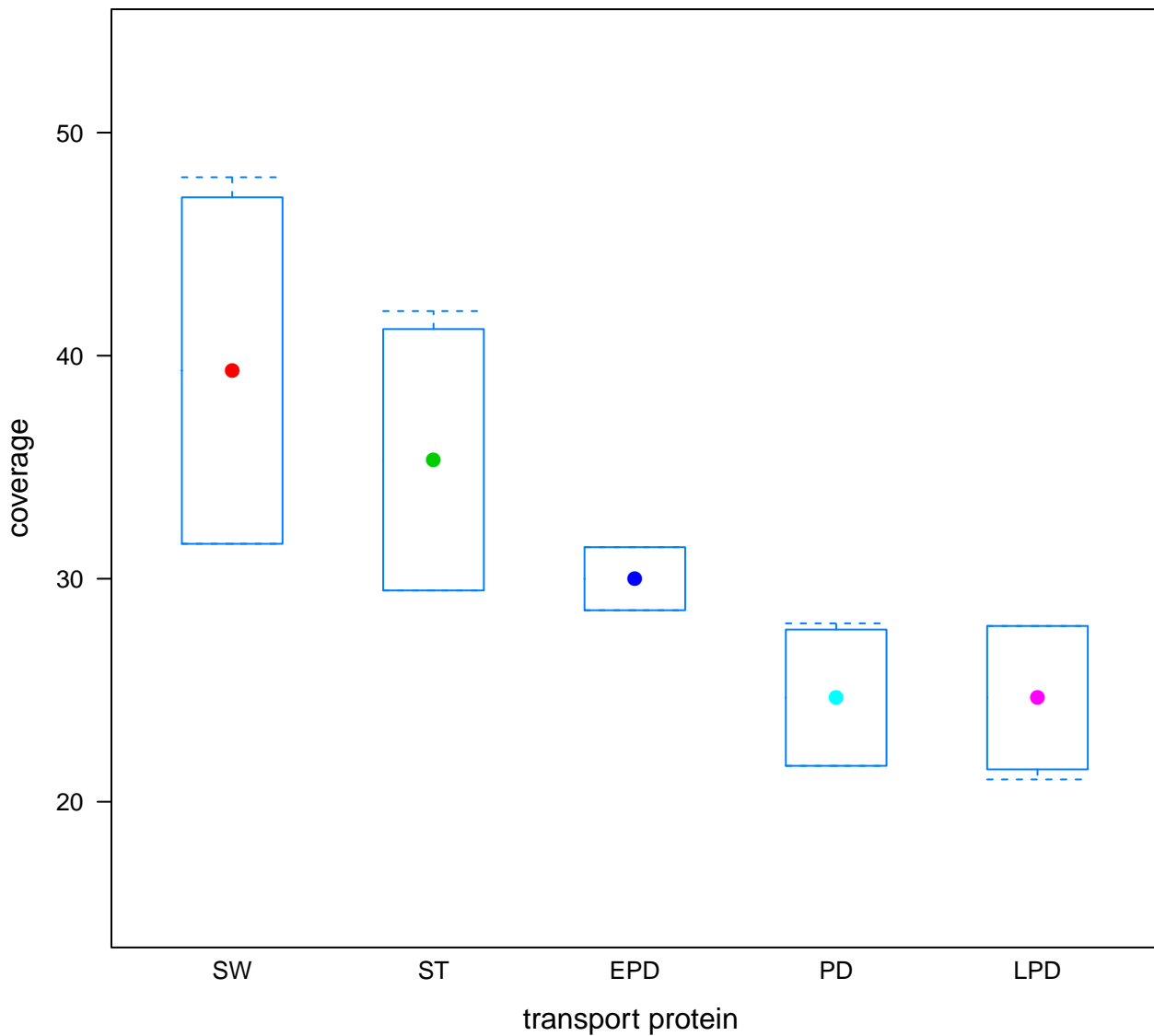

**Fold of change: 1.64**  
**baySeq likelihood: 0.524**

Supplement: Additional file 9: Figure S2 — Expression profiles of all identified CCR genes. [file 1471-2164-14-450-S9.zip › FigureS2/CCNA_00494.pdf]
