# Supplementary material for: Transcriptomic and phylogenetic analysis of a bacterial cell cycle reveals strong associations between gene co-expression and evolution
Source: BMC Genomics. 2013 Jul 5;14:450. doi: 10.1186/1471-2164-14-450 (PMC3829707; doi:10.1186/1471-2164-14-450)
Supplement: Additional file 13: Figure S4 — Co-expression network topologies of all 76 modules. [file 1471-2164-14-450-S13.zip › FigureS4/antiquewhite4.pdf]

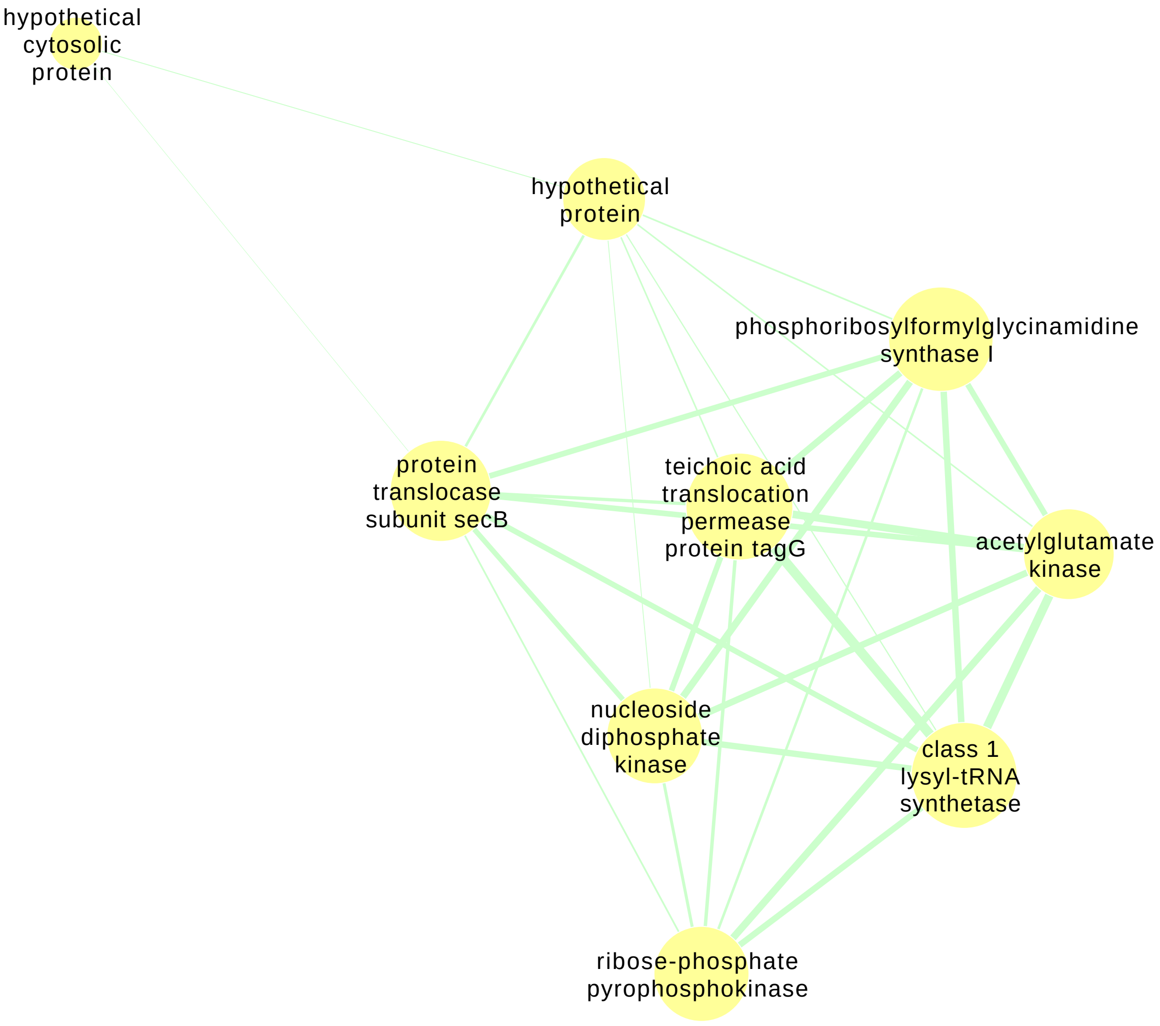
hypothetical cytosolic protein
hypothetical protein
phosphoribosylformylglycinamidine synthase I
protein translocase subunit secB
teichoic acid translocation permease protein tagG
acetylglutamate kinase
nucleoside diphosphate kinase
class 1 lysyl-tRNA synthetase
ribose-phosphate pyrophosphokinase
