# Supplementary material for: Transcriptomic and phylogenetic analysis of a bacterial cell cycle reveals strong associations between gene co-expression and evolution
Source: BMC Genomics. 2013 Jul 5;14:450. doi: 10.1186/1471-2164-14-450 (PMC3829707; doi:10.1186/1471-2164-14-450)
Supplement: Additional file 17: Figure S7 — Persistent index distributions. [file 1471-2164-14-450-S17.pdf]

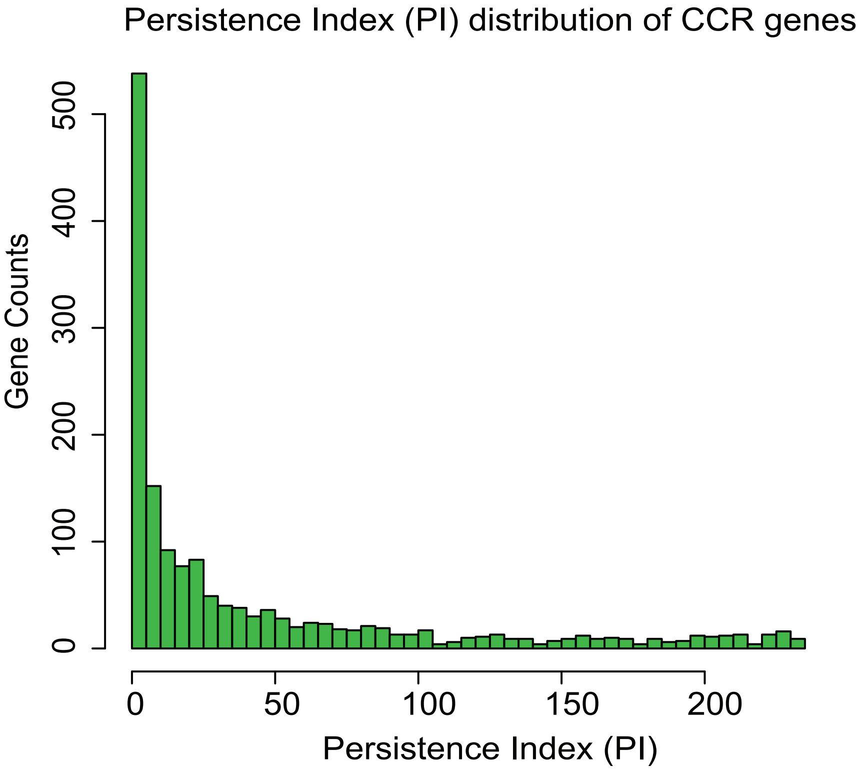

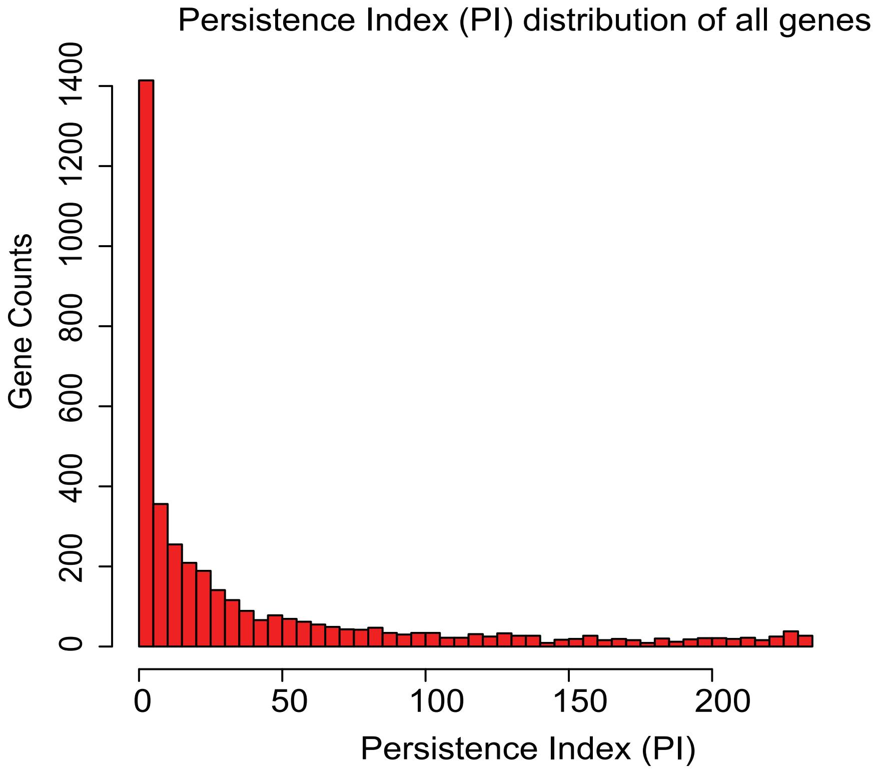

t-test: no difference between all and CCR genes in terms of PI
