# Supplementary material for: Transcriptomic and phylogenetic analysis of a bacterial cell cycle reveals strong associations between gene co-expression and evolution
Source: BMC Genomics. 2013 Jul 5;14:450. doi: 10.1186/1471-2164-14-450 (PMC3829707; doi:10.1186/1471-2164-14-450)
Supplement: Additional file 19: Figure S6 — Phylogenetic profiles and positions in MPD and MNTD coordinates for all modules. [file 1471-2164-14-450-S19.zip › FigureS6/black.pdf]

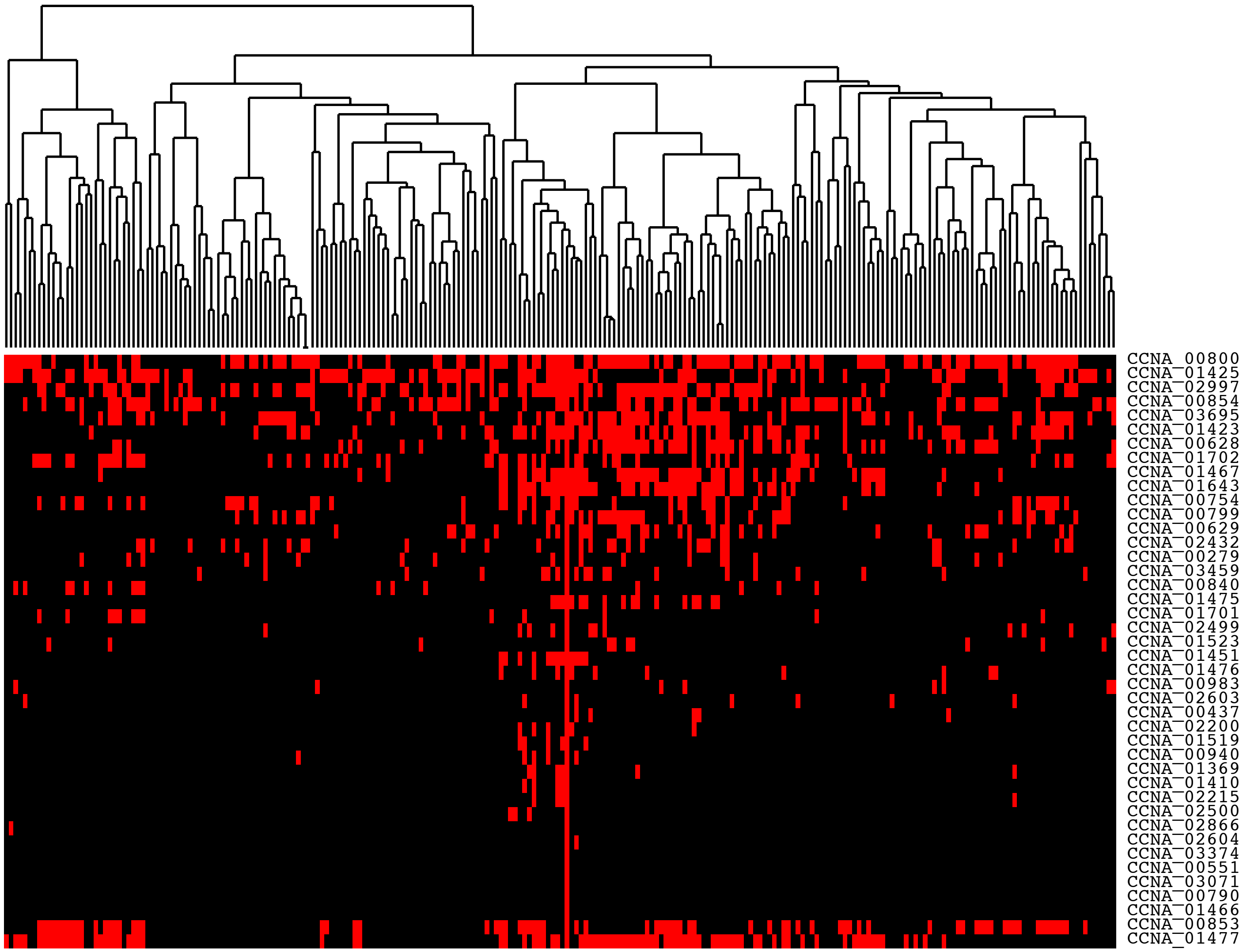
CCNA_00800
CCNA_01425
CCNA_02997
CCNA_00854
CCNA_03695
CCNA_01423
CCNA_00628
CCNA_01702
CCNA_01467
CCNA_01643
CCNA_00754
CCNA_00799
CCNA_00629
CCNA_02432
CCNA_00279
CCNA_03459
CCNA_00840
CCNA_01475
CCNA_01701
CCNA_02499
CCNA_01523
CCNA_01451
CCNA_01476
CCNA_00983
CCNA_02603
CCNA_00437
CCNA_02200
CCNA_01519
CCNA_00940
CCNA_01369
CCNA_01410
CCNA_02215
CCNA_02500
CCNA_02866
CCNA_02604
CCNA_03374
CCNA_00551
CCNA_03071
CCNA_00790
CCNA_01466
CCNA_00853
CCNA_01477
