# Supplementary material for: Transcriptomic and phylogenetic analysis of a bacterial cell cycle reveals strong associations between gene co-expression and evolution
Source: BMC Genomics. 2013 Jul 5;14:450. doi: 10.1186/1471-2164-14-450 (PMC3829707; doi:10.1186/1471-2164-14-450)
Supplement: Additional file 19: Figure S6 — Phylogenetic profiles and positions in MPD and MNTD coordinates for all modules. [file 1471-2164-14-450-S19.zip › FigureS6/blue.pdf]

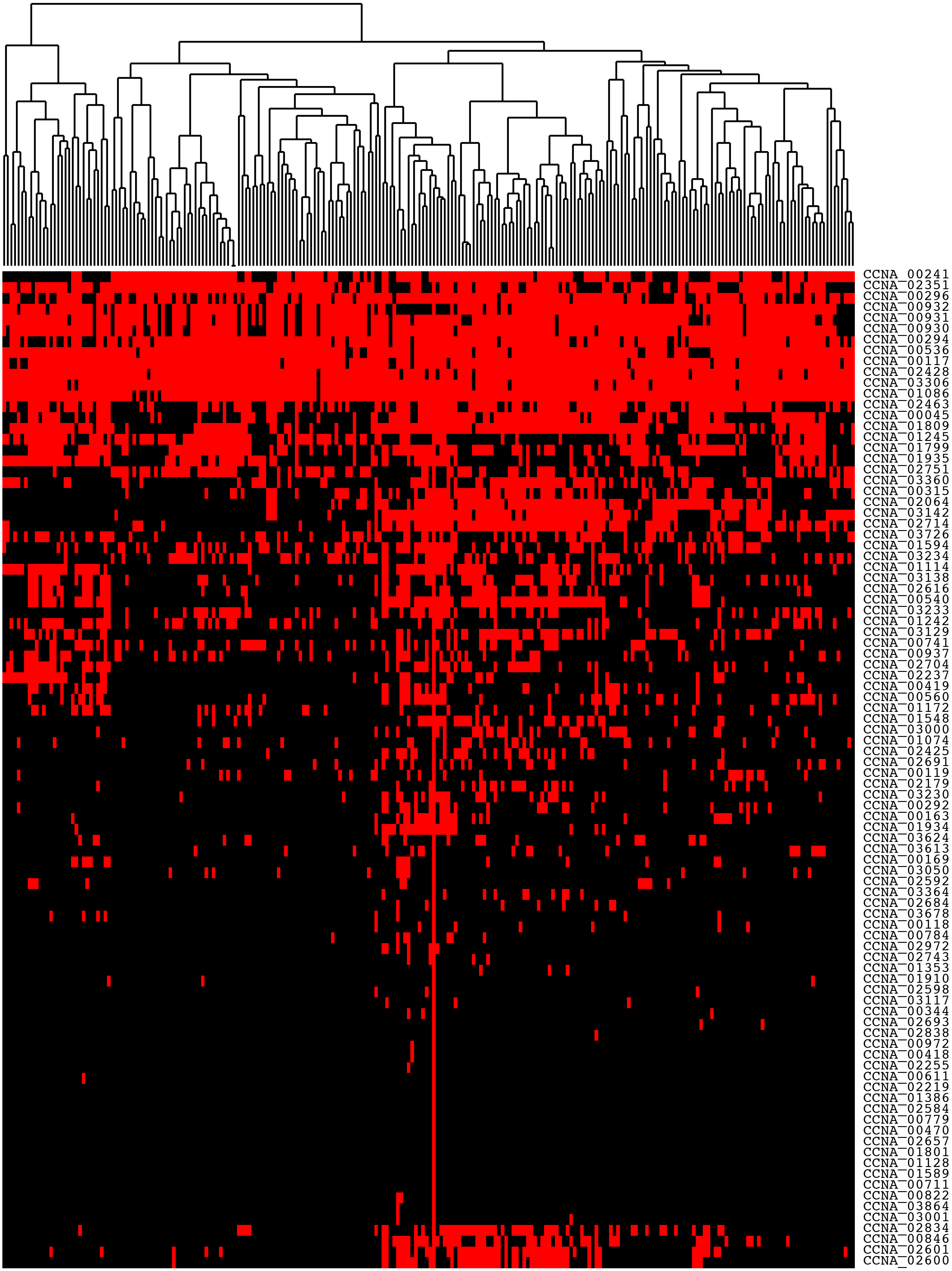
CCNA_00241
CCNA_02351
CCNA_00296
CCNA_00932
CCNA_00931
CCNA_00930
CCNA_00294
CCNA_00536
CCNA_00117
CCNA_02428
CCNA_03306
CCNA_01086
CCNA_02463
CCNA_00045
CCNA_01809
CCNA_01245
CCNA_01799
CCNA_01935
CCNA_02751
CCNA_03360
CCNA_00315
CCNA_02064
CCNA_03142
CCNA_02714
CCNA_03726
CCNA_01594
CCNA_03234
CCNA_01114
CCNA_03138
CCNA_02616
CCNA_00540
CCNA_03233
CCNA_01242
CCNA_03129
CCNA_00741
CCNA_00937
CCNA_02704
CCNA_02237
CCNA_00419
CCNA_00560
CCNA_01172
CCNA_01548
CCNA_03000
CCNA_01074
CCNA_02425
CCNA_02691
CCNA_00119
CCNA_02179
CCNA_03230
CCNA_00292
CCNA_00163
CCNA_01934
CCNA_03624
CCNA_03613
CCNA_00169
CCNA_03050
CCNA_02592
CCNA_03364
CCNA_02684
CCNA_03678
CCNA_00118
CCNA_00784
CCNA_02972
CCNA_02743
CCNA_01353
CCNA_01910
CCNA_02598
CCNA_03117
CCNA_00344
CCNA_02693
CCNA_02838
CCNA_00972
CCNA_00418
CCNA_02255
CCNA_00611
CCNA_02219
CCNA_01386
CCNA_02584
CCNA_00779
CCNA_00470
CCNA_02657
CCNA_01801
CCNA_01128
CCNA_01589
CCNA_00711
CCNA_00822
CCNA_03864
CCNA_03001
CCNA_02834
CCNA_00846
CCNA_02601
CCNA_02600
