# Supplementary material for: Transcriptomic and phylogenetic analysis of a bacterial cell cycle reveals strong associations between gene co-expression and evolution
Source: BMC Genomics. 2013 Jul 5;14:450. doi: 10.1186/1471-2164-14-450 (PMC3829707; doi:10.1186/1471-2164-14-450)
Supplement: Additional file 19: Figure S6 — Phylogenetic profiles and positions in MPD and MNTD coordinates for all modules. [file 1471-2164-14-450-S19.zip › FigureS6/brown.pdf]

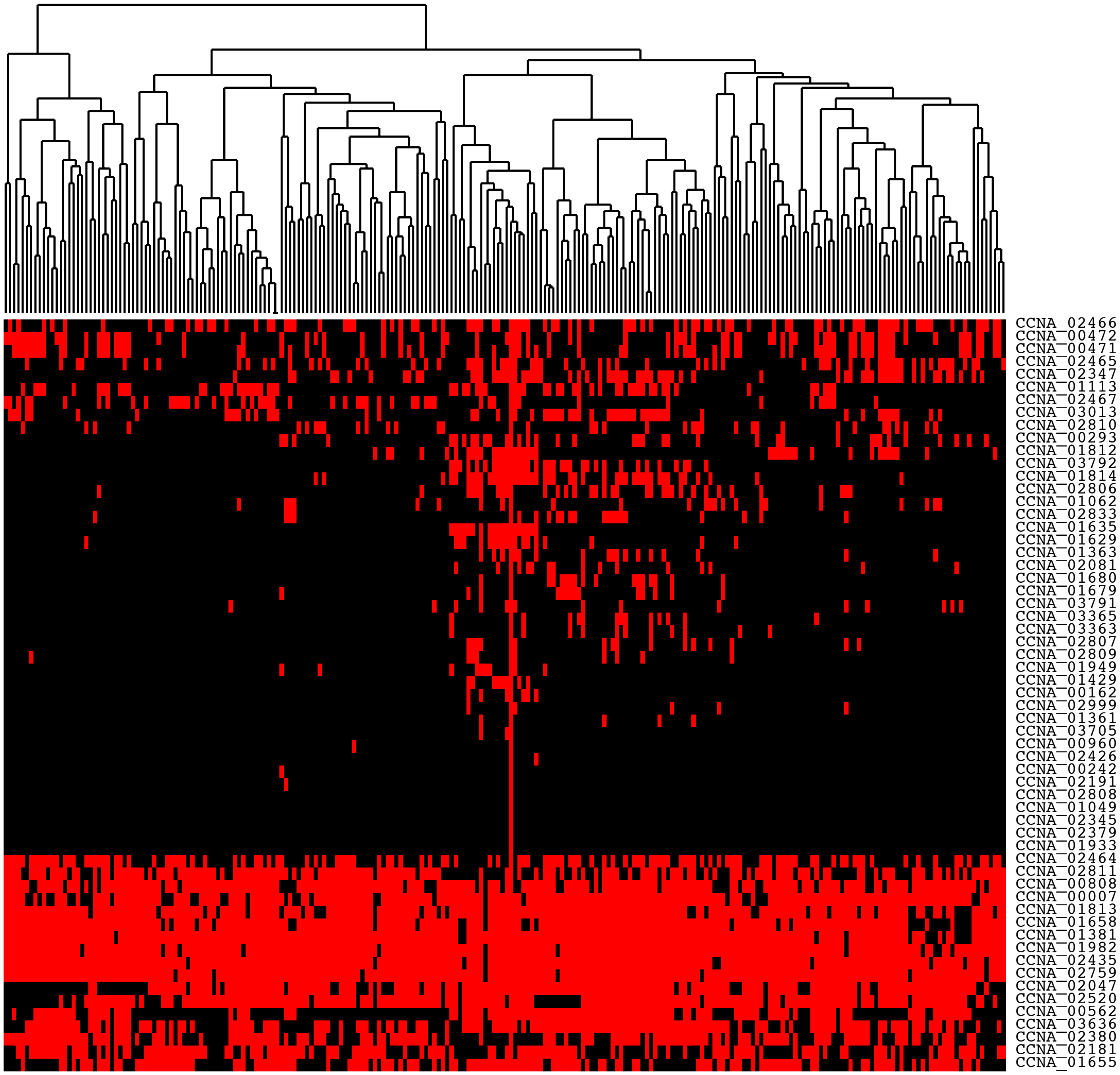
CCNA_02466
CCNA_00472
CCNA_00471
CCNA_02465
CCNA_02347
CCNA_01113
CCNA_02467
CCNA_03013
CCNA_02810
CCNA_00293
CCNA_01812
CCNA_03792
CCNA_01814
CCNA_02806
CCNA_01062
CCNA_02833
CCNA_01635
CCNA_01629
CCNA_01363
CCNA_02081
CCNA_01680
CCNA_01679
CCNA_03791
CCNA_03365
CCNA_03363
CCNA_02807
CCNA_02809
CCNA_01949
CCNA_01429
CCNA_00162
CCNA_02999
CCNA_01361
CCNA_03705
CCNA_00960
CCNA_02426
CCNA_00242
CCNA_02191
CCNA_02808
CCNA_01049
CCNA_02345
CCNA_02379
CCNA_01933
CCNA_02464
CCNA_02811
CCNA_00808
CCNA_00007
CCNA_01813
CCNA_01658
CCNA_01381
CCNA_01982
CCNA_02435
CCNA_02759
CCNA_02047
CCNA_02520
CCNA_00562
CCNA_03636
CCNA_02380
CCNA_02181
CCNA_01655
