# Supplementary material for: Transcriptomic and phylogenetic analysis of a bacterial cell cycle reveals strong associations between gene co-expression and evolution
Source: BMC Genomics. 2013 Jul 5;14:450. doi: 10.1186/1471-2164-14-450 (PMC3829707; doi:10.1186/1471-2164-14-450)
Supplement: Additional file 19: Figure S6 — Phylogenetic profiles and positions in MPD and MNTD coordinates for all modules. [file 1471-2164-14-450-S19.zip › FigureS6/cyan.pdf]

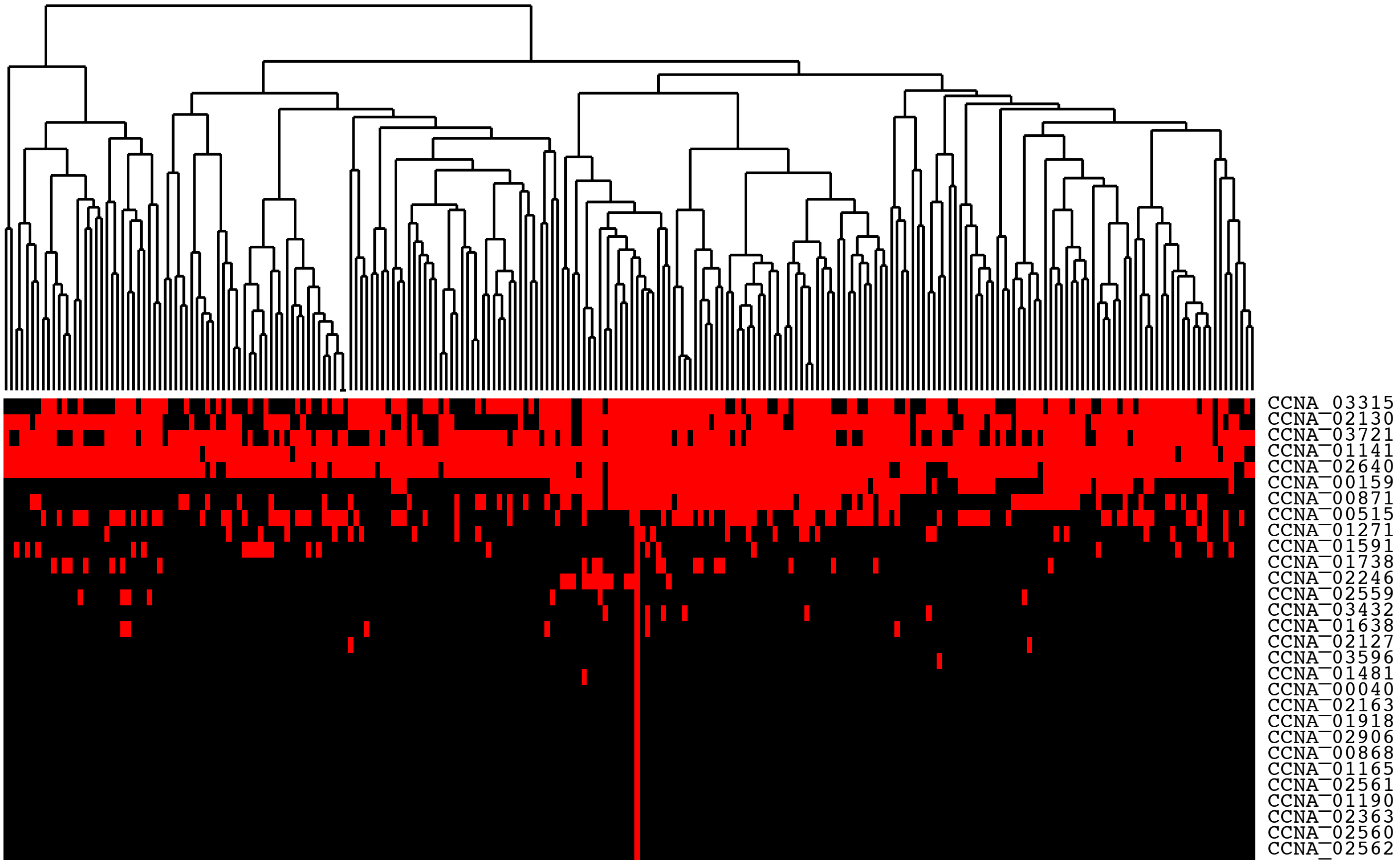
CCNA_03315
CCNA_02130
CCNA_03721
CCNA_01141
CCNA_02640
CCNA_00159
CCNA_00871
CCNA_00515
CCNA_01271
CCNA_01591
CCNA_01738
CCNA_02246
CCNA_02559
CCNA_03432
CCNA_01638
CCNA_02127
CCNA_03596
CCNA_01481
CCNA_00040
CCNA_02163
CCNA_01918
CCNA_02906
CCNA_00868
CCNA_01165
CCNA_02561
CCNA_01190
CCNA_02363
CCNA_02560
CCNA_02562
