# Supplementary material for: Transcriptomic and phylogenetic analysis of a bacterial cell cycle reveals strong associations between gene co-expression and evolution
Source: BMC Genomics. 2013 Jul 5;14:450. doi: 10.1186/1471-2164-14-450 (PMC3829707; doi:10.1186/1471-2164-14-450)
Supplement: Additional file 19: Figure S6 — Phylogenetic profiles and positions in MPD and MNTD coordinates for all modules. [file 1471-2164-14-450-S19.zip › FigureS6/darkgreen.pdf]

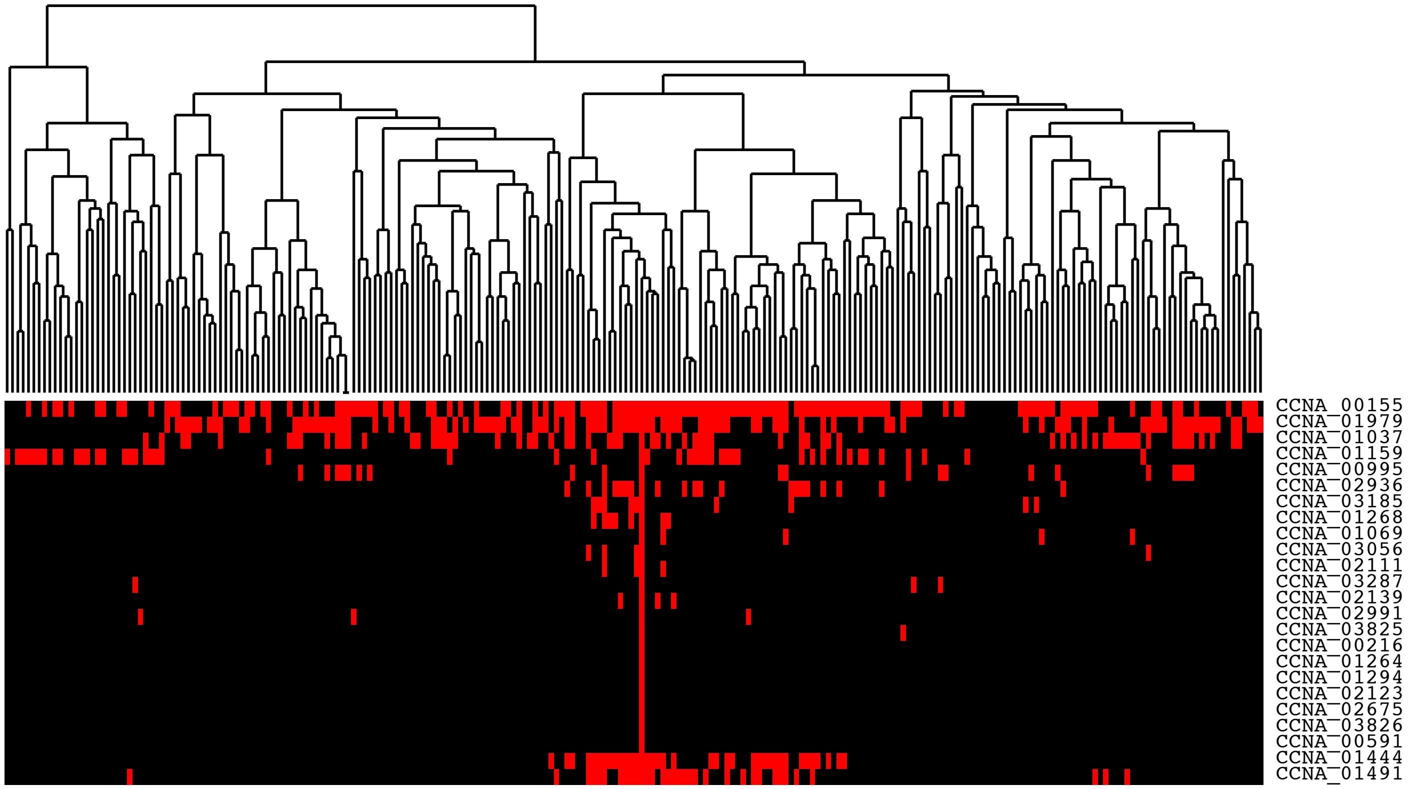
CCNA_00155
CCNA_01979
CCNA_01037
CCNA_01159
CCNA_00995
CCNA_02936
CCNA_03185
CCNA_01268
CCNA_01069
CCNA_03056
CCNA_02111
CCNA_03287
CCNA_02139
CCNA_02991
CCNA_03825
CCNA_00216
CCNA_01264
CCNA_01294
CCNA_02123
CCNA_02675
CCNA_03826
CCNA_00591
CCNA_01444
CCNA_01491
