# Supplementary material for: Transcriptomic and phylogenetic analysis of a bacterial cell cycle reveals strong associations between gene co-expression and evolution
Source: BMC Genomics. 2013 Jul 5;14:450. doi: 10.1186/1471-2164-14-450 (PMC3829707; doi:10.1186/1471-2164-14-450)
Supplement: Additional file 19: Figure S6 — Phylogenetic profiles and positions in MPD and MNTD coordinates for all modules. [file 1471-2164-14-450-S19.zip › FigureS6/darkgrey.pdf]

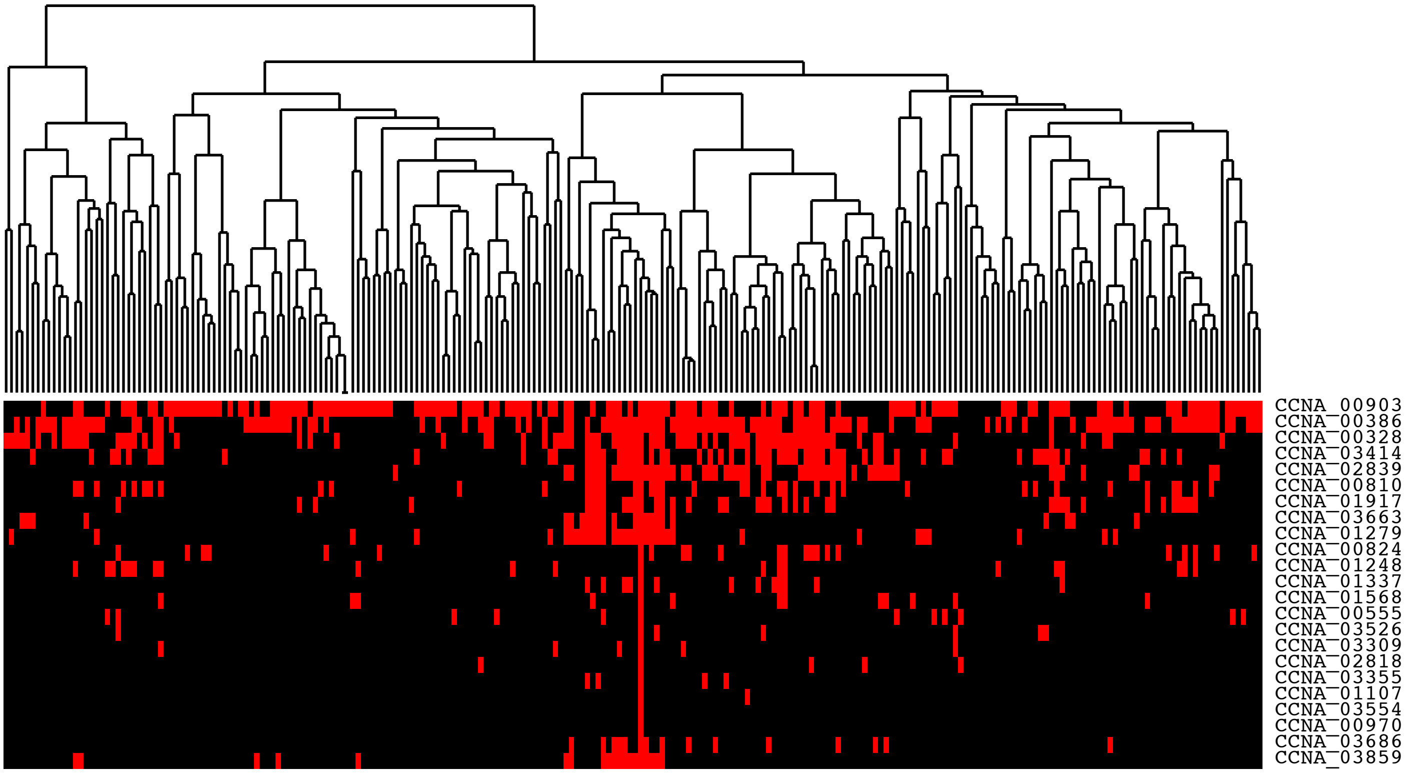
CCNA_00903
CCNA_00386
CCNA_00328
CCNA_03414
CCNA_02839
CCNA_00810
CCNA_01917
CCNA_03663
CCNA_01279
CCNA_00824
CCNA_01248
CCNA_01337
CCNA_01568
CCNA_00555
CCNA_03526
CCNA_03309
CCNA_02818
CCNA_03355
CCNA_01107
CCNA_03554
CCNA_00970
CCNA_03686
CCNA_03859
