# Supplementary material for: Transcriptomic and phylogenetic analysis of a bacterial cell cycle reveals strong associations between gene co-expression and evolution
Source: BMC Genomics. 2013 Jul 5;14:450. doi: 10.1186/1471-2164-14-450 (PMC3829707; doi:10.1186/1471-2164-14-450)
Supplement: Additional file 19: Figure S6 — Phylogenetic profiles and positions in MPD and MNTD coordinates for all modules. [file 1471-2164-14-450-S19.zip › FigureS6/darkorange.pdf]

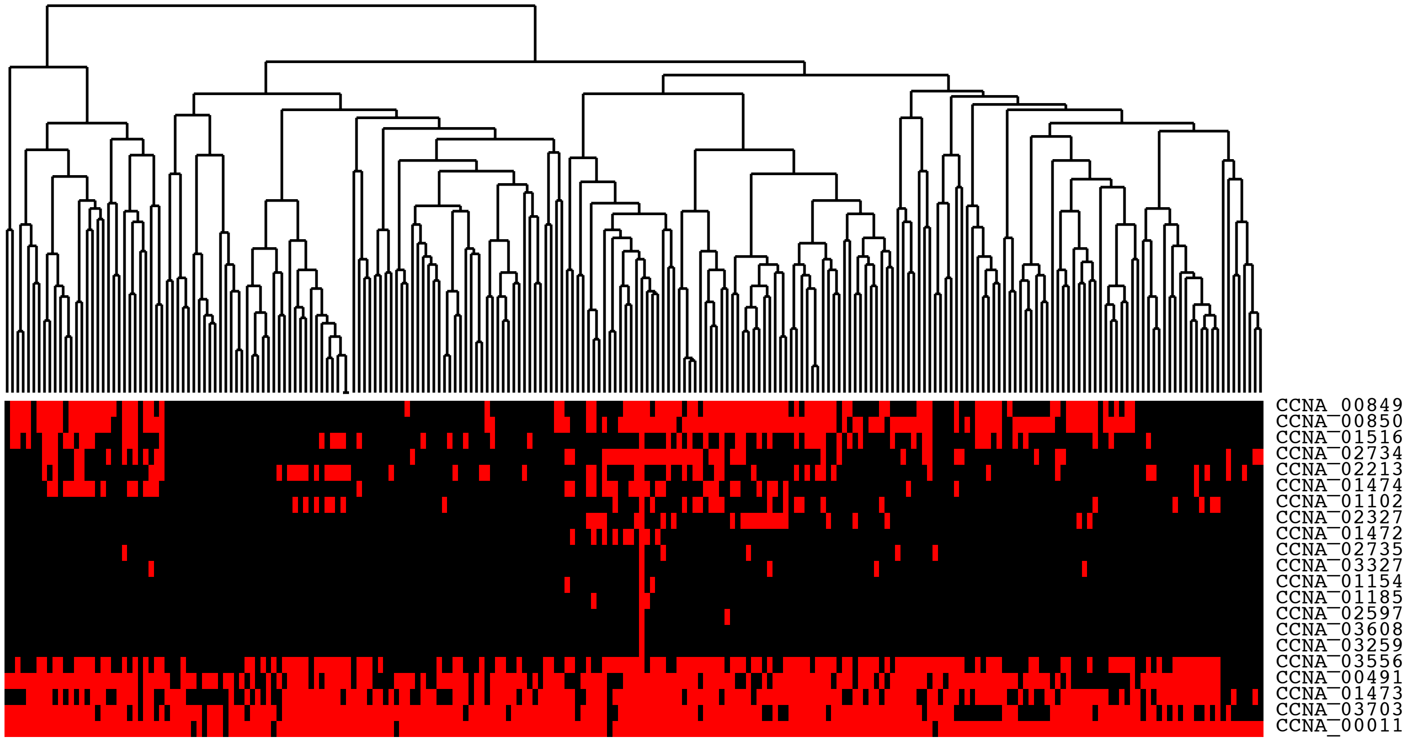
CCNA_00849
CCNA_00850
CCNA_01516
CCNA_02734
CCNA_02213
CCNA_01474
CCNA_01102
CCNA_02327
CCNA_01472
CCNA_02735
CCNA_03327
CCNA_01154
CCNA_01185
CCNA_02597
CCNA_03608
CCNA_03259
CCNA_03556
CCNA_00491
CCNA_01473
CCNA_03703
CCNA_00011
