# Supplementary material for: Transcriptomic and phylogenetic analysis of a bacterial cell cycle reveals strong associations between gene co-expression and evolution
Source: BMC Genomics. 2013 Jul 5;14:450. doi: 10.1186/1471-2164-14-450 (PMC3829707; doi:10.1186/1471-2164-14-450)
Supplement: Additional file 19: Figure S6 — Phylogenetic profiles and positions in MPD and MNTD coordinates for all modules. [file 1471-2164-14-450-S19.zip › FigureS6/darkred.pdf]

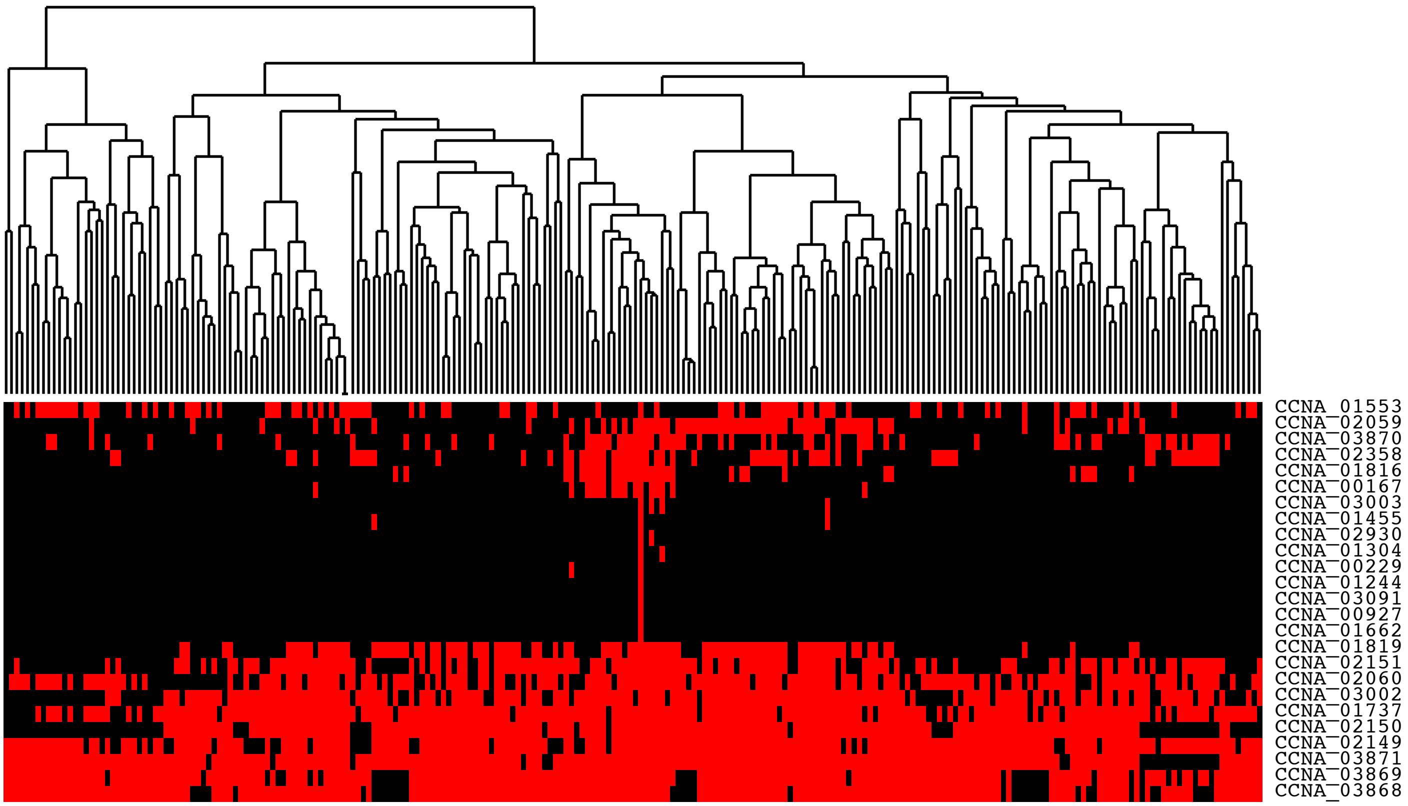
CCNA_01553
CCNA_02059
CCNA_03870
CCNA_02358
CCNA_01816
CCNA_00167
CCNA_03003
CCNA_01455
CCNA_02930
CCNA_01304
CCNA_00229
CCNA_01244
CCNA_03091
CCNA_00927
CCNA_01662
CCNA_01819
CCNA_02151
CCNA_02060
CCNA_03002
CCNA_01737
CCNA_02150
CCNA_02149
CCNA_03871
CCNA_03869
CCNA_03868
