# Supplementary material for: Transcriptomic and phylogenetic analysis of a bacterial cell cycle reveals strong associations between gene co-expression and evolution
Source: BMC Genomics. 2013 Jul 5;14:450. doi: 10.1186/1471-2164-14-450 (PMC3829707; doi:10.1186/1471-2164-14-450)
Supplement: Additional file 19: Figure S6 — Phylogenetic profiles and positions in MPD and MNTD coordinates for all modules. [file 1471-2164-14-450-S19.zip › FigureS6/darkturquoise.pdf]

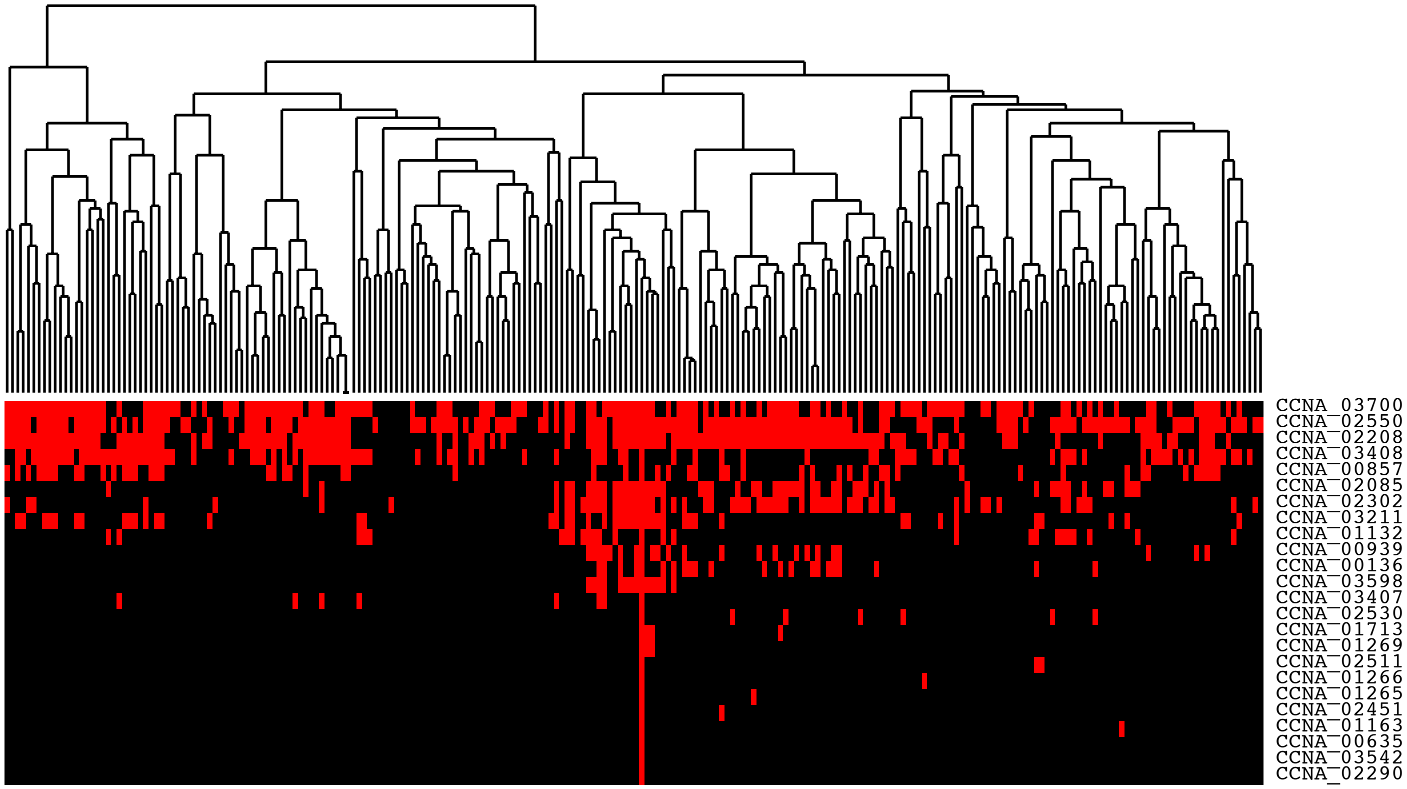

CCNA_03700
CCNA_02550
CCNA_02208
CCNA_03408
CCNA_00857
CCNA_02085
CCNA_02302
CCNA_03211
CCNA_01132
CCNA_00939
CCNA_00136
CCNA_03598
CCNA_03407
CCNA_02530
CCNA_01713
CCNA_01269
CCNA_02511
CCNA_01266
CCNA_01265
CCNA_02451
CCNA_01163
CCNA_00635
CCNA_03542
CCNA_02290
