# Supplementary material for: Transcriptomic and phylogenetic analysis of a bacterial cell cycle reveals strong associations between gene co-expression and evolution
Source: BMC Genomics. 2013 Jul 5;14:450. doi: 10.1186/1471-2164-14-450 (PMC3829707; doi:10.1186/1471-2164-14-450)
Supplement: Additional file 19: Figure S6 — Phylogenetic profiles and positions in MPD and MNTD coordinates for all modules. [file 1471-2164-14-450-S19.zip › FigureS6/green.pdf]

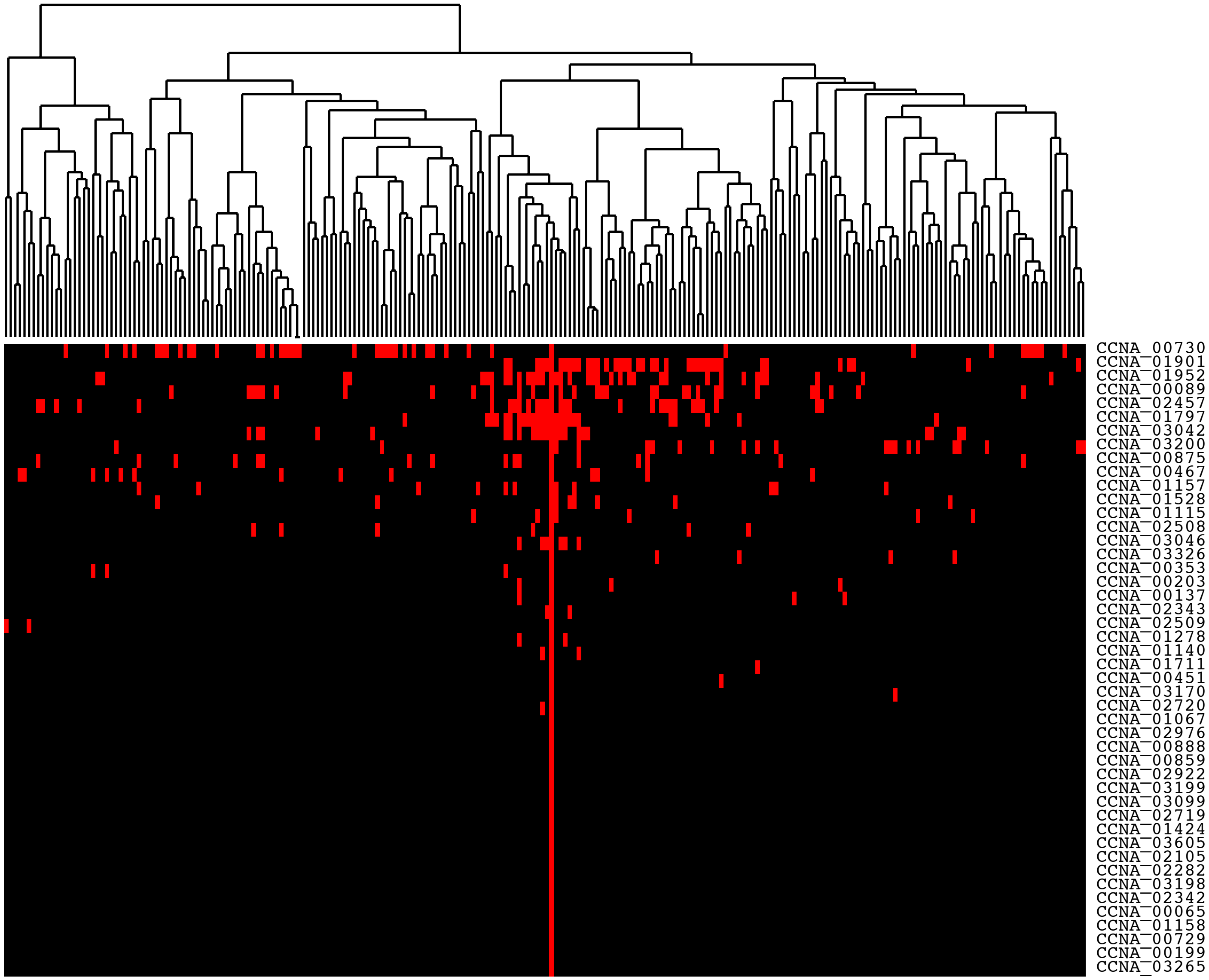
CCNA_00730
CCNA_01901
CCNA_01952
CCNA_00089
CCNA_02457
CCNA_01797
CCNA_03042
CCNA_03200
CCNA_00875
CCNA_00467
CCNA_01157
CCNA_01528
CCNA_01115
CCNA_02508
CCNA_03046
CCNA_03326
CCNA_00353
CCNA_00203
CCNA_00137
CCNA_02343
CCNA_02509
CCNA_01278
CCNA_01140
CCNA_01711
CCNA_00451
CCNA_03170
CCNA_02720
CCNA_01067
CCNA_02976
CCNA_00888
CCNA_00859
CCNA_02922
CCNA_03199
CCNA_03099
CCNA_02719
CCNA_01424
CCNA_03605
CCNA_02105
CCNA_02282
CCNA_03198
CCNA_02342
CCNA_00065
CCNA_01158
CCNA_00729
CCNA_00199
CCNA_03265
