# Supplementary material for: Transcriptomic and phylogenetic analysis of a bacterial cell cycle reveals strong associations between gene co-expression and evolution
Source: BMC Genomics. 2013 Jul 5;14:450. doi: 10.1186/1471-2164-14-450 (PMC3829707; doi:10.1186/1471-2164-14-450)
Supplement: Additional file 19: Figure S6 — Phylogenetic profiles and positions in MPD and MNTD coordinates for all modules. [file 1471-2164-14-450-S19.zip › FigureS6/greenyellow.pdf]

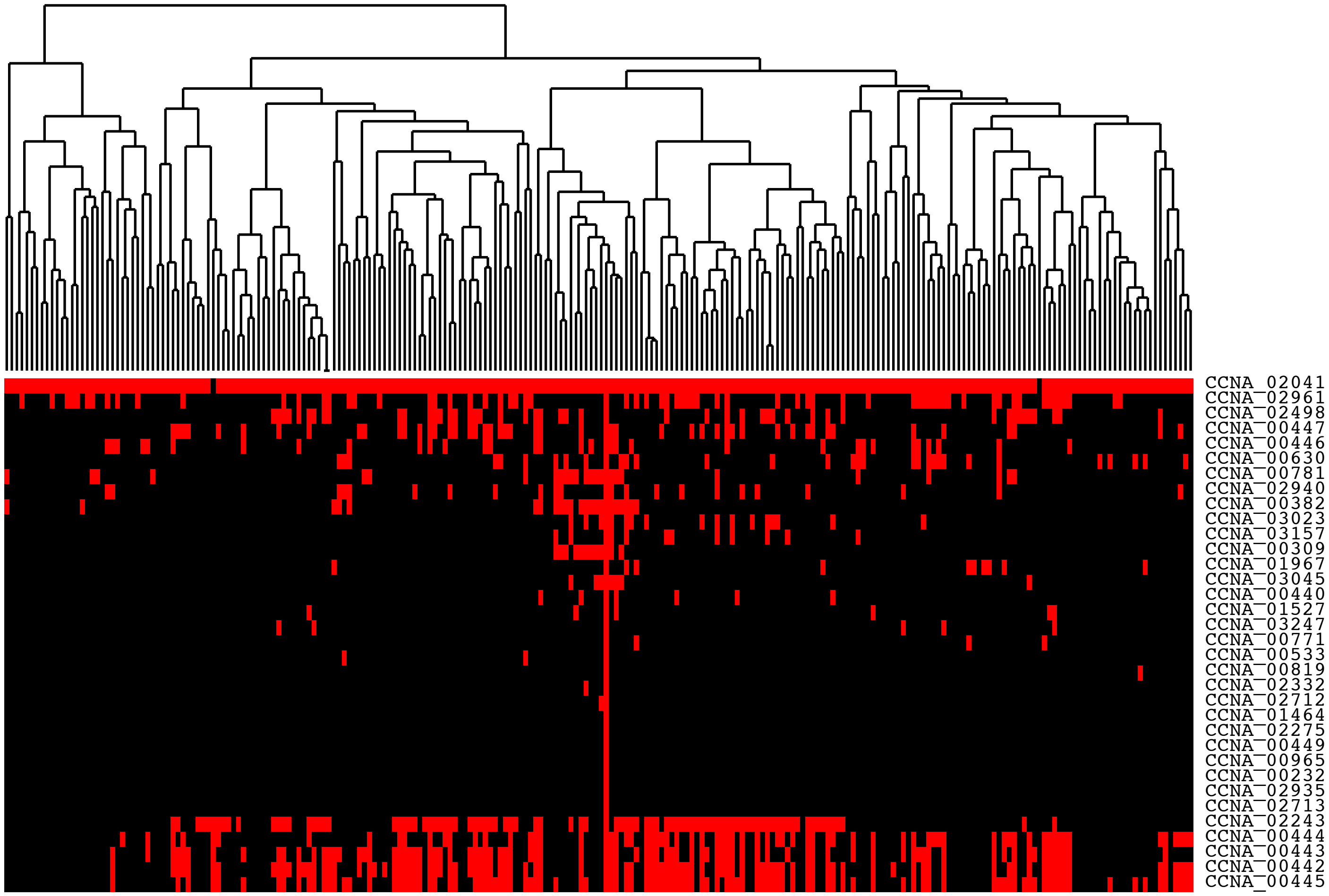
CCNA_02041
CCNA_02961
CCNA_02498
CCNA_00447
CCNA_00446
CCNA_00630
CCNA_00781
CCNA_02940
CCNA_00382
CCNA_03023
CCNA_03157
CCNA_00309
CCNA_01967
CCNA_03045
CCNA_00440
CCNA_01527
CCNA_03247
CCNA_00771
CCNA_00533
CCNA_00819
CCNA_02332
CCNA_02712
CCNA_01464
CCNA_02275
CCNA_00449
CCNA_00965
CCNA_00232
CCNA_02935
CCNA_02713
CCNA_02243
CCNA_00444
CCNA_00443
CCNA_00442
CCNA_00445
