# Supplementary material for: Transcriptomic and phylogenetic analysis of a bacterial cell cycle reveals strong associations between gene co-expression and evolution
Source: BMC Genomics. 2013 Jul 5;14:450. doi: 10.1186/1471-2164-14-450 (PMC3829707; doi:10.1186/1471-2164-14-450)
Supplement: Additional file 19: Figure S6 — Phylogenetic profiles and positions in MPD and MNTD coordinates for all modules. [file 1471-2164-14-450-S19.zip › FigureS6/grey60.pdf]

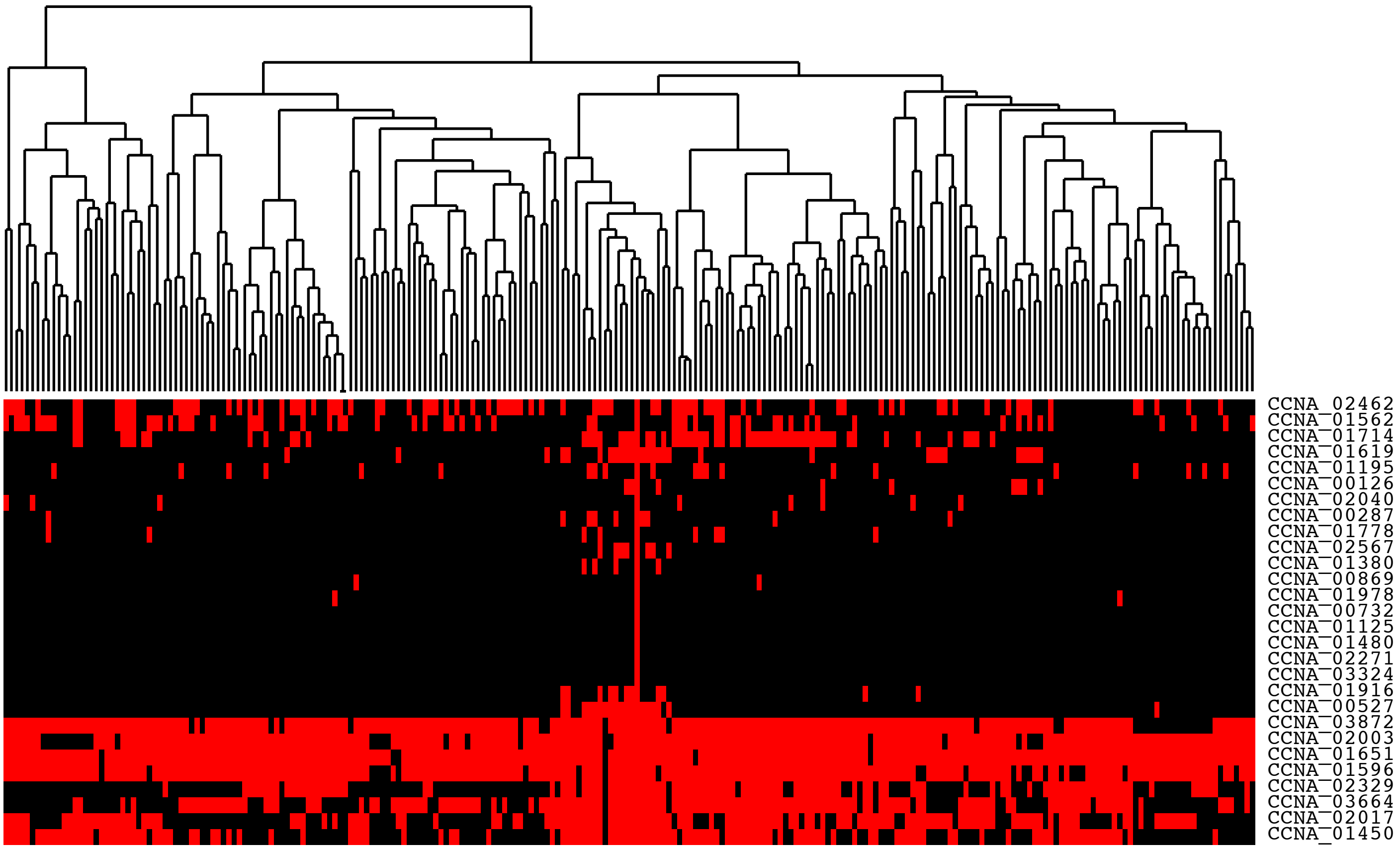
CCNA_02462
CCNA_01562
CCNA_01714
CCNA_01619
CCNA_01195
CCNA_00126
CCNA_02040
CCNA_00287
CCNA_01778
CCNA_02567
CCNA_01380
CCNA_00869
CCNA_01978
CCNA_00732
CCNA_01125
CCNA_01480
CCNA_02271
CCNA_03324
CCNA_01916
CCNA_00527
CCNA_03872
CCNA_02003
CCNA_01651
CCNA_01596
CCNA_02329
CCNA_03664
CCNA_02017
CCNA_01450
