# Supplementary material for: Transcriptomic and phylogenetic analysis of a bacterial cell cycle reveals strong associations between gene co-expression and evolution
Source: BMC Genomics. 2013 Jul 5;14:450. doi: 10.1186/1471-2164-14-450 (PMC3829707; doi:10.1186/1471-2164-14-450)
Supplement: Additional file 19: Figure S6 — Phylogenetic profiles and positions in MPD and MNTD coordinates for all modules. [file 1471-2164-14-450-S19.zip › FigureS6/lightcyan.pdf]

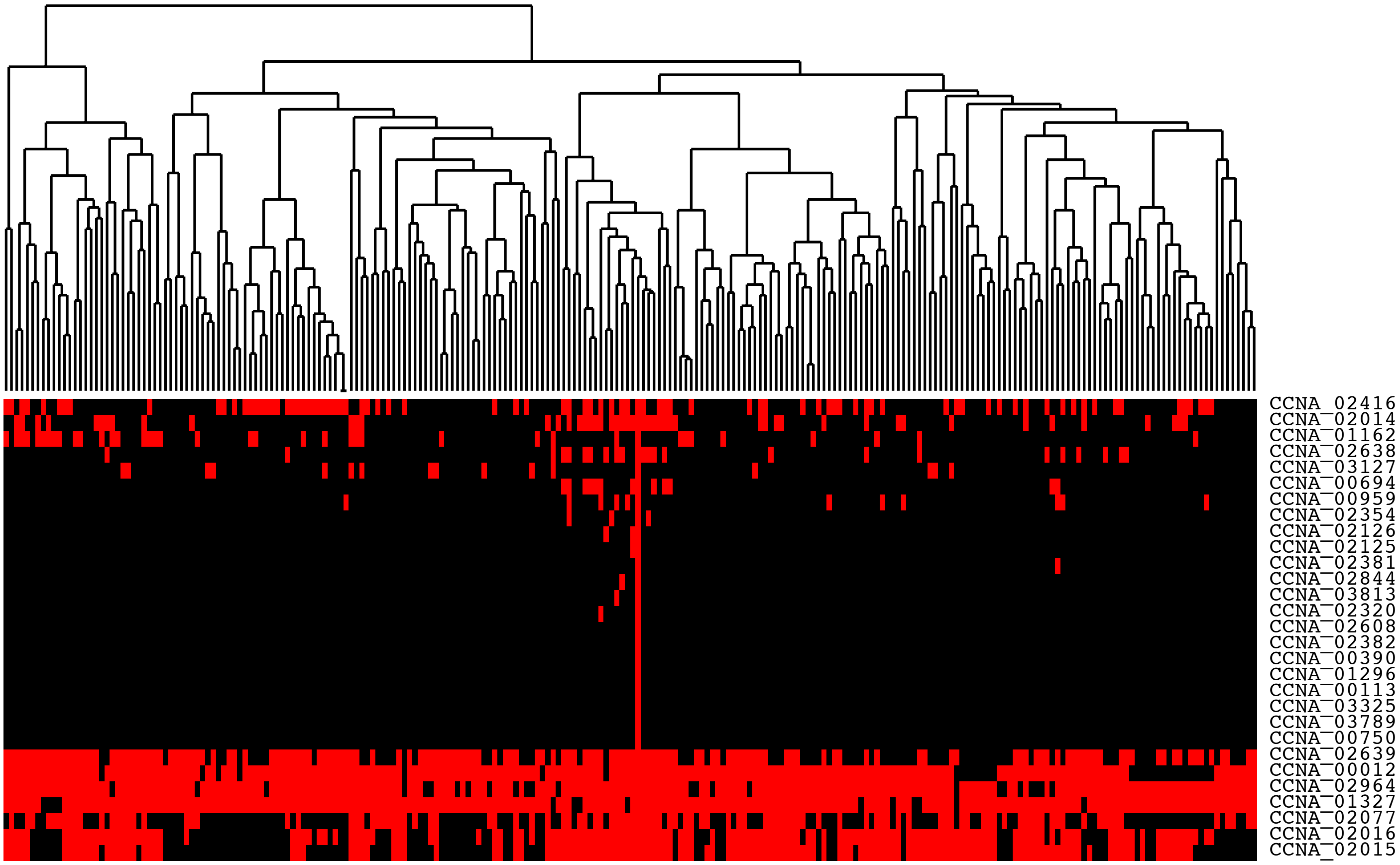
CCNA_02416
CCNA_02014
CCNA_01162
CCNA_02638
CCNA_03127
CCNA_00694
CCNA_00959
CCNA_02354
CCNA_02126
CCNA_02125
CCNA_02381
CCNA_02844
CCNA_03813
CCNA_02320
CCNA_02608
CCNA_02382
CCNA_00390
CCNA_01296
CCNA_00113
CCNA_03325
CCNA_03789
CCNA_00750
CCNA_02639
CCNA_00012
CCNA_02964
CCNA_01327
CCNA_02077
CCNA_02016
CCNA_02015
