# Supplementary material for: Transcriptomic and phylogenetic analysis of a bacterial cell cycle reveals strong associations between gene co-expression and evolution
Source: BMC Genomics. 2013 Jul 5;14:450. doi: 10.1186/1471-2164-14-450 (PMC3829707; doi:10.1186/1471-2164-14-450)
Supplement: Additional file 19: Figure S6 — Phylogenetic profiles and positions in MPD and MNTD coordinates for all modules. [file 1471-2164-14-450-S19.zip › FigureS6/lightgreen.pdf]

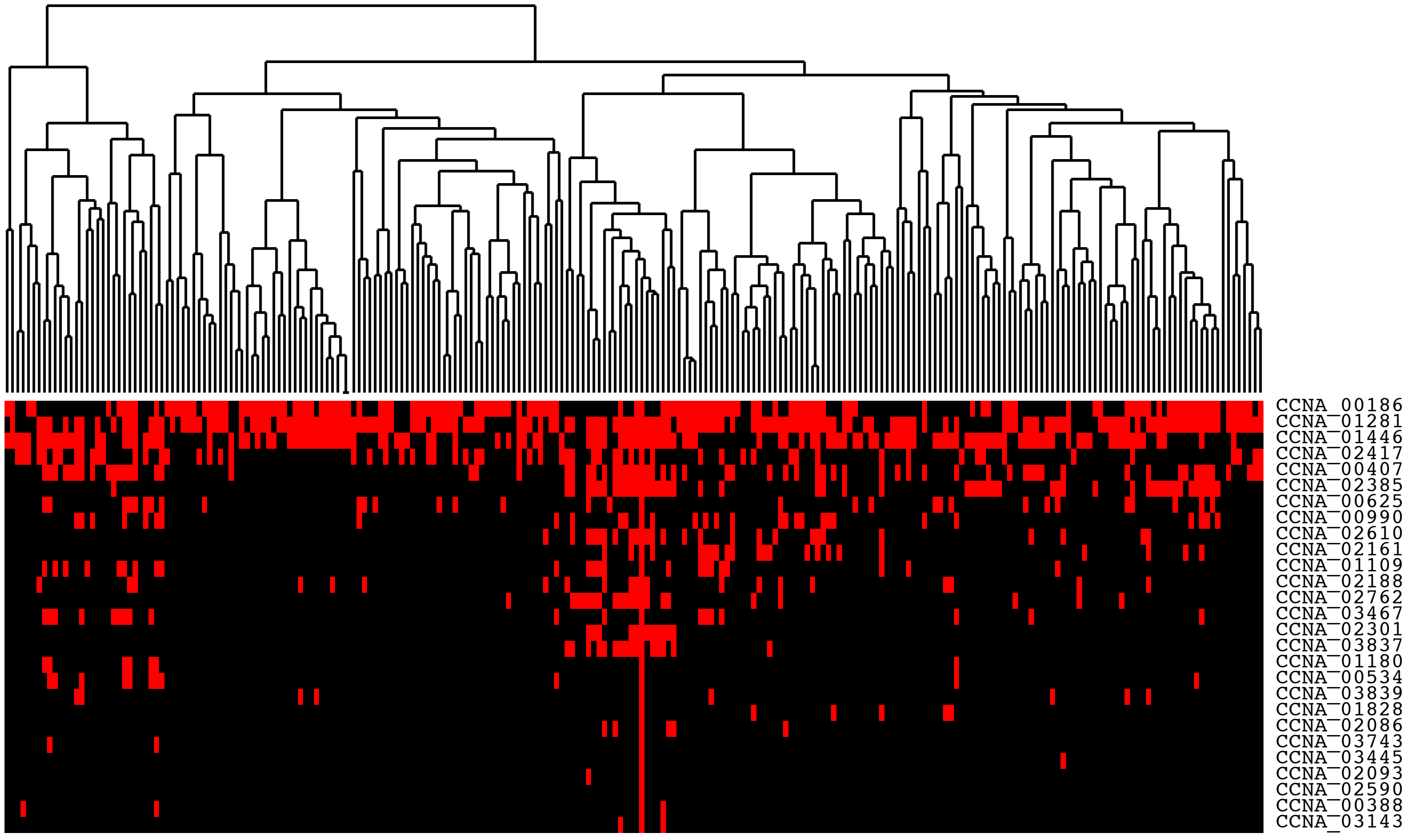
CCNA_00186
CCNA_01281
CCNA_01446
CCNA_02417
CCNA_00407
CCNA_02385
CCNA_00625
CCNA_00990
CCNA_02610
CCNA_02161
CCNA_01109
CCNA_02188
CCNA_02762
CCNA_03467
CCNA_02301
CCNA_03837
CCNA_01180
CCNA_00534
CCNA_03839
CCNA_01828
CCNA_02086
CCNA_03743
CCNA_03445
CCNA_02093
CCNA_02590
CCNA_00388
CCNA_03143
