# Supplementary material for: Transcriptomic and phylogenetic analysis of a bacterial cell cycle reveals strong associations between gene co-expression and evolution
Source: BMC Genomics. 2013 Jul 5;14:450. doi: 10.1186/1471-2164-14-450 (PMC3829707; doi:10.1186/1471-2164-14-450)
Supplement: Additional file 19: Figure S6 — Phylogenetic profiles and positions in MPD and MNTD coordinates for all modules. [file 1471-2164-14-450-S19.zip › FigureS6/lightyellow.pdf]

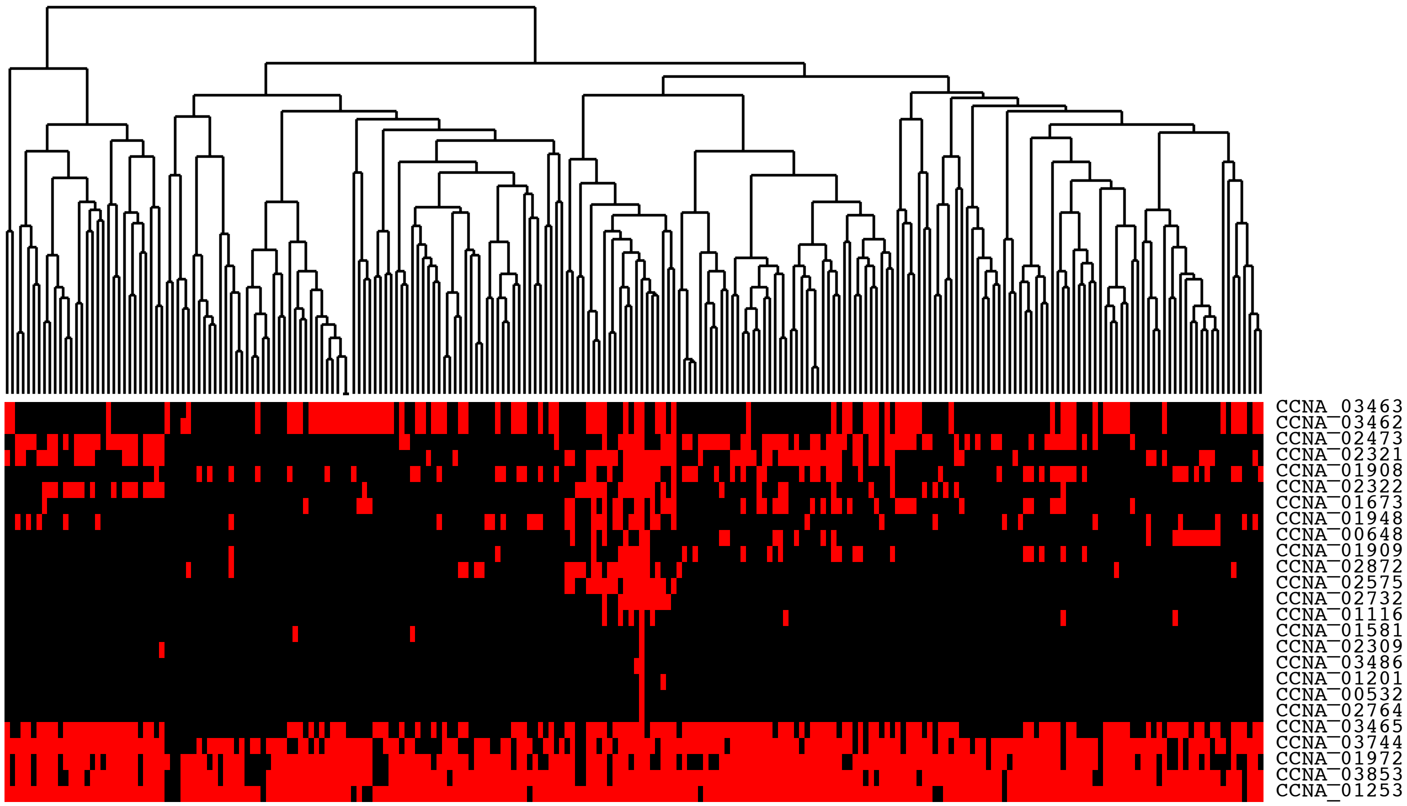
CCNA_03463
CCNA_03462
CCNA_02473
CCNA_02321
CCNA_01908
CCNA_02322
CCNA_01673
CCNA_01948
CCNA_00648
CCNA_01909
CCNA_02872
CCNA_02575
CCNA_02732
CCNA_01116
CCNA_01581
CCNA_02309
CCNA_03486
CCNA_01201
CCNA_00532
CCNA_02764
CCNA_03465
CCNA_03744
CCNA_01972
CCNA_03853
CCNA_01253
