# Supplementary material for: Transcriptomic and phylogenetic analysis of a bacterial cell cycle reveals strong associations between gene co-expression and evolution
Source: BMC Genomics. 2013 Jul 5;14:450. doi: 10.1186/1471-2164-14-450 (PMC3829707; doi:10.1186/1471-2164-14-450)
Supplement: Additional file 19: Figure S6 — Phylogenetic profiles and positions in MPD and MNTD coordinates for all modules. [file 1471-2164-14-450-S19.zip › FigureS6/magenta.pdf]

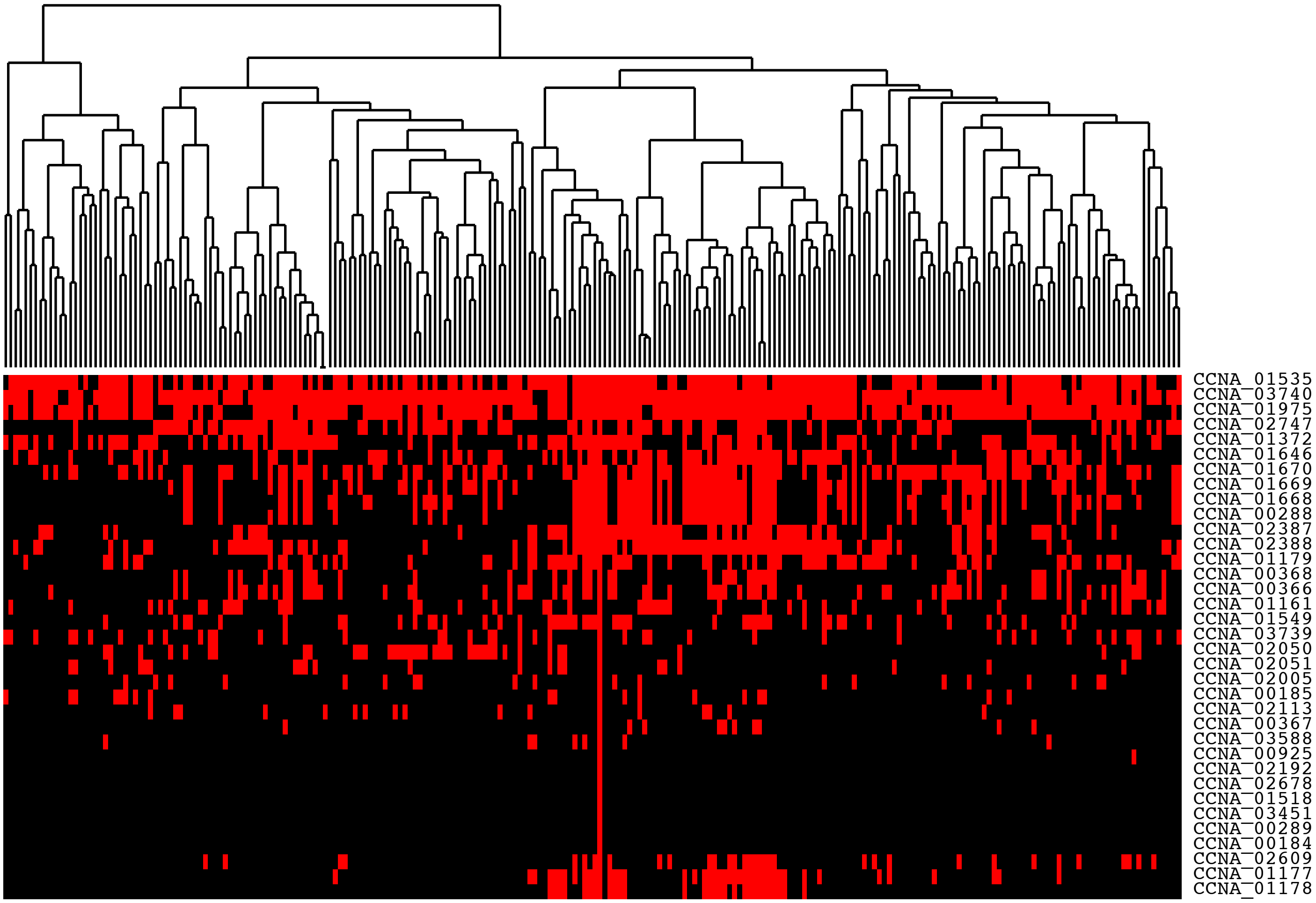
CCNA_01535
CCNA_03740
CCNA_01975
CCNA_02747
CCNA_01372
CCNA_01646
CCNA_01670
CCNA_01669
CCNA_01668
CCNA_00288
CCNA_02387
CCNA_02388
CCNA_01179
CCNA_00368
CCNA_00366
CCNA_01161
CCNA_01549
CCNA_03739
CCNA_02050
CCNA_02051
CCNA_02005
CCNA_00185
CCNA_02113
CCNA_00367
CCNA_03588
CCNA_00925
CCNA_02192
CCNA_02678
CCNA_01518
CCNA_03451
CCNA_00289
CCNA_00184
CCNA_02609
CCNA_01177
CCNA_01178
