# Supplementary material for: Transcriptomic and phylogenetic analysis of a bacterial cell cycle reveals strong associations between gene co-expression and evolution
Source: BMC Genomics. 2013 Jul 5;14:450. doi: 10.1186/1471-2164-14-450 (PMC3829707; doi:10.1186/1471-2164-14-450)
Supplement: Additional file 19: Figure S6 — Phylogenetic profiles and positions in MPD and MNTD coordinates for all modules. [file 1471-2164-14-450-S19.zip › FigureS6/midnightblue.pdf]

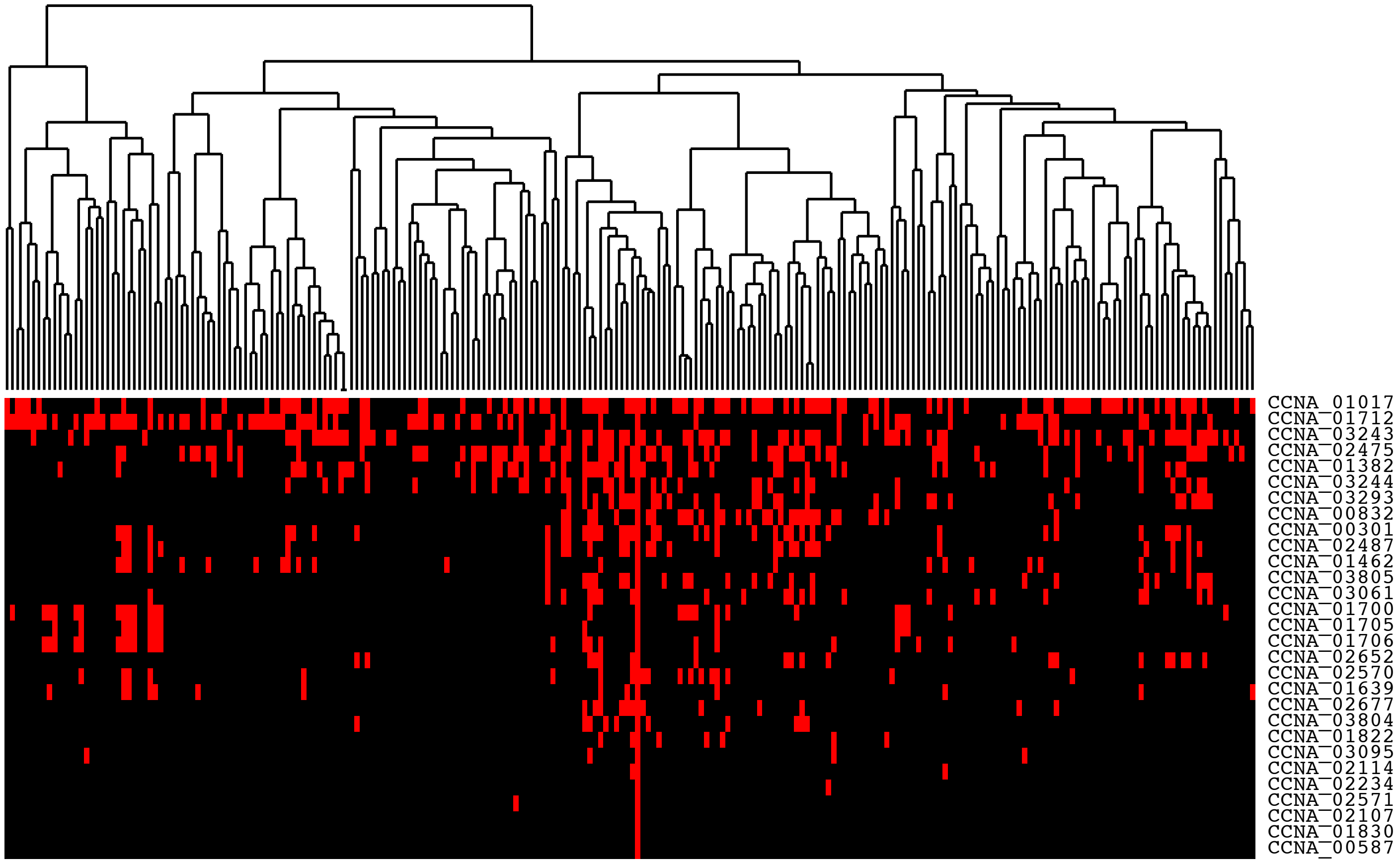

CCNA_01017
CCNA_01712
CCNA_03243
CCNA_02475
CCNA_01382
CCNA_03244
CCNA_03293
CCNA_00832
CCNA_00301
CCNA_02487
CCNA_01462
CCNA_03805
CCNA_03061
CCNA_01700
CCNA_01705
CCNA_01706
CCNA_02652
CCNA_02570
CCNA_01639
CCNA_02677
CCNA_03804
CCNA_01822
CCNA_03095
CCNA_02114
CCNA_02234
CCNA_02571
CCNA_02107
CCNA_01830
CCNA_00587
