# Supplementary material for: Transcriptomic and phylogenetic analysis of a bacterial cell cycle reveals strong associations between gene co-expression and evolution
Source: BMC Genomics. 2013 Jul 5;14:450. doi: 10.1186/1471-2164-14-450 (PMC3829707; doi:10.1186/1471-2164-14-450)
Supplement: Additional file 19: Figure S6 — Phylogenetic profiles and positions in MPD and MNTD coordinates for all modules. [file 1471-2164-14-450-S19.zip › FigureS6/orange.pdf]

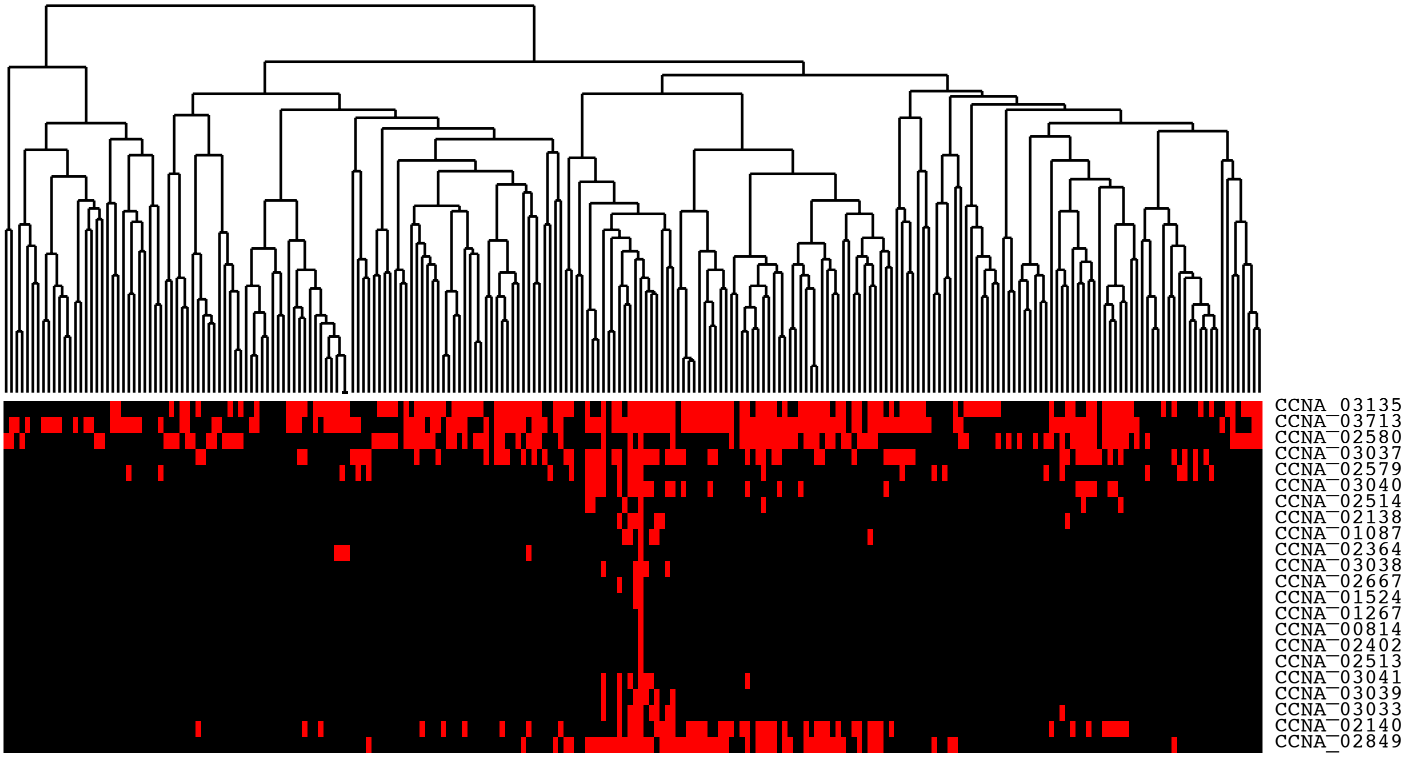
CCNA_03135
CCNA_03713
CCNA_02580
CCNA_03037
CCNA_02579
CCNA_03040
CCNA_02514
CCNA_02138
CCNA_01087
CCNA_02364
CCNA_03038
CCNA_02667
CCNA_01524
CCNA_01267
CCNA_00814
CCNA_02402
CCNA_02513
CCNA_03041
CCNA_03039
CCNA_03033
CCNA_02140
CCNA_02849
