# Supplementary material for: Transcriptomic and phylogenetic analysis of a bacterial cell cycle reveals strong associations between gene co-expression and evolution
Source: BMC Genomics. 2013 Jul 5;14:450. doi: 10.1186/1471-2164-14-450 (PMC3829707; doi:10.1186/1471-2164-14-450)
Supplement: Additional file 19: Figure S6 — Phylogenetic profiles and positions in MPD and MNTD coordinates for all modules. [file 1471-2164-14-450-S19.zip › FigureS6/paleturquoise.pdf]

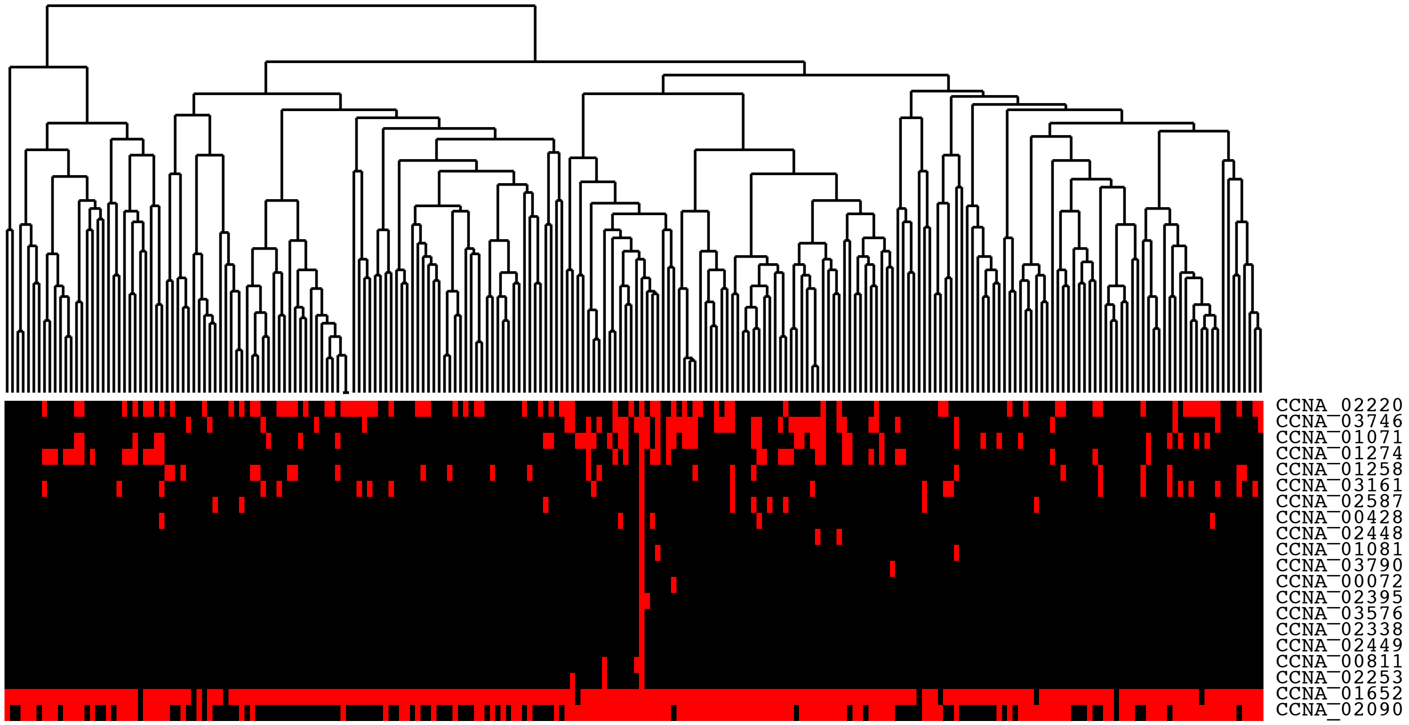
CCNA_02220
CCNA_03746
CCNA_01071
CCNA_01274
CCNA_01258
CCNA_03161
CCNA_02587
CCNA_00428
CCNA_02448
CCNA_01081
CCNA_03790
CCNA_00072
CCNA_02395
CCNA_03576
CCNA_02338
CCNA_02449
CCNA_00811
CCNA_02253
CCNA_01652
CCNA_02090
