# Supplementary material for: Transcriptomic and phylogenetic analysis of a bacterial cell cycle reveals strong associations between gene co-expression and evolution
Source: BMC Genomics. 2013 Jul 5;14:450. doi: 10.1186/1471-2164-14-450 (PMC3829707; doi:10.1186/1471-2164-14-450)
Supplement: Additional file 19: Figure S6 — Phylogenetic profiles and positions in MPD and MNTD coordinates for all modules. [file 1471-2164-14-450-S19.zip › FigureS6/pink.pdf]

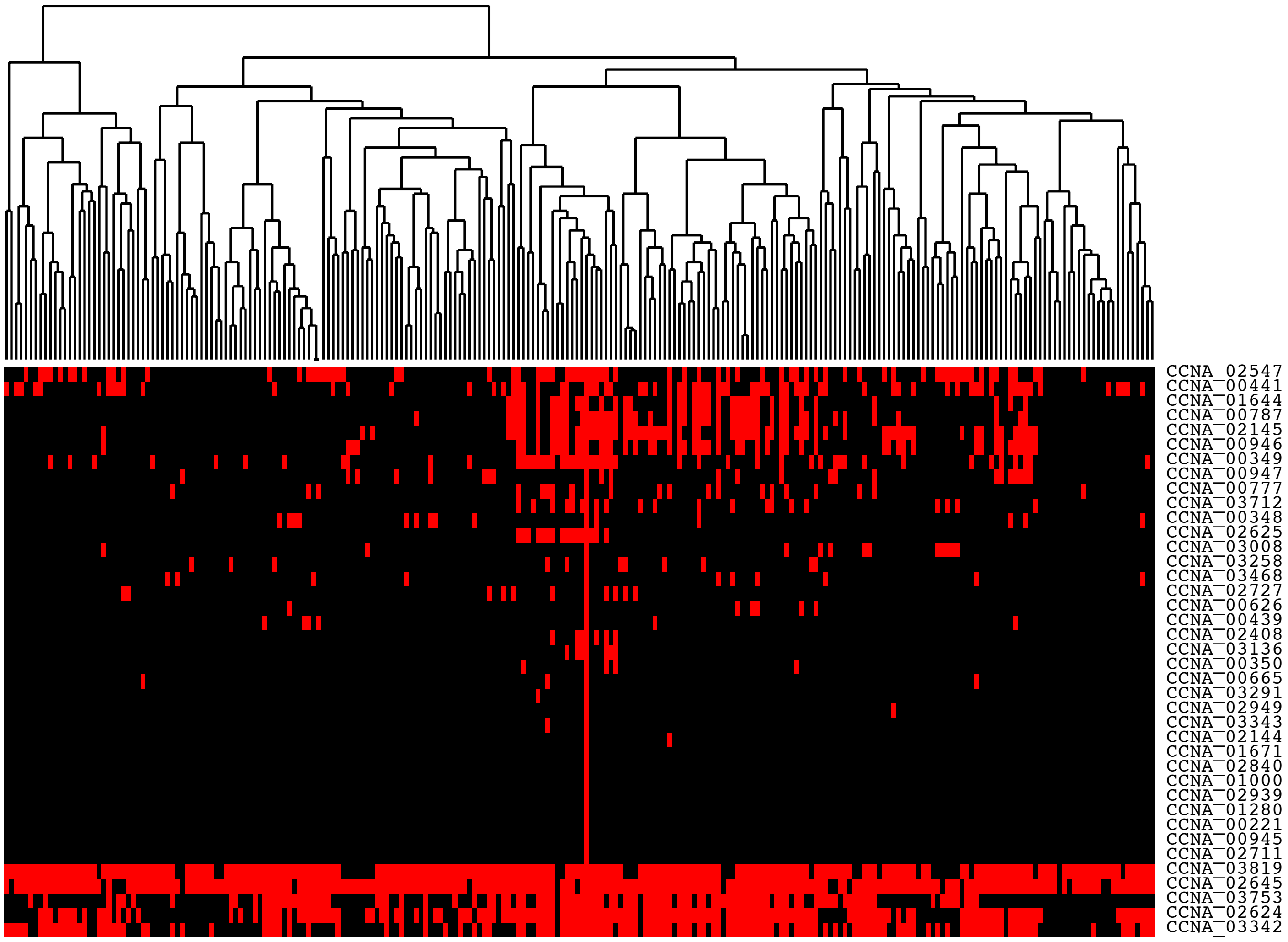
CCNA_02547
CCNA_00441
CCNA_01644
CCNA_00787
CCNA_02145
CCNA_00946
CCNA_00349
CCNA_00947
CCNA_00777
CCNA_03712
CCNA_00348
CCNA_02625
CCNA_03008
CCNA_03258
CCNA_03468
CCNA_02727
CCNA_00626
CCNA_00439
CCNA_02408
CCNA_03136
CCNA_00350
CCNA_00665
CCNA_03291
CCNA_02949
CCNA_03343
CCNA_02144
CCNA_01671
CCNA_02840
CCNA_01000
CCNA_02939
CCNA_01280
CCNA_00221
CCNA_00945
CCNA_02711
CCNA_03819
CCNA_02645
CCNA_03753
CCNA_02624
CCNA_03342
