# Supplementary material for: Transcriptomic and phylogenetic analysis of a bacterial cell cycle reveals strong associations between gene co-expression and evolution
Source: BMC Genomics. 2013 Jul 5;14:450. doi: 10.1186/1471-2164-14-450 (PMC3829707; doi:10.1186/1471-2164-14-450)
Supplement: Additional file 19: Figure S6 — Phylogenetic profiles and positions in MPD and MNTD coordinates for all modules. [file 1471-2164-14-450-S19.zip › FigureS6/purple.pdf]

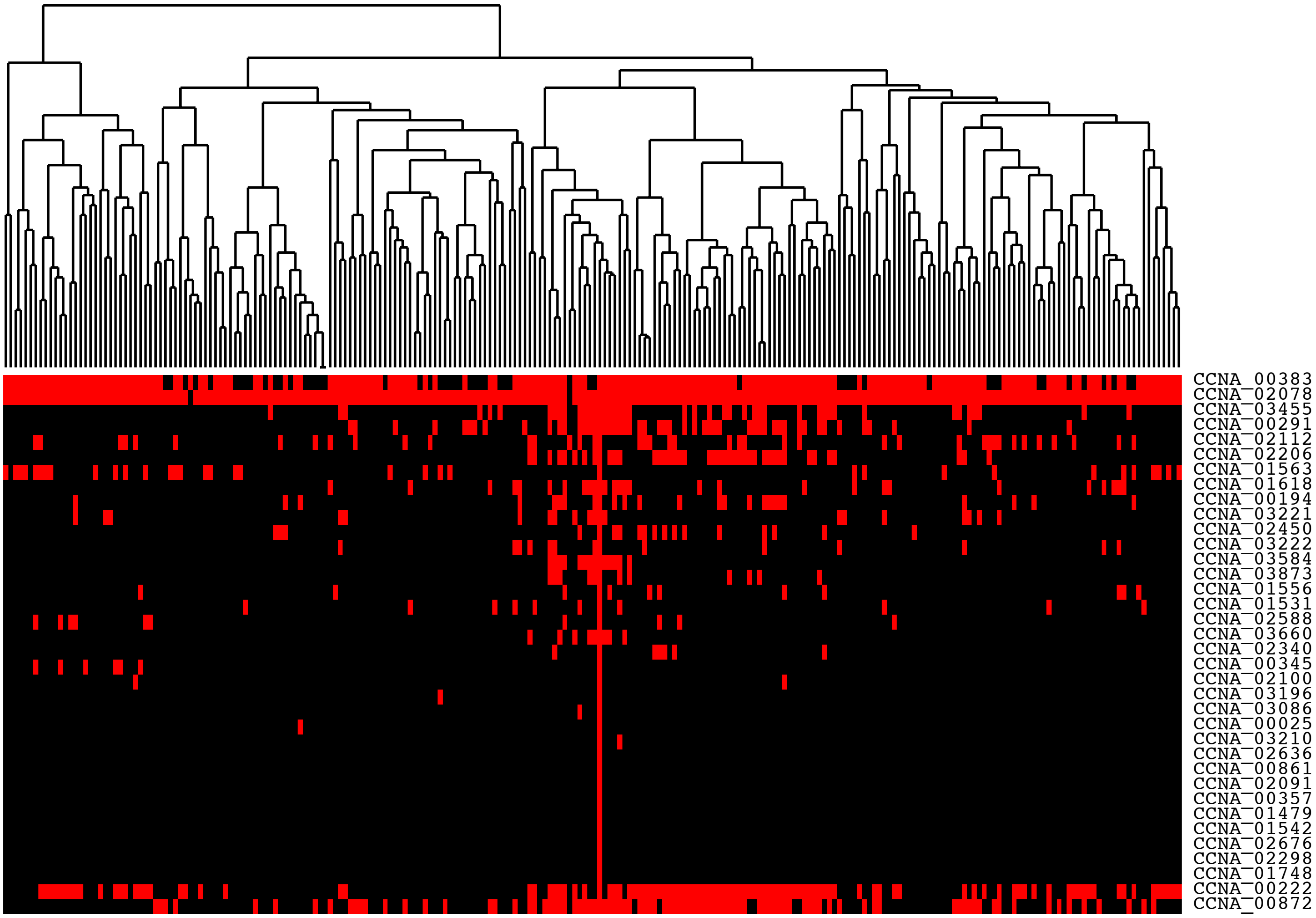
CCNA_00383
CCNA_02078
CCNA_03455
CCNA_00291
CCNA_02112
CCNA_02206
CCNA_01563
CCNA_01618
CCNA_00194
CCNA_03221
CCNA_02450
CCNA_03222
CCNA_03584
CCNA_03873
CCNA_01556
CCNA_01531
CCNA_02588
CCNA_03660
CCNA_02340
CCNA_00345
CCNA_02100
CCNA_03196
CCNA_03086
CCNA_00025
CCNA_03210
CCNA_02636
CCNA_00861
CCNA_02091
CCNA_00357
CCNA_01479
CCNA_01542
CCNA_02676
CCNA_02298
CCNA_01748
CCNA_00222
CCNA_00872
