# Supplementary material for: Transcriptomic and phylogenetic analysis of a bacterial cell cycle reveals strong associations between gene co-expression and evolution
Source: BMC Genomics. 2013 Jul 5;14:450. doi: 10.1186/1471-2164-14-450 (PMC3829707; doi:10.1186/1471-2164-14-450)
Supplement: Additional file 19: Figure S6 — Phylogenetic profiles and positions in MPD and MNTD coordinates for all modules. [file 1471-2164-14-450-S19.zip › FigureS6/red.pdf]

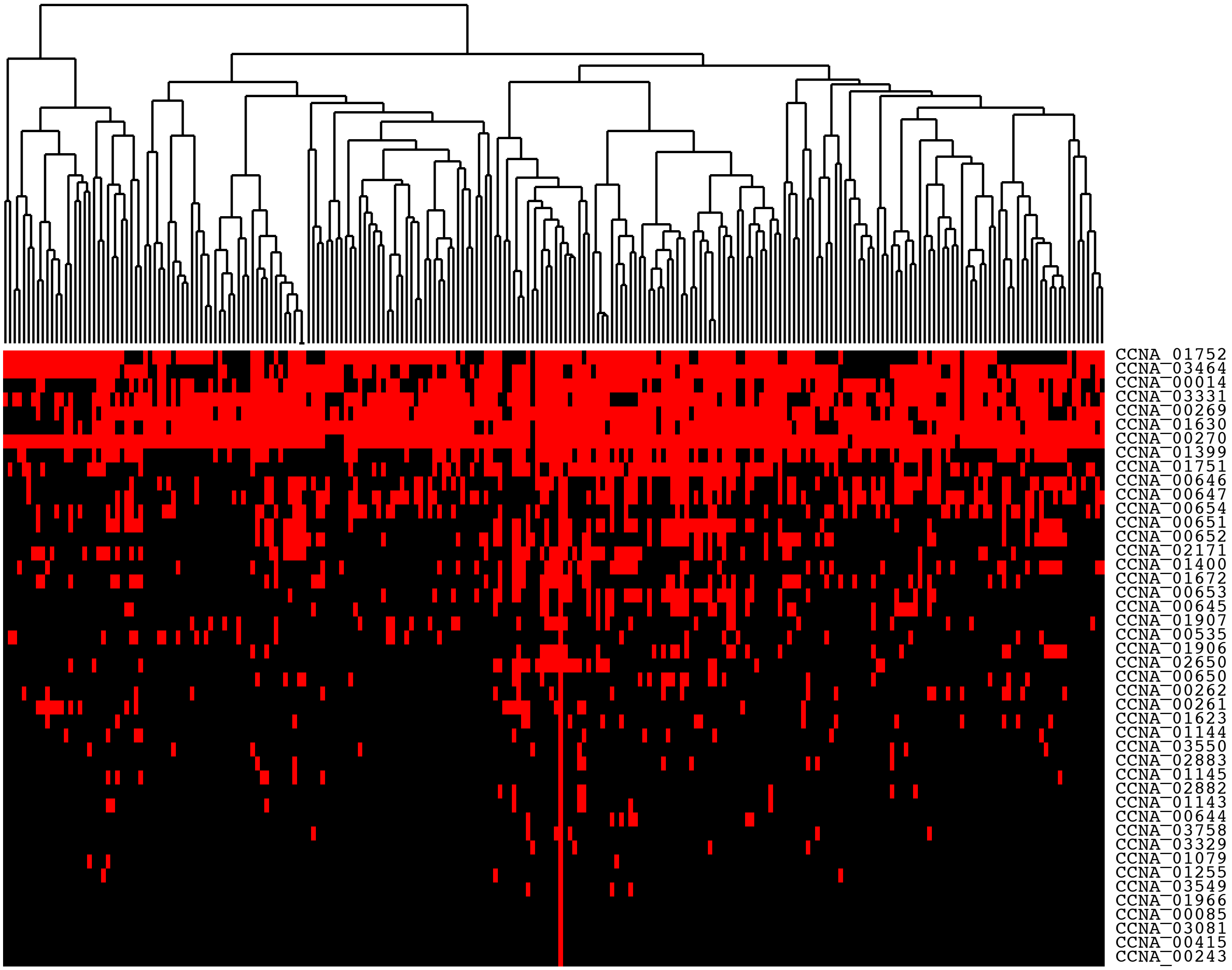
CCNA_01752
CCNA_03464
CCNA_00014
CCNA_03331
CCNA_00269
CCNA_01630
CCNA_00270
CCNA_01399
CCNA_01751
CCNA_00646
CCNA_00647
CCNA_00654
CCNA_00651
CCNA_00652
CCNA_02171
CCNA_01400
CCNA_01672
CCNA_00653
CCNA_00645
CCNA_01907
CCNA_00535
CCNA_01906
CCNA_02650
CCNA_00650
CCNA_00262
CCNA_00261
CCNA_01623
CCNA_01144
CCNA_03550
CCNA_02883
CCNA_01145
CCNA_02882
CCNA_01143
CCNA_00644
CCNA_03758
CCNA_03329
CCNA_01079
CCNA_01255
CCNA_03549
CCNA_01966
CCNA_00085
CCNA_03081
CCNA_00415
CCNA_00243
