# Supplementary material for: Transcriptomic and phylogenetic analysis of a bacterial cell cycle reveals strong associations between gene co-expression and evolution
Source: BMC Genomics. 2013 Jul 5;14:450. doi: 10.1186/1471-2164-14-450 (PMC3829707; doi:10.1186/1471-2164-14-450)
Supplement: Additional file 19: Figure S6 — Phylogenetic profiles and positions in MPD and MNTD coordinates for all modules. [file 1471-2164-14-450-S19.zip › FigureS6/royalblue.pdf]

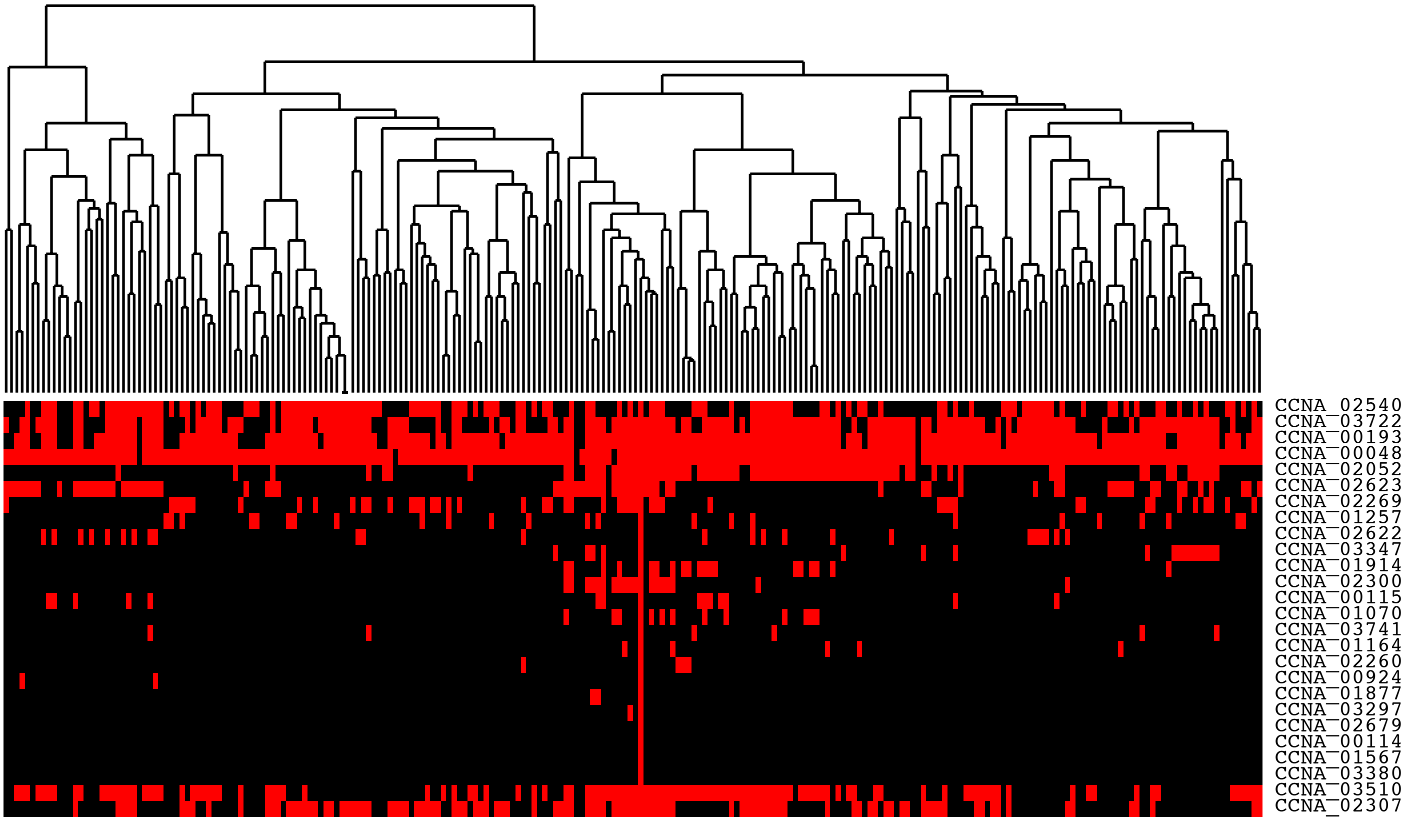
CCNA_02540
CCNA_03722
CCNA_00193
CCNA_00048
CCNA_02052
CCNA_02623
CCNA_02269
CCNA_01257
CCNA_02622
CCNA_03347
CCNA_01914
CCNA_02300
CCNA_00115
CCNA_01070
CCNA_03741
CCNA_01164
CCNA_02260
CCNA_00924
CCNA_01877
CCNA_03297
CCNA_02679
CCNA_00114
CCNA_01567
CCNA_03380
CCNA_03510
CCNA_02307
