# Supplementary material for: Transcriptomic and phylogenetic analysis of a bacterial cell cycle reveals strong associations between gene co-expression and evolution
Source: BMC Genomics. 2013 Jul 5;14:450. doi: 10.1186/1471-2164-14-450 (PMC3829707; doi:10.1186/1471-2164-14-450)
Supplement: Additional file 19: Figure S6 — Phylogenetic profiles and positions in MPD and MNTD coordinates for all modules. [file 1471-2164-14-450-S19.zip › FigureS6/salmon.pdf]

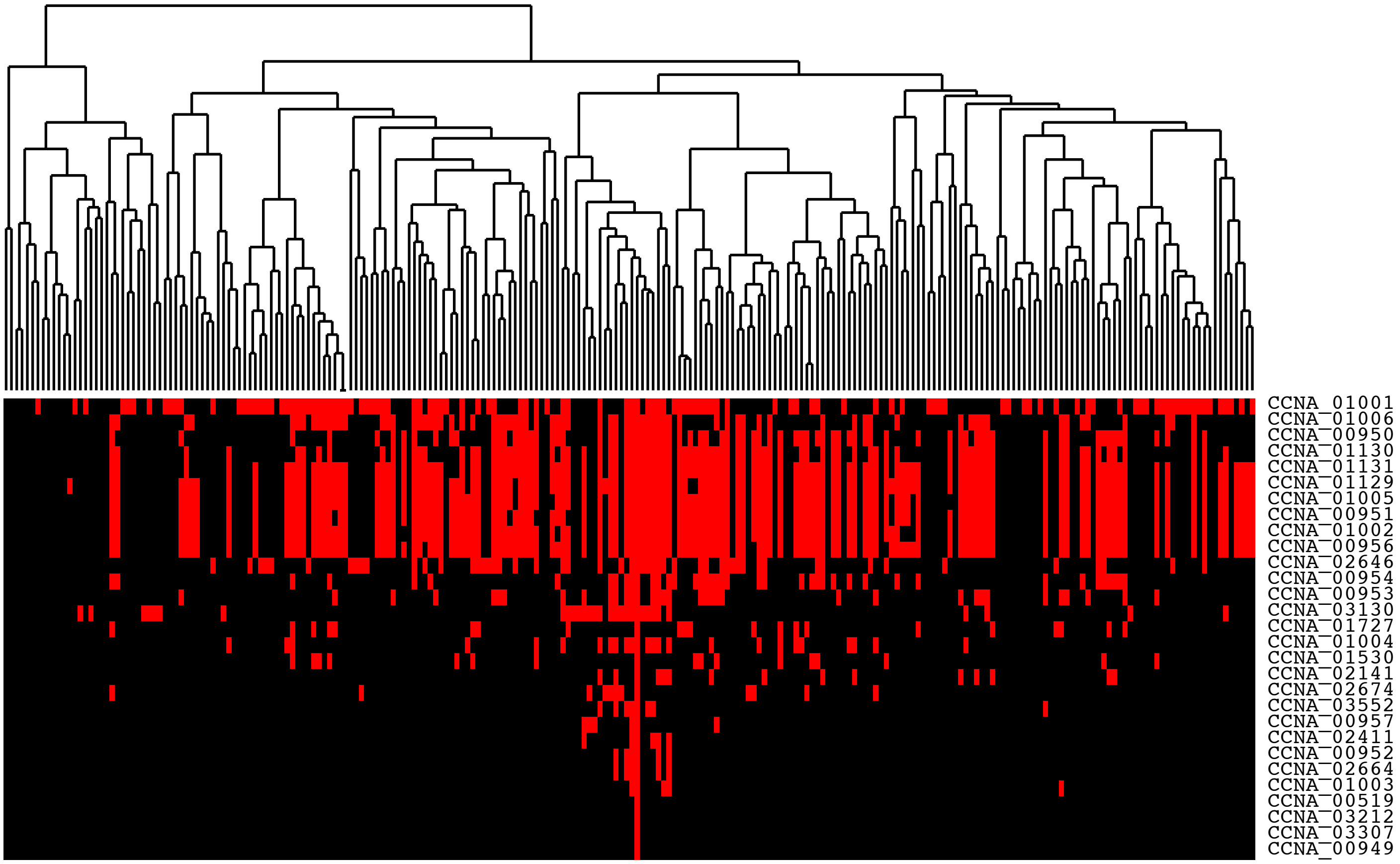
CCNA_01001
CCNA_01006
CCNA_00950
CCNA_01130
CCNA_01131
CCNA_01129
CCNA_01005
CCNA_00951
CCNA_01002
CCNA_00956
CCNA_02646
CCNA_00954
CCNA_00953
CCNA_03130
CCNA_01727
CCNA_01004
CCNA_01530
CCNA_02141
CCNA_02674
CCNA_03552
CCNA_00957
CCNA_02411
CCNA_00952
CCNA_02664
CCNA_01003
CCNA_00519
CCNA_03212
CCNA_03307
CCNA_00949
