# Supplementary material for: Transcriptomic and phylogenetic analysis of a bacterial cell cycle reveals strong associations between gene co-expression and evolution
Source: BMC Genomics. 2013 Jul 5;14:450. doi: 10.1186/1471-2164-14-450 (PMC3829707; doi:10.1186/1471-2164-14-450)
Supplement: Additional file 19: Figure S6 — Phylogenetic profiles and positions in MPD and MNTD coordinates for all modules. [file 1471-2164-14-450-S19.zip › FigureS6/skyblue.pdf]

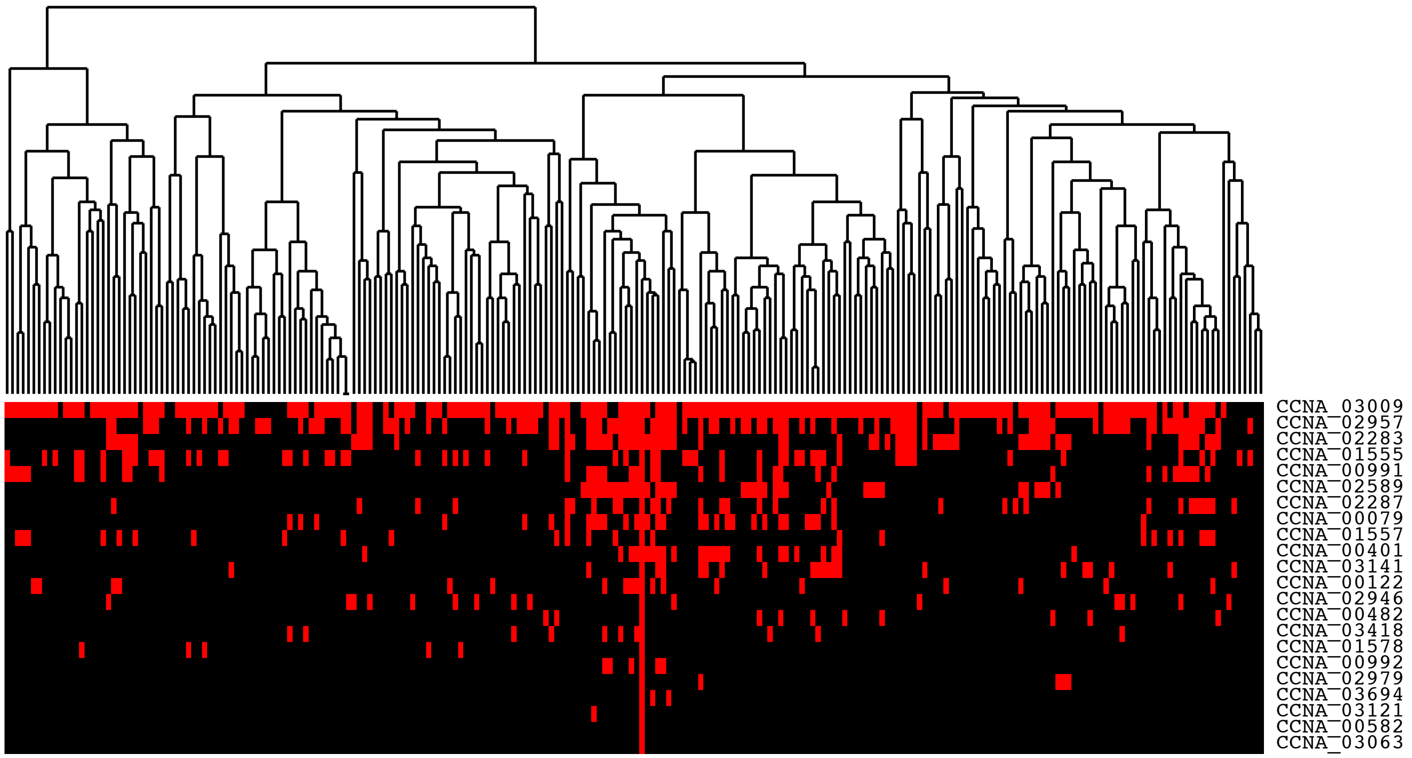
CCNA_03009
CCNA_02957
CCNA_02283
CCNA_01555
CCNA_00991
CCNA_02589
CCNA_02287
CCNA_00079
CCNA_01557
CCNA_00401
CCNA_03141
CCNA_00122
CCNA_02946
CCNA_00482
CCNA_03418
CCNA_01578
CCNA_00992
CCNA_02979
CCNA_03694
CCNA_03121
CCNA_00582
CCNA_03063
