# Supplementary material for: Transcriptomic and phylogenetic analysis of a bacterial cell cycle reveals strong associations between gene co-expression and evolution
Source: BMC Genomics. 2013 Jul 5;14:450. doi: 10.1186/1471-2164-14-450 (PMC3829707; doi:10.1186/1471-2164-14-450)
Supplement: Additional file 19: Figure S6 — Phylogenetic profiles and positions in MPD and MNTD coordinates for all modules. [file 1471-2164-14-450-S19.zip › FigureS6/tan.pdf]

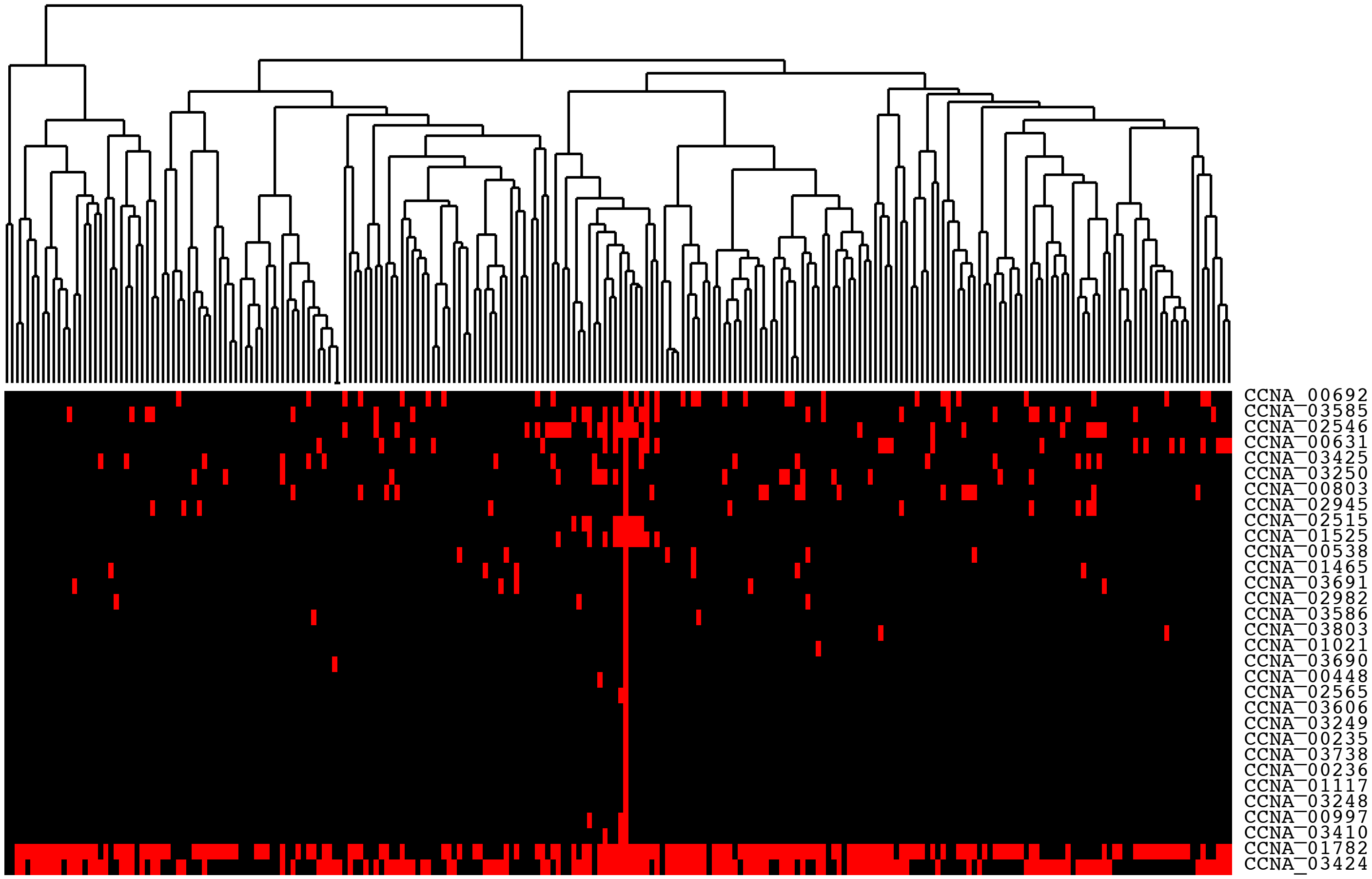
CCNA_00692
CCNA_03585
CCNA_02546
CCNA_00631
CCNA_03425
CCNA_03250
CCNA_00803
CCNA_02945
CCNA_02515
CCNA_01525
CCNA_00538
CCNA_01465
CCNA_03691
CCNA_02982
CCNA_03586
CCNA_03803
CCNA_01021
CCNA_03690
CCNA_00448
CCNA_02565
CCNA_03606
CCNA_03249
CCNA_00235
CCNA_03738
CCNA_00236
CCNA_01117
CCNA_03248
CCNA_00997
CCNA_03410
CCNA_01782
CCNA_03424
