# Supplementary material for: Transcriptomic and phylogenetic analysis of a bacterial cell cycle reveals strong associations between gene co-expression and evolution
Source: BMC Genomics. 2013 Jul 5;14:450. doi: 10.1186/1471-2164-14-450 (PMC3829707; doi:10.1186/1471-2164-14-450)
Supplement: Additional file 19: Figure S6 — Phylogenetic profiles and positions in MPD and MNTD coordinates for all modules. [file 1471-2164-14-450-S19.zip › FigureS6/turquoise.pdf]

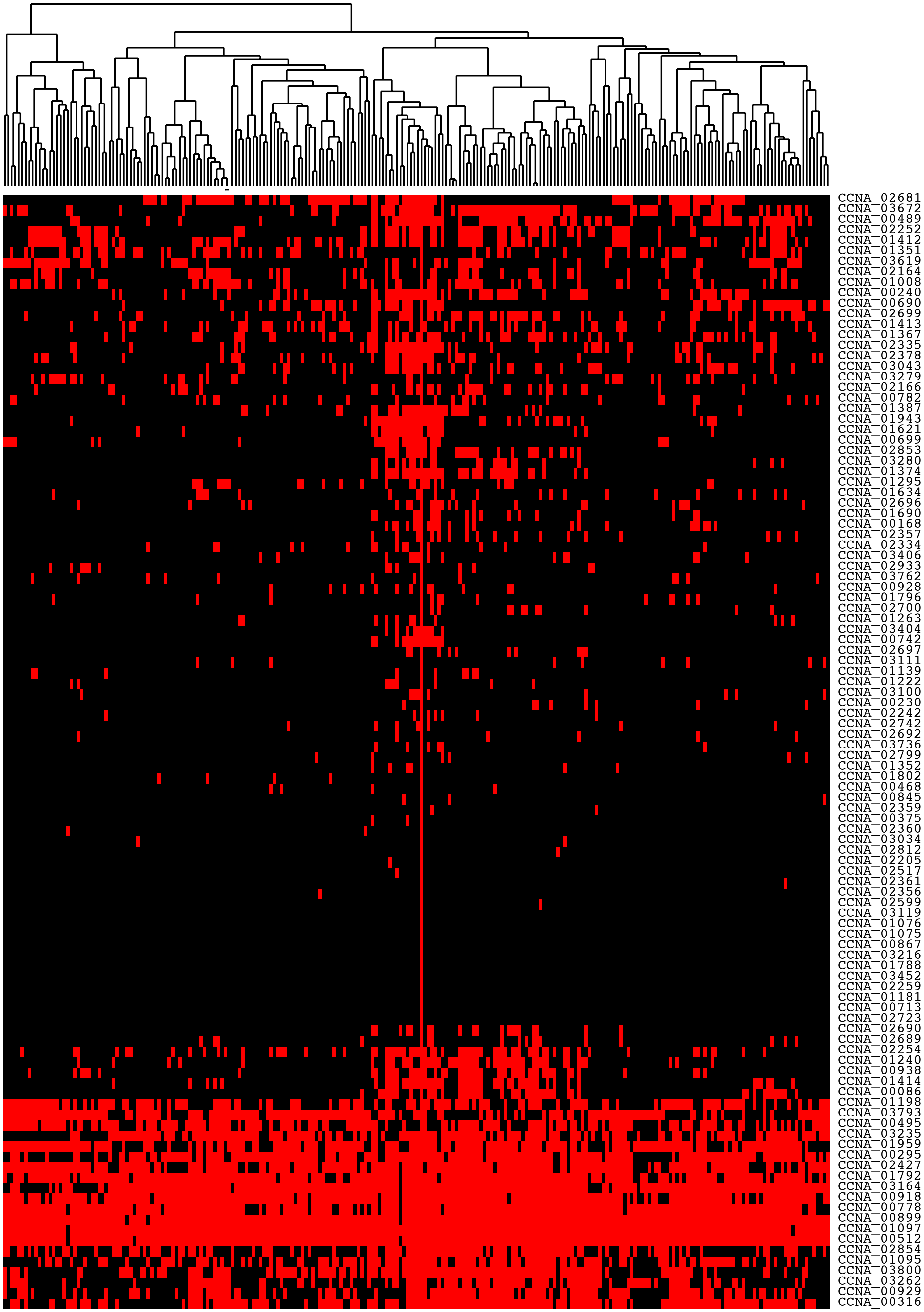
CCNA_02681
CCNA_03672
CCNA_00489
CCNA_02252
CCNA_01412
CCNA_01351
CCNA_03619
CCNA_02164
CCNA_01008
CCNA_00240
CCNA_00690
CCNA_02699
CCNA_01413
CCNA_01367
CCNA_02335
CCNA_02378
CCNA_03043
CCNA_03279
CCNA_02166
CCNA_00782
CCNA_01387
CCNA_01943
CCNA_01621
CCNA_00699
CCNA_02853
CCNA_03280
CCNA_01374
CCNA_01295
CCNA_01634
CCNA_02696
CCNA_01690
CCNA_00168
CCNA_02357
CCNA_02334
CCNA_03406
CCNA_02933
CCNA_03762
CCNA_00928
CCNA_01796
CCNA_02700
CCNA_01263
CCNA_03404
CCNA_00742
CCNA_02697
CCNA_03111
CCNA_01139
CCNA_01222
CCNA_03100
CCNA_00230
CCNA_02242
CCNA_02742
CCNA_02692
CCNA_03736
CCNA_02799
CCNA_01352
CCNA_01802
CCNA_00468
CCNA_00845
CCNA_02359
CCNA_00375
CCNA_02360
CCNA_03034
CCNA_02812
CCNA_02205
CCNA_02517
CCNA_02361
CCNA_02356
CCNA_02599
CCNA_03119
CCNA_01076
CCNA_01075
CCNA_00867
CCNA_03216
CCNA_01788
CCNA_03452
CCNA_02259
CCNA_01181
CCNA_00713
CCNA_02723
CCNA_02690
CCNA_02689
CCNA_02254
CCNA_01240
CCNA_00938
CCNA_01414
CCNA_00086
CCNA_01198
CCNA_03793
CCNA_00495
CCNA_03235
CCNA_01959
CCNA_00295
CCNA_02427
CCNA_01792
CCNA_03164
CCNA_00918
CCNA_00778
CCNA_00899
CCNA_01097
CCNA_00512
CCNA_02854
CCNA_01095
CCNA_03800
CCNA_03262
CCNA_00929
CCNA_00316
