# Supplementary material for: Transcriptomic and phylogenetic analysis of a bacterial cell cycle reveals strong associations between gene co-expression and evolution
Source: BMC Genomics. 2013 Jul 5;14:450. doi: 10.1186/1471-2164-14-450 (PMC3829707; doi:10.1186/1471-2164-14-450)
Supplement: Additional file 19: Figure S6 — Phylogenetic profiles and positions in MPD and MNTD coordinates for all modules. [file 1471-2164-14-450-S19.zip › FigureS6/white.pdf]

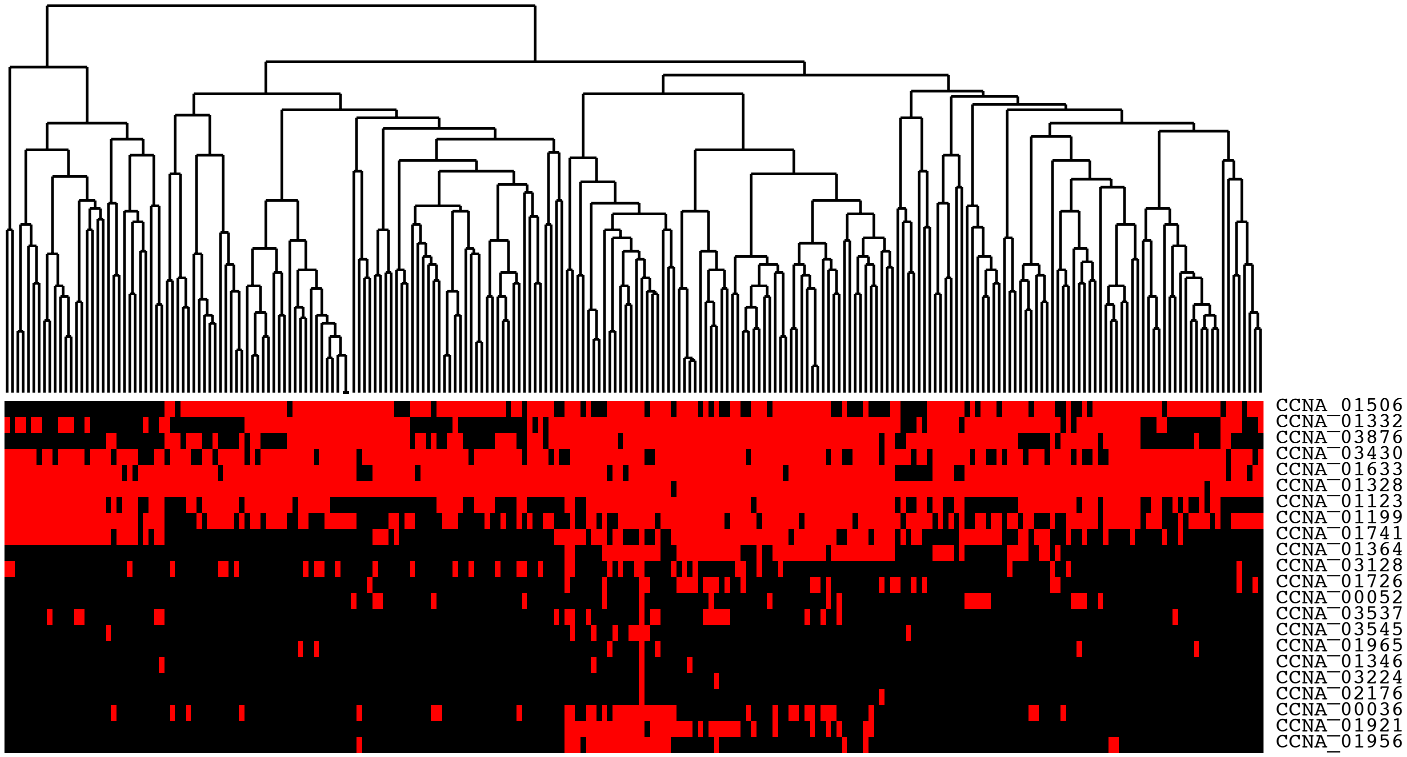
CCNA_01506
CCNA_01332
CCNA_03876
CCNA_03430
CCNA_01633
CCNA_01328
CCNA_01123
CCNA_01199
CCNA_01741
CCNA_01364
CCNA_03128
CCNA_01726
CCNA_00052
CCNA_03537
CCNA_03545
CCNA_01965
CCNA_01346
CCNA_03224
CCNA_02176
CCNA_00036
CCNA_01921
CCNA_01956
