# Supplementary material for: Transcriptomic and phylogenetic analysis of a bacterial cell cycle reveals strong associations between gene co-expression and evolution
Source: BMC Genomics. 2013 Jul 5;14:450. doi: 10.1186/1471-2164-14-450 (PMC3829707; doi:10.1186/1471-2164-14-450)
Supplement: Additional file 19: Figure S6 — Phylogenetic profiles and positions in MPD and MNTD coordinates for all modules. [file 1471-2164-14-450-S19.zip › FigureS6/yellow.pdf]

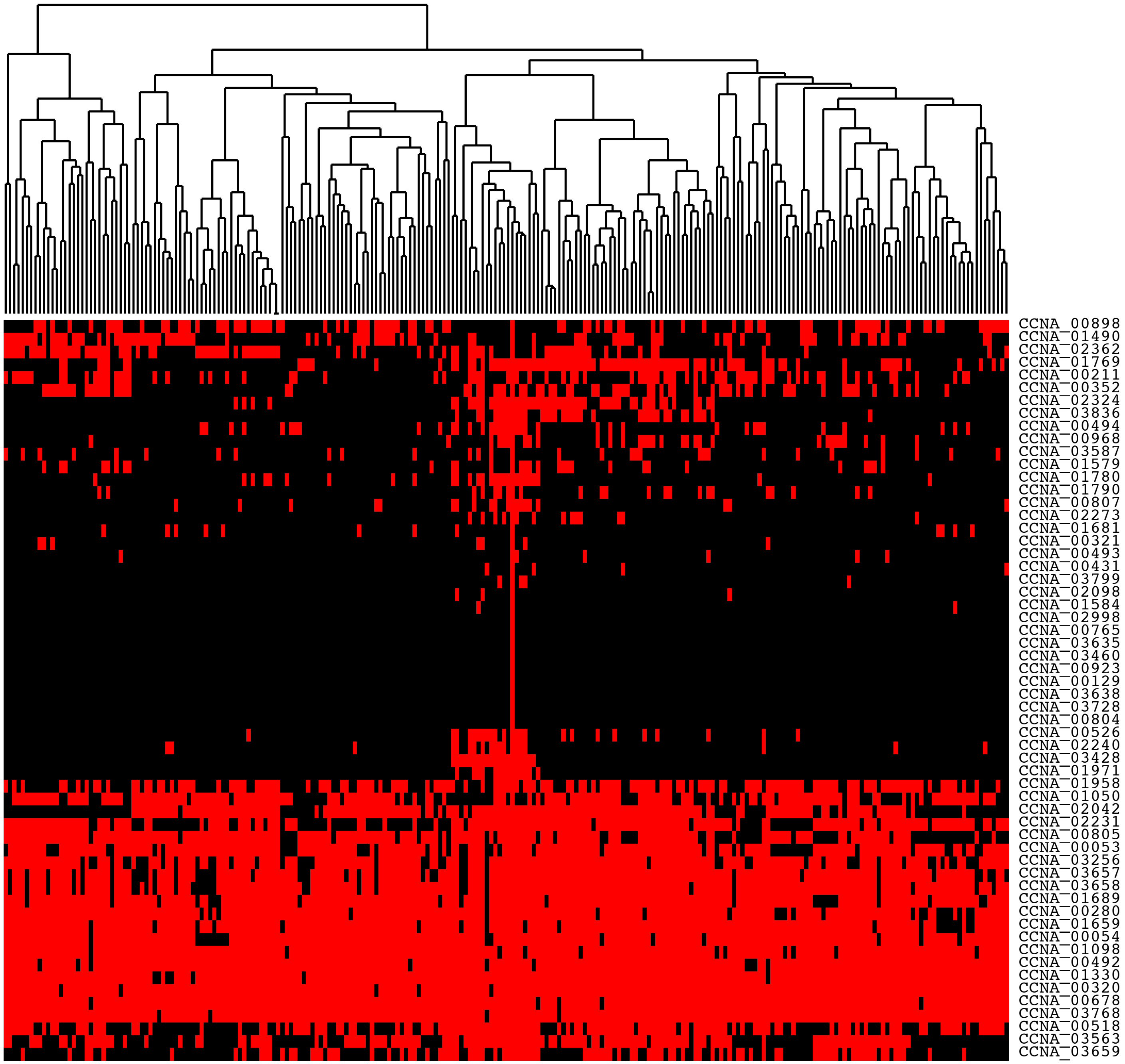
CCNA_00898
CCNA_01490
CCNA_02362
CCNA_01769
CCNA_00211
CCNA_00352
CCNA_02324
CCNA_03836
CCNA_00494
CCNA_00968
CCNA_03587
CCNA_01579
CCNA_01780
CCNA_01790
CCNA_00807
CCNA_02273
CCNA_01681
CCNA_00321
CCNA_00493
CCNA_00431
CCNA_03799
CCNA_02098
CCNA_01584
CCNA_02998
CCNA_00765
CCNA_03635
CCNA_03460
CCNA_00923
CCNA_00129
CCNA_03638
CCNA_03728
CCNA_00804
CCNA_00526
CCNA_02240
CCNA_03428
CCNA_01971
CCNA_01958
CCNA_01050
CCNA_02042
CCNA_02231
CCNA_00805
CCNA_00053
CCNA_03256
CCNA_03657
CCNA_03658
CCNA_01689
CCNA_00280
CCNA_01659
CCNA_00054
CCNA_01098
CCNA_00492
CCNA_01330
CCNA_00320
CCNA_00678
CCNA_03768
CCNA_00518
CCNA_03563
CCNA_03659
