# Supplementary figures and images for: Transcriptomic and phylogenetic analysis of a bacterial cell cycle reveals strong associations between gene co-expression and evolution (part 4 of 4)
Source: BMC Genomics. 2013 Jul 5;14:450. doi: 10.1186/1471-2164-14-450 (PMC3829707; doi:10.1186/1471-2164-14-450)

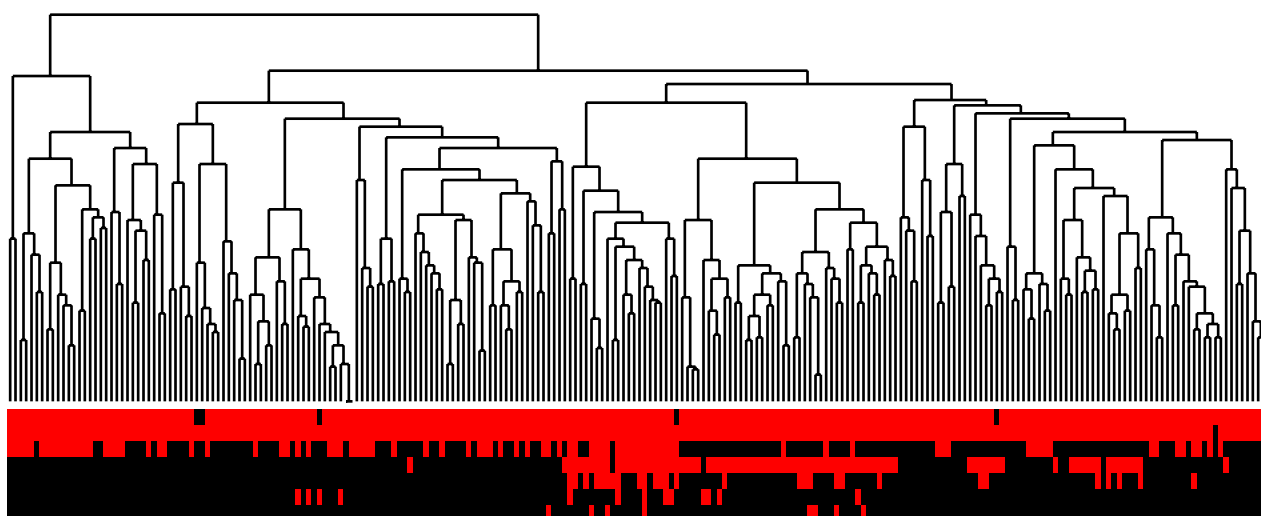

CCNA\_03352  
CCNA\_00496  
CCNA\_03028  
CCNA\_00806  
CCNA\_01583  
CCNA\_00249  
CCNA\_03666

Supplement: Additional file 19: Figure S6 — Phylogenetic profiles and positions in MPD and MNTD coordinates for all modules. [file 1471-2164-14-450-S19.zip › FigureS6/lightsteelblue.pdf]

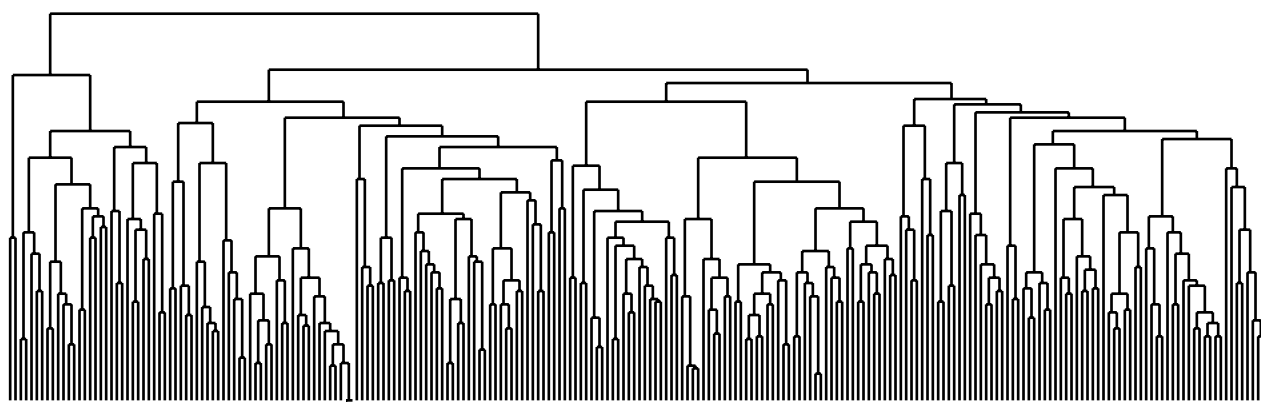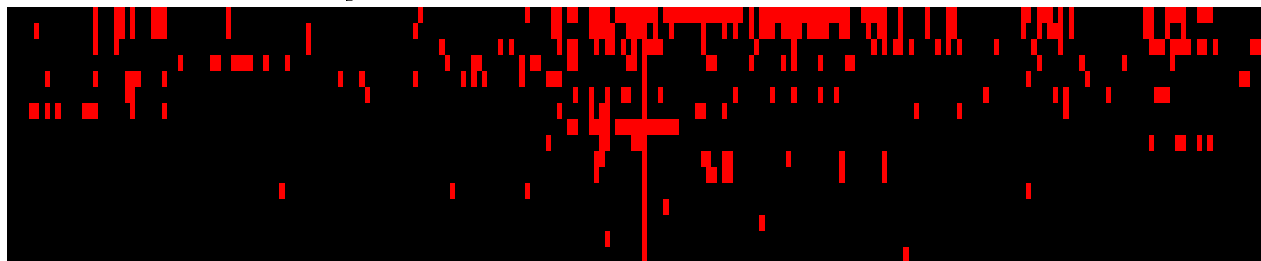

CCNA\_03412  
CCNA\_03413  
CCNA\_03847  
CCNA\_03379  
CCNA\_03064  
CCNA\_00192  
CCNA\_01256  
CCNA\_02430  
CCNA\_02407  
CCNA\_01194  
CCNA\_00455  
CCNA\_01041  
CCNA\_02912  
CCNA\_02110  
CCNA\_00111  
CCNA\_00986

Supplement: Additional file 19: Figure S6 — Phylogenetic profiles and positions in MPD and MNTD coordinates for all modules. [file 1471-2164-14-450-S19.zip › FigureS6/lightsteelblue1.pdf]

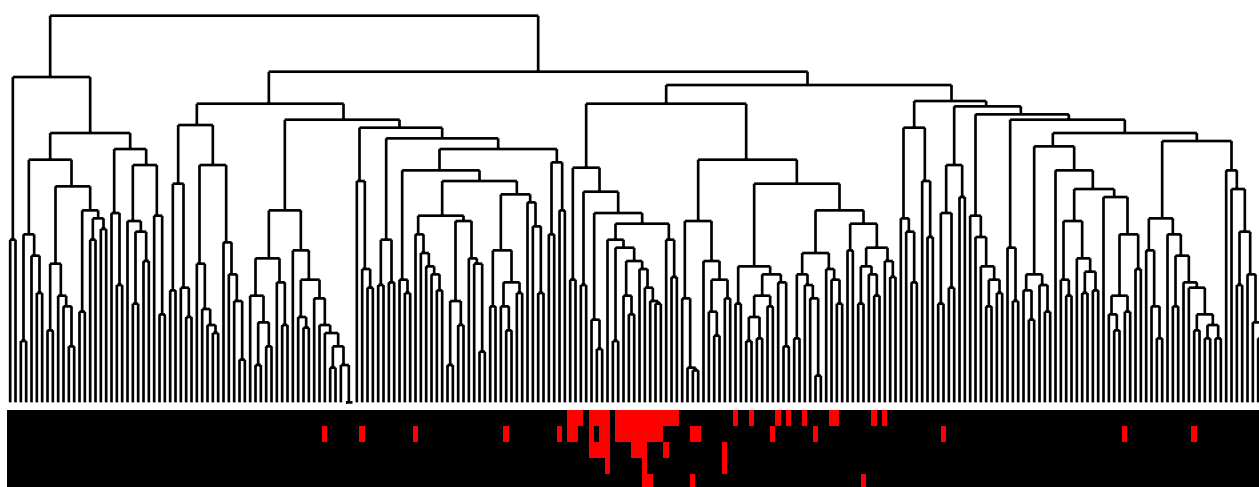

CCNA\_01783  
CCNA\_00410  
CCNA\_02169  
CCNA\_01344  
CCNA\_02174

Supplement: Additional file 19: Figure S6 — Phylogenetic profiles and positions in MPD and MNTD coordinates for all modules. [file 1471-2164-14-450-S19.zip › FigureS6/maroon.pdf]

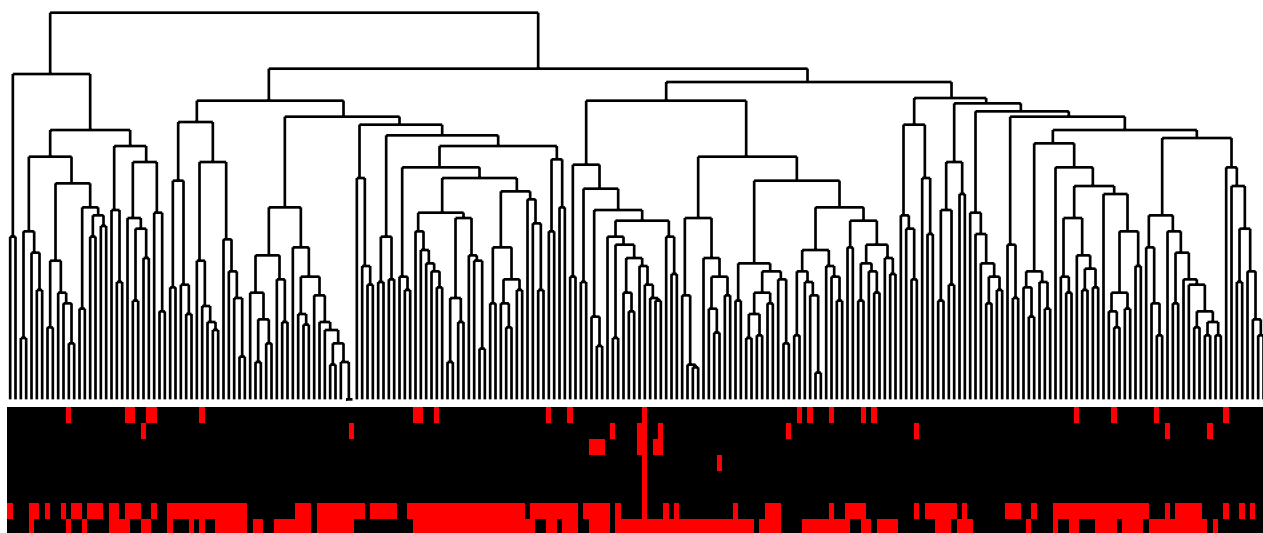

Supplement: Additional file 19: Figure S6 — Phylogenetic profiles and positions in MPD and MNTD coordinates for all modules. [file 1471-2164-14-450-S19.zip › FigureS6/mediumorchid.pdf]

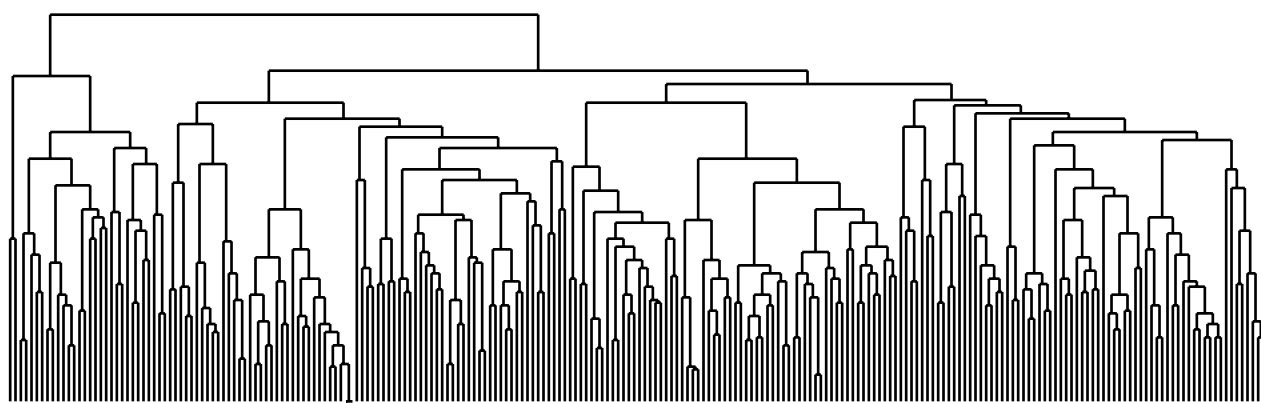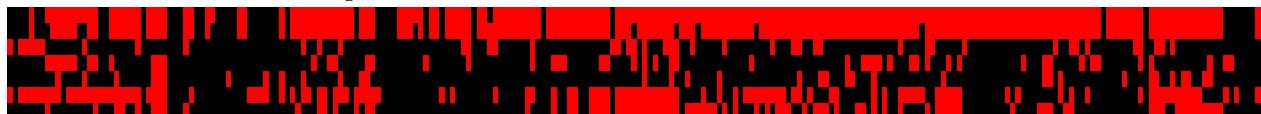

CCNA\_01409  
CCNA\_00070  
CCNA\_03840  
CCNA\_02094  
CCNA\_01408  
CCNA\_00766  
CCNA\_01492

Supplement: Additional file 19: Figure S6 — Phylogenetic profiles and positions in MPD and MNTD coordinates for all modules. [file 1471-2164-14-450-S19.zip › FigureS6/mediumpurple2.pdf]

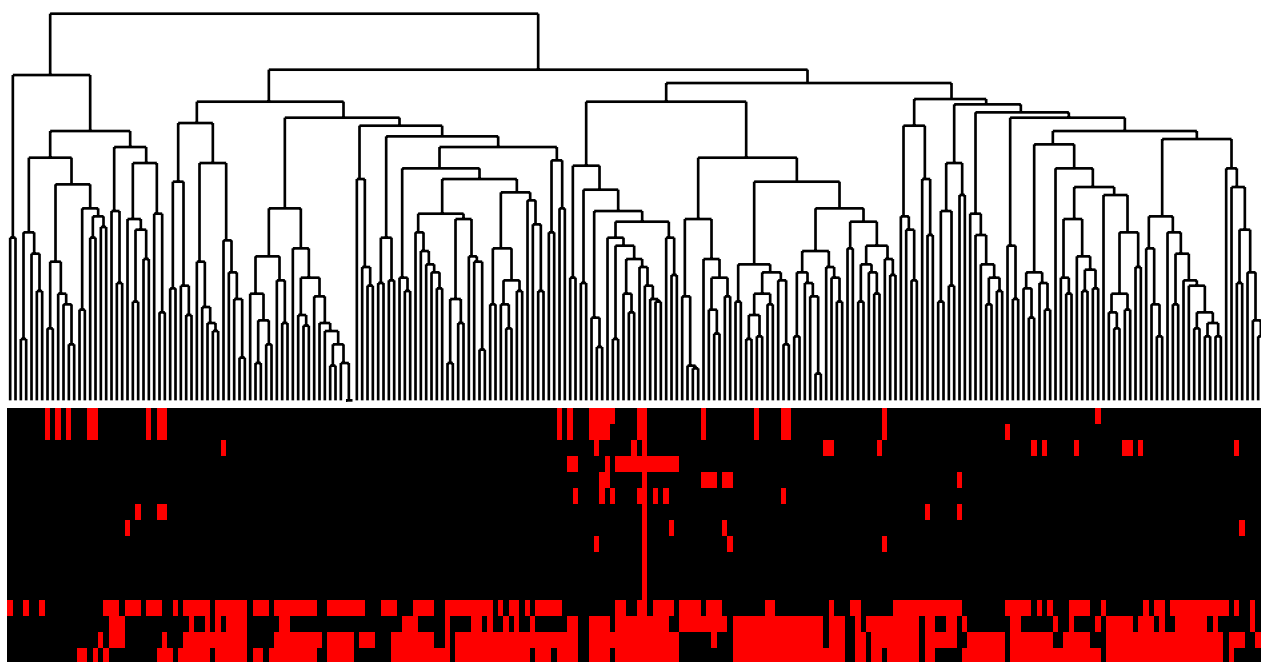

Supplement: Additional file 19: Figure S6 — Phylogenetic profiles and positions in MPD and MNTD coordinates for all modules. [file 1471-2164-14-450-S19.zip › FigureS6/mediumpurple3.pdf]

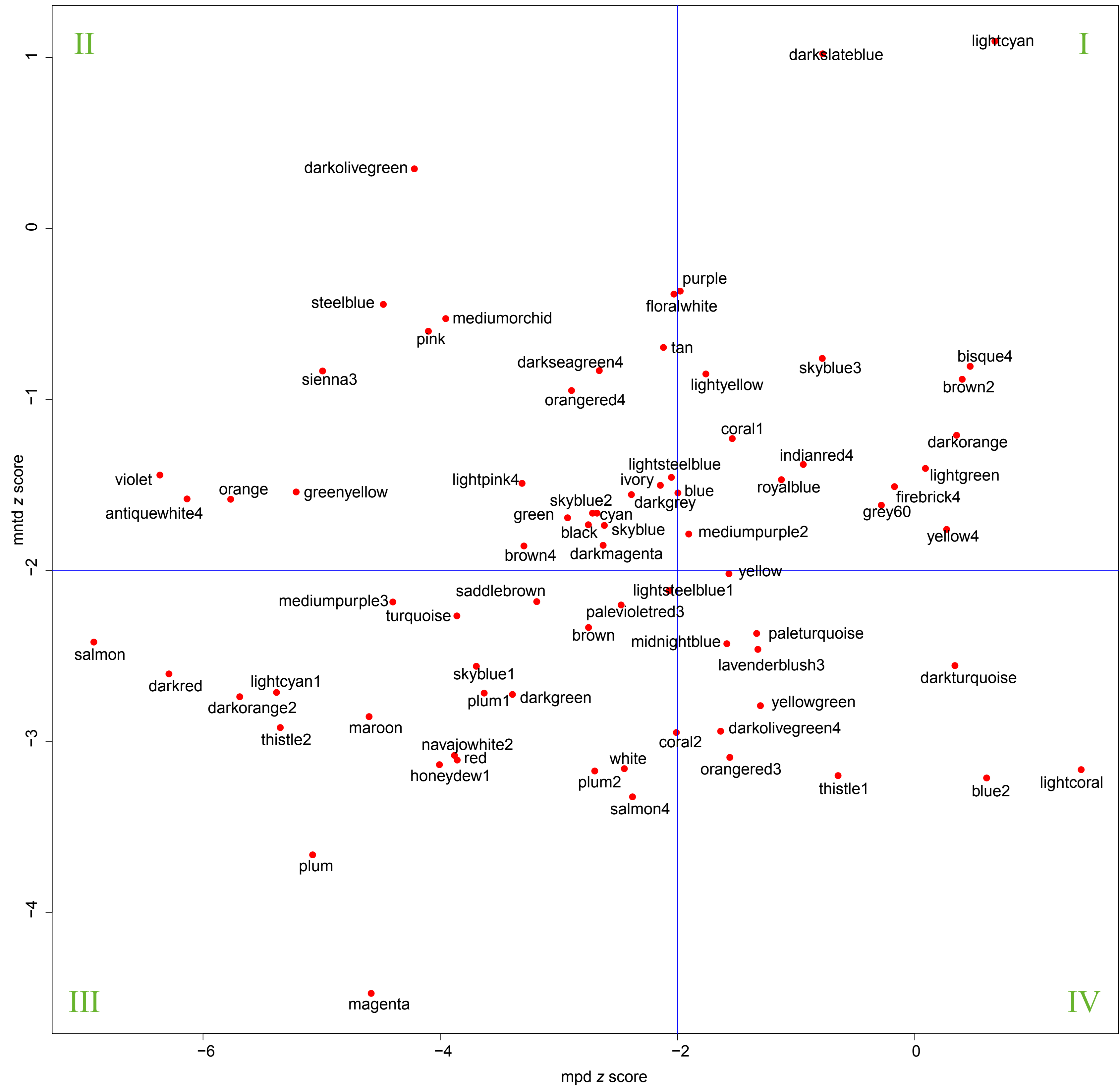

Supplement: Additional file 19: Figure S6 — Phylogenetic profiles and positions in MPD and MNTD coordinates for all modules. [file 1471-2164-14-450-S19.zip › FigureS6/MM_Coordinates.pdf]

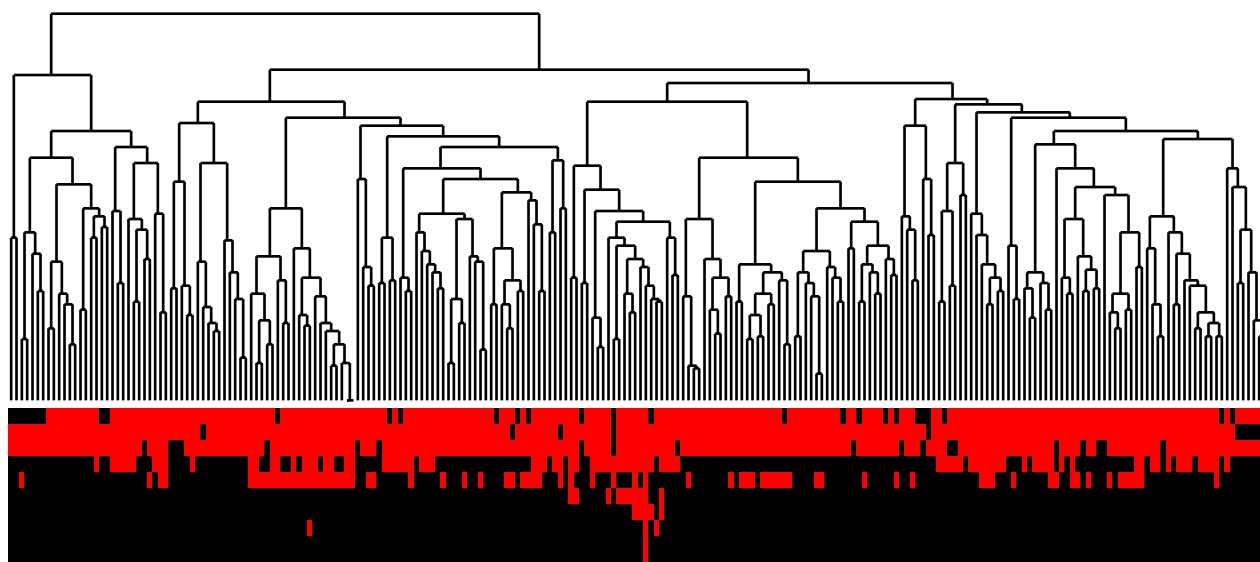

Supplement: Additional file 19: Figure S6 — Phylogenetic profiles and positions in MPD and MNTD coordinates for all modules. [file 1471-2164-14-450-S19.zip › FigureS6/navajowhite2.pdf]

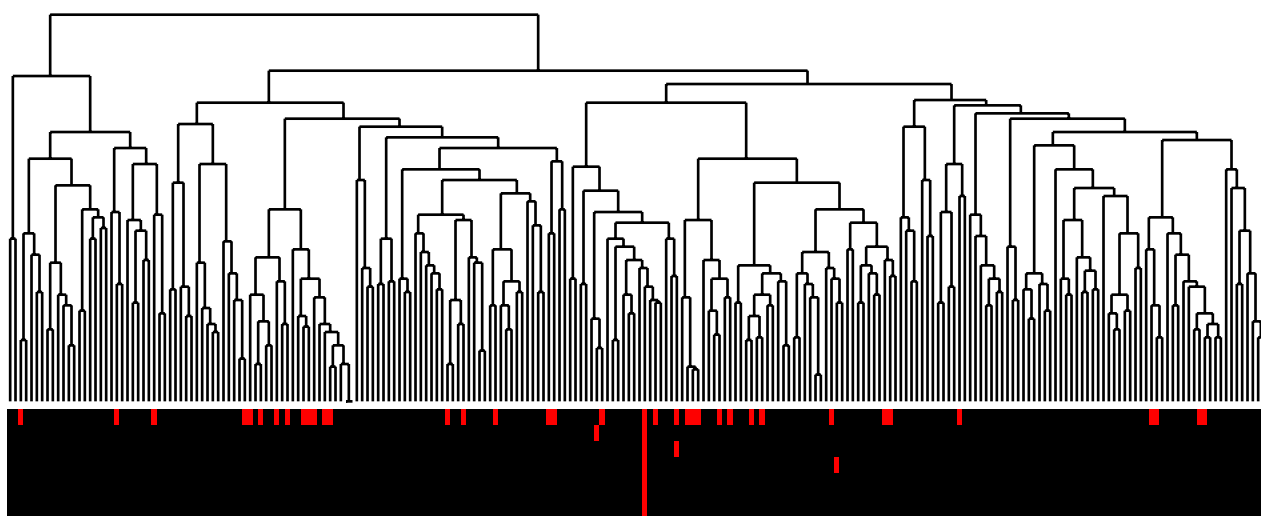

Supplement: Additional file 19: Figure S6 — Phylogenetic profiles and positions in MPD and MNTD coordinates for all modules. [file 1471-2164-14-450-S19.zip › FigureS6/orangered3.pdf]

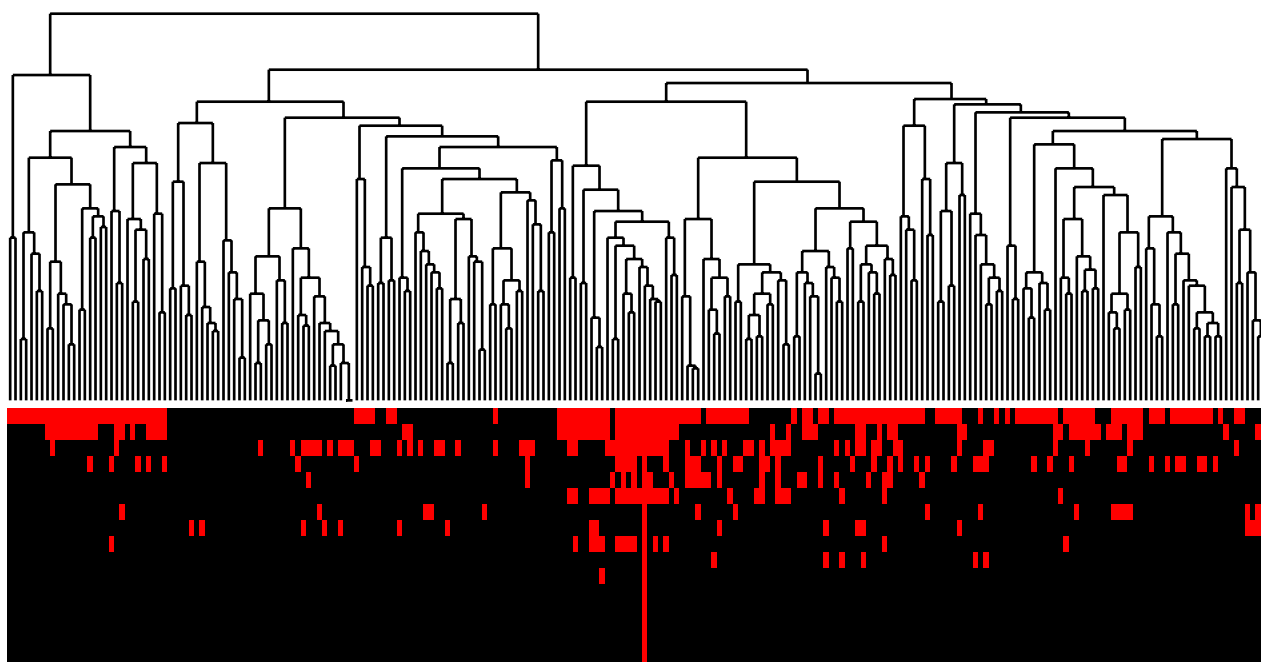

CCNA\_02535  
CCNA\_01817  
CCNA\_01818  
CCNA\_01773  
CCNA\_01848  
CCNA\_00250  
CCNA\_00835  
CCNA\_00834  
CCNA\_03110  
CCNA\_02931  
CCNA\_01186  
CCNA\_02409  
CCNA\_00438  
CCNA\_01768  
CCNA\_02061  
CCNA\_03268

Supplement: Additional file 19: Figure S6 — Phylogenetic profiles and positions in MPD and MNTD coordinates for all modules. [file 1471-2164-14-450-S19.zip › FigureS6/orangered4.pdf]

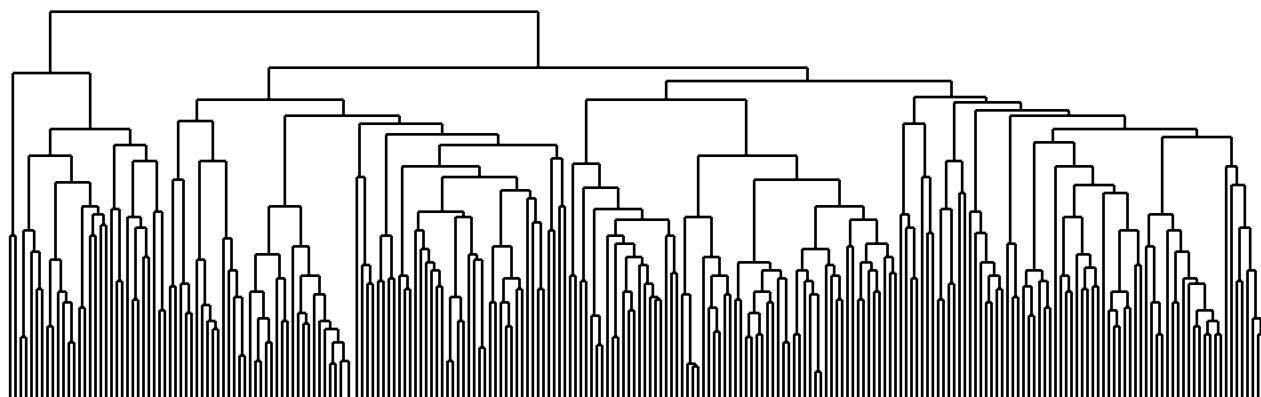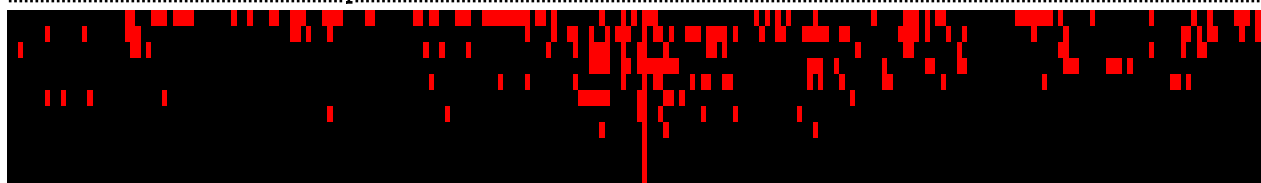

CCNA\_00738  
CCNA\_00825  
CCNA\_03589  
CCNA\_02225  
CCNA\_02447  
CCNA\_03607  
CCNA\_03330  
CCNA\_01218  
CCNA\_03015  
CCNA\_02986  
CCNA\_02523

Supplement: Additional file 19: Figure S6 — Phylogenetic profiles and positions in MPD and MNTD coordinates for all modules. [file 1471-2164-14-450-S19.zip › FigureS6/palevioletred3.pdf]

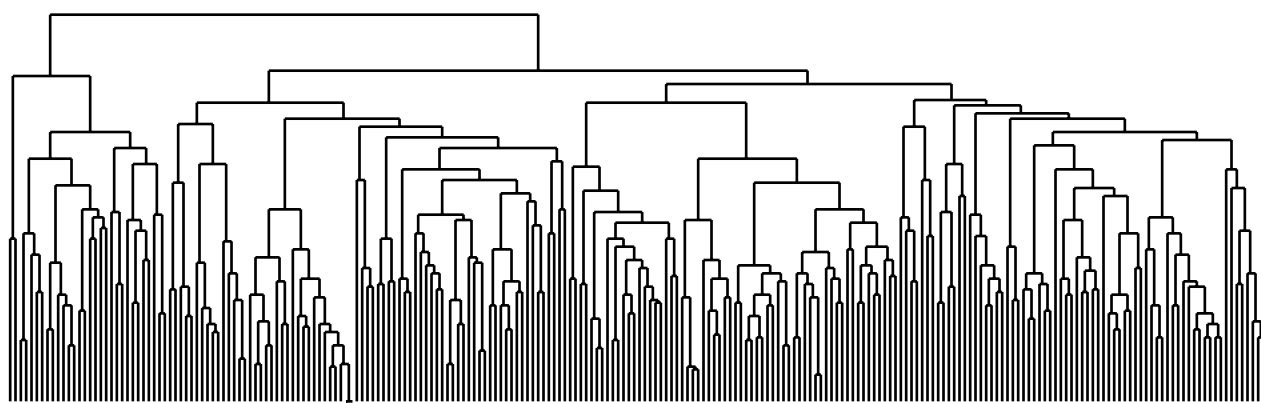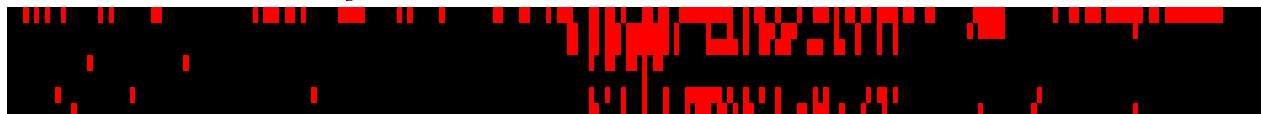

CCNA\_00801  
CCNA\_01468  
CCNA\_01470  
CCNA\_01469  
CCNA\_00797  
CCNA\_00802  
CCNA\_01585

Supplement: Additional file 19: Figure S6 — Phylogenetic profiles and positions in MPD and MNTD coordinates for all modules. [file 1471-2164-14-450-S19.zip › FigureS6/plum.pdf]

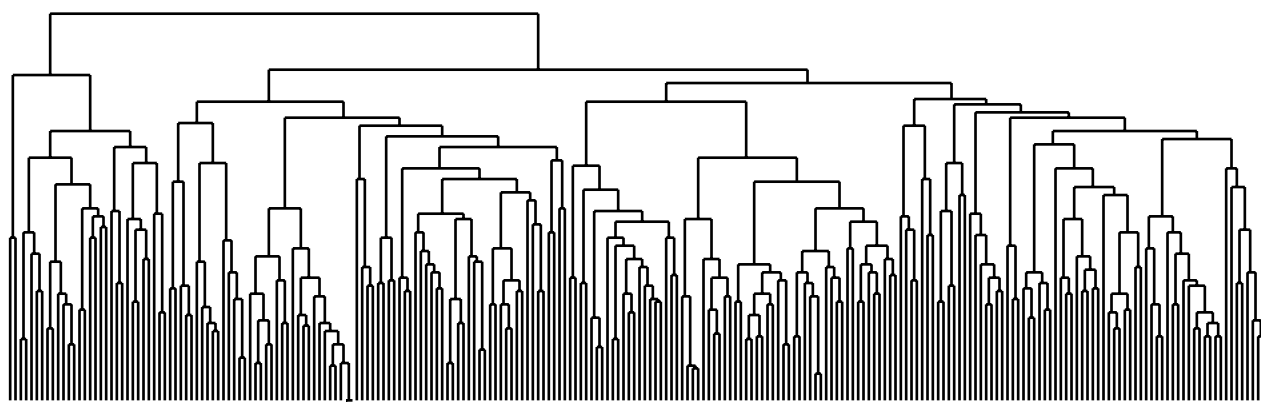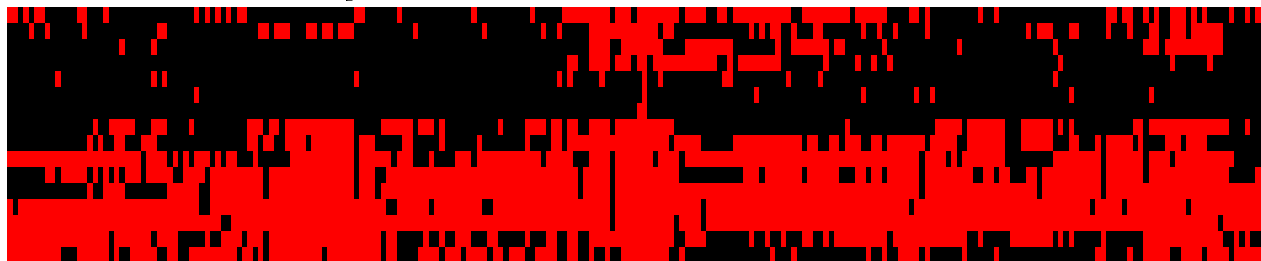

CCNA\_01811  
CCNA\_00987  
CCNA\_03764  
CCNA\_02182  
CCNA\_01928  
CCNA\_00161  
CCNA\_01418  
CCNA\_02586  
CCNA\_03833  
CCNA\_02247  
CCNA\_02526  
CCNA\_01775  
CCNA\_01970  
CCNA\_01969  
CCNA\_01940  
CCNA\_03628

Supplement: Additional file 19: Figure S6 — Phylogenetic profiles and positions in MPD and MNTD coordinates for all modules. [file 1471-2164-14-450-S19.zip › FigureS6/plum1.pdf]

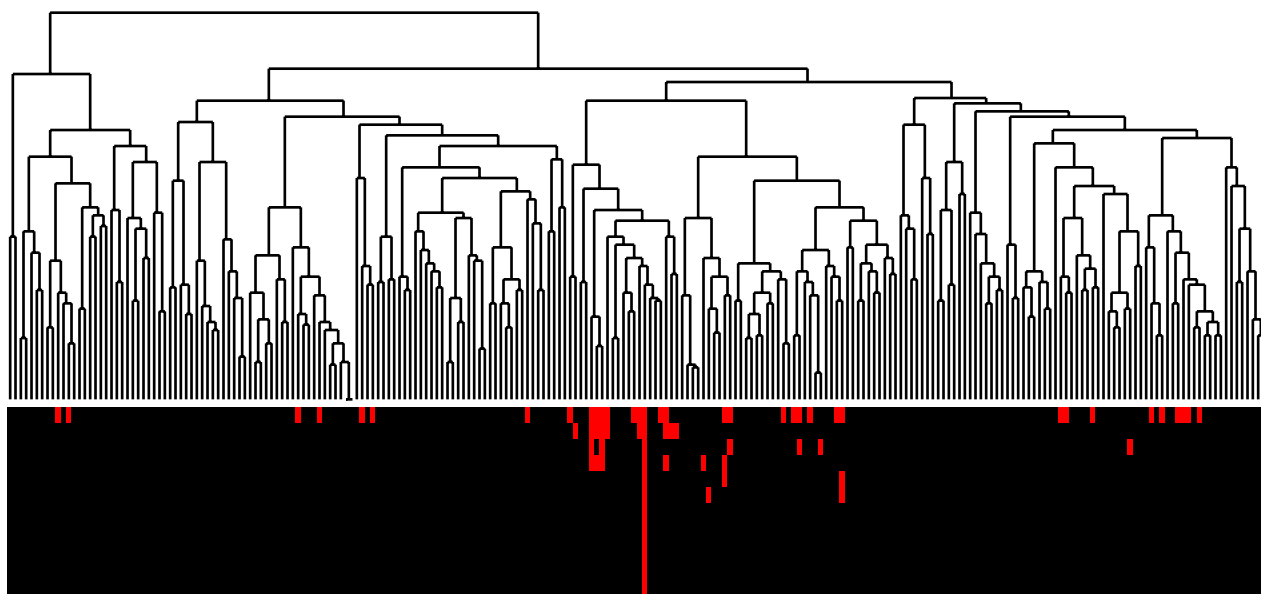

CCNA\_00077  
CCNA\_03405  
CCNA\_03444  
CCNA\_03574  
CCNA\_01023  
CCNA\_02895  
CCNA\_03448  
CCNA\_02937  
CCNA\_00585  
CCNA\_03487  
CCNA\_03266  
CCNA\_02433

Supplement: Additional file 19: Figure S6 — Phylogenetic profiles and positions in MPD and MNTD coordinates for all modules. [file 1471-2164-14-450-S19.zip › FigureS6/plum2.pdf]

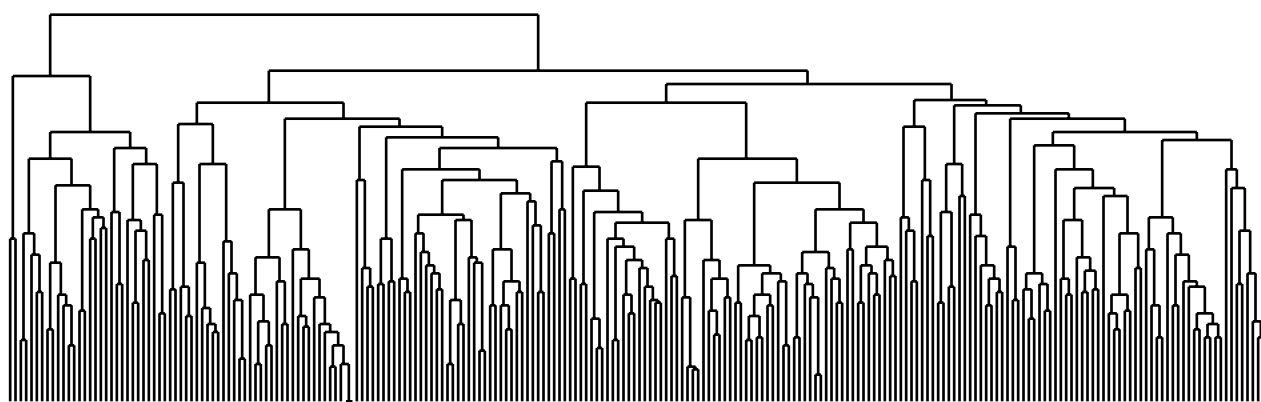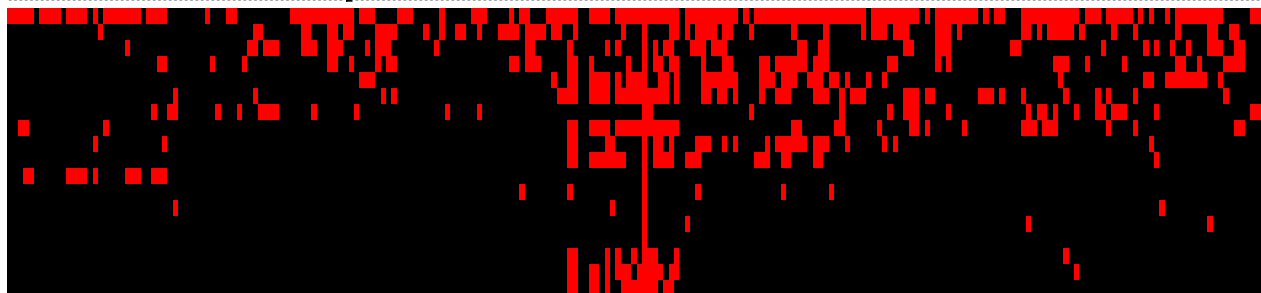

CCNA\_03696  
CCNA\_02687  
CCNA\_02698  
CCNA\_02701  
CCNA\_02250  
CCNA\_00153  
CCNA\_02173  
CCNA\_03849  
CCNA\_02688  
CCNA\_00265  
CCNA\_00858  
CCNA\_03579  
CCNA\_00897  
CCNA\_01436  
CCNA\_03169  
CCNA\_01546  
CCNA\_00042  
CCNA\_02249

Supplement: Additional file 19: Figure S6 — Phylogenetic profiles and positions in MPD and MNTD coordinates for all modules. [file 1471-2164-14-450-S19.zip › FigureS6/saddlebrown.pdf]

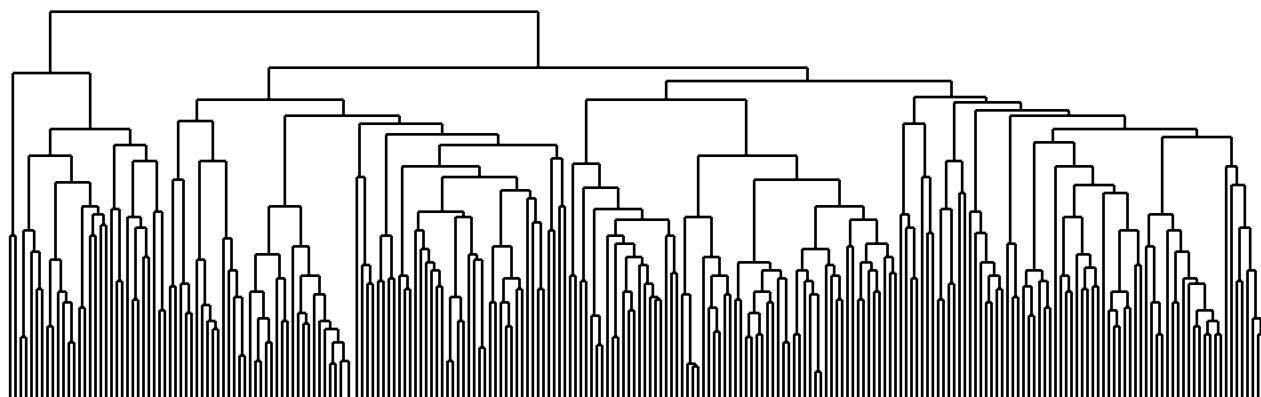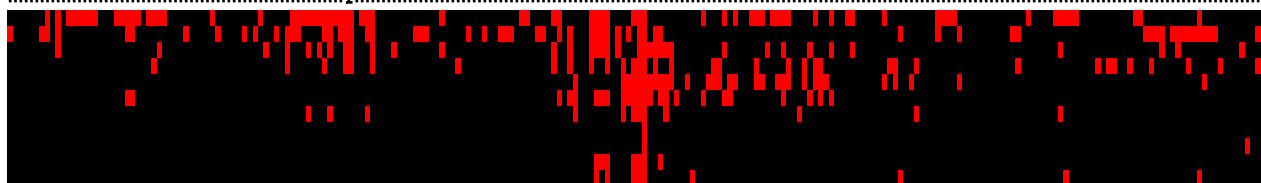

CCNA\_03575  
CCNA\_03124  
CCNA\_00246  
CCNA\_02216  
CCNA\_03245  
CCNA\_00095  
CCNA\_02716  
CCNA\_01092  
CCNA\_01631  
CCNA\_02128  
CCNA\_00244

Supplement: Additional file 19: Figure S6 — Phylogenetic profiles and positions in MPD and MNTD coordinates for all modules. [file 1471-2164-14-450-S19.zip › FigureS6/salmon4.pdf]

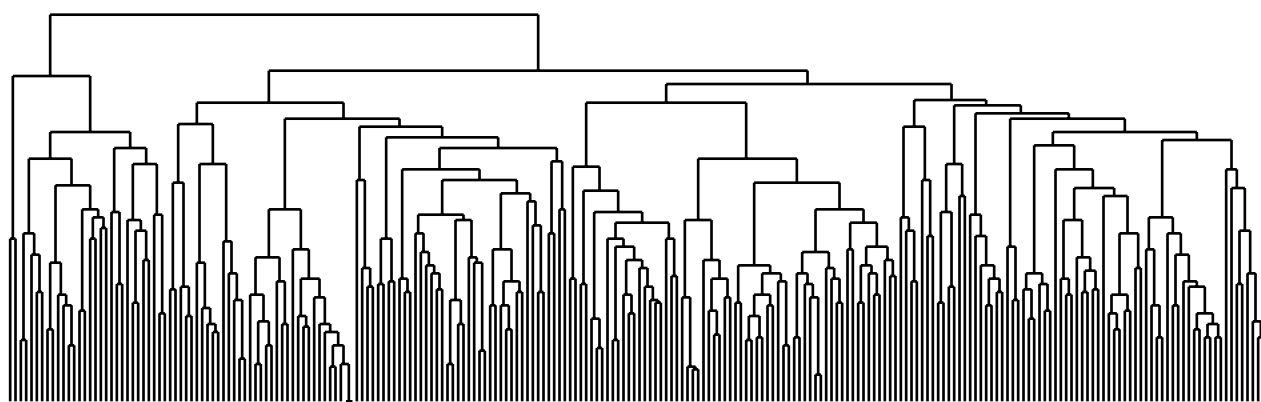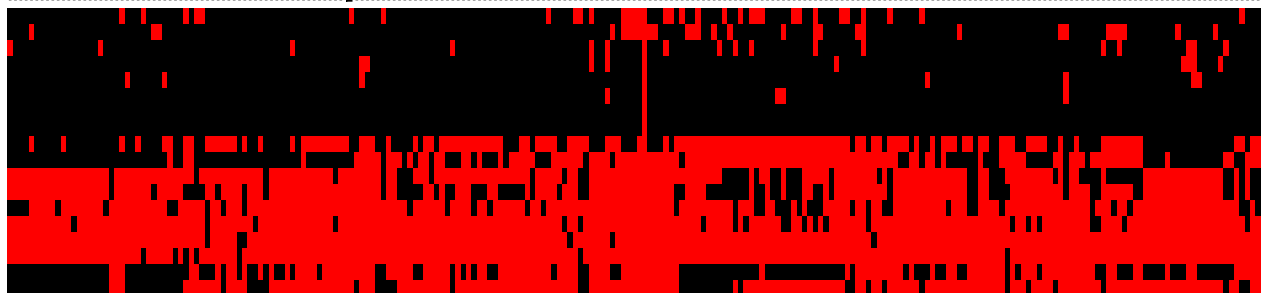

CCNA\_03533  
CCNA\_03260  
CCNA\_01210  
CCNA\_01937  
CCNA\_01939  
CCNA\_03362  
CCNA\_01565  
CCNA\_02025  
CCNA\_01942  
CCNA\_00063  
CCNA\_01936  
CCNA\_01938  
CCNA\_00062  
CCNA\_01950  
CCNA\_01254  
CCNA\_01791  
CCNA\_03704  
CCNA\_03850

Supplement: Additional file 19: Figure S6 — Phylogenetic profiles and positions in MPD and MNTD coordinates for all modules. [file 1471-2164-14-450-S19.zip › FigureS6/sienna3.pdf]

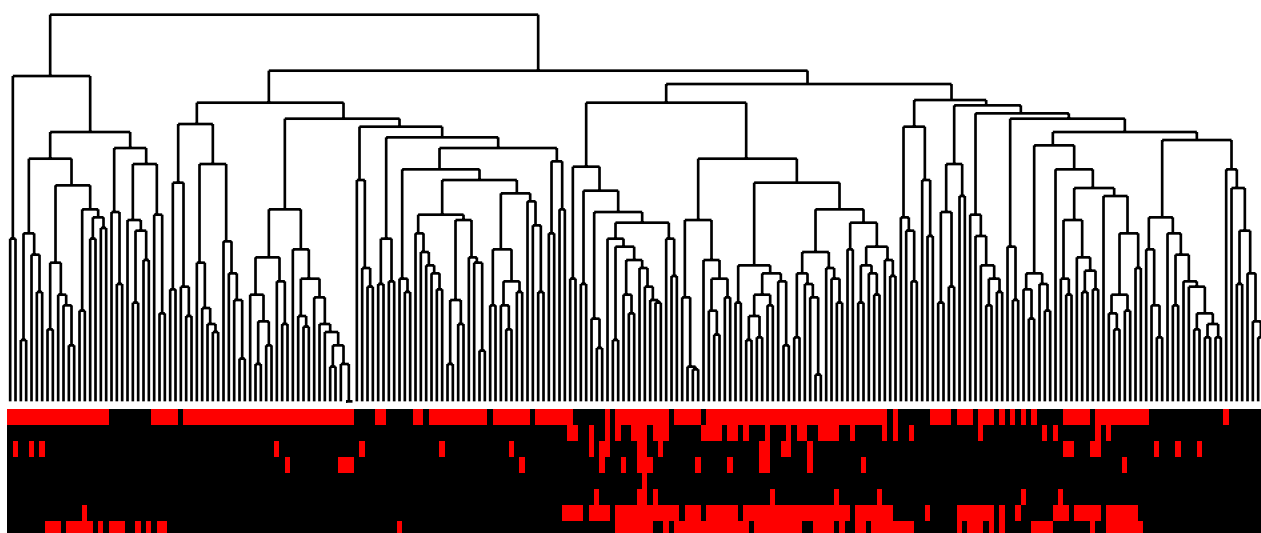

Supplement: Additional file 19: Figure S6 — Phylogenetic profiles and positions in MPD and MNTD coordinates for all modules. [file 1471-2164-14-450-S19.zip › FigureS6/skyblue1.pdf]

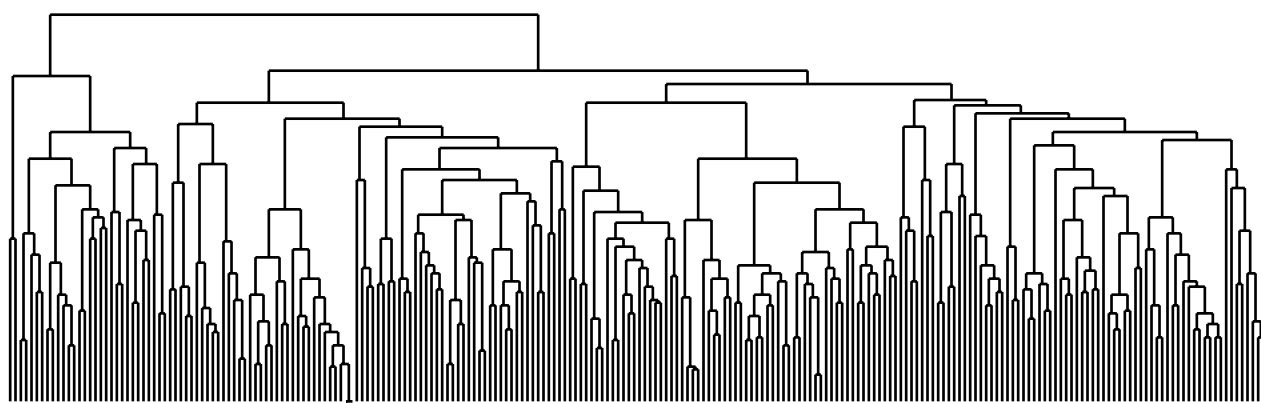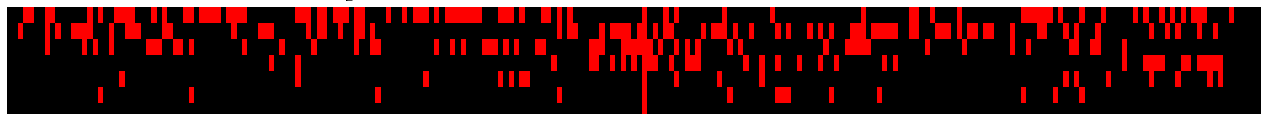

CCNA\_03681  
CCNA\_03529  
CCNA\_00744  
CCNA\_01852  
CCNA\_01931  
CCNA\_01512  
CCNA\_01142

Supplement: Additional file 19: Figure S6 — Phylogenetic profiles and positions in MPD and MNTD coordinates for all modules. [file 1471-2164-14-450-S19.zip › FigureS6/skyblue2.pdf]

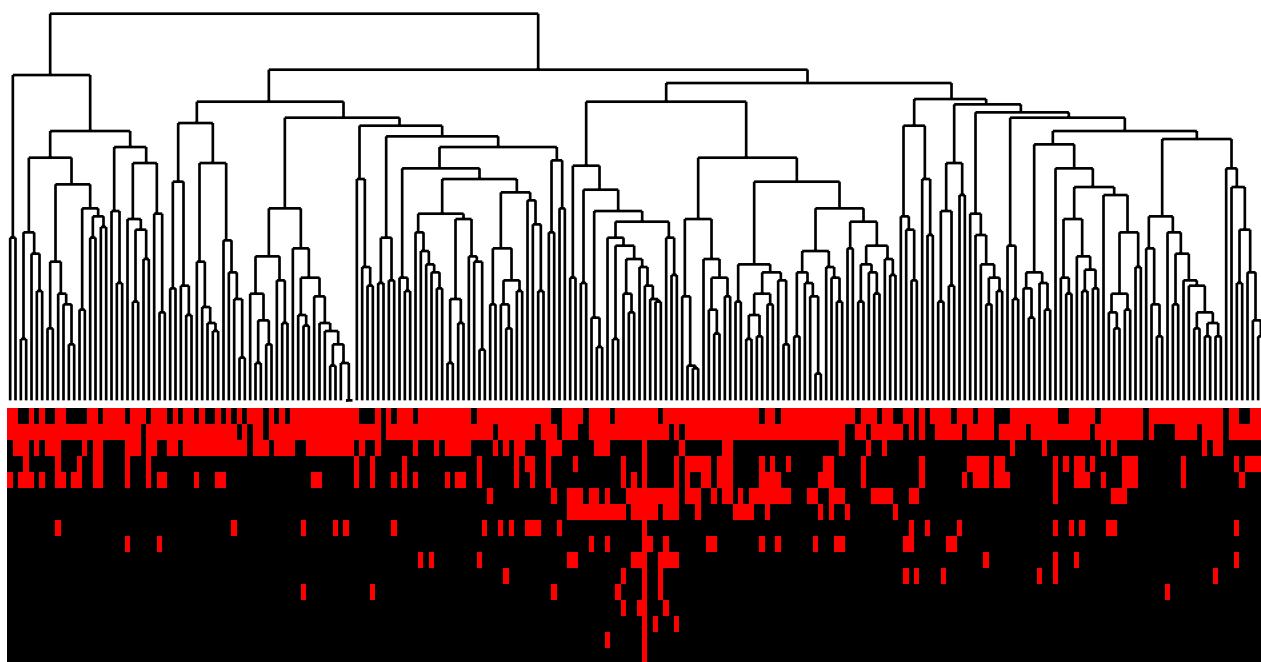

Supplement: Additional file 19: Figure S6 — Phylogenetic profiles and positions in MPD and MNTD coordinates for all modules. [file 1471-2164-14-450-S19.zip › FigureS6/skyblue3.pdf]

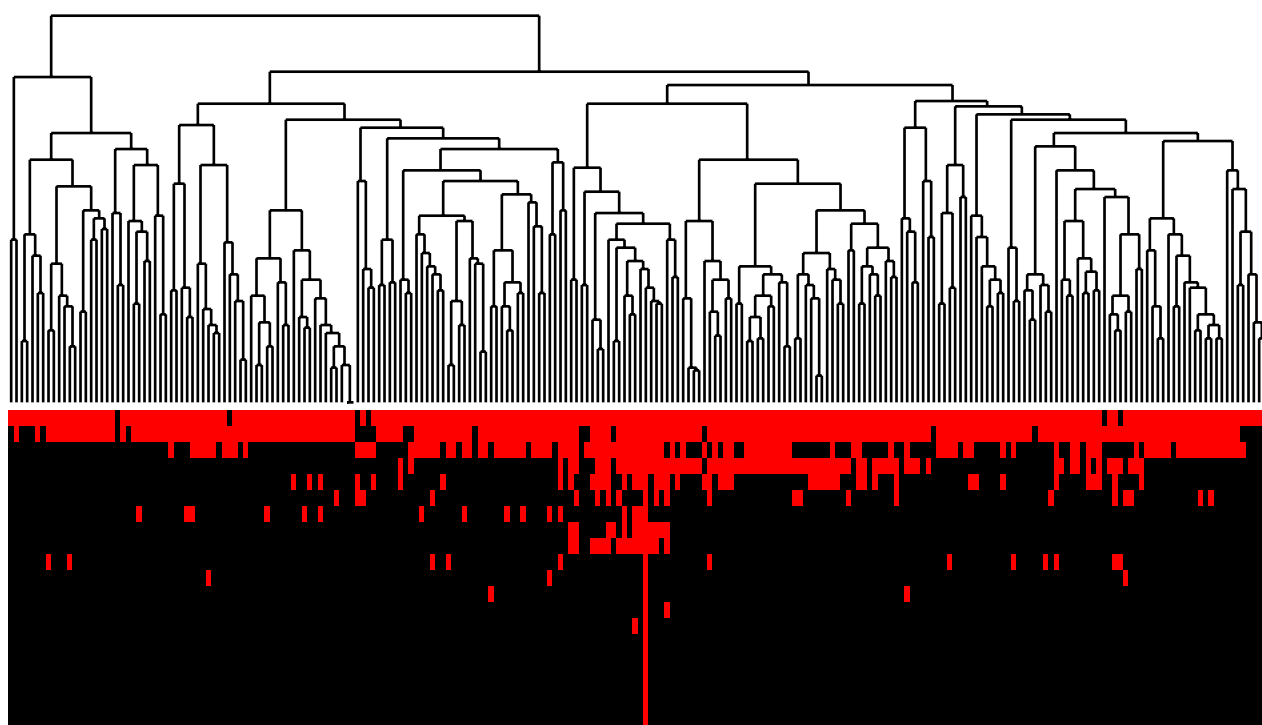

Supplement: Additional file 19: Figure S6 — Phylogenetic profiles and positions in MPD and MNTD coordinates for all modules. [file 1471-2164-14-450-S19.zip › FigureS6/steelblue.pdf]

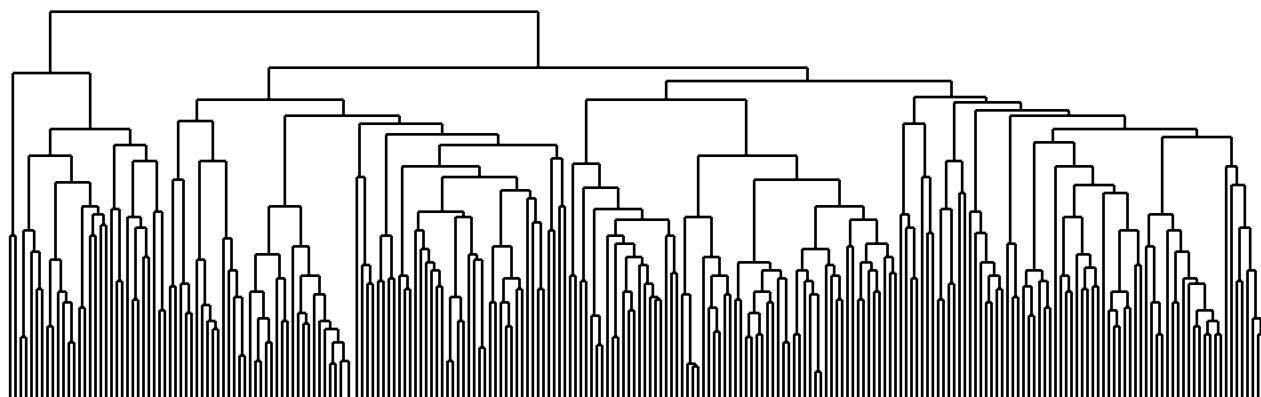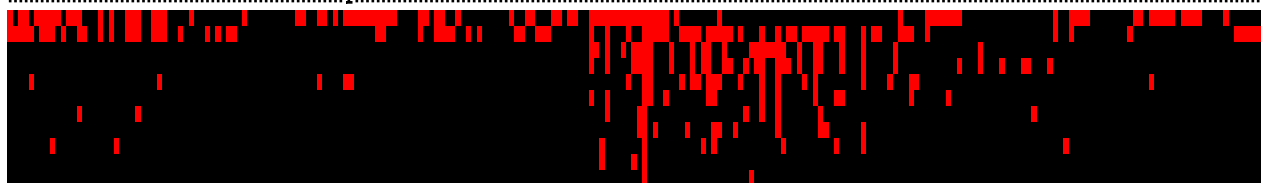

CCNA\_02011  
CCNA\_03158  
CCNA\_00028  
CCNA\_00027  
CCNA\_03159  
CCNA\_03156  
CCNA\_03022  
CCNA\_02277  
CCNA\_00138  
CCNA\_02274  
CCNA\_03065

Supplement: Additional file 19: Figure S6 — Phylogenetic profiles and positions in MPD and MNTD coordinates for all modules. [file 1471-2164-14-450-S19.zip › FigureS6/thistle1.pdf]

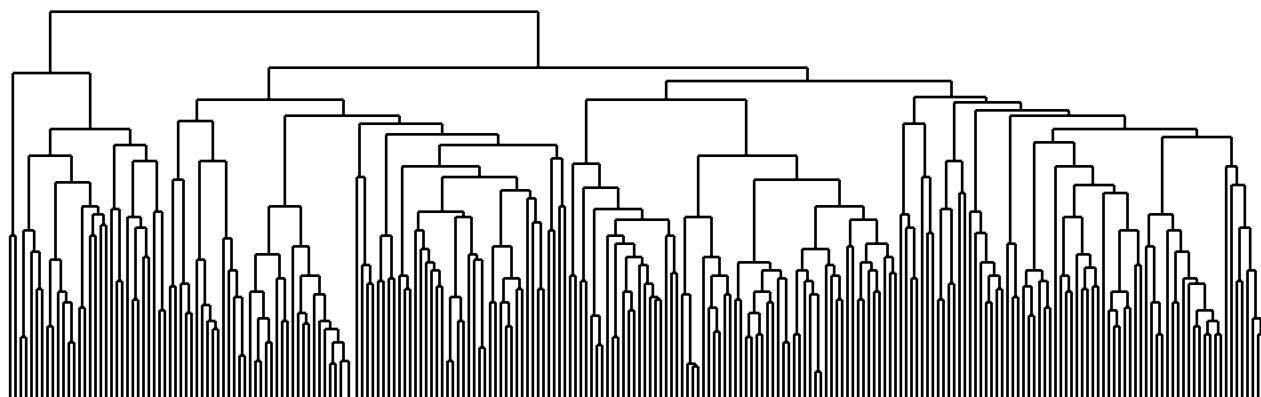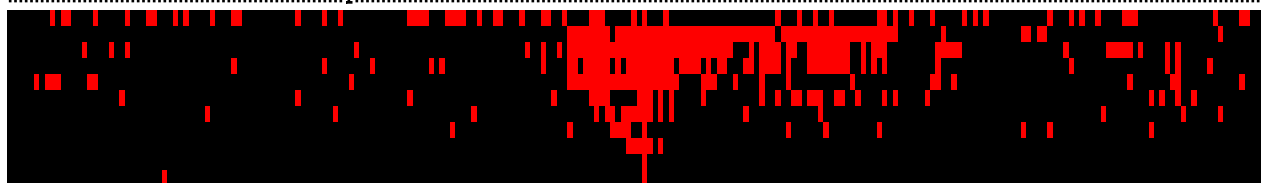

CCNA\_00748  
CCNA\_00106  
CCNA\_03786  
CCNA\_03788  
CCNA\_01417  
CCNA\_01015  
CCNA\_03515  
CCNA\_03787  
CCNA\_02850  
CCNA\_03321  
CCNA\_00589

Supplement: Additional file 19: Figure S6 — Phylogenetic profiles and positions in MPD and MNTD coordinates for all modules. [file 1471-2164-14-450-S19.zip › FigureS6/thistle2.pdf]

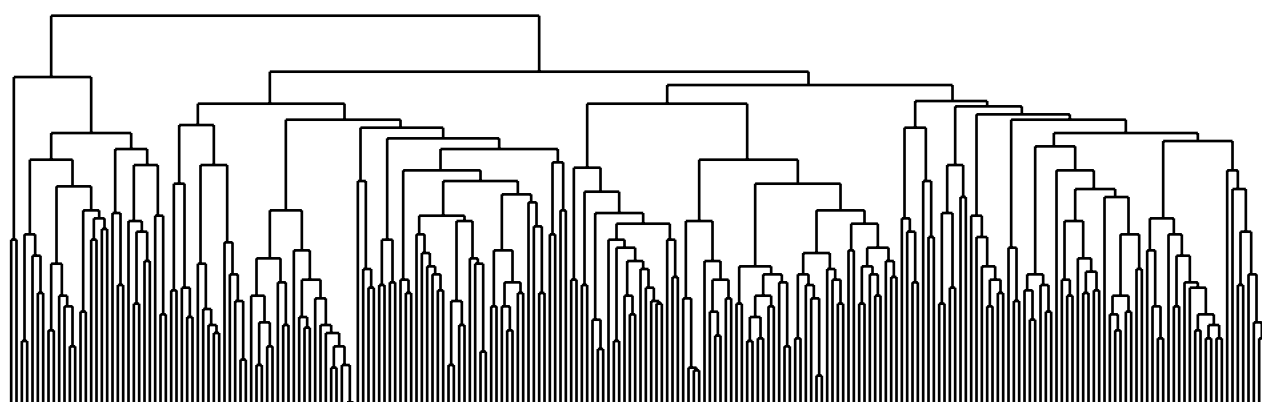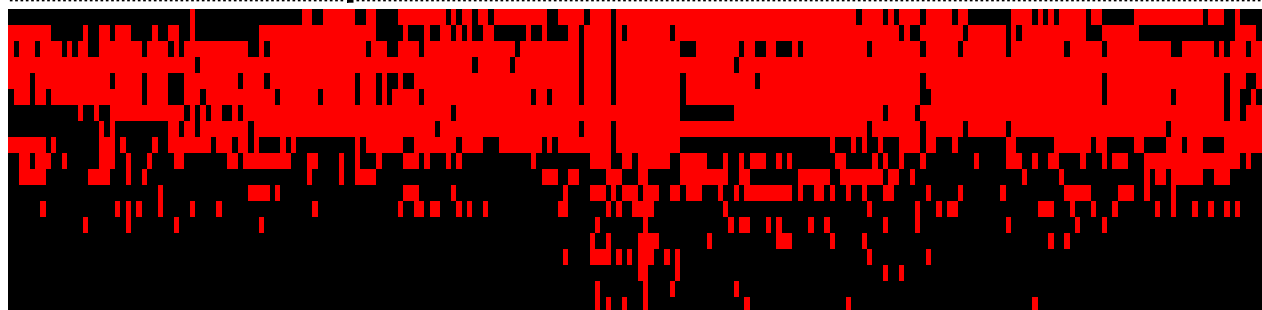

CCNA\_00151  
CCNA\_03735  
CCNA\_00128  
CCNA\_01795  
CCNA\_02994  
CCNA\_02518  
CCNA\_02325  
CCNA\_01538  
CCNA\_02344  
CCNA\_01547  
CCNA\_00130  
CCNA\_02326  
CCNA\_00282  
CCNA\_03769  
CCNA\_00365  
CCNA\_01929  
CCNA\_00210  
CCNA\_01347

Supplement: Additional file 19: Figure S6 — Phylogenetic profiles and positions in MPD and MNTD coordinates for all modules. [file 1471-2164-14-450-S19.zip › FigureS6/violet.pdf]

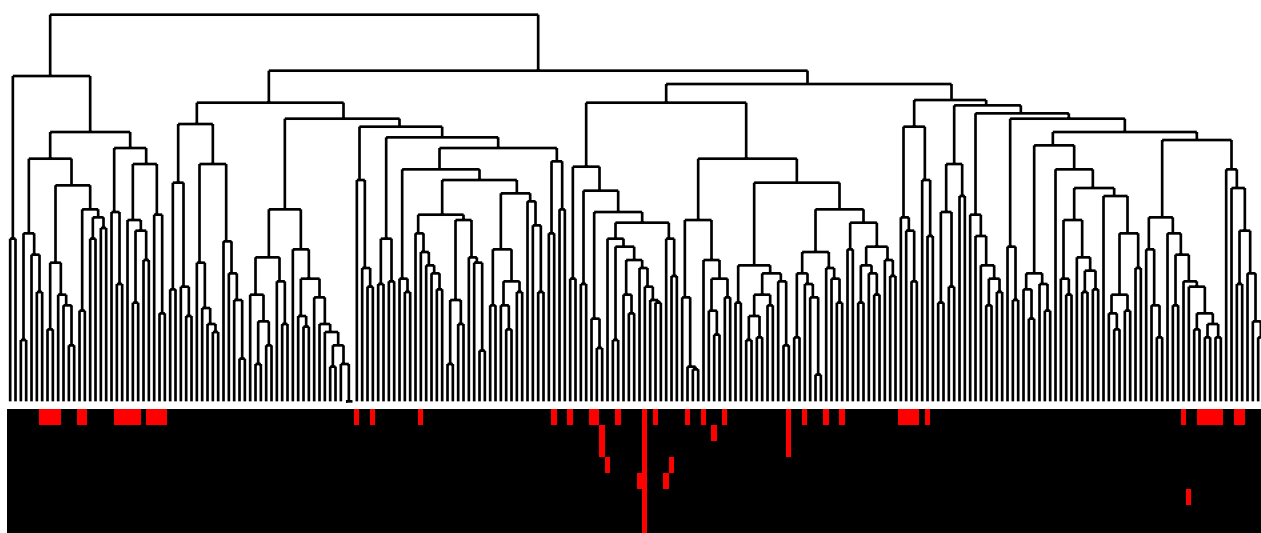

CCNA\_01703  
CCNA\_02497  
CCNA\_02496  
CCNA\_01932  
CCNA\_01426  
CCNA\_02544  
CCNA\_00542  
CCNA\_01779

Supplement: Additional file 19: Figure S6 — Phylogenetic profiles and positions in MPD and MNTD coordinates for all modules. [file 1471-2164-14-450-S19.zip › FigureS6/yellow4.pdf]

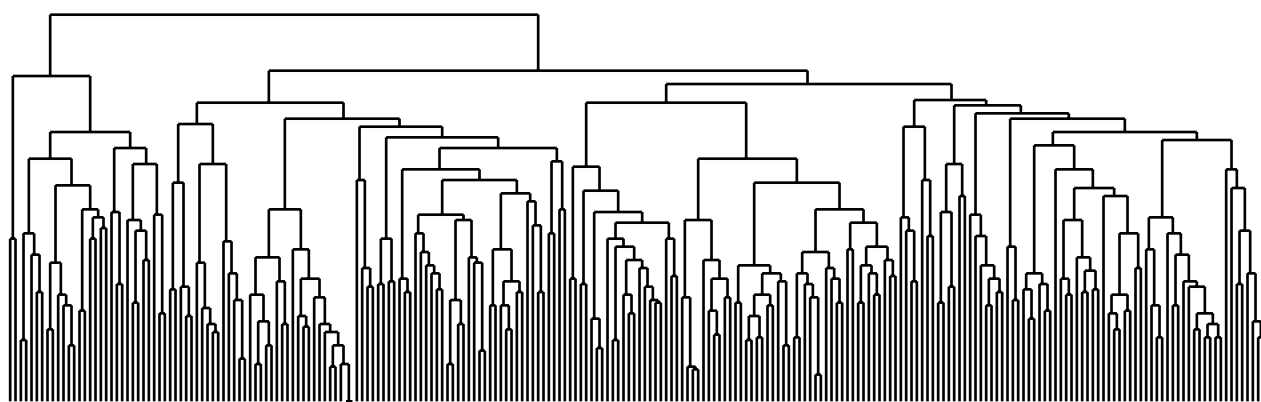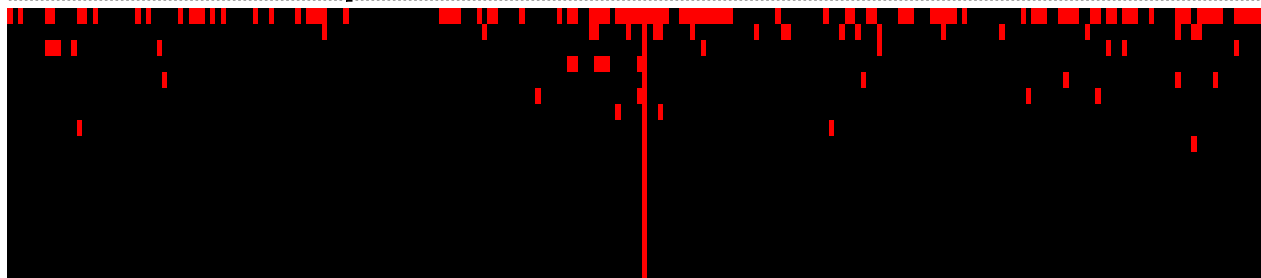

CCNA\_00876  
CCNA\_00900  
CCNA\_03171  
CCNA\_03246  
CCNA\_00860  
CCNA\_02248  
CCNA\_02257  
CCNA\_00026  
CCNA\_00081  
CCNA\_03426  
CCNA\_03490  
CCNA\_02950  
CCNA\_01302  
CCNA\_00318  
CCNA\_00450  
CCNA\_00483  
CCNA\_00728

Supplement: Additional file 19: Figure S6 — Phylogenetic profiles and positions in MPD and MNTD coordinates for all modules. [file 1471-2164-14-450-S19.zip › FigureS6/yellowgreen.pdf]
